# Optimizing experimental procedures for quantitative evaluation of crop plant performance in high throughput phenotyping systems

**DOI:** 10.3389/fpls.2014.00770

**Published:** 2015-01-20

**Authors:** Astrid Junker, Moses M. Muraya, Kathleen Weigelt-Fischer, Fernando Arana-Ceballos, Christian Klukas, Albrecht E. Melchinger, Rhonda C. Meyer, David Riewe, Thomas Altmann

**Affiliations:** ^1^Department of Molecular Genetics, Leibniz Institute of Plant Genetics and Crop Plant Research (IPK) GaterslebenStadt Seeland, Germany; ^2^Seed Science and Population Genetics, Institute of Plant Breeding, University of HohenheimStuttgart, Germany

**Keywords:** automated high-throughput plant phenotyping, Arabidopsis, maize, plant growth protocol, image analysis

## Abstract

Detailed and standardized protocols for plant cultivation in environmentally controlled conditions are an essential prerequisite to conduct reproducible experiments with precisely defined treatments. Setting up appropriate and well defined experimental procedures is thus crucial for the generation of solid evidence and indispensable for successful plant research. Non-invasive and high throughput (HT) phenotyping technologies offer the opportunity to monitor and quantify performance dynamics of several hundreds of plants at a time. Compared to small scale plant cultivations, HT systems have much higher demands, from a conceptual and a logistic point of view, on experimental design, as well as the actual plant cultivation conditions, and the image analysis and statistical methods for data evaluation. Furthermore, cultivation conditions need to be designed that elicit plant performance characteristics corresponding to those under natural conditions. This manuscript describes critical steps in the optimization of procedures for HT plant phenotyping systems. Starting with the model plant Arabidopsis, HT-compatible methods were tested, and optimized with regard to growth substrate, soil coverage, watering regime, experimental design (considering environmental inhomogeneities) in automated plant cultivation and imaging systems. As revealed by metabolite profiling, plant movement did not affect the plants' physiological status. Based on these results, procedures for maize HT cultivation and monitoring were established. Variation of maize vegetative growth in the HT phenotyping system did match well with that observed in the field. The presented results outline important issues to be considered in the design of HT phenotyping experiments for model and crop plants. It thereby provides guidelines for the setup of HT experimental procedures, which are required for the generation of reliable and reproducible data of phenotypic variation for a broad range of applications.

## Introduction

The genotype-phenotype-concept introduced by Johannson (1909) defined a phenotype as the overall constitution of an organism including all possible characteristics that can be assessed by a multitude of analytical methods ranging from morphological, physiological, anatomical traits to chemical composition. Plant phenotyping as an emerging area of science addresses the interaction of genotypes with their environment that manifests in multiple plant morphological parameters and ultimately in their accumulated biomass and yield (all together the plant phenotype). Referred to as the “phenotyping gap,” the lack of quantitative and high throughput (HT) plant phenotyping methods became more and more obvious in the last years due to the increasing demand for the development of higher yielding crops that are resource efficient and stress-resistant (Finkel, 2009; Houle et al., 2010; Furbank and Tester, 2011; Cobb et al., 2013; Fiorani and Schurr, 2013). While major advances in genotyping and sequencing technology led to readily available detailed genomic data of huge, genetically diverse plant populations such as breeding material, diversity collections, or mapping populations, the acquisition of precise and comprehensive phenotypic information needed to understand the genetic contribution to phenotypic variation has been much more demanding. Screening of such large plant populations requires methods with increased precision and accuracy in phenotypic trait acquisition paired with decreased labor input as achieved by automation, remote control and data (image) analysis pipelines amenable to HT. Nowadays the term “phenomics” refers mainly to imaging based HT procedures that employ a wide range of electromagnetic radiation wavelength bands monitored by camera sensors detecting plant-specific patterns of absorption, reflection or emission. Corresponding HT plant phenotyping systems have been set up increasingly during the last years. Two fundamentally different but complementary approaches are followed: Field phenotyping to assess trait expression usually of stands of plants under natural conditions and phenotyping in controlled (inhouse) environments in climatised glasshouses or phytochambers to monitor plant features expressed under defined conditions (Fiorani and Schurr, 2013). While the field situation is characterized by high spatial and temporal heterogeneities with very limited opportunities to modulate conditions experimentally and to reproduce experiments under the same conditions (Araus and Cairns, 2014), controlled environments offer the advantage that cultivation conditions can be set to the experimental needs of the addressed scientific questions and can be repeatedly applied to check reproducibility of observations and to extend analyses to new or further developed plant material. However, results obtained in controlled environment experiments such as traits expressed at vegetative stages or QTL and underlying genes are difficult to relate to or translate directly into yield performance under field conditions (Araus and Cairns, 2014). Thus, advances of the concepts applied to experimental setups and to the use of results obtained from phenotyping platforms in controlled environments are needed. The corresponding installation types can be grouped into sensor-to-plant and plant-to-sensor systems depending on the movement of either the camera sensors or the plants. The Phenopsis system for Arabidopsis phenotyping (Granier et al., 2006) and a pepper plant imaging facility (Van Der Heijden et al., 2012) follow the sensor-to-plant principle. Systems representing the plant-to-sensor concept have been set up at the Jülich Plant Phenotyping Center (Growscreen, (Walter et al., 2007; Biskup et al., 2009; Jansen et al., 2009; Nagel et al., 2012), at INRA Montpellier (Phenopsis, https://www1.montpellier.inra.fr/ibip/lepse/english/ressources/phenopsis.htm) (Tisné et al., 2013), GlyPH (Pereyra-Irujo et al., 2012), and at the University of Ghent (WIWAM, Skirycz et al., 2011). A small number of companies offer customized solutions for HT plant phenotyping systems, such as the LemnaTec Scanalyzer (LemnaTec AG, Aachen, www.lemnatec.de) or PlantScreen Conveyor systems (Qubit Phenomics, Kingston, Ontario, Canada, www.qubitphenomics.com). In public research institutions, LemnaTec systems have for instance been installed in Adelaide (The Plant Accelerator as part of the Australian Plant Phenomics Facility, http://www.plantphenomics.org.au/, (Crowe, 2011), at INRA Dijon and Montpellier (PPHD and Phenoarch, http://bioweb.supagro.inra.fr/phenoarch/index.php/en/), at Aberystwyth University (http://www.aber.ac.uk/en/), and at the Leibniz Institute of Plant Genetics and Crop Plant Research (IPK; http://www.ipk-gatersleben.de/en). Qubit Phenomics Trayscan systems are for instance running at the The High Resolution Plant Phenomics Center Canberra (http://www.csiro.au/Outcomes/Food-and-Agriculture/HRPPC/PlantScan.aspx), the ARC Center of Excellence in Plant Energy Biology, Acton, Australia, (http://www.plantenergy.uwa.edu.au/research/tech_platforms_main.shtml), the C4 Rice Center at the International Rice Research Institute in Los Baños, Laguna, Philippines and the Nam Laboratory for Complex Biology at the Daegu Gyeongbuk Institute of Science & Technology (DGIST) in South Korea (www.dgist.ac.kr).

The increasing number of installed systems illustrates the need for standardized plant growth protocols which maximize reproducibility and reliability of HT phenotyping experiments and ensure the ability to precisely quantify variation of trait expression. In order to do so, it is important to consider that the phenotype is the result of the interaction of genotype, environment and the phenotype (vitality) of its parents (GxExP). A sufficiently large genotypic component of the variation is required in order to uncover genotype-phenotype relationships by e.g., association studies to identify valuable polymorphisms and examine their functional significance in the context of a given (un-)favorable plant trait.

In contrast to genetic variation of the analyzed plant lines, unaccounted environmental influences should be minimized. Growth and development of plants is affected by the life cycle history of its parental generation as well as seed size (Meyer et al., 2004; Elwell et al., 2011) and seed quality (Rajjou et al., 2012) thereby adding variability to the behavior of the offspring which is possibly mediated by adaptive mechanisms contributing to plant plasticity. Divergent environmental conditions affecting the development and growth of the parental lines can be reduced by using simultaneously propagated seed material for particular experiment series. The effect of seed size on growth, which is influenced by environmental as well as genetic factors, can be accounted for by measuring seed size and by considering this value to adjust results. Seed quality can be assessed by a range of analytical procedures (Rajjou et al., 2012) which can be used to check and select good seed lots, but it would be difficult to use such data for adjustment of obeserved plant performance values. Controlling and minimizing environmental variation is therefore important not only for phenotyping experiments in HT systems (in phytochambers as well as greenhouses) themselves, but also for the preparatory seed multiplication, and represents an important and necessary requirements for the reproducible quantification of the genotype effects on plant phenotypes. The power of detection of even subtle differences in plant growth between genotypes, possibly masked by environmental inhomogeneities leading to growth variability among replicates, can be increased using different strategies. First of all, continuous monitoring of environmental conditions (such as intensity and spectrum of the incident light, CO_2_ level, air humidity and temperature, as well as soil parameters including water and nutrient availability) using respective sensors can provide detailed information about microclimatic fluctuations within the HT phenotyping system down to the single plant level. Wireless sensor networks (WSN) are widely used in order to monitor natural environmental variation in field trials (Wark et al., 2007; Bogena et al., 2010; Lee et al., 2010, http://www.csiro.au/Outcomes/ICT-and-Services/National-Challenges/Wireless-sensors-in-agriculture.aspx). Although much reduced, environmental fluctuations occur within the plant growth area of greenhouses or phytochambers, especially between central and side regions (Granier et al., 2006; Poorter et al., 2012), and should be monitored by the help of sensor networks (with different numbers and types of sensors) (Sadok et al., 2007). The obtained information can then be incorporated into adapted experimental designs with sufficient randomization and replication.

Feature extraction from HT phenotyping experiments requires automated and powerful image analysis pipelines in order to extract biologically relevant information from images, with a huge number of phenotypic traits to be quantified from a single image. A range of software applications have been developed for whole plant analysis, such as IAP (Klukas et al., 2014), PhenoPhyte (Green et al., 2012), Rosette Tracker (De Vylder et al., 2012), HTPheno (Hartmann et al., 2011), HYPOTrace (Wang et al., 2009), LAMINA (Bylesjo et al., 2008), or dedicated to plant organs such as roots RootNav (Pound et al., 2013) or leaves LeafAnalyser (Weight et al., 2008) and seeds SmartGrain (Tanabata et al., 2012). These software applications use a variety of algorithms to extract a wide range of plant architectural and physiological parameters from images acquired with dedicated camera sensors. The extracted data need to be documented with a precise description of the applied phenotyping workflow as a standardized process. This requires the documentation of image analysis procedures integrated with contextual information providing a detailed documentation of experimental metadata. For this purpose standard metadata formats have been developed such as ISATools (Rocca-Serra et al., 2010) and eXtensible Mark-up Language (XEML) (Hannemann et al., 2009) which support the integration of phenomics datasets with respective protocols, their publication, and their sharing or reuse in or via public domains (PhenopsisDB: Fabre et al., 2011; Arend et al., 2014).

Here we describe the testing and optimization of plant growth protocols adapted to the special requirements of HT plant phenotyping approaches, which conceptually differ from smaller scale experimental setups with manual or visual data acquisition. Growing large numbers of plants requires large plant growth areas wherein spatial inhomogeneities have to be reduced as far as possible. Beyond environmental uniformity plants have to be treated uniformly during automated plant handling (with respect to watering, fertilization, stress treatments etc.) and even plants with different phenotypes (e.g., different sizes), which may have different rates of resource consumption, need to be kept under comparable conditions. Furthermore, specific settings are necessary while growing and handling large numbers of plants that reduce or even avoid errors during automated data acquisition (e.g., imaging and image analysis) which is ideally run without visual checking and manual correction. Taking these considerations into account, this manuscript highlights important issues to be aware of during the implementation of HT plant phenotyping procedures as well as planning, conducting, analyzing and documenting of large-scale experiments. We provide guidelines for design and conductance of experiments to help maximizing the detection power and significance of HT plant phenotyping experiments, their reproducibility and reusability of results for future investigations.

## Materials and methods

### Arabidopsis plant material and growth conditions

*Arabidopsis thaliana* (L.) Heynh. accessions Col-0 and C24 (Meyer et al., 2004) were grown under controlled conditions at 20/18°C, 60/75% relative humidity, 130–150 μmol m^−2^ s^−1^ photosynthetically active radiation (PAR) from Whitelux Plus metal halide lamps (Venture Lighting Europe Ltd., Rickmansworth, Hertfordshire, England, see Figure S1A for spectral composition of the emitted light) and a 16/8 h day/night regime in a walk-in growth-chamber. After 2–3 days of stratification at 5°C in constant darkness, seeds were germinated and seedlings cultivated under a 16/8 h day/night regime with 16/14°C, 75% relative humidity, and 130–150 μmol m^−2^ s^−1^ light intensity until 3 days after appearance of both cotyledons [usually reached at 4 days after sowing (DAS)]. For each experiment, light intensity (PAR lite Meteon, Kipp&Zonen, Reichenbach, Germany, 400–700 nm), air temperature and relative humidity (Testo 175-H2 data logger, Testo AG, Lenzkirch, Germany) were measured manually at the plant level. Pots were filled with a mixture of 85% (v) red substrate 2 (Klasmann-Deilmann GmbH, Geeste, Germany) composed of a blend of white and frozen through black phagnum peat, pH 5.5, supplemented with lime and NPK fertilizer (280 mg/l N, 200 mg/l P_2_O_5_, 360 mg/l K_2_O, 100 mg/l Mg, 180 mg/l S, with micronutrients including chelated Fe) and 15% (v) sand and soil moisture was re-adjusted daily to 70% field capacity.

Soil water content corresponding to 100% field capacity was determined by weighing soil-filled pots after full watering and after drying for 3 day at 80°C. The weight corresponding to 70% field capacity was calculated in an analogous manner as for maize pots (see below).

One day prior to the experiment start, pots were filled with the soil mixture and watered to reach 70% field capacity. A blue rubber mat was placed as a soil cover and the pots were inserted into the carriers of the LemnaTec system where the weight of each pot was measured to determine the target weight. In the course of the experiment, the changes of weight that occurred in the intervals from 1 day to the next were used as measures of the amount of water lost from the soil and the equivalent volume of water was added through a peristaltic pump. A layer of textile material covered with a perforated black foil (to improve the background surrounding the pots in the top view images) was used in the supporting containers of the carriers to improve the water distribution. Prior to sowing, each carrier received 50 ml water pumped into the bottom container to increase the moisture during germination. Seeds were imbibed on moist filter paper for 48 h in the dark at 5°C. Thereafter, they were transferred to the soil using tooth picks. The pots were covered with plastic caps to maintain high humidity conditions during germination. These were removed after germination and development of the second rosette leaf.

### Maize plant material and growth conditions

#### Maize inbred lines

Three panels of diverse maize inbred lines were used. One panel consisted of 44 highly diverse maize inbred lines that were selected from a set of 285 Dent inbred lines from worldwide sources and four popular European flint inbred lines, which were evaluated for their biomass and bioenergy related traits in the field in three agro-ecologically diverse locations for 2 years (Grieder et al., 2012; Strigens et al., 2012). Selection of the 44 lines was done so as to represent a set of lines with maximal variation in terms of biomass production, ranging from low to high biomass yielding lines. In this study, this panel was cultivated in the HT plant phenotyping system using normal glasshouse conditions (20/25°C night/day temperatures). Due to the observed low correlation between glasshouse and field cultivation, as second panel consisting of 25 highly diverse maize inbred, with 18 selected from the first panel of 44 inbred lines and further seven inbred lines were included to widen variation observed in under field condition. Consequently, selection was done to provide a good representation of inbred lines with low, medium and high biomass production, taking into account both previous cultivations at field and glasshouse. This panel was used to optimize glasshouse cultivation conditions to closely resemble that of field cultivation conditions. A third panel, consisting of 63 inbred lines, with 19 inbred lines overlapping in one of the first two panels or in both panels, was cultivated under optimized conditions in the HT plant phenotyping glasshouse. The geographic origins and other pedigree data of the maize inbred lines used in these studies are presented in Table S1.

#### Standard cultivation conditions

*Zea mays* plants were grown in a climate controlled glass house at 25/20°C day/night, 65% relative air humidity, and 205–245 μmol m^−2^s^−1^ PAR supplemental illumination using SonT Agro high pressure sodium lamp (Philips, Amsterdam, The Netherlands, see Figure S1B for spectral composition of the emitted light) with the light period set to 16 h (06:00–22:00 h). Due to the use of shading (when outside sun light exceeded 65 klux), total light intensity (natural sunlight + supplemental illumination) only rarely exceeded 380 μmol m^−2^s^−1^ PAR. The seeds were germinated and seedlings pre-cultured in small pots (9 cm diameter) filled with substrate 2 (Klasmann-Deilmann GmbH, Geeste, Germany), composition given above, for 5 days. Thereafter, plants were transferred to 5 liter pots filled with 4 kg of an IPK soil mixture composed of 40% IPK made compost (composed of 9% organic matter, pH 6.9, with 153 mg/l N, 731 mg/l P_2_O_5_, 1259 mg/l K_2_O, 272 mg/l Mg), 40% substrate 2 (see above) and 20% sand and were further cultivated under the same conditions until harvest (37 days).

#### Optimized growth conditions

Seeds were germinated and pre-cultured in small pots (9 cm diameter) for 13 days and transplanted into 5 liter pots filled with the IPK soil mixture mentioned above and were grown for 29 more days. The plants were subjected to a temperature regime mimicking Gatersleben spring temperatures, with temperatures raised stepwise sequentially during the growth period starting with 15/10°C day/night for the first 3 weeks (including the germination and pre-culture period), then 20/13°C day/night for 1 week and finally to 25/18°C day/night temperature for further 2 weeks. During the entire cultivation period relative air humidity was set at 65% and the light period was set to 16 h (06:00–22:00 h). For illumination, HPI-T quartz metal halide lamps (Philips, Amsterdam, The Netherlands) were used (see Figure S1C for spectral composition of the emitted light). Plants were harvested at 21, 28, 33, and 42 DAS for fresh and dry biomass measurements. Furthermore, plant height measurements were taken thrice a week starting from 16 DAS.

### Manual measurement of shoot fresh and dry weights

Maize shoot fresh weight (mg) was determined on a single-plant basis by cutting the shoot directly above ground level and by measuring it using a medium-scale balance. Dry weight was measured after placing the plant material into a drying oven for 3 days at 80°C. Arabidopsis dry weight was measured using a fine-scale balance with automated recording.

### Soil field capacity determination

Ten 5-liter pots were randomly selected from ca 1600 5-liter pots filled with 5 liters of soil mixture, which were filled to be used in the entire cultivation. The soil was thoroughly watered to saturation and weight was taken after 3 h, when there was no sign of water dropping at the bottom of the pot. The soils were then allowed to drain for 3 days after soil saturation in darkness and weights were taken, which are equivalent to soil water holding or field capacities. The soils were then dried for 7 days in an oven at 70°C, until complete dryness. Data derived from the above soil water moisture status were then used to compute estimates of the gravimetric water content (θg) and the amount of water needed to be added to establish specific field capacities. Soil moisture sensors were used to analyze the same pots, taking the mean of 5 random points per pot, at the same time. The evaporative water loss was determined by filling pots with a soil mixture adjusted to the desired relative soil moisture and covered with blue rubber mats. The pots were weighed every day and the reduction of pot weight was used to calculate relative water losses.

### Image acquisition and analysis

In automated plant transport and imaging systems (the IPK LemnaTec Scanalyzer systems for small and for large plants), top and side view images are taken of the visible range of the light spectrum (VIS), of fluorescence signals (FLUOR), and of a broader band of the near infrared spectrum (NIR). VIS images are taken using piA2400-17gc CCD cameras (Basler, Ahrensburg, Germany) with top and side illumination of plants through incandescent bulbs (FQ 24W 865 HO or FH 28W 865 HE, respectively, Osram GmbH, München, Germany). scA1400-17gc CCD cameras (Basler, Ahrensburg, Germany) equipped with filters that pass light of ca. 540 nm and longer wave lengths are used for the acquisition of FLUOR images, where plants are illuminated with blue light from incandescent bulbs (FQ 24W 865 HO or FH 28W 865 HE, respectively, Osram GmbH, München, Germany) that passed a plexiglass filter that blocks light of 525 nm or longer wave lengths. NIR images are taken with a NIR-300 PGE camera (VDS Vosskühler GmbH, Osnabrück, Germany) equipped with a 1400–1510 nm (max.) band pass filter and using halogen lamps (Sylvania Hi-Spot Superia ES50 35W) for illumination.

The IAP (Integrated Analysis Platform) open-source software for high-throughput plant image analyses (Klukas et al., 2014) was used for image-based plant feature extraction. The implemented analysis pipelines for Arabidopsis and maize plants have been optimized with regard to plant architectural features of these two species. The processing pipelines are arranged in a block-based manner and can be divided into four main steps: (i) pre-processing, (ii) segmentation, (iii) feature extraction and (iv) post-processing. Values for projected leaf area (for *Arabidopsis*) were calculated from images taken in the visible light spectrum and correspond to the number of detected foreground pixels after the foreground/background separation. For Maize, a volume estimation is used as a proxy for the estimated biomass of the plants. The volume is calculated from the projected side and top area of the plant foreground pixels, according to the following formula: volume_IAP = Sqrt (side projected leaf area^∧^2 ^*^ top projected leaf area).

Please refer to the online documentation (http://iap.ipk-gatersleben.de/documentation.pdf) for detailed descriptions of the phenotypic traits.

### Standardized representation of experimental metadata

The IsaTab “Investigation” (the project context), “Study” (a unit of research) and “Assay” (analytical measurement) describes experimental metadata and experimental procedures for data acquisition thereby linking contextual information with the experimentental results (Rocca-Serra et al., 2010). This standardized metadata format was used here for the representation of an integrated analysis comprising high-throughput plant phenotyping, metabolite profiling and manual measurements of growth parameters of Arabidopsis plants grown under different conditions (stationary vs. rotating and covered vs. uncovered). Respective IsaTab file and associated raw and derived data files are published under: http://dx.doi.org/10.5447/IPK/2014/4.

### Statistical analyses

Statistical analyses of the Arabidopsis experiments were performed using the SPSS software package version 20 (IBM), GenStat 16th Edition, and R. The variation in dry weight, biomass, water loss and positional effect were analyzed using ANOVA, Student's *t*-tests, General Linear Model analysis (GLM) and subsequent *post-hoc* analysis (Tukey's range test) and Principal Component Analysis (PCA). For statistical evaluation of the maize experiments the PROC GLM procedure of the software SAS (SAS Institute, 2004) was used. The effects of genotypes were assumed as fixed while the effects of replicates, seasons and position of the experimental unit as random. Pearson correlations were calculated for correlation analyses.

### Metabolite profiling

Two rosette leaves per plant were harvested at 30 DAS (including 3 days of vernalization) from the *Arabidopsis thaliana* genotype C24 directly after the last cycle of movement (corresponding to 12 h after onset of illumination) by shock freezing rosette tissue in liquid N_2_. The material was homogenized at −80°C using a mill (Retsch, Haan, Germany). Fifteen mg deep frozen plant material was extracted, in-line derivatized and analyzed as described previously (Riewe et al., 2012) using an MPS2-XL autosampler (Gerstel, Mühlheim, Germany) and a 7890 gas chromatograph (Agilent, Santa Clara, CA, USA coupled to a Pegasus HT mass spectrometer (Leco, St. Joseph, MI, USA). Analyte mass spectra were deconvoluted using ChromaTOF software (Leco, St. Joseph, MI, USA) and annotated by querying the Golm Metabolome Database (GMD, http://gmd.mpimp-golm.mpg.de/). Quantitative information of 262 analytes of which 53 were identified was extracted using the R software package TargetSearch (Cuadros-Inostroza et al., 2009). Prior to statistical analysis using ANOVA, data were log10 transformed to achieve normal distribution and outliers (outside median ± 2 × SD) were removed (Steinfath et al., 2008). Normalized raw data is available under the: http://dx.doi.org/10.5447/IPK/2014/4 (“PeakTable_weight_normalized_outliercorrected”).

## Results

### Establishment of cultivation protocols for *Arabidopsis thaliana* in a high throughput plant phenotyping system

#### Features of the automated transport and imaging system for small plants

**Figure 1:**
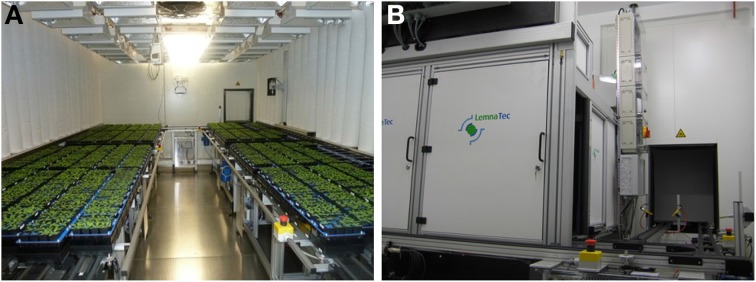
**Automated high-throughput cultivation and imaging system for small plants**. Phytochamber **(A)** and imaging chamber with weighing/watering station **(B)**.

**Figure 2:**
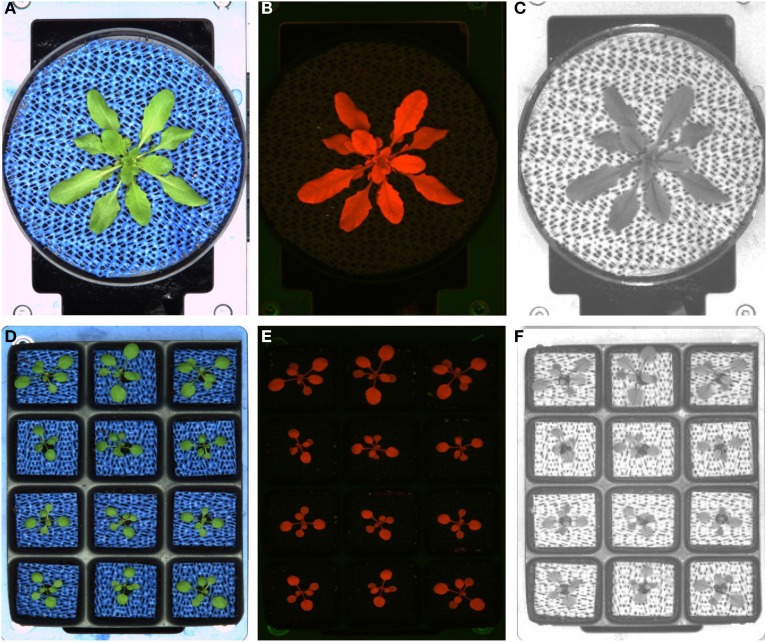
**Two different possible pot configurations used in the small LemnaTec Scanalyzer system**. Single pot **(A–C)**, 12-well tray **(D–F)** and exemplary images acquired with the three different camera sensors used in the automated HT screening system for small plants: imaging in the visible light spectrum **(A,D)**, fluorescence imaging **(B,E)**, imaging in the NIR light spectrum **(C,F)**.

The IPK LemnaTec Scanalyzer system for small plants combines growth under well controlled environmental conditions in a growth-chamber with non-destructive trait assessment of up to 4600 Arabidopsis plants in imaging chambers (Figure 1). The growth-chamber is equipped with conveyor belts loaded with 384 carriers. Each carrier is designed to contain either a single pot, a 6-well tray, or a 12-well tray for plant cultivation (Figure 2). The different pot configurations determine the maximum number of plants to be analyzed in one experiment (384, 2304 or 4608 plants, respectively), the soil volume accessible for the single plant, and therefore the cultivation time (duration of an experiment).

Plants can be sequentially moved through all positions as groups of eight carriers (blocks) or as single carriers using the pot-by-pot configuration (full rotation mode) up to 12 times every 24 h. For imaging, carriers are transported to three fixed image stations and the watering/weighing station. The non-invasive image acquisition is carried out with three different camera systems taking images in the visible wavelength range (VIS), near-infrared (NIR) and fluorescence (FLUOR) images of each carrier. It is possible to record the objects from the top and several side views. This supports the assessment of architectural traits, colorization-related traits, and measures related to the water content (NIR) or levels of fluorophores including chlorophyll (FLUOR). The recorded images and weight data are combined with the unique plant-ID information and stored on a special server for further data management and analysis using the Integrated Analysis Platform (Klukas et al., 2014).

Protocols for cultivation and characterization of Arabidopsis plants are well established and are in routine use in many labs operating rather small-scale experiments. A quite challenging task is the precise and simultaneous phenotyping of large populations of individuals. To perform large-scale experiments existing methods have to be adapted and optimized to the different demands of HT procedures like experimental design, plant cultivation and high quality imaging.

#### Optimization of the watering regime

In the automated plant phenotyping platforms watering is performed at the watering/weighing station using peristaltic pumps that supply water or nutrient solutions either as a pre-defined fixed volume or as an individually calculated amount as the difference of a carrier (incl. pot) weight to a pre-defined target weight. According to the applied watering regime, which may differ from pot to pot and from day to day, the target weight can be used for calculating and creating the watering jobs for each day or even several times per day or less than once per day. In order to optimize the watering regime for Arabidopsis growth in the different pots, several combinations of dispensing water to the soil surface (the covering rubber mat, see below), = “top watering,” or of pumping it into the bottom container of the carrier, where the solution is “stored” in the non-woven mat and soaked into the pot or the tray via capillary force, = “bottom watering,” were evaluated. The watering option(s) (top and/or bottom watering) have to be chosen according to the special requirements of each plant species and the developmental stage. Based on experience from tests with exclusive top or bottom watering combined with or without growing plants, we concluded that top watering was generally preferable over bottom watering: It results in a better moisture distribution in the soil, allows the precise application of small amounts of water directly available to the plants (e.g., for defined levels of water deprivation), and supports a more precise calculation of the plant's water use. On the other hand, this configuration may result in formation of mineral deposits and algal growth on the soil surface area. This affects the image background quality seen as particle fluorescent signals, but this disadvantage can be overcome through the use of special soil covers (see Section below). At very early stages of cultivation, Arabidopsis seedlings are sensitive to very wet soil surfaces and also to mechanical forces that occur from the water flow on the top surface. Bottom watering is therefore generally preferable during the early phase of an experiment. Thus, a combination of top and bottom watering settings was found to be optimal for growing single plants in pots. During the germination phase until the two leaf stage, pots were covered with plastic caps in order to keep high air humidity levels. During that time bottom watering is done every second day. After removal of the caps, watering is generally switched to daily top watering and is set to maintain soil moisture at 70% field capacity.

For growth of multiple Arabidopsis plants in trays (6-well or 12-well tray), only bottom watering ensures water supply to each well of the tray in a homogeneous fashion. During the germination phase until 6 DAS, the trays are covered with perforated plastic foil and the watering regime is carried out as described previously for the single pots. After removal of the cover foil, bottom watering is continued but switched to daily intervals.

#### Test of soil covers

**Figure 3:**
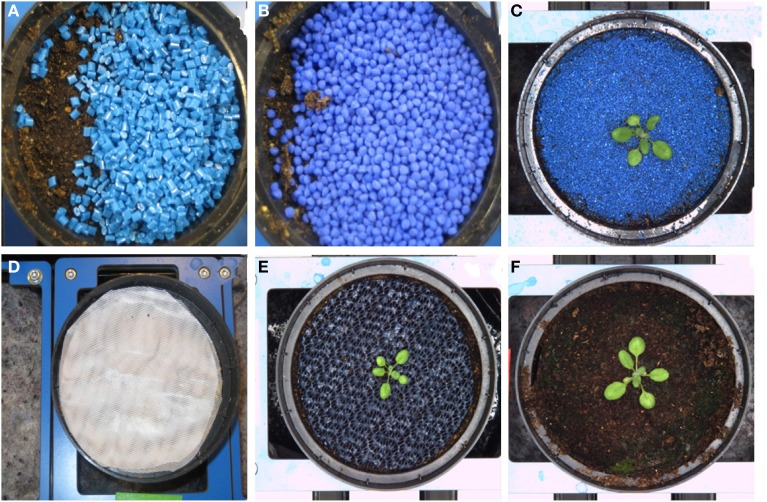
**Different soil cover materials tested in order to improve background/object contrast and to reduce evaporation**. **(A)** Blue plastic granules **(B)** Blue round gravels **(C)** Blue sand fixed with hair spray **(D)** fine-meshed mosquito net layer **(E)** perforated blue rubber mat **(F)** black substrate soil (without cover).

Further optimization of the HT phenotyping process involved the test of different soil cover materials, primarily for the purpose of image quality improvement. During automated HT image analysis, it is of utmost importance to avoid errors or incorrect calculations caused by suboptimal segmentation, which is used to separate the plant from the background. Furthermore, soil covers are advantageous in terms of homogenization of cultivation conditions as they can reduce evaporation, which could otherwise cause different soil moisture due to different timing of watering. Thus, soil covers ensure to keep the water status of the plants (the soil moisture) as uniform as possible with only one or two watering cycles per day. However, covering materials must not have any negative effects on the physiological status of the plants (e.g., by releasing compounds with negative effects into the soil or by causing hypoxia to the roots). Several cover materials were tested such as plastic granules, round gravels, sand, in bulk or fixed with hair spray, lac, fine-meshed mosquito net layer and perforated rubber mat (Figure 3) which differ in their suitability with respect to different aspects such as color, stability on a moving (starting and stopping) system, display in the image and effectiveness in reduction of water evaporation. Blue materials were found to be the best choice in terms of image quality as the blue color does not naturally appear in plants and was used to support a better segmentation of object and background.

**Figure 4:**
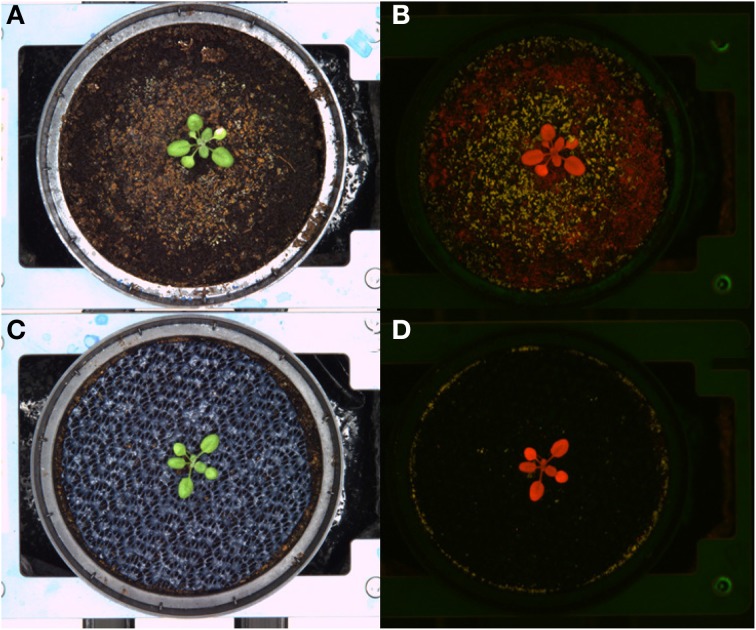
**Images of Arabidopsis plants grown in black substrate soil uncovered (upper panel) or covered with blue rubber mat (lower panel)**. Visible light **(A,C)** and fluorescence images **(B,D)** are shown.

During the rotation modus the carriers are transported from one position to the next and stopped at a stopper or directly adjacent to the previous carrier. Granules, gravels or sand, in bulk or fixed with hair spray or with lac, were not useful for our requirements. They moved with the direction of the movement, were partly falling off the carriers onto the conveyor belt and left part of the soil surface uncovered. Using a fine-meshed layer (mosquito net) or a rubber mat appeared to be more promising. These materials, the blue rubber mats in particular, did not shift positions during the rotation process, and did not interfere with germination or seedling establishment. Furthermore, the blue rubber mat strongly improved the image background and supported a clear object-background segmentation of images acquired in the visible spectral range. The improvement in segmentation was even more pronounced for the fluorescence images (Figure 4).

**Figure 5:**
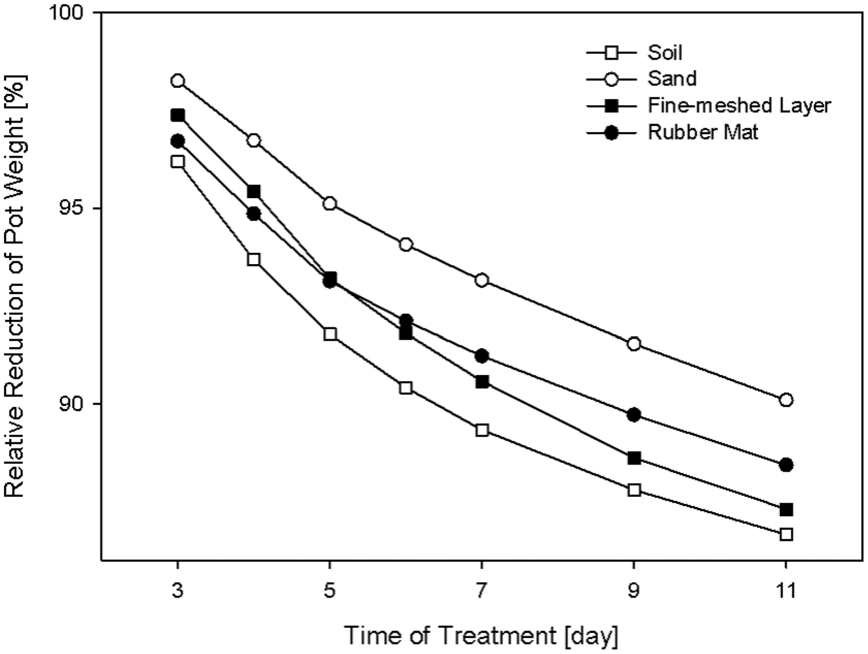
**Relative reduction of pot weight over a time period of 11 days using pots covered with rubber mat, fine-meshed net, or sand, and the uncovered version with black substrate soil**.

Three of the different cover materials (perforated blue rubber mats, sand and fine-meshed mosquito net) were also evaluated regarding their effectiveness in reducing the water evaporation of the soil. Over the time period of 11 days, soil-filled pots with the respective covers were weighed daily and the relative loss of water was calculated (Figure 5). Uncovered pots with black substrate soil were used as controls. All pots started with the same initial weight set to 100%. After 3 days of measurement clear differences in weight (water loss) were observed. All the pots show a consistent daily water loss over the time period of treatment. The maximum difference of water loss of 5% was found in sand-covered pots compared to uncovered pots, suggesting the sand-cover was most effective in controlling water loss. However, granular materials were disadvantageous due to their movement during the rotation process. The pots covered with blue rubber mat reached a difference in pot weight reduction of up to 3% compared to uncovered pots. However, further improvement may be advisable (e.g., by combination of multiple layers of the same or different cover material) if HT water-use-efficiency phenotyping experiments are intended, which require precise control of the soil water status.

**Figure 6:**
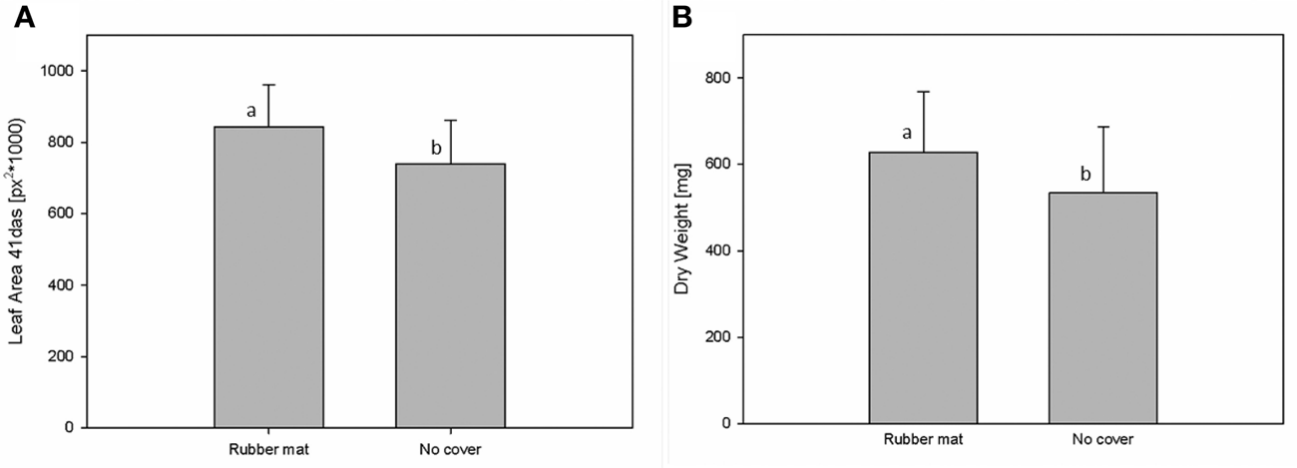
**Measurements of leaf area (A) and shoot dry weight (B) of plants that were either grown in pots covered with blue rubber mats (rubber mat) or uncovered (no cover)**.

Potential influences of the cover material on plant development were assessed by HT phenotypic and metabolic analysis of Arabidopsis plants grown either in uncovered pots or pots covered with perforated blue rubber mats. Plants of the Arabidopsis accession C24, with a replication of *n* = 192 for each treatment, were grown under standard cultivation conditions until 44 DAS. Imaging was done daily and projected leaf area was extracted from images (as number of pixels) at 41 DAS. Additionally, the shoot dry weight was manually measured after 44 DAS. Analysis of variance (Two-Factor ANOVA; *p* ≤ 0.001), considering the cover status as an influencing factor, indicate that plants grown in pots covered with rubber mats had significantly higher dry weights (627.5 ± 143.6 mg) and leaf areas (582.05 ± 153.9 px^2^) compared to plants of uncovered pots (533.7 ± 153.0 mg; respectively 542.03 ± 133.1 px^2^) (Figure 6). A significant correlation (*r* = 0.853; *p* ≤ 0.001) between dry weight and projected leaf area was observed.

The utilization of the blue rubber mats to reduce background effects may affect plant metabolism by several ways, such as the release of negative effect substances to the soil/plant or by reducing gas exchange between soil and air (potentially causing hypoxia). We tested potential physiological effects of the cover material by subjecting shoot material of covered/uncovered plants to GC-MS analysis. Seventeen replicates of each factorial condition (covered/uncovered) were harvested by shock freezing rosette leaves in liquid nitrogen at 30 DAS and subjecting them to GC-MS analysis. Fifty-three metabolites of known chemical structure and 209 metabolites of unknown chemical structure were quantified (File S1). Coefficients of variation ranged from 11 to 118%, the median was 37% and the inter quartile range 16% (Table S2). In line with the ANOVA results (Table S2), we found no evidence for a treatment effect on metabolite abundance variation using principal component analysis (PCA, **Figure 8**).

#### Check of plant movement effects

**Figure 7:**
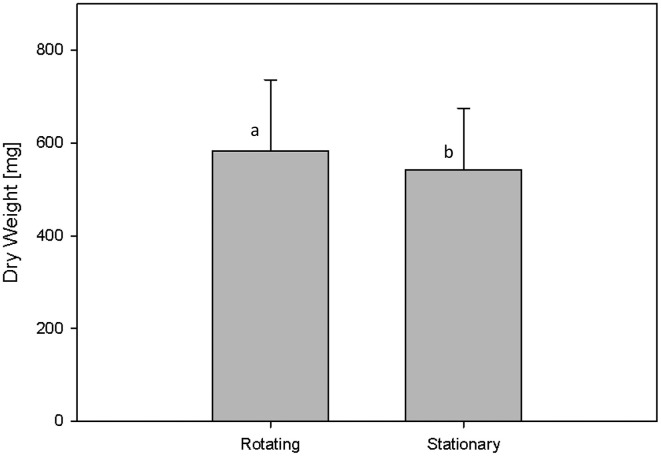
**Shoot dry weight of C24 plants that were either grown on the rotating HT phenotyping system or stationary inside the same phytochamber**. Rotating plants showed a weak but significantly increased biomass compared to stationary plants (as by Two-Factor ANOVA; *p* ≤ 0.001).

In the used HT plant phenotyping system imaging is performed according to the plant-to-sensor principle and plants are regularly moved on conveyor belts for the purpose of imaging, weighing and watering. In order to analyze possible effects of plant movement on plant growth behavior an experiment was designed for the analysis of biomass development and metabolite composition in Arabidopsis C24 plants grown for 28 days under either rotating or stationary conditions, whereas plants of both conditions grew under identical environmental conditions in the same phytochamber. Rotating conditions comprised four rotation cycles per day: (1) during the 8 h dark phase (0:00–8:00 am) movement of blocks of eight carriers every 10 min (48 block movements = one full rotation) inside the phytochamber without imaging and watering; (2) from 8:00 to 10:00 a.m. continuous one-by-one carrier movement inside the phytochamber without imaging and watering; (3) block wise movement of all carriers through the imaging chambers (with top view imaging) and the watering/weighing station (10:13 a.m. to 1:11 p.m.); and (4) block wise movement of all carriers every 13.5 min without imaging and watering (until 12:00 pm). For the comparison of biomass development of plants grown under rotating and stationary conditions (no imaging) manual measurements of the shoot dry weight were performed indicating that plants grown on the rotating phenotyping system performed slightly better with respect to dry weight (582.05 ± 153.39 mg) compared to the stationary plants (542.03 ± 133.1 mg) (Figure 7).

**Figure 8:**
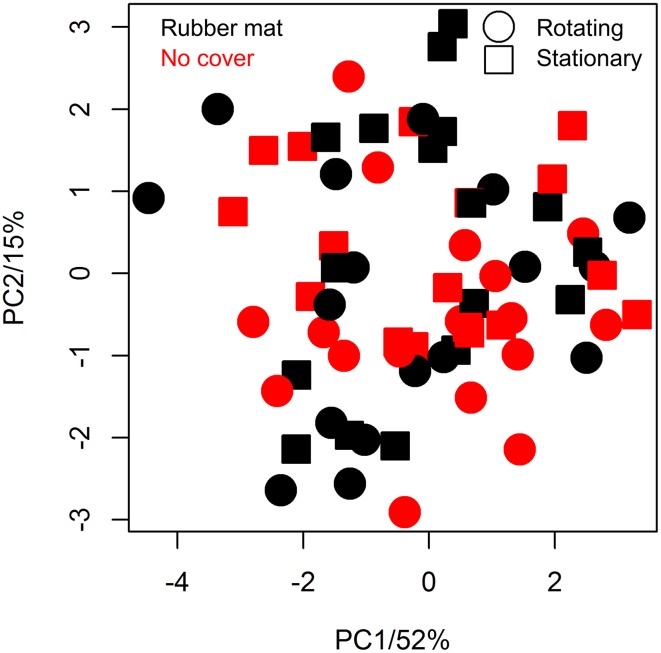
**Principal component analysis (PCA) of metabolic variation of differentially cultivated plants**. Metabolite profiles of plants that were either grown on the LemnaTec phenotyping system (points) or stationary (squares), either covered with blue mats (black) or not (red) were pareto normalized and centered prior PCA (*n* = 17 per condition).

The usage of a conveyor belt transportation system in plant phenotyping may be regarded as an environmental factor, though plants are frequently stimulated mechanically in nature (by wind, rain, etc.), and could result in changes in metabolism even though morphological changes are not detectable with the methods available. Metabolite analysis was performed analogously as described above for the comparison of plants cultivated with/without soil cover. Having identified the same number of known/unknown metabolites, no metabolite was found to be significantly altered on response to movement (File S1). The plants grown on the rotating conveyor belt system only had marginally lower median relative standard deviations in metabolite abundance than those grown stationary (35.8 and 36.3%, respectively) (Table S2). PCA revealed no effects of treatment (Figure 8).

#### Environmental inhomogeneity within the cultivation and improvement of experiment design

**Figure 9:**
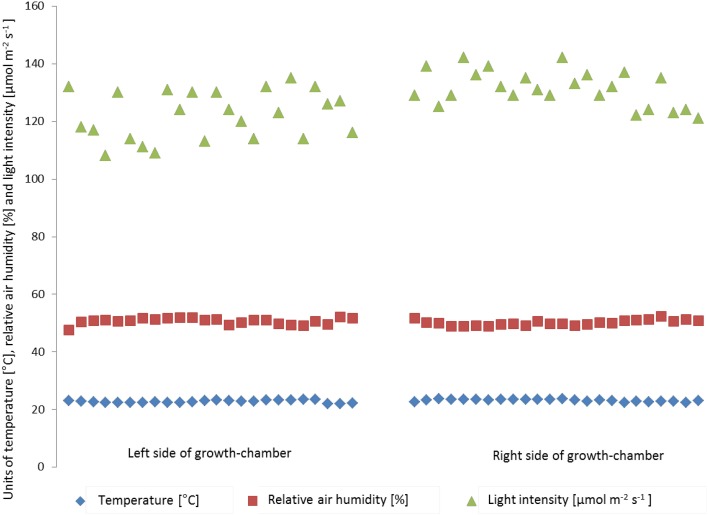
**Variation in air temperature (blue), relative air humidity (red) and light intensity (green) within the HT phenotyping phytochamber**. Air temperature and relative air humidity are stable within the phytochamber (no side effects), whereas the right side of the phytochamber shows a clear trend toward higher light intensity compared to the left side.

Inside the high throughput plant phenotyping systems, environmental conditions (temperature, light intensity and air humidity) are monitored by single sensors for automatic climate control (Johnson Controls Systems & Service GmbH, Mannheim, Germany). In order to check the effects of environmental inhomogeneity on plant growth, light intensity, temperature and air humidity were measured manually in different positions of the cultivation area (one measurement point for each of the 8-carrier blocks, 48 measurement points inside the phytochamber) and relations to plant shoot dry weight and rosette diameter were tested. One hundred and ninety two individuals of the Col-0 accession of Arabidopsis uniformly distributed throughout the system were grown under standard conditions and automated watering and imaging was performed daily. Before the start and during the experiment micro-environmental parameters were quantified on plant level at several points of the growth-chamber (Figure S2). Temperature (ranging from 21.9°C to 23.7°C) and relative air humidity (ranging from 47.5 to 52.4%) varied spatially, but no particular hot spot areas inside the phytochamber were detected for these parameters. The mean values did not vary significantly between left and right side, corner and central areas or the lanes per side. However, the mean values of the light intensity significantly differed between the right (131.4 ± 6.2 μmol m^−2^ s^−1^) and the left side (122.1 ± 8.4 μmol m^−2^ s^−1^) of the phytochamber (One-Way ANOVA, *p* < 0.05) (Figure 9). This difference was detectable as side effect but not as effect between the lanes per side. The enhanced light intensity on the right side of the growth-chamber might influence the plant growth and induce a higher biomass, leaf area and plant height. Therefore, shoot dry weight was measured manually after 28 DAS. Furthermore, the projected leaf area (in pixels) was extracted from VIS top view images of the same day.

**Figure 10:**
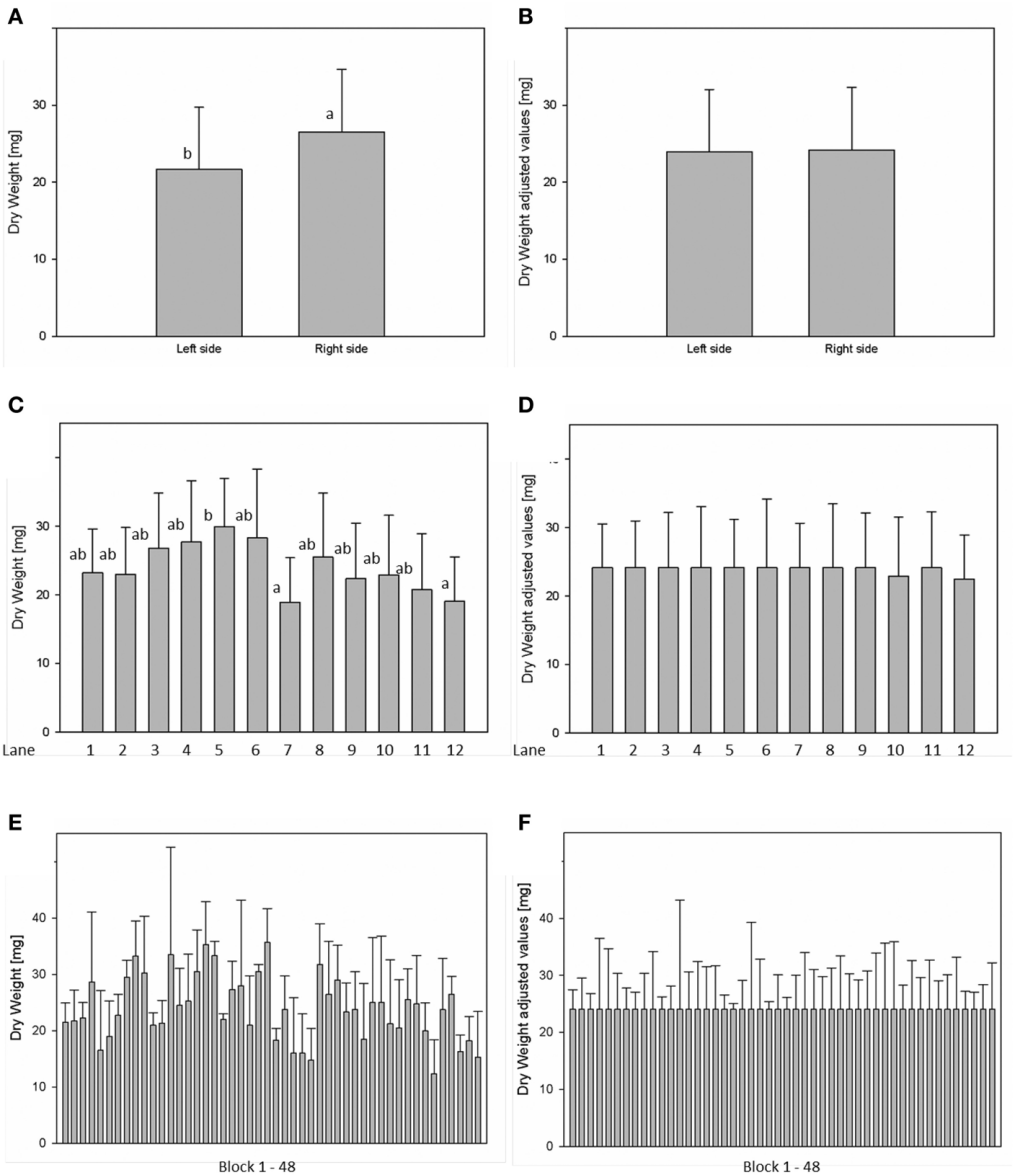
**Analysis of variance (ANOVA) of Arabidopsis shoot dry weight (A,C,E) at stage 28 DAS including environmental influences as side, lane and block effects**. Estimation of adjusted means accounting for the environmental inhomogeneity inside the growth-chamber: side **(B)**, lane **(D)**, and block **(F)**.

**Figure 11:**
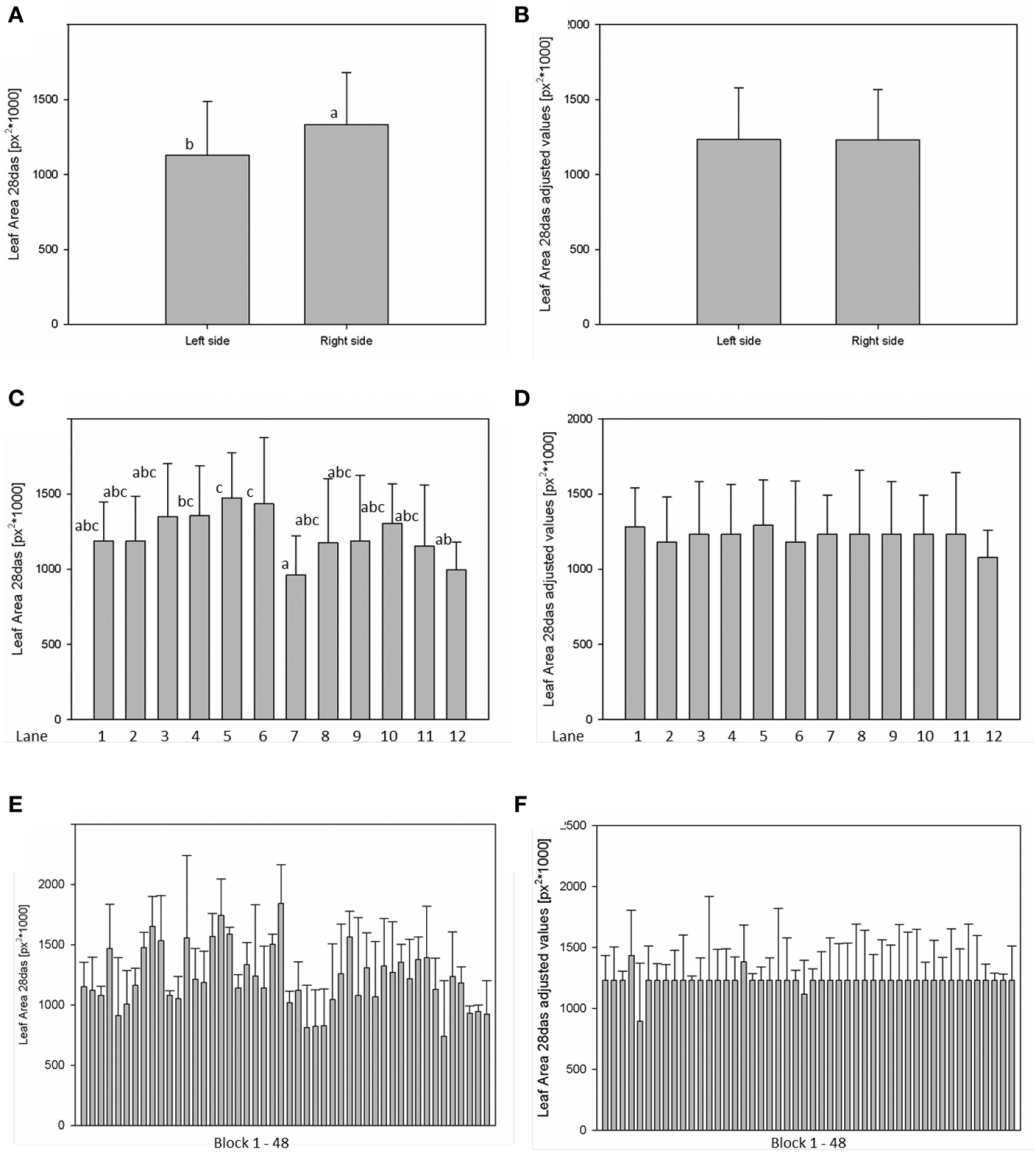
**ANOVA of Arabidopsis projected leaf area (A,C,E) at stage 28 DAS including environmental influences as side, lane and block effects**. Estimation of adjusted means accounting for the environmental inhomogeneity inside the growth-chamber: side **(B)**, lane **(D)**, and block **(F)**.

For analysis of spatial effects, several subdivisions of the cultivation area were considered and three possible factors (side, lane, and block) were tested (Figures 10, 11). The factor “side” was used to test and to correct for influences between the left and the right side of the chamber. The factor “lane” was used to consider potential influences of positions in the 12 lanes, lane number 1–6 on the right side and lane number 7–12 on the left side of the chamber. The factor “block” represents the eight-carrier blocks moving together, up to 48 blocks per experiment. One-Factor ANOVA using the factor “side” resulted in a significant (*p* ≤ 0.001) side effect. Plants grown on the right side of the chamber had a significantly increased dry weight (26.48 ± 8.17 mg) compared to those from the left side (21.85 ± 8.26 mg; Figure 10A). ANOVA checking for “lane” and “blocks” effects indicate significant differences between lanes 5, 7, and 12, respectively between the blocks 17, 24, and 43. Similar inhomogeneities were observed when analyzing leaf area (Figure 11). There are significant differences in leaf area between sides, lanes and several blocks. Plants grown on the right side of the chamber had an increased leaf area (1.332.417 ± 346.925 px^2^) compared to those from the left side (1.129.895 ± 355.858 px^2^). Adjusted means for the measured dry weight and calculated leaf area at 28 DAS were estimated using Linear Mixed Model setting the side, lane or block effect as fixed model terms and the interaction term side.lane.block as random. The chosen model successfully accounted for the environmental inhomogeneity inside the chamber. A positive linear correlation (*r* = 0.888; *p* ≤ 0.001) between dry weight and projected leaf area was observed. The good correlation confirms that the parameter leaf area from the image analysis can be used to predict the biomass of a plant.

### From model to crop: adaptation of *Arabidopsis* protocols for investigation of maize in a high throughput plant phenotyping system

In the next step, experiences gathered from a model plant system were used to establish an optimized cultivation protocols for maize in a HT phenotyping system. These protocols on the one hand were specifically adapted for studying specific aspects such as water use efficiency and on the other hand aim at elicitation of growth trait expression similar to that in the field by optimizing growth conditions with respect to temperature, watering and light regimes in the climate controlled glasshouse chamber housing the HT phenotyping system.

#### Features of the automated transport and imaging system for large plants

**Figure 12:**
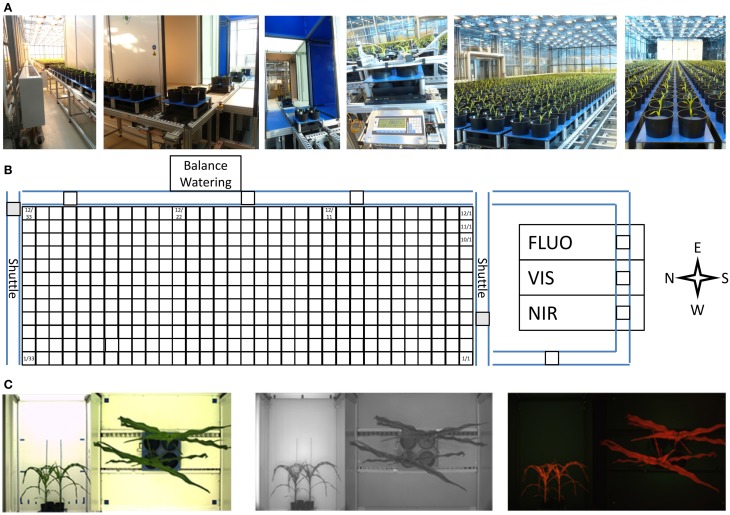
**(A) Automated transport, imaging, and weighing/watering system for HT phenotyping of large plants (LemnaTec Scanalyzer). (B)** The platform uses a conveyor system with two shuttles to automatically transport pot-grown plants in special carriers to three imaging stations for high resolution imaging and to a weighing and watering station. **(C)** Each carrier (carrying one or more plants) is imaged sequentially from the top and the side in multiple scanalyzer camera units for the visible light spectrum (left), near infrared (center) and fluorescence (right) imaging.

The HT automated non-invasive phenotyping (LemnaTec) system for midsized to large plant has a capacity for the cultivation of 396 individual large plants of up to 220 cm height or up to 1584 midsized plants. The system (ca. 100 m^2^) is equipped with 396 carriers (48 × 48 cm in size) used for automated transport of plants to three imaging chambers equipped with different camera sensors for VIS, FLUOR and NIR imaging. Weighing and watering is performed in an automated manner (Figure 12). The carriers are placed on a conveyor system and can carry one 20-liter pot (for a single large plant) or up to four 5-liter pots (for mid-sized plants) with a maximum load of 30 kg per carrier. The system consists of 12 lanes each storing 33 carriers which can be shuffled carrier-wise, in groups of carriers or lane-wise among the lanes and/or within the lanes. This HT phenotyping system is placed in a glasshouse chamber equipped with supplemental illumination. Two shading systems (one inside, one outside with 50% shading effect each, 3 threshold levels) control the plants exposure to sunlight (including thermal radiation). Climate control allows for setting temperatures in the range between 10 and 40°C. Air humidity levels can be raised to and kept at levels up to 95% (to avoid problems caused by condensation in parts of the electrical system, relative air humidity is usually set to less than 80%).

#### Standard cultivation and phenotyping regime adapted from the model system (*Arabidopsis*)

Using the experience gained with Arabidopsis in the automated phenotyping system for small plants, a standard setup and cultivation regime was implemented for single large and multiple midsized plants in the large plant system. In brief (for details of cultivation conditions see above: Materials and Methods), seeds were either directly germinated in the pots in the system (usually multiple seeds per pot, with plants thinned after emergence and seedling establishment) or seedlings were transplanted into the pots after pre-cultivation. Watering was arranged exclusively with supply from the top with either a single pump head driving water through a single tubing (for the single large pot configuration) or with four pump heads driving four tubes dispensing the same amount of water separately into the four pots per carrier. After an initial watering with fixed volumes usually done in multiple successive cycles to avoid drainage of excessive water not immediately soaked by the soil, the target weight of carrier was determined and used for control of the daily watering. To ensure equal soil water content, pots were filled by weighing with a fixed amount of soil from a sufficiently large stock of premixed soil with uniform moisture. After seedling emergence or transplanting the soil surface was covered with a single layer of the same perforated blue rubber mat as was used in the system for small plants. Depending on the size of the growing plants and their water consumption, either a single or two watering cylces were run per day. When switching to two daily waterings the target weight of the carriers were adjusted by adding an estimated weight of the plant(s) of the carriers. Imaging jobs were programmed similarly to those of the small plant system usually with a single top view and three or four side view images taken. A major difference to the small plant system to be considered in the experiment arrangement, however, was the much longer duration of a full system imaging (usually coupled with weighing/watering) cycle of 8–9 h and a watering only cycle of ca. 5 h. This is due to the different design of the conveyor system with each lane independently and sequentially operated and the much larger transport distances. After reaching an appropriate size, usually shortly after establishment, plants were kept upright by fixing them to blue sticks (painted or coated) or supported by a metal cage painted blue, which were stuck into the soil in a way not interfering with the watering tubes.

#### Specific modifications of experimental procedures to determine water use efficiency

**Figure 13:**
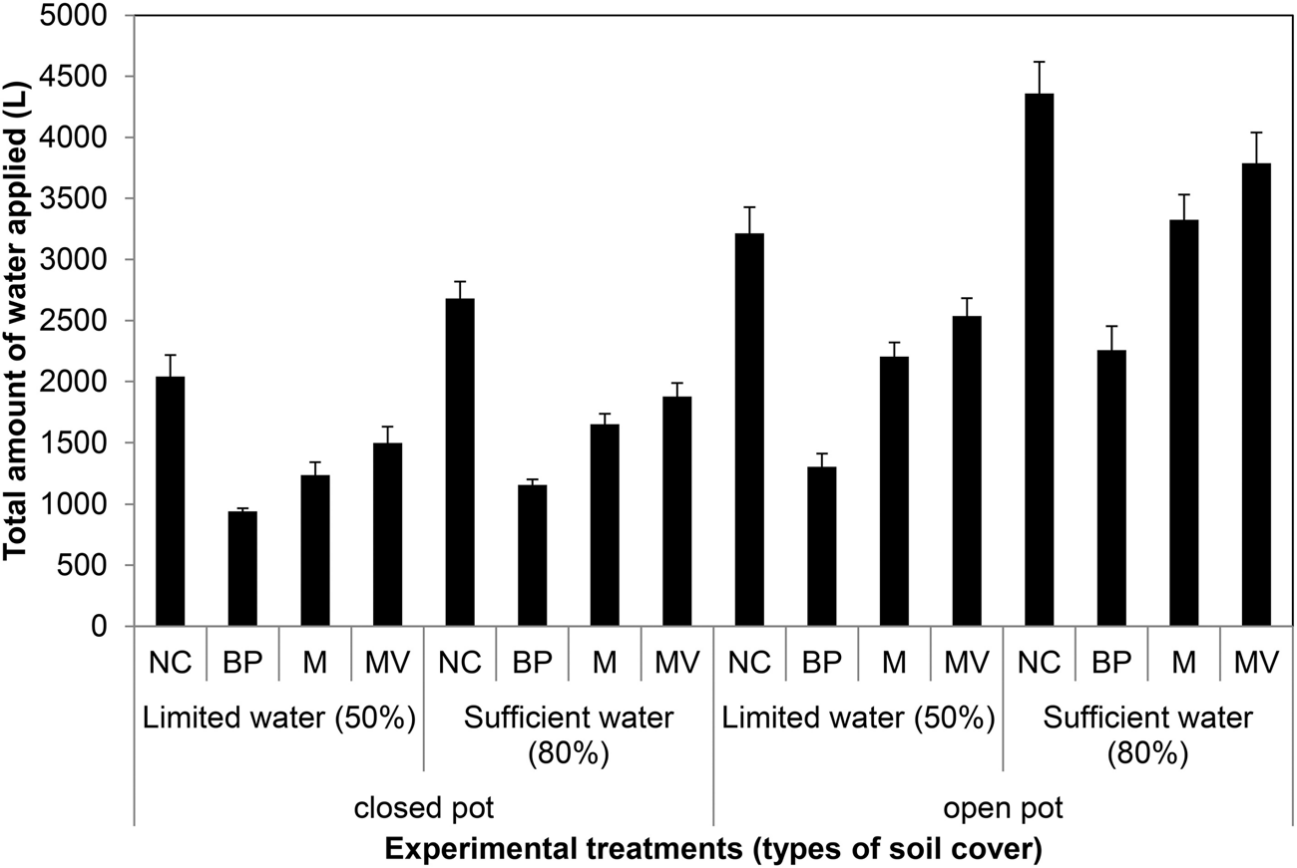
**Water evaporation from pots with closed and open bottom with the soil surface uncovered or covered with different materials (NC, uncovered; BP, blue pellets; M, blue rubber mat; and MV, black mulch mat)**. Water was applied at 2 levels: limited water supply corresponding to 50% field capacity and sufficient supply of water corresponding to 80% field capacity. Plants were grown under long-day conditions (06:00–22:00 h) at 25/20°C day/night, 65% relative humidity.

**Figure 14:**
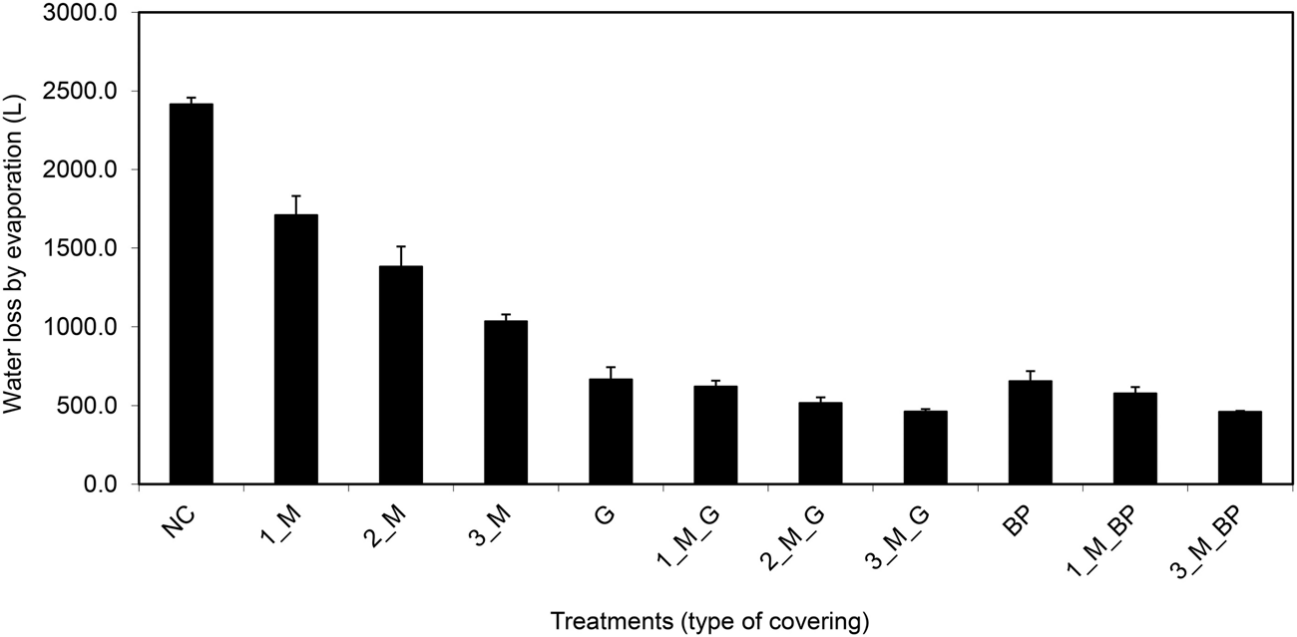
**Water evaporation from pots (bottom closed) with soil surface uncovered or covered with different materials (where, M, blue rubber mat; G, gravel; and BP, blue pellets; the pre-numerical value indicate the number of mats used in a given treatment) and sufficient supply of water (80% field capacity)**. Plants were grown under long-day conditions (06:00–22:00 h) at 25/20°C day/night, 65% relative humidity.

On the basis of the aforementioned standard setup, we aimed to further improve plant growth procedures in the HT phenotyping system for the assessment of water use efficiency (WUE) in the crop species maize. Two sets of experiments were carried out to test various adaptations using the maize standard cultivation procedures and to investigate the watering amounts and effects of water evaporation from the soil during the assessment of WUE. The volumetric soil moisture content was estimated at the start of each experiment (Figure S3) and was used to define the initial target weight of the pots to enable uniform consecutive automated watering regime for all pots. In the first experiment, two set of pot types, bottom open or closed by use of a plastic bag, with four treatments, open soil surface, soil surface covered with blue pellets, blue rubber mat or black mulch mat were used. In the second experiment closed pots, with 11 treatments, i.e., no soil covering, soil covered with blue pellets, blue mat, stone gravel (~10 mm diameter) and combinations of gravels or blue pellets with blue rubber mat were used. Blue rubber mats were applied at three levels, i.e., using single, double or triple mats. The first experiment was split into two groups: one set of plants was supplied with sufficient water (80% field capacity) and the other set was subjected to water limiting conditions (50% field capacity). The second set of experiments was performed keeping a target field capacity of 80% for all pots/plants. The volumetric soil moisture content was estimated at the start of each experiment (Figure S3) and was used to define the initial target weight of the pots to enable uniform consecutive automated watering regime for all pots. Our experiments showed that closed pots were more efficient in controlling water loss via evaporation than open pots, different covering material displaying variable potential to control water loss via evaporation with blue pellets being the best in both pot types among the tested materials (Figure 13). The ability to control water loss via evaporation, irrespective of covering treatment used, was found to be related to the amount of water applied (Figure 13). Using closed pots, a combination of two or more materials offered superior results (Figure 14), though we found a decreasing plant growth with increase in number of mats per pot (Figure S4).

According to these results, the finally established procedure for WUE assessment involves (i) the use of plastic bags inside the pots into which the soil was filled to block drainage through the bottom of the pots; (ii) adding 450 g of stone gravel (corresponding to a layer of about one cm) and placing three layers of the perforated blue rubber mat on top to reduce and equalize evaporation from the soil surface as much as possible; (iii) calculation of the amount of water consumed by the plants by correcting the water use of plant-containing pots with the water losses measured for plant-free pots filled with the same amount and type of soil and equipped with the same soil covers and kept at the same soil moisture level (field capacity level).

#### Environmental inhomogeneity and corresponding improvement of experiment design

To determine the spatial variation of environmental conditions within the plant growth area of the HT phenotyping system for large plants, data loggers were used to track temperature and relative humidity at four and two positions, respectively, in the system (four corners south-east, south-west, north-east, north-west) over a time period of 2 days. The results revealed a strong spatial variation, especially for temperature, across the west-east direction and a small variation along the south-north direction, especially on the boarders of the growth area (Figure S5). A panel of 44 maize inbred lines was grown in the HT system under standard conditions for two seasons. The genotypes were randomly assigned to the carrying units with 32 replications, assigned to 8 blocks of 4 plants each. Analysis of variance indicated no significant difference in biomass production with regard to the replicates (blocks = 4 plants), season, position in the lane (south-north orientation) and genotype^*^season interaction (Table 1), indicating high reproducibility of data even across seasons. However, a significant difference in biomass production was observed with regard to carrier position in the system and the genotype^*^carrier position interaction (Table 1). To test what causes the positional interaction effect the biomass of all replicates of the control genotype, that was randomly distributed over 28 positions (equal in both seasons) within the phytochamber (Figure S6), was analyzed. We found a gradient from east to west (biomass production at 42 DAS ranging from 218.3–273.2 g/plant; range = 54.94 g/plant, *SD* = 16.76, variance = 280.7) which corresponds to variation in environmental parameters (Figure S4).

**Table 1:** **Reproducibility of maize plant growth in the HT phenotyping system**.

**Source**	**DF**	**MS**	***F*-Value**	**Pr > F**
Genotype	43	25132.5	80.24	<0.0001
Rep (Blocks)	7	509.2	1.63	0.1298
Season	1	4.4	0.01	0.9062
Position	181	389.6	1.24	0.0653
Genotype^*^Season	39	109.6	0.35	0.8949
Genotype^*^Position	141	472.1	1.51	0.0037

*Analysis of variance (ANOVA) for 44 maize inbred lines cultivated over two seasons in the HT system indicate position but no season effects on plant biomass*.

As mentioned for the Arabidopsis experiment, two strategies are conceivable for correction of environmental inhomogeneities within the plant cultivation area: (1) use of an optimal experimental design suitable to estimate (and thus correct for) environmental influence (sufficient replication, and optimal positioning of replicates, suitable estimation models) and (2) shuffling strategies which ensure equal exposure of all plants of an experiment to divergent environmental conditions. The HT phenotyping system for large plants provides the possibility, by using shuttles accessing every lane individually, to implement shuffling strategies that optimize the carrier arrangement (as additional rotation cycles) in order to reduce effects of the spatial variation of the climatic conditions. A lane was defined as the primary shuffling unit composed of a set of 33 experimental plots (= carriers). In addition each primary shuffling unit (entire lane) was further portioned into 3 blocks of adjacent 11 experimental units, and each block within a lane defined as a secondary shuffling unit. An experiment was carried out to investigate the effect of systematic shuffling of experimental units within the HT glasshouse chamber. Plants of the control genotype were distributed over different positions in each of the 12 lanes (Figure S7). The primary shuffling was done by moving every day all carriers of a lane to the next lane in west-east direction. The secondary shuffling unit was done every other day in south-north direction by moving the blocks of 11 carriers one block forward (and returning the carriers of the northern block to the south block position). This shuffling approach eliminated the spatial variation of biomass production as demonstrated by the values of the control genotype, which did not show any significant differences among the 12 experimental units = lanes (biomass production at 42 DAS ranging from 377.9 to 391.5 g/plant; range = 13.9 g/plant, *SD* = 4.629, variance = 21.43).

#### Modification of maize cultivation conditions in a HT glasshouse to improve the match to field cultivation

A panel of 25 maize inbred lines (also grown under field conditions at the University of Hohenheim) was grown for three successive cultivations with eight replicate individuals per line in a climate-controlled glasshouse chamber under optimized growth conditions, closely resembling field cultivation with regard to temperature conditions during spring time in Gatersleben. The experiments were arranged in four blocks each block representing different time of harvesting (at 21, 28, 33, and 42 days after sowing). Genotypes were randomly assigned to these blocks. A significant correlation (*r* = 0.707; *p* > 0.01) between early fresh biomass under optimized growth conditions and under field conditions was observed (Table 2). The correlation was higher than that observed when plants were grown under conventional (normal) greenhouse conditions, where plants are exposed to a constant temperature of 25/20°C day/night during the entire growth period. The improved growth regime was implemented in the automated HT platform with small modification (15/10°C day/night for 4 weeks, then 20/13°C day/night for 1 week and finally 25/18°C day/night temperature for further 2 weeks; supplemental illumination using SonT Agro lamps). A set 63 lines (also grown and evaluated in field conditions at the University of Hohenheim) was cultivated for two seasons with eight replicate individuals of each line in the HT phenotyping system for large plants. The lines were characterized for biomass production from early to medium development stages, with various shoot traits and estimates of fresh shoot biomass extracted from the digital images taken daily from 14 days after sowing until final harvesting. A significant correlation (*r* = 0.747; *p* > 0.01) was observed between early fresh biomass formed both under the field and the optimized HT conditions (Table 3).

**Table 2:** **Correlation between fresh biomass of 25 maize inbred lines grown in various growth conditions (standard and optimized glasshouse conditions and field)**.

	**1**	**2**	**3**	**4**
Early biomass GH_Optimal (1)				
Mid biomass GH_Optimal (2)	0.658^**^			
Early biomass_Field (3)	0.707^**^	0.650^**^		
Final biomass_Field (4)	0.628^**^	0.604^**^	0.820^**^	
Early biomass_GH_Normal (5)	0.513^*^	0.731^**^	0.535^**^	0.415

** *Correlation is significant at the 0.01 level*.

* *Correlation is significant at the 0.05 level*.

*Where, “GH” denote glasshouse, “GH-Optimal” denote growth conditions established in this study, “Field” denote fresh biomass obtained under field conditions (data from, UHOH), and “GH-Normal” denote growth under standard greenhouse conditions (25/20°C day/night temperature throughout the growth period). Early biomass was measured after 3 and 4 weeks after sowing in GH and field, respectively; Mid-biomass was measured 6 weeks after sowing in the GH; Final biomass was measured in the field at plant maturity*.

**Table 3:** **Correlation between fresh biomass and estimated volume of 63 maize inbred lines grown in various growth conditions (optimized conditions in the HT phenotyping system and field conditions)**.

	**Early biomass GH-LT**	**Mid biomass GH-LT**	**Field_Early biomass**	**Field_Final biomass**	**Early estimated volume**
Mid biomass GH-LT	0.847^**^				
Field_Early biomass	0.747^**^	0.734^**^			
Field_Final biomass	0.689^**^	0.794^**^	0.733^**^		
Early estimated volume	0.709^**^	0.682^**^	0.638^**^	0.621^**^	
Mid estimated volume	0.690^**^	0.887^**^	0.572^**^	0.680^**^	0.622^**^

** *Correlation is significant at the 0.01 level (2-tailed). GH-LT = glass house with HT phenotyping system. Early biomass was measured after 3 and 4 weeks after sowing in GH-LT and field, respectively; Mid-biomass was measured 6 weeks after sowing in the GH-LT; Final biomass was measured in the field at plant maturity; Early- and mid-estimated volumes were extracted from inages acquired digitally at 3 and 6 weeks after sowing, respectively*.

## Discussion

Quantification of plant phenotypes using imaging based procedures in a HT, automated manner for plants grown under controlled conditions is indispensable for the generation of large high quality datasets describing characteristics of the plants habitus and the dynamics of their changes. The high degree of throughput and automation in plant care and measurement procedures, in comparison to usual small scale experiments with manual measurements, requires specific adjustments for all steps of the HT phenotyping workflow. It is essential to avoid manual steps as much as possible, in order to realistically profit from the reduced time demand to be achieved through process automation. In addition, it is crucial to establish conditions for all plants of an experiment, that enable reliable comparisons, even though different plant genotypes might have different properties, such as growth rates which might cause differences in water and/or nutrient consumption. All plants analyzed in the course of an experiment should experience identical environmental (aerial and soil) conditions, which represents a major challenge since large numbers of monitored individuals require large cultivation areas, which in turn are prone to spatial inhomogeneity of environmental conditions. Although in a reduced manner, seasonal, diurnal, and shorter term fluctuations observed under natural/field conditions occur in greenhouses. Even in climate-controlled phytochambers, that are supposed to offer the best possibility to dissect plant traits with good replicability and reproducibility, spatial and temporal variation of environmental conditions may occur (Poorter et al., 2012; Tisné et al., 2013). The low consistency of phenological observations of Arabidopsis plants grown in 10 different labs under controlled conditions, using a standardized protocol was suggested to be due to microenvironmental variation (Massonnet et al., 2010).

In the present study, we describe methods which aim at correcting for the effect on plant growth of existing spatial variation of environmental conditions in two different HT plant phenotyping systems with consideration of their special features and location. Two options are conceivable: (1) If a limited number of genotypes allows sufficient replication, an appropriate experiment design can be used to estimate and account for spatial effects, a procedure very commonly applied in field experiments (Petersen, 1994; Clewer and Scarisbrick, 2001). (2) If a very large number of genotypes needs to be screened and the degree of replication is low, carriers/plants can be moved (frequently) through all positions so as to expose all plants equally to all environmental conditions. This procedure is very demanding in terms of error-free operation of the thousands of movements. The automated phenotyping system for small plants, which was used here for Arabidopsis growth and phenotyping is placed inside of a phytochamber. Variation in environmental conditions was about 1% of the mean temp/humidity/light intensity. In the present study microenvironmental variation was found to cause differences in leaf area and dry weight of the plants grown in the different sides of the system/phytochamber. This HT phenotyping system does not allow to change the order of the carriers, as it is setup as a continuous conveyor system, but carriers can be moved through all positions of the plant cultivation area multiple times per day. As an alternative, appropriate experimental design and statistical methods could be shown to account for the existing variation. The data reveal that the selection of an optimized experimental design, including an adequate number of replicates distributed in an appropriate pattern across the chamber enables the application of statistical methods accounting for environmental inhomogeneities. Therefore, for phenotyping large populations without constant rotation, an alpha-lattice design incorporating the 8-carrier blocks is used, whereas the incomplete 8-carrier-blocks are combined to form a complete replicate or “super-block.” Each experiment consists of at least three such replicates. The exact number depends, e.g., on the number of lines to be analyzed and on the type of pot/tray to be used. Positional (and other) effects (caused by environmental inhomogeneities) are accounted for by applying mixed-effect linear models to calculate adjusted entry means. These approaches have been proven to serve as efficient tools during the analysis of high-dimensional phenotypic data (Granier and Vile, 2014). Accordingly, their use has been reported for phenomics datasets acquired with other HT plant phenotyping systems (Skirycz et al., 2011). Brien et al. (2013) compared different carrier relocation strategies with the application of appropriate experimental design and statistics and concluded that the latter strategy is more efficient to account for environmental variation. In contrast to this observation, in the present study, we found that shuffling procedures in the automated phenotyping system for large plants (in the glasshouse, used for maize analyses) were suitable to eliminate effects of spatial environmental inhomogeneity. Variation in maize plant growth was very considerably reduced by daily relocation of the carriers (plants) lane-by-lane from east to west and block-to-block from south to north within lanes inside the glasshouse (returning to its original position at sawing date every 12th day). Supporting this strategy, Tisné et al. (2013) even used permanent rotation of plants in order to reduce environmental variation within the plant growth area. Similarly, Wallihan and Garber (1971) found an increased precision in rice growth quantification on a rotating system compared to stationary workbenches. Although plants are frequently stimulated mechnically in nature (e.g., by wind, rain, animals, etc.), movement as caused by carrier transport might be considered as additional environmental factor possibly resulting in phenotypic differences with regard to plant habitus and/or metabolism. The latter is often referred to as the penultimate phenotypic expression of an organism a level below its morphological phenotype. Metabolic alterations due to genotypic or environmental variation may directly be linked to morphological alterations but over time, differences in metabolic performance can be expected to affect growth and development. In the present study, we were able to show that multiple rotation cycles per day neither lead to significant changes in plant growth and biomass accumulation nor in metabolite composition. Plants are known to respond to mechano-stimulation showing effects on plant morphology (thigmomorphogenesis), chlorophyll content, stress resistance and flowering time as mediated by a complex signaling network including hormonal and transcriptional signals (Chehab et al., 2009). The fact that the plant movements in the HT phenotyping system (start/stop impulses, vibrations during transport) did not lead to morphological or physiological alterations in Arabidopsis indicates that the transportation induces only subtle mechano-stimulation below the critical threshold. In comparison to touch stimulation (Braam, 2005) this stimulus might be too weak to cause effects or frequently/continuously occuring stimulation might lead to a rapid desensitization of Arabidopsis plants (Wallihan and Garber, 1971). The movement effect might be dependent on the species as well as on its developmental stage and habitus. Thus, similar checks are advisable for experiments involving other plant species or addressing other developmental phases.

The microclimatic variation in phytochambers/glasshouses represents an important factor also for the soil/plant water status and transpiration processes (Granier et al., 2006; Tisné et al., 2013). Given that effects caused by environmental variation can be reduced/corrected for as much as possible by either of the above proposed strategies (plant rotation and appropriate statistical analyses), further factors have to be considered in HT plant phenotyping systems which are related to automation of plant care: On the one hand, there are too many plants for manual care, and on the other hand, at least in the system for large plants a very large fraction of the plants is inaccessible to people. Among the most important issues are watering procedures, which have to be adapted with regard to the way of dispensing, the different culture vessels (pot sizes, tray arrangements), the frequency of watering (avoidance of too much fluctuation in water availability) and fertilizer application, as well as the specific demands of different plant species during their development. As watering in the HT phenotpying systems is performed sequentially, one has to carefully consider the timing, with potential effects caused by treatment at different time points of the day. In the LemnaTec Scanalyzer systems, controlled watering has been implemented here using predefined target weights: Each carrier is weighed and watered with the amount of water equalizing the difference to a given target weight. Thus, the loss of water monitored by the loss of weight is compensated at regular intervals. Otherwise, soil water content will vary und thus will influence water availability, in particular under non-saturating conditions. This poses quite some logistic demands, as pot filling needs to be done using either a very exact volume or a precise weight of soil, which needs to be highly homogenous with respect to composition and moisture from the first to the last pot. Furthermore, the gain of weight due to the growth (fresh weight accumulation) of the plants has either to be small relative to the amount of water available to/consumed by a plant and thus be neglible. Or target weights have to be adjusted at appropriate times using proper estimates of the weight added by the growing plants. Considering these prerequisites standard watering protocols were established and implemented for small (e.g., Arabidopsis) and large plans (e.g., maize) in the two HT plant phenotyping systems which include the use of blue cover material resulting in reduced water evaporation from the soil. Another solution was worked out, e.g., for the GlyPh phenotyping system in which water is dispensed into funnels and lead into the soil (Pereyra-Irujo et al., 2012).

Plant growth protocols applicable to HT phenotyping systems also have to be optimized with regard to image analysis procedures in order to support precise quantification of plant features in a time-saving and reliable manner. Specific adjustments are necessary in order to avoid errors in automated measurements and to circumvent time-consuming manual or visual re-checking. A critical step of HT (batch) image analysis is the separation of plant structures from the background (segmentation). In addition to its function in reduction of water transpiration, the use of blue soil cover materials or specific growth substrates which are compatible with the measurement procedure, substantially facilitates the segmentation process. Minimization of background noise, caused for example by moss growth on the wet soil surface, enables a clear separation of plant structures from the soil background and ensures precision in trait quantification. Pereyra-Irujo et al. (2012) similarly describe the application of white funnels for background signal reduction. Furthermore, the data show that using the perforated blue rubber mats covering the soil surface does not cause significant changes in metabolite contents. Growth of Arabidopsis plants was slightly enhanced upon use of the blue mats, which might be caused by the reduction in water evaporation from the soil surface and thus a more uniform water supply to the plants. The mats may also affect slightly the temperature of the rosette leaves of the plants by isolating them from the cooler wet soil surface. In summary, we conclude that covering the soil with the blue mats has no negative or unintended effects on the plants metabolism and growth and development und thus on the plants phenome.

In the present study the Integrated Analysis Platform (Klukas et al., 2014) was used to calculate projected leaf area (Arabidopsis) and estimated volume (maize) from the digital images acquired daily during the plant growth period. These are only two of several hundred features that are extracted from the images through a run of this software from each set of pictures (usually each set of images taken of a certain carrier at one particular time point consists of a top view images and several side view images). Details of this (including aspects of data mining) will be published elsewhere (Chen et al., 2014). For interpretation of this huge amounts of data collected in a single plant phenotyping experiment, for their efficient reuse in future investigations, and for the documentation of the applied experimental procedures, the image analysis results (phenomics data) have to be linked to contextual information (i.e., metadata; sample characteristics, technology and measurement types; instrument parameters and sample-to-data relationships). Here, we used the Investigation/Study/Assay (ISA) infrastructure, which represents a general-purpose format designed to regularize the local, integrated management of experimental results and metadata by aiming at the unambigiuous documentation and traceability of the phenotyping workflow, supporting ontologies and other community-defined reporting standards and preparing studies for submission to public repositories (Rocca-Serra et al., 2010).

As mentioned above, the experience gained during the establishment of the Arabidopsis HT-compatible cultivation protocol was further used to setup a protocol for standard cultivation and analysis of maize plants in a greenhouse HT phenotyping system. The basic procedures thus established were further developed for two special purposes:

1. [(1)] A specific watering procedure has been developed to asses directly water use efficiency (WUE) in maize, defined as the units of plant biomass harvested per cumulative volume of water consumed. The specific adaptations included the adherence to a certain field capacity level during the entire cultivation period and taking measures to reduce as much as possible loss of water from the pots and to measure as precisely as possibe the inevitable evaporation. Precise control of the watering procedures (volumes per day) and the resulting flexibility for establishing multiple watering scenarios (including transpiration control by covers) enables to assess WUE under close to natural conditions (Pereyra-Irujo et al., 2012). This approach is as precise as ^13^C isotope discrimination analyses, which are often used as indirect measures of WUE (Martin et al., 1999; Impa et al., 2005). The results (WUE estimates) obtained for vegetative growth may, however, differ from those related to fruit or grain yield and may vary considerably under different environmental conditions ranging from pot cultivation in the glasshouse to growth in the field.
2. [(2)] An increased match of the performance relationships (rankings) among divergent genotypes to that observed in the field. To achieve this, we modified the maize plant growth protocol to mimick in the greenhouse and the HT plant phenotyping system therein more the naturally occuring seasonal temperature variations in the field. The temperature regime was adjusted to the incremental increase in temperature in Gatersleben during spring time and maize biomass development under these conditions correlated well to that observed under field conditions.

Field conditions represent the plants natural environment with diurnally and seasonally varying environmental parameters such as light, temperature, water. From an economic perspective, only if effects observed under controlled conditions can be reproduced under field conditions (natural) it is of relevance to breeders and has the potential to be included into breeding programs establishing commercial plant lines (Zhu et al., 2011; Cobb et al., 2013; Araus and Cairns, 2014). Field trials are very labor- and time consuming, requiring multi-location experiments over several years in order to identify genetic determinants of plant growth/yield or any trait of interest. Therefore, it is important to establish growth protocols which are able to imitate the plant growth behavior in the field. The most sophisticated solution for multivariate environmental simulation mimicking different natural climate scenarios can be achieved by the use of special phytochambers with precise control and of as much as possible environmental parameters (http://www.csf.ac.at/facilities/plants/). Nevertheless, complex and non-systematic variation of multiple parameters (such as under field conditions) makes it difficult to draw conclusions on causal effects of individual parameters whereas variation of single or few desired parameters by purpose allow for drawing specific conclusions on environmental effects. Therefore, many analyses comparing natural and controlled conditions focus on one specific parameter such as light conditions. Several reports gave evidence for the potential of artificially fluctuating light to mimick naturally occuring photosynthetic acclimation processes in Arabidopsis and maize (Suorsa et al., 2012; Hirth et al., 2013). In this study, the plants cultivated in the glasshouse were exposed to temporally varying light intensities, although with much restricted intensity ranges and lower dynamics as compared to the field situation. Thus, modifications were focused here on the temperature regime and resulted in very substantial improvement, as shown by increased rank correlations between data of the glasshouse cultivation and of field trials. Further optimization of plant growth under controlled vs. field conditions is supposed to be achievable by considering additional environmental parameters which vary between field and pot/glasshouse cultivation, such as soil quality, rooting space, plant density and light quality. Optimizing the simulation of field conditions in controlled systems however at first requires comprehensive monitoring of these parameters in field trials.

As maize is one of the most important crop plants worldwide and improvement of maize biomass accumulation is of high relevance to breeders and agronomists, this study is of relevance for scientists interested in plant phenotyping, both from a technical as well as a biological perspective.

Quantitative and non-invasive HT plant phenotyping represents an integrative, multi-disciplinary approach of crucial relevance for the acquisition of large-scale phenomic datasets with high precision and reproducibility. Such data may be associated with genetic data in order to identify genetic causes of variation in trait expression, they may be integrated with molecular / biochemical profile data to uncover molecular-physiological processes underlying important plant properties such as growth performance or yield formation or they may be used directly for the selection of genotypes with superior properties in plant breeding. Beyond the development of suitable sensors and the identification of relevant readouts to assess important plant properties (or proxies thereof) a major challenge is the optimization of each of the multiple interdependent steps of the phenotyping process. This manuscript addresses issues concerning each phase of the phenotyping workflow: planning and preparation of a plant phenotyping experiment (e.g., experimental setup), issues during the process of plant cultivation and data acquisition (pointing to critical cultivation parameters which can affect plant growth and habitus as well as its physiological status as analyzed here by metabolite profiling) as well as post-experimental procedures such as image data analysis, statistical evaluation and the appropriate documentation of the plant phenotyping experiment in conjunction with its results. The presented optimization strategies for plant cultivation protocols should be regarded as indications and guidelines rather than ready-to-use protocols for other HT plant phenotyping facilities, because each installation and each cultivation infrastructure will have its own specific properties to be considered. Therefore, the key message of this manuscript is to increase the awareness of plant scientists with regard to issues that need to be considered during the establishment and use of HT plant phenotyping systems and that will need to be adapted specifically for each type of facility and/or related biological question.

## Author contributions

Astrid Junker: created standardized experiment documentation (IsaTab), wrote manuscript, Moses M. Muraya: performed maize experiments and carried out data analyses, wrote manuscript, Kathleen Weigelt-Fischer: performed Arabidopsis experiments and carried out data analyses, wrote manuscript, Fernando Arana-Ceballos: performed Arabidopsis experiments, Christian Klukas: performed image analyses, Albrecht E. Melchinger: provided maize seed stocks and field data, Rhonda C. Meyer: supervised Arabidopsis experiments and data analyses, Thomas Altmann: supervised the project, wrote manuscript.

### Conflict of interest statement

The authors declare that the research was conducted in the absence of any commercial or financial relationships that could be construed as a potential conflict of interest.

## Supplementary material

Figure S1 **Light emission spectra of the Whitelux Plus metal halide lamps (A) used for illumination of cultivated Arabidopsis plants, and of the using SonT Agro high pressure sodium lamps (B) and HPI-T quartz metal halide lamps (C) used for supplemental illumination during maize cultivations**.

Figure S2 **Environmental inhomogeneities within the small (Arabidopsis) high throughput phenotyping phytochamber**.

Figure S3 **Soil water moisture content: Gravimetric water content (θg) = (Weight of soil at potting—weight of oven-dried soil)/weight of oven dried soil); Field capacity = (weight of soil at maximum water holding capacity—weight of oven-dried soil)/weight of oven-dried soil)**. Sensor = soil moisture content at field capacity measure using moisture sensor. Soil constitution: 40% composite, 40% substrate 2, and 20% sand.

Figure S4 **Fresh biomass of maize lines in different pots/different covers**. Effects of increasing the number of blue rubber mats on fresh biomass production of a maize inbred line and hybrid: NC, uncovered; BP, blue pellets; G, gravel; and M, blue rubber mat; the pre-numerical value indicate the number of blue rubber mats used in a given treatment.

Figure S5 **Environmental variation in the greenhouse housing the HTP system for large plants. (A)** Temperature measurements were performed over 2 days at four positions inside the glasshouse by using Voltcraft WDL-TH und WDL-K" loggers (TH—temperature and humidity, K—temperature). K02 and TH3 were placed in carrier position 3 and 30 of lane 1 (west) and K04 and TH1 were placed in carrier position 3 and 30 of lane 12 (east) as marked in blue. **(B)** Temperature profile in the glasshouse. Black: Western lane (1), Gray: Eastern lane (12).

Figure S6 **Distribution of 28 replicates of the standard genotype in the glasshouse**. Carrier positions are marked in blue. The lanes were not shifted within the phytochamber.

Figure S7 **Distribution of 12 replicates of the standard genotype in the glasshouse**. Carrier positions are marked in blue. The lanes were shifted within the phytochamber. Each day each lane shifts one more position from west to east, taking 12 days to return to its original position. In addition, within the lane a block of 11 plots were shifted every other day from south to north, taking 6 days for the experimental plot to return to its original position within the lane.

File S1 **Metabolite Profiles of differentially cultivated plants (n = 17)**.

Table S1 **The geographic origins and pedigree data of the maize inbred lines**.

Table S2 **Bonferroni corrected *p*-values of a Two Factorial ANOVA analysis having location (LT/Stationary) and cover (Covered/Uncovered) as factors**.
