# Supplementary figures and images for: Optimizing experimental procedures for quantitative evaluation of crop plant performance in high throughput phenotyping systems
Source: Front Plant Sci. 2015 Jan 20;5:770. doi: 10.3389/fpls.2014.00770 (PMC4299434; doi:10.3389/fpls.2014.00770)

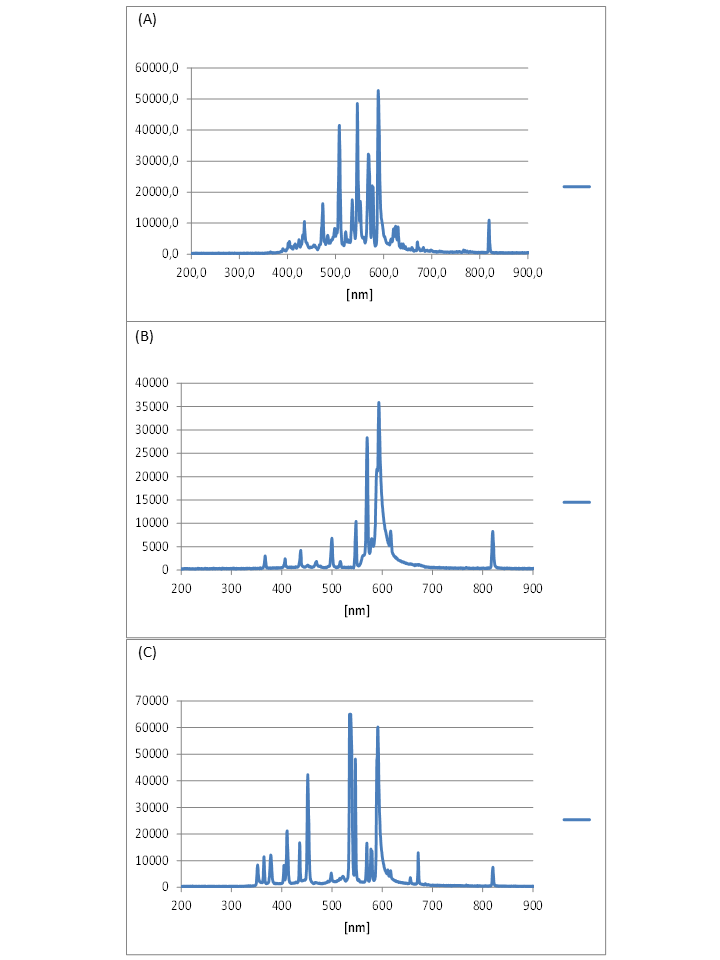

Supplement: Figure S1 — Light emission spectra of the Whitelux Plus metal halide lamps (A) used for illumination of cultivated Arabidopsis plants, and of the using SonT Agro high pressure sodium lamps (B) and HPI-T quartz metal halide lamps (C) used for supplemental illumination during maize cultivations. [file Image1.TIF]

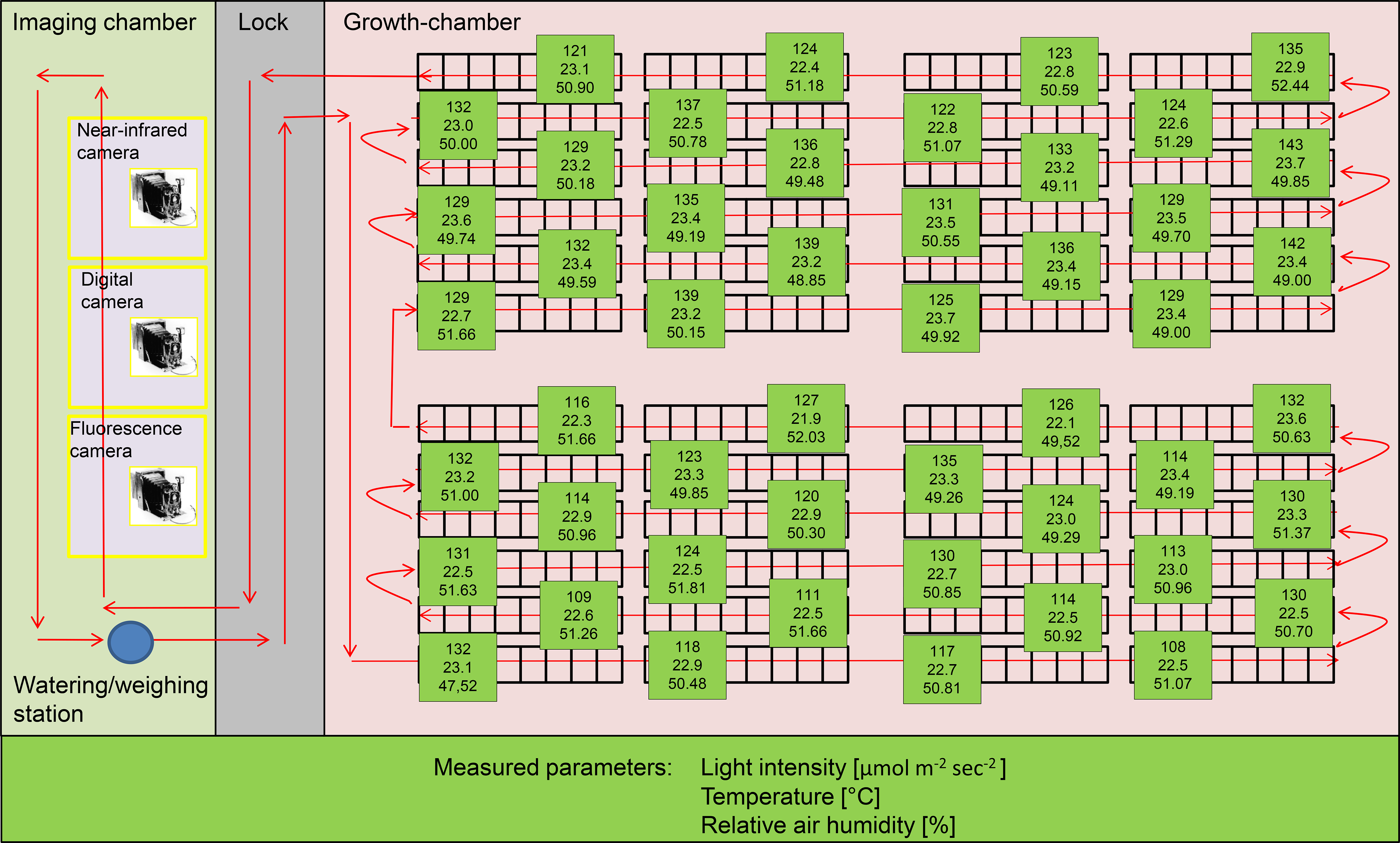

Supplement: Figure S2 — Environmental inhomogeneities within the small (Arabidopsis) high throughput phenotyping phytochamber. [file Image2.TIFF]

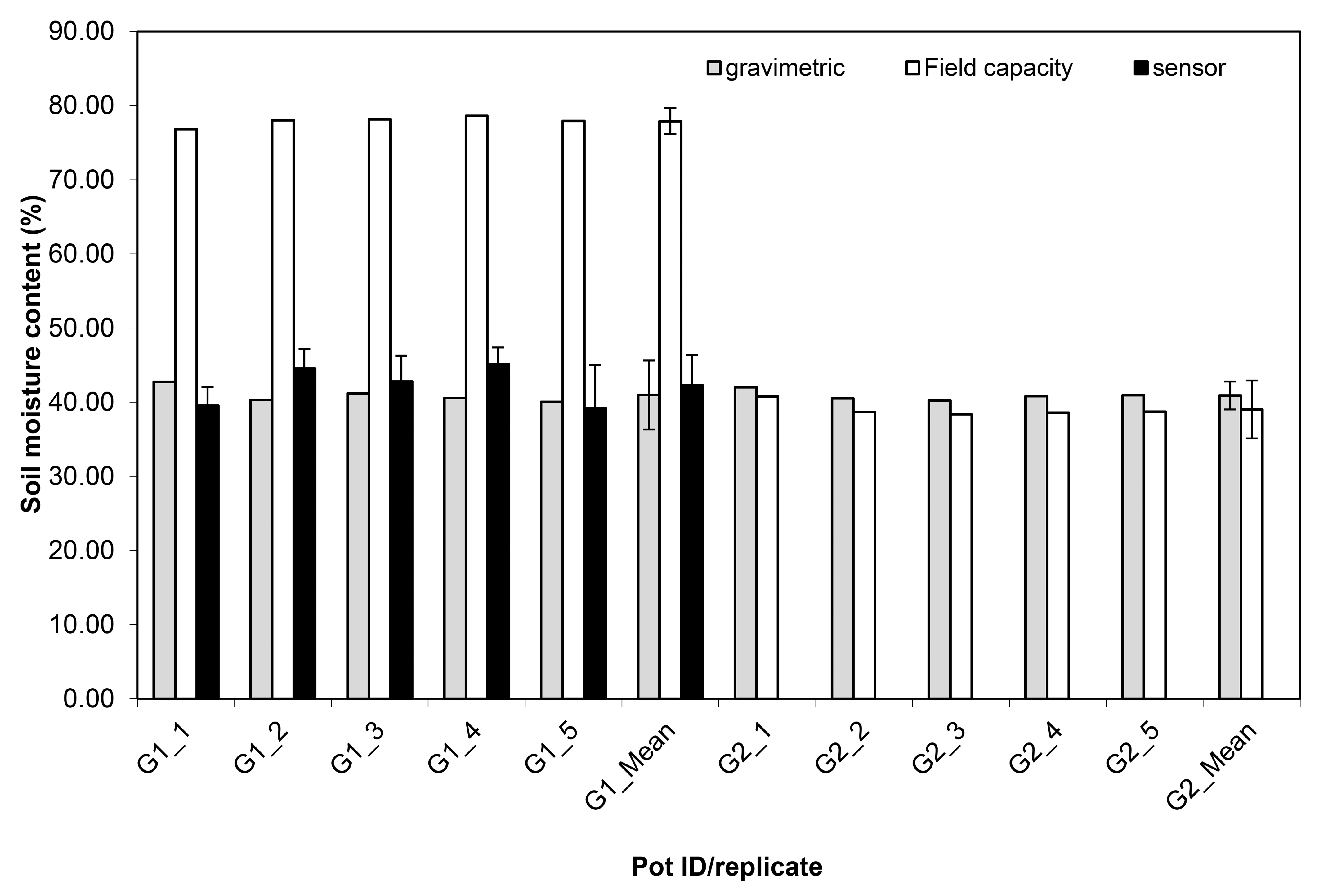

Supplement: Figure S3 — Soil water moisture content: Gravimetric water content (θg) = (Weight of soil at potting—weight of oven-dried soil)/weight of oven dried soil); Field capacity = (weight of soil at maximum water holding capacity—weight of oven-dried soil)/weight of oven-dried soil). Sensor = soil moisture content at field capacity measure using moisture sensor. Soil constitution: 40% composite, 40% substrate 2, and 20% sand. [file Image3.TIFF]

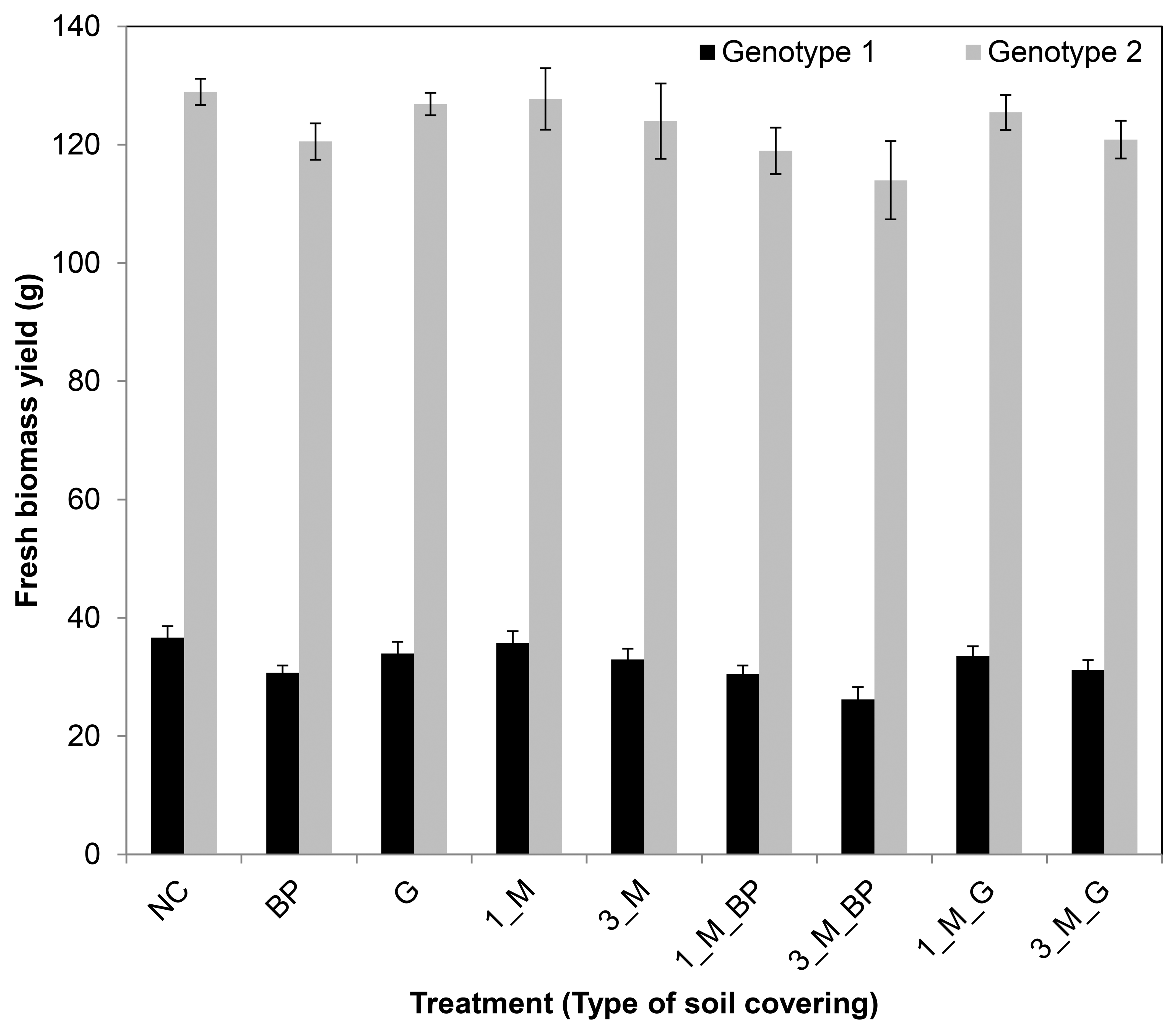

Supplement: Figure S4 — Fresh biomass of maize lines in different pots/different covers. Effects of increasing the number of blue rubber mats on fresh biomass production of a maize inbred line and hybrid: NC, uncovered; BP, blue pellets; G, gravel; and M, blue rubber mat; the pre-numerical value indicate the number of blue rubber mats used in a given treatment. [file Image4.TIFF]

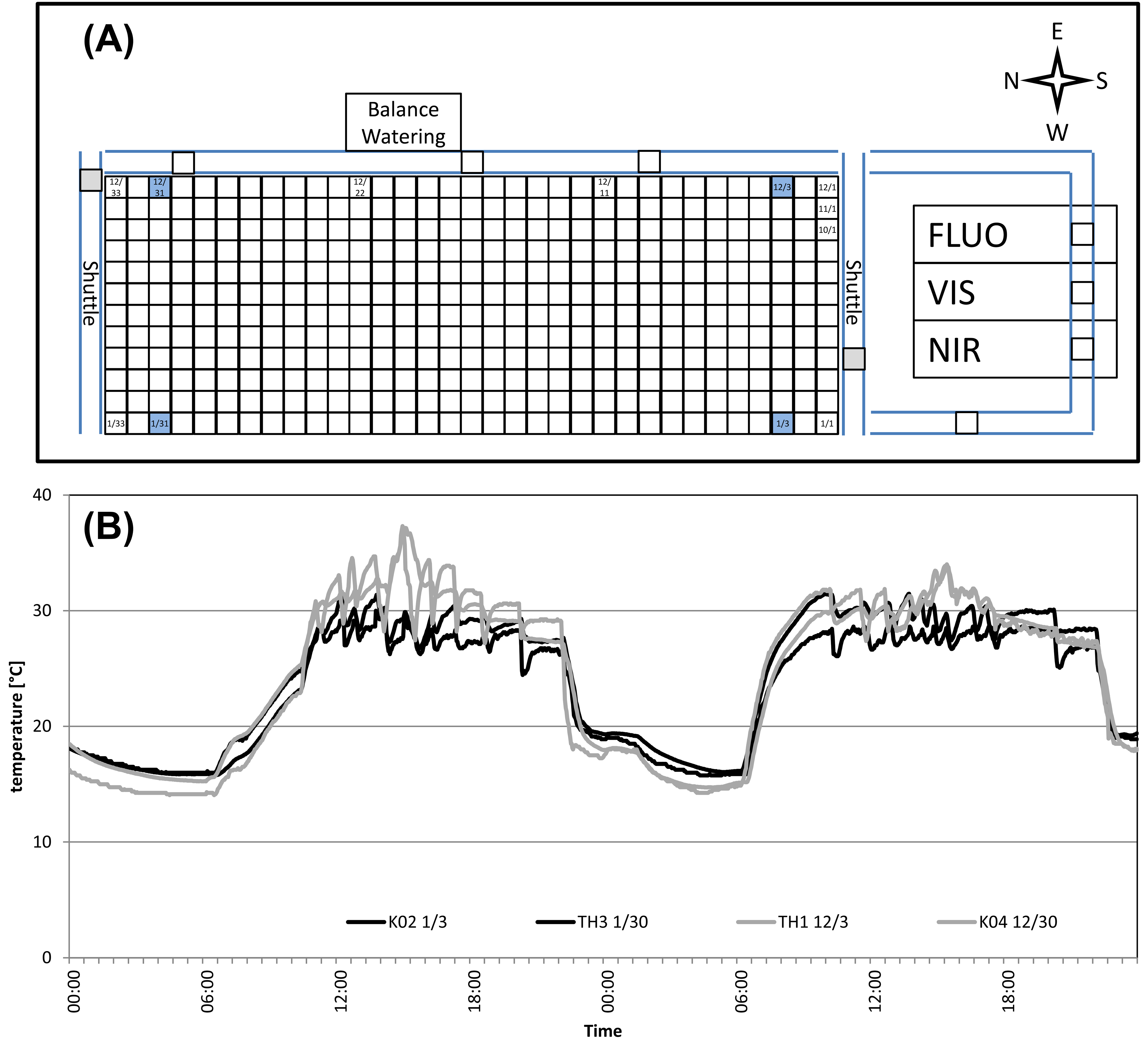

Supplement: Figure S5 — Environmental variation in the greenhouse housing the HTP system for large plants. (A) Temperature measurements were performed over 2 days at four positions inside the glasshouse by using Voltcraft WDL-TH und WDL-K" loggers (TH—temperature and humidity, K—temperature). K02 and TH3 were placed in carrier position 3 and 30 of lane 1 (west) and K04 and TH1 were placed in carrier position 3 and 30 of lane 12 (east) as marked in blue. (B) Temperature profile in the glasshouse. Black: Western lane (1), Gray: Eastern lane (12). [file Image5.TIFF]

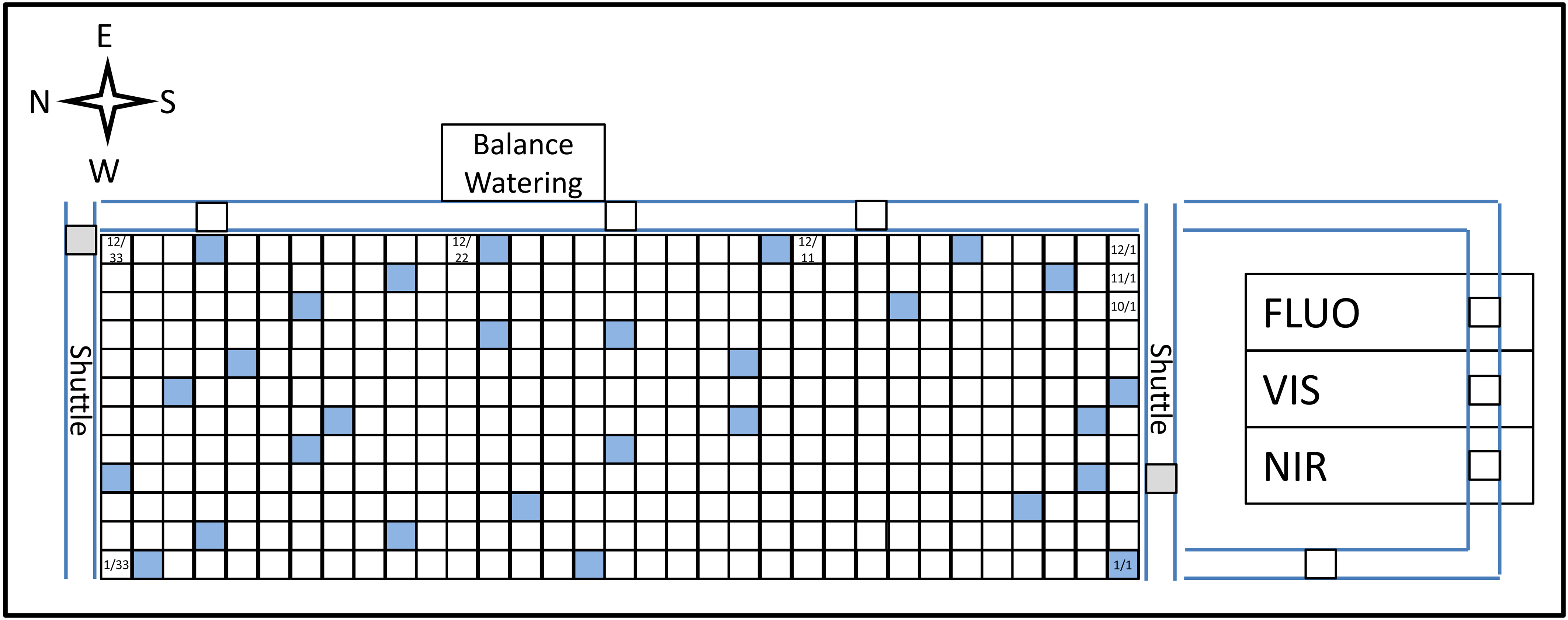

Supplement: Figure S6 — Distribution of 28 replicates of the standard genotype in the glasshouse. Carrier positions are marked in blue. The lanes were not shifted within the phytochamber. [file Image6.TIFF]

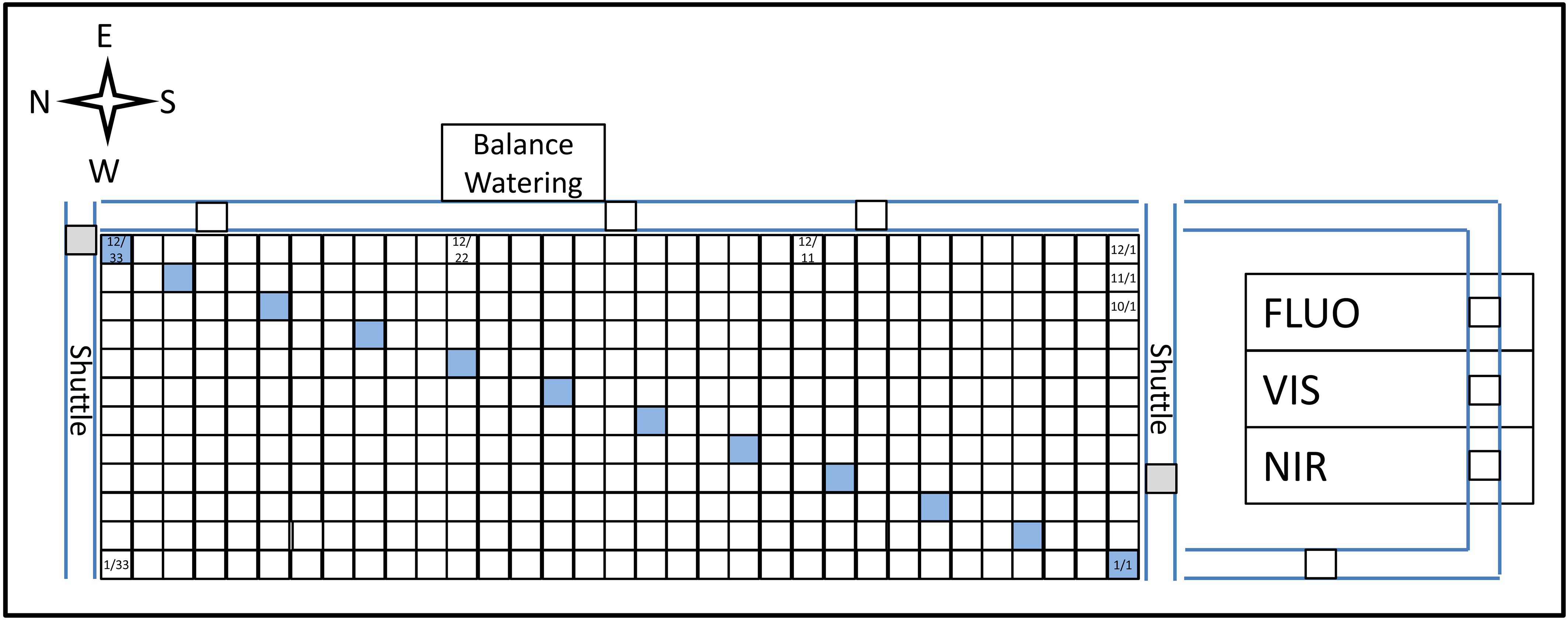

Supplement: Figure S7 — Distribution of 12 replicates of the standard genotype in the glasshouse. Carrier positions are marked in blue. The lanes were shifted within the phytochamber. Each day each lane shifts one more position from west to east, taking 12 days to return to its original position. In addition, within the lane a block of 11 plots were shifted every other day from south to north, taking 6 days for the experimental plot to return to its original position within the lane. [file Image7.TIFF]
