# Supplementary material for: Optimizing experimental procedures for quantitative evaluation of crop plant performance in high throughput phenotyping systems
Source: Front Plant Sci. 2015 Jan 20;5:770. doi: 10.3389/fpls.2014.00770 (PMC4299434; doi:10.3389/fpls.2014.00770)
Supplement: File S1 — Metabolite Profiles of differentially cultivated plants (n = 17). [file DataSheet1.PDF]

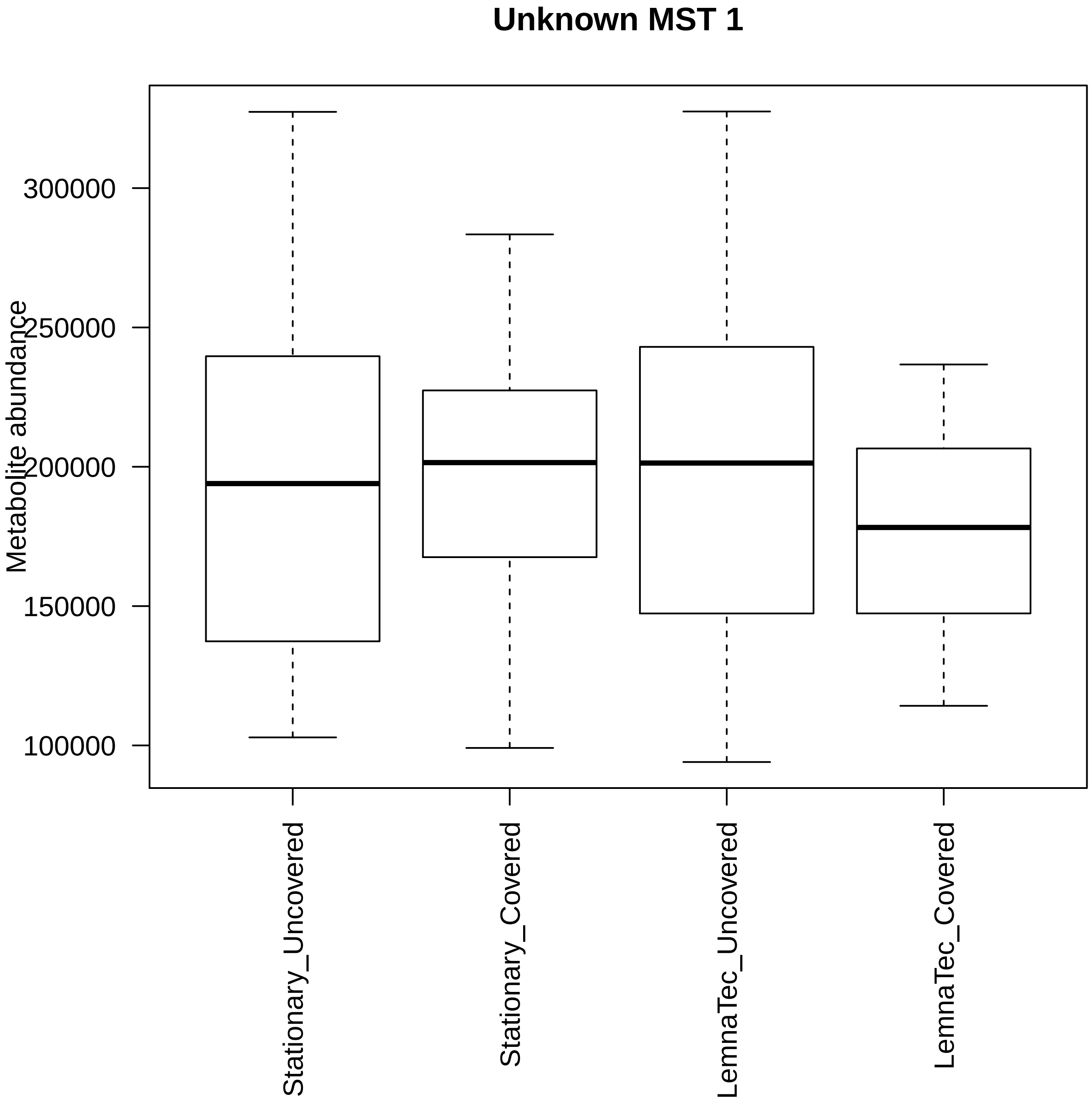

Unknown MST 1
Metabolite abundance
300000
250000
200000
150000
100000
Stationary_Uncovered
Stationary_Covered
LemnaTec_Uncovered
LemnaTec_Covered

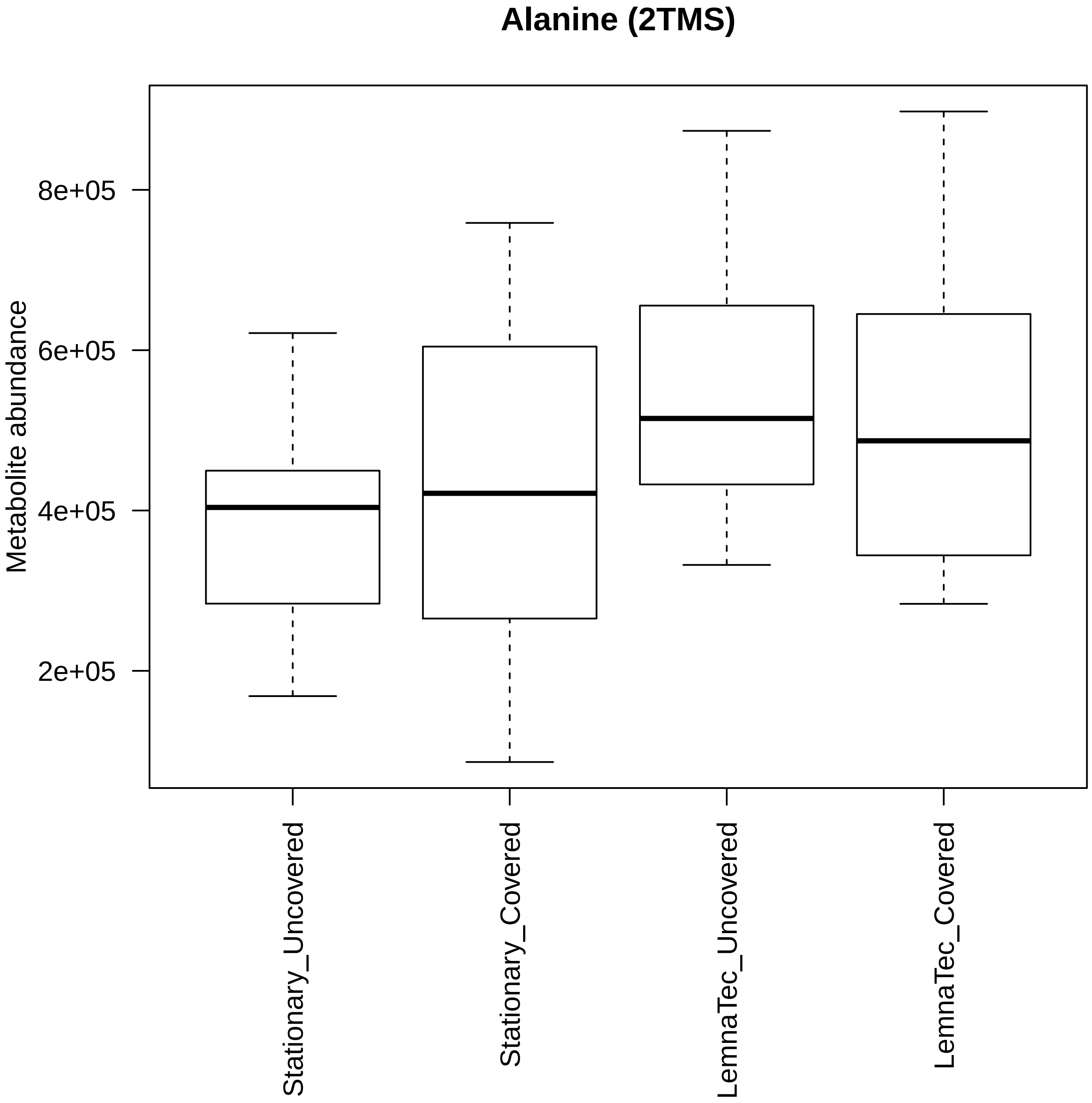

Alanine (2TMS)
Metabolite abundance
8e+05
6e+05
4e+05
2e+05
Stationary_Uncovered
Stationary_Covered
LemnaTec_Uncovered
LemnaTec_Covered

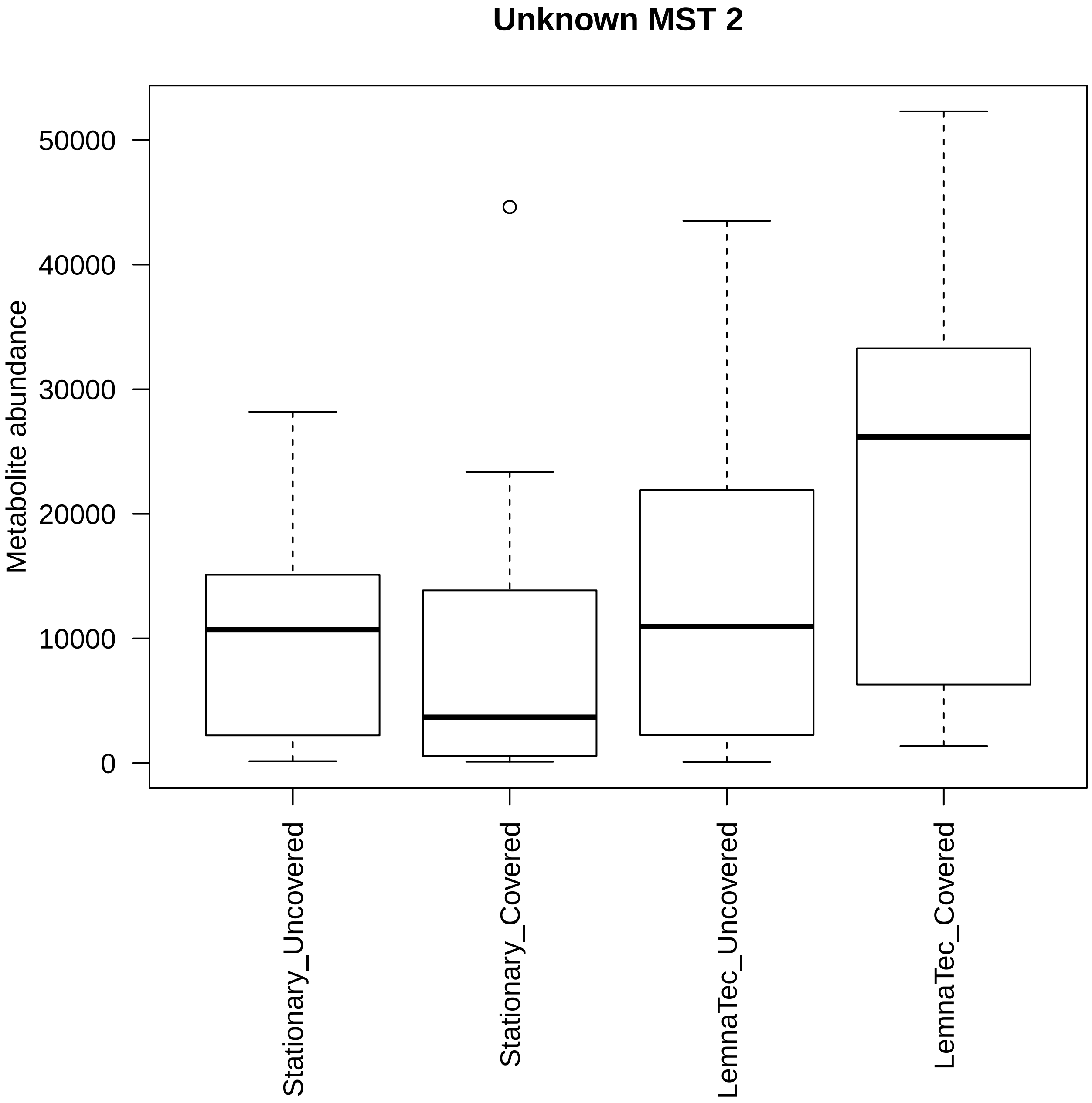

Unknown MST 2
Metabolite abundance
0
10000
20000
30000
40000
50000
Stationary_Uncovered
Stationary_Covered
LemnaTec_Uncovered
LemnaTec_Covered

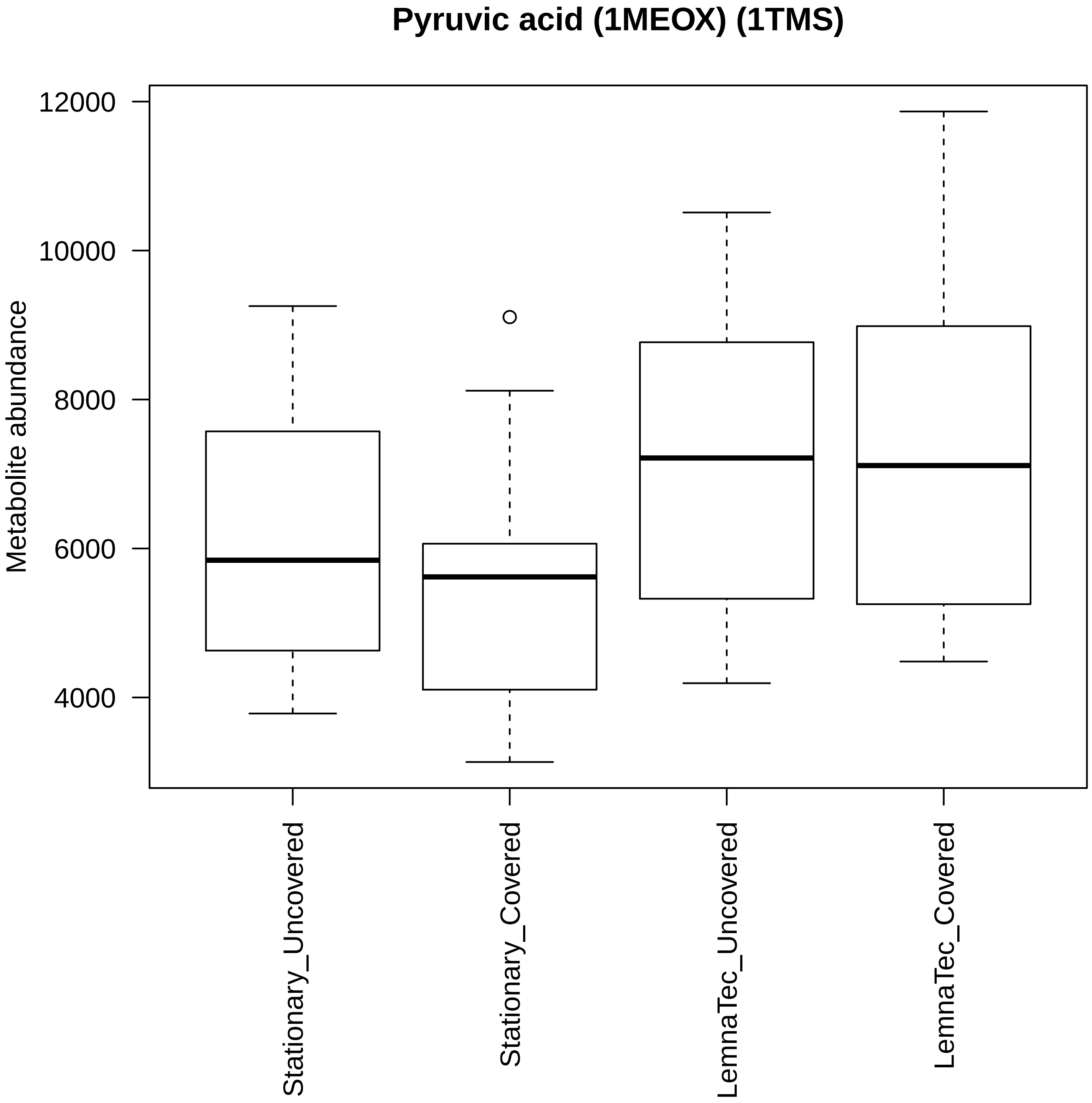

Pyruvic acid (1MEOX) (1TMS)
Metabolite abundance
12000
10000
8000
6000
4000
Stationary_Uncovered
Stationary_Covered
LemnaTec_Uncovered
LemnaTec_Covered

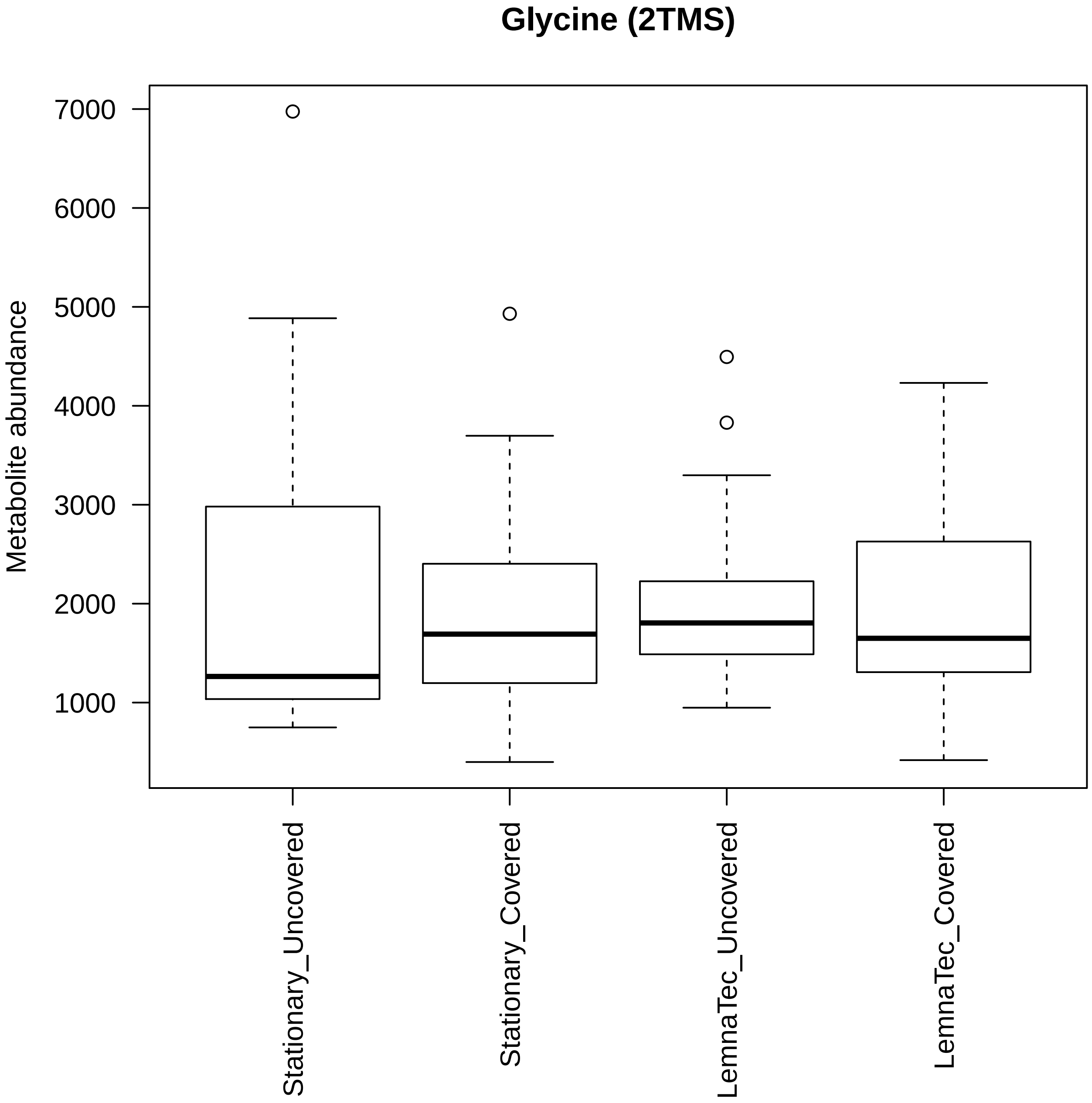
Glycine (2TMS)
Metabolite abundance
7000
6000
5000
4000
3000
2000
1000
Stationary_Uncovered
Stationary_Covered
LemnaTec_Uncovered
LemnaTec_Covered

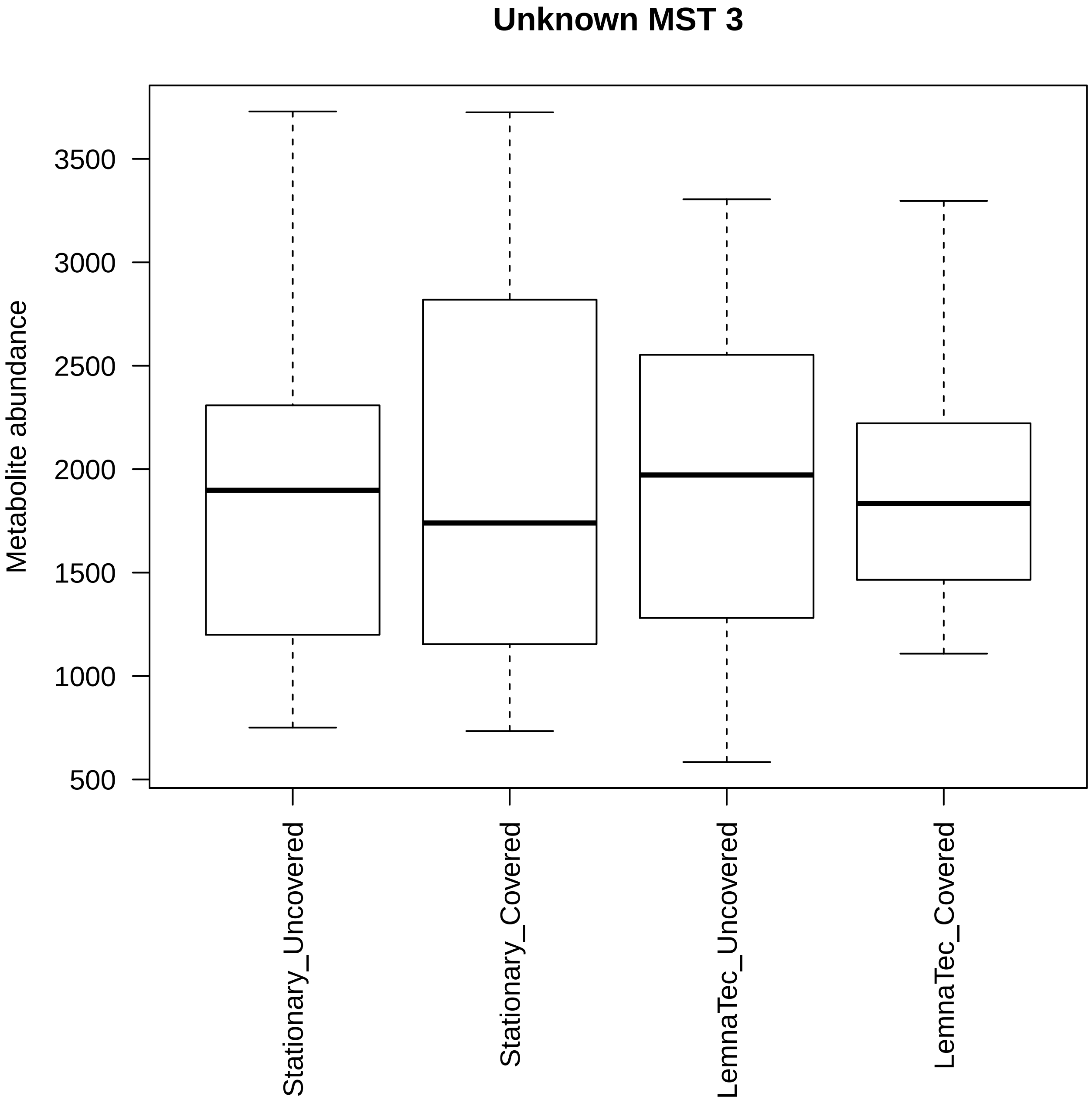

Unknown MST 3
Metabolite abundance
3500
3000
2500
2000
1500
1000
500
Stationary_Uncovered
Stationary_Covered
LemnaTec_Uncovered
LemnaTec_Covered

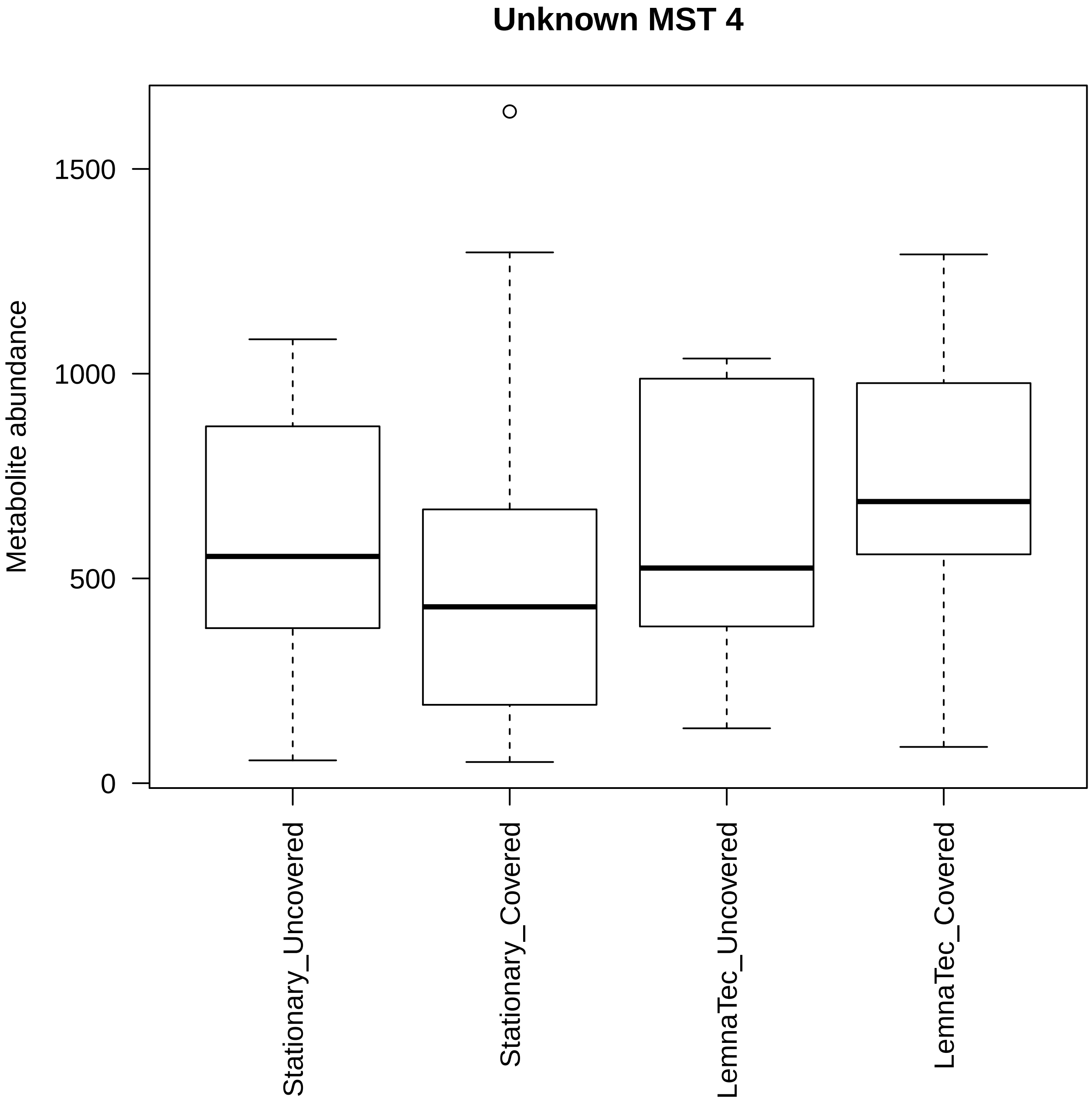

Unknown MST 4
Metabolite abundance
1500
1000
500
0
Stationary_Uncovered
Stationary_Covered
LemnaTec_Uncovered
LemnaTec_Covered

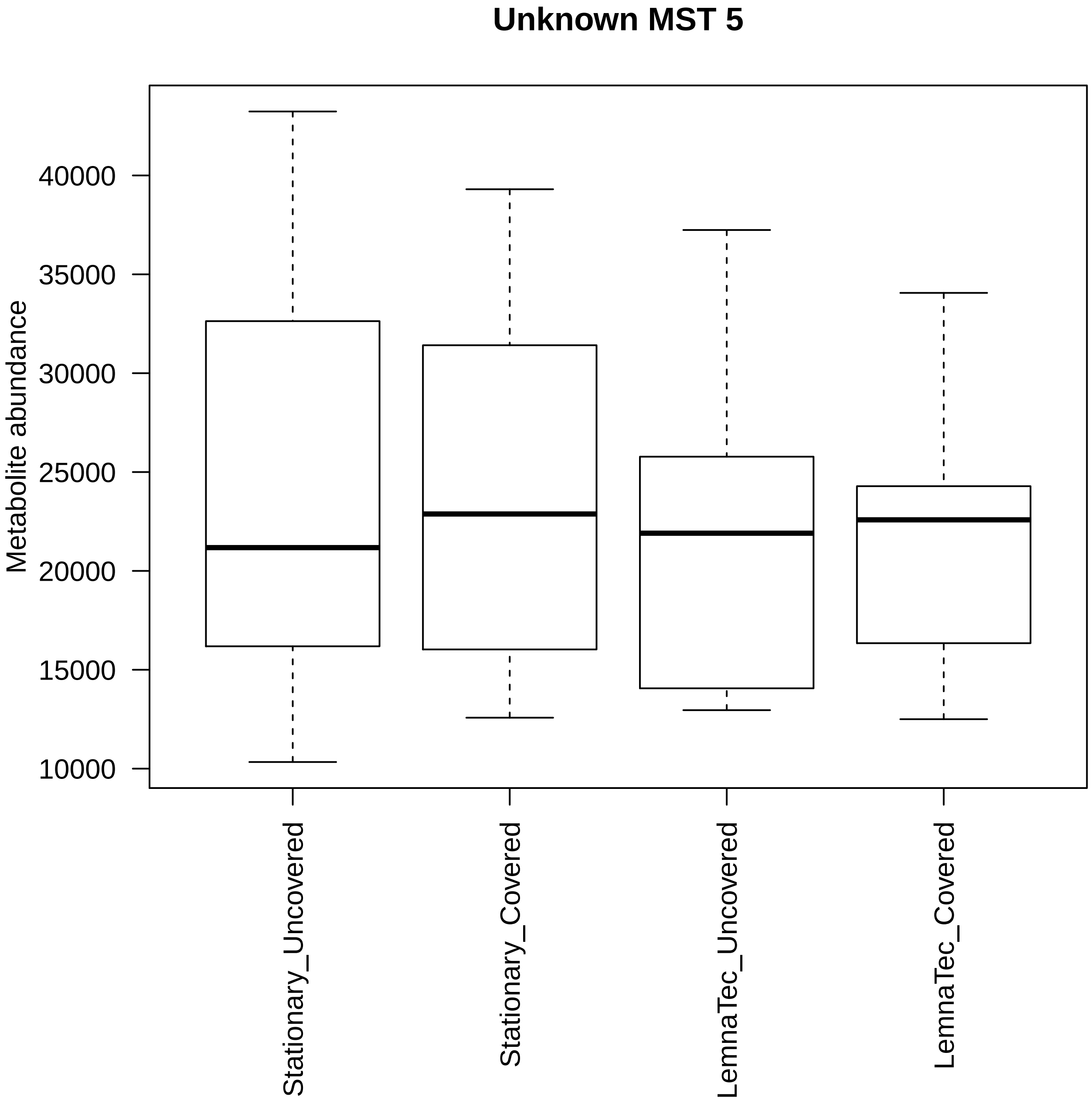

Unknown MST 5
Metabolite abundance
40000
35000
30000
25000
20000
15000
10000
Stationary_Uncovered
Stationary_Covered
LemnaTec_Uncovered
LemnaTec_Covered

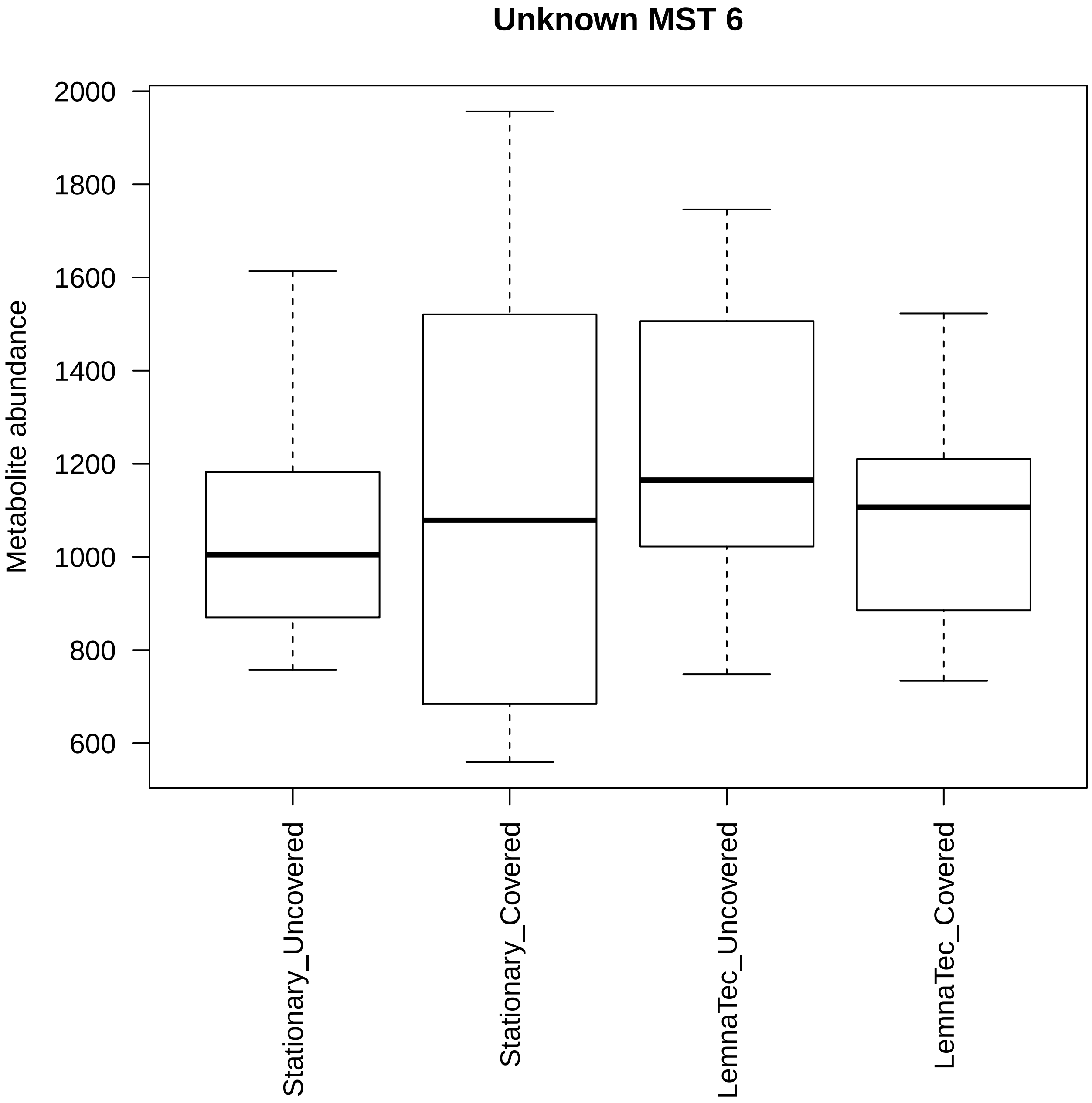
Unknown MST 6
Metabolite abundance
2000
1800
1600
1400
1200
1000
800
600
Stationary_Uncovered
Stationary_Covered
LemnaTec_Uncovered
LemnaTec_Covered

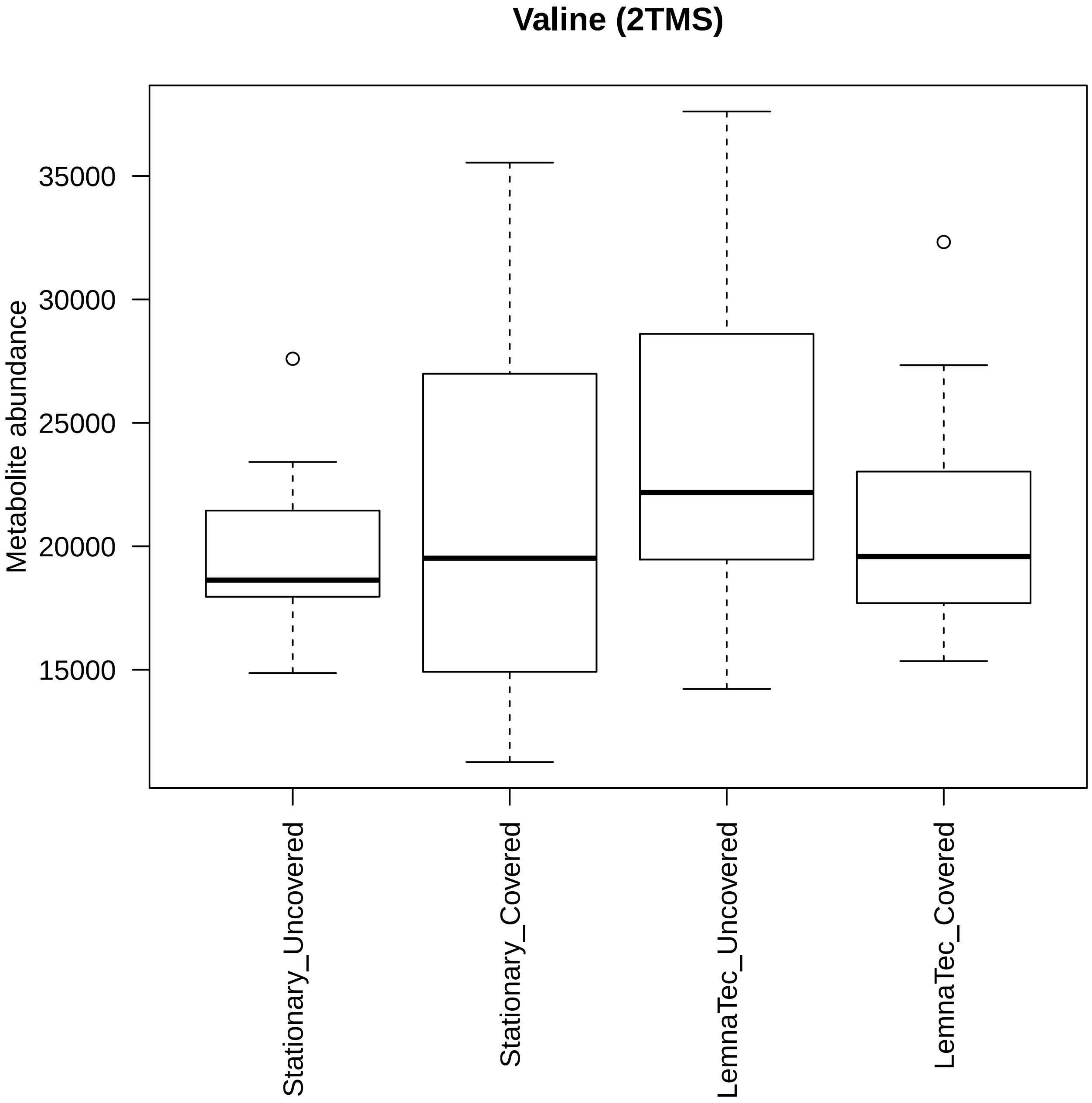
Valine (2TMS)
Metabolite abundance
35000
30000
25000
20000
15000
Stationary_Uncovered
Stationary_Covered
LemnaTec_Uncovered
LemnaTec_Covered

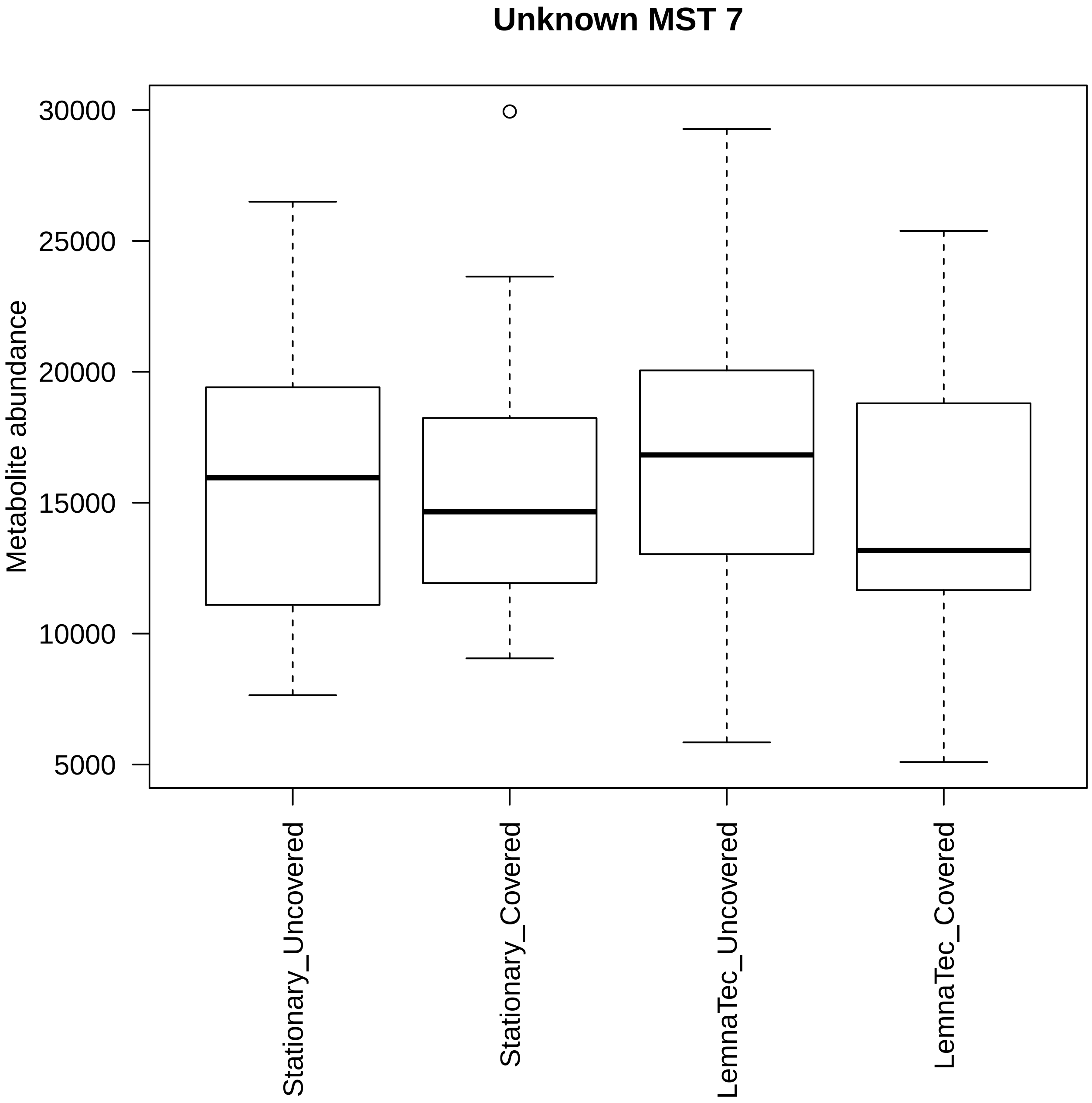

Unknown MST 7
Metabolite abundance
30000
25000
20000
15000
10000
5000
Stationary_Uncovered
Stationary_Covered
LemnaTec_Uncovered
LemnaTec_Covered

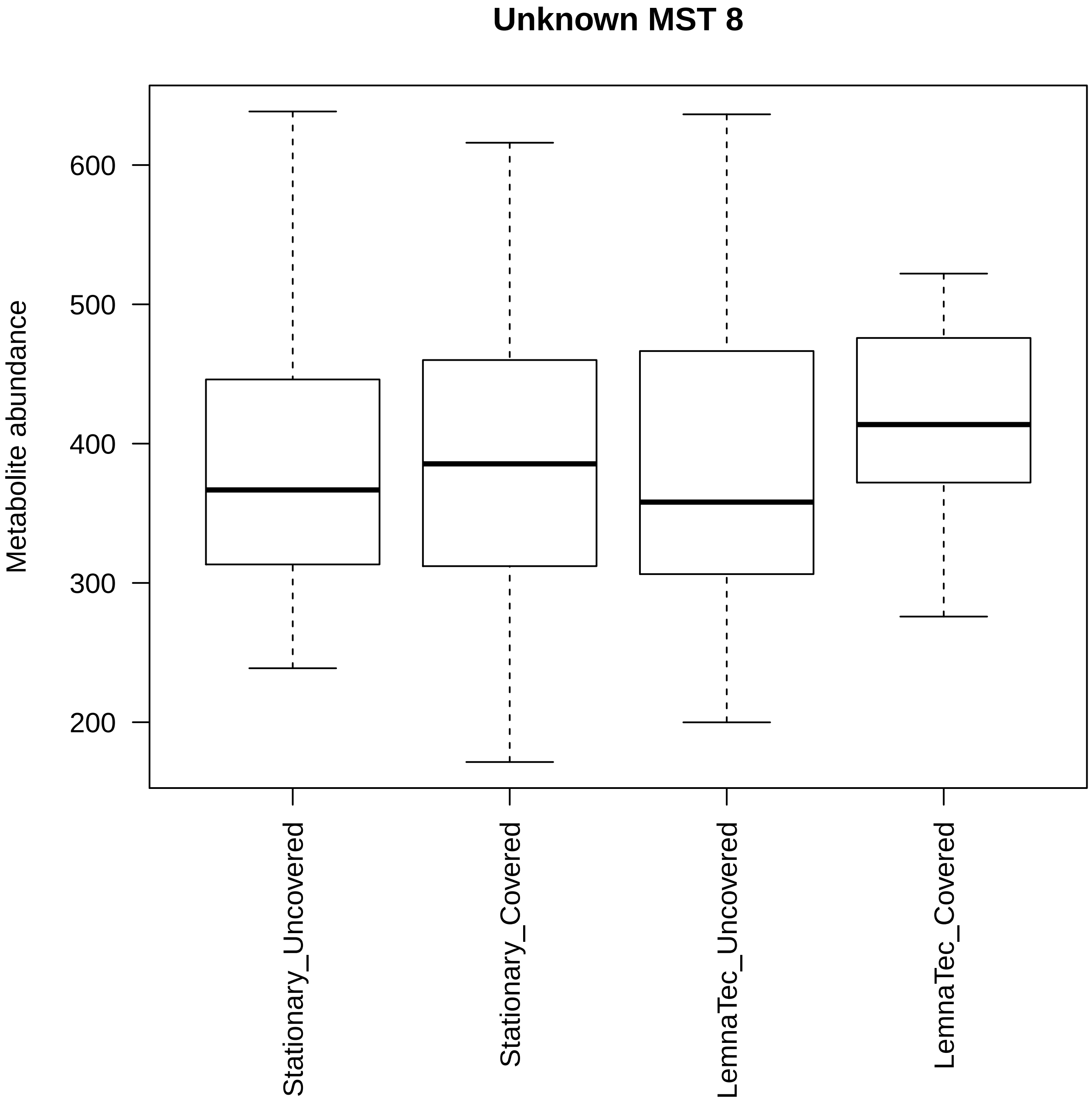

Unknown MST 8
Metabolite abundance
600
500
400
300
200
Stationary_Uncovered
Stationary_Covered
LemnaTec_Uncovered
LemnaTec_Covered

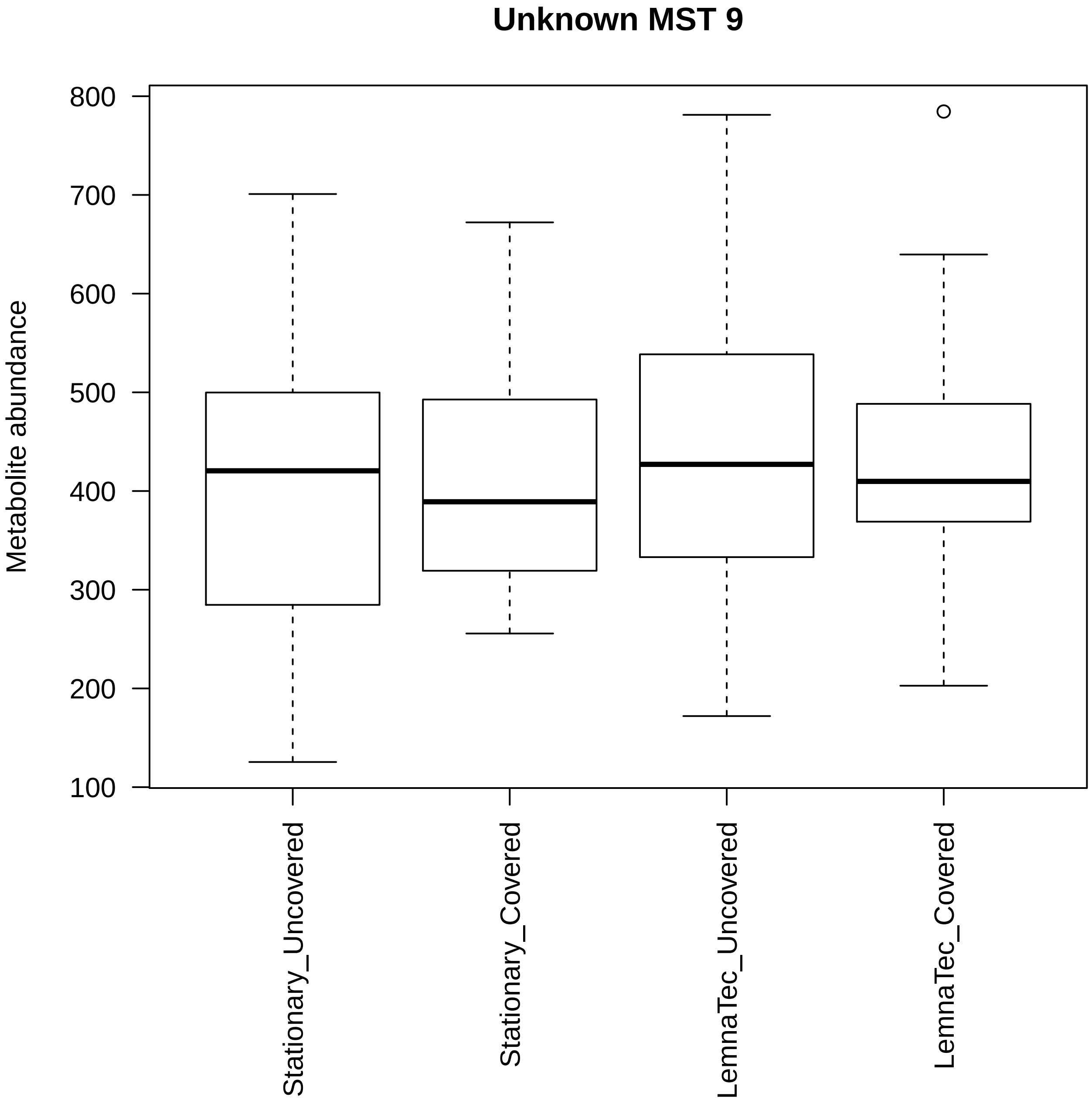
Unknown MST 9
Metabolite abundance
800
700
600
500
400
300
200
100
Stationary_Uncovered
Stationary_Covered
LemnaTec_Uncovered
LemnaTec_Covered

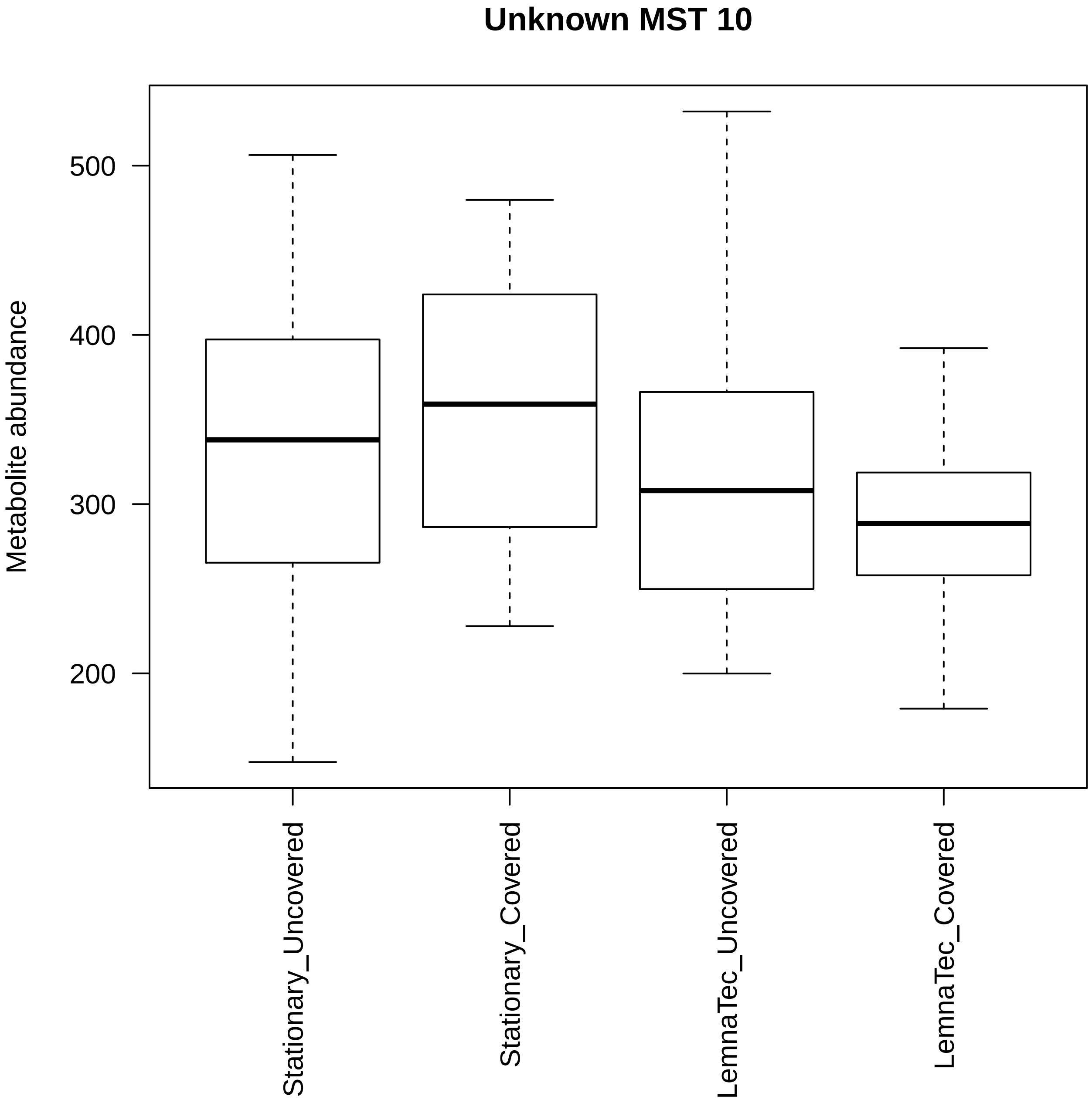

Unknown MST 10
Metabolite abundance
500
400
300
200
Stationary_Uncovered
Stationary_Covered
LemnaTec_Uncovered
LemnaTec_Covered

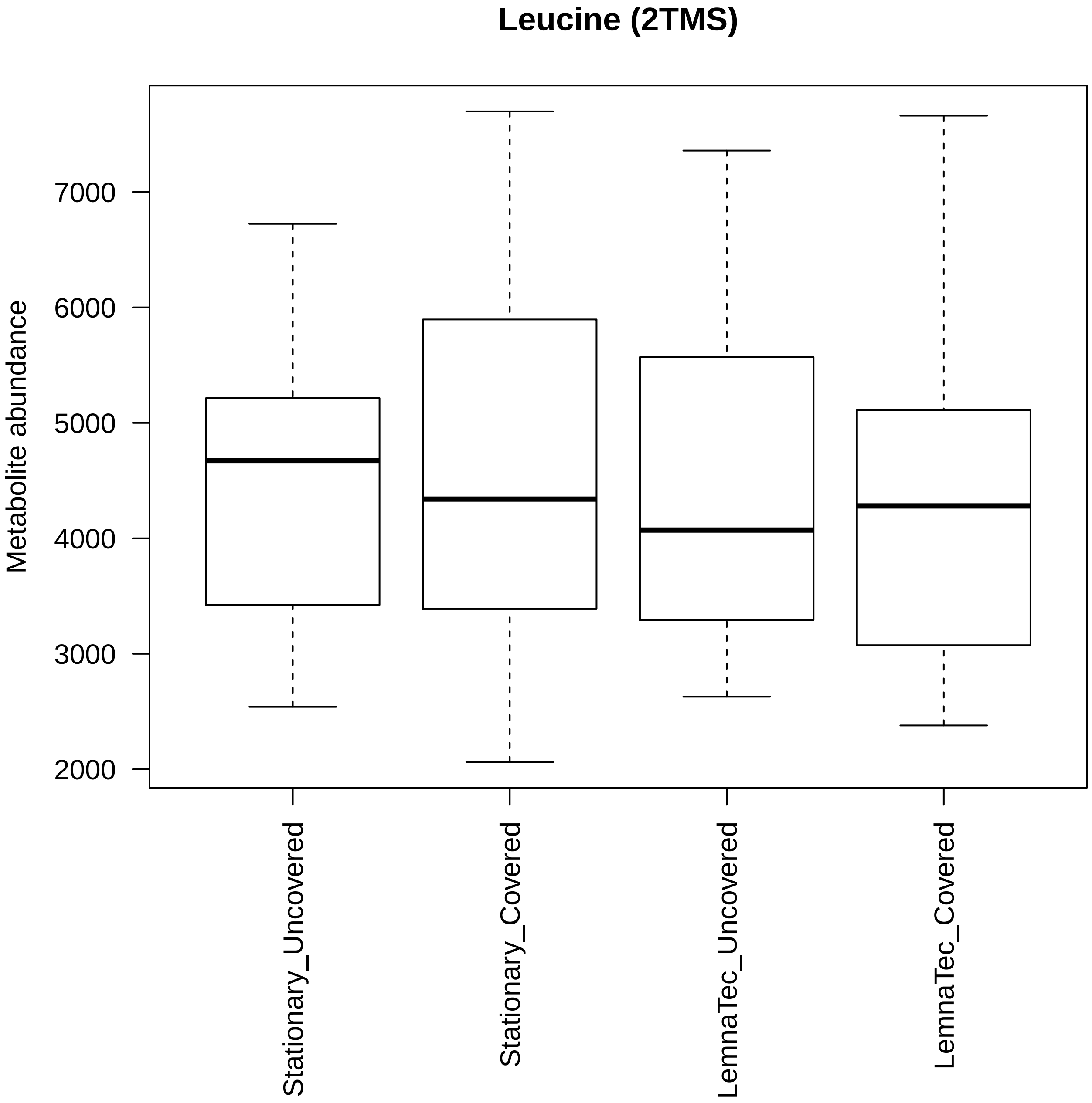
Leucine (2TMS)
Metabolite abundance
2000
3000
4000
5000
6000
7000
Stationary_Uncovered
Stationary_Covered
LemnaTec_Uncovered
LemnaTec_Covered

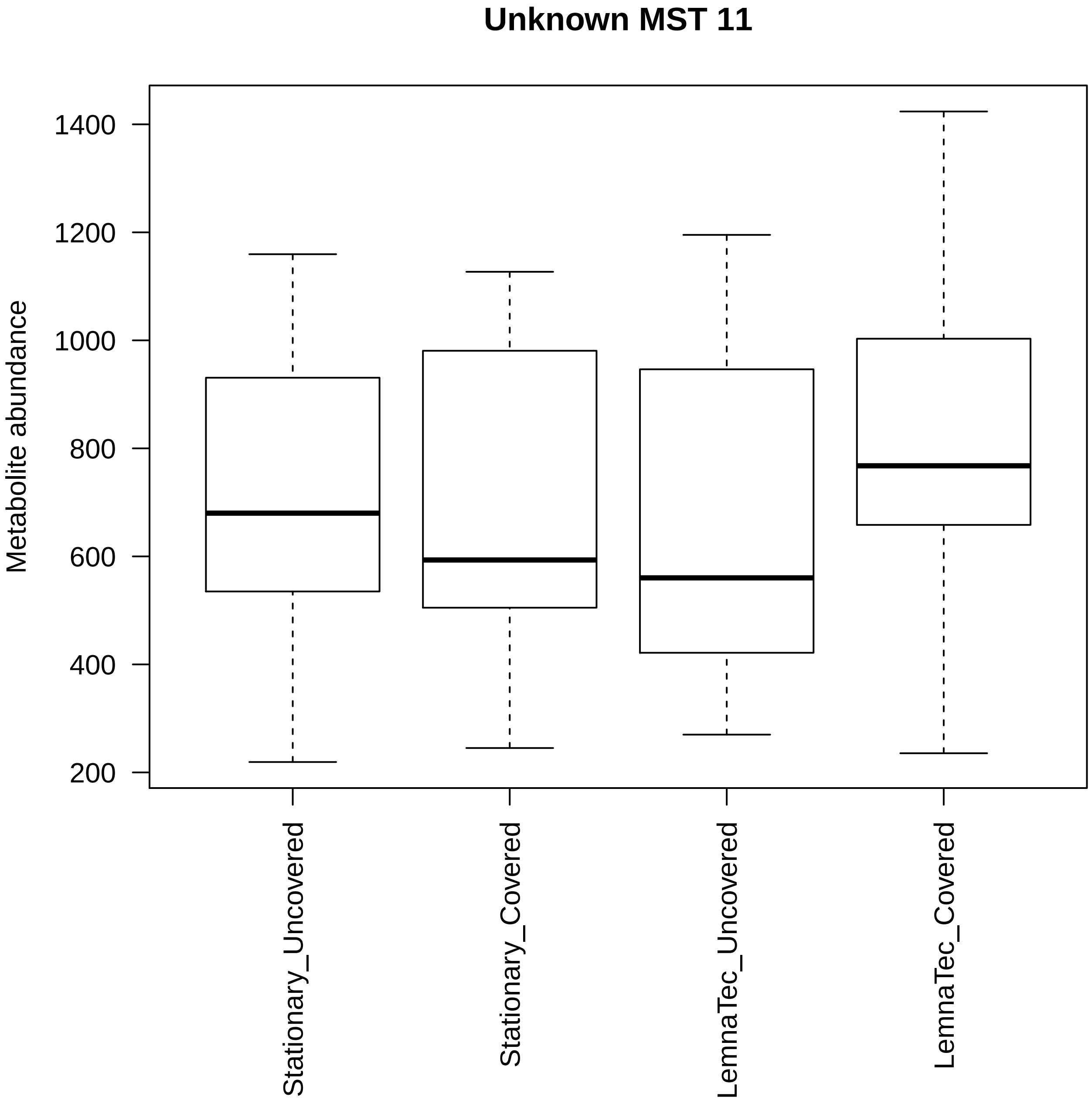

Unknown MST 11
Metabolite abundance
200
400
600
800
1000
1200
1400
Stationary_Uncovered
Stationary_Covered
LemnaTec_Uncovered
LemnaTec_Covered

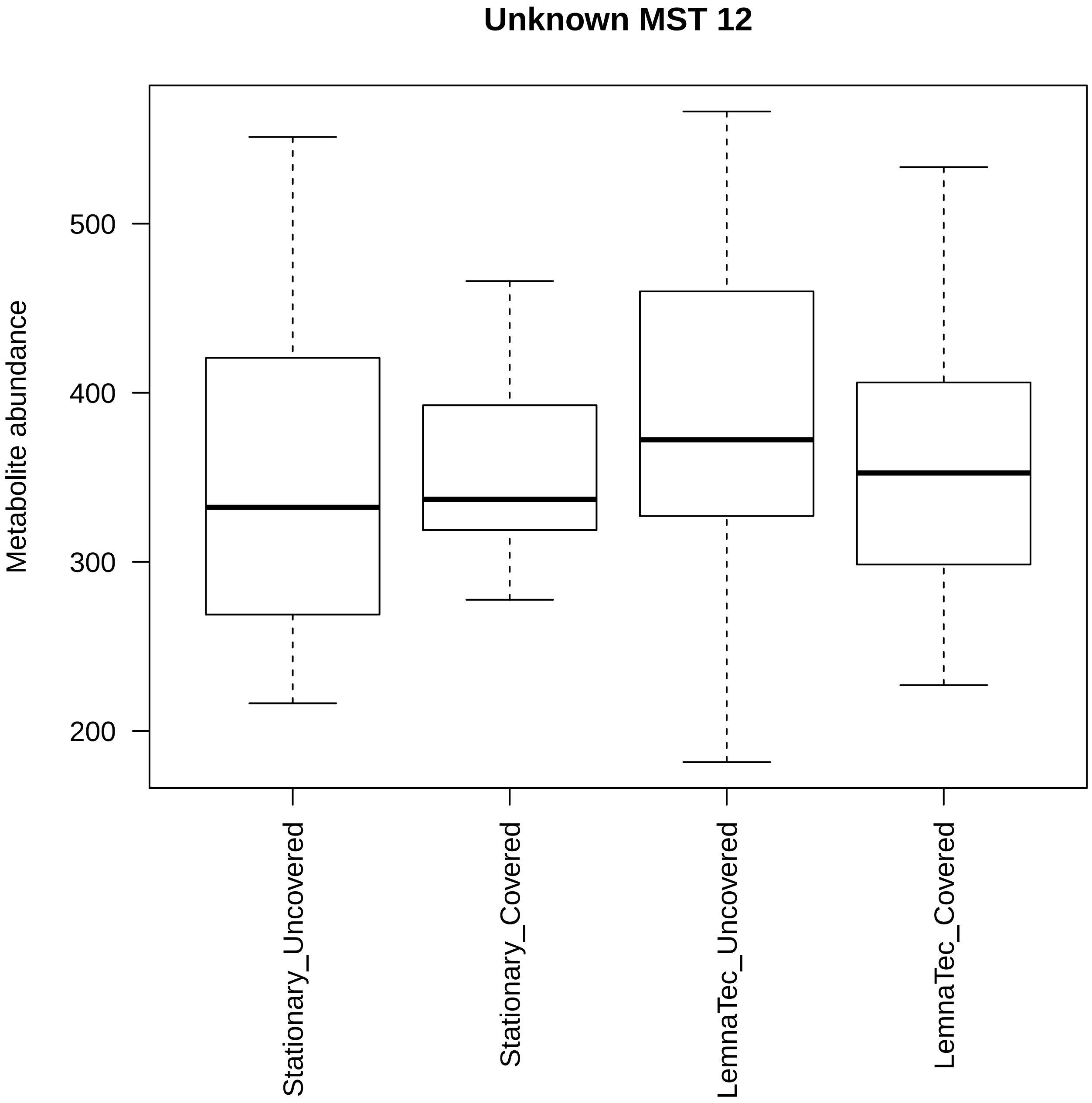
Unknown MST 12
Metabolite abundance
500
400
300
200
Stationary_Uncovered
Stationary_Covered
LemnaTec_Uncovered
LemnaTec_Covered

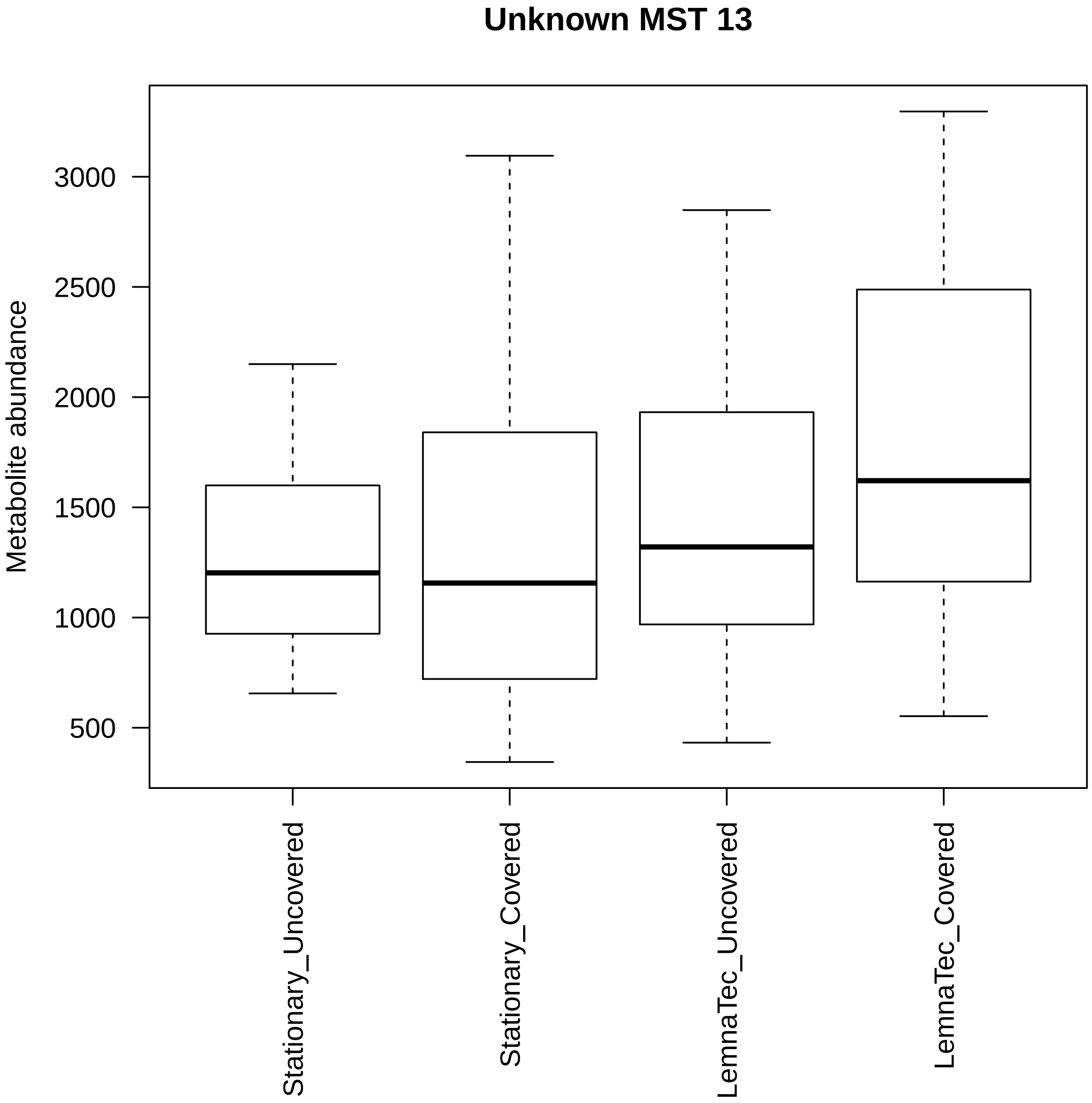
Unknown MST 13
Metabolite abundance
3000
2500
2000
1500
1000
500
Stationary_Uncovered
Stationary_Covered
LemnaTec_Uncovered
LemnaTec_Covered

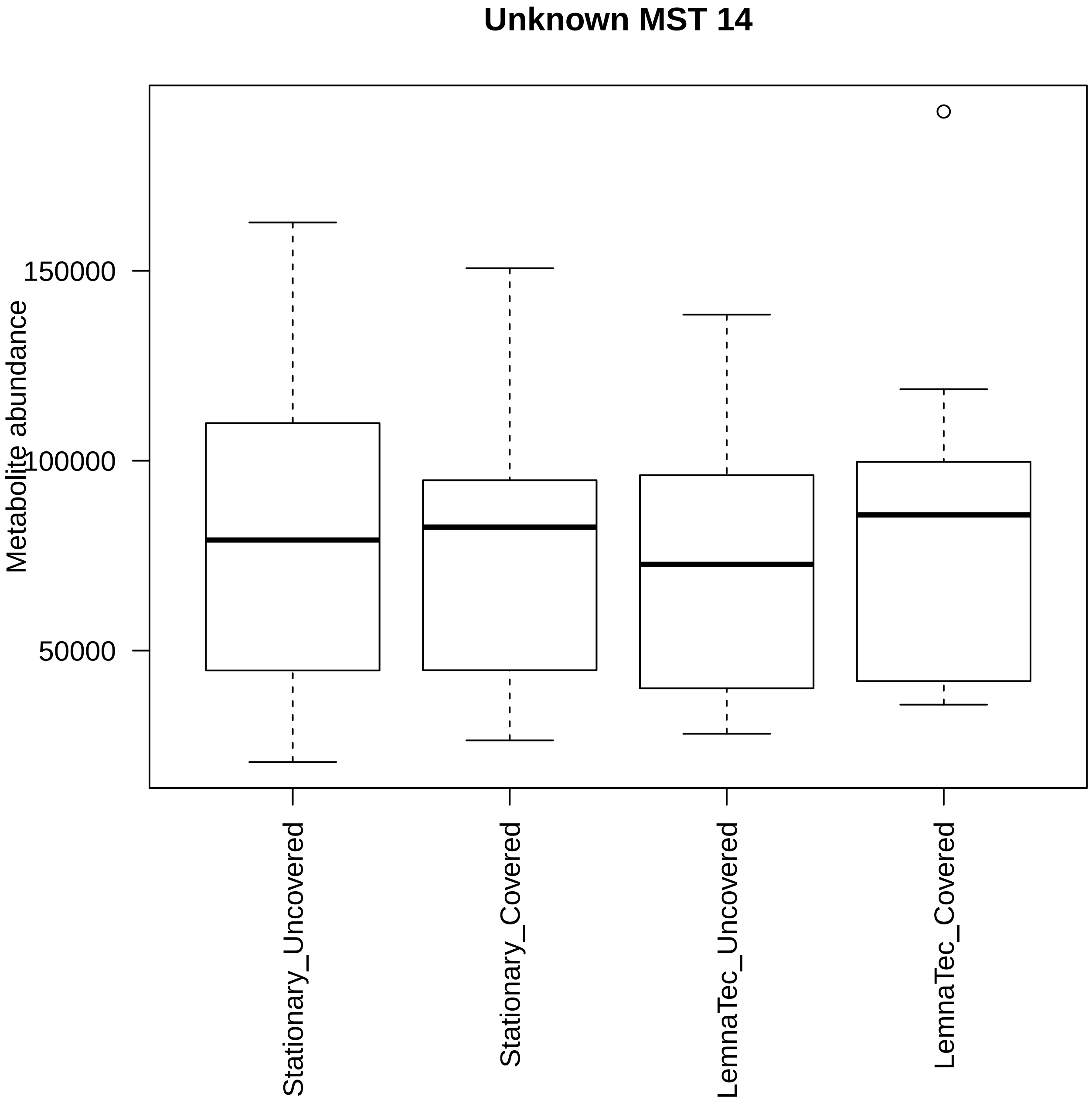

Unknown MST 14
Metabolite abundance
150000
100000
50000
Stationary_Uncovered
Stationary_Covered
LemnaTec_Uncovered
LemnaTec_Covered

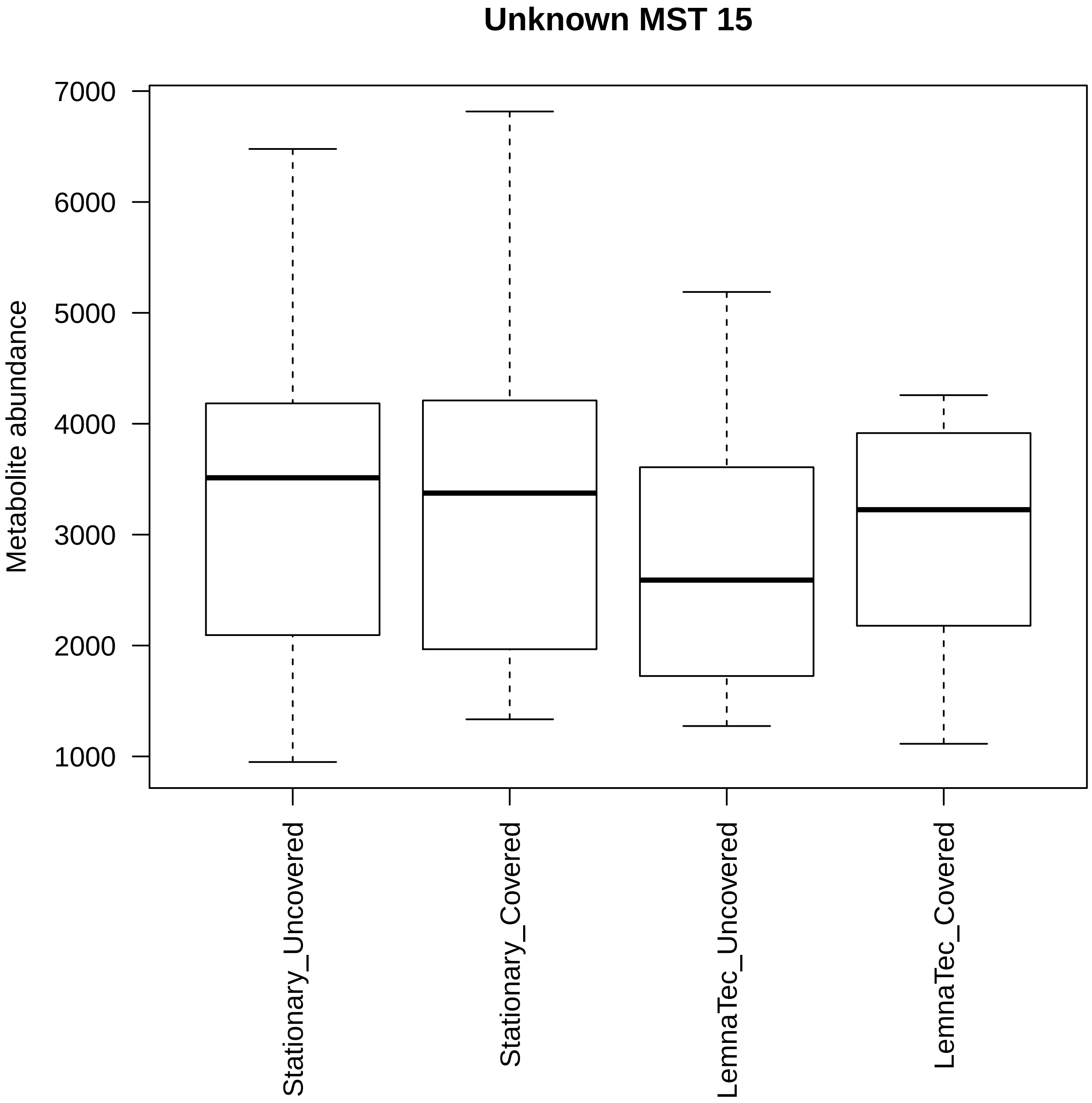

Unknown MST 15
Metabolite abundance
7000
6000
5000
4000
3000
2000
1000
Stationary_Uncovered
Stationary_Covered
LemnaTec_Uncovered
LemnaTec_Covered

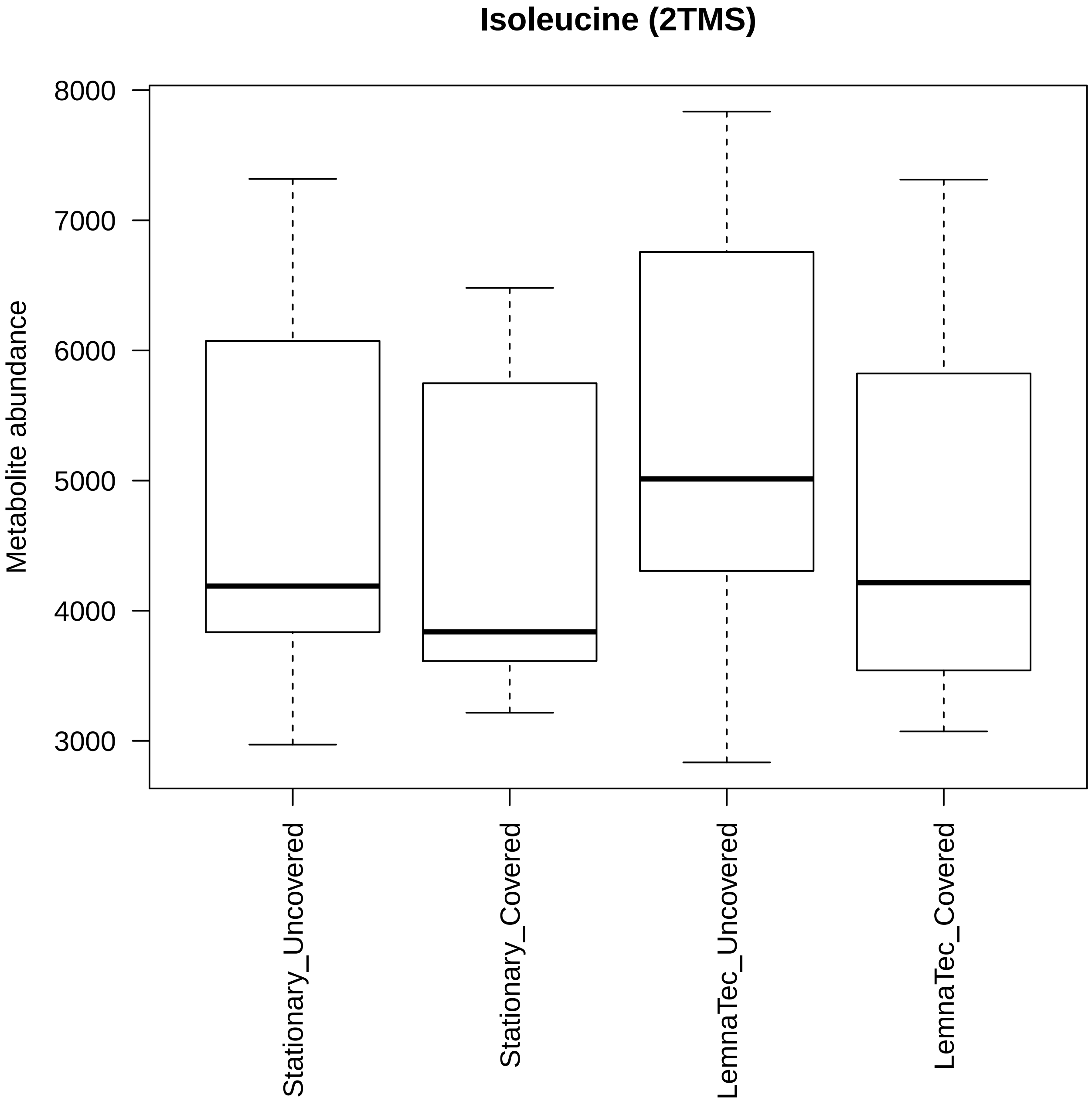

Isoleucine (2TMS)
Metabolite abundance
8000
7000
6000
5000
4000
3000
Stationary_Uncovered
Stationary_Covered
LemnaTec_Uncovered
LemnaTec_Covered

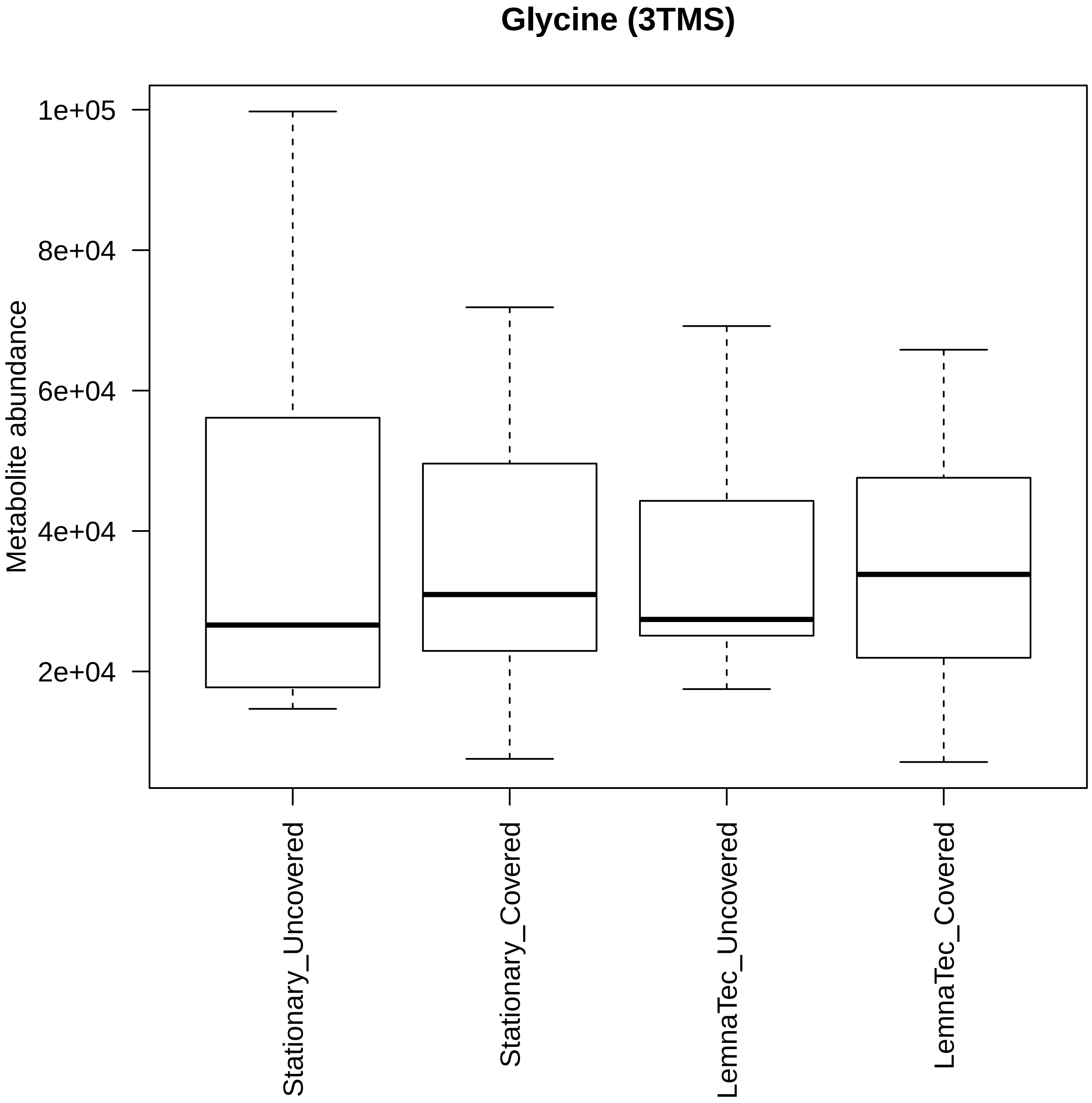

Glycine (3TMS)
Metabolite abundance
1e+05
8e+04
6e+04
4e+04
2e+04
Stationary_Uncovered
Stationary_Covered
LemnaTec_Uncovered
LemnaTec_Covered

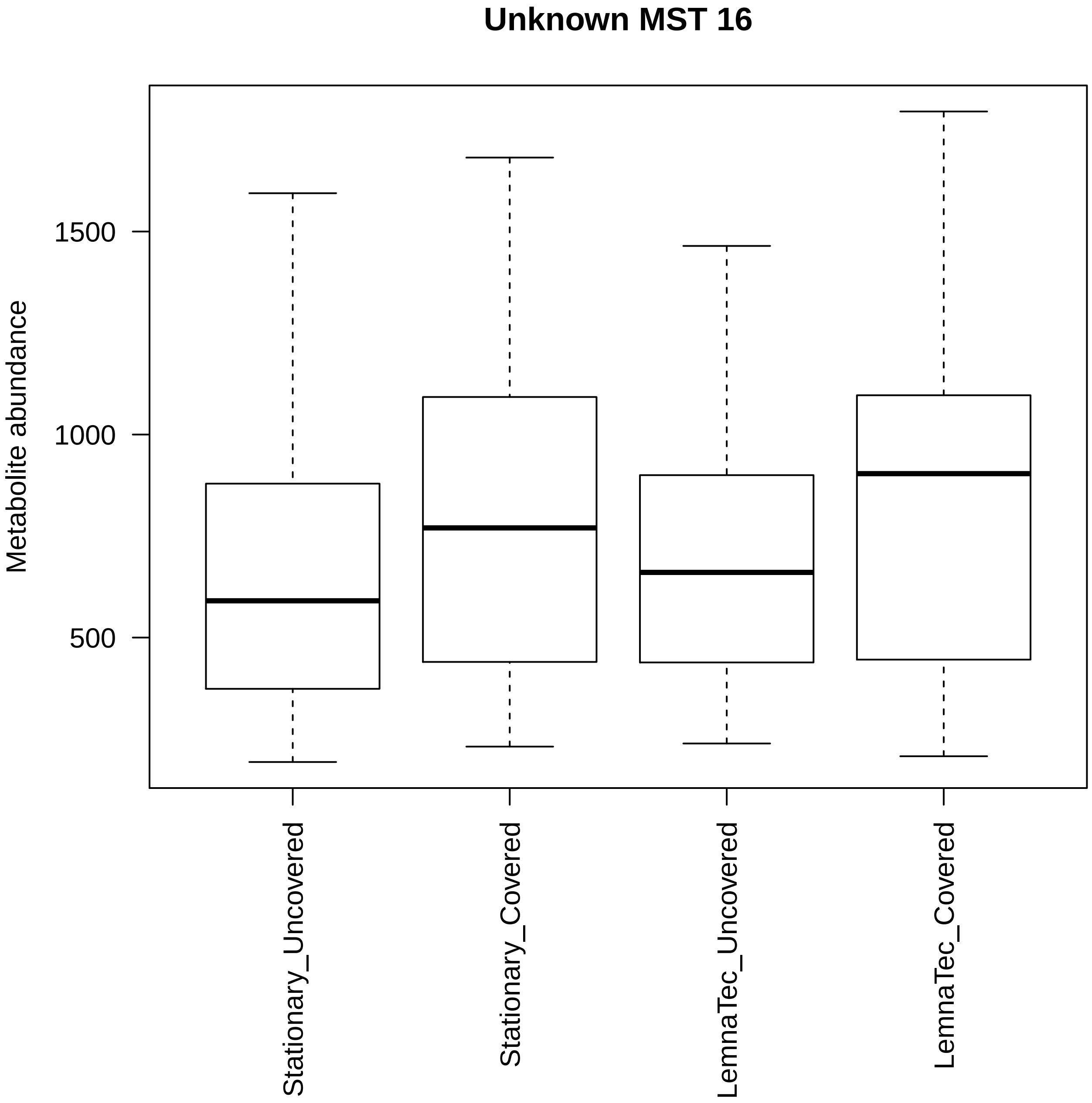

Unknown MST 16
Metabolite abundance
1500
1000
500
Stationary_Uncovered
Stationary_Covered
LemnaTec_Uncovered
LemnaTec_Covered

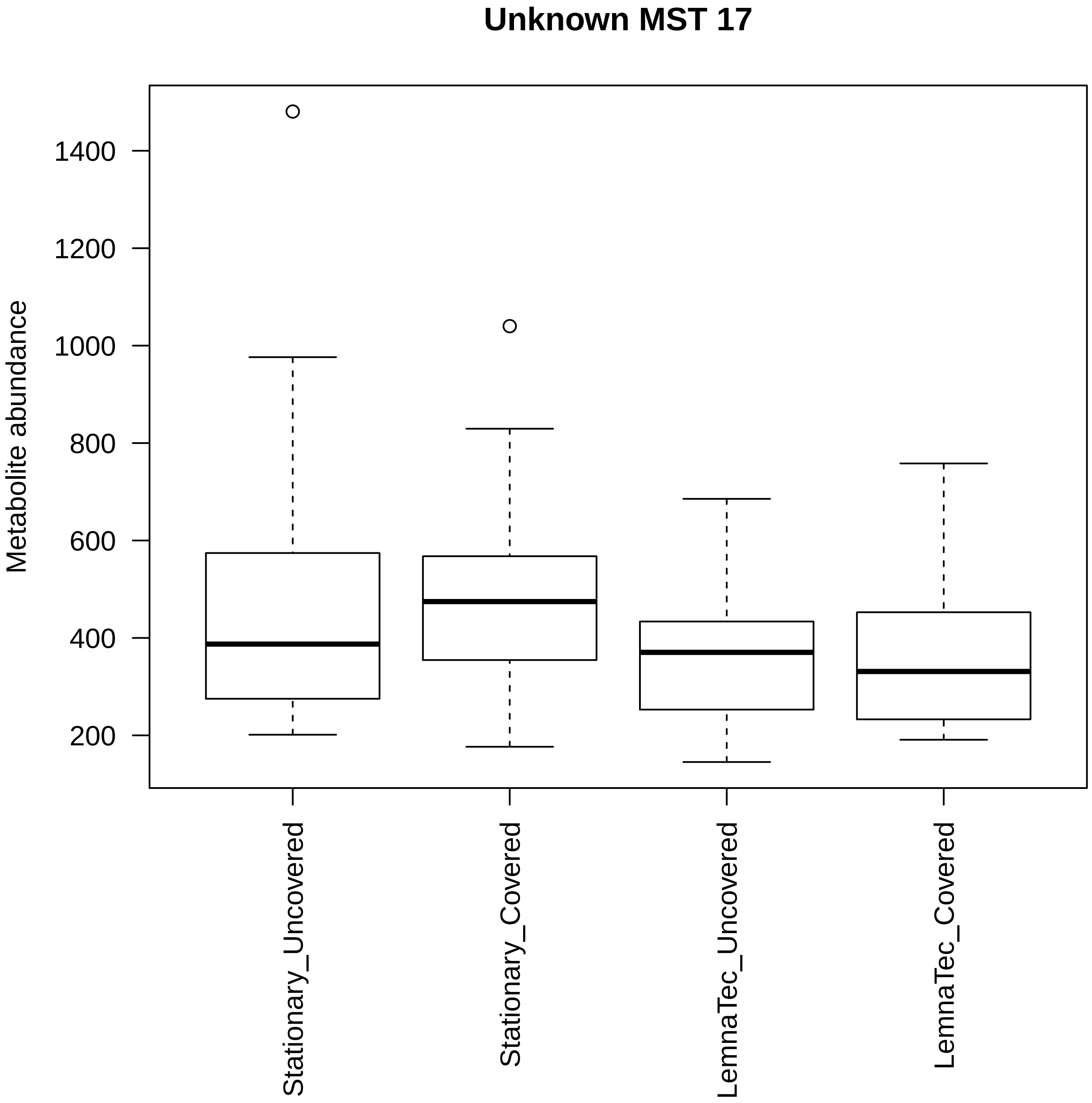

Unknown MST 17
Metabolite abundance
1400
1200
1000
800
600
400
200
Stationary_Uncovered
Stationary_Covered
LemnaTec_Uncovered
LemnaTec_Covered

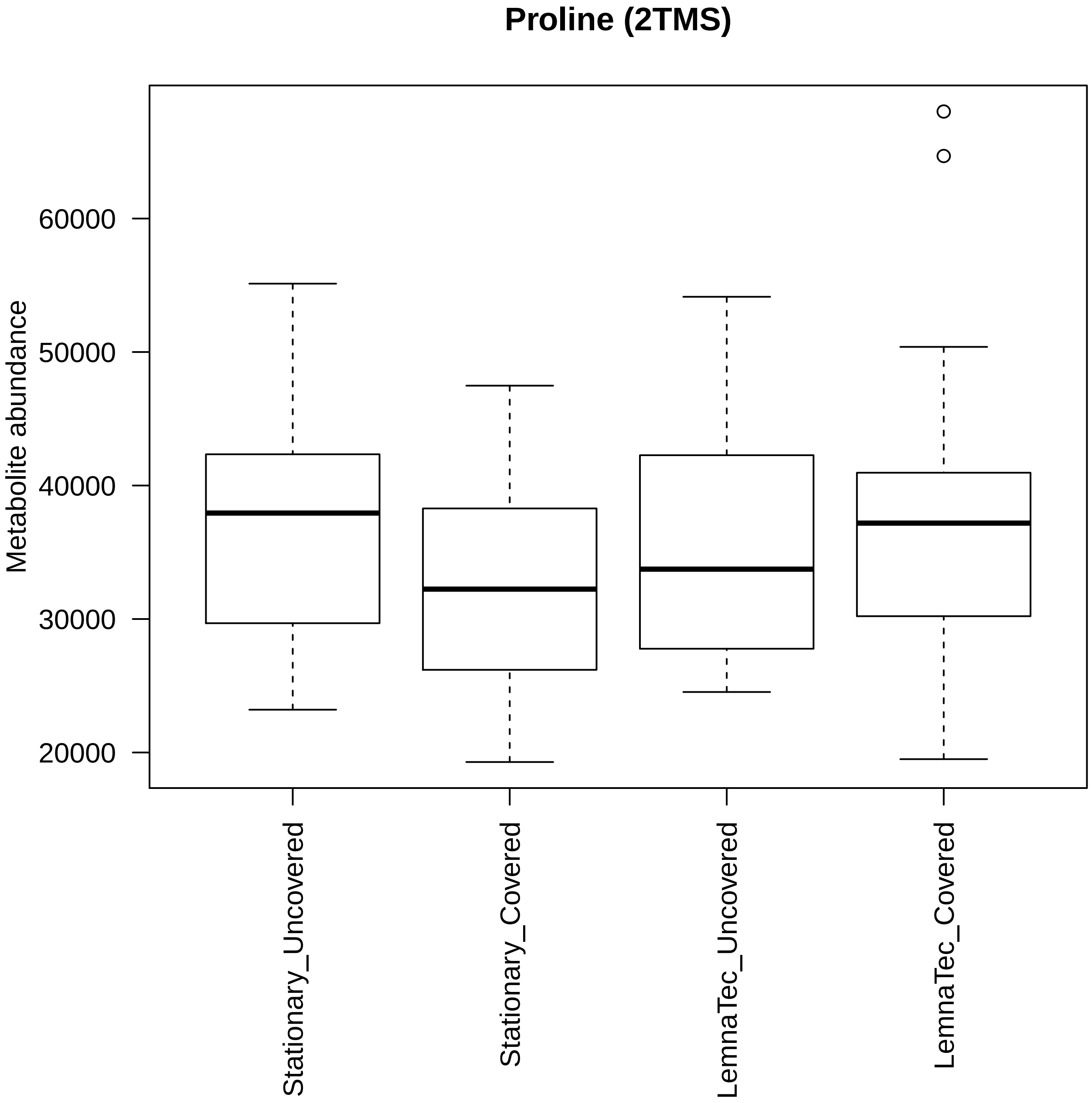
Proline (2TMS)
Metabolite abundance
60000
50000
40000
30000
20000
Stationary_Uncovered
Stationary_Covered
LemnaTec_Uncovered
LemnaTec_Covered

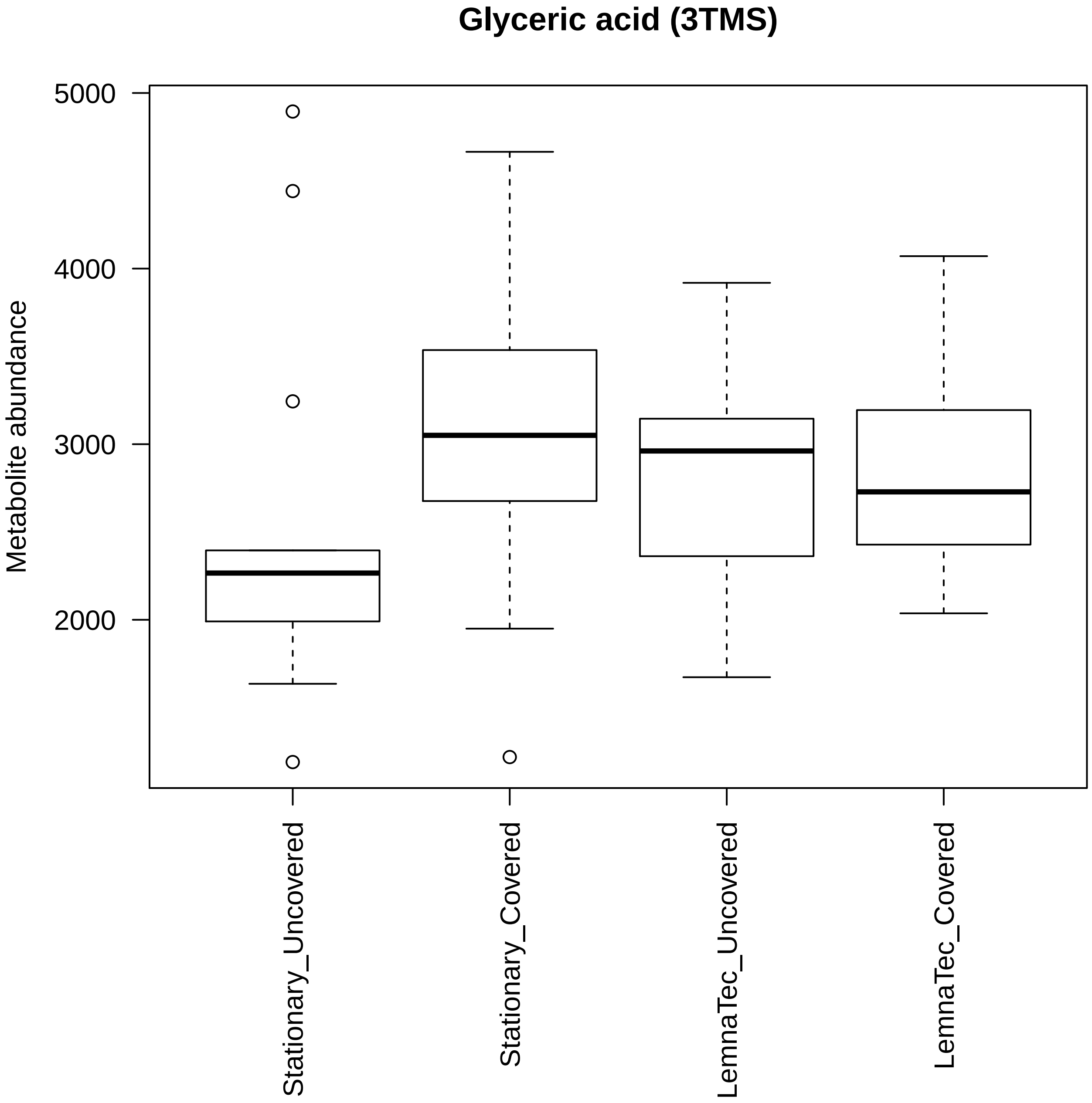

Glyceric acid (3TMS)
Metabolite abundance
5000
4000
3000
2000
Stationary_Uncovered
Stationary_Covered
LemnaTec_Uncovered
LemnaTec_Covered

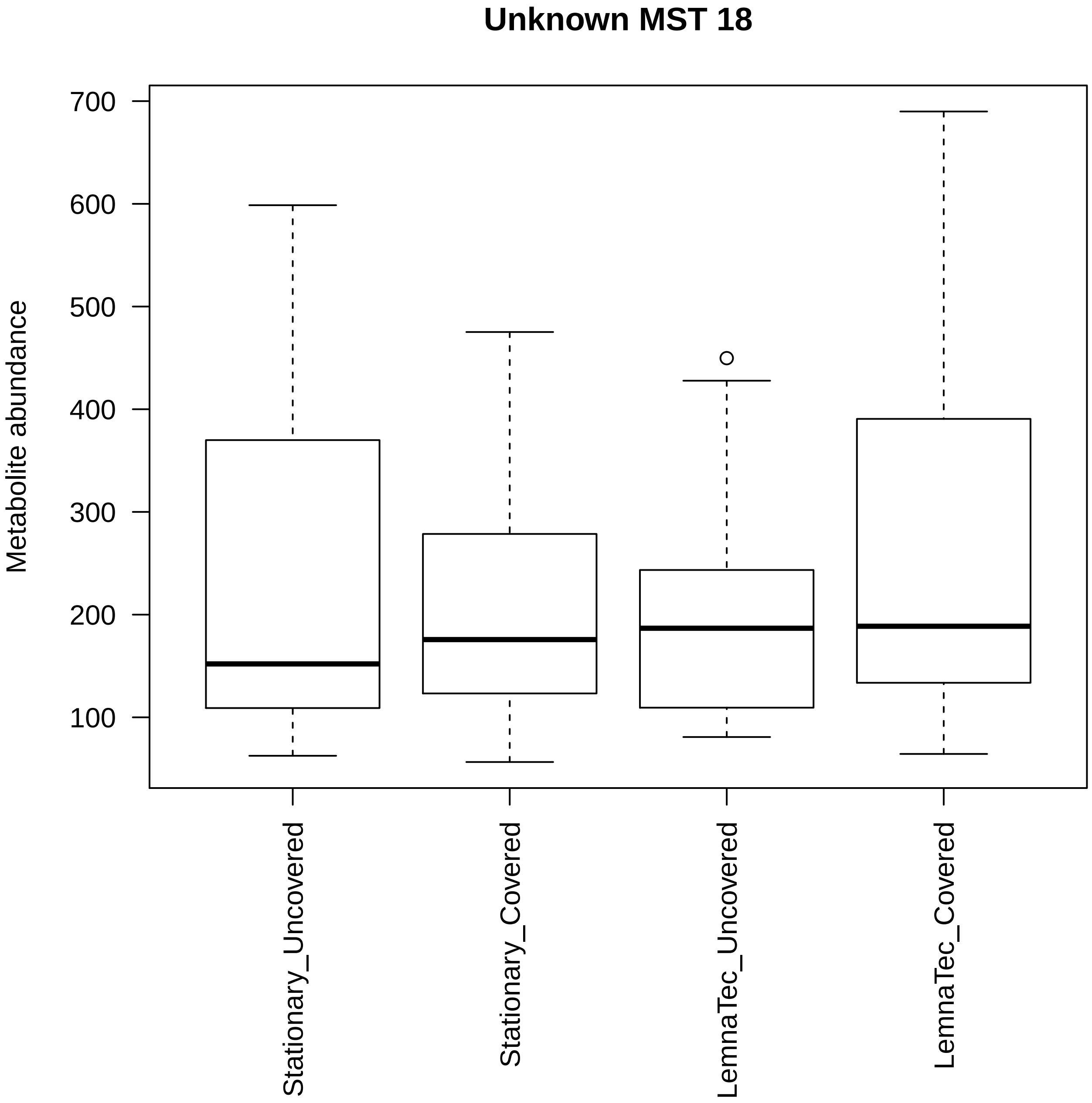

Unknown MST 18
Metabolite abundance
700
600
500
400
300
200
100
Stationary_Uncovered
Stationary_Covered
LemnaTec_Uncovered
LemnaTec_Covered

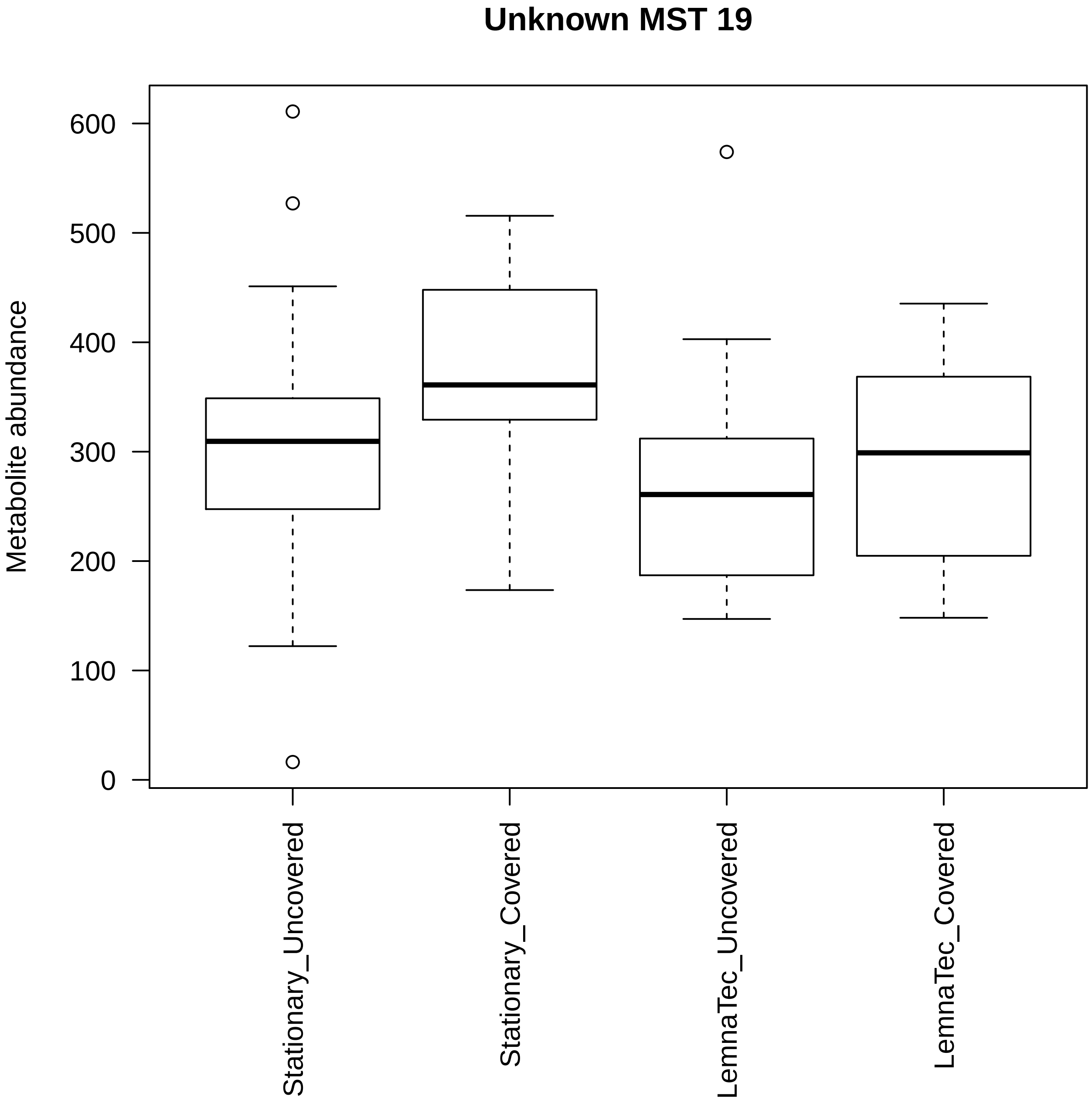
Unknown MST 19
Metabolite abundance
0
100
200
300
400
500
600
Stationary_Uncovered
Stationary_Covered
LemnaTec_Uncovered
LemnaTec_Covered

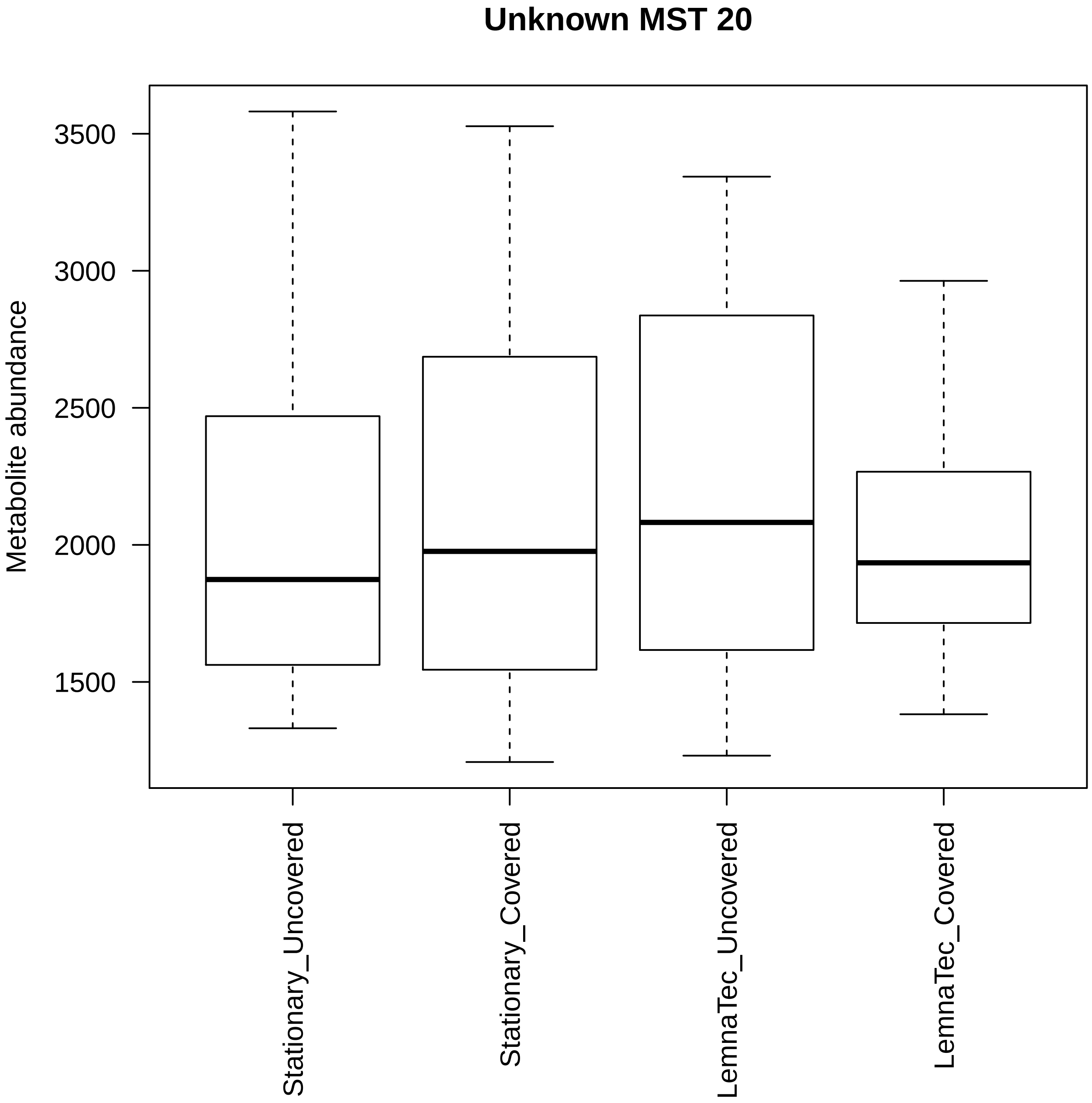
Unknown MST 20
Metabolite abundance
3500
3000
2500
2000
1500
Stationary_Uncovered
Stationary_Covered
LemnaTec_Uncovered
LemnaTec_Covered

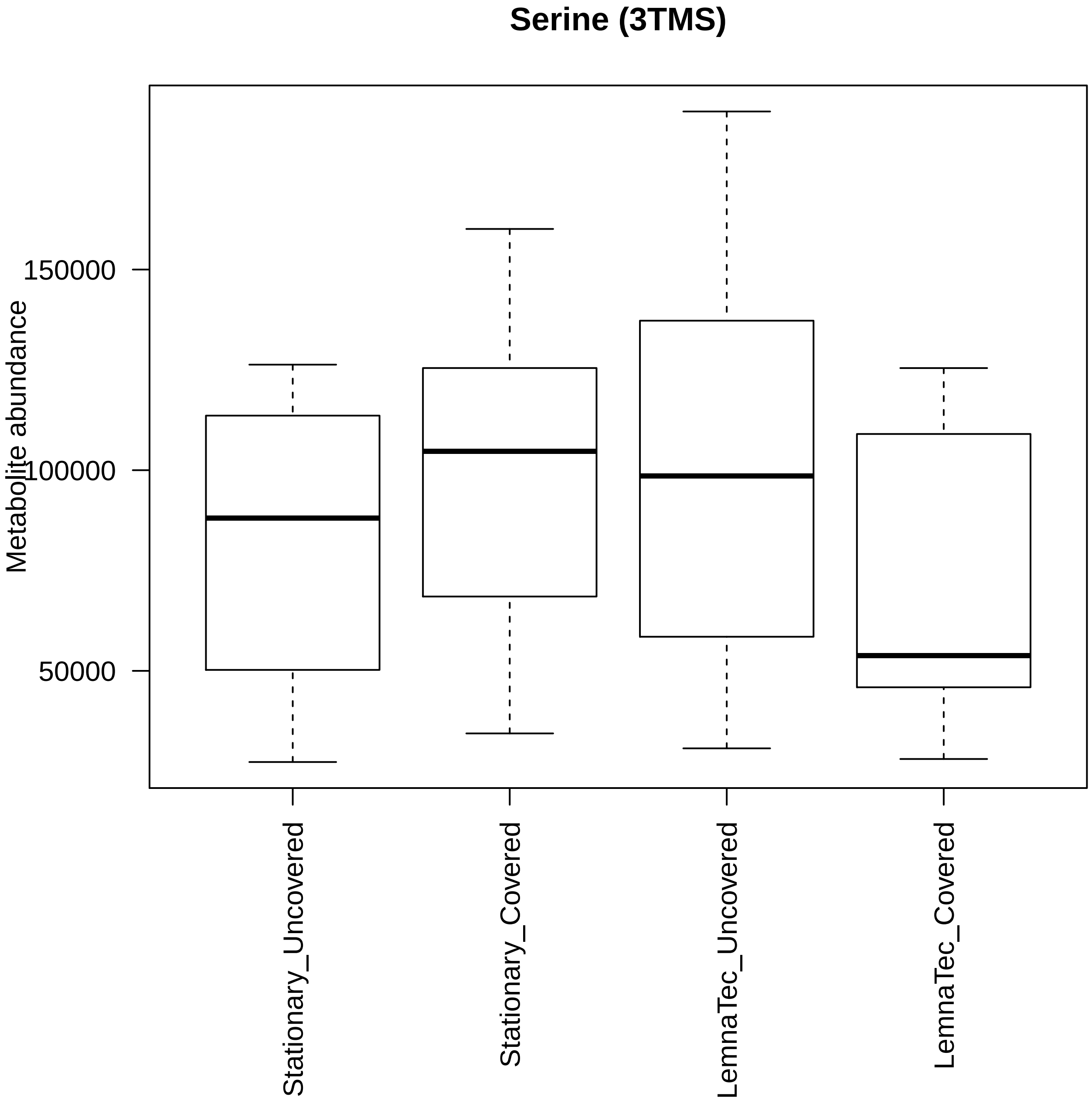
Serine (3TMS)
Metabolite abundance
150000
100000
50000
Stationary_Uncovered
Stationary_Covered
LemnaTec_Uncovered
LemnaTec_Covered

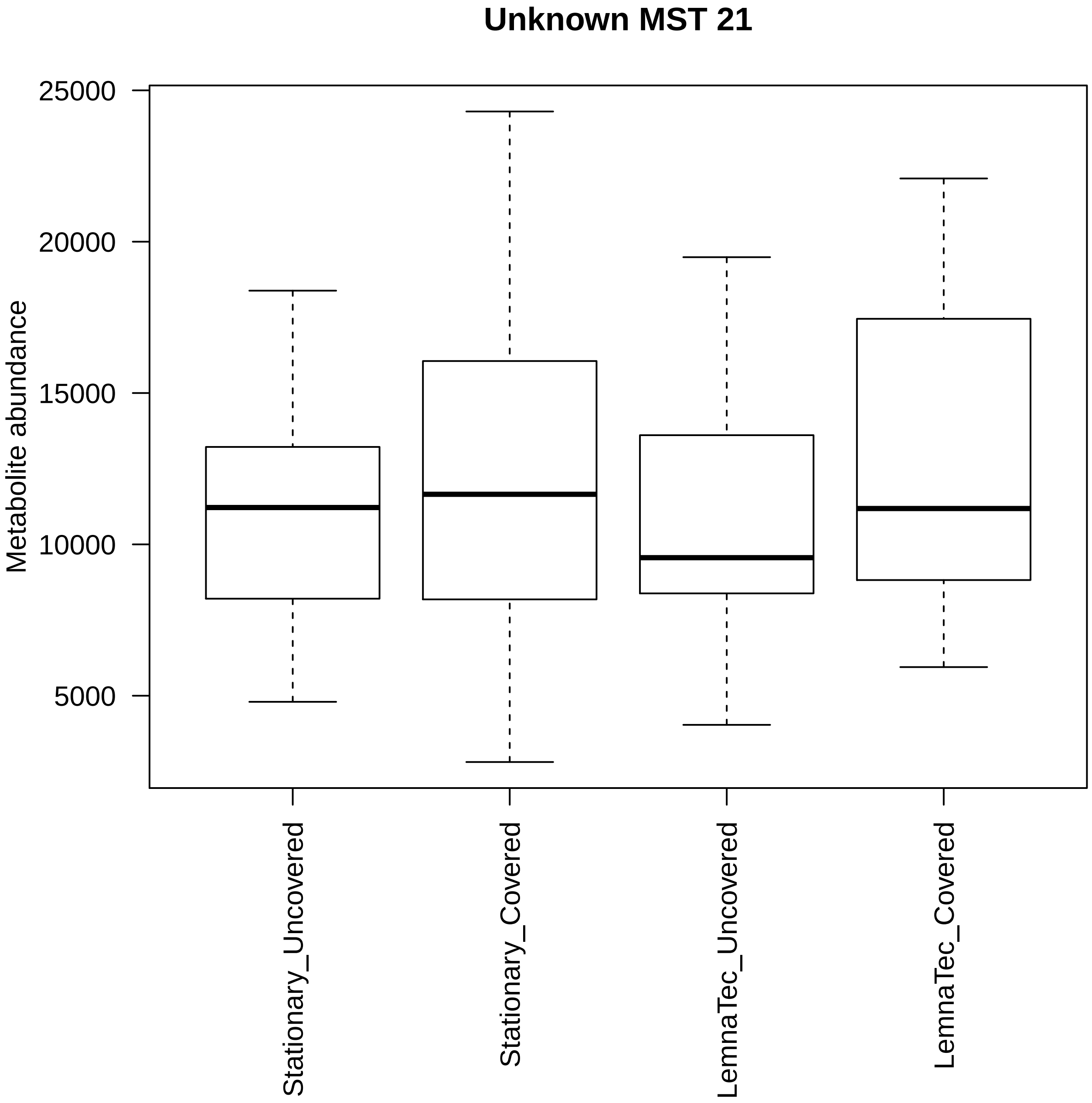
Unknown MST 21
Metabolite abundance
25000
20000
15000
10000
5000
Stationary_Uncovered
Stationary_Covered
LemnaTec_Uncovered
LemnaTec_Covered

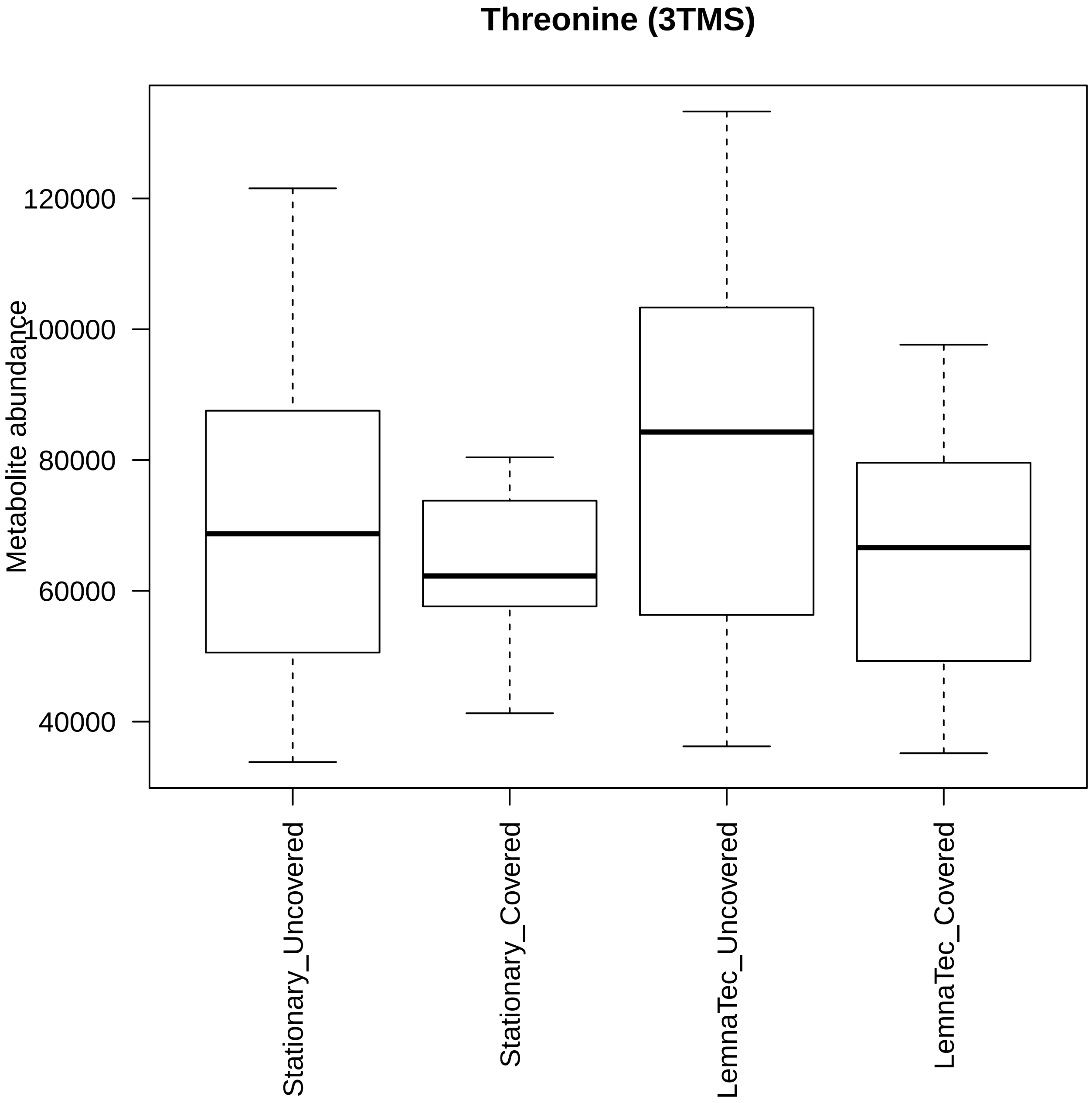
Threonine (3TMS)
Metabolite abundance
120000
100000
80000
60000
40000
Stationary_Uncovered
Stationary_Covered
LemnaTec_Uncovered
LemnaTec_Covered

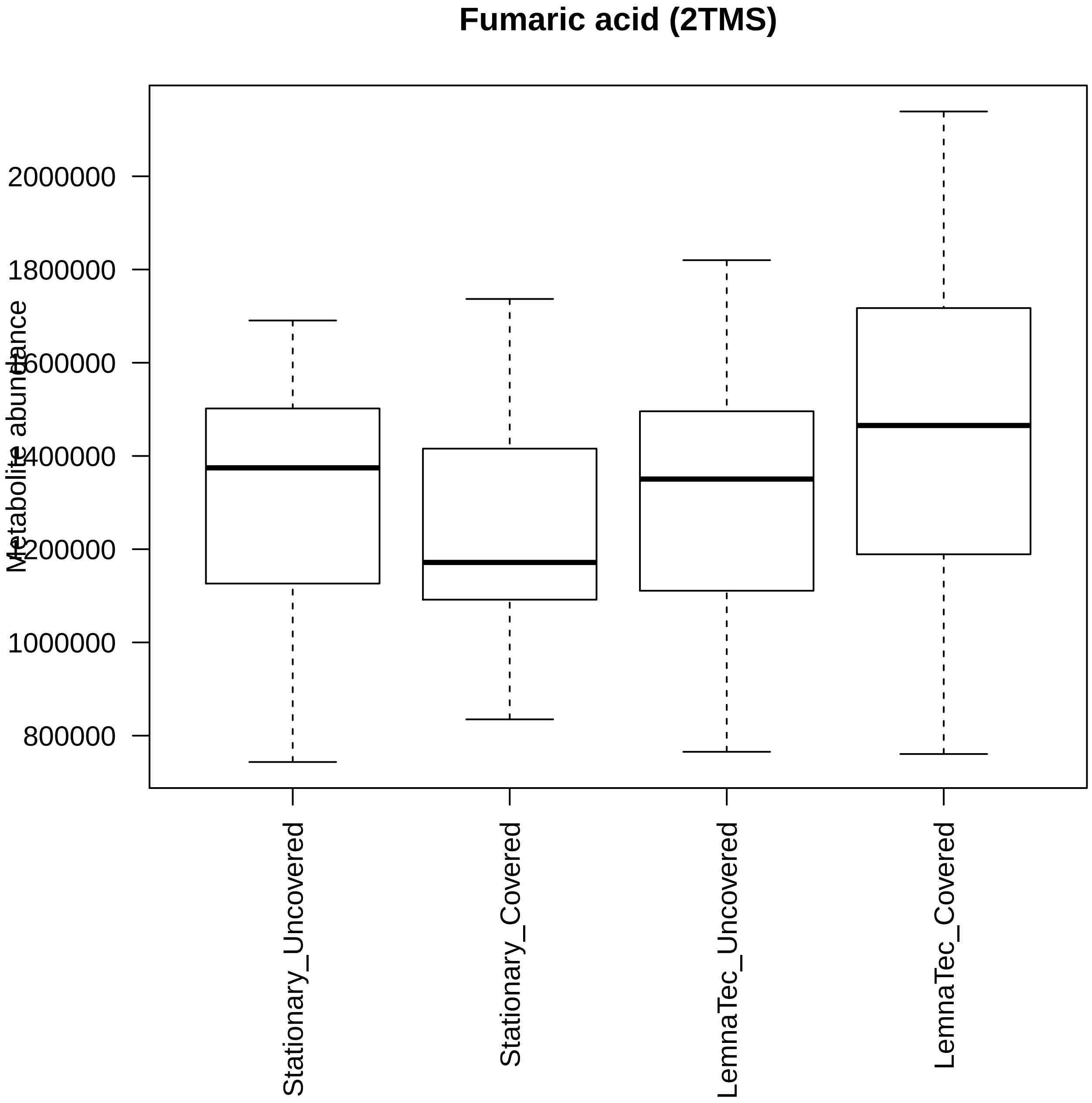
Fumaric acid (2TMS)
Metabolite abundance
2000000
1800000
1600000
1400000
1200000
1000000
800000
Stationary_Uncovered
Stationary_Covered
LemnaTec_Uncovered
LemnaTec_Covered

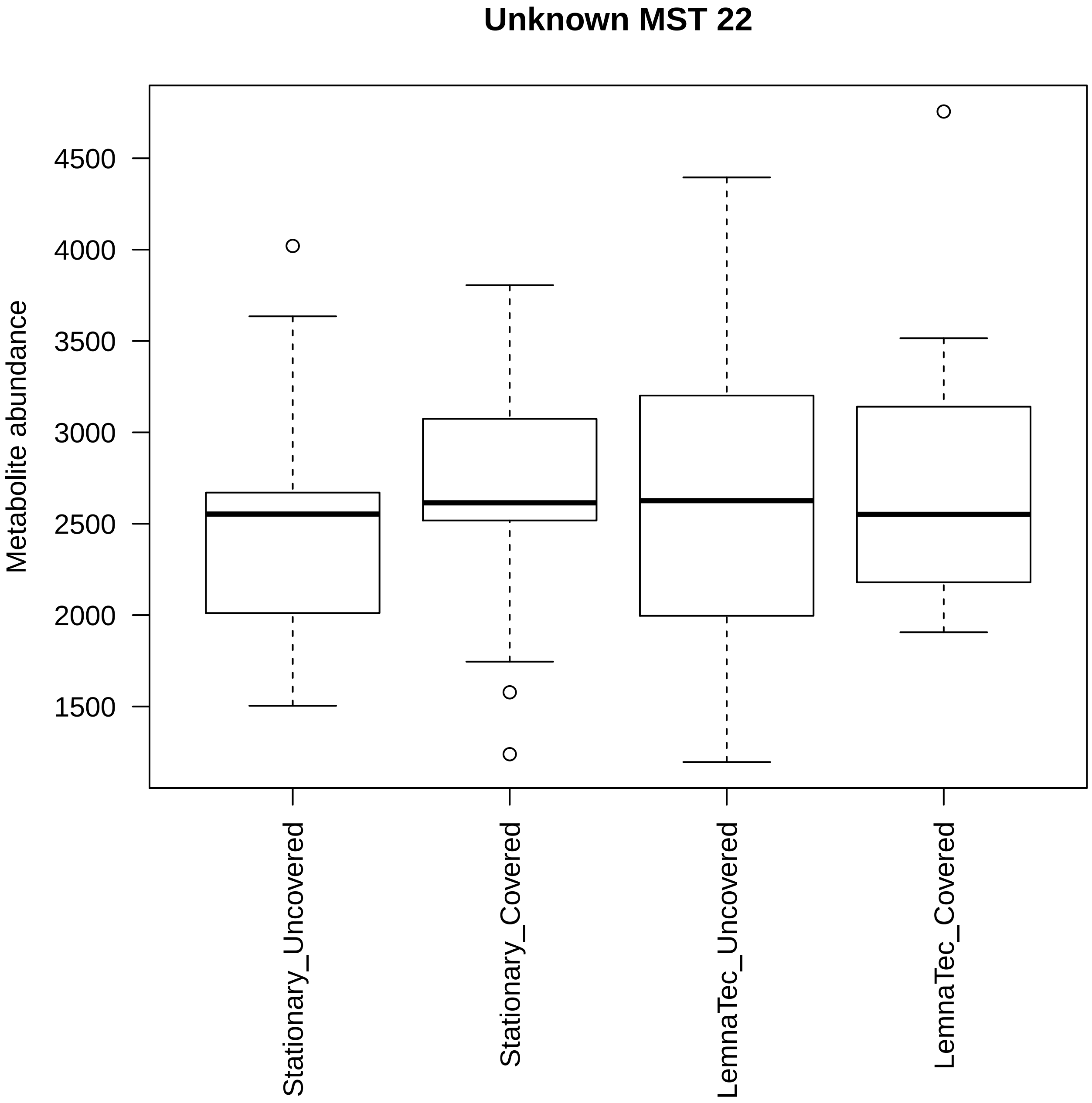

Unknown MST 22
Metabolite abundance
4500
4000
3500
3000
2500
2000
1500
Stationary_Uncovered
Stationary_Covered
LemnaTec_Uncovered
LemnaTec_Covered

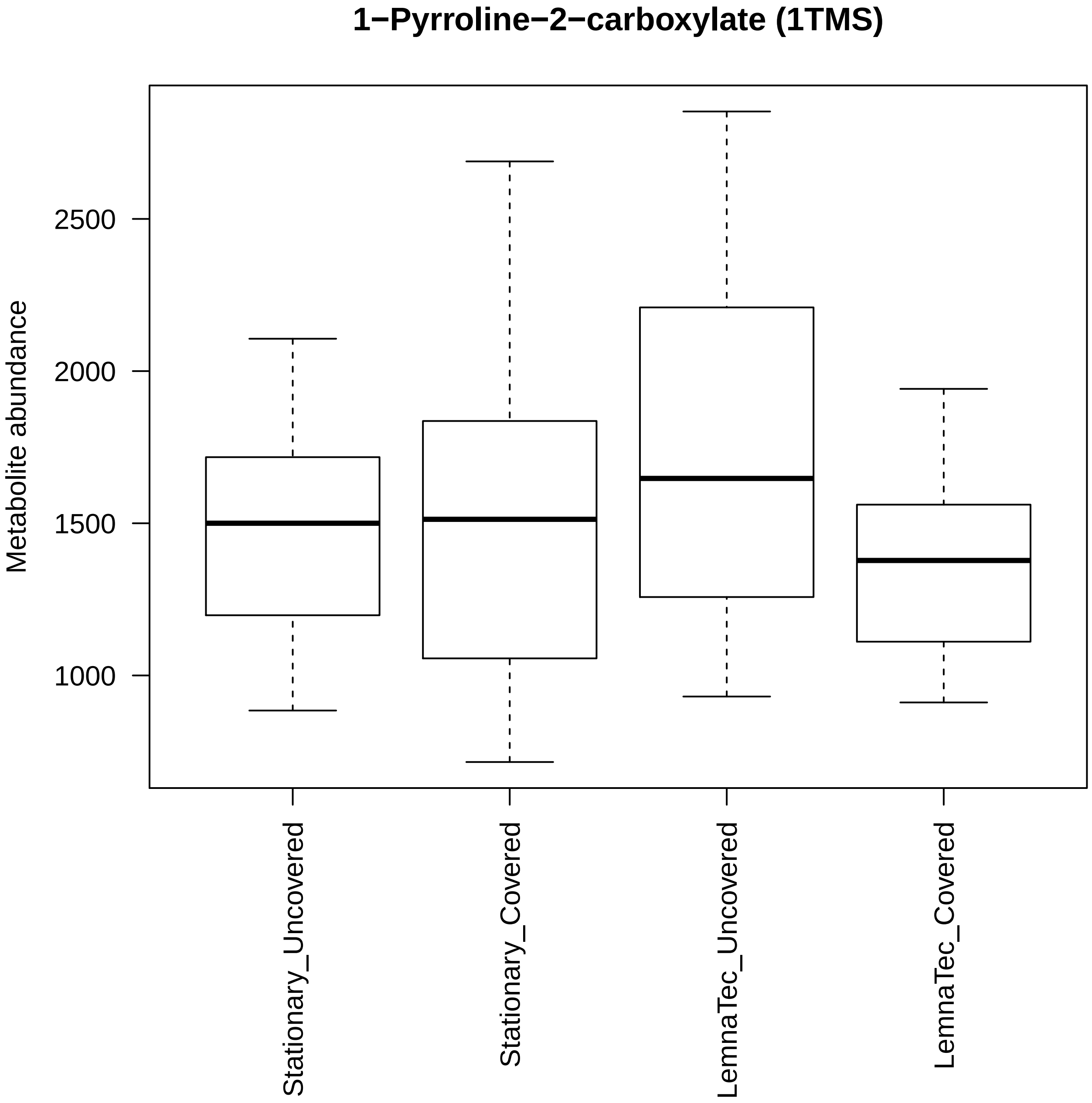
1−Pyrroline−2−carboxylate (1TMS)
Metabolite abundance
2500
2000
1500
1000
Stationary_Uncovered
Stationary_Covered
LemnaTec_Uncovered
LemnaTec_Covered

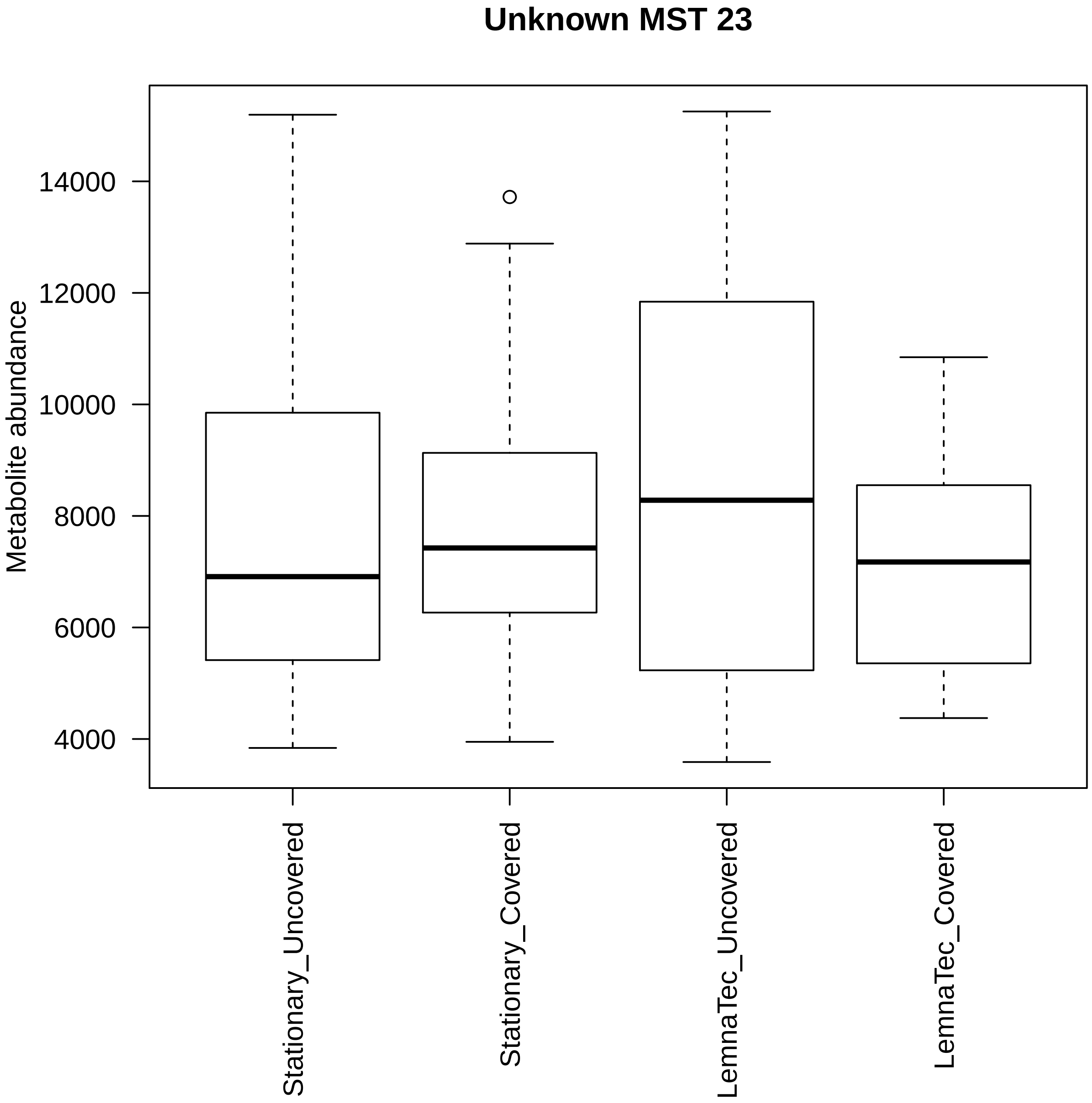
Unknown MST 23
Metabolite abundance
14000
12000
10000
8000
6000
4000
Stationary_Uncovered
Stationary_Covered
LemnaTec_Uncovered
LemnaTec_Covered

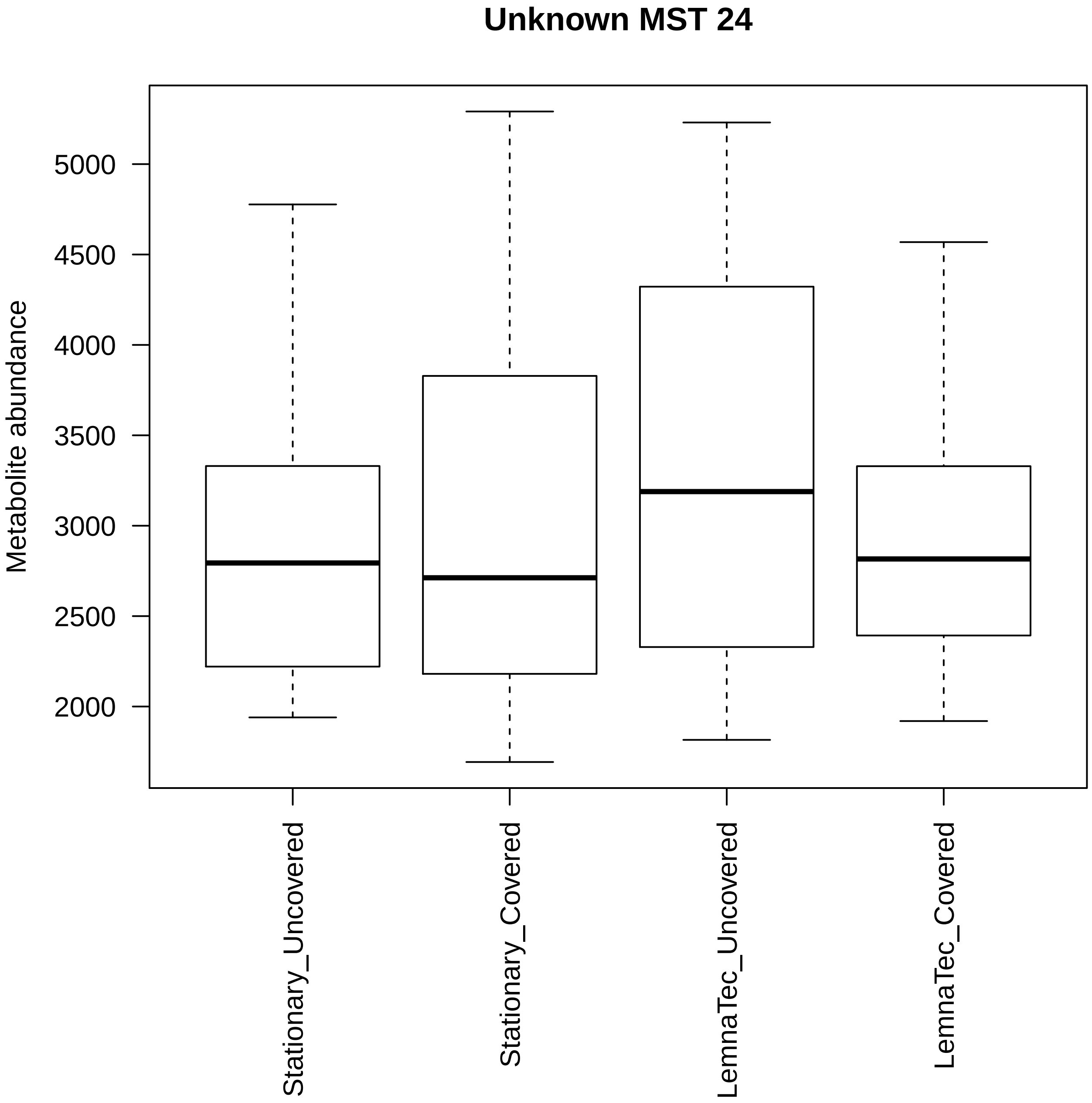
Unknown MST 24
Metabolite abundance
5000
4500
4000
3500
3000
2500
2000
Stationary_Uncovered
Stationary_Covered
LemnaTec_Uncovered
LemnaTec_Covered

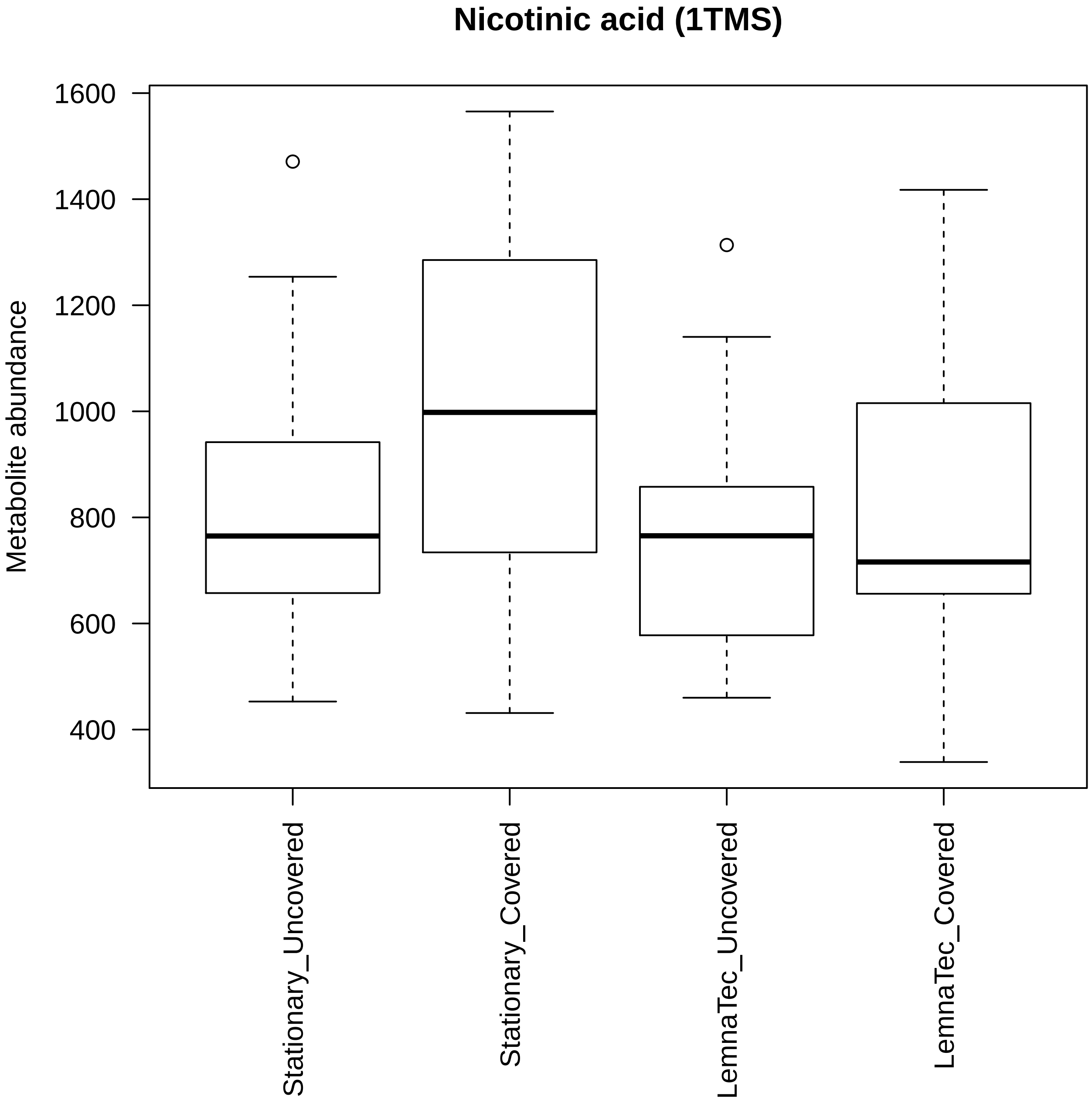

Nicotinic acid (1TMS)
Metabolite abundance
1600
1400
1200
1000
800
600
400
Stationary_Uncovered
Stationary_Covered
LemnaTec_Uncovered
LemnaTec_Covered

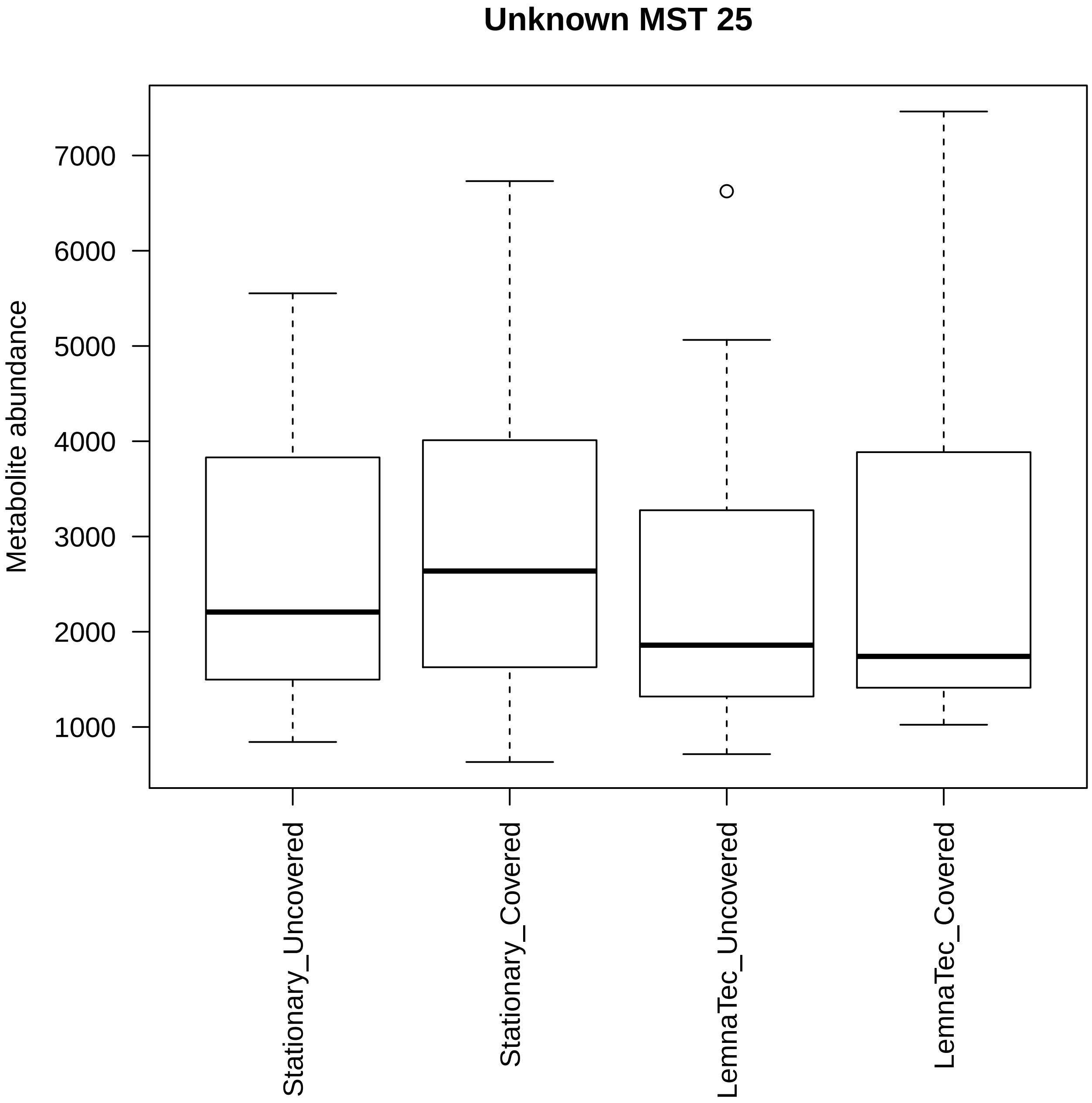

Unknown MST 25
Metabolite abundance
7000
6000
5000
4000
3000
2000
1000
Stationary_Uncovered
Stationary_Covered
LemnaTec_Uncovered
LemnaTec_Covered

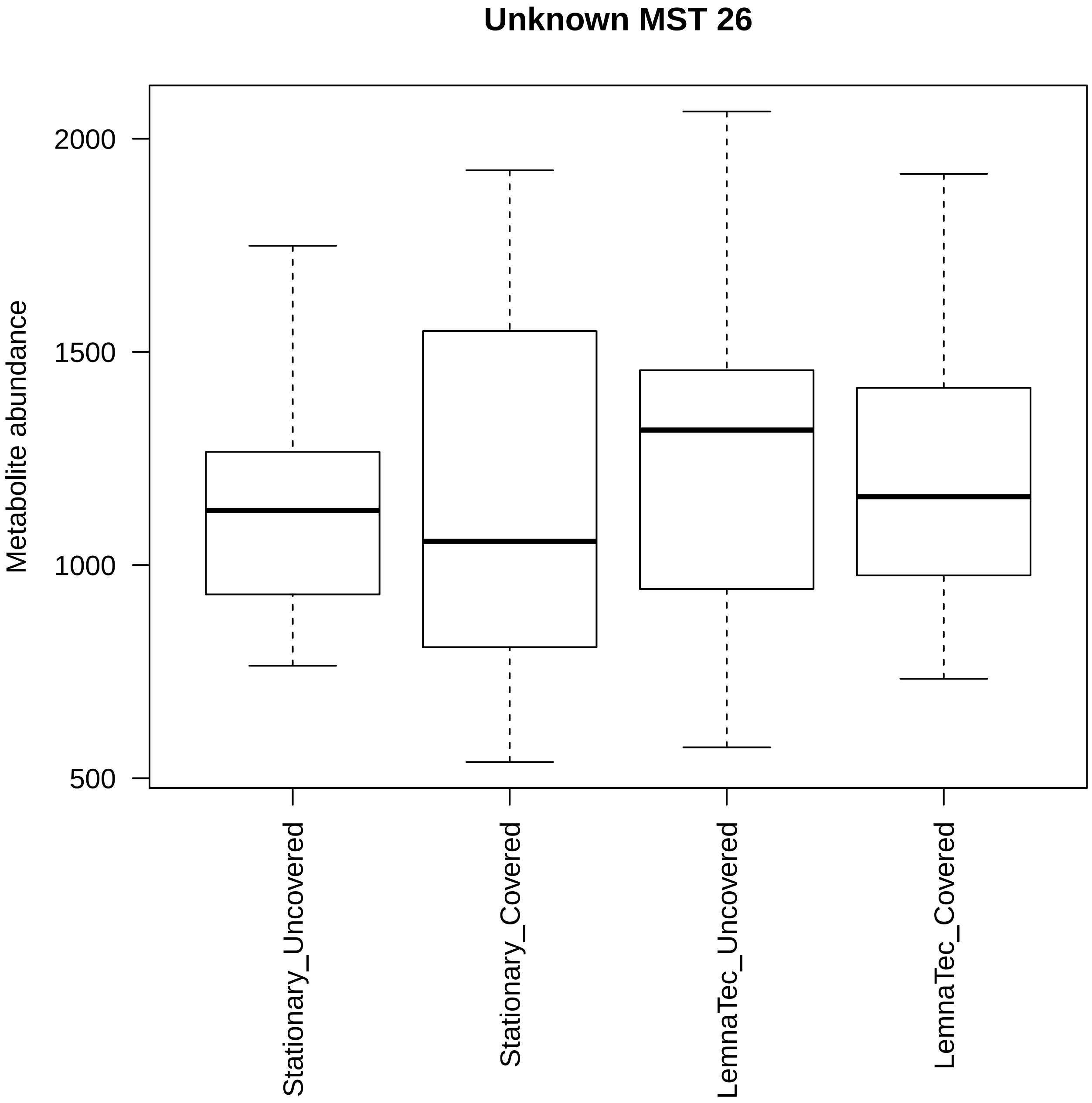

Unknown MST 26
Metabolite abundance
2000
1500
1000
500
Stationary_Uncovered
Stationary_Covered
LemnaTec_Uncovered
LemnaTec_Covered

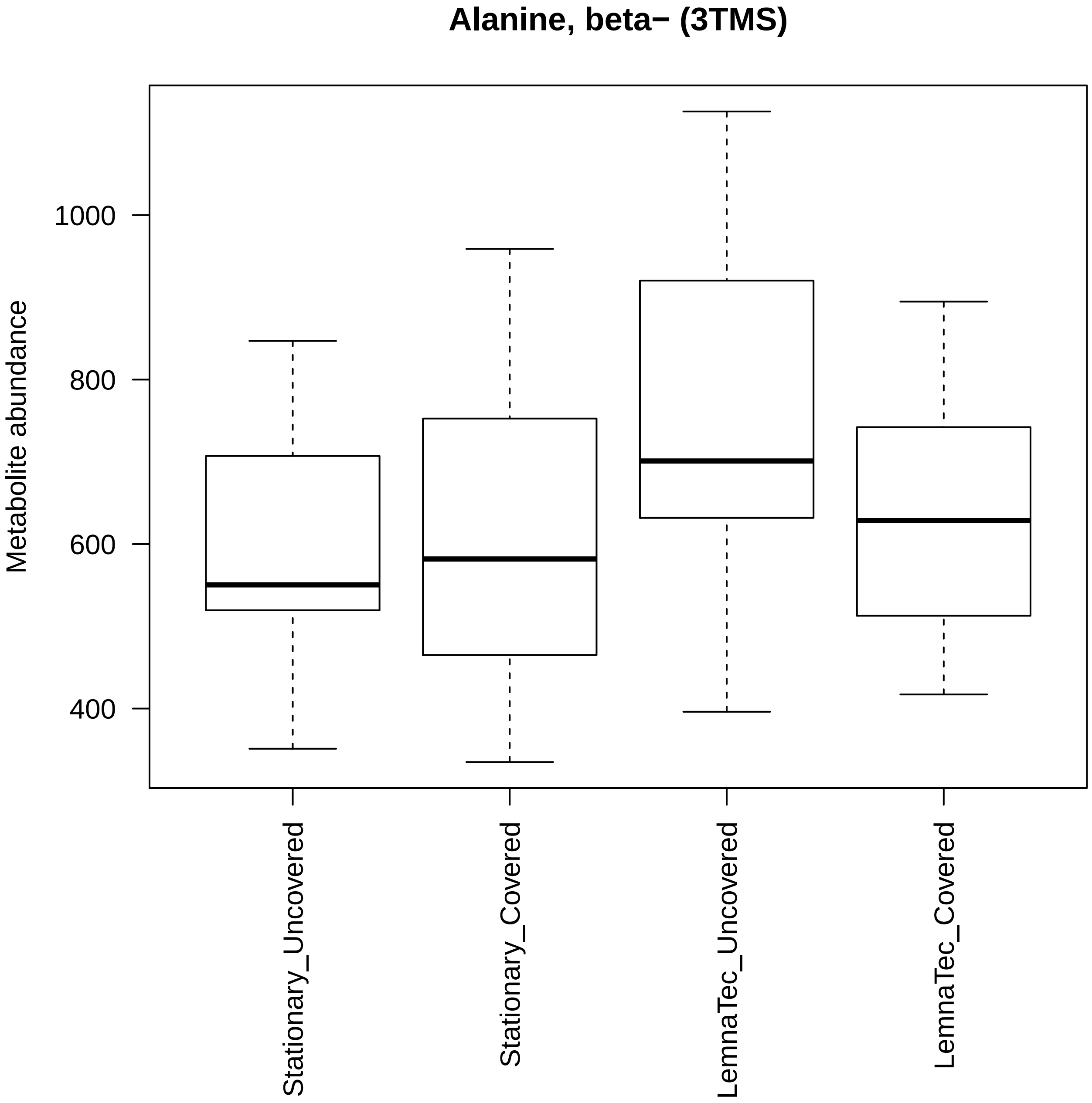

Alanine, beta− (3TMS)
Metabolite abundance
1000
800
600
400
Stationary_Uncovered
Stationary_Covered
LemnaTec_Uncovered
LemnaTec_Covered

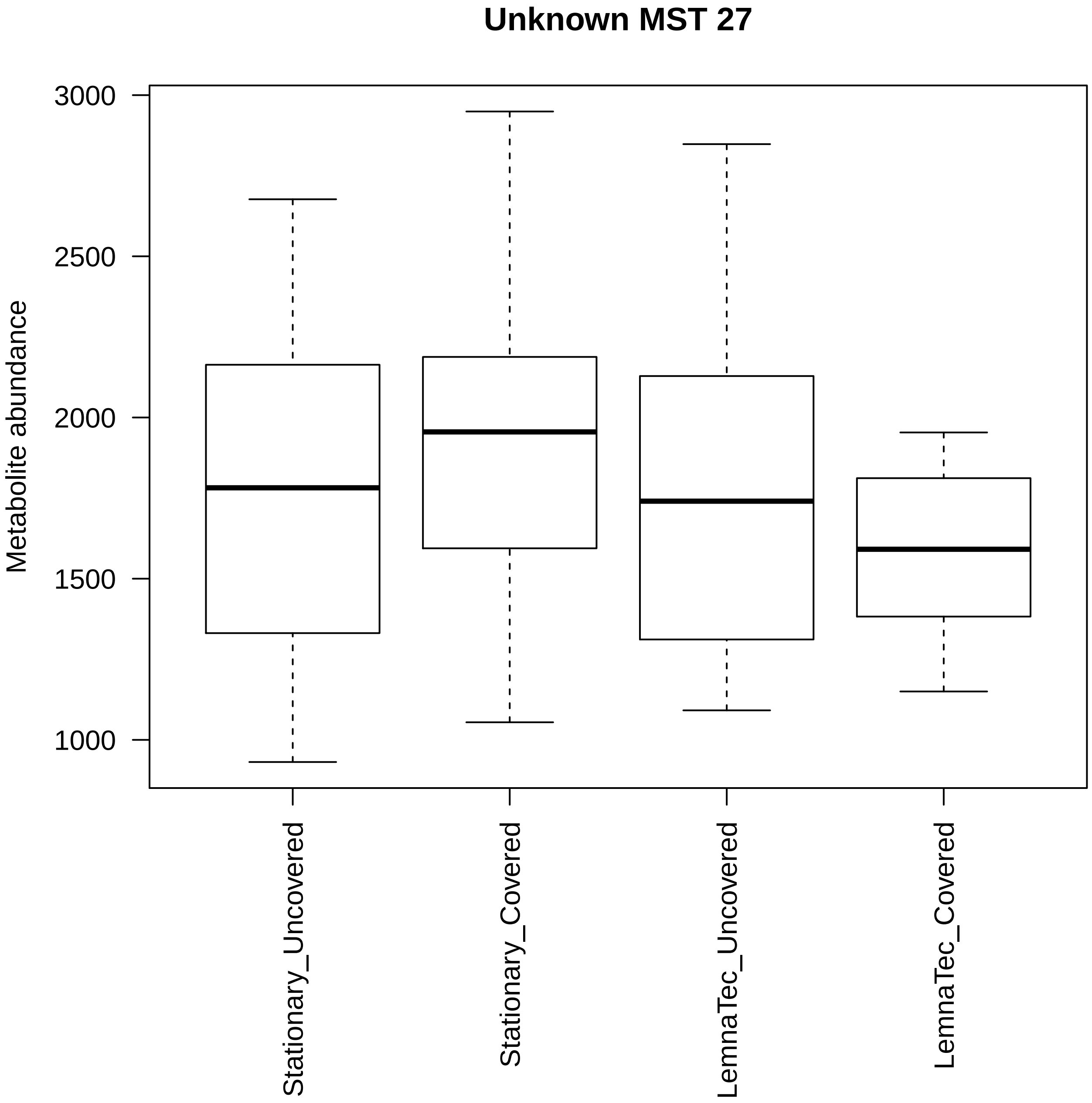
Unknown MST 27
Metabolite abundance
3000
2500
2000
1500
1000
Stationary_Uncovered
Stationary_Covered
LemnaTec_Uncovered
LemnaTec_Covered

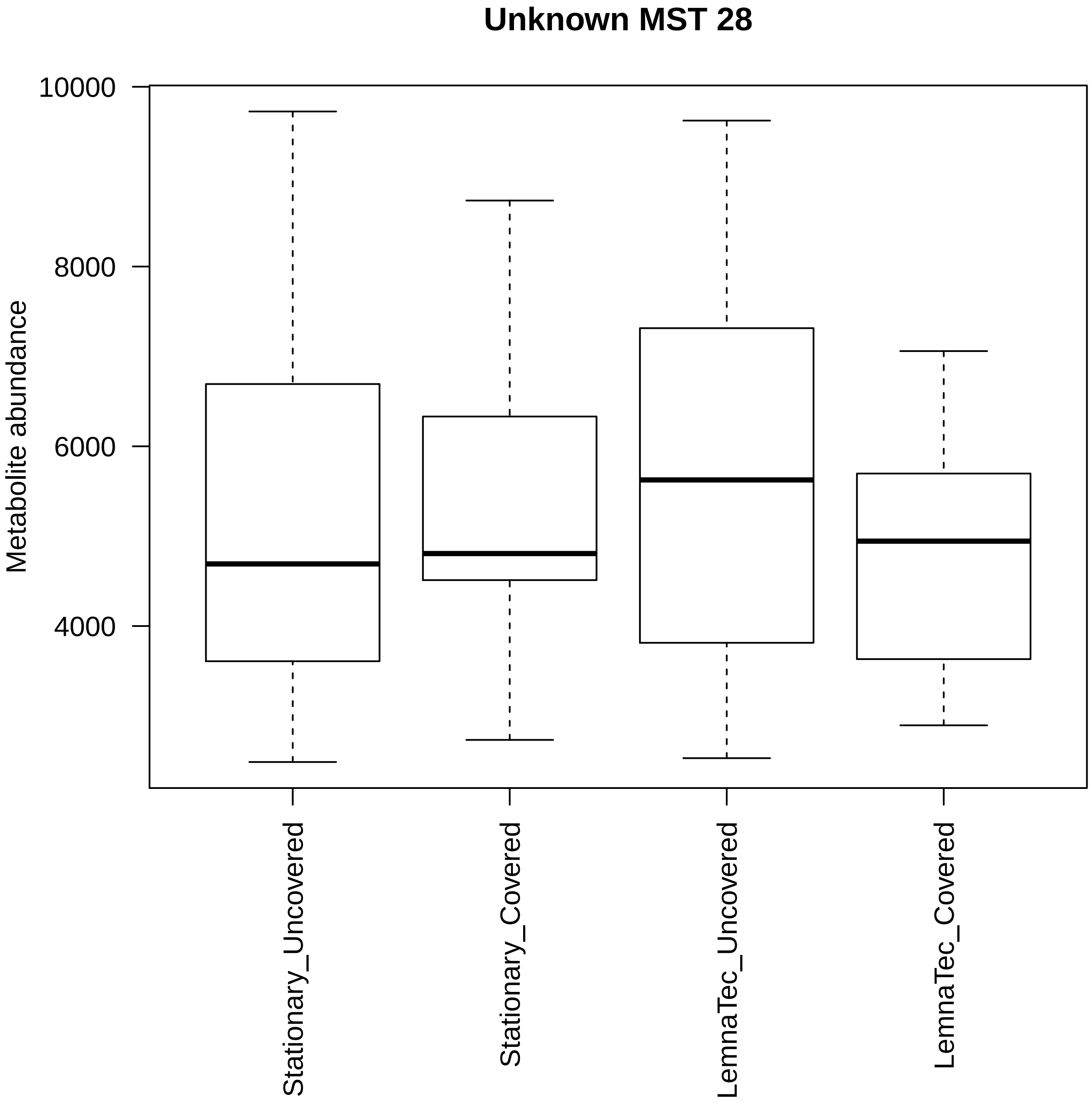
Unknown MST 28
Metabolite abundance
10000
8000
6000
4000
Stationary_Uncovered
Stationary_Covered
LemnaTec_Uncovered
LemnaTec_Covered

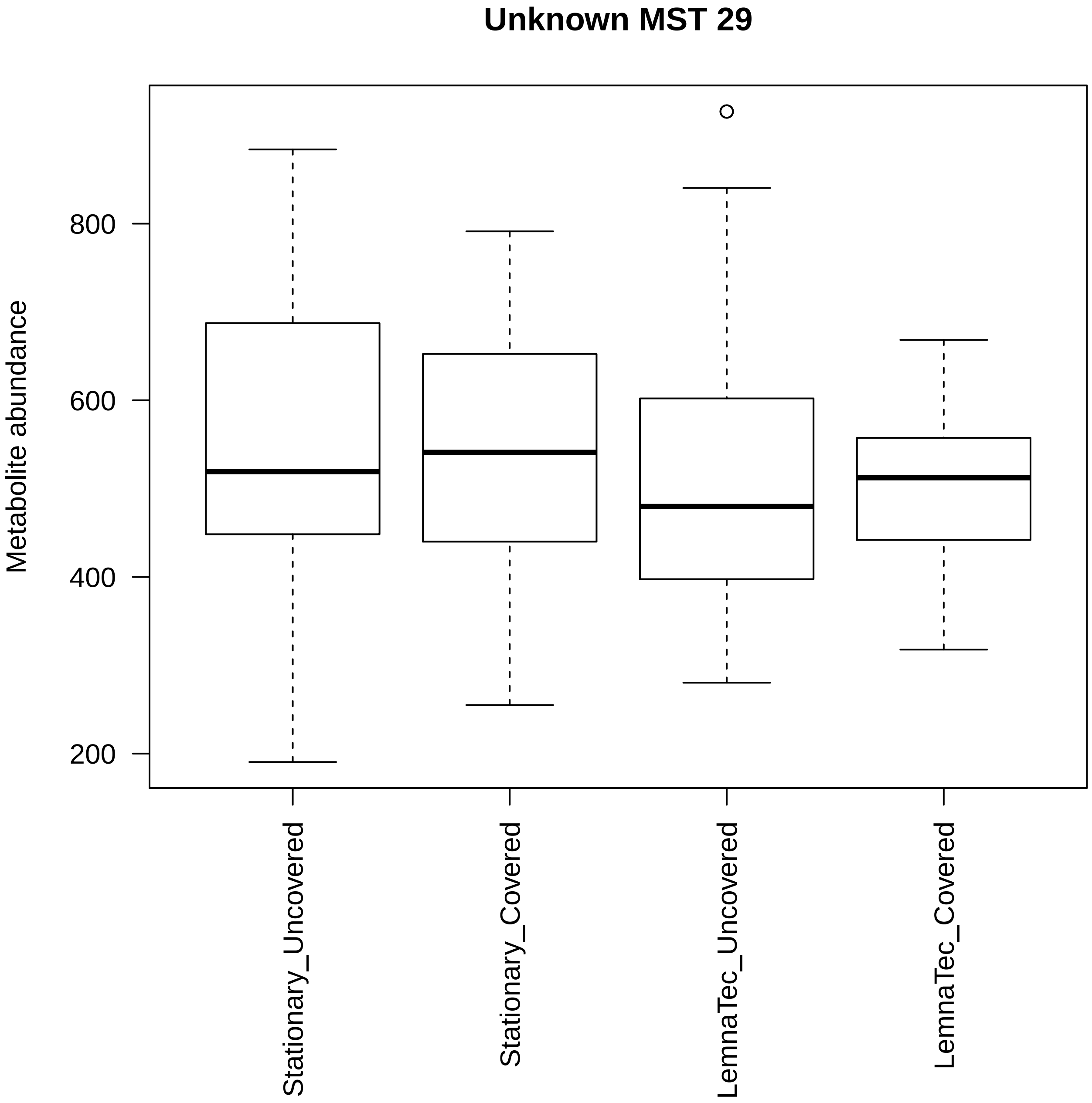

Unknown MST 29
Metabolite abundance
200
400
600
800
Stationary_Uncovered
Stationary_Covered
LemnaTec_Uncovered
LemnaTec_Covered

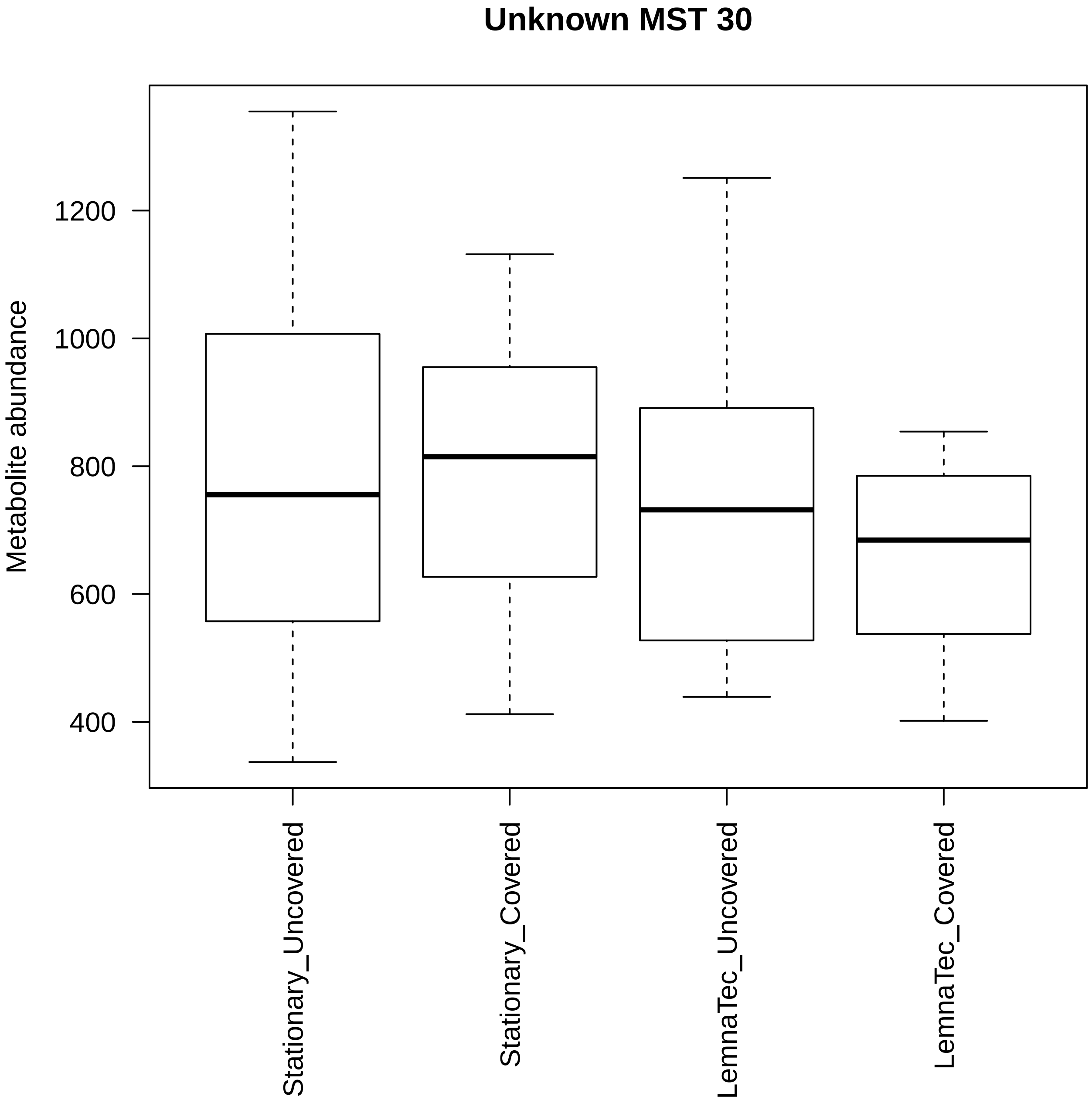

Unknown MST 30
Metabolite abundance
400
600
800
1000
1200
Stationary_Uncovered
Stationary_Covered
LemnaTec_Uncovered
LemnaTec_Covered

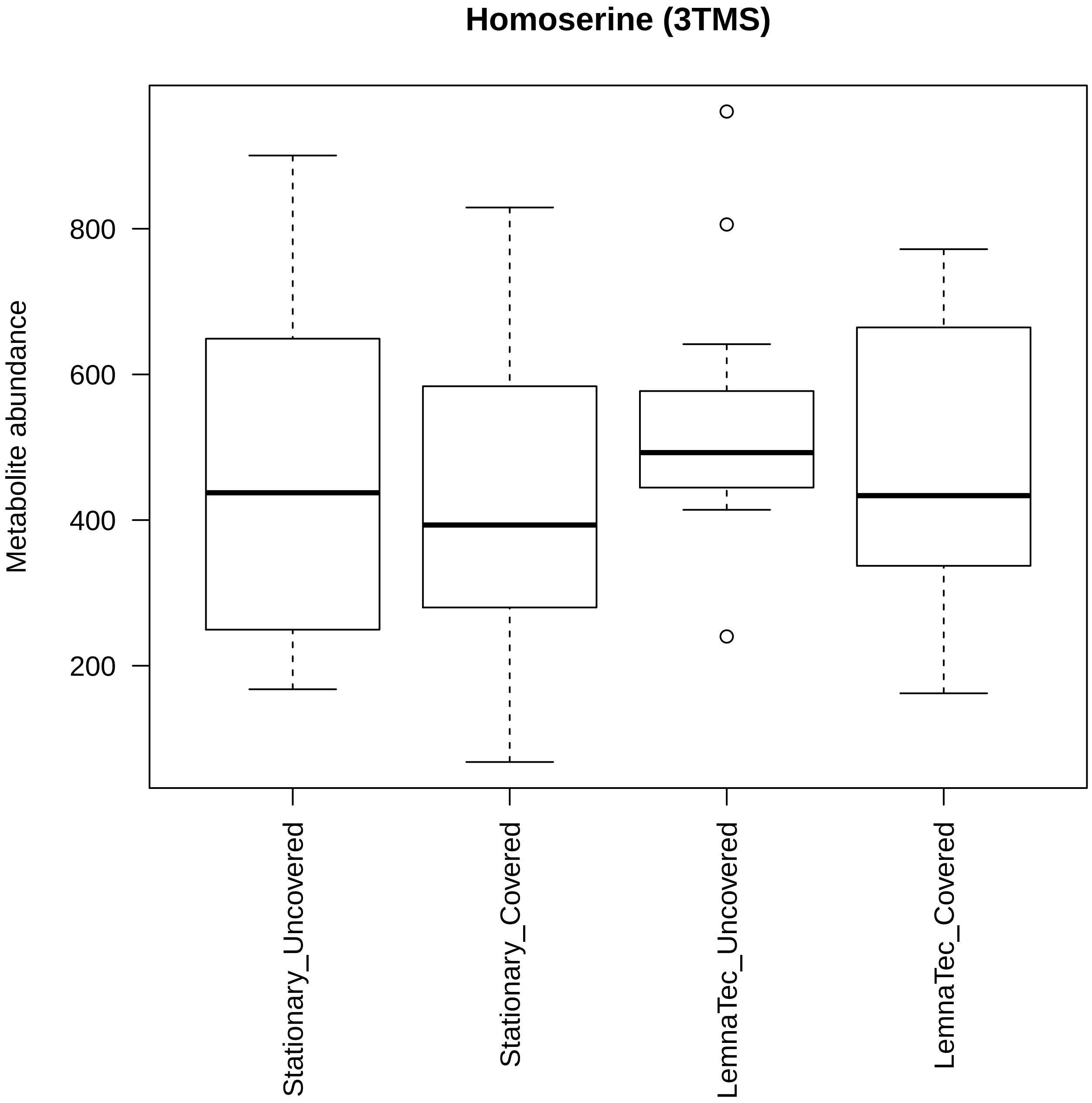

Homoserine (3TMS)
Metabolite abundance
800
600
400
200
Stationary_Uncovered
Stationary_Covered
LemnaTec_Uncovered
LemnaTec_Covered

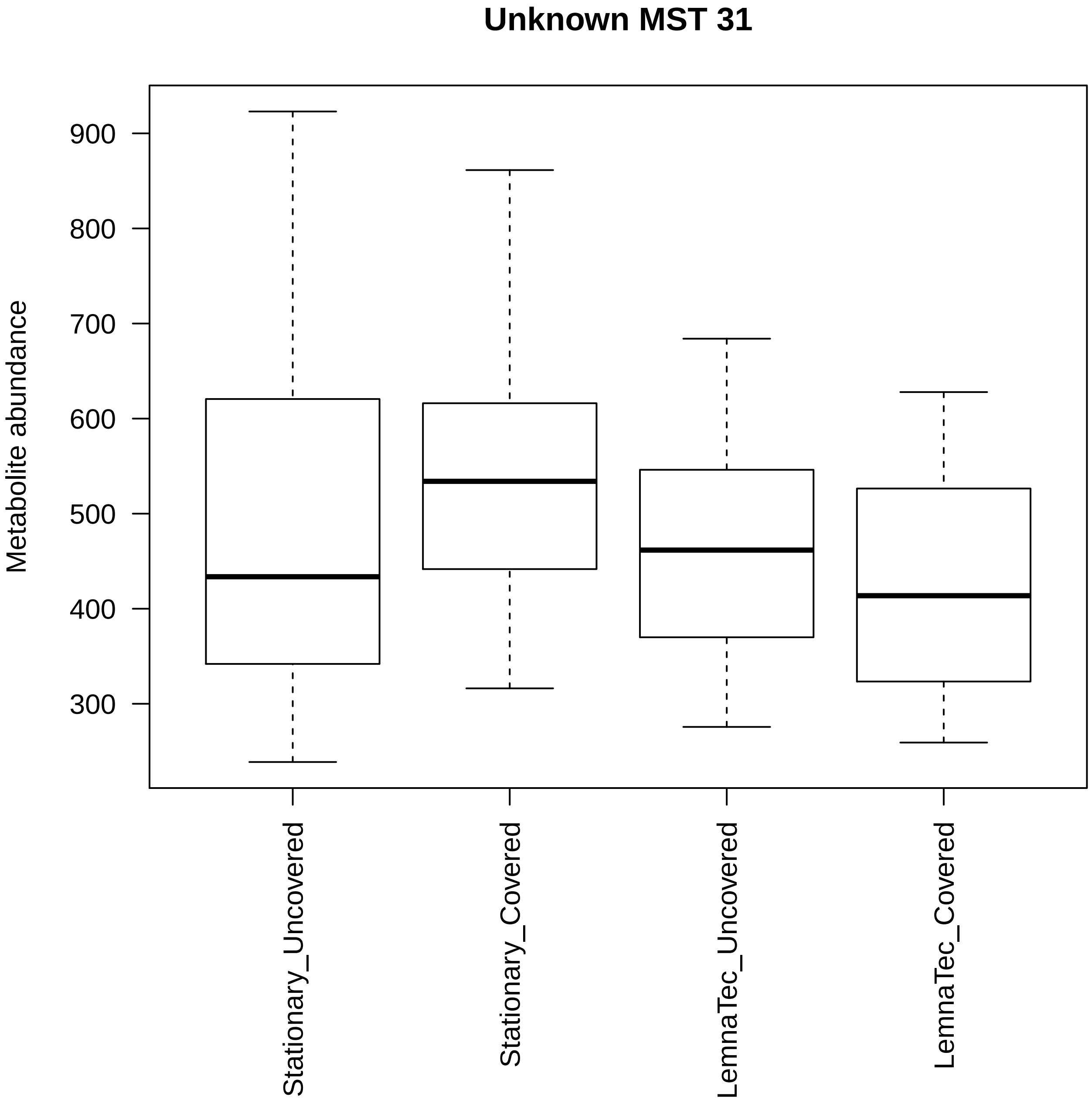
Unknown MST 31
Metabolite abundance
900
800
700
600
500
400
300
Stationary_Uncovered
Stationary_Covered
LemnaTec_Uncovered
LemnaTec_Covered

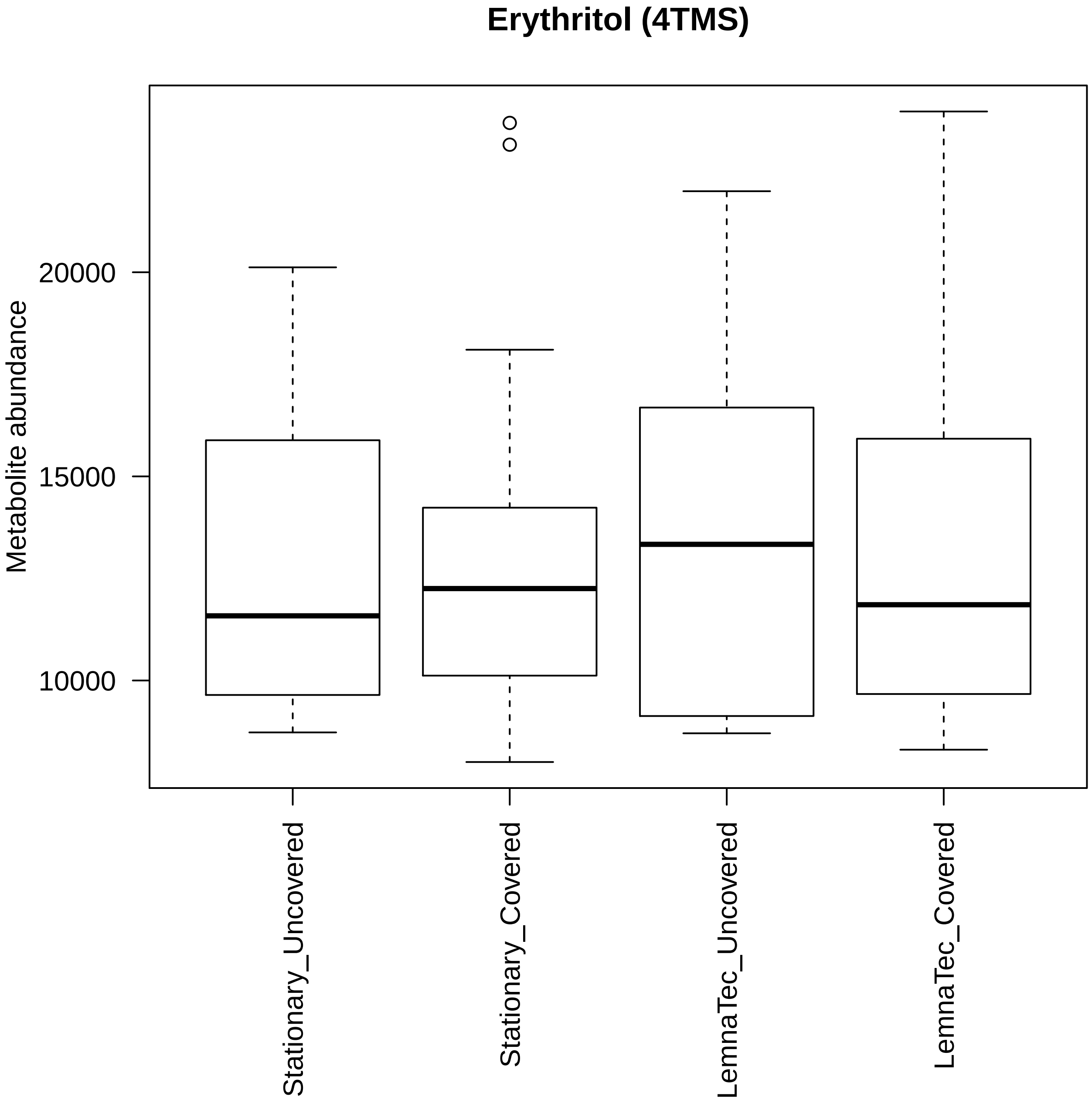
Erythritol (4TMS)
Metabolite abundance
20000
15000
10000
Stationary_Uncovered
Stationary_Covered
LemnaTec_Uncovered
LemnaTec_Covered

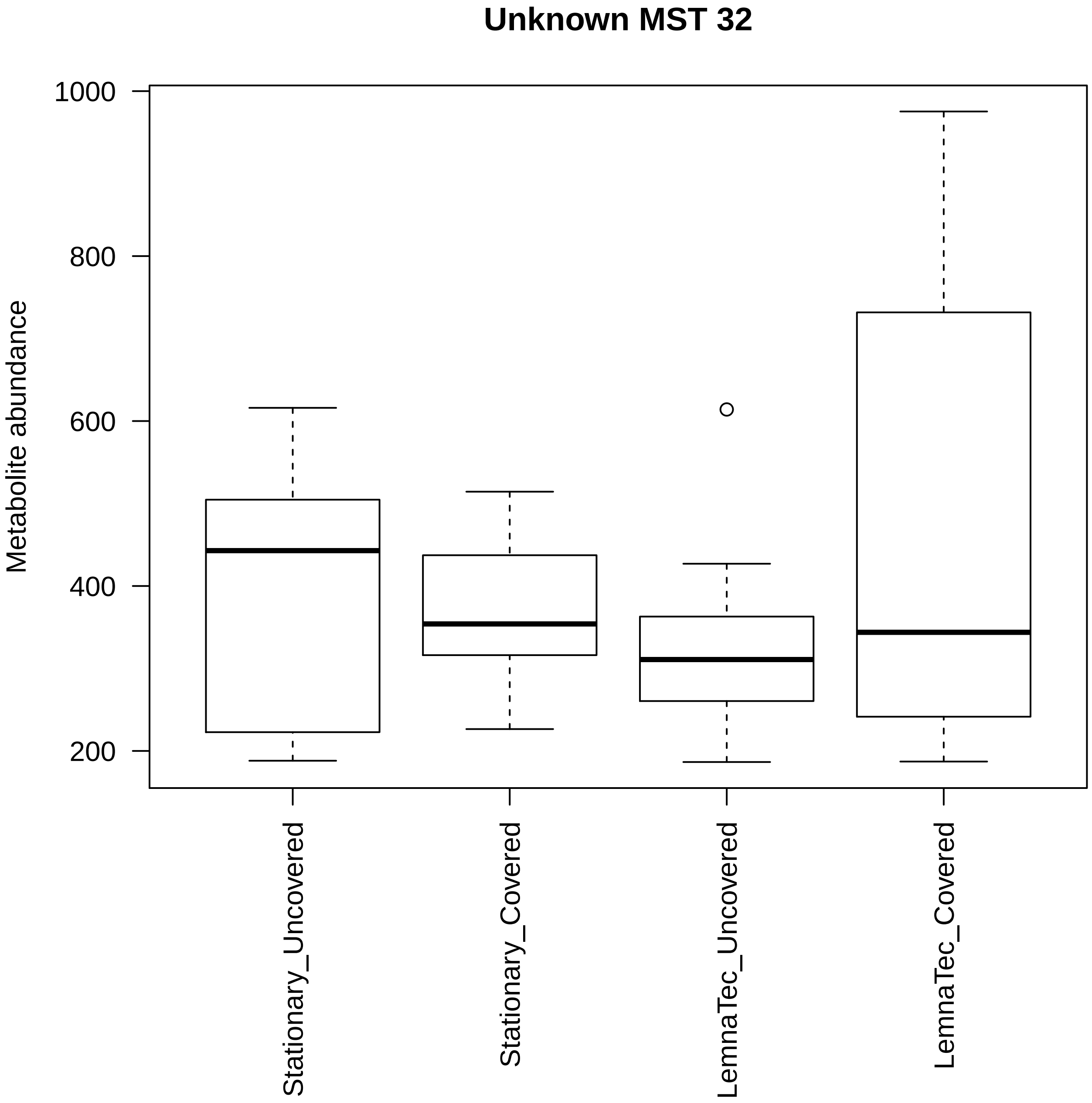

Unknown MST 32
Metabolite abundance
1000
800
600
400
200
Stationary_Uncovered
Stationary_Covered
LemnaTec_Uncovered
LemnaTec_Covered

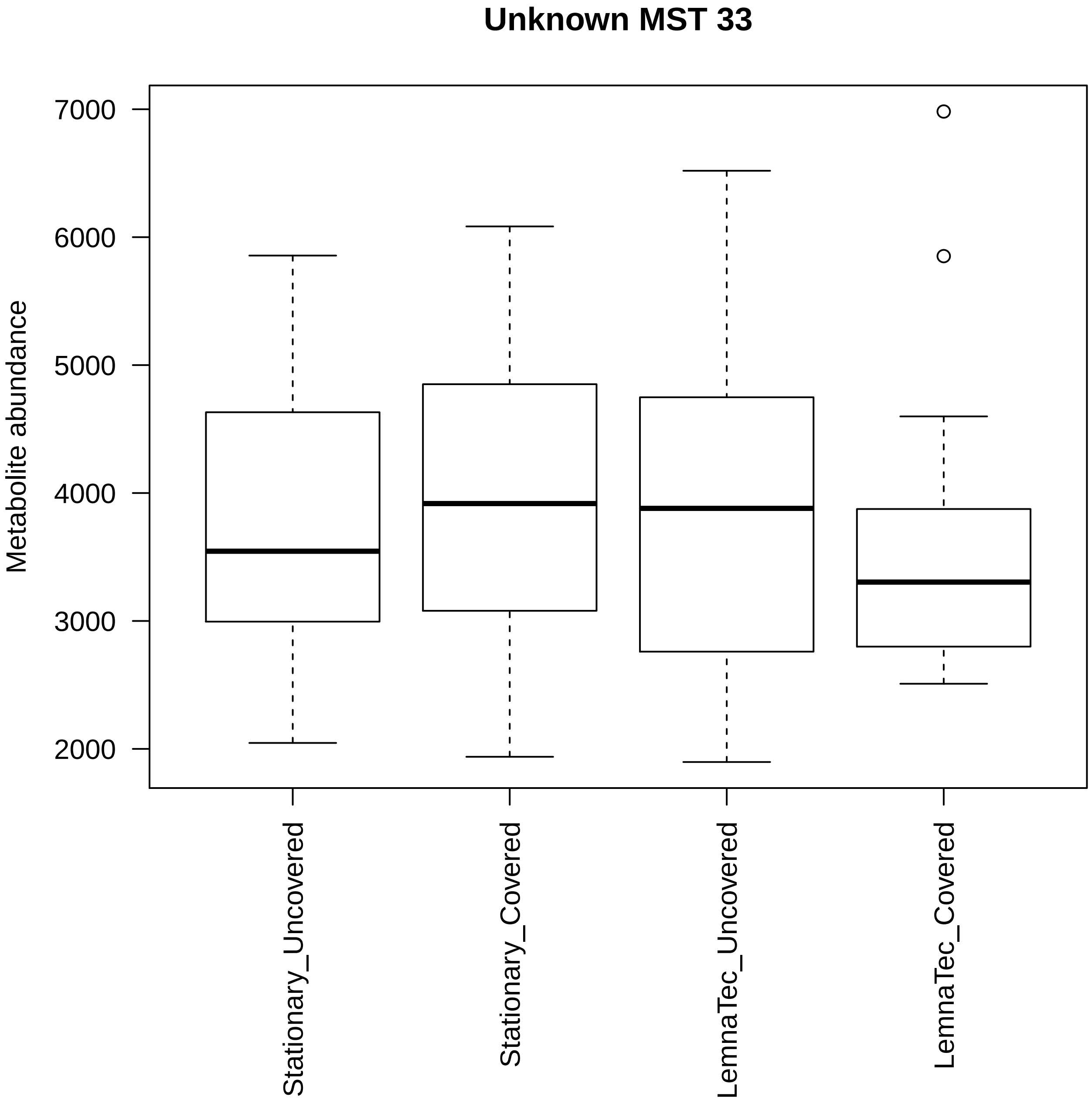

Unknown MST 33
Metabolite abundance
7000
6000
5000
4000
3000
2000
Stationary_Uncovered
Stationary_Covered
LemnaTec_Uncovered
LemnaTec_Covered

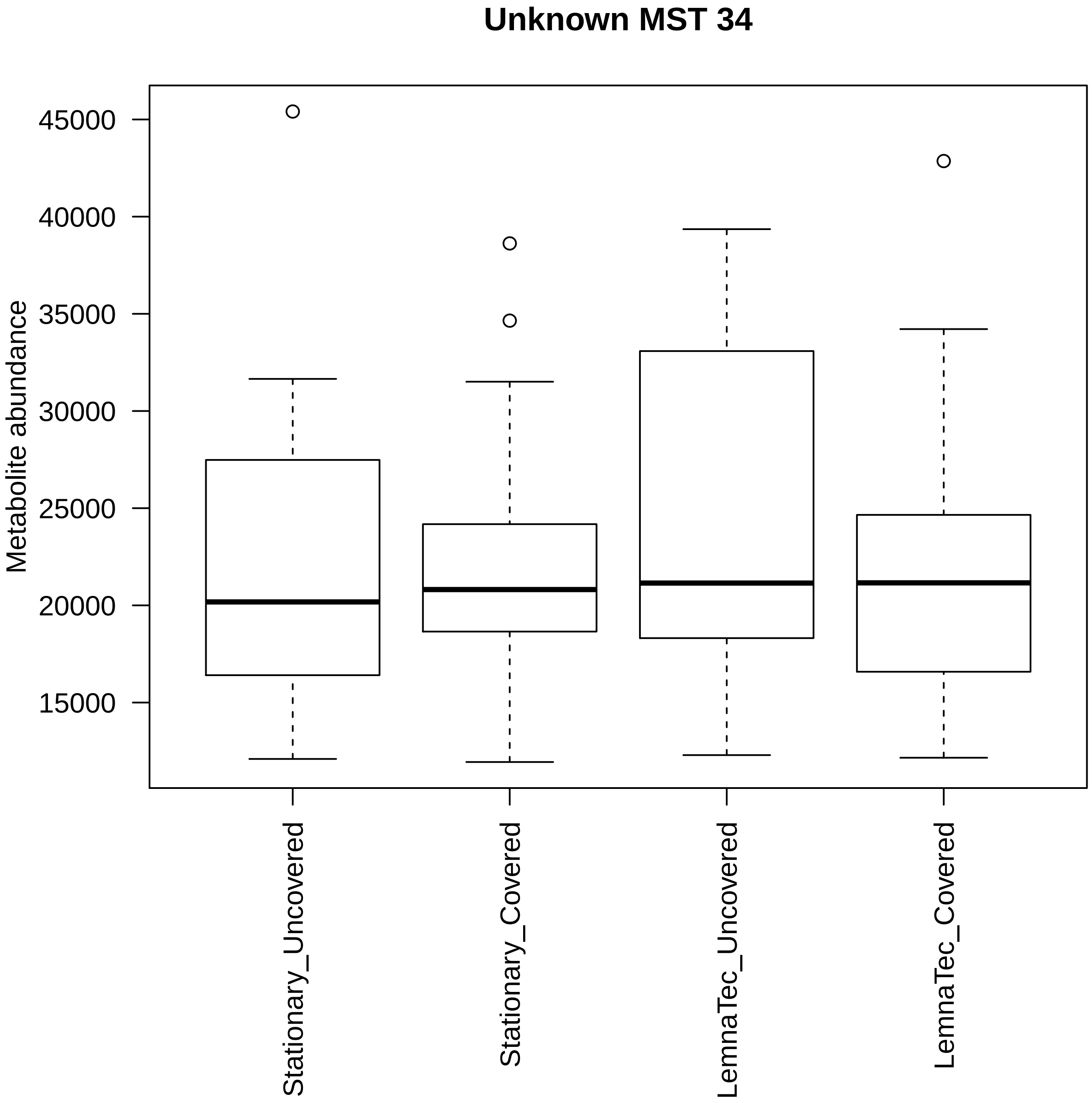
Unknown MST 34
Metabolite abundance
45000
40000
35000
30000
25000
20000
15000
Stationary_Uncovered
Stationary_Covered
LemnaTec_Uncovered
LemnaTec_Covered

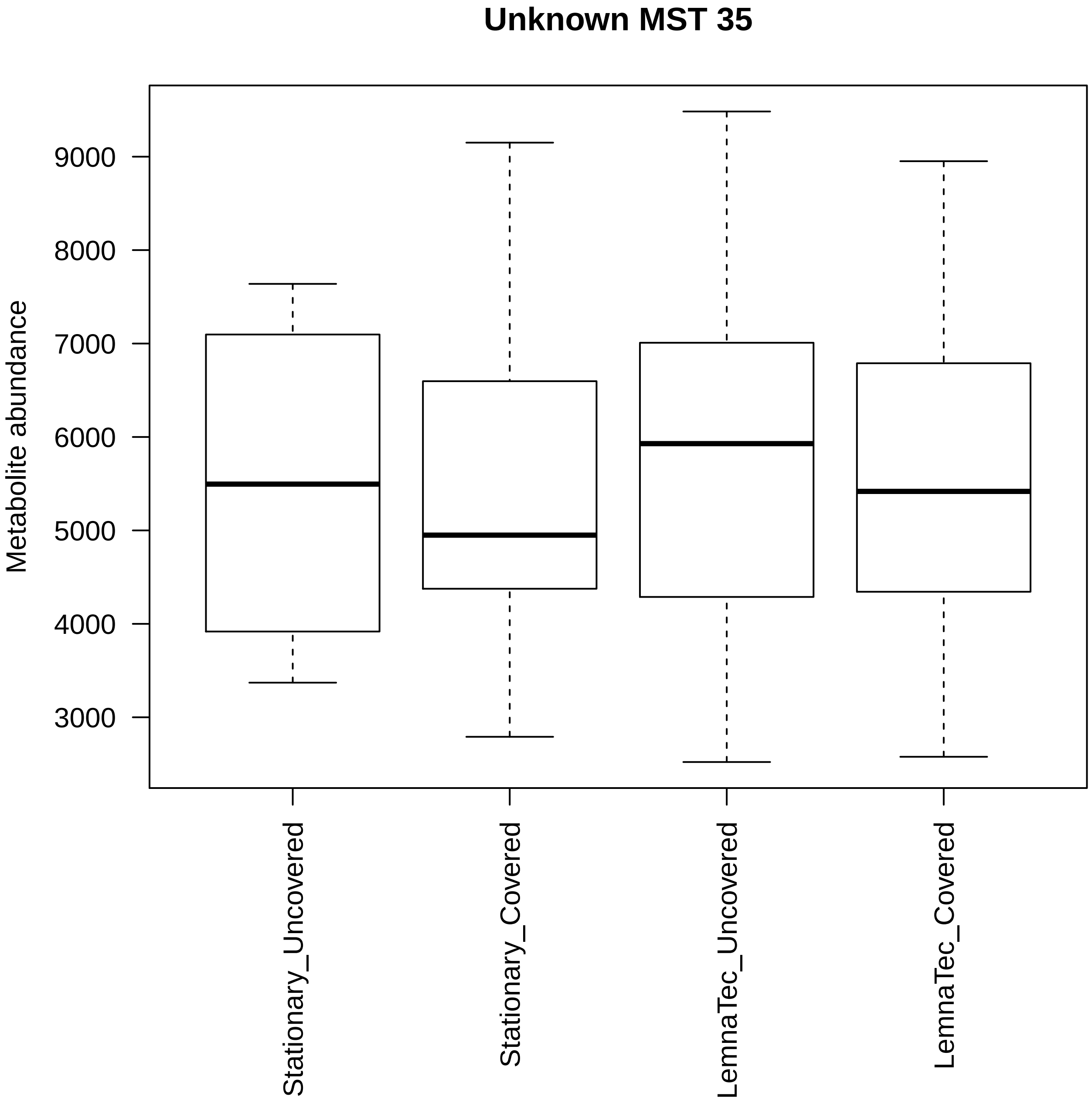

Unknown MST 35
Metabolite abundance
9000
8000
7000
6000
5000
4000
3000
Stationary_Uncovered
Stationary_Covered
LemnaTec_Uncovered
LemnaTec_Covered

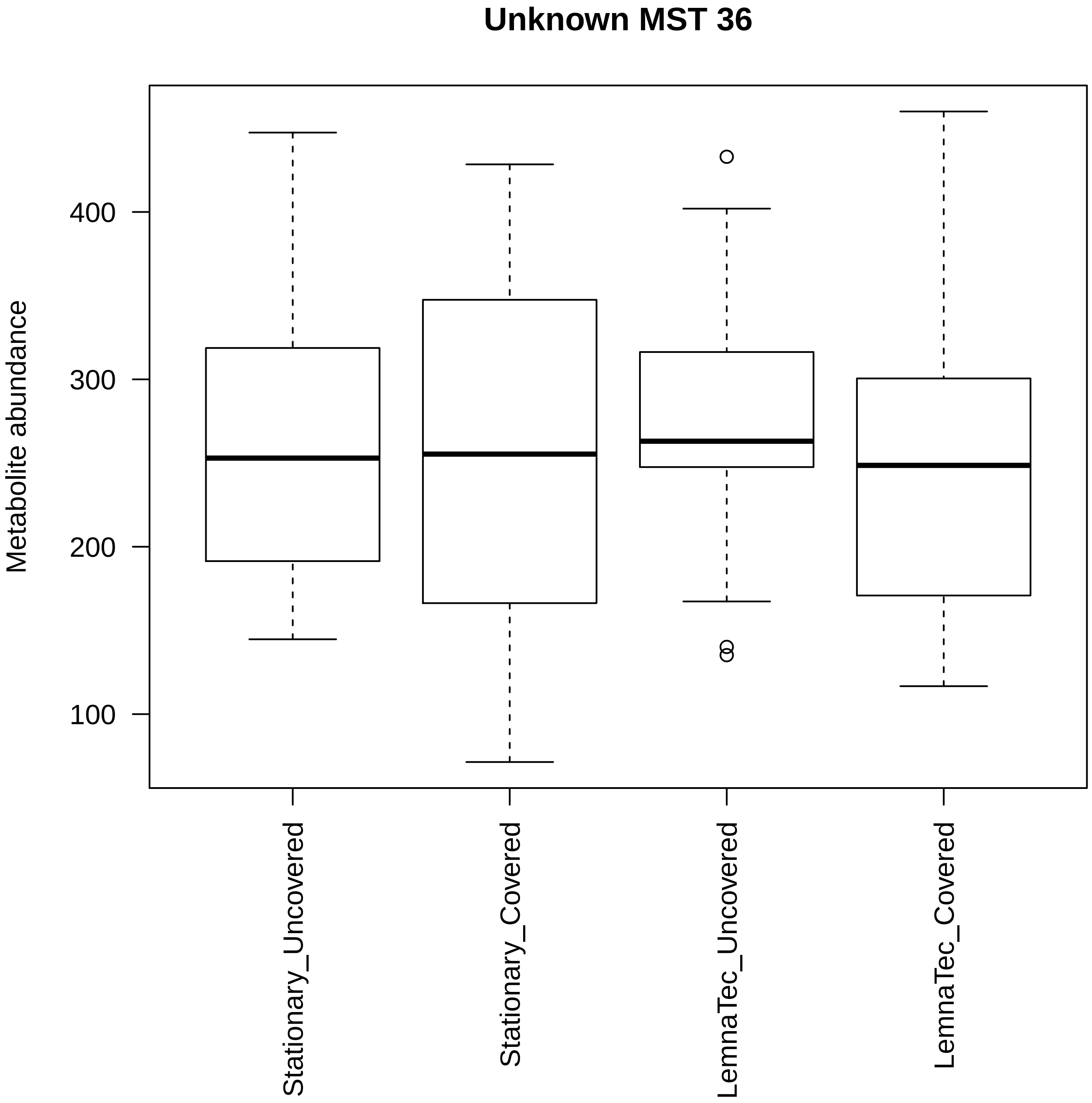
Unknown MST 36
Metabolite abundance
100
200
300
400
Stationary_Uncovered
Stationary_Covered
LemnaTec_Uncovered
LemnaTec_Covered

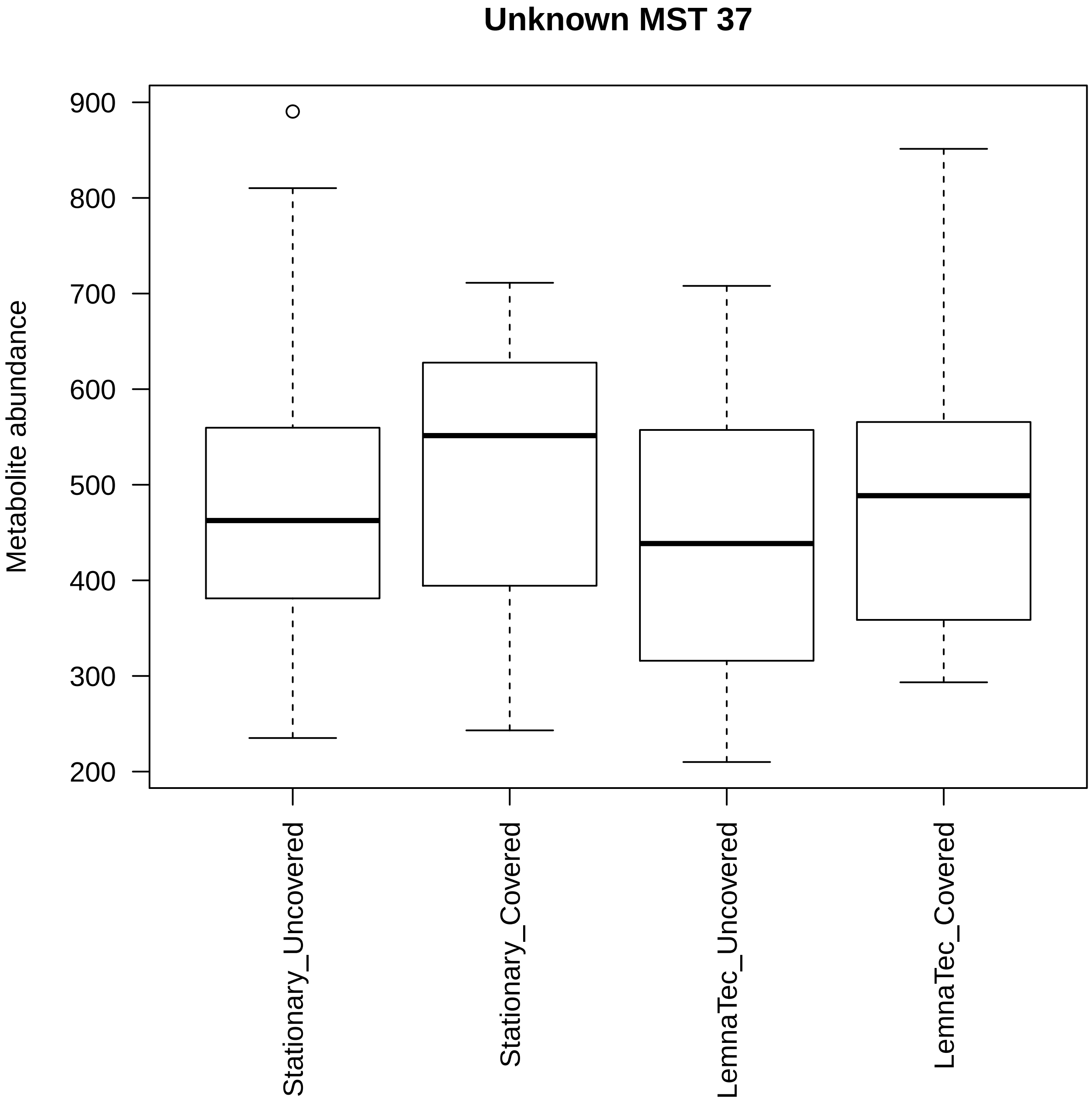
Unknown MST 37
Metabolite abundance
900
800
700
600
500
400
300
200
Stationary_Uncovered
Stationary_Covered
LemnaTec_Uncovered
LemnaTec_Covered

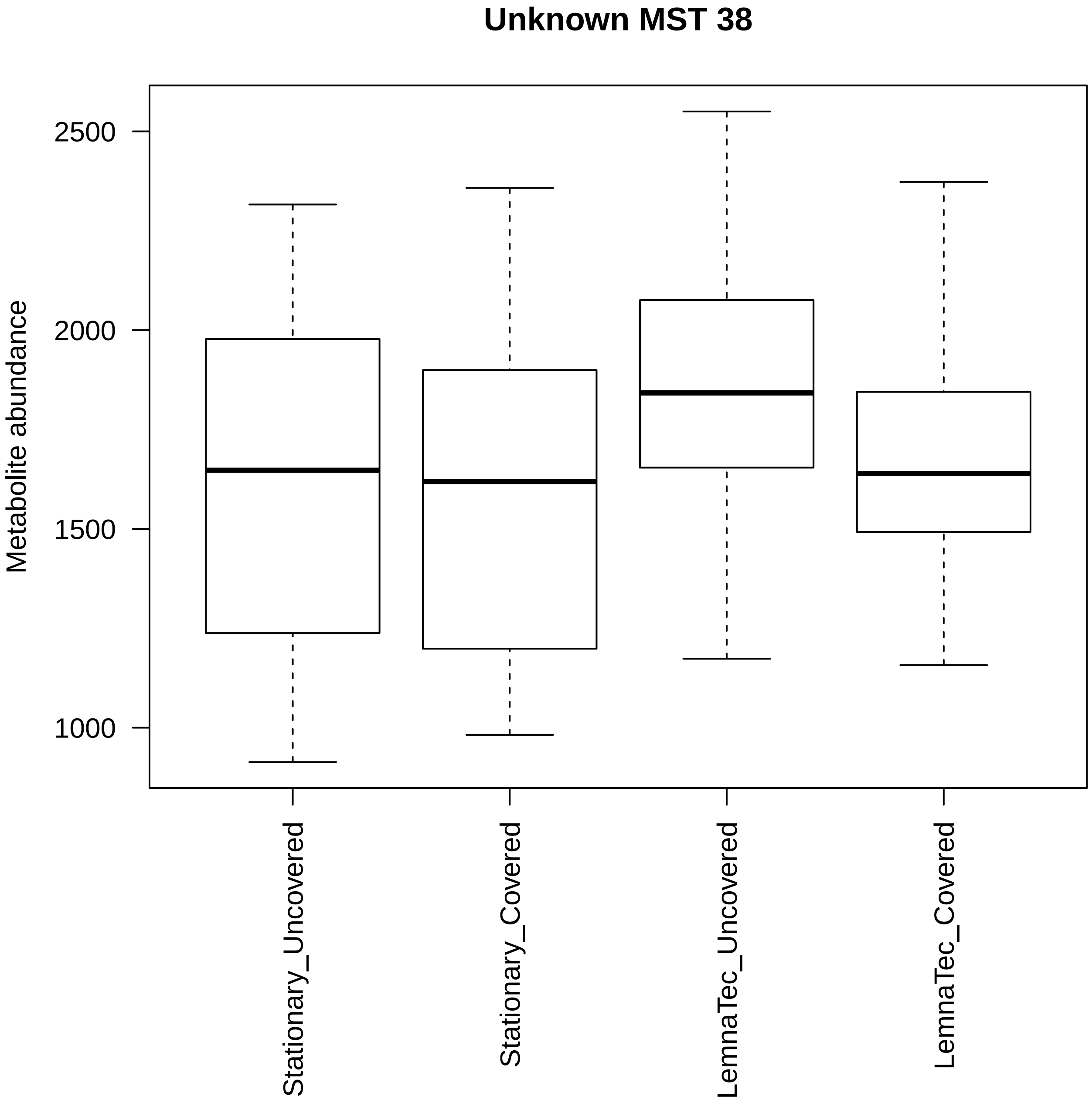

Unknown MST 38
Metabolite abundance
2500
2000
1500
1000
Stationary_Uncovered
Stationary_Covered
LemnaTec_Uncovered
LemnaTec_Covered

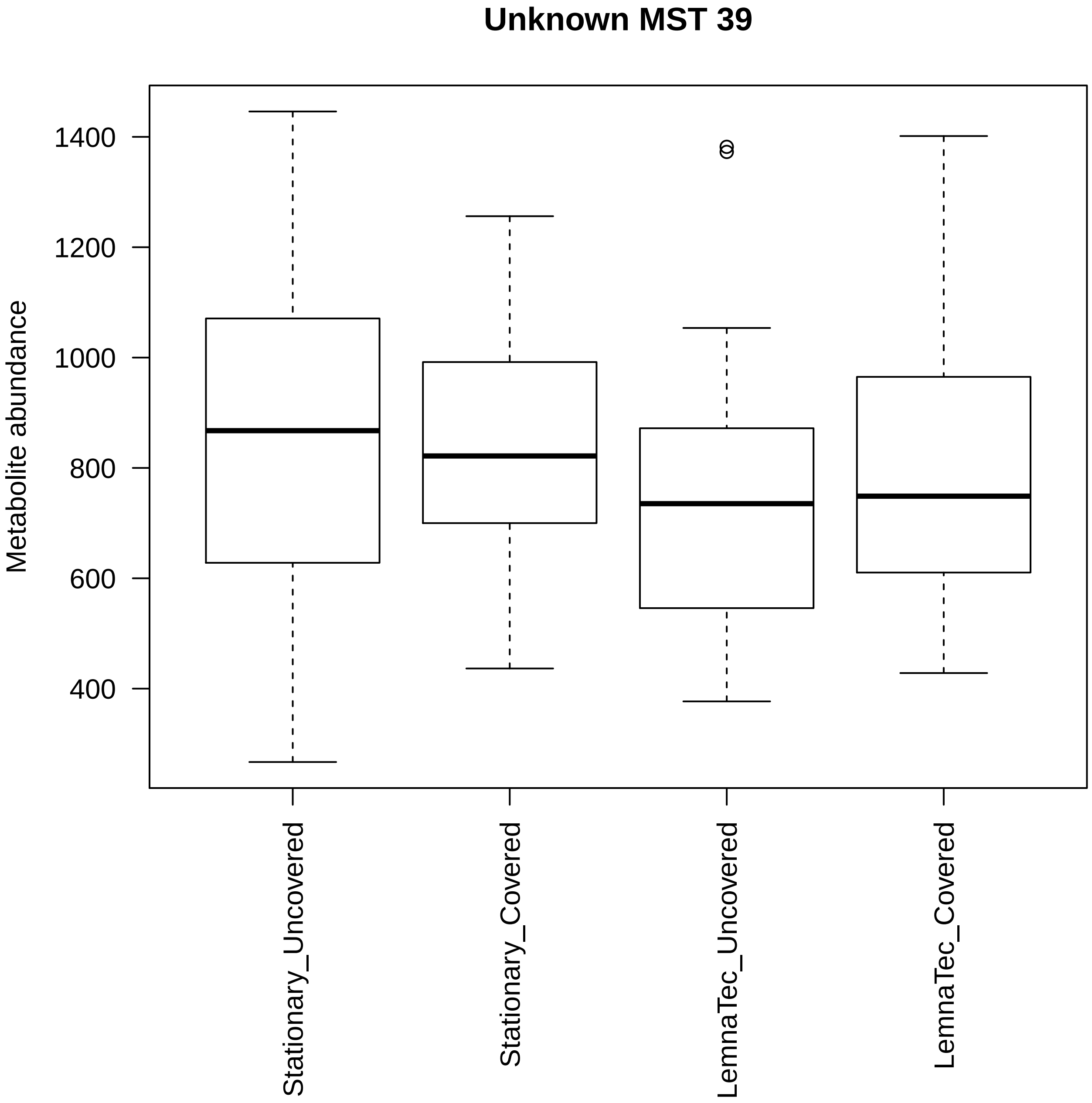
Unknown MST 39
Metabolite abundance
1400
1200
1000
800
600
400
Stationary_Uncovered
Stationary_Covered
LemnaTec_Uncovered
LemnaTec_Covered

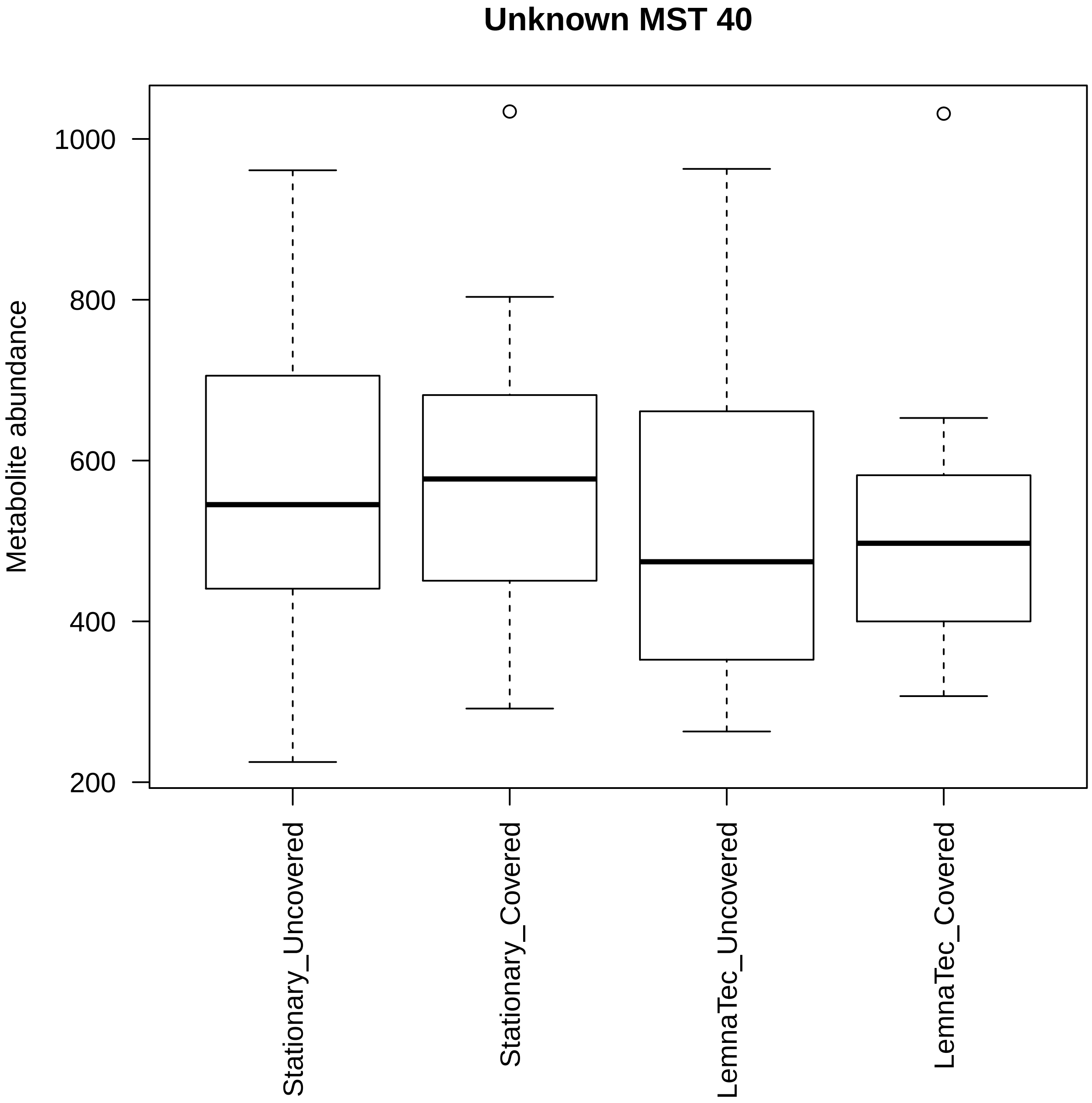
Unknown MST 40
Metabolite abundance
1000
800
600
400
200
Stationary_Uncovered
Stationary_Covered
LemnaTec_Uncovered
LemnaTec_Covered

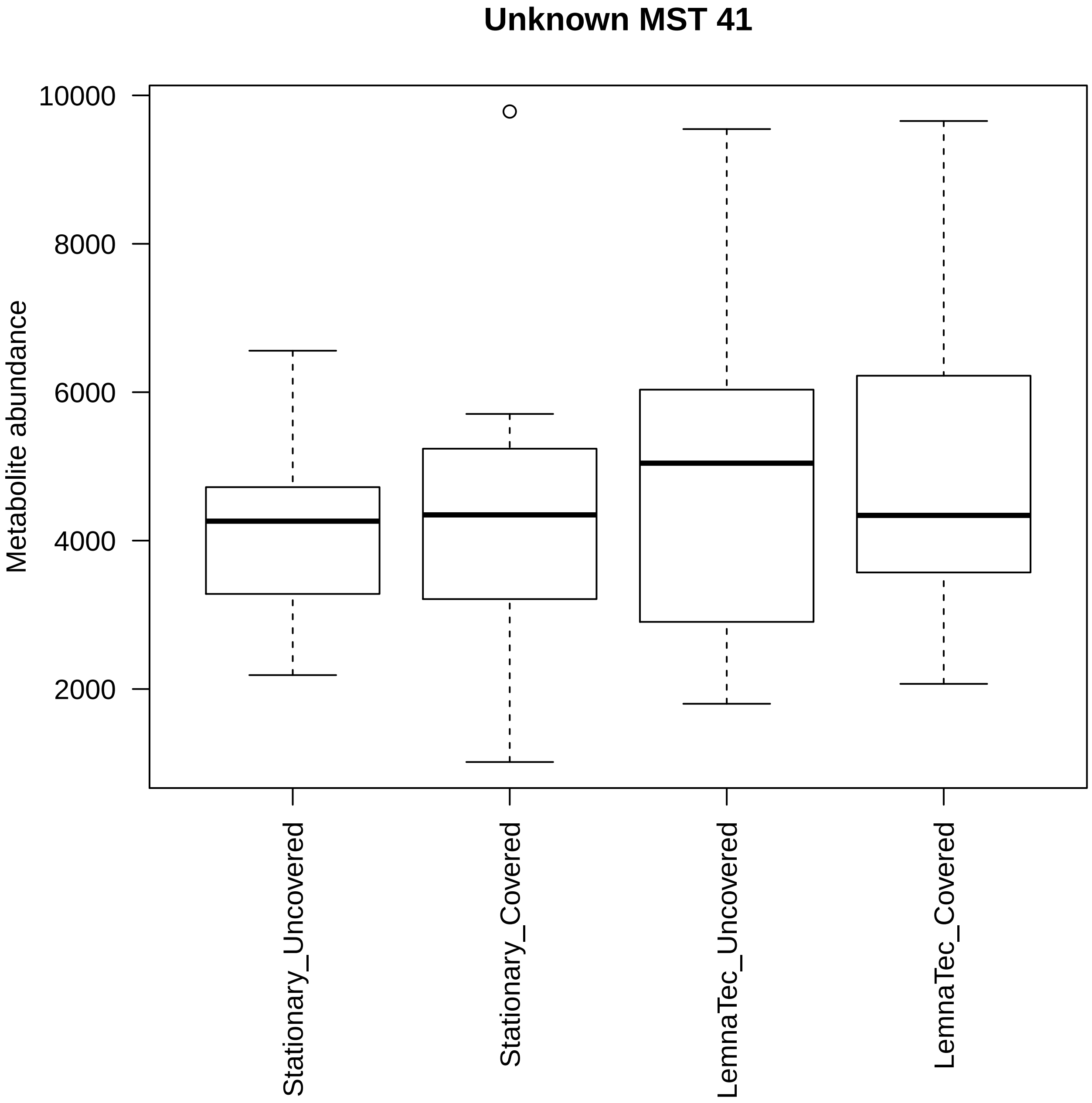

Unknown MST 41
Metabolite abundance
10000
8000
6000
4000
2000
Stationary_Uncovered
Stationary_Covered
LemnaTec_Uncovered
LemnaTec_Covered

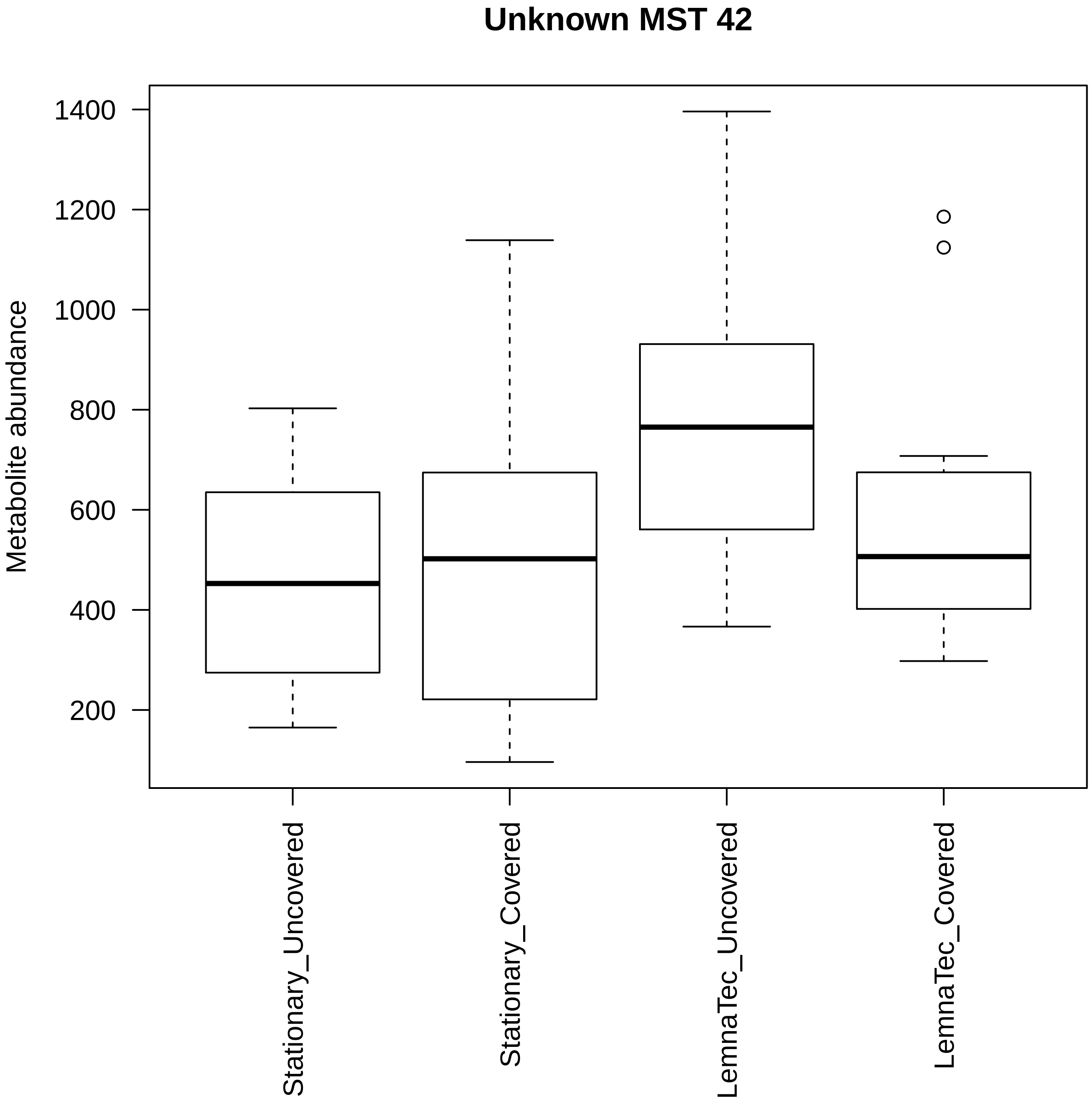
Unknown MST 42
Metabolite abundance
1400
1200
1000
800
600
400
200
Stationary_Uncovered
Stationary_Covered
LemnaTec_Uncovered
LemnaTec_Covered

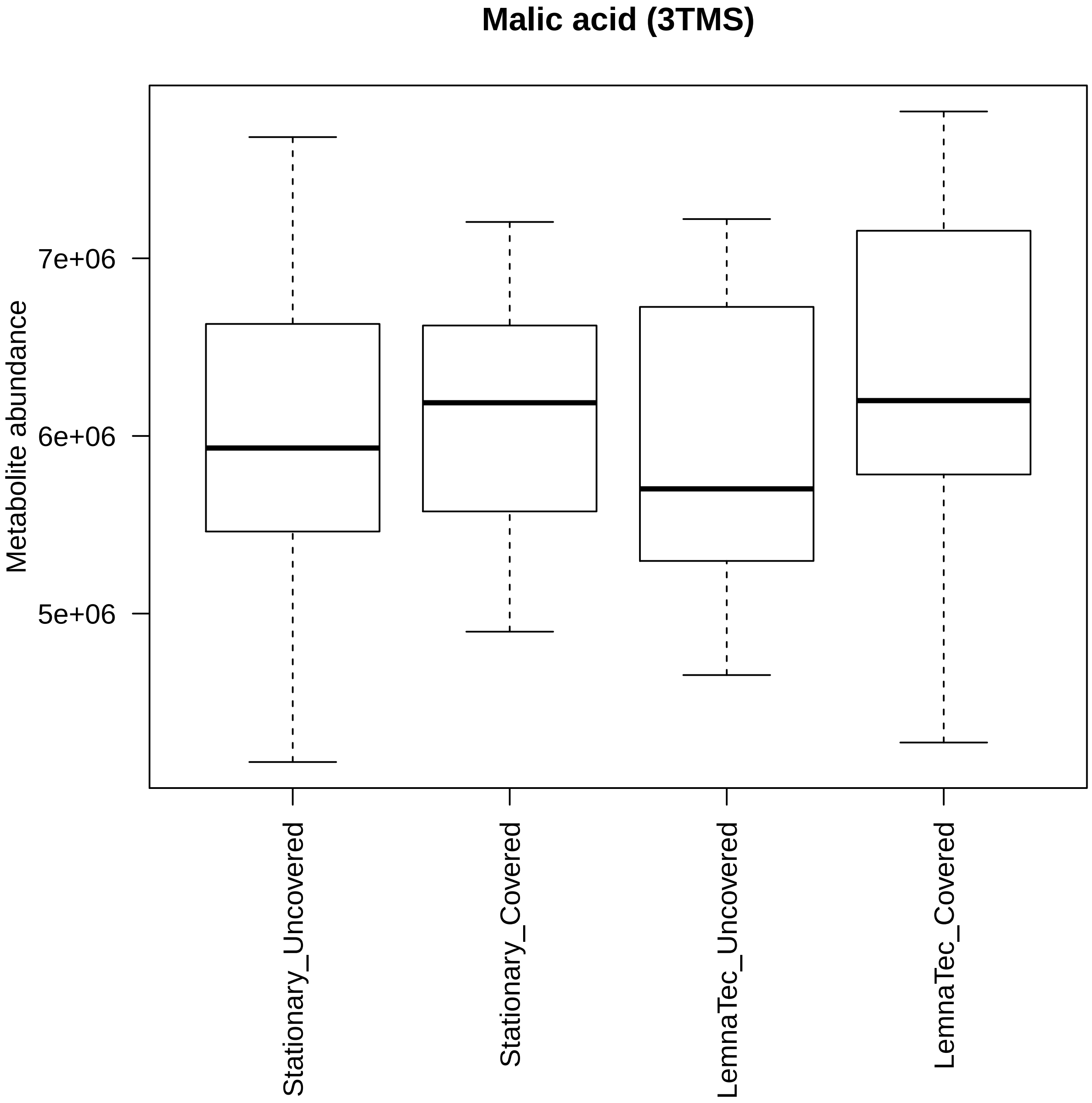

Malic acid (3TMS)
Metabolite abundance
7e+06
6e+06
5e+06
Stationary_Uncovered
Stationary_Covered
LemnaTec_Uncovered
LemnaTec_Covered

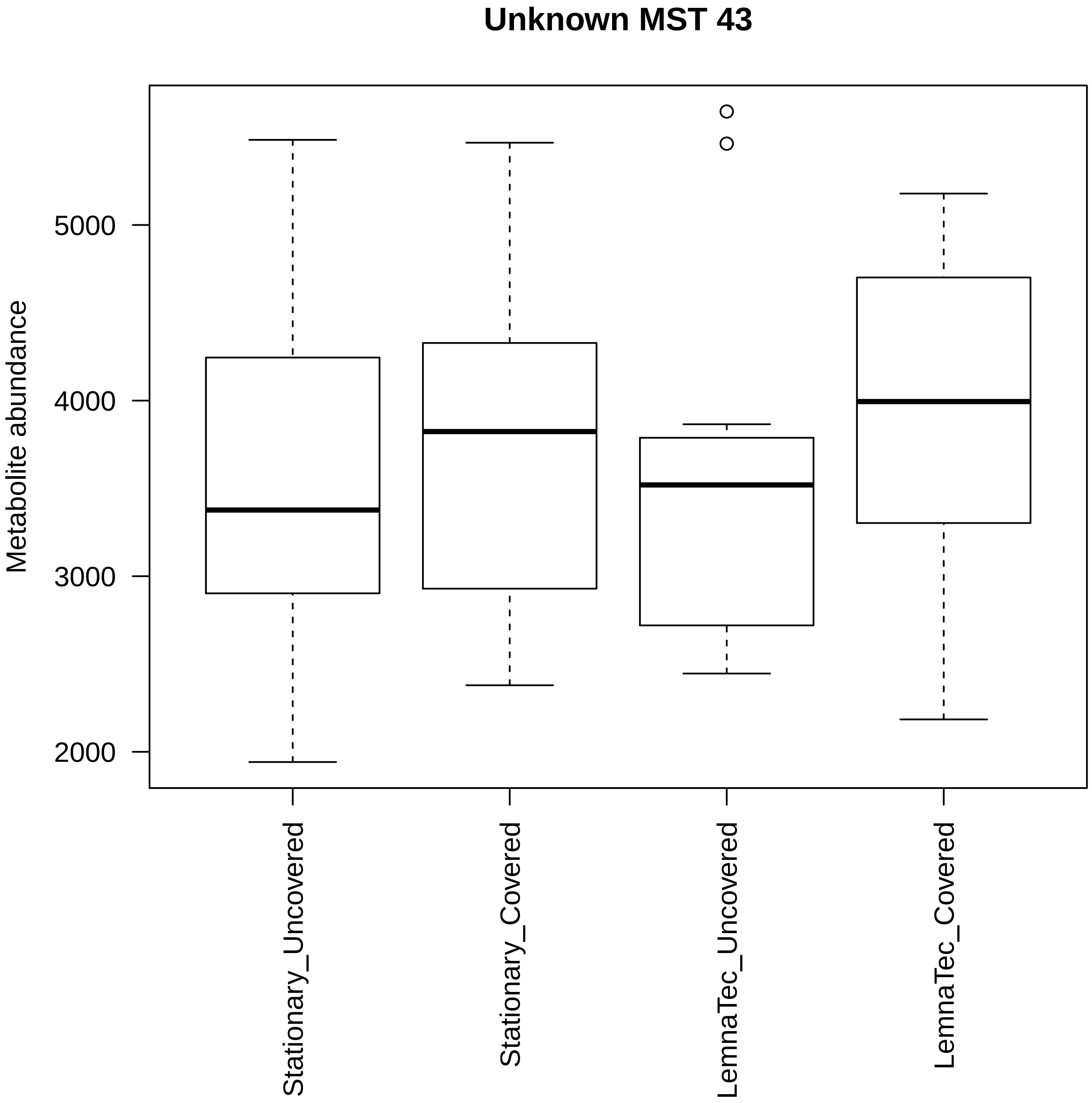

Unknown MST 43
Metabolite abundance
5000
4000
3000
2000
Stationary_Uncovered
Stationary_Covered
LemnaTec_Uncovered
LemnaTec_Covered

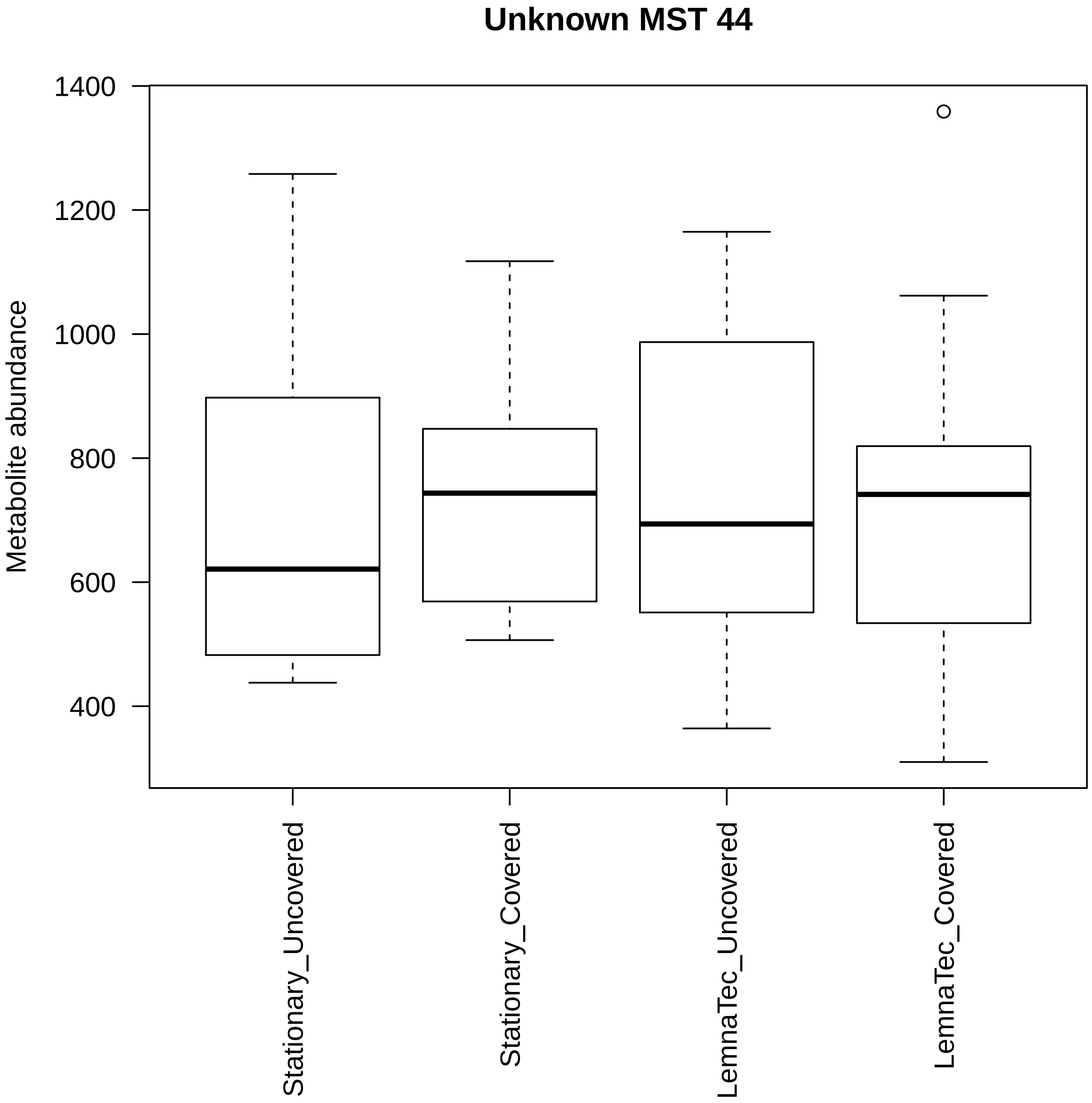
Unknown MST 44
Metabolite abundance
1400
1200
1000
800
600
400
Stationary_Uncovered
Stationary_Covered
LemnaTec_Uncovered
LemnaTec_Covered

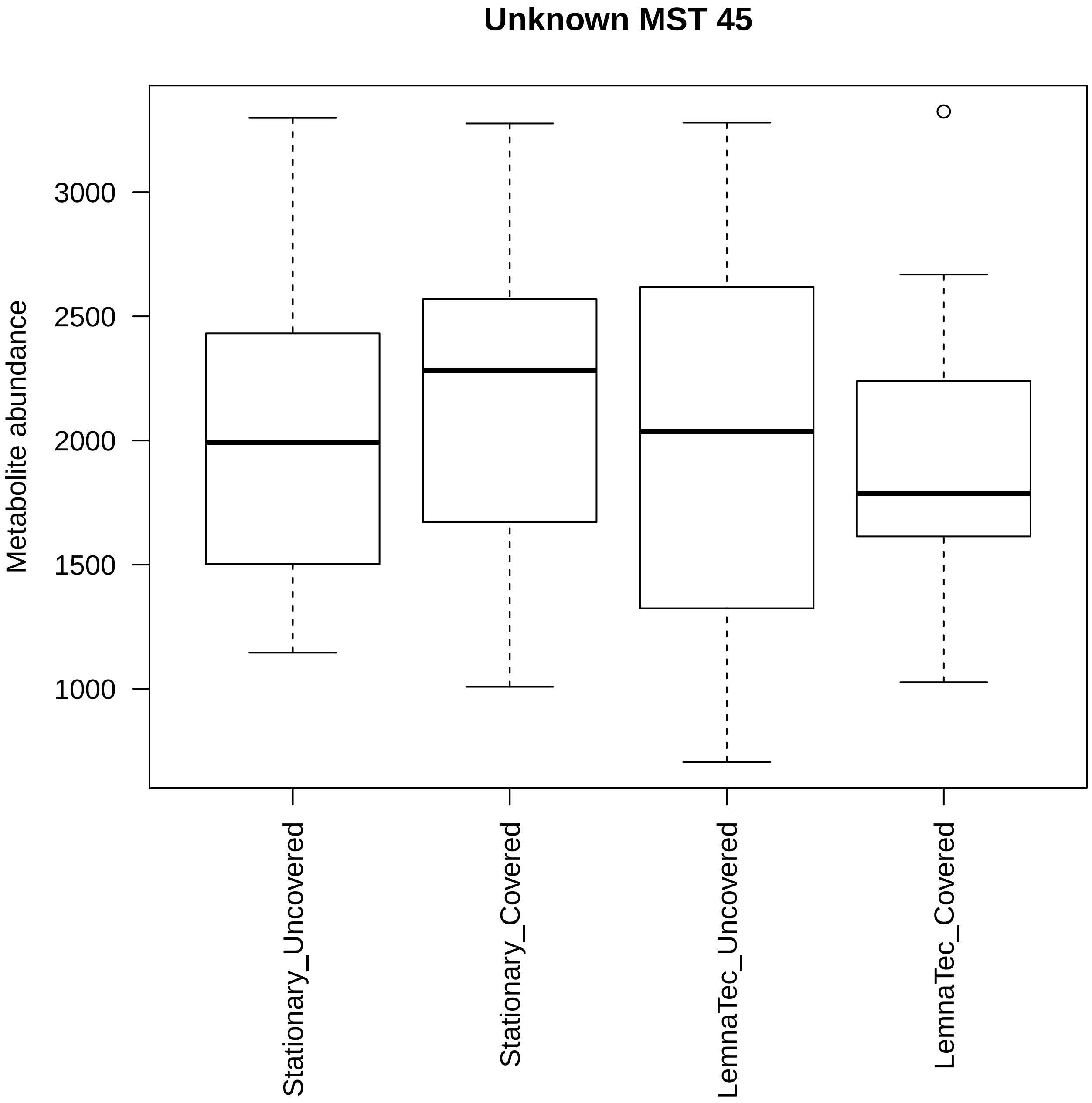
Unknown MST 45
Metabolite abundance
3000
2500
2000
1500
1000
Stationary_Uncovered
Stationary_Covered
LemnaTec_Uncovered
LemnaTec_Covered

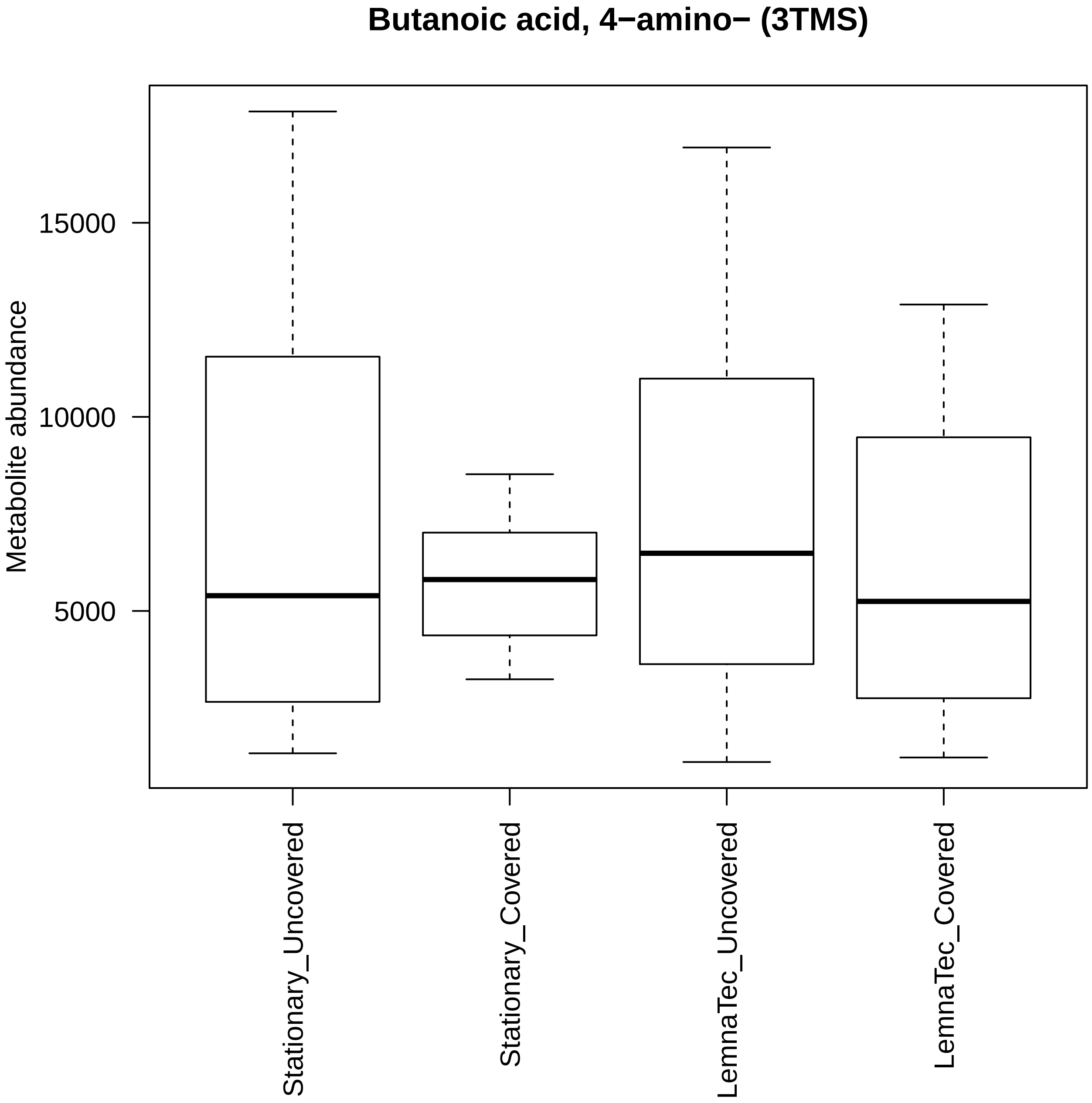
Butanoic acid, 4−amino− (3TMS)
Metabolite abundance
15000
10000
5000
Stationary_Uncovered
Stationary_Covered
LemnaTec_Uncovered
LemnaTec_Covered

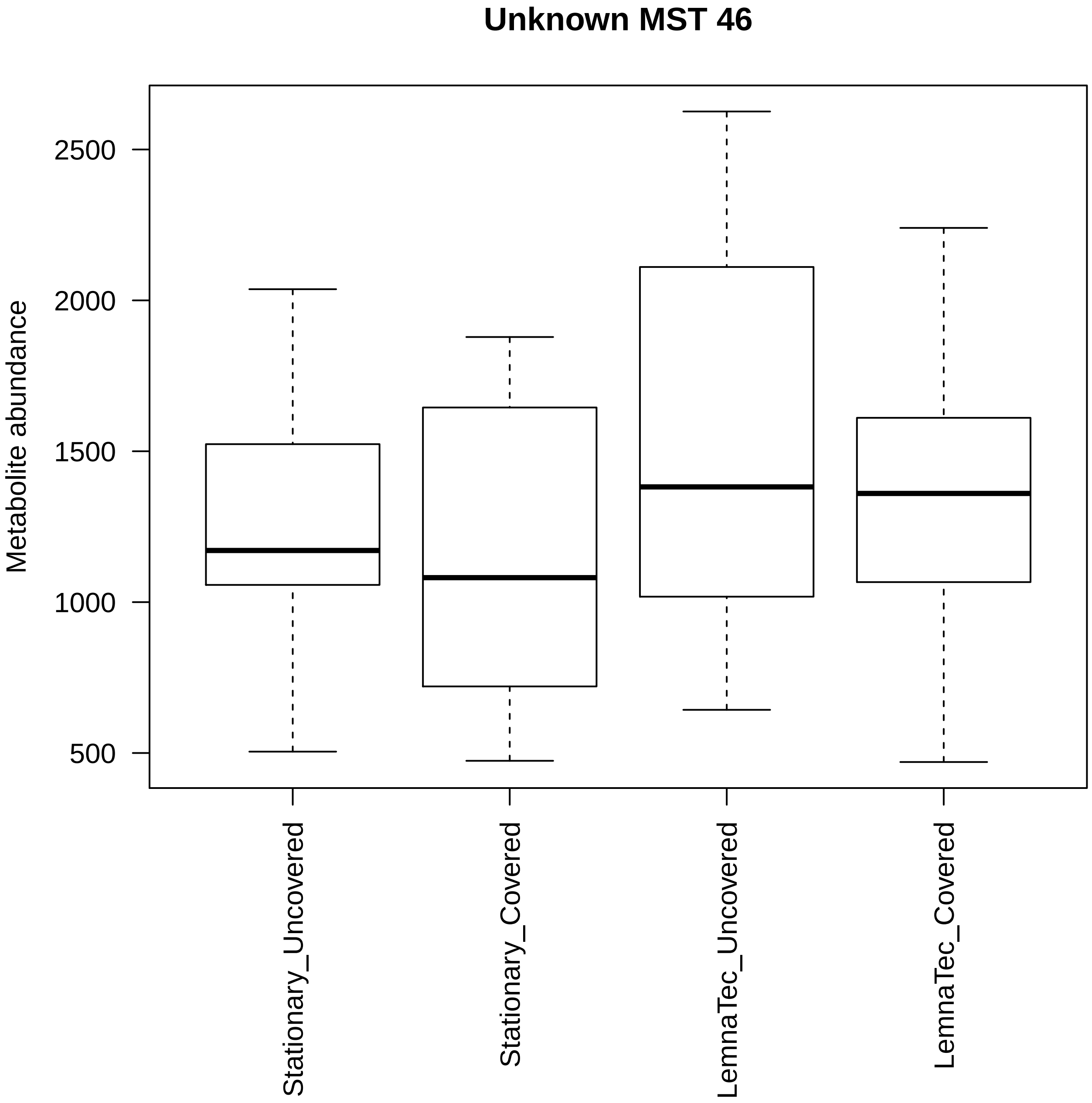

Unknown MST 46
Metabolite abundance
2500
2000
1500
1000
500
Stationary_Uncovered
Stationary_Covered
LemnaTec_Uncovered
LemnaTec_Covered

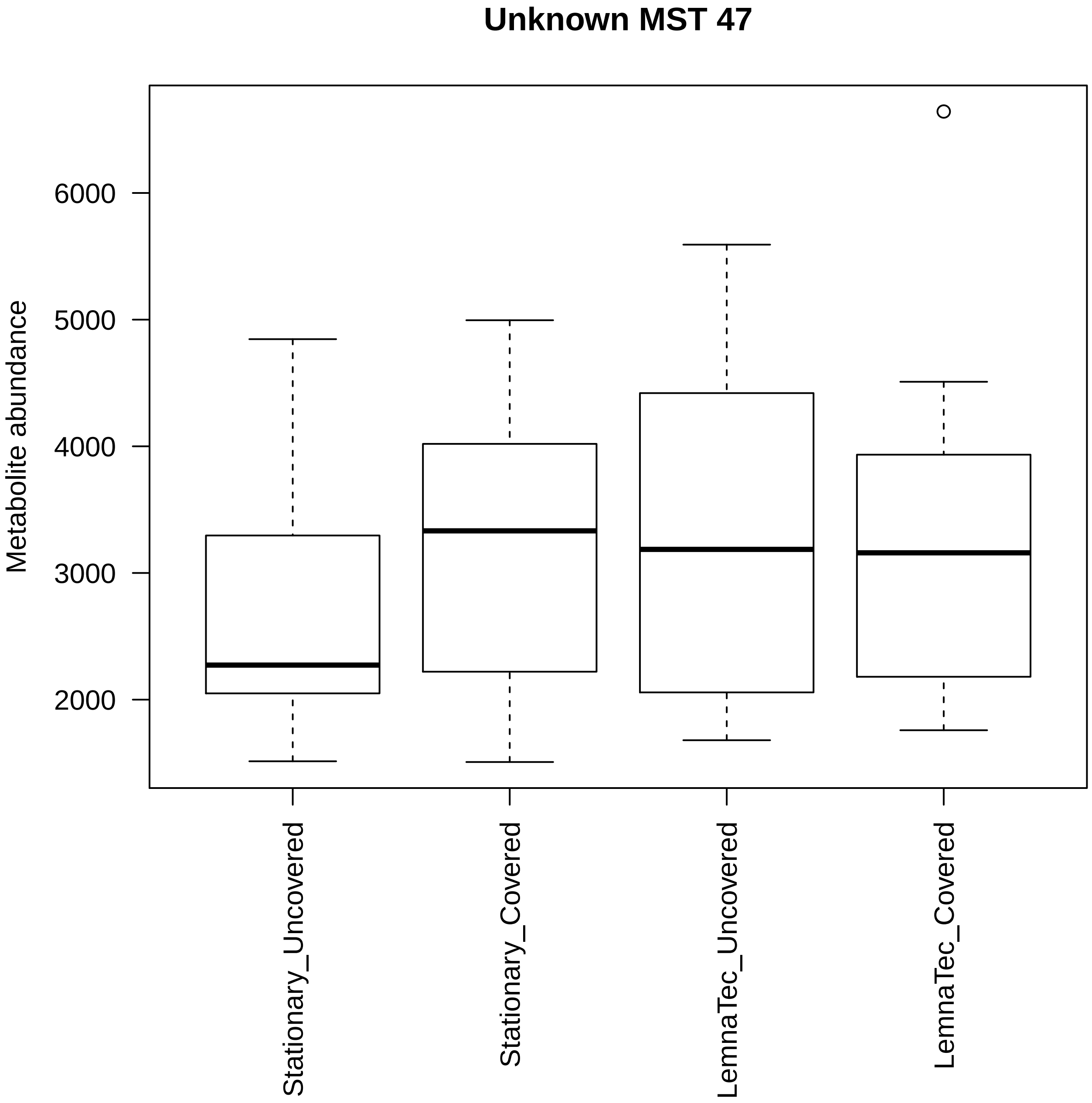

Unknown MST 47
Metabolite abundance
2000
3000
4000
5000
6000
Stationary_Uncovered
Stationary_Covered
LemnaTec_Uncovered
LemnaTec_Covered

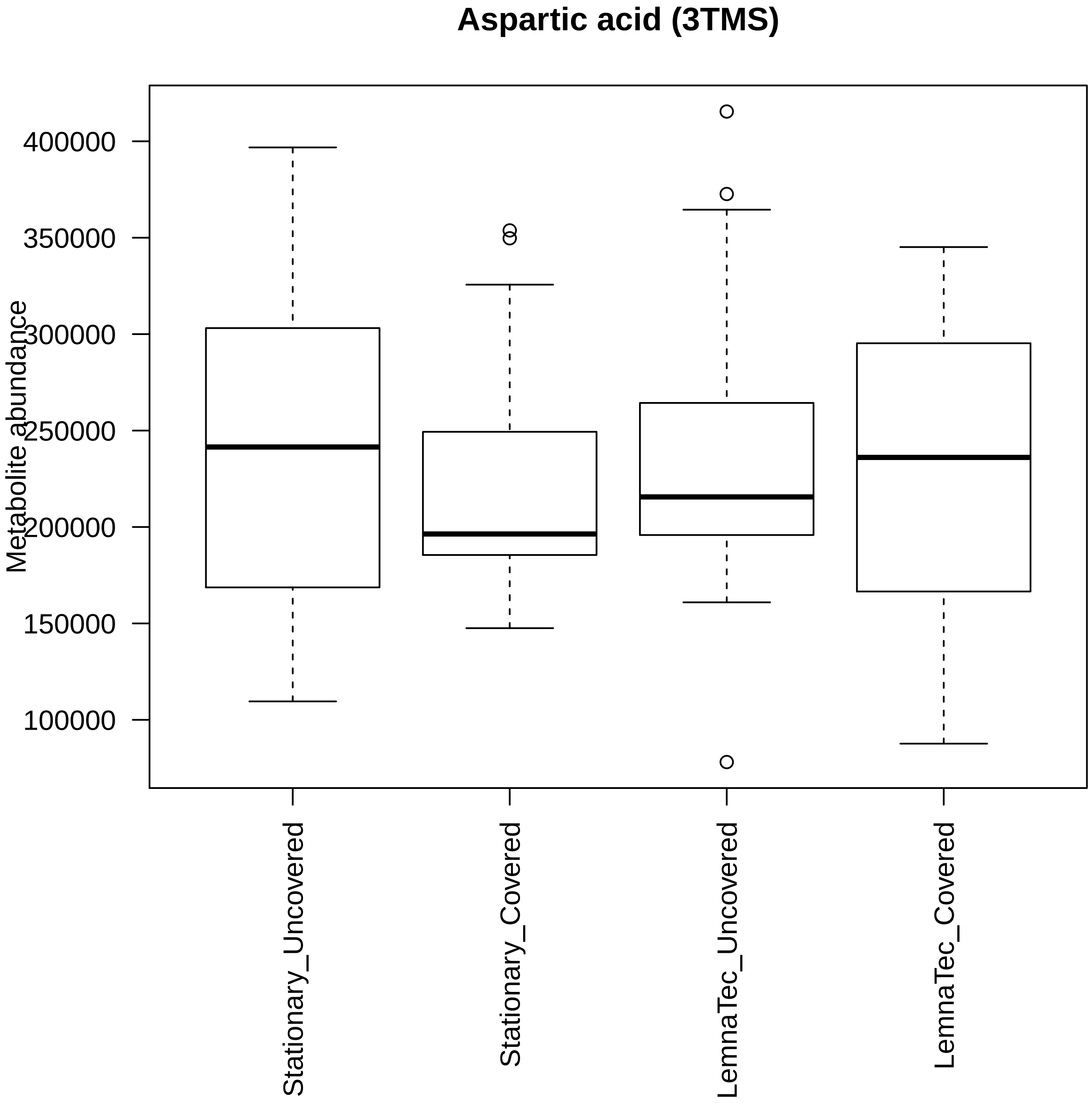

Aspartic acid (3TMS)
Metabolite abundance
400000
350000
300000
250000
200000
150000
100000
Stationary_Uncovered
Stationary_Covered
LemnaTec_Uncovered
LemnaTec_Covered

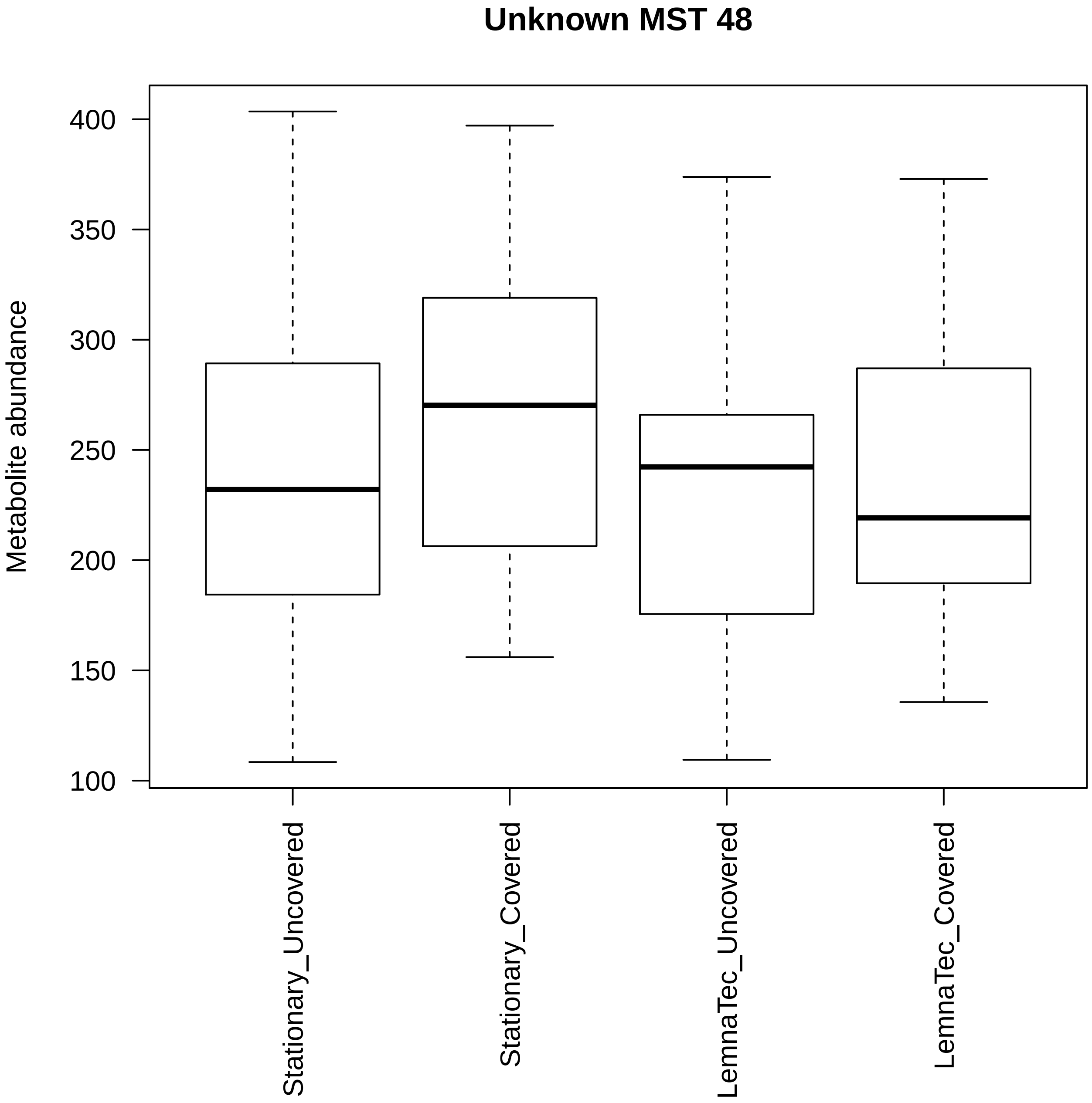
Unknown MST 48
Metabolite abundance
400
350
300
250
200
150
100
Stationary_Uncovered
Stationary_Covered
LemnaTec_Uncovered
LemnaTec_Covered

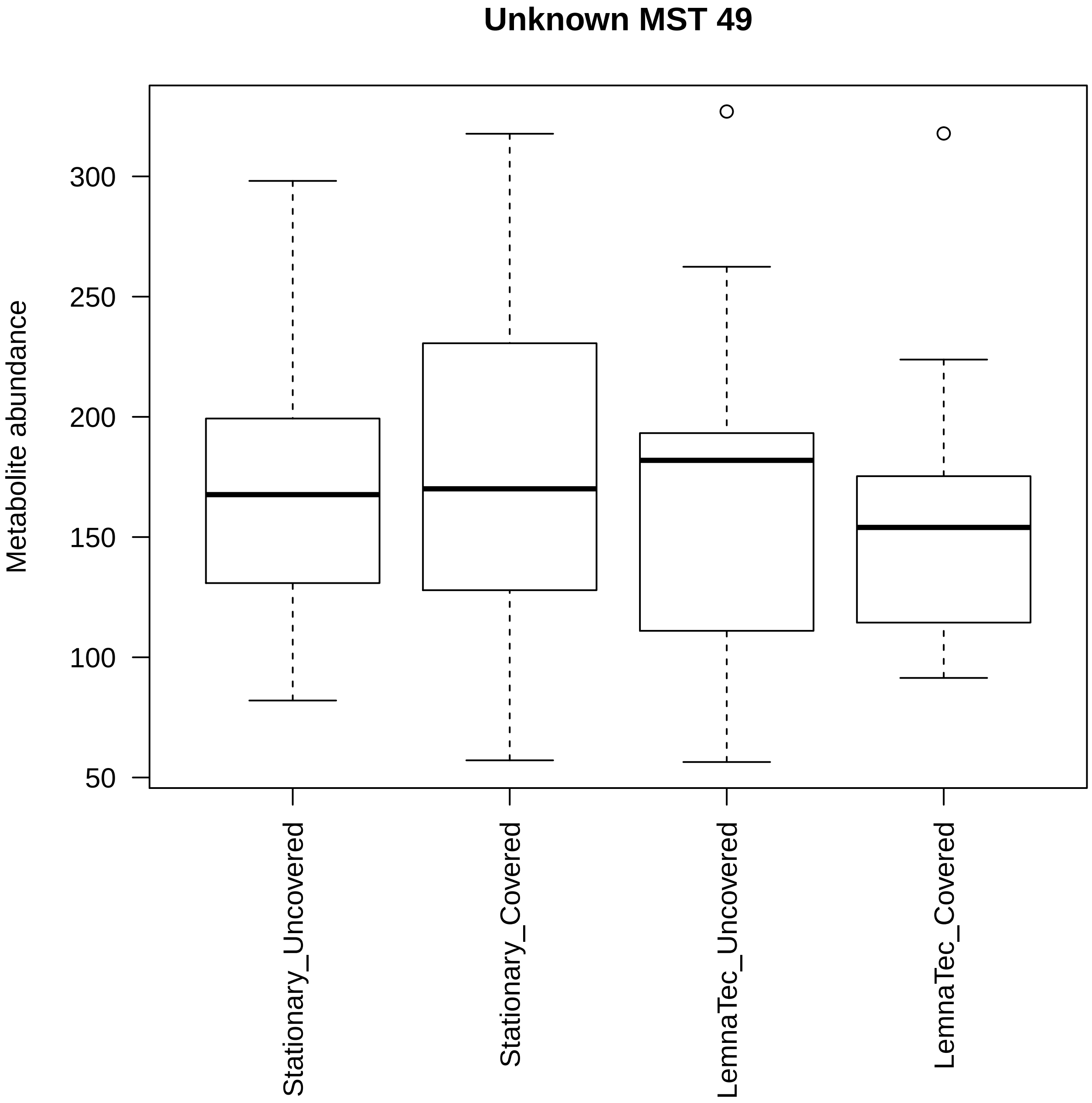

Unknown MST 49
Metabolite abundance
300
250
200
150
100
50
Stationary_Uncovered
Stationary_Covered
LemnaTec_Uncovered
LemnaTec_Covered

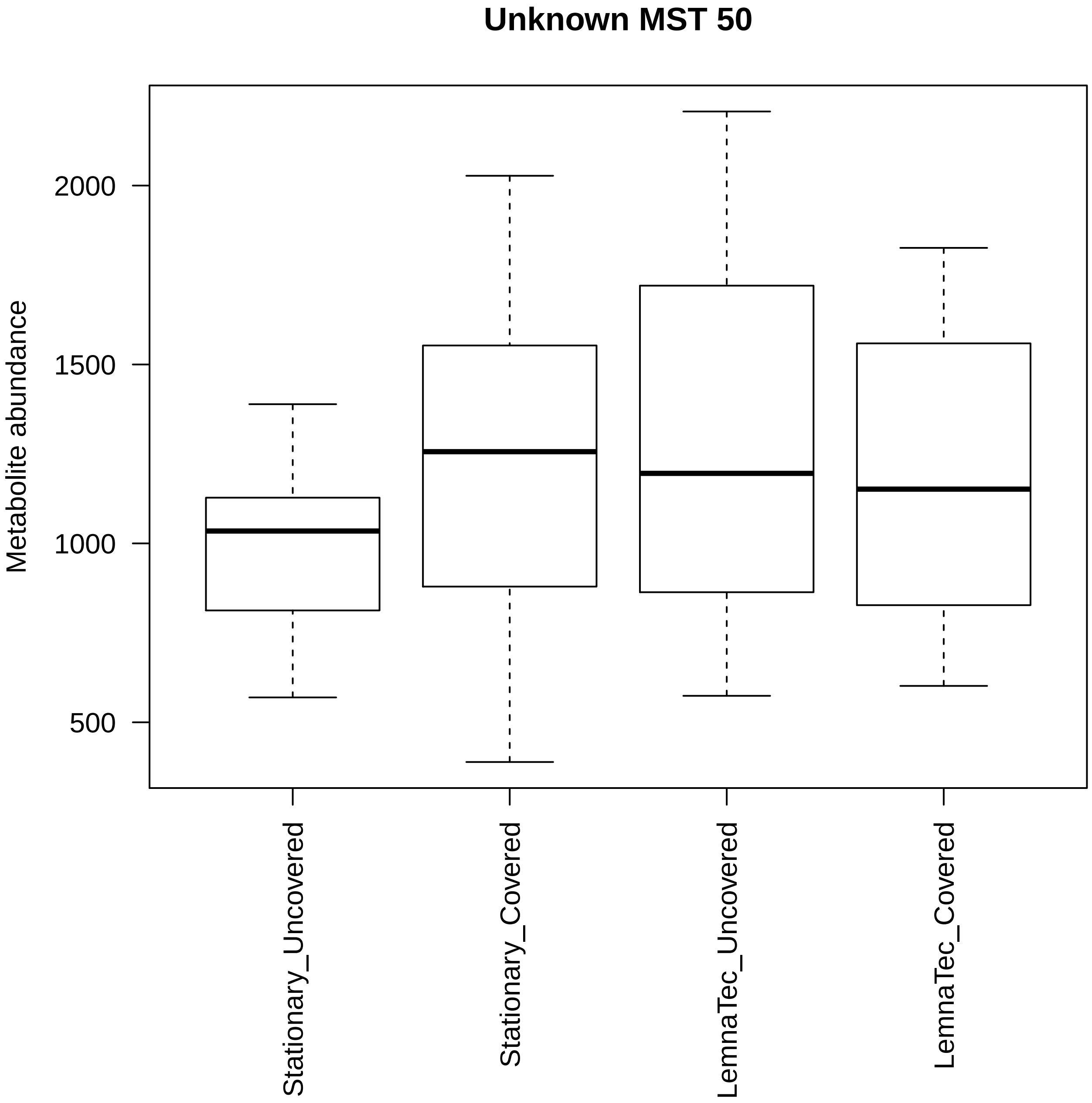

Unknown MST 50
Metabolite abundance
2000
1500
1000
500
Stationary_Uncovered
Stationary_Covered
LemnaTec_Uncovered
LemnaTec_Covered

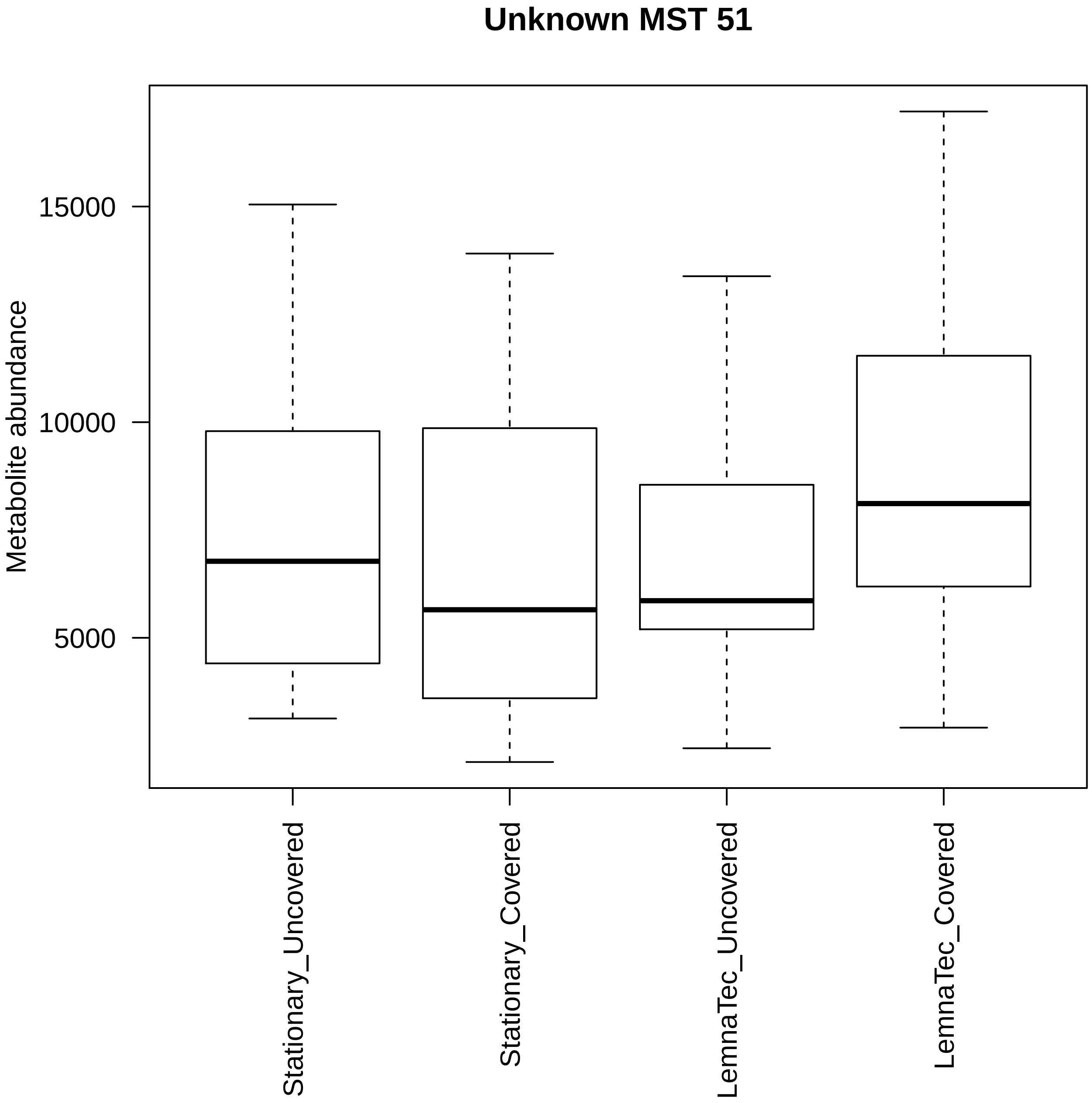

Unknown MST 51
Metabolite abundance
15000
10000
5000
Stationary_Uncovered
Stationary_Covered
LemnaTec_Uncovered
LemnaTec_Covered

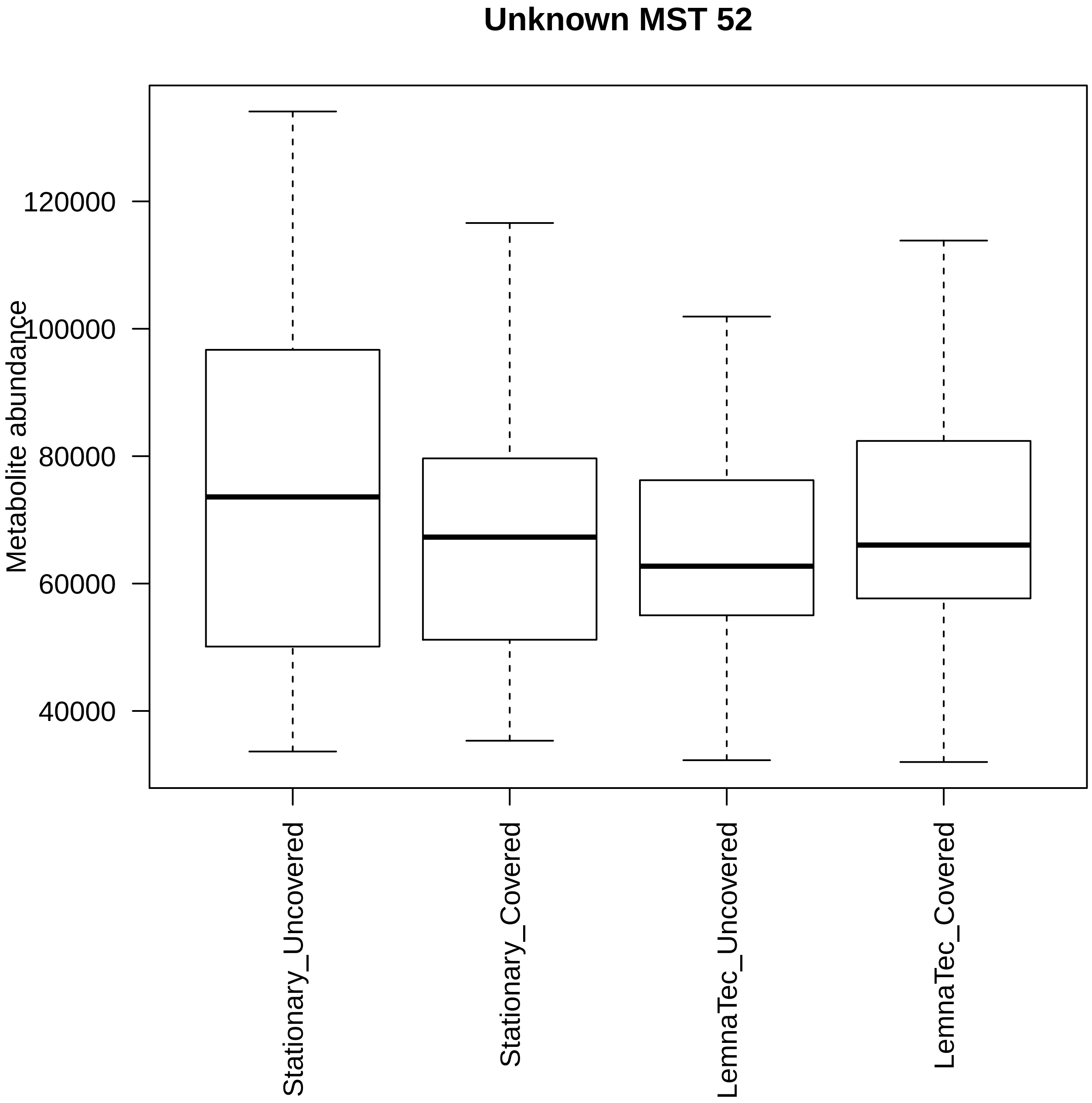
Unknown MST 52
Metabolite abundance
120000
100000
80000
60000
40000
Stationary_Uncovered
Stationary_Covered
LemnaTec_Uncovered
LemnaTec_Covered

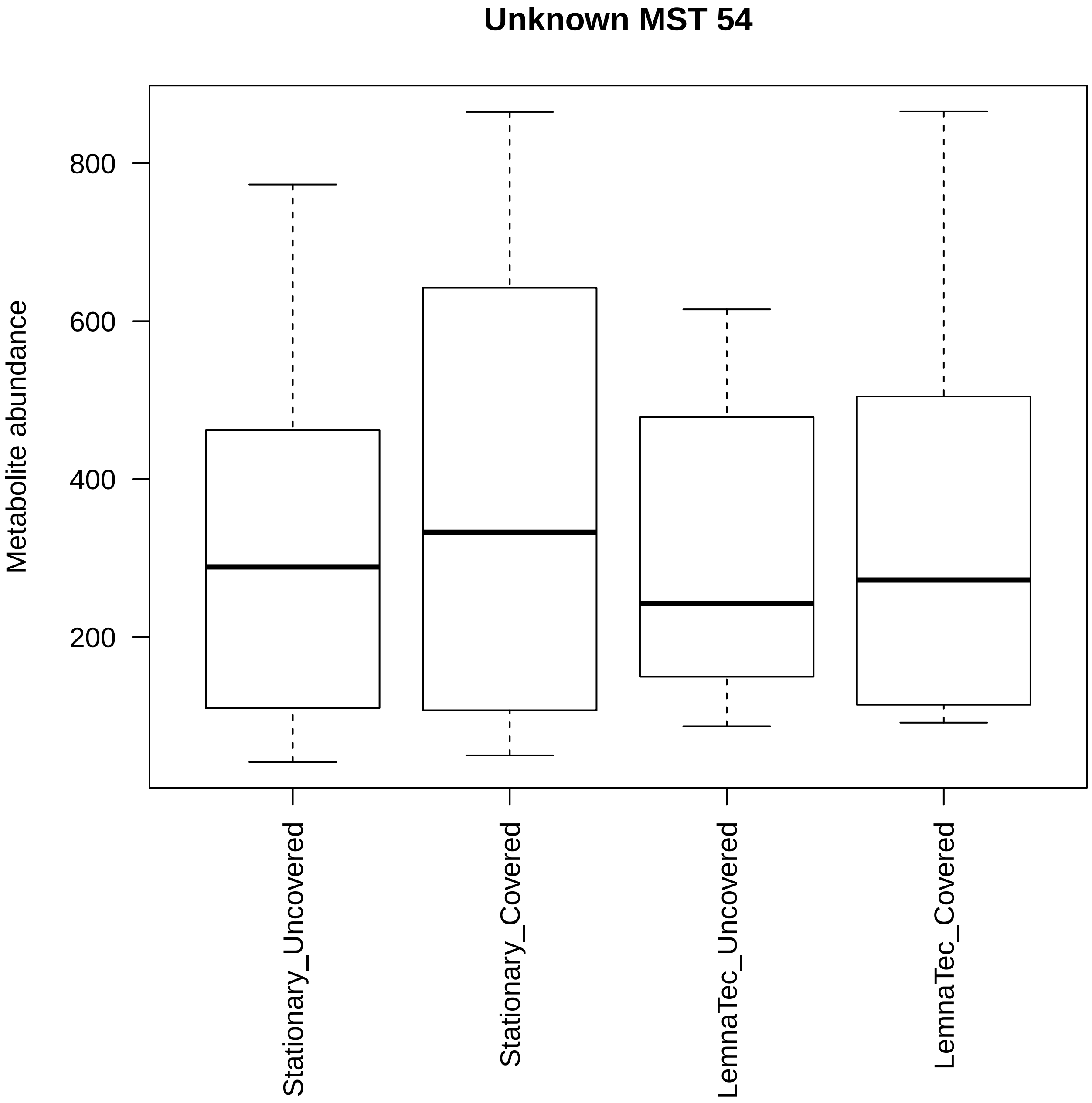
Unknown MST 54
Metabolite abundance
800
600
400
200
Stationary_Uncovered
Stationary_Covered
LemnaTec_Uncovered
LemnaTec_Covered

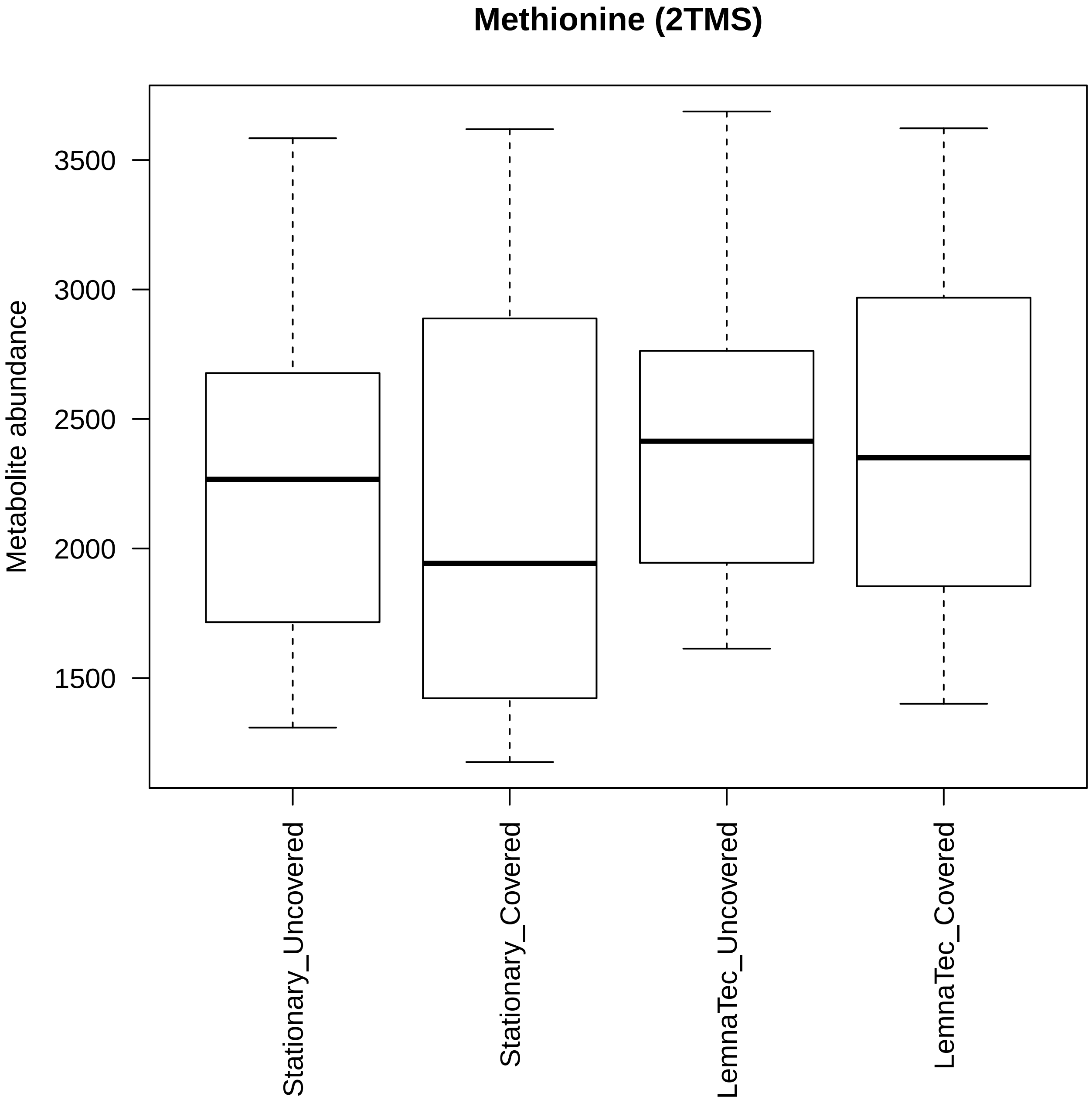

Methionine (2TMS)
Metabolite abundance
3500
3000
2500
2000
1500
Stationary_Uncovered
Stationary_Covered
LemnaTec_Uncovered
LemnaTec_Covered

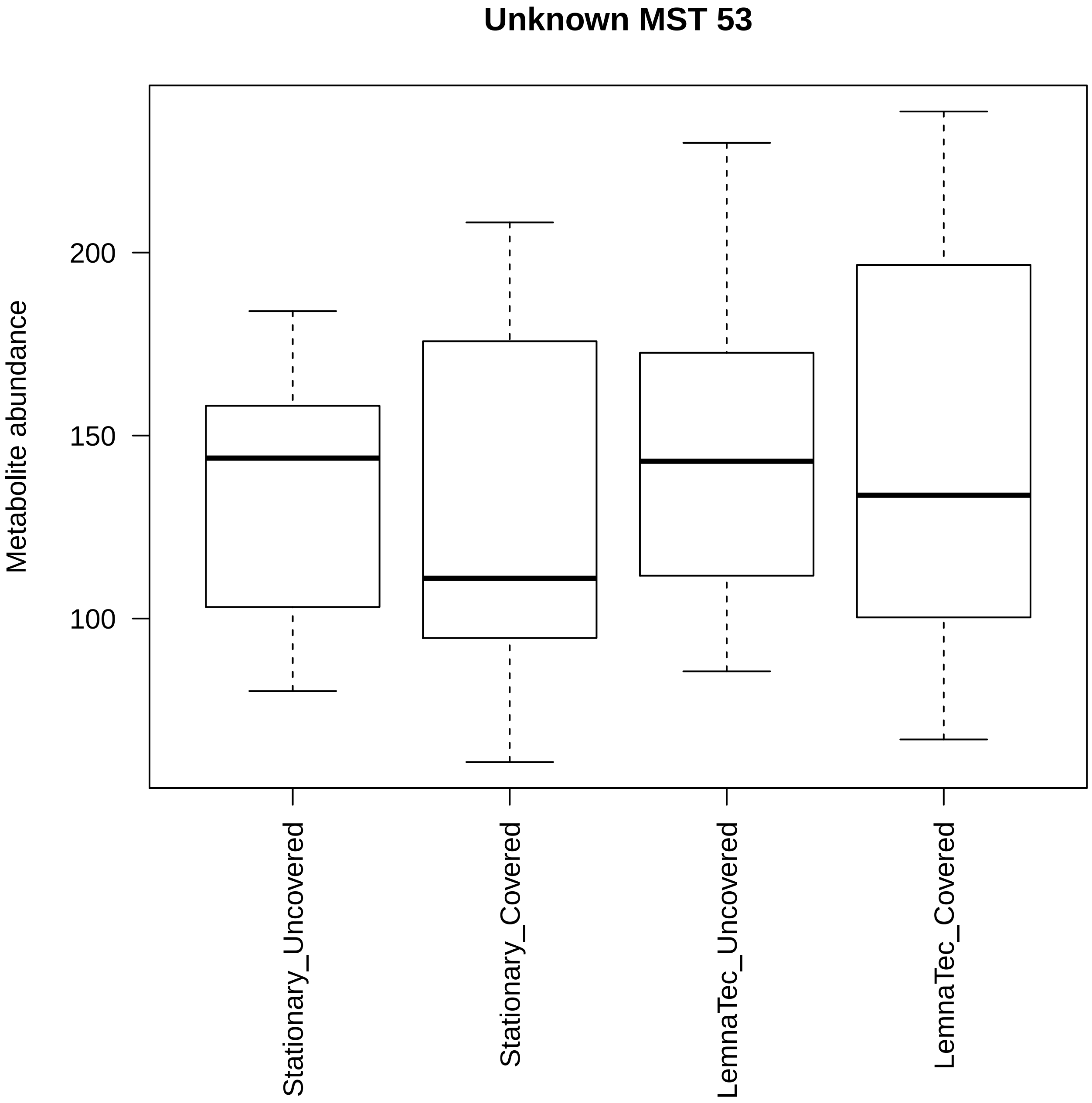

Unknown MST 53
Metabolite abundance
200
150
100
Stationary_Uncovered
Stationary_Covered
LemnaTec_Uncovered
LemnaTec_Covered

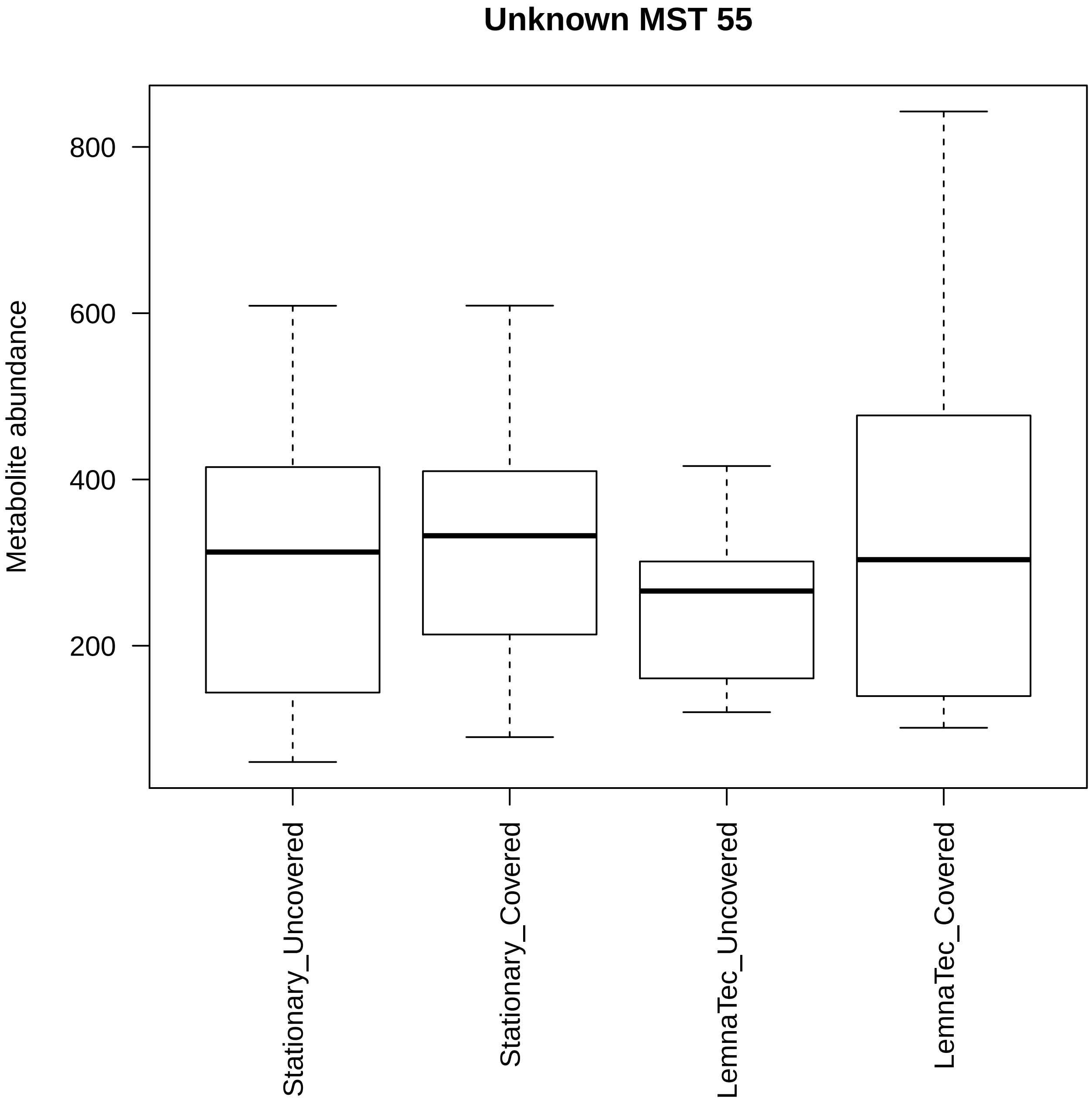

Unknown MST 55
Metabolite abundance
800
600
400
200
Stationary_Uncovered
Stationary_Covered
LemnaTec_Uncovered
LemnaTec_Covered

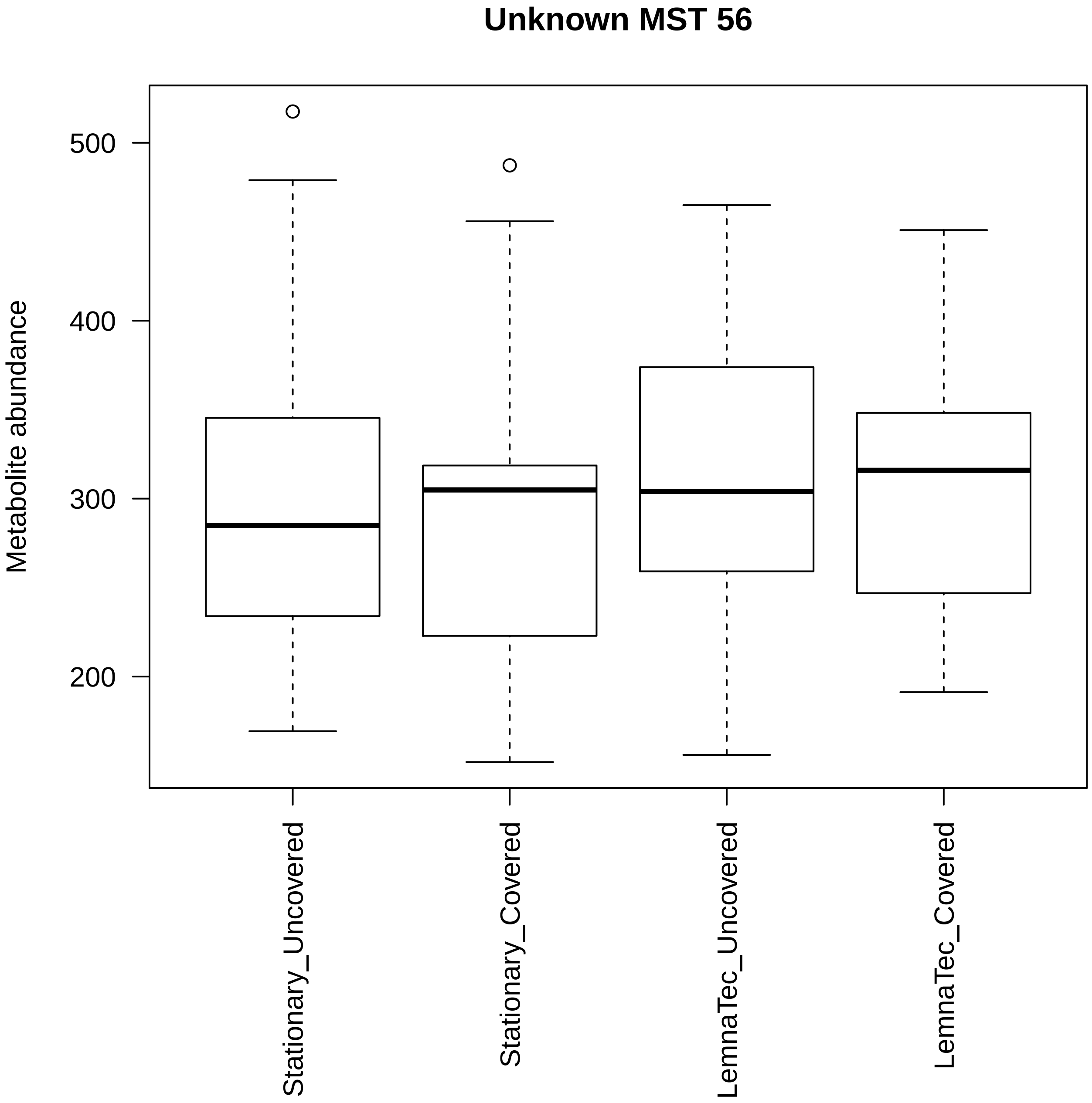
Unknown MST 56
Metabolite abundance
500
400
300
200
Stationary_Uncovered
Stationary_Covered
LemnaTec_Uncovered
LemnaTec_Covered

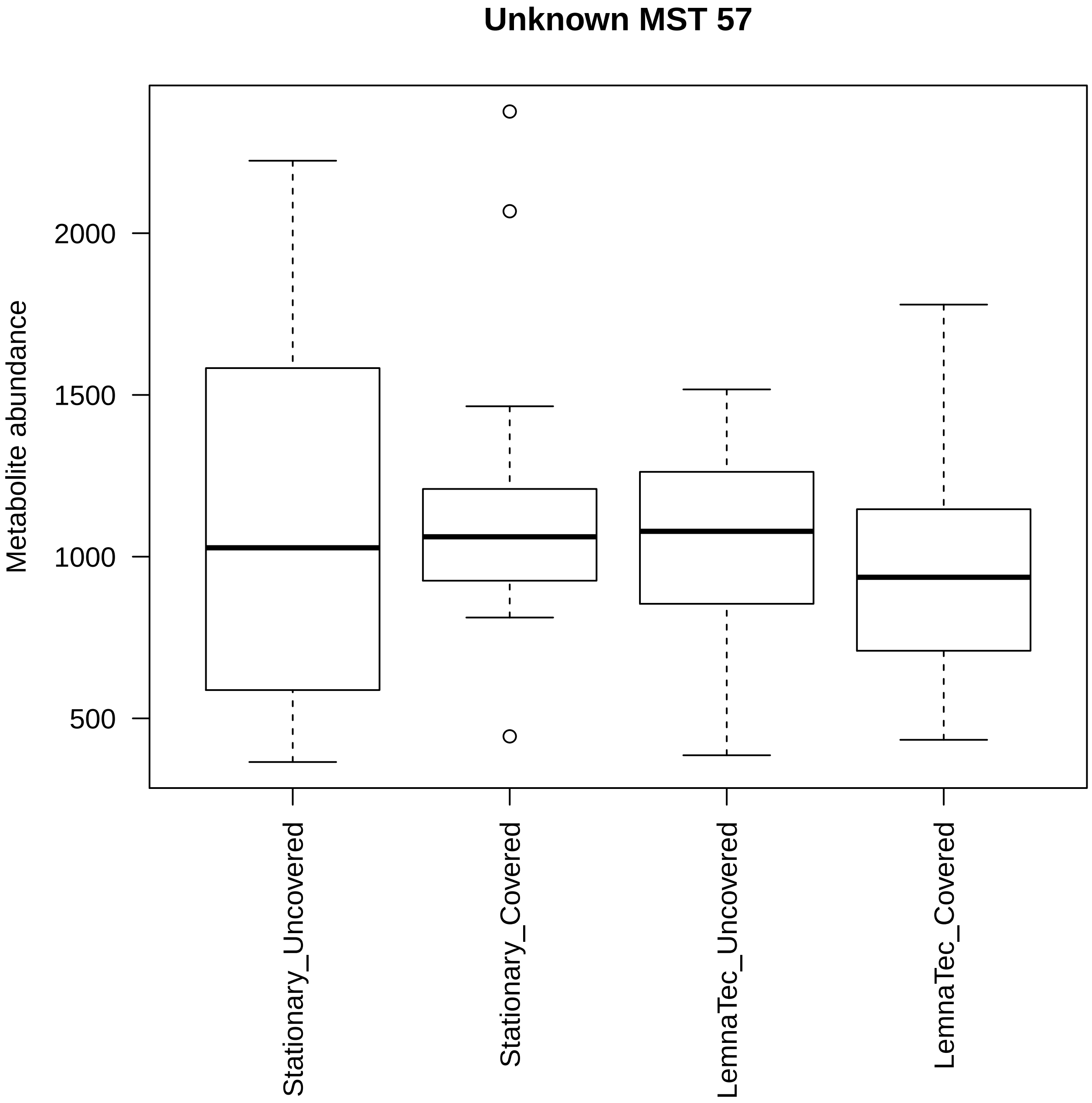

Unknown MST 57
Metabolite abundance
2000
1500
1000
500
Stationary_Uncovered
Stationary_Covered
LemnaTec_Uncovered
LemnaTec_Covered

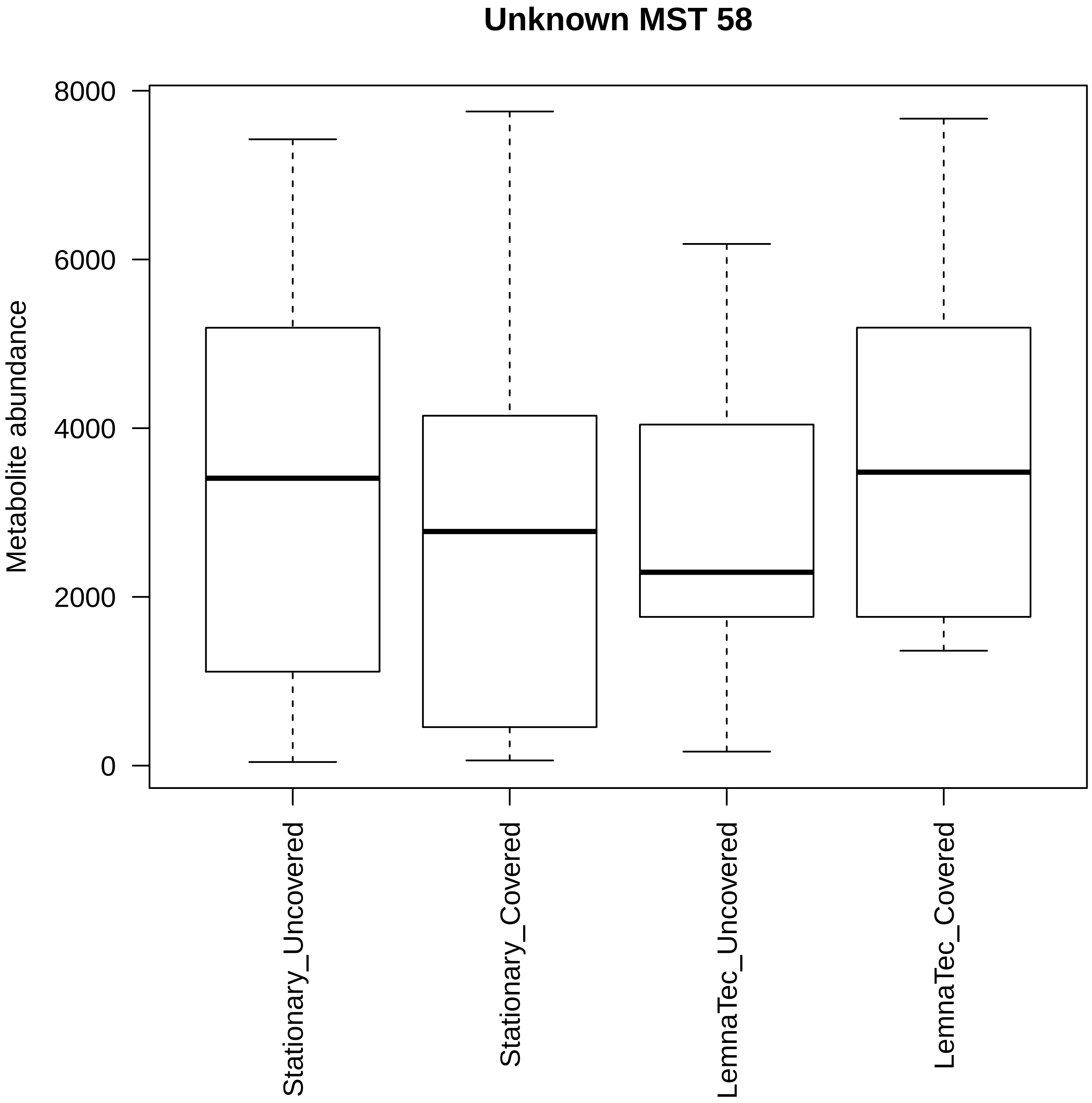

Unknown MST 58
Metabolite abundance
8000
6000
4000
2000
0
Stationary_Uncovered
Stationary_Covered
LemnaTec_Uncovered
LemnaTec_Covered

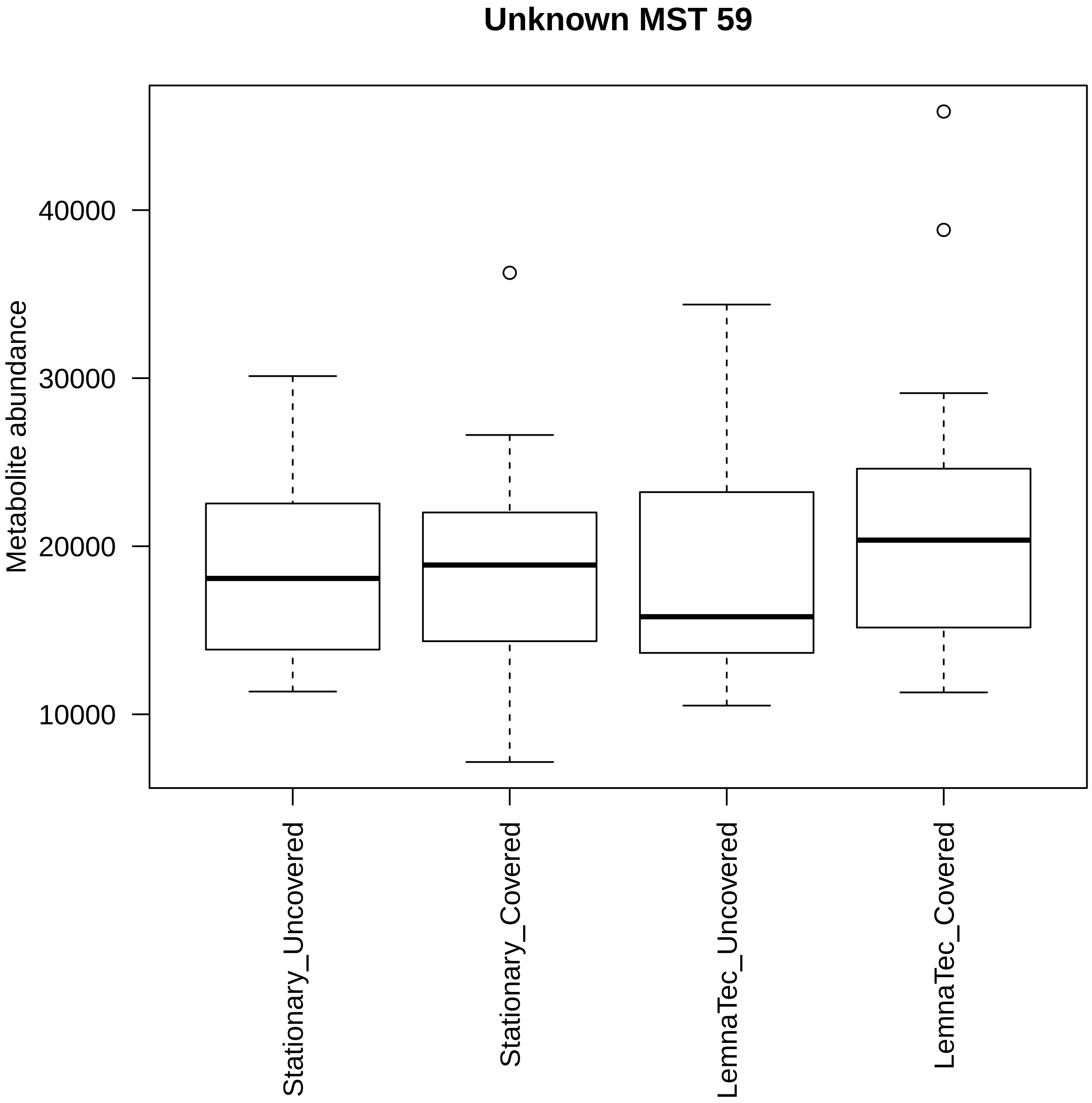

Unknown MST 59
Metabolite abundance
40000
30000
20000
10000
Stationary_Uncovered
Stationary_Covered
LemnaTec_Uncovered
LemnaTec_Covered

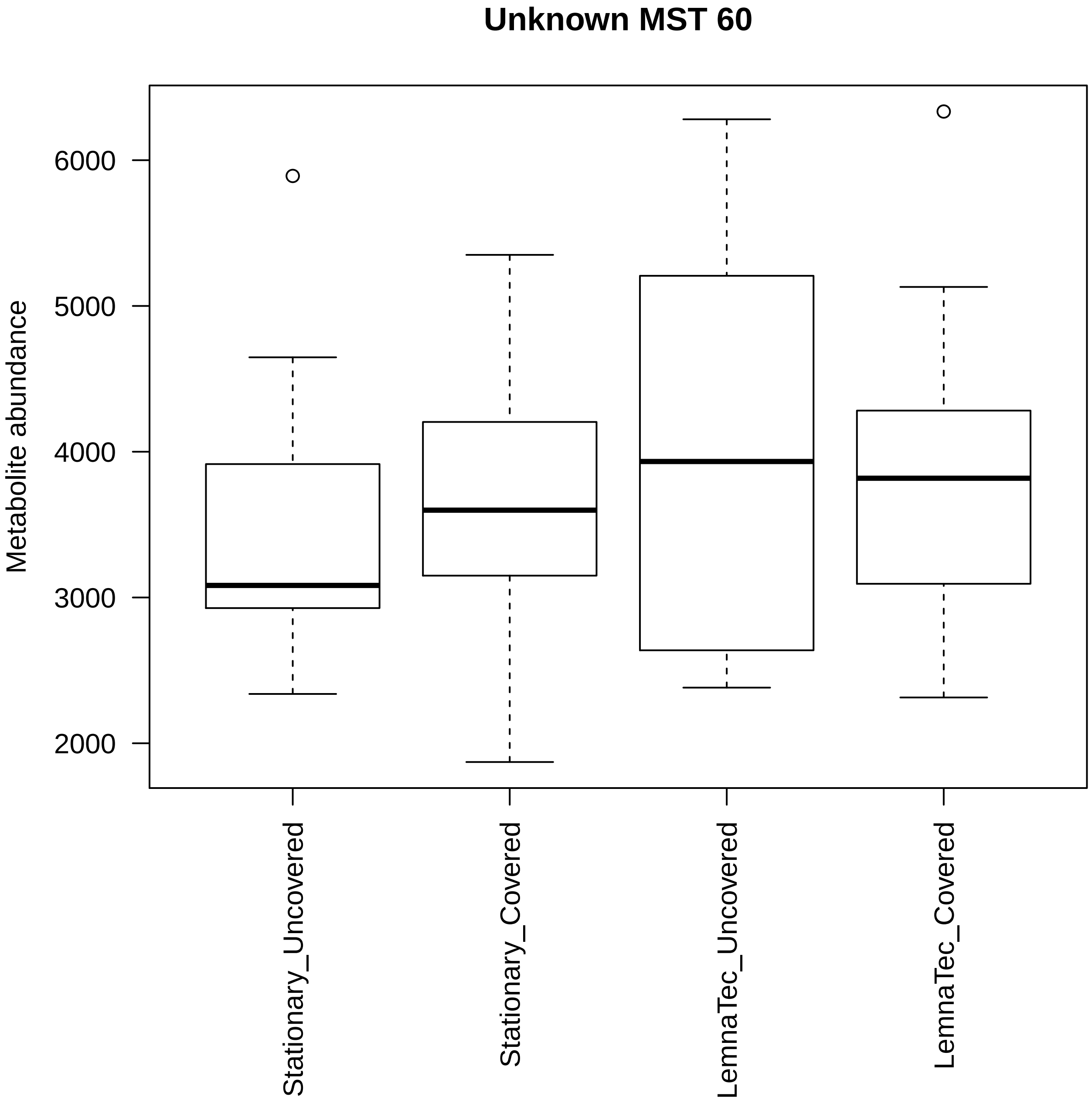
Unknown MST 60
Metabolite abundance
2000
3000
4000
5000
6000
Stationary_Uncovered
Stationary_Covered
LemnaTec_Uncovered
LemnaTec_Covered

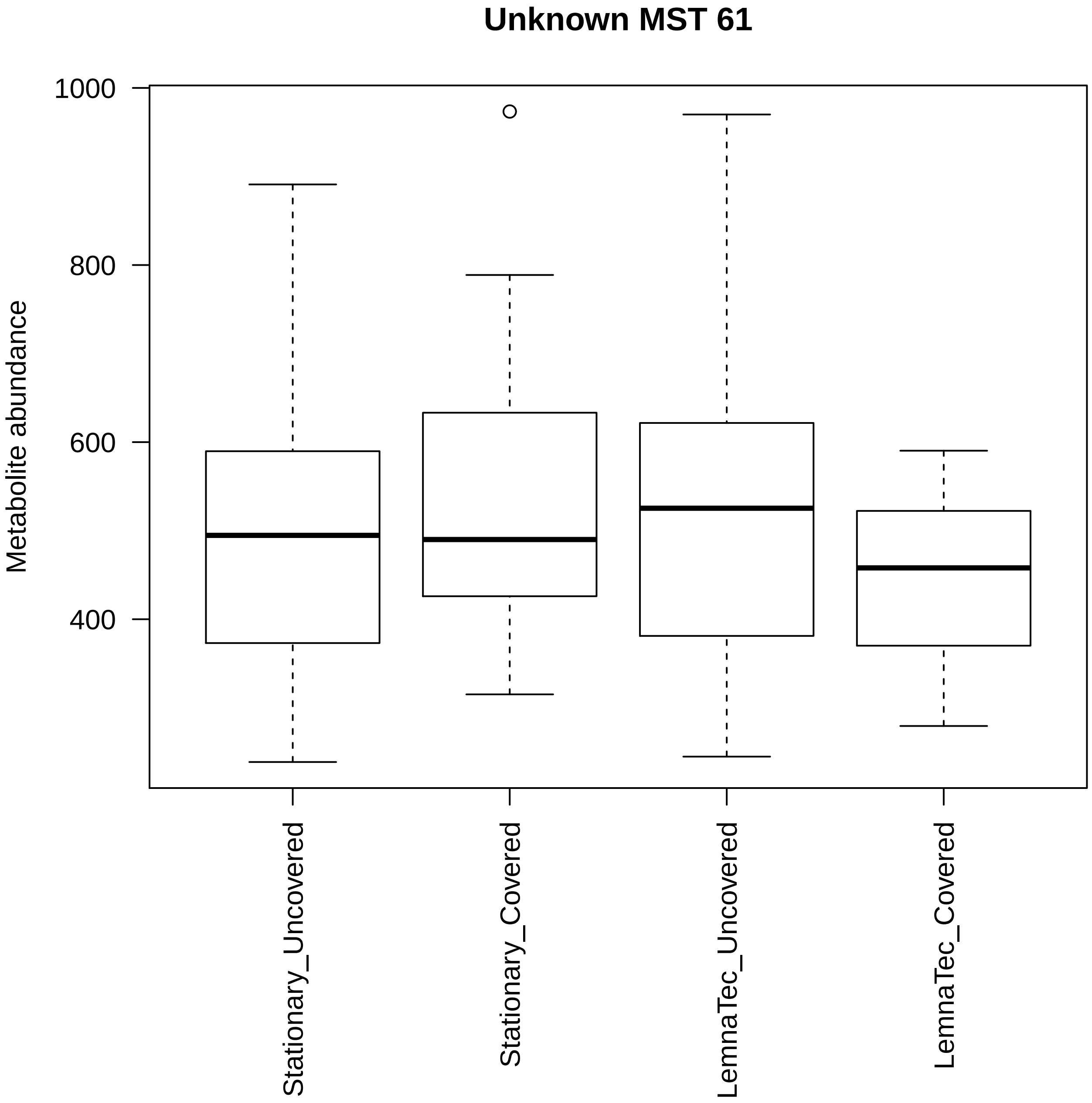
Unknown MST 61
Metabolite abundance
1000
800
600
400
Stationary_Uncovered
Stationary_Covered
LemnaTec_Uncovered
LemnaTec_Covered

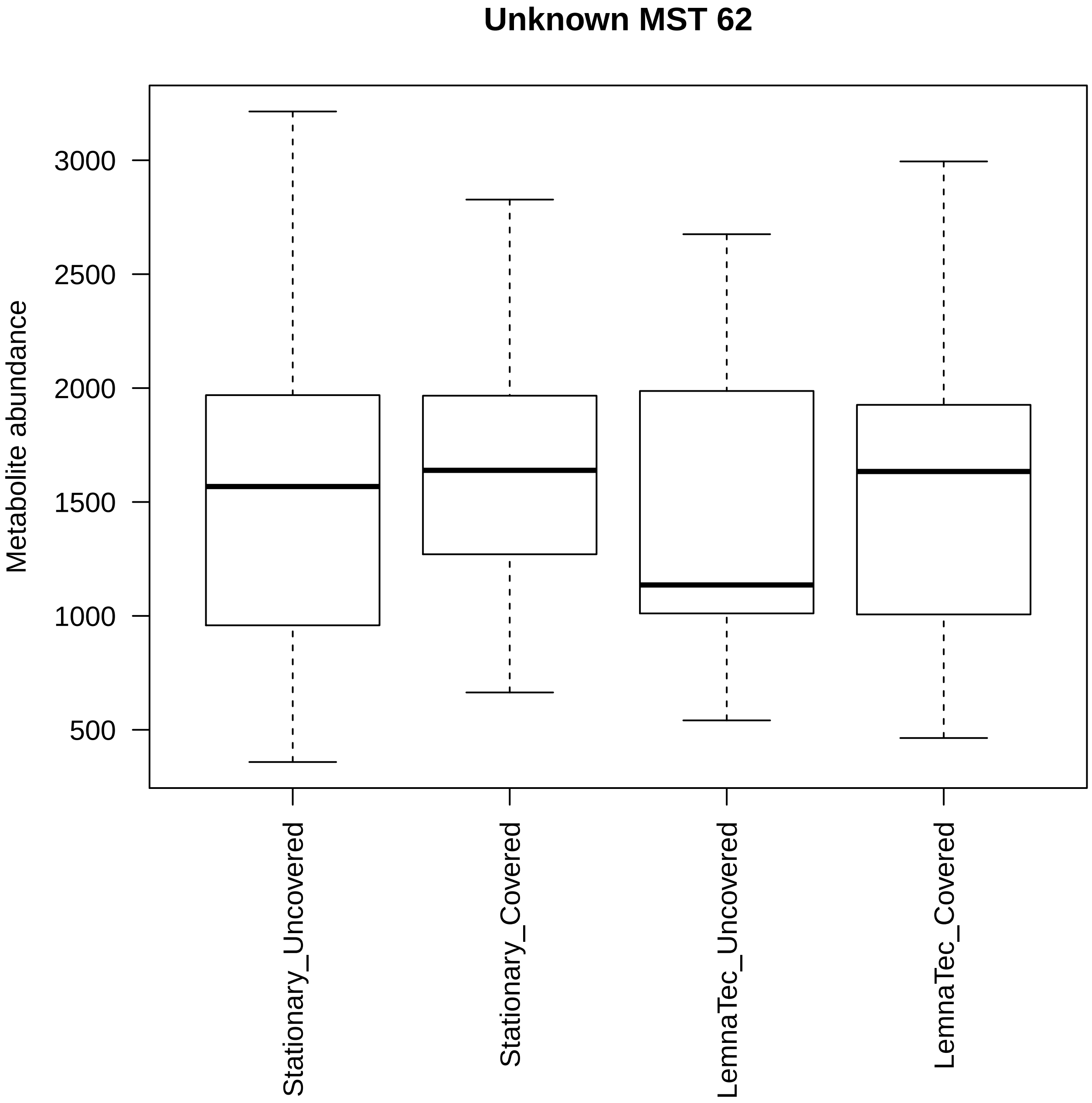
Unknown MST 62
Metabolite abundance
500
1000
1500
2000
2500
3000
Stationary_Uncovered
Stationary_Covered
LemnaTec_Uncovered
LemnaTec_Covered

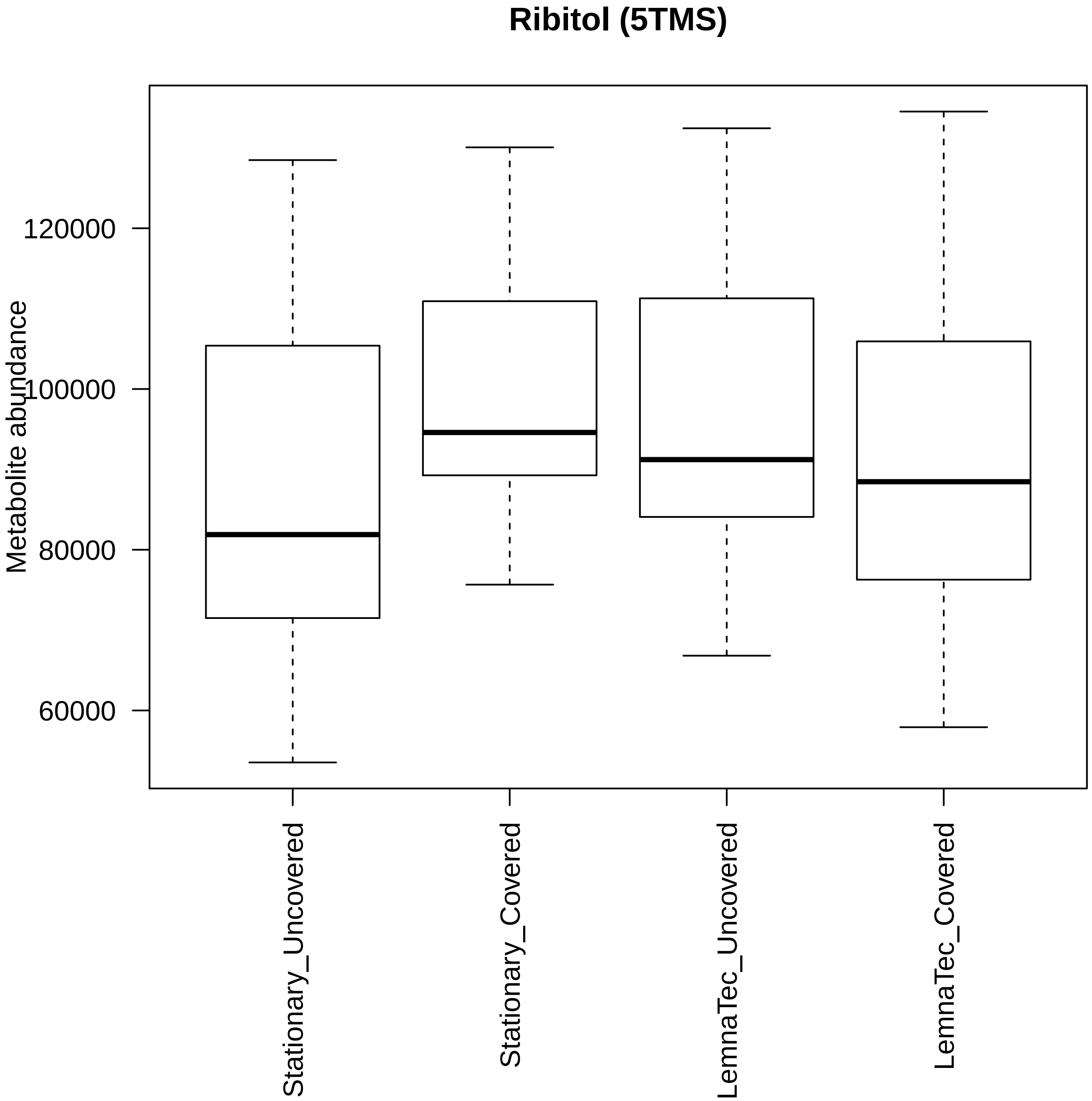
Ribitol (5TMS)
Metabolite abundance
120000
100000
80000
60000
Stationary_Uncovered
Stationary_Covered
LemnaTec_Uncovered
LemnaTec_Covered

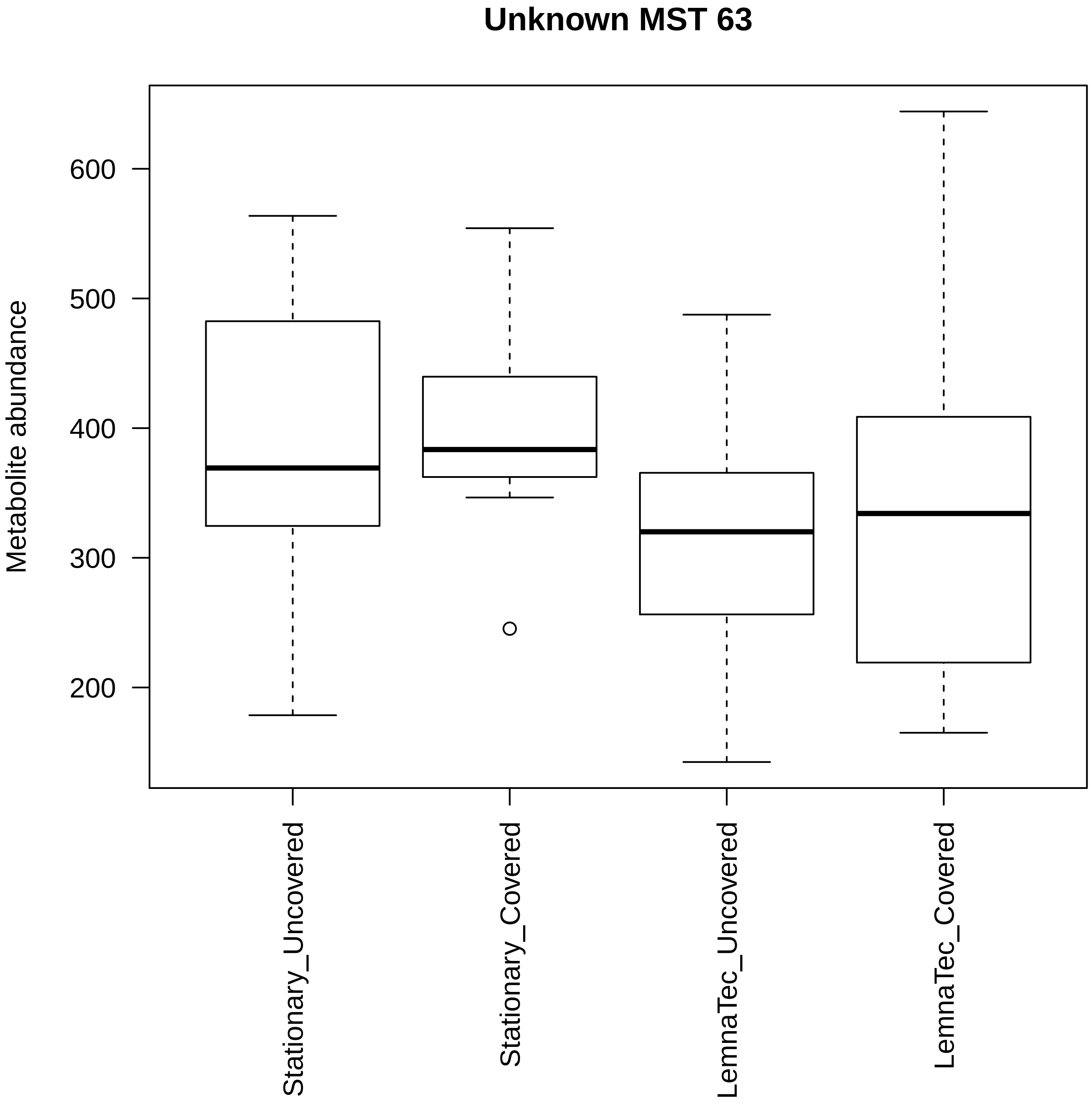

Unknown MST 63
Metabolite abundance
600
500
400
300
200
Stationary_Uncovered
Stationary_Covered
LemnaTec_Uncovered
LemnaTec_Covered

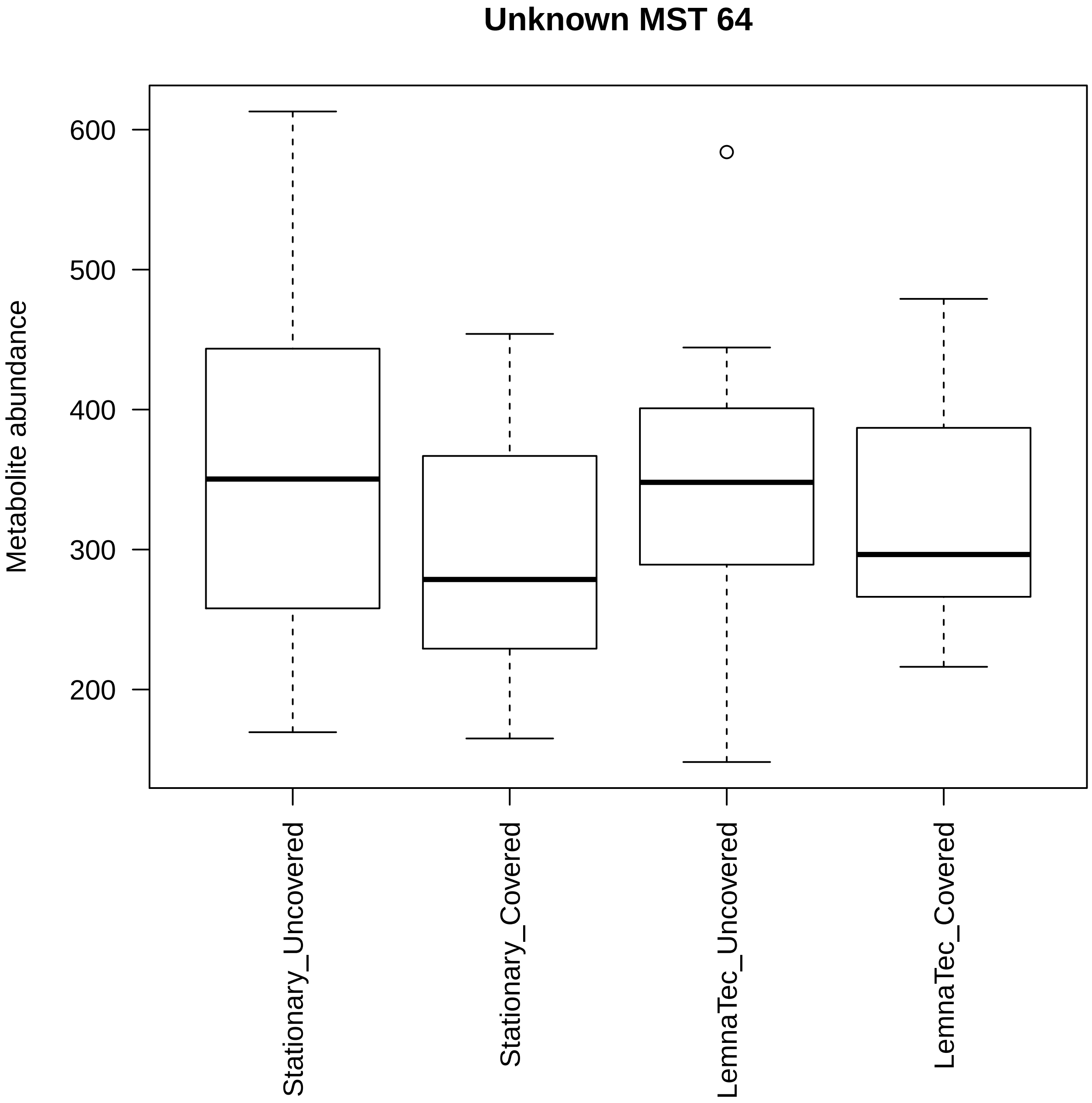
Unknown MST 64
Metabolite abundance
600
500
400
300
200
Stationary_Uncovered
Stationary_Covered
LemnaTec_Uncovered
LemnaTec_Covered

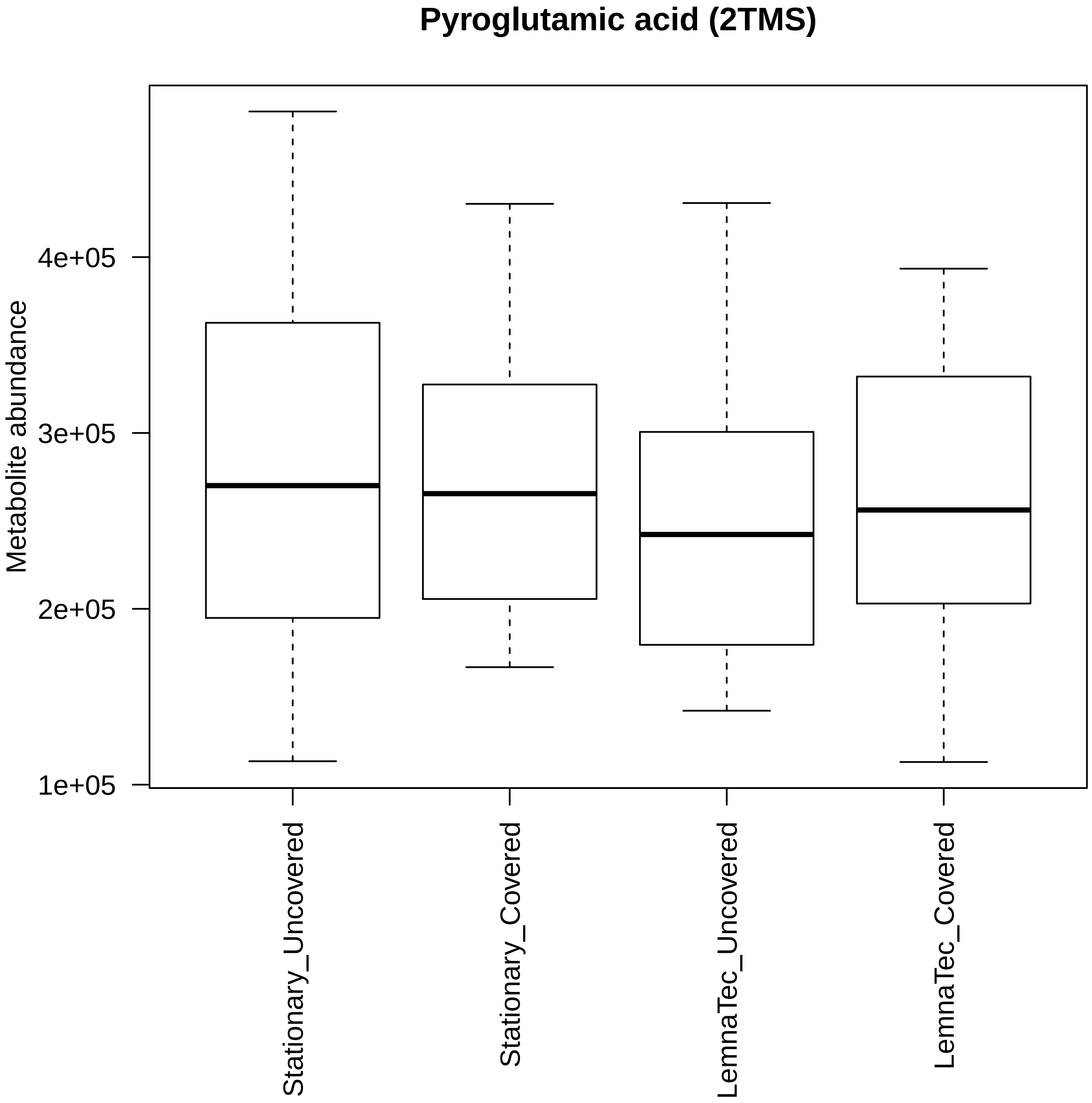
Pyroglutamic acid (2TMS)
Metabolite abundance
4e+05
3e+05
2e+05
1e+05
Stationary_Uncovered
Stationary_Covered
LemnaTec_Uncovered
LemnaTec_Covered

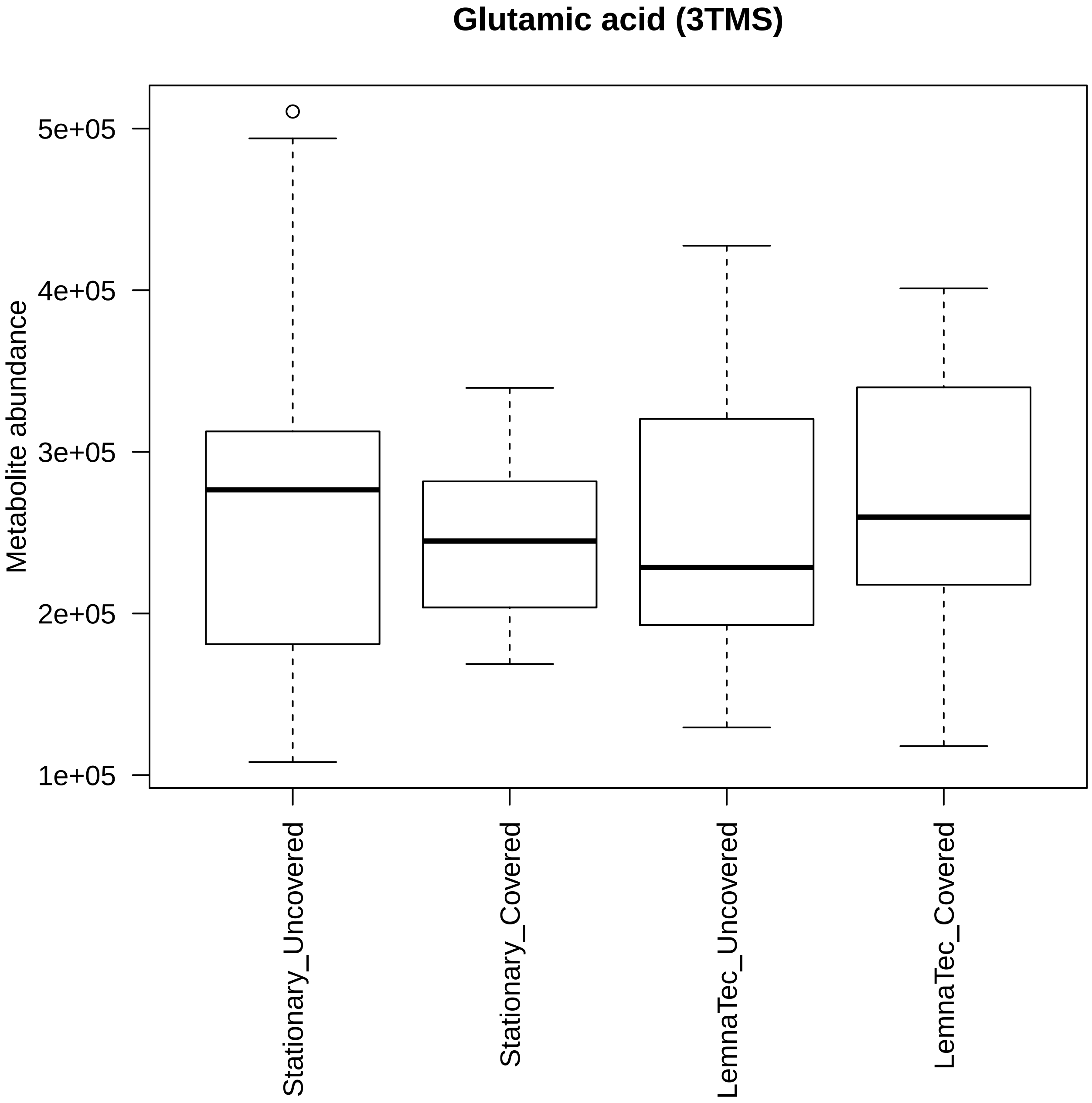

Glutamic acid (3TMS)
Metabolite abundance
5e+05
4e+05
3e+05
2e+05
1e+05
Stationary_Uncovered
Stationary_Covered
LemnaTec_Uncovered
LemnaTec_Covered

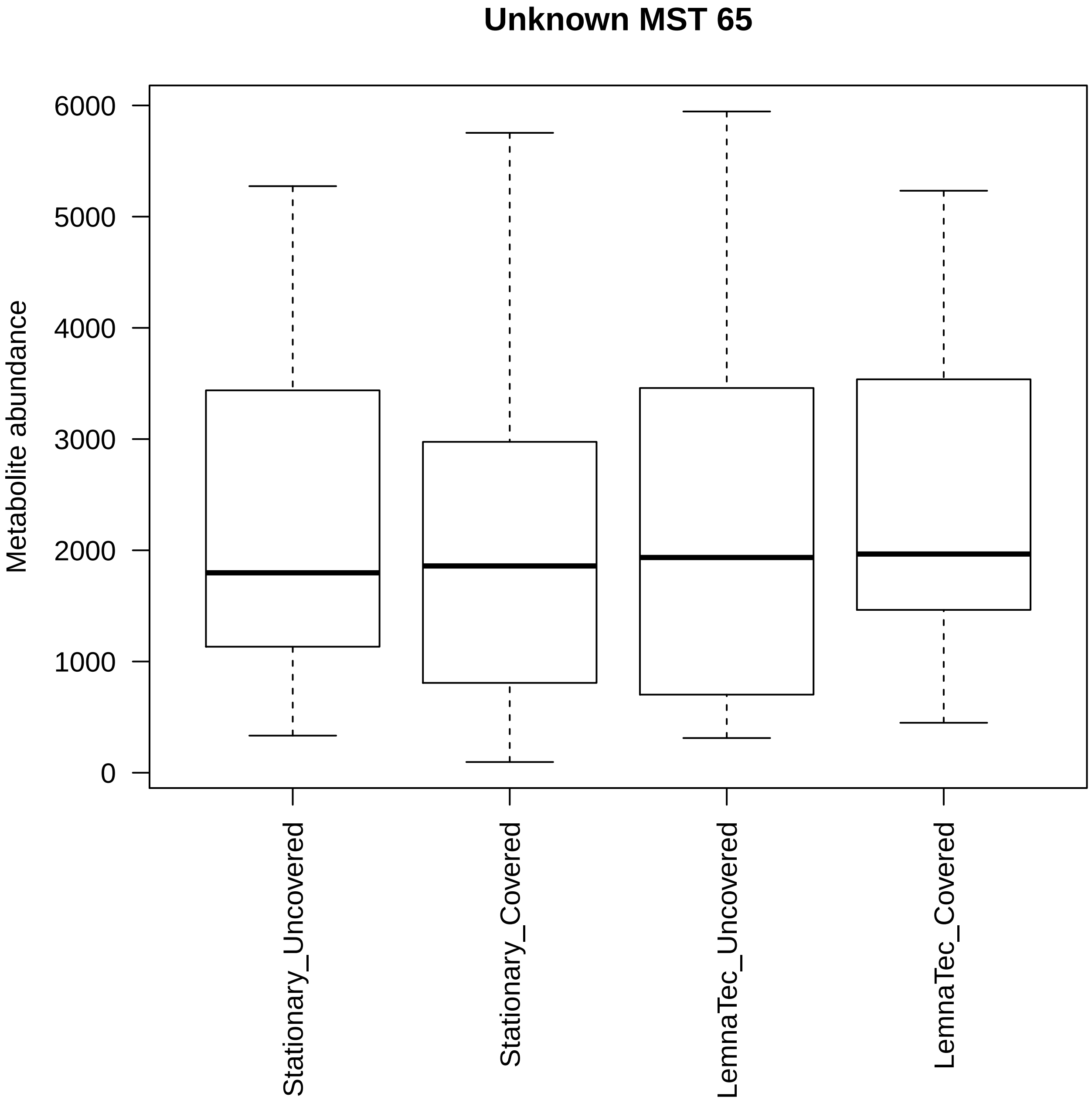

Unknown MST 65
Metabolite abundance
0
1000
2000
3000
4000
5000
6000
Stationary_Uncovered
Stationary_Covered
LemnaTec_Uncovered
LemnaTec_Covered

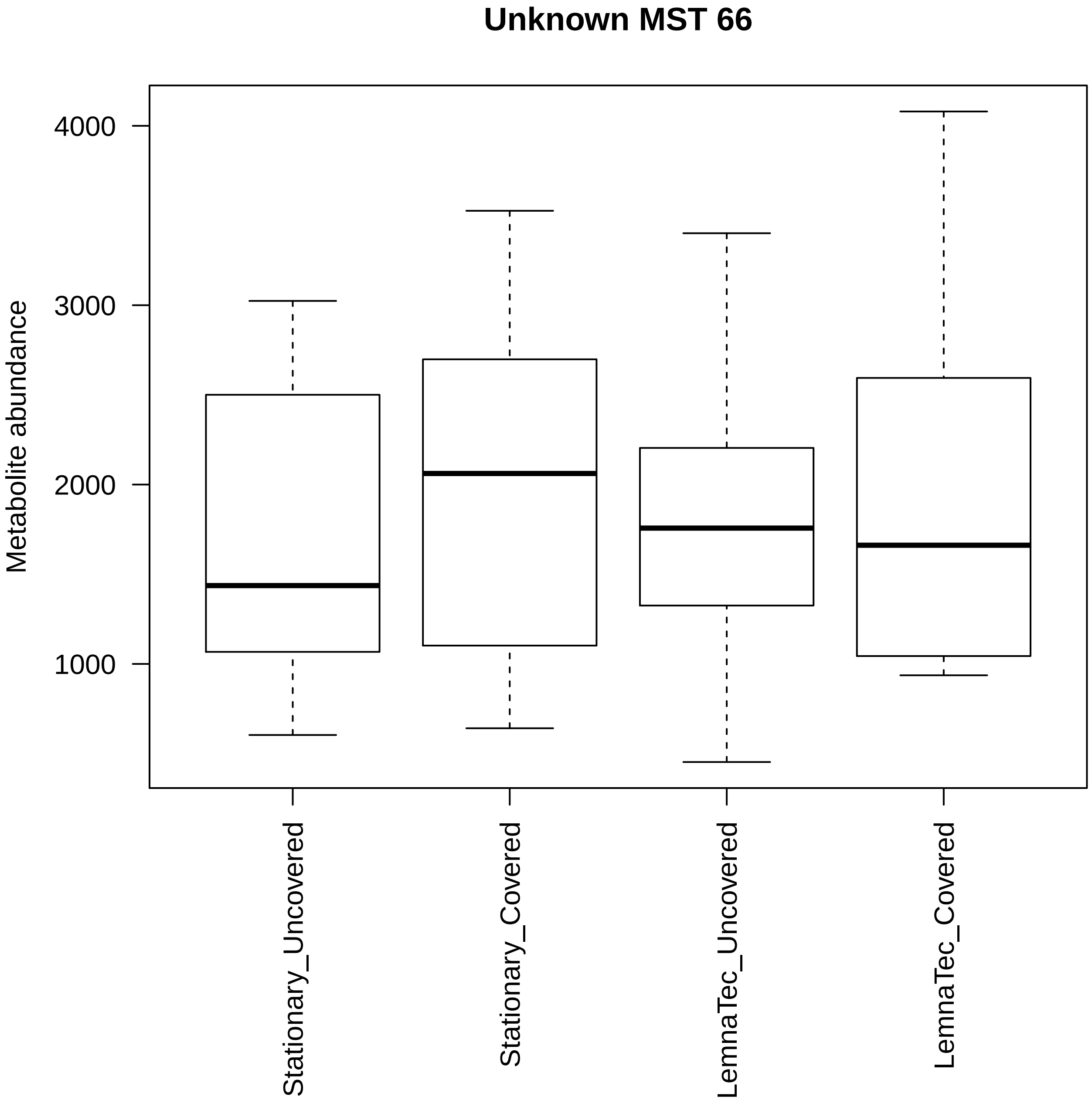
Unknown MST 66
Metabolite abundance
4000
3000
2000
1000
Stationary_Uncovered
Stationary_Covered
LemnaTec_Uncovered
LemnaTec_Covered

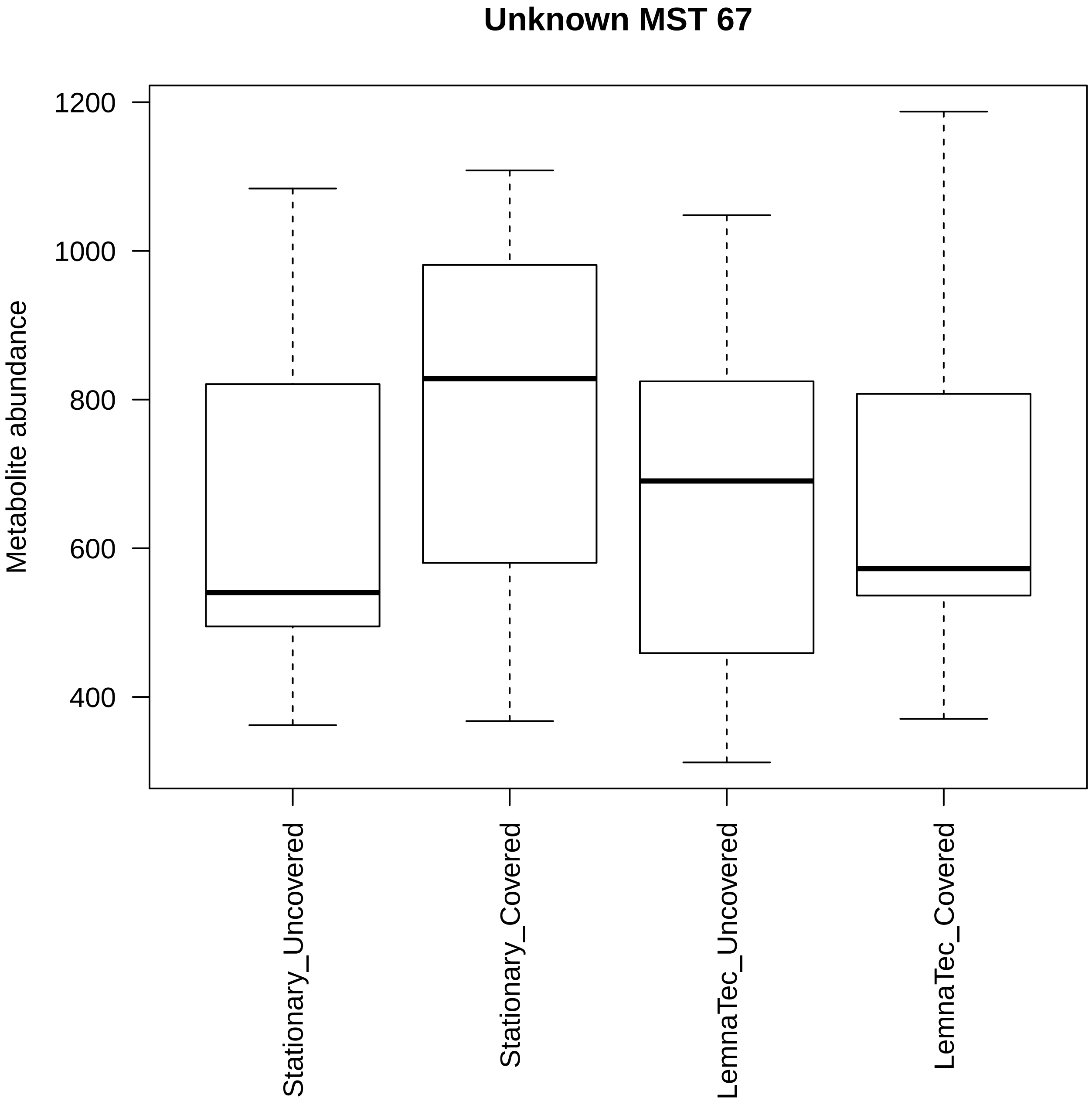
Unknown MST 67
Metabolite abundance
1200
1000
800
600
400
Stationary_Uncovered
Stationary_Covered
LemnaTec_Uncovered
LemnaTec_Covered

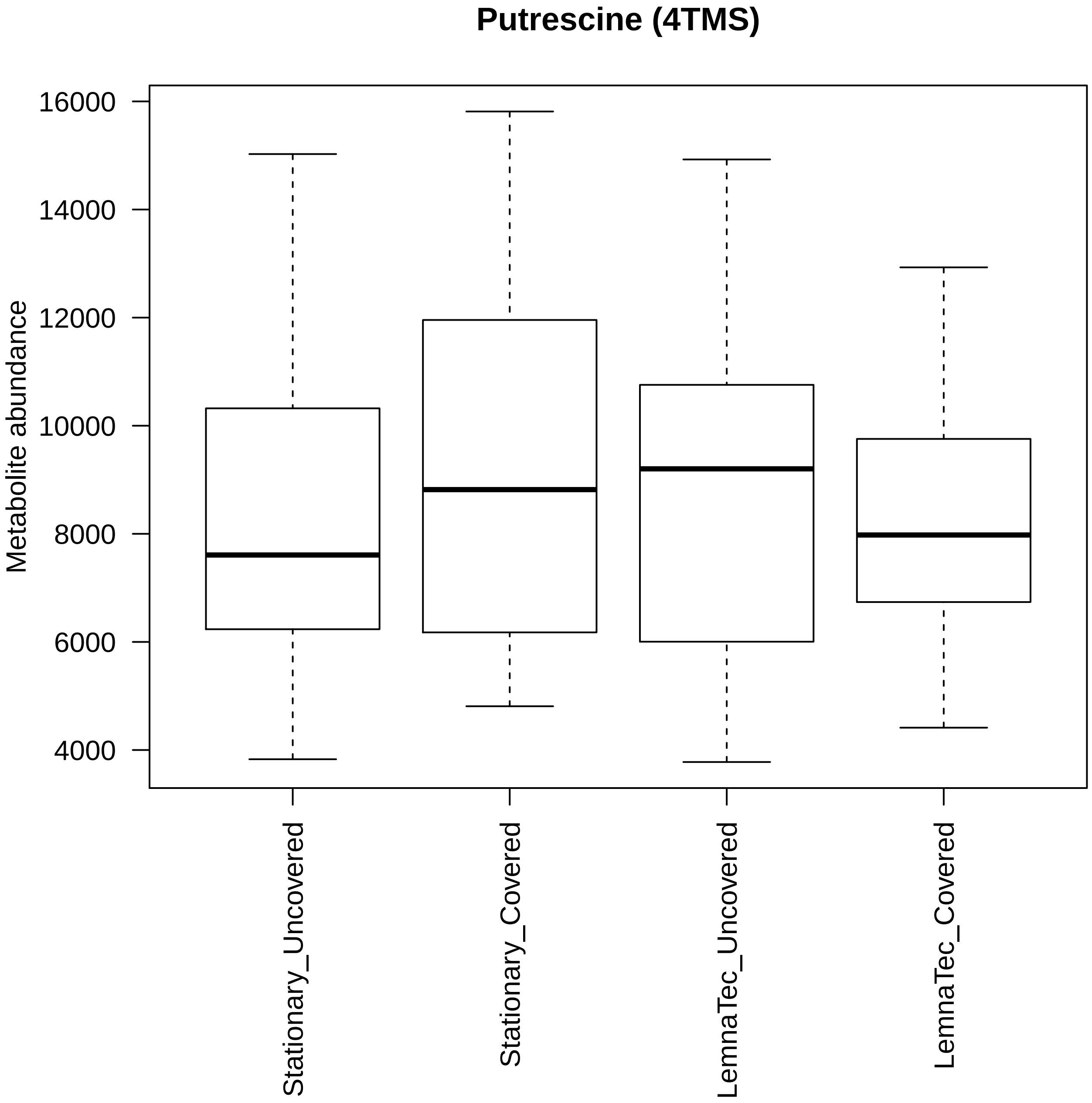

Putrescine (4TMS)
Metabolite abundance
16000
14000
12000
10000
8000
6000
4000
Stationary_Uncovered
Stationary_Covered
LemnaTec_Uncovered
LemnaTec_Covered

Unknown MST 68

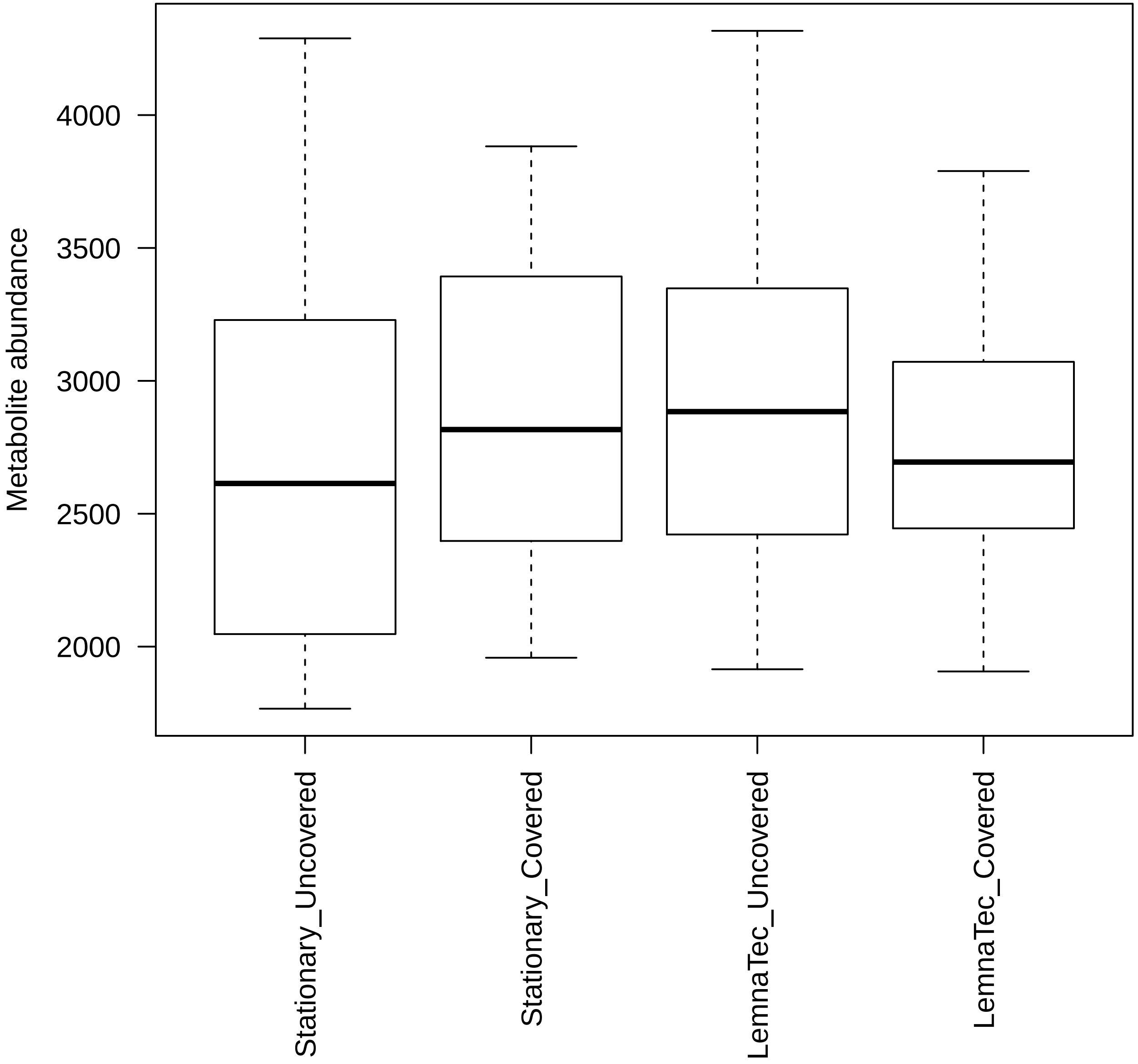

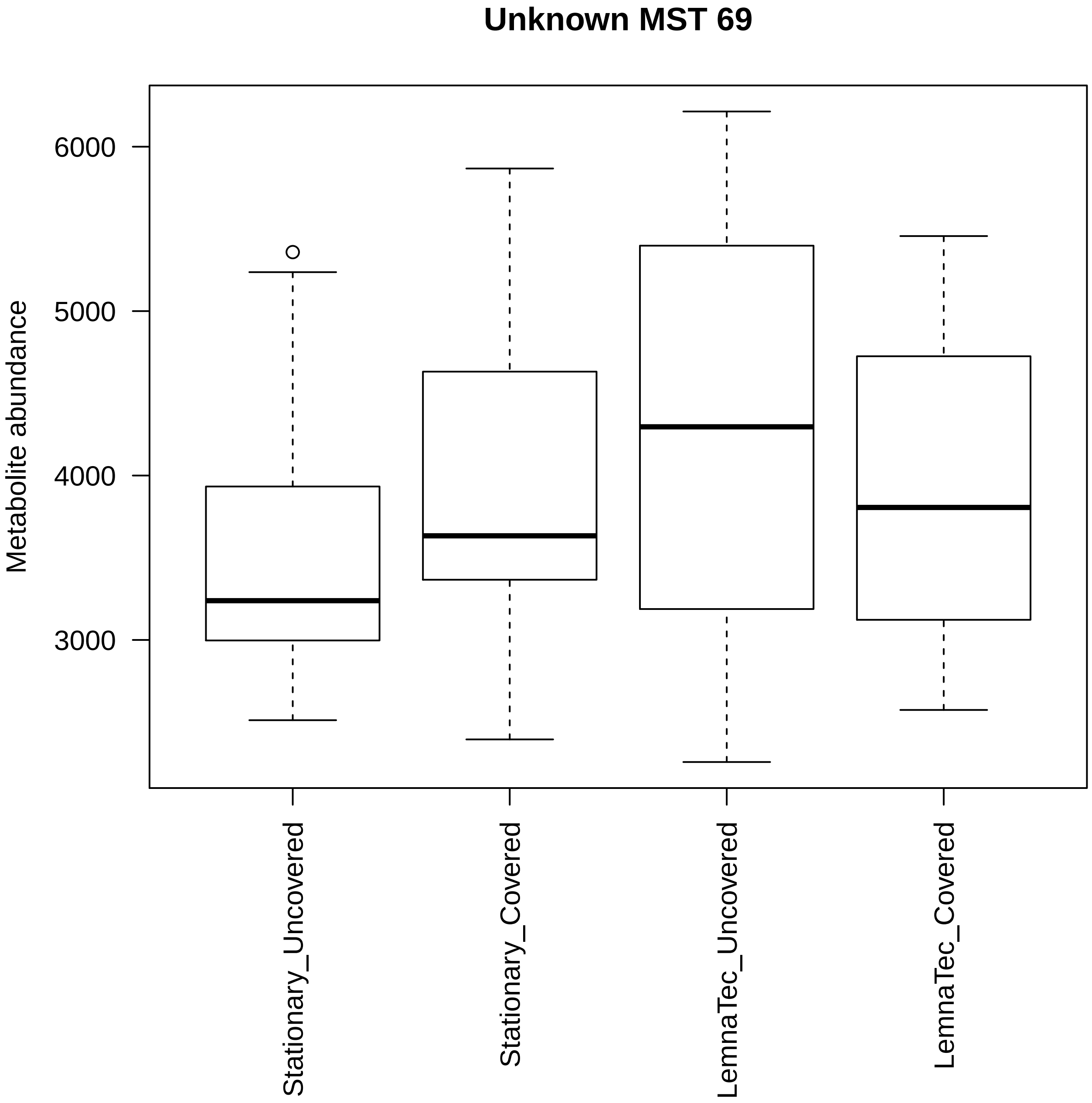
Unknown MST 69
Metabolite abundance
6000
5000
4000
3000
Stationary_Uncovered
Stationary_Covered
LemnaTec_Uncovered
LemnaTec_Covered

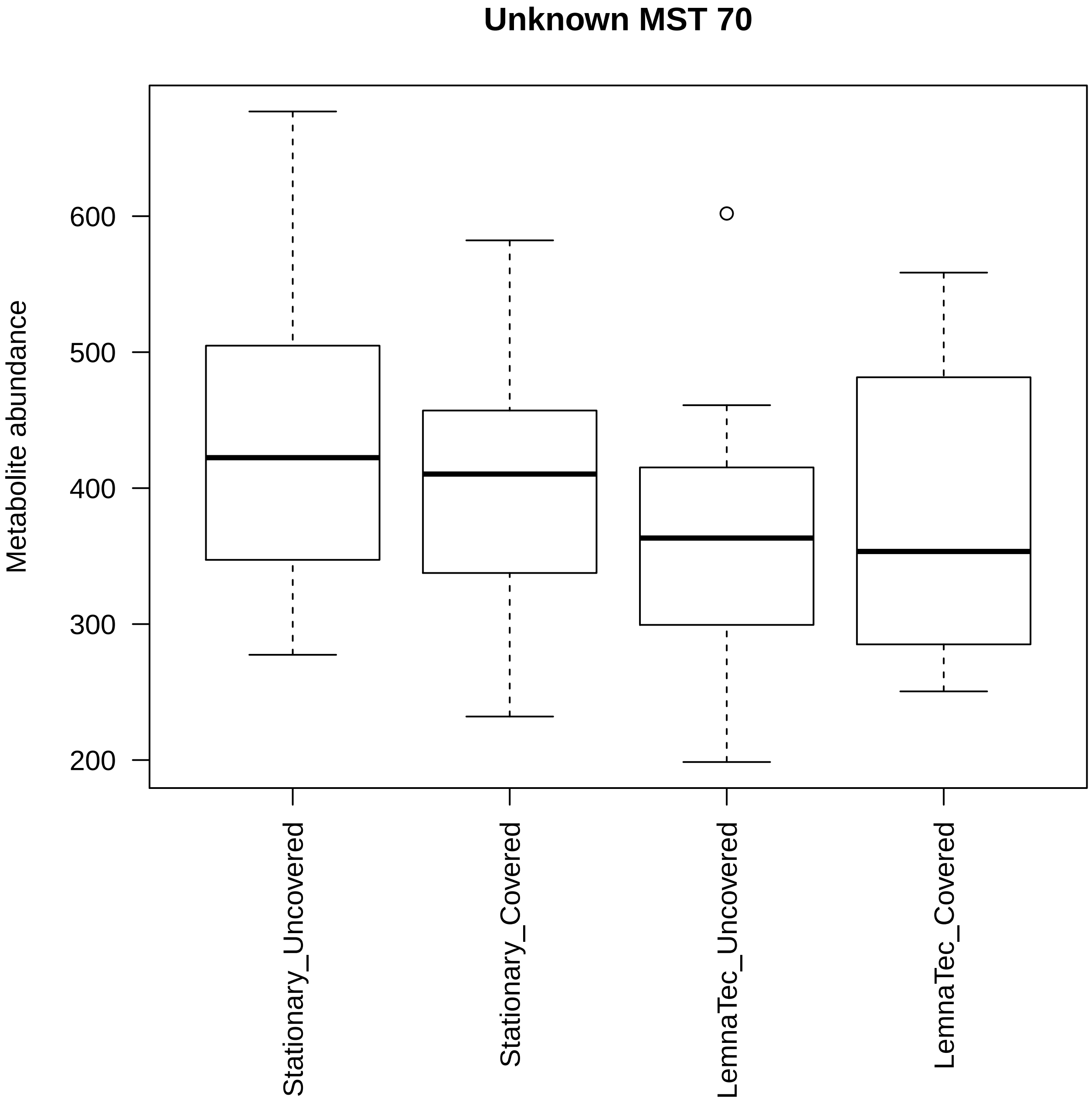

Unknown MST 70
Metabolite abundance
600
500
400
300
200
Stationary_Uncovered
Stationary_Covered
LemnaTec_Uncovered
LemnaTec_Covered

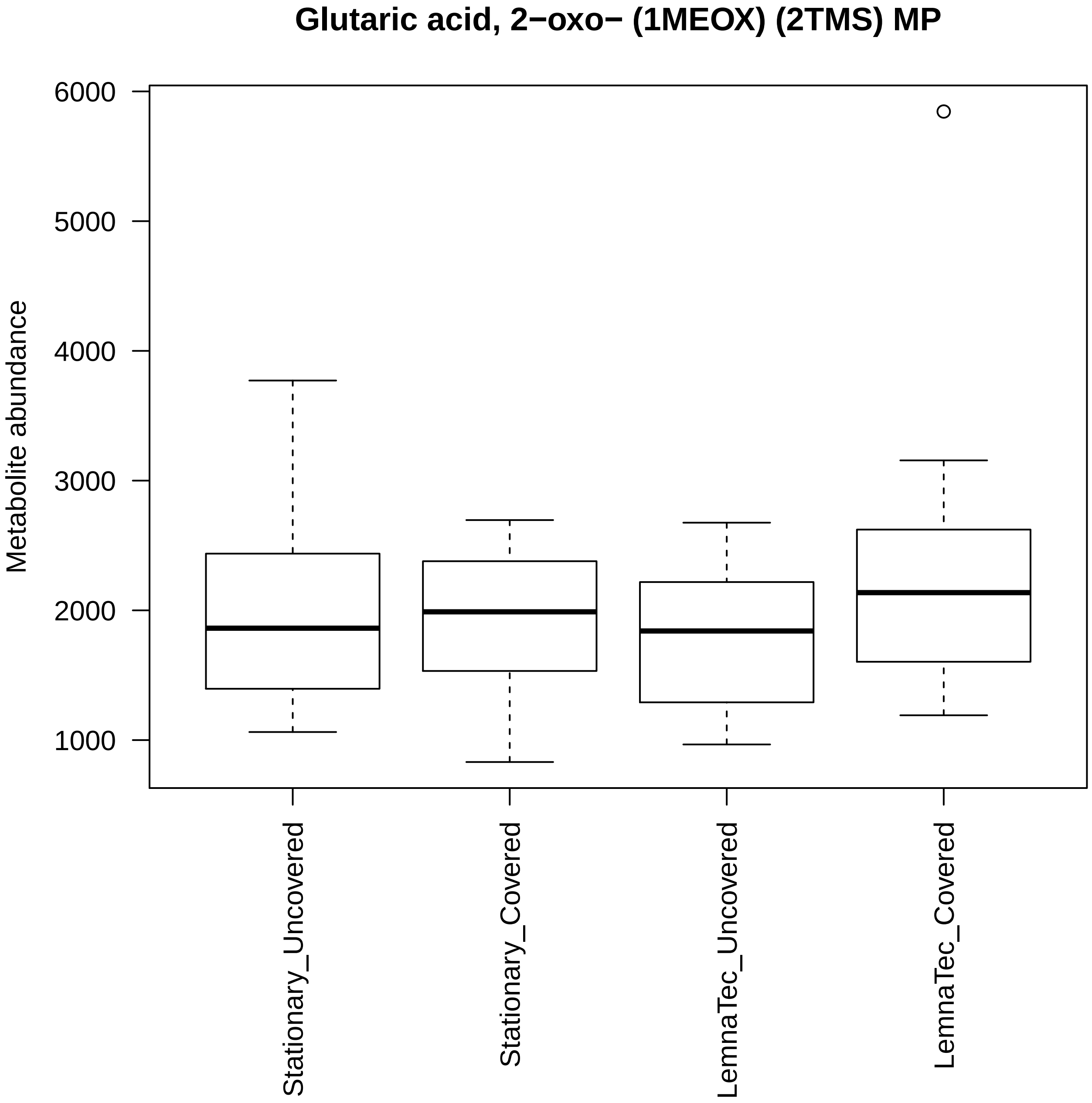
Glutaric acid, 2−oxo− (1MEOX) (2TMS) MP
Metabolite abundance
1000
2000
3000
4000
5000
6000
Stationary_Uncovered
Stationary_Covered
LemnaTec_Uncovered
LemnaTec_Covered

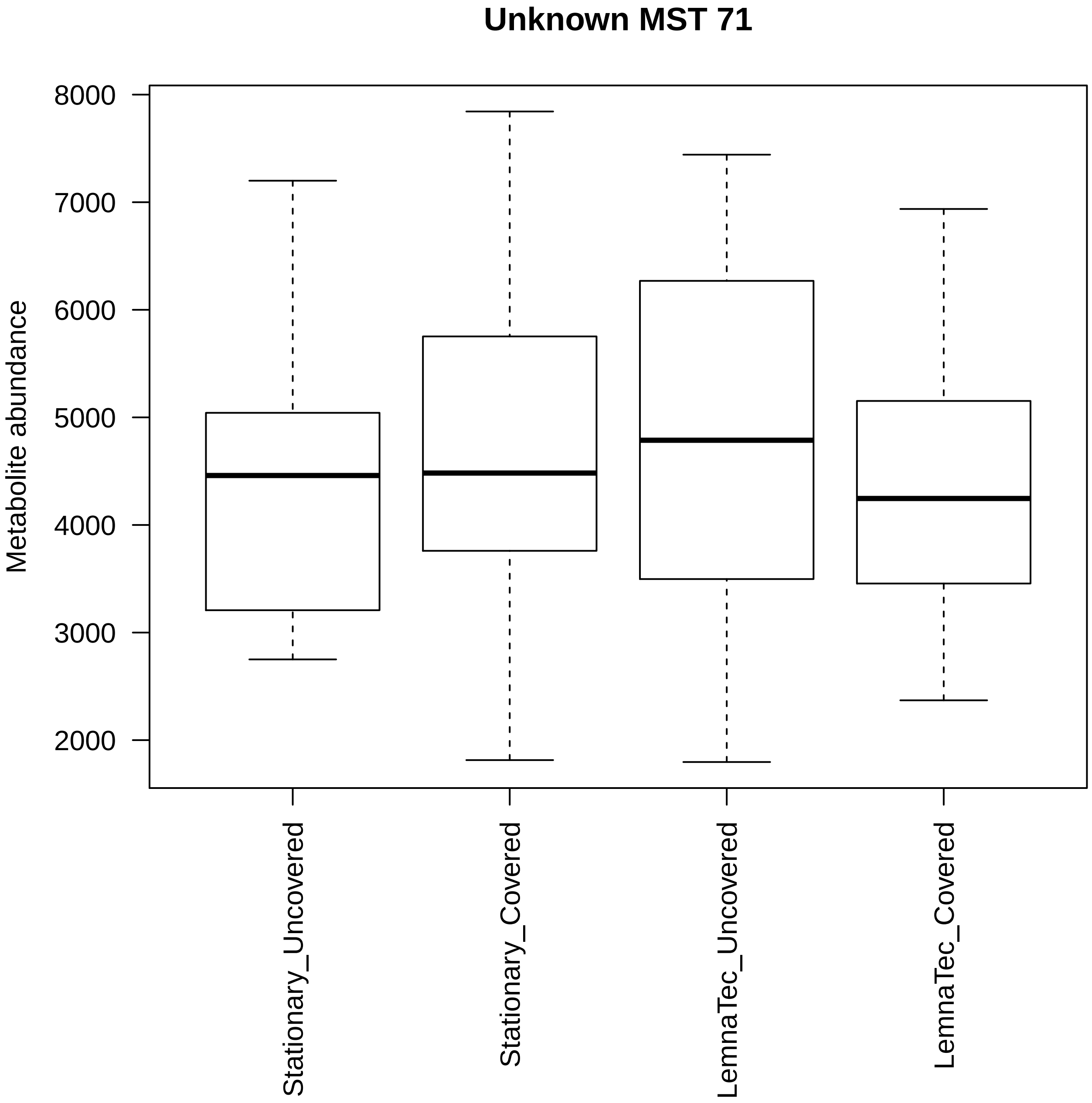

Unknown MST 71
Metabolite abundance
8000
7000
6000
5000
4000
3000
2000
Stationary_Uncovered
Stationary_Covered
LemnaTec_Uncovered
LemnaTec_Covered

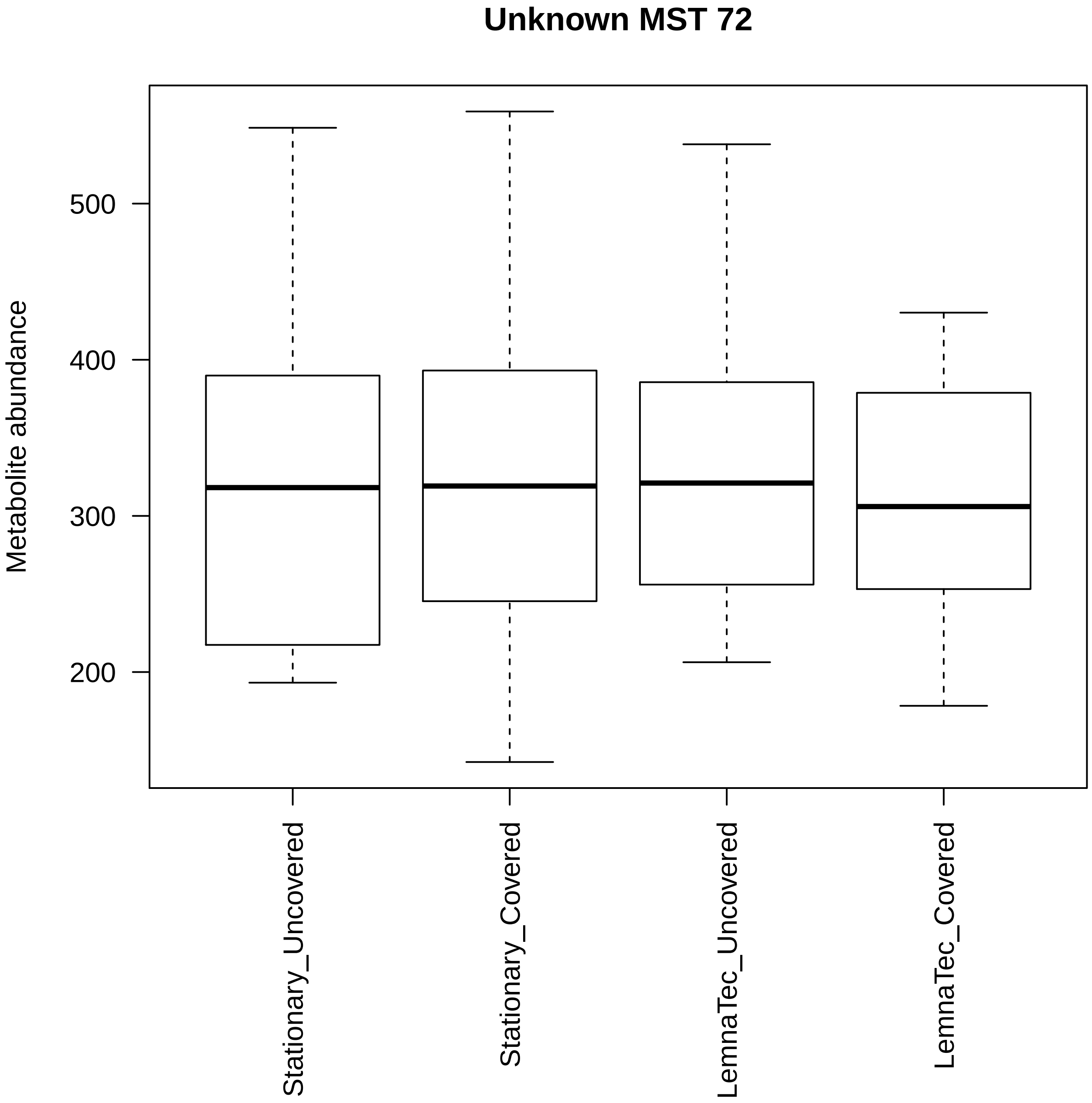

Unknown MST 72
Metabolite abundance
500
400
300
200
Stationary_Uncovered
Stationary_Covered
LemnaTec_Uncovered
LemnaTec_Covered

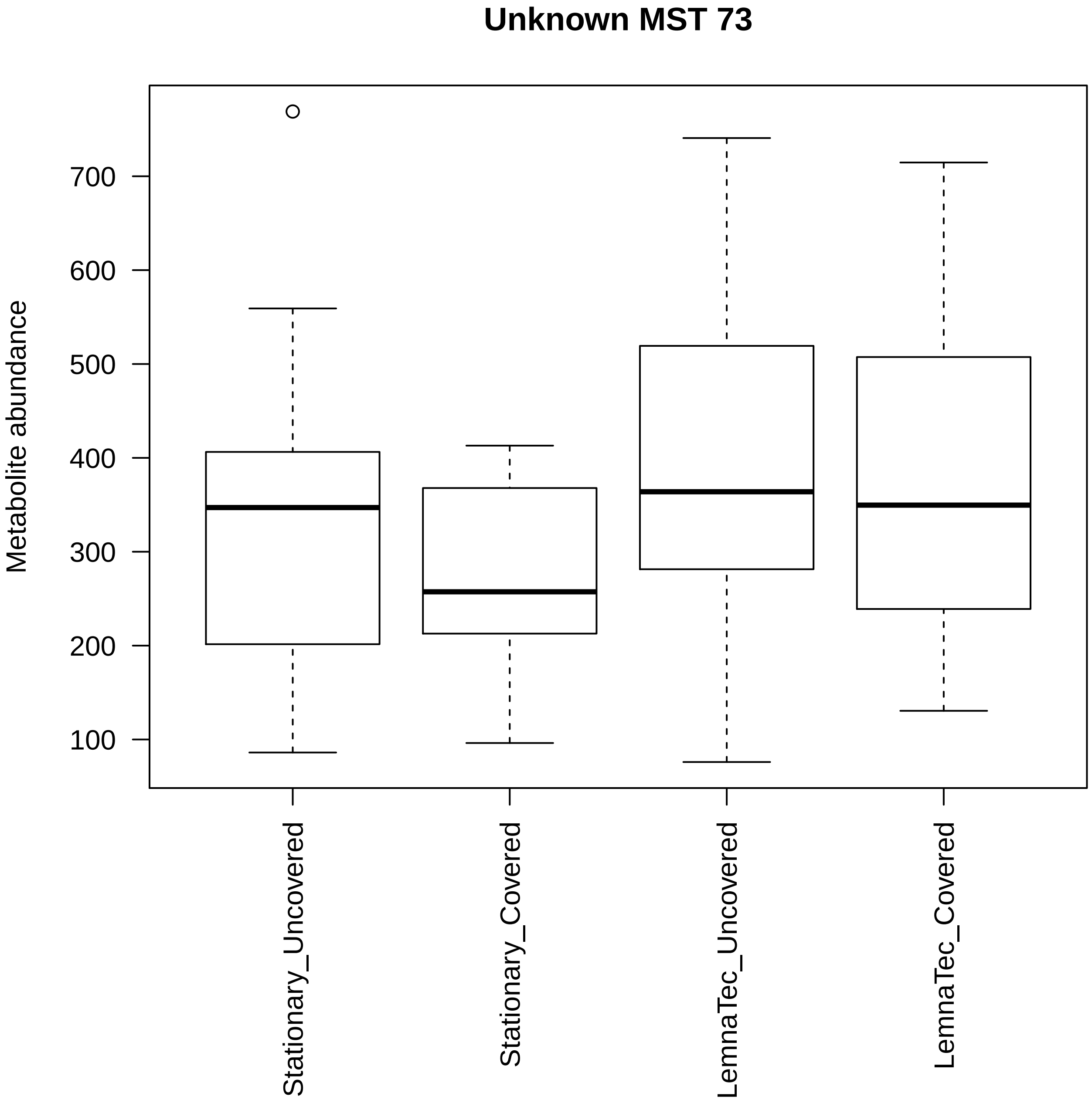

Unknown MST 73
Metabolite abundance
100
200
300
400
500
600
700
Stationary_Uncovered
Stationary_Covered
LemnaTec_Uncovered
LemnaTec_Covered

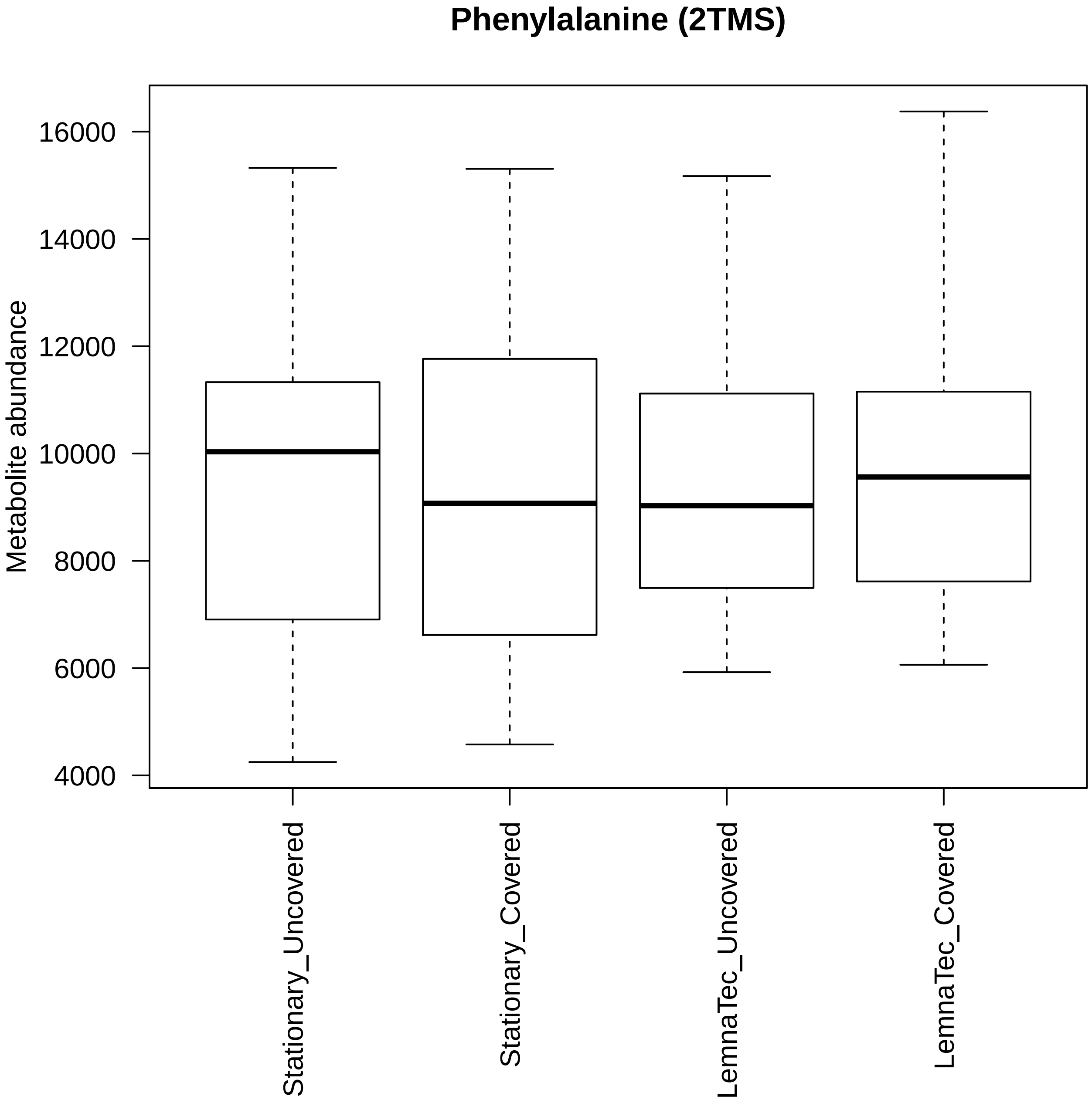
Phenylalanine (2TMS)
Metabolite abundance
16000
14000
12000
10000
8000
6000
4000
Stationary_Uncovered
Stationary_Covered
LemnaTec_Uncovered
LemnaTec_Covered

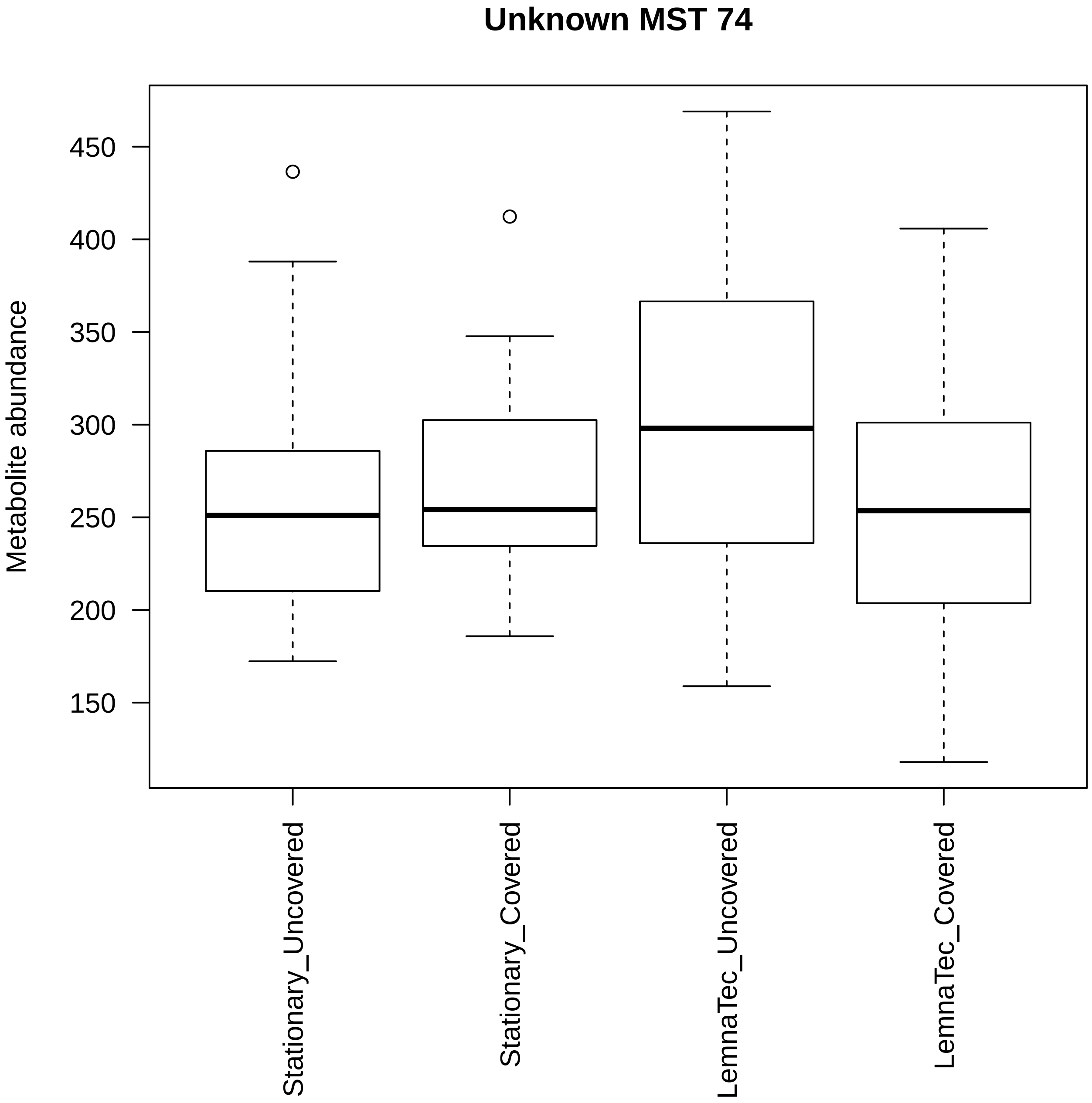
Unknown MST 74
Metabolite abundance
450
400
350
300
250
200
150
Stationary_Uncovered
Stationary_Covered
LemnaTec_Uncovered
LemnaTec_Covered

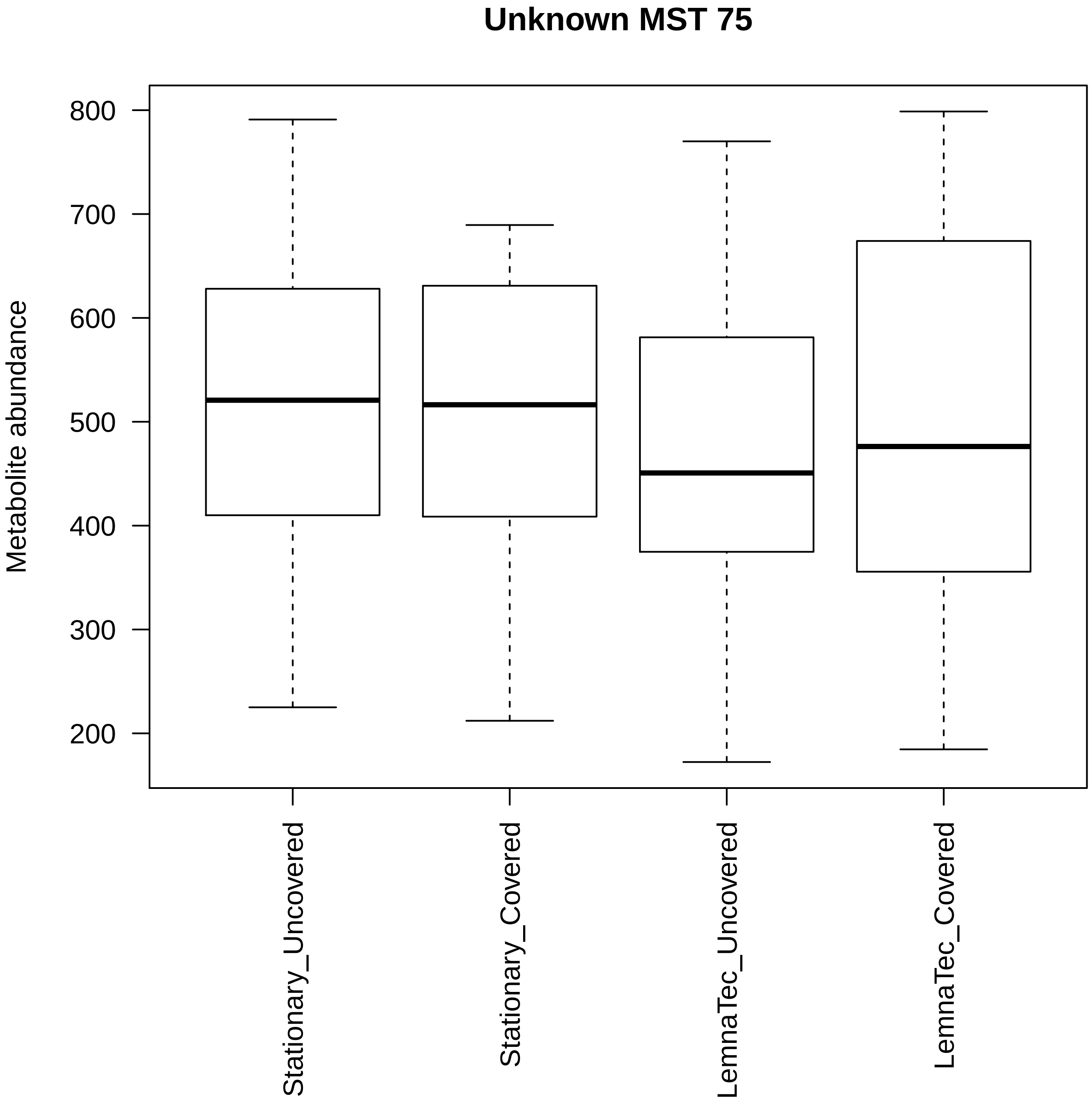
Unknown MST 75
Metabolite abundance
800
700
600
500
400
300
200
Stationary_Uncovered
Stationary_Covered
LemnaTec_Uncovered
LemnaTec_Covered

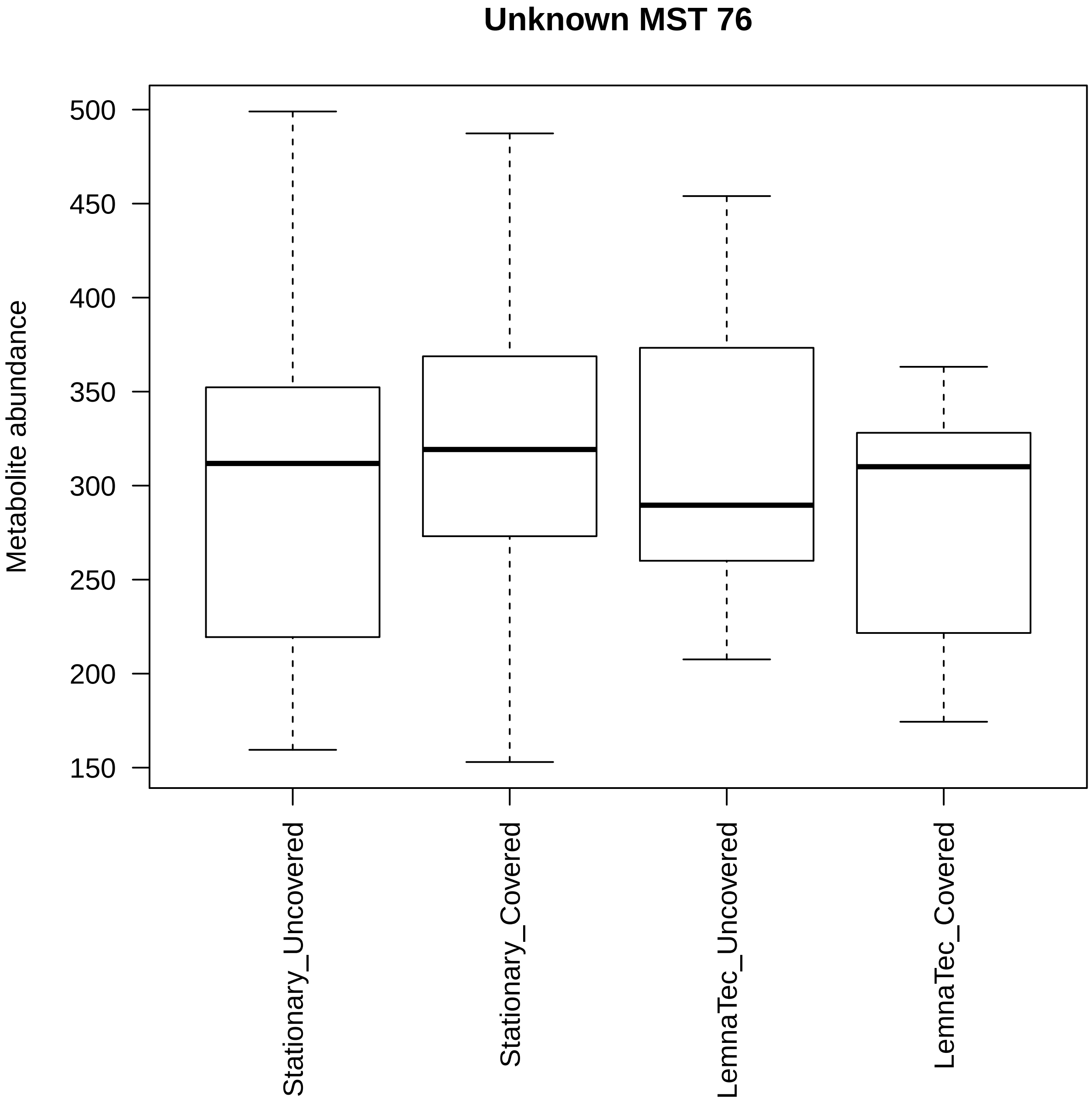

Unknown MST 76
Metabolite abundance
500
450
400
350
300
250
200
150
Stationary_Uncovered
Stationary_Covered
LemnaTec_Uncovered
LemnaTec_Covered

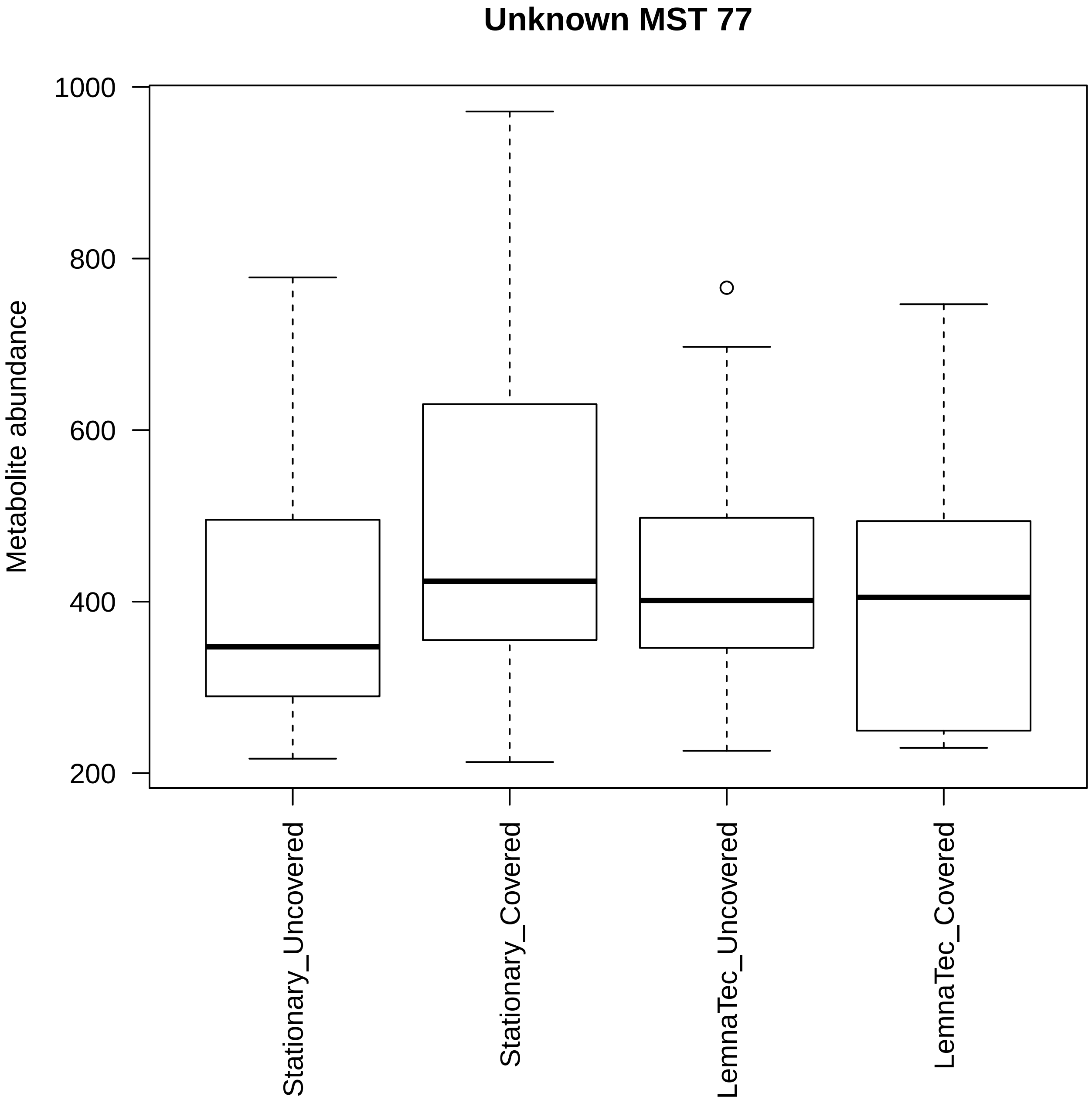

Unknown MST 77
Metabolite abundance
1000
800
600
400
200
Stationary_Uncovered
Stationary_Covered
LemnaTec_Uncovered
LemnaTec_Covered

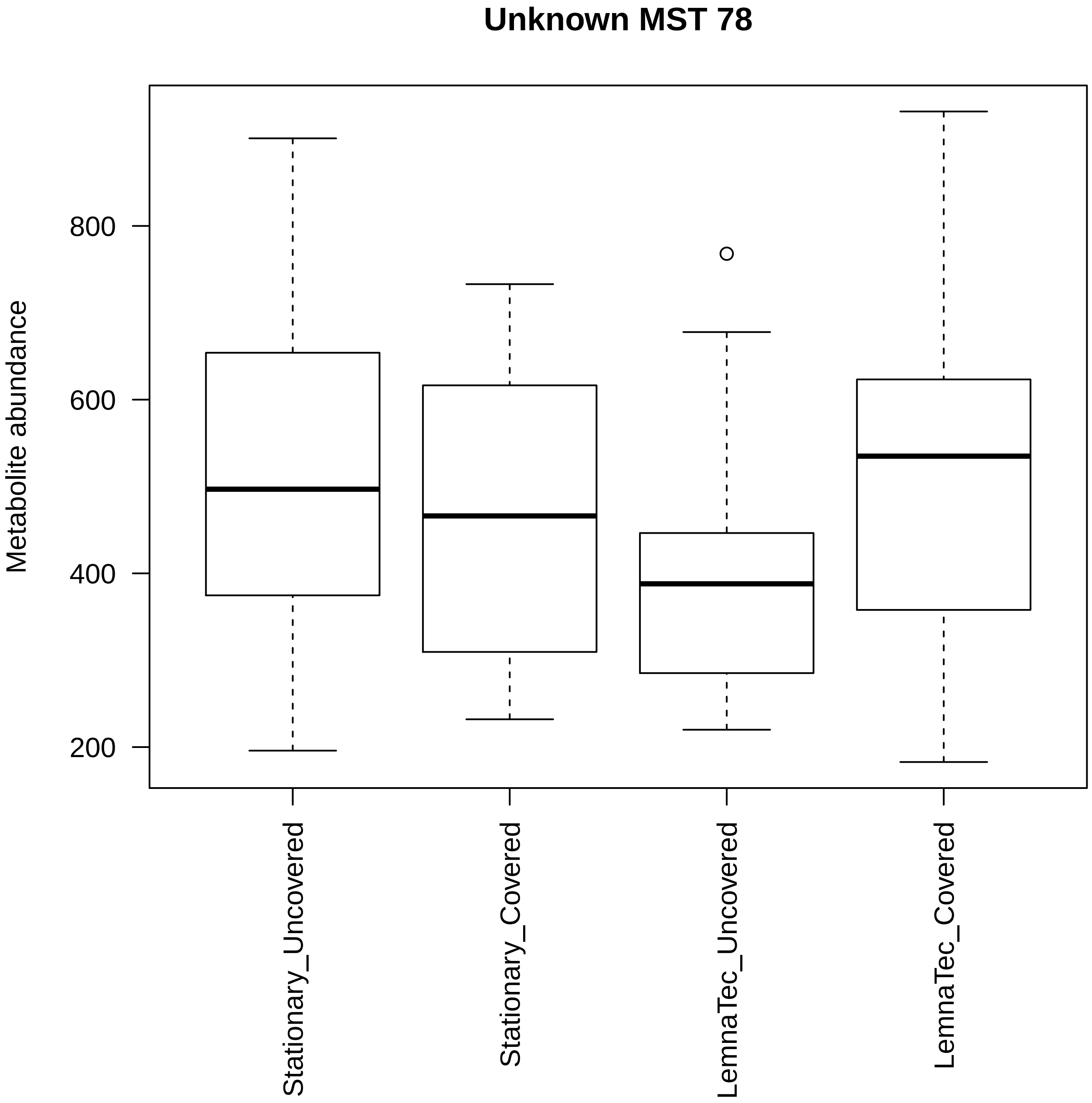

Unknown MST 78
Metabolite abundance
200
400
600
800
Stationary_Uncovered
Stationary_Covered
LemnaTec_Uncovered
LemnaTec_Covered

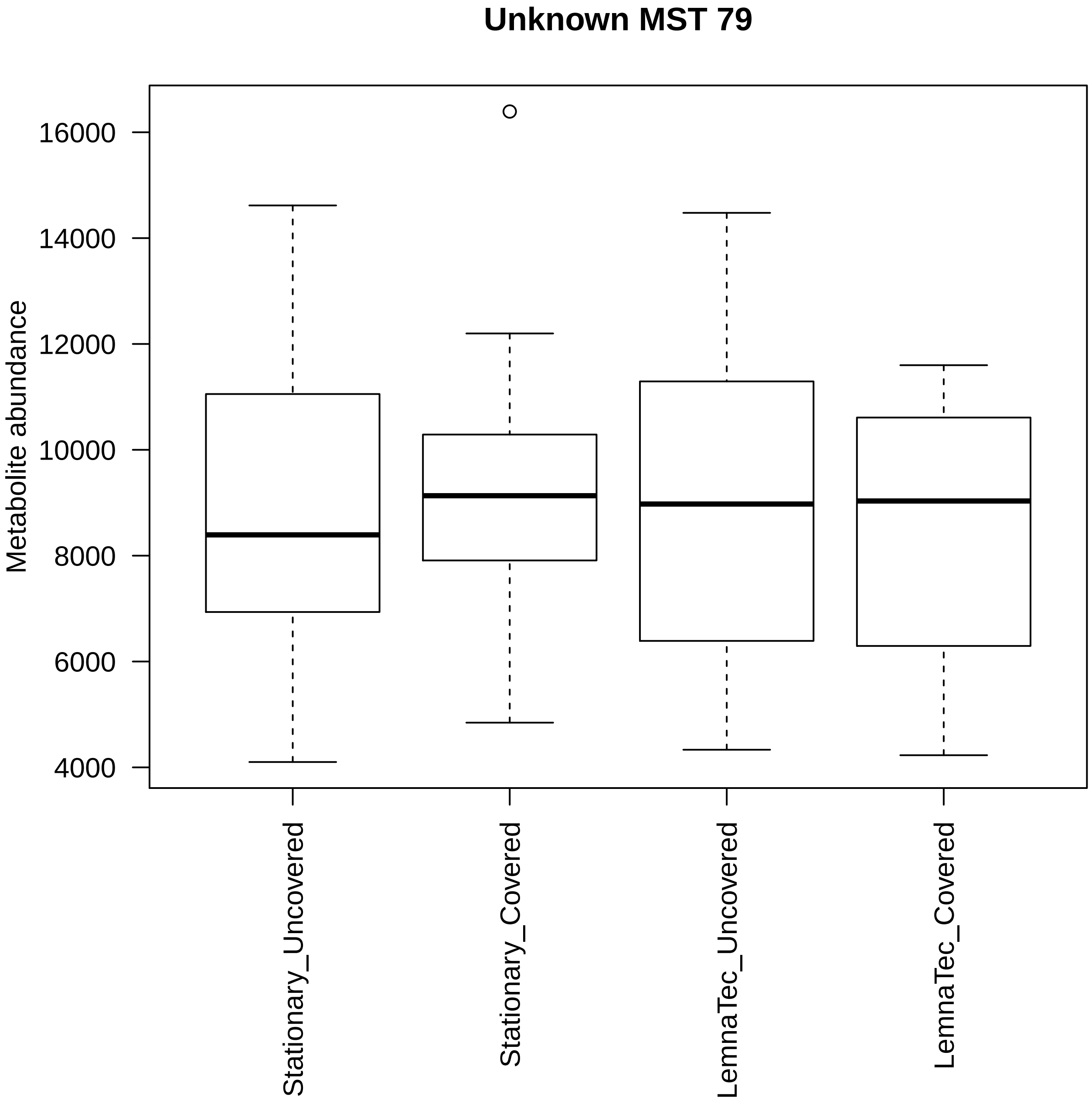

Unknown MST 79
Metabolite abundance
4000
6000
8000
10000
12000
14000
16000
Stationary_Uncovered
Stationary_Covered
LemnaTec_Uncovered
LemnaTec_Covered

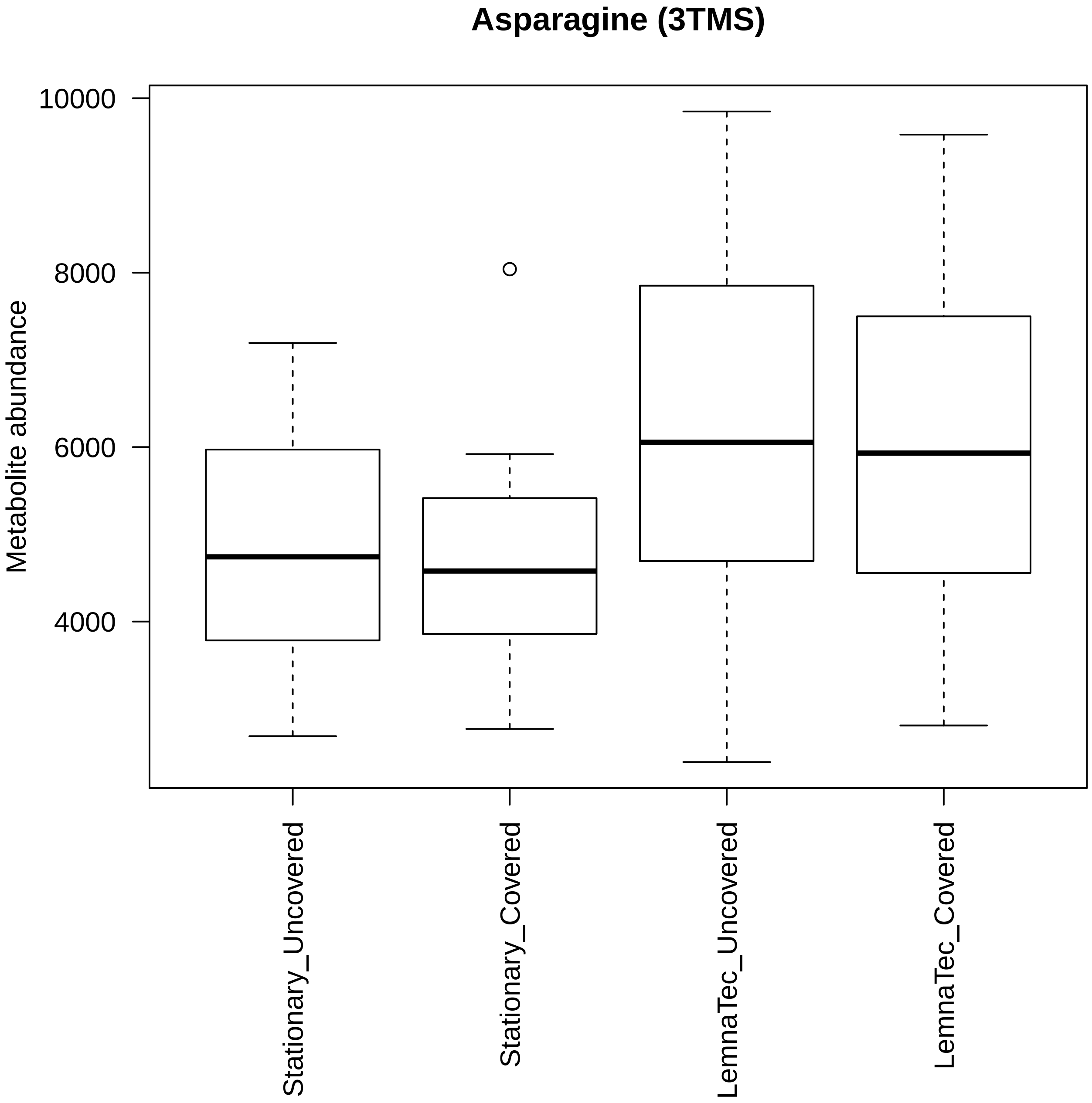
Asparagine (3TMS)
Metabolite abundance
10000
8000
6000
4000
Stationary_Uncovered
Stationary_Covered
LemnaTec_Uncovered
LemnaTec_Covered

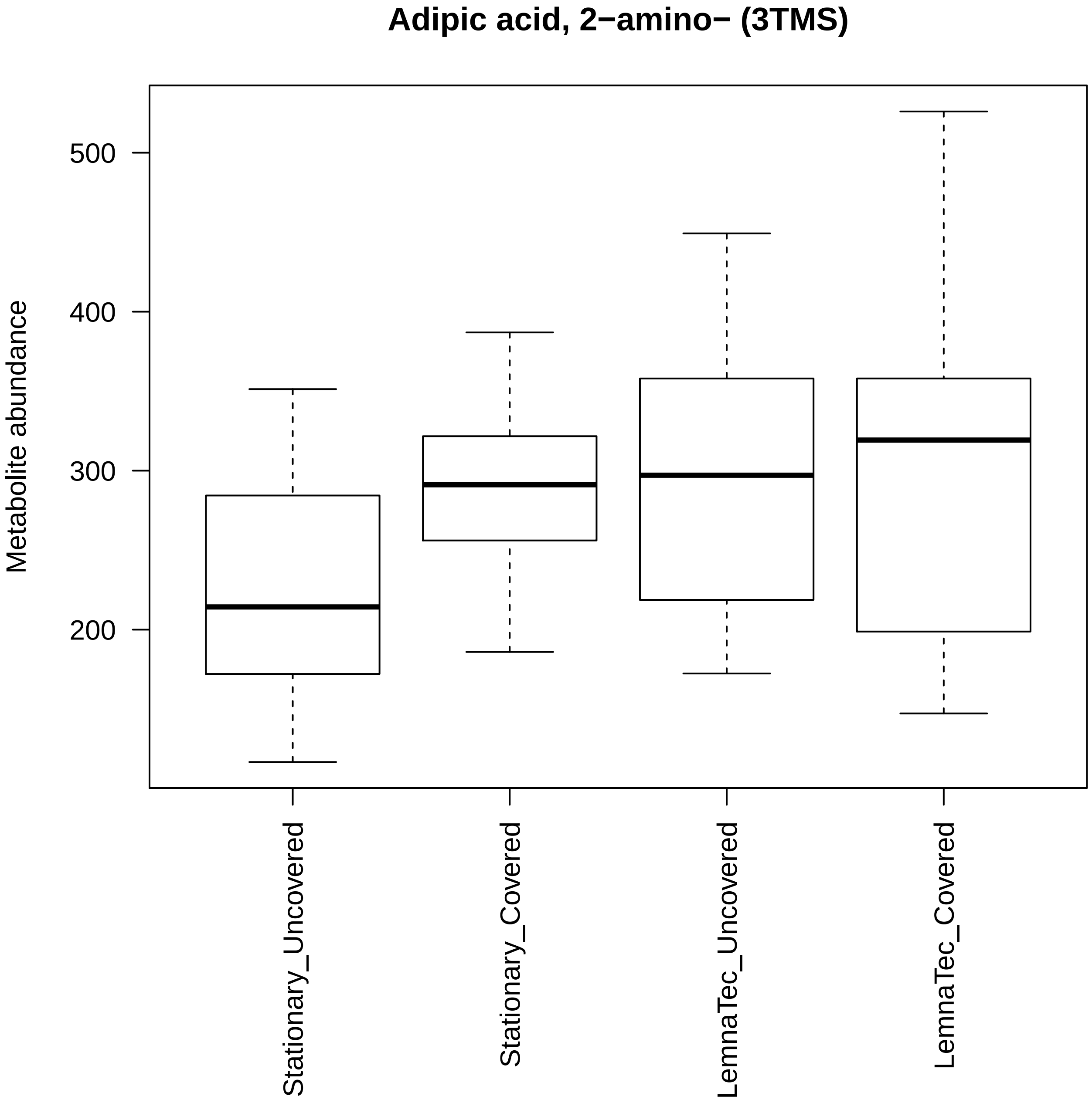

Adipic acid, 2−amino− (3TMS)
Metabolite abundance
500
400
300
200
Stationary_Uncovered
Stationary_Covered
LemnaTec_Uncovered
LemnaTec_Covered

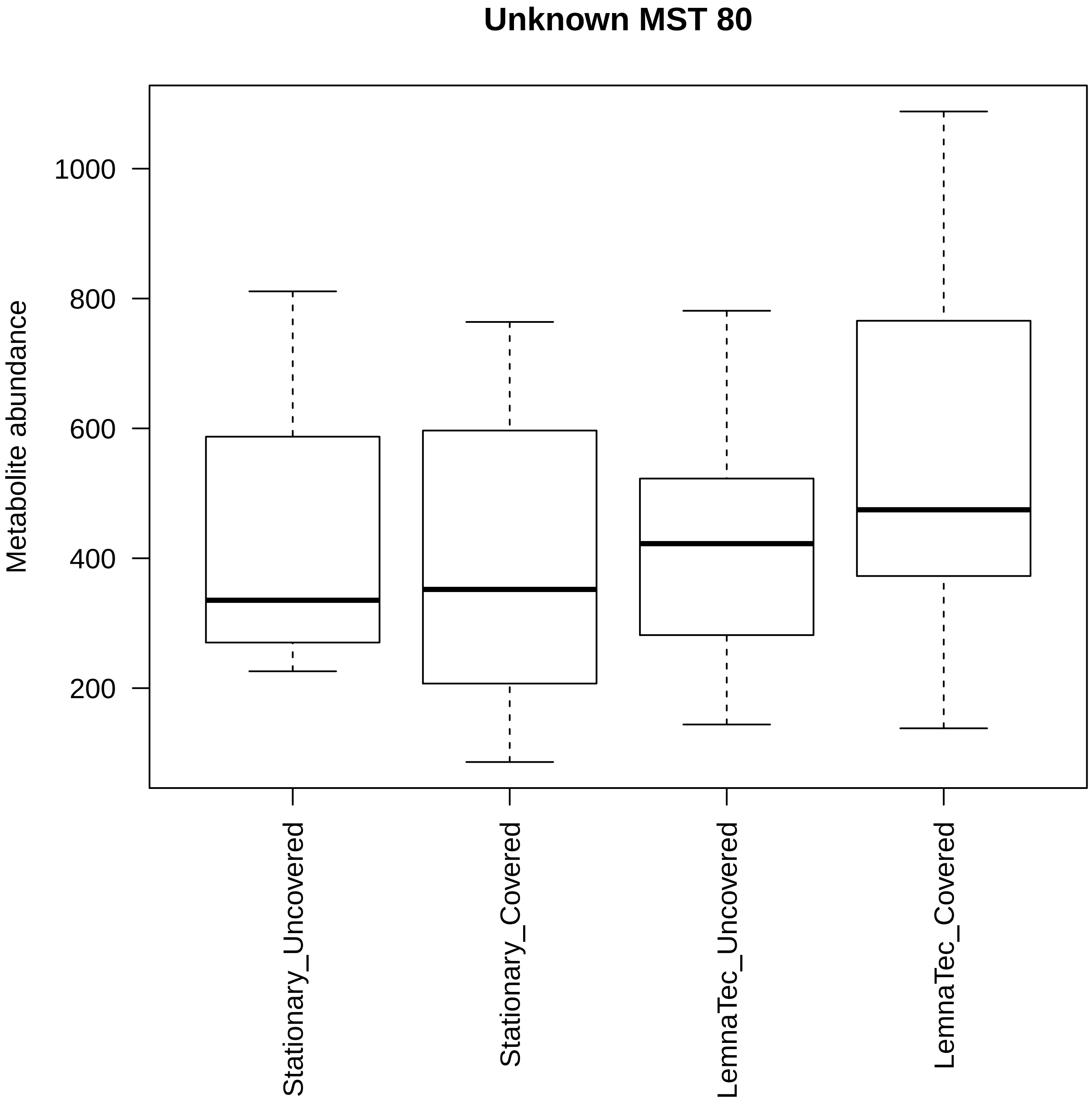

Unknown MST 80
Metabolite abundance
1000
800
600
400
200
Stationary_Uncovered
Stationary_Covered
LemnaTec_Uncovered
LemnaTec_Covered

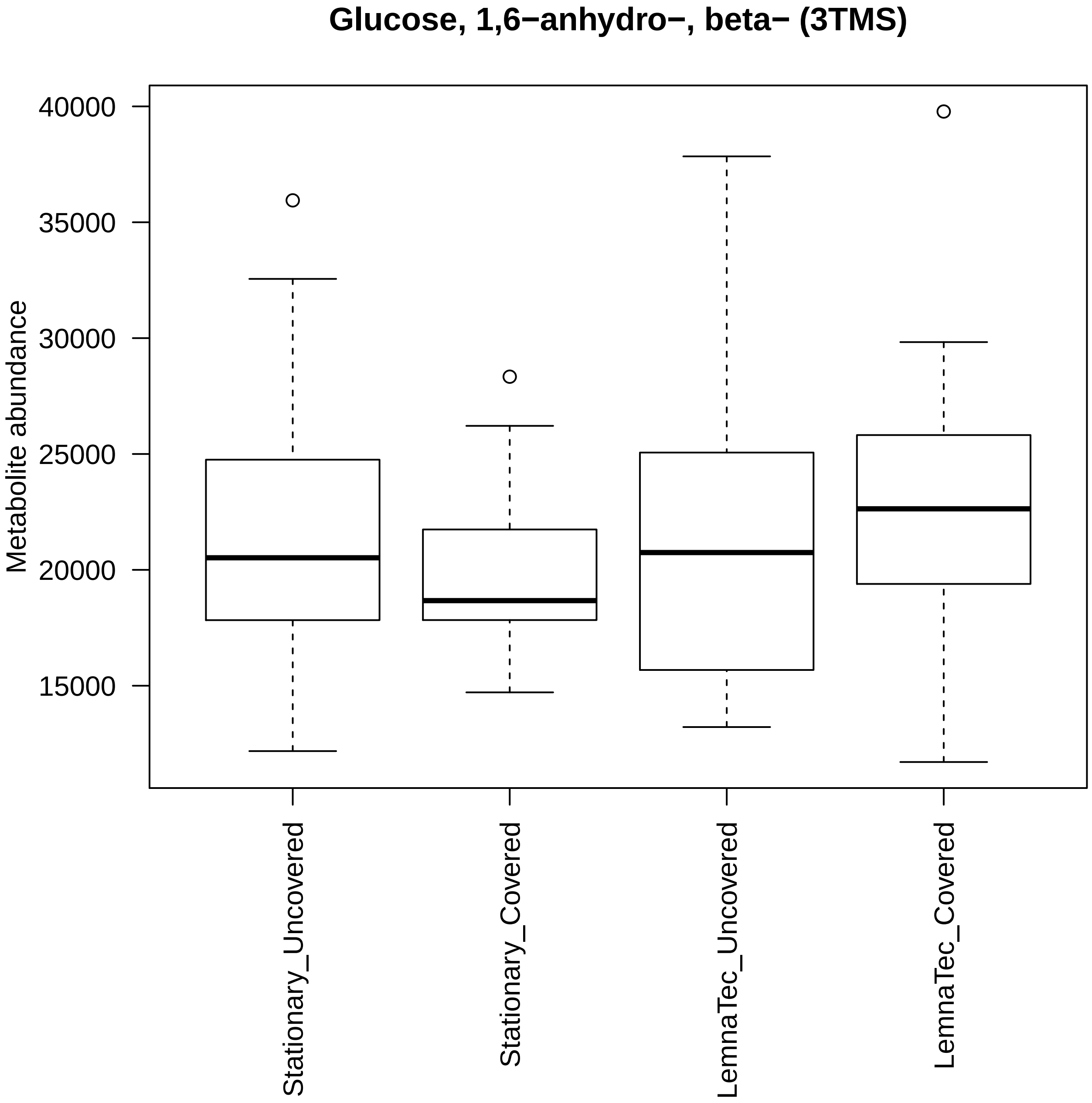

Glucose, 1,6−anhydro−, beta− (3TMS)
Metabolite abundance
40000
35000
30000
25000
20000
15000
Stationary_Uncovered
Stationary_Covered
LemnaTec_Uncovered
LemnaTec_Covered

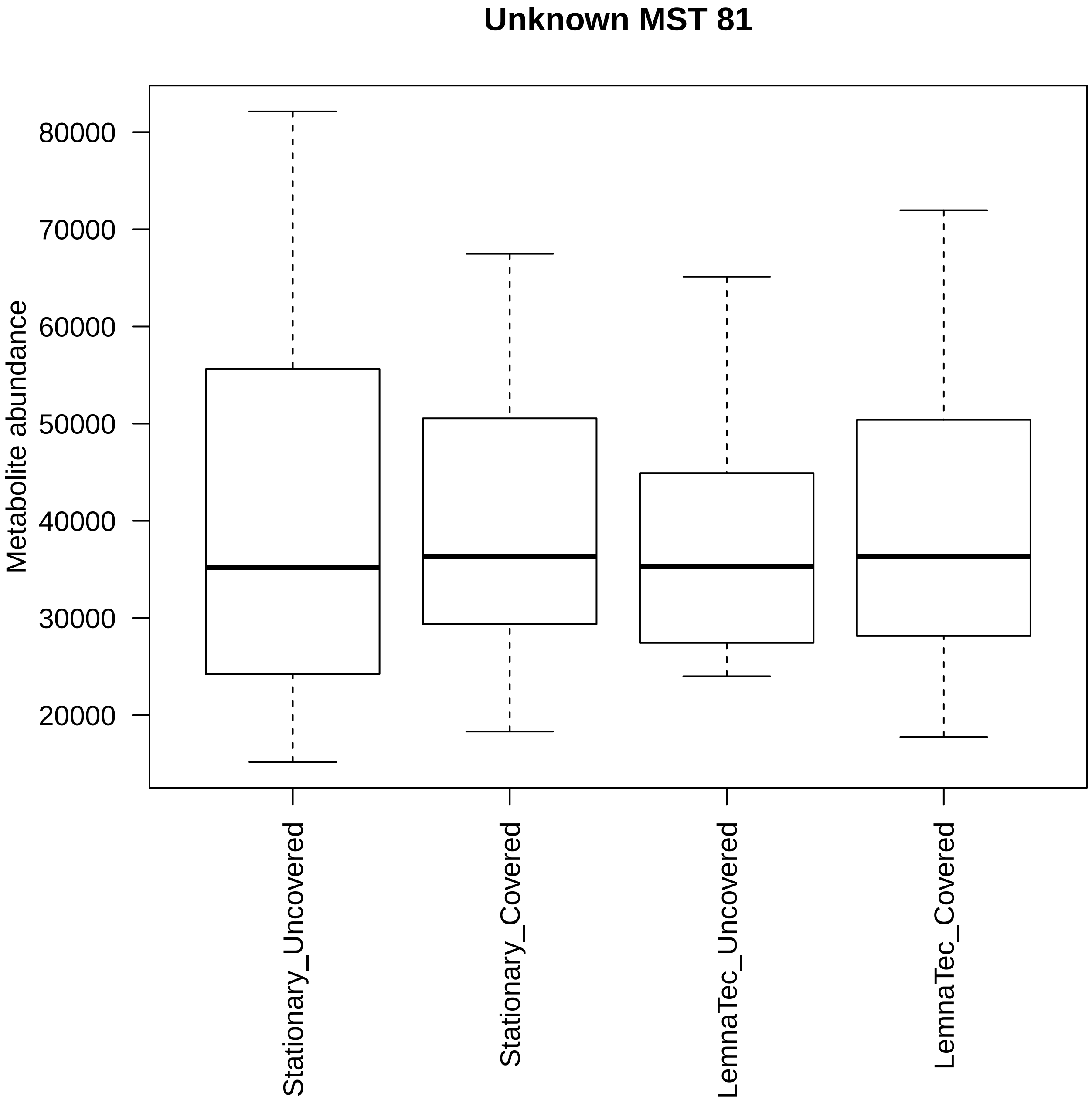

Unknown MST 81
Metabolite abundance
80000
70000
60000
50000
40000
30000
20000
Stationary_Uncovered
Stationary_Covered
LemnaTec_Uncovered
LemnaTec_Covered

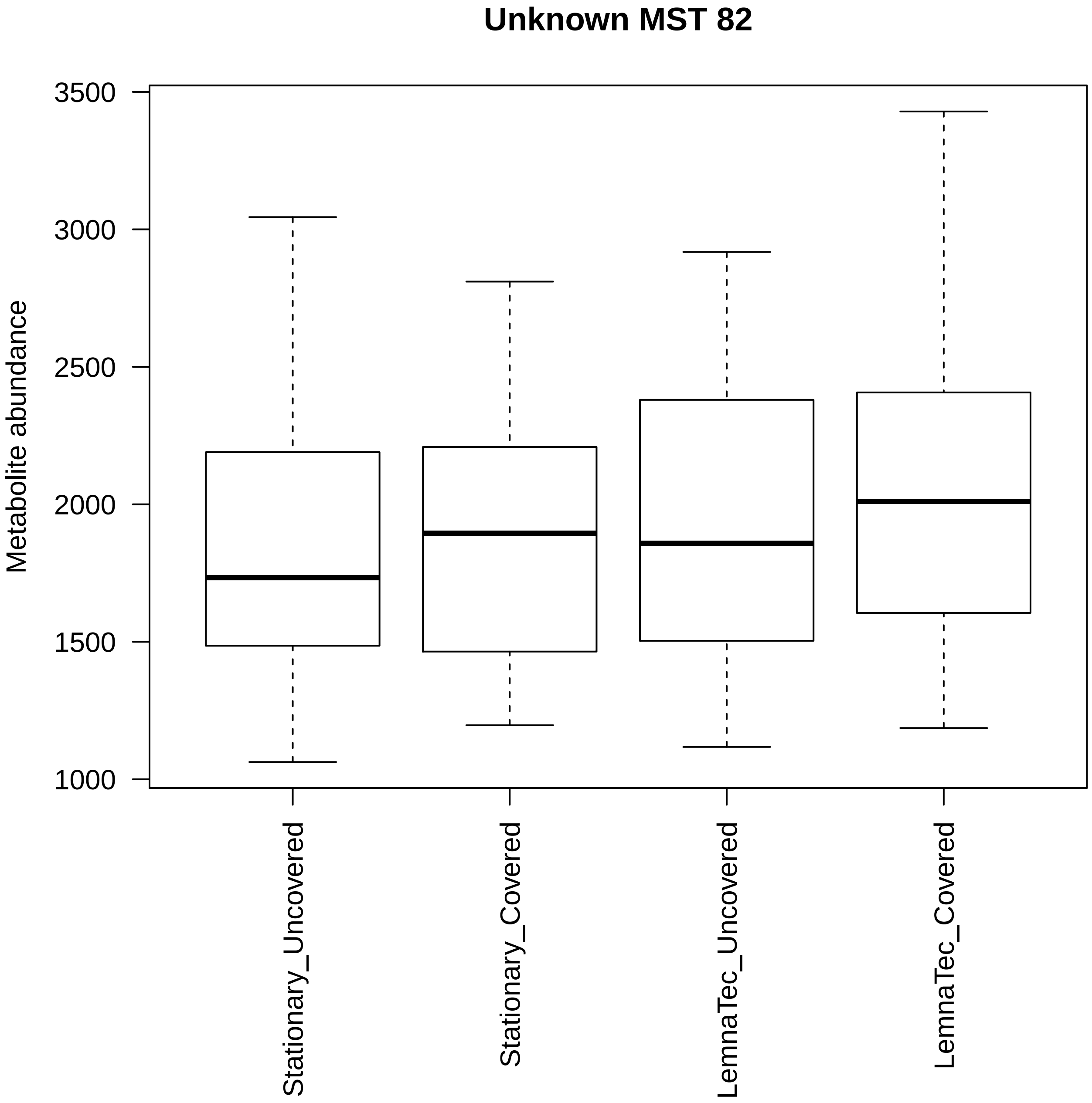

Unknown MST 82
Metabolite abundance
3500
3000
2500
2000
1500
1000
Stationary_Uncovered
Stationary_Covered
LemnaTec_Uncovered
LemnaTec_Covered

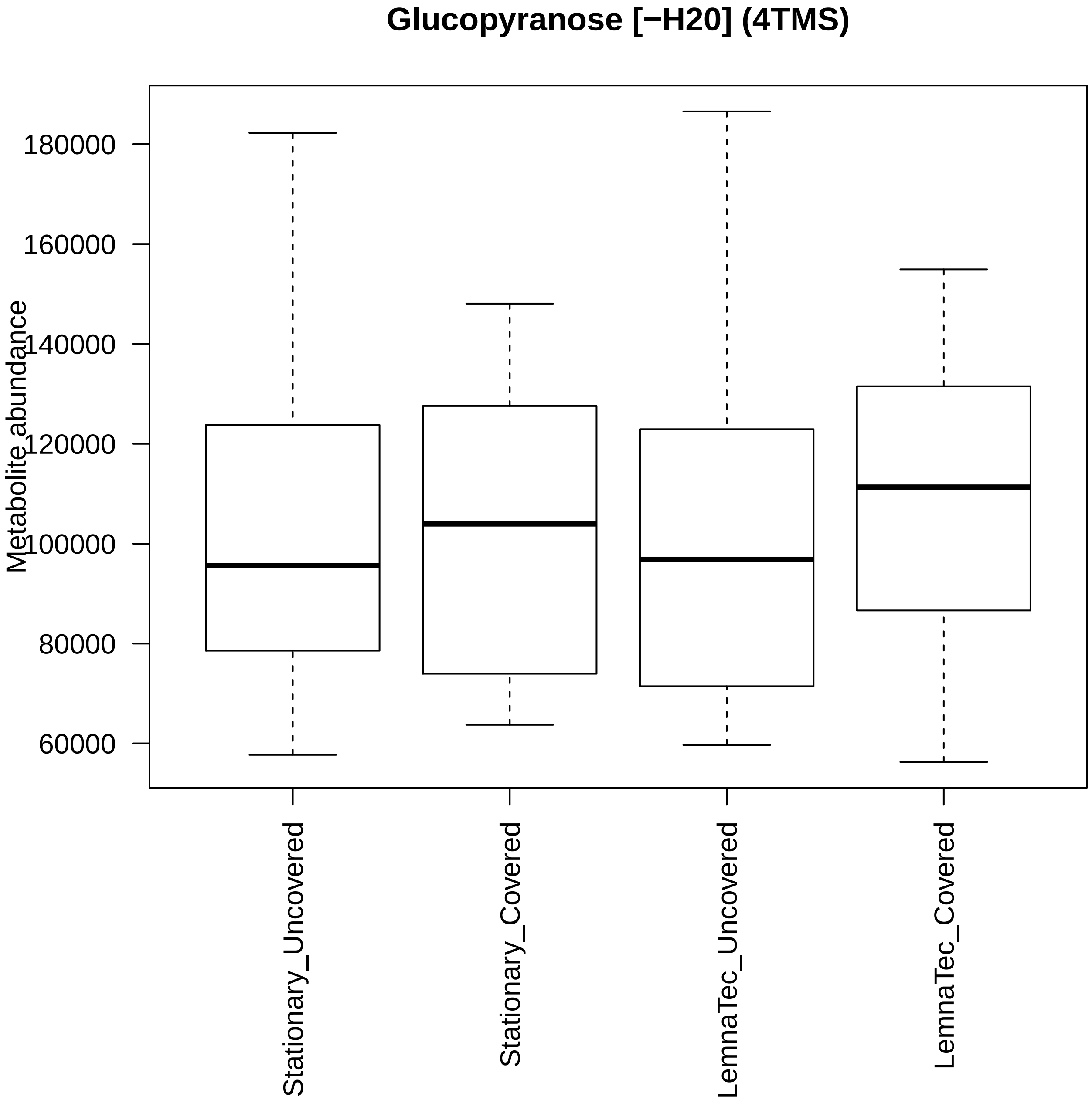

Glucopyranose [−H20] (4TMS)
Metabolite abundance
180000
160000
140000
120000
100000
80000
60000
Stationary_Uncovered
Stationary_Covered
LemnaTec_Uncovered
LemnaTec_Covered

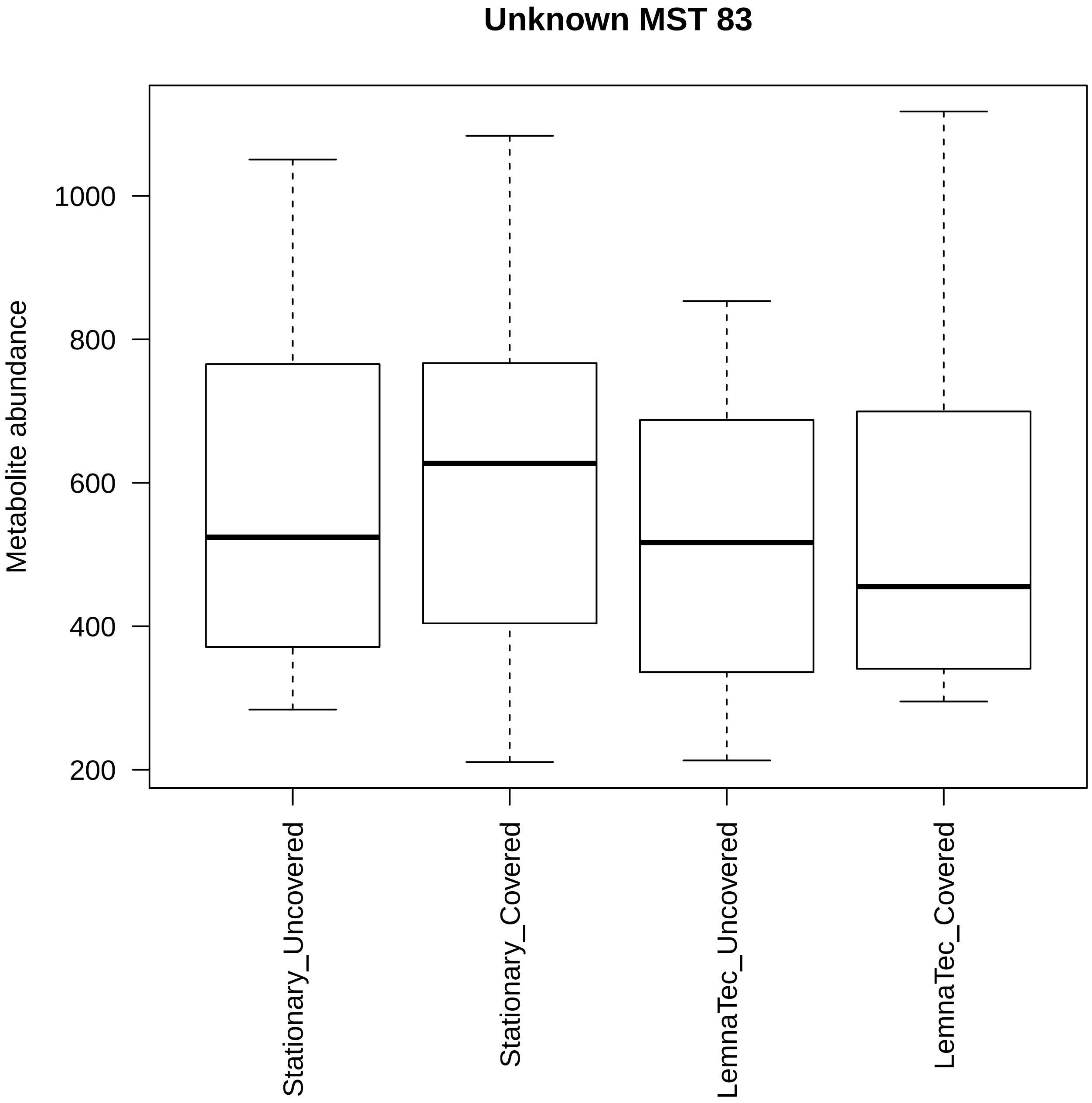

Unknown MST 83
Metabolite abundance
1000
800
600
400
200
Stationary_Uncovered
Stationary_Covered
LemnaTec_Uncovered
LemnaTec_Covered

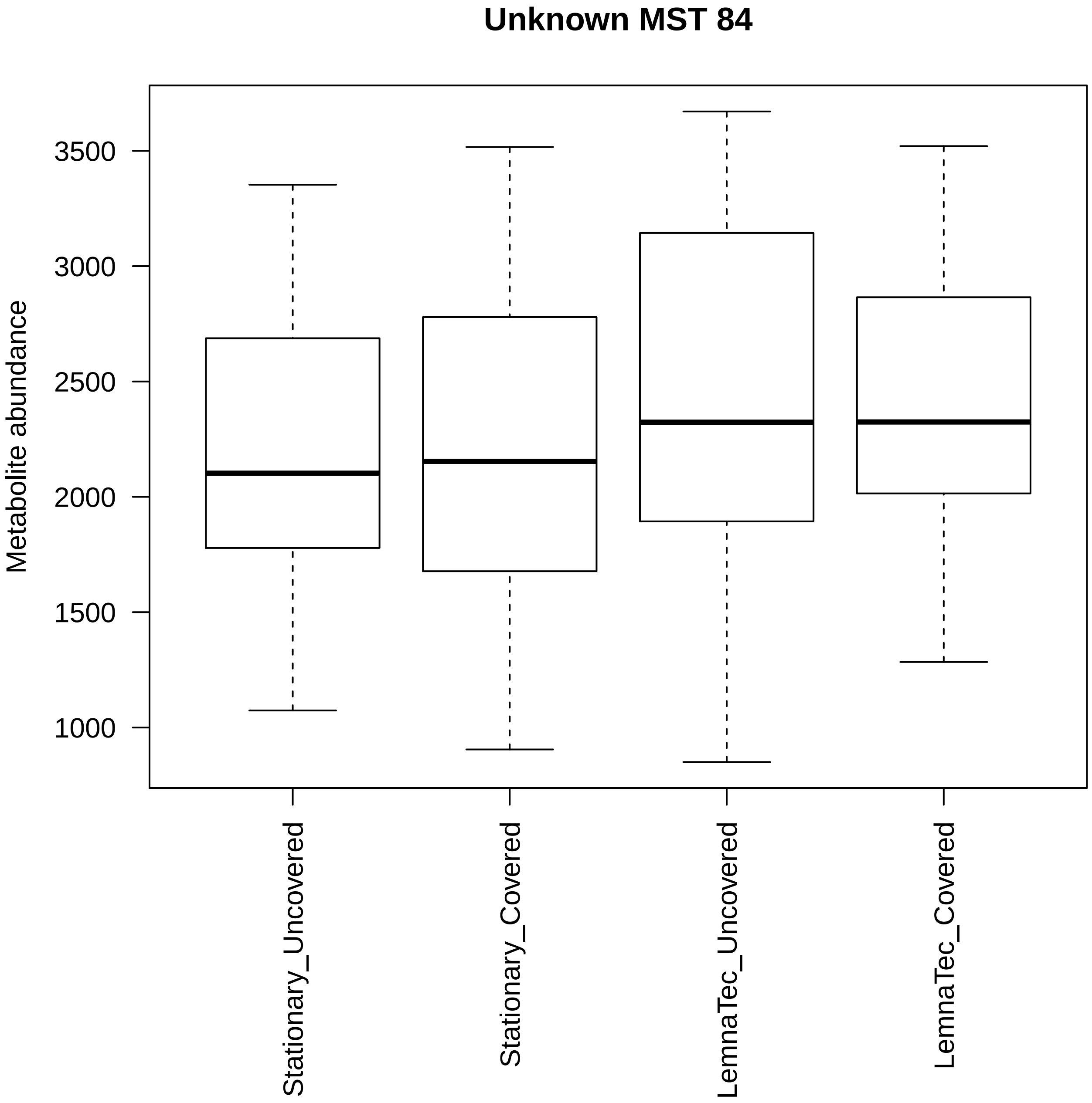

Unknown MST 84
Metabolite abundance
3500
3000
2500
2000
1500
1000
Stationary_Uncovered
Stationary_Covered
LemnaTec_Uncovered
LemnaTec_Covered

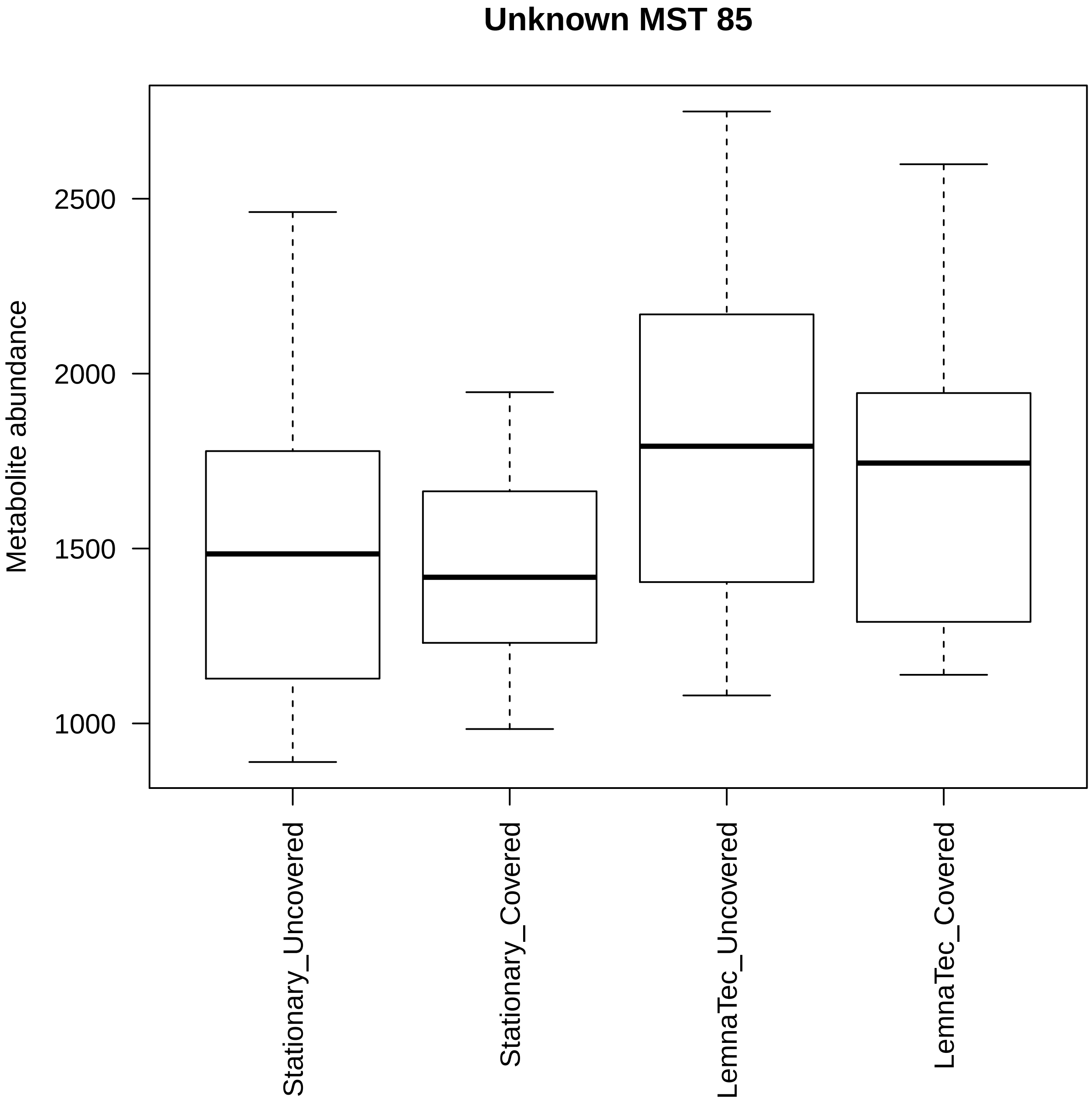

Unknown MST 85
Metabolite abundance
2500
2000
1500
1000
Stationary_Uncovered
Stationary_Covered
LemnaTec_Uncovered
LemnaTec_Covered

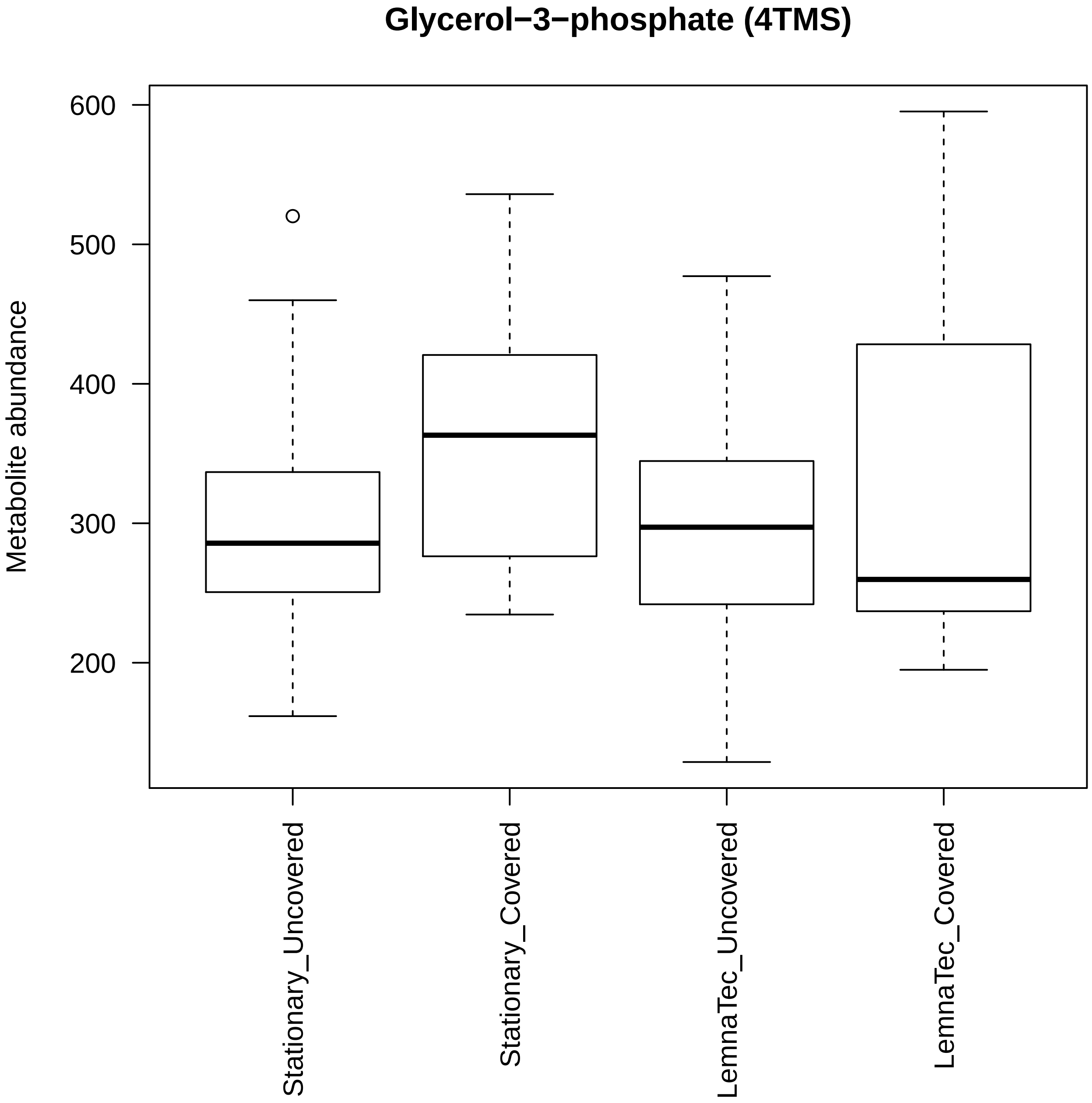

Glycerol−3−phosphate (4TMS)
Metabolite abundance
600
500
400
300
200
Stationary_Uncovered
Stationary_Covered
LemnaTec_Uncovered
LemnaTec_Covered

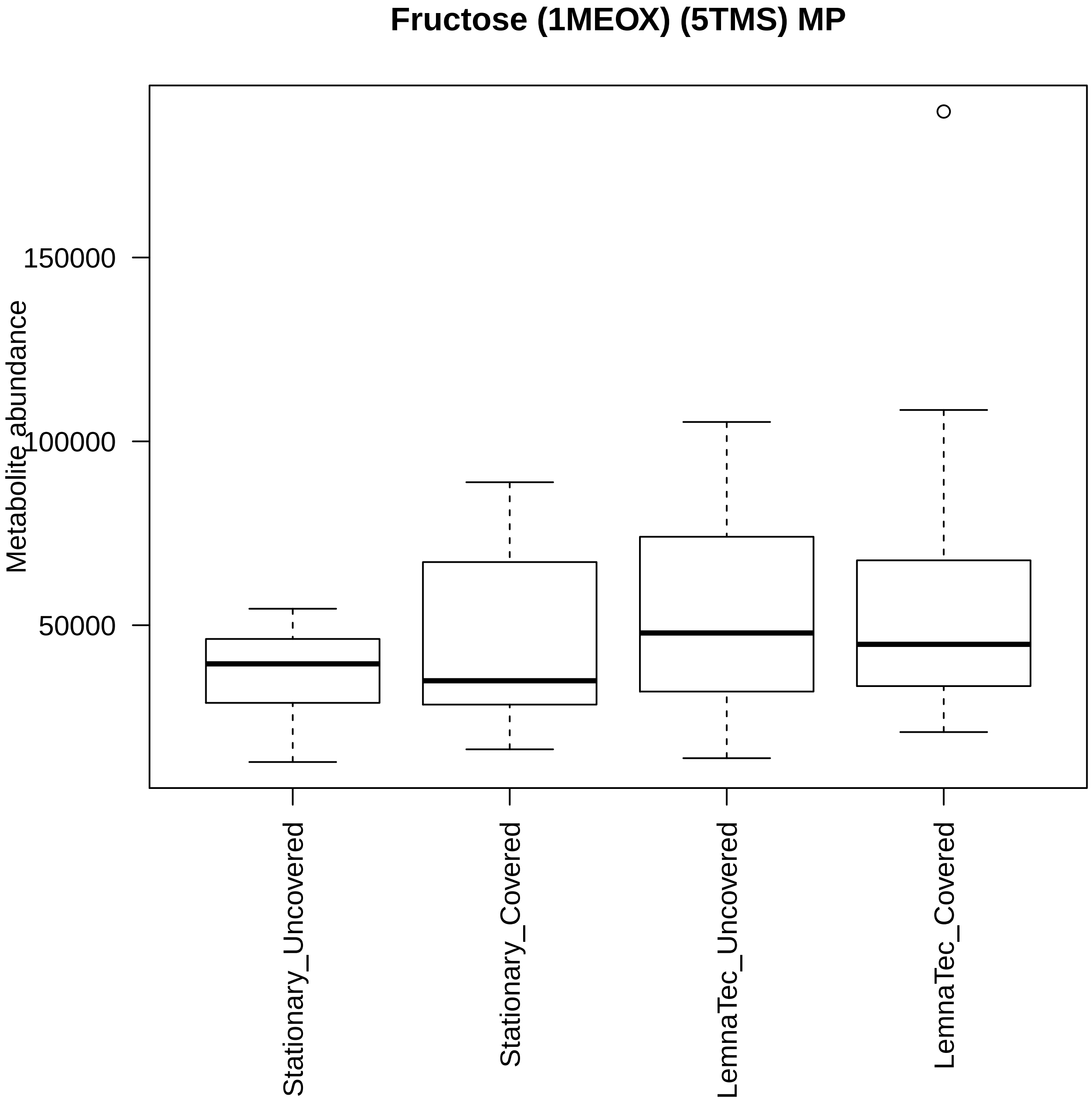
Fructose (1MEOX) (5TMS) MP
Metabolite abundance
150000
100000
50000
Stationary_Uncovered
Stationary_Covered
LemnaTec_Uncovered
LemnaTec_Covered

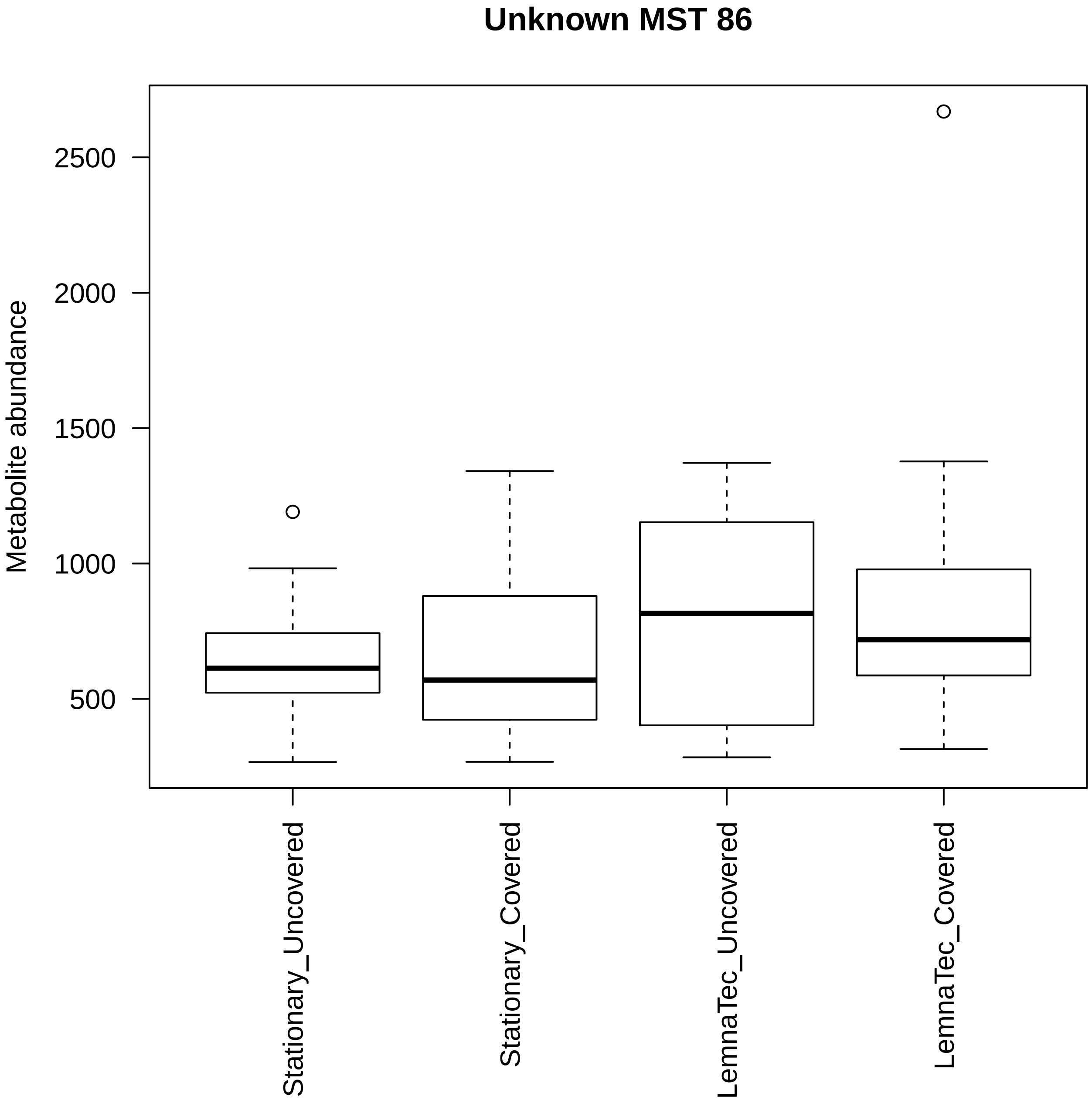
Unknown MST 86
Metabolite abundance
2500
2000
1500
1000
500
Stationary_Uncovered
Stationary_Covered
LemnaTec_Uncovered
LemnaTec_Covered

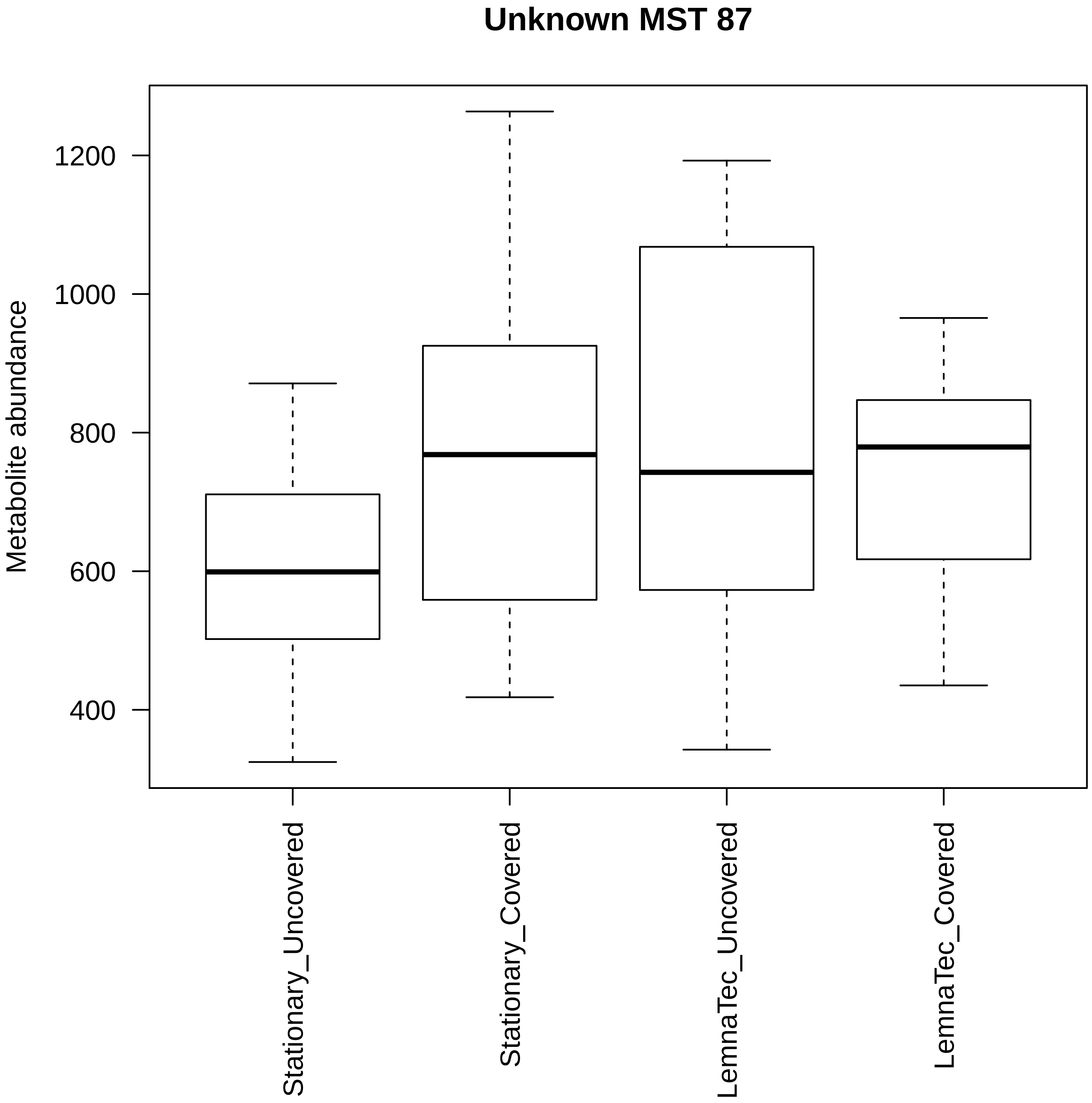
Unknown MST 87
Metabolite abundance
1200
1000
800
600
400
Stationary_Uncovered
Stationary_Covered
LemnaTec_Uncovered
LemnaTec_Covered

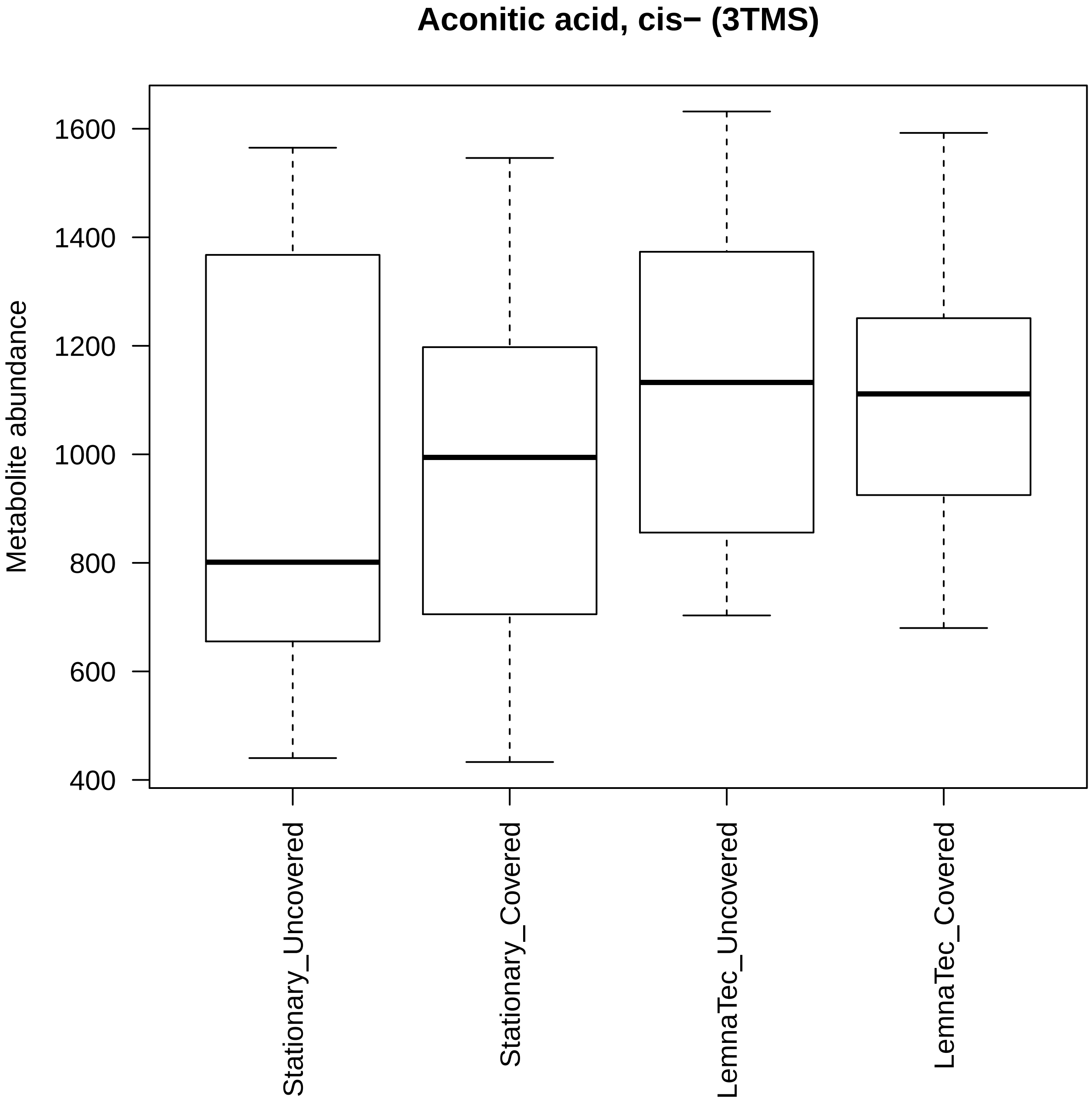

Aconitic acid, cis− (3TMS)
Metabolite abundance
1600
1400
1200
1000
800
600
400
Stationary_Uncovered
Stationary_Covered
LemnaTec_Uncovered
LemnaTec_Covered

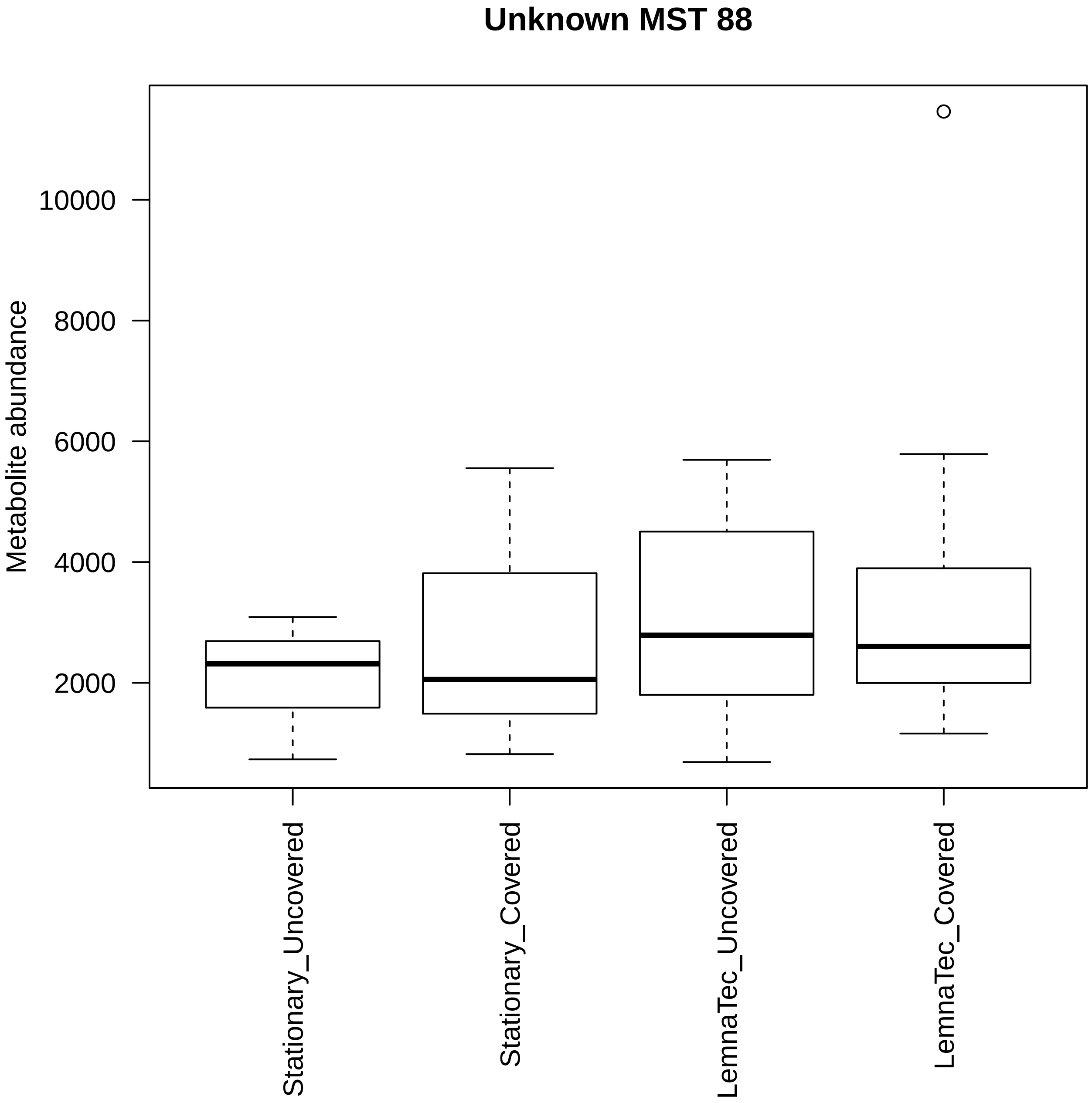

Unknown MST 88
Metabolite abundance
10000
8000
6000
4000
2000
Stationary_Uncovered
Stationary_Covered
LemnaTec_Uncovered
LemnaTec_Covered

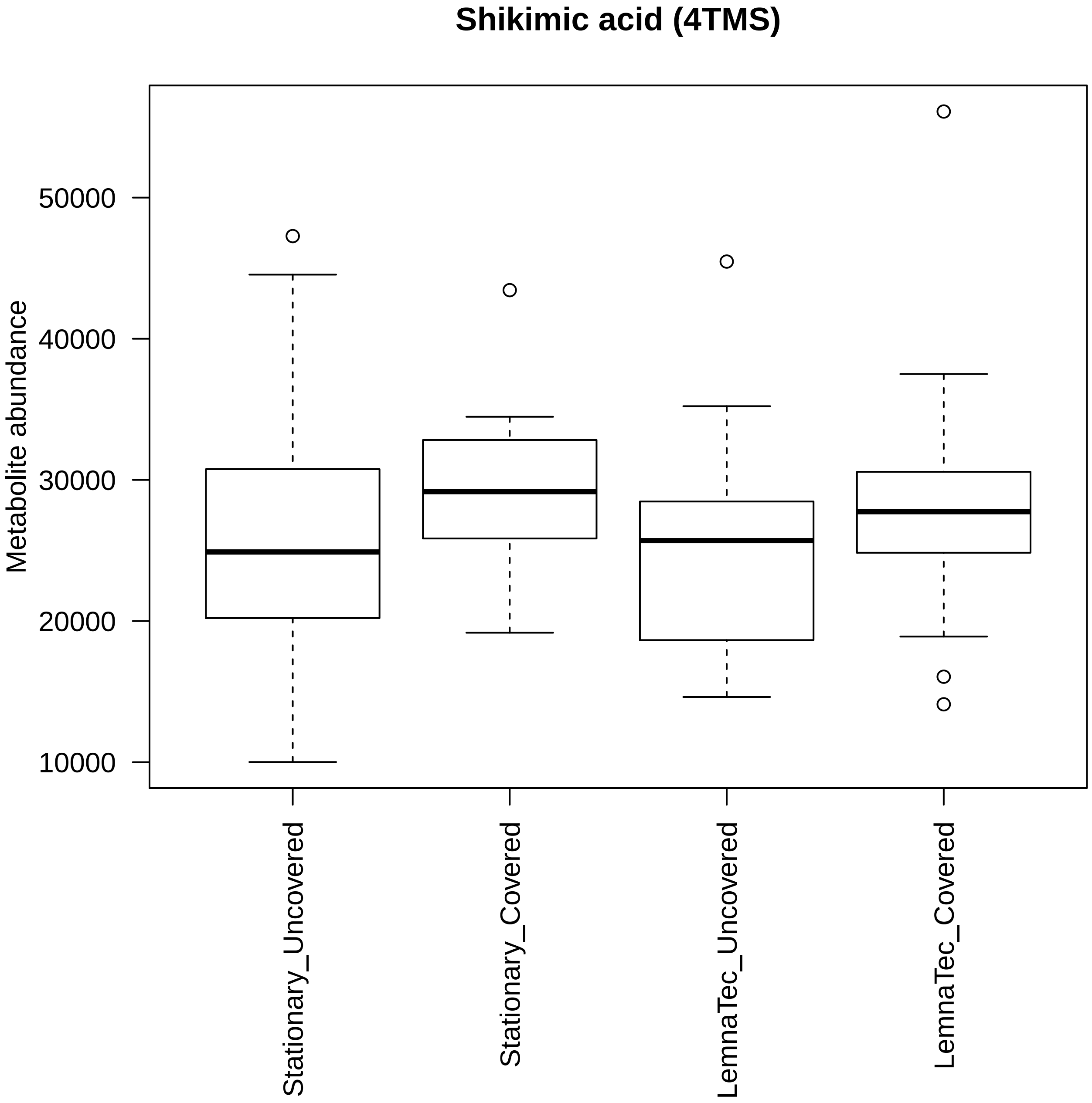
Shikimic acid (4TMS)
Metabolite abundance
10000
20000
30000
40000
50000
Stationary_Uncovered
Stationary_Covered
LemnaTec_Uncovered
LemnaTec_Covered

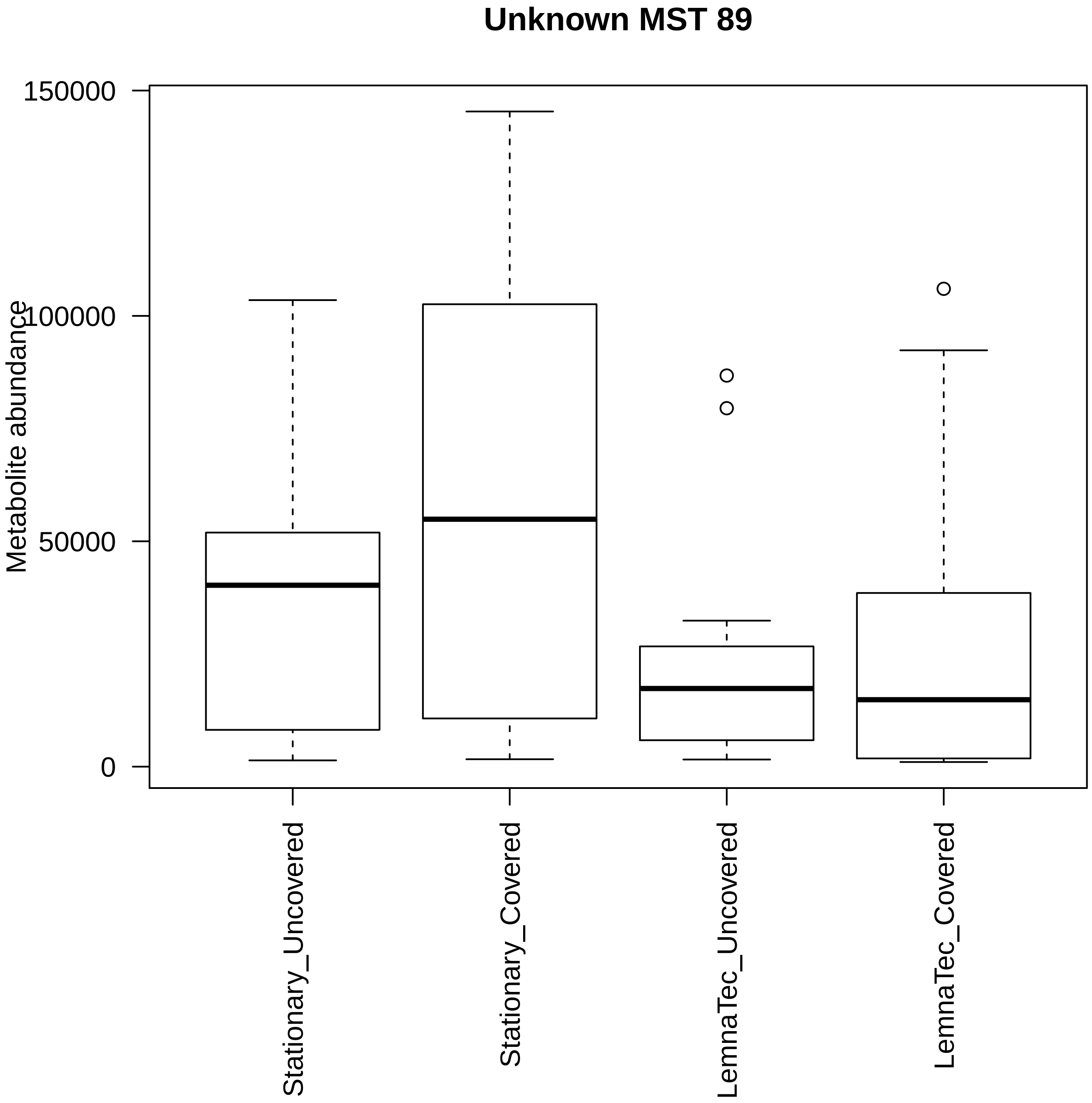

Unknown MST 89
Metabolite abundance
150000
100000
50000
0
Stationary_Uncovered
Stationary_Covered
LemnaTec_Uncovered
LemnaTec_Covered

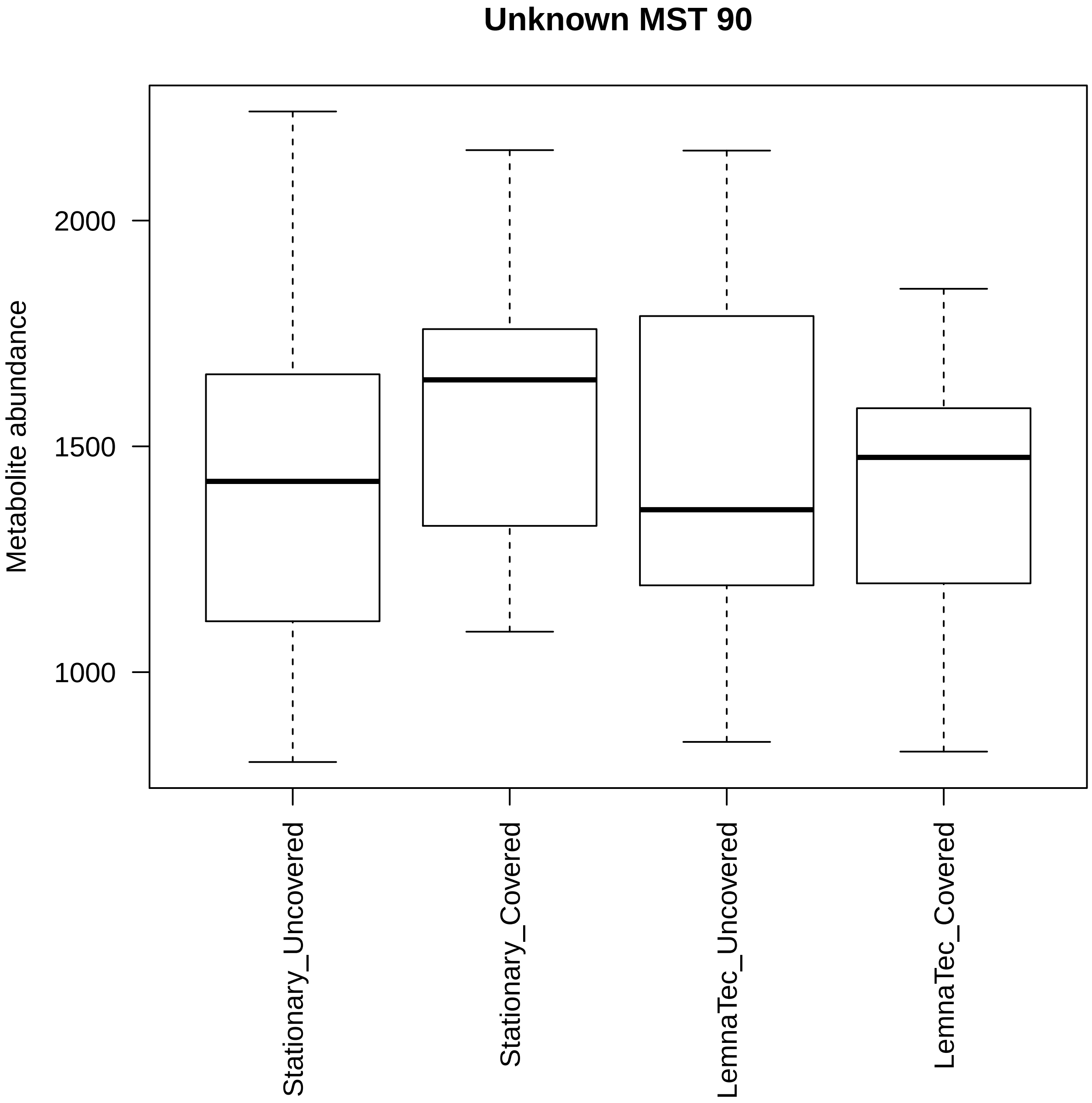
Unknown MST 90
Metabolite abundance
2000
1500
1000
Stationary_Uncovered
Stationary_Covered
LemnaTec_Uncovered
LemnaTec_Covered

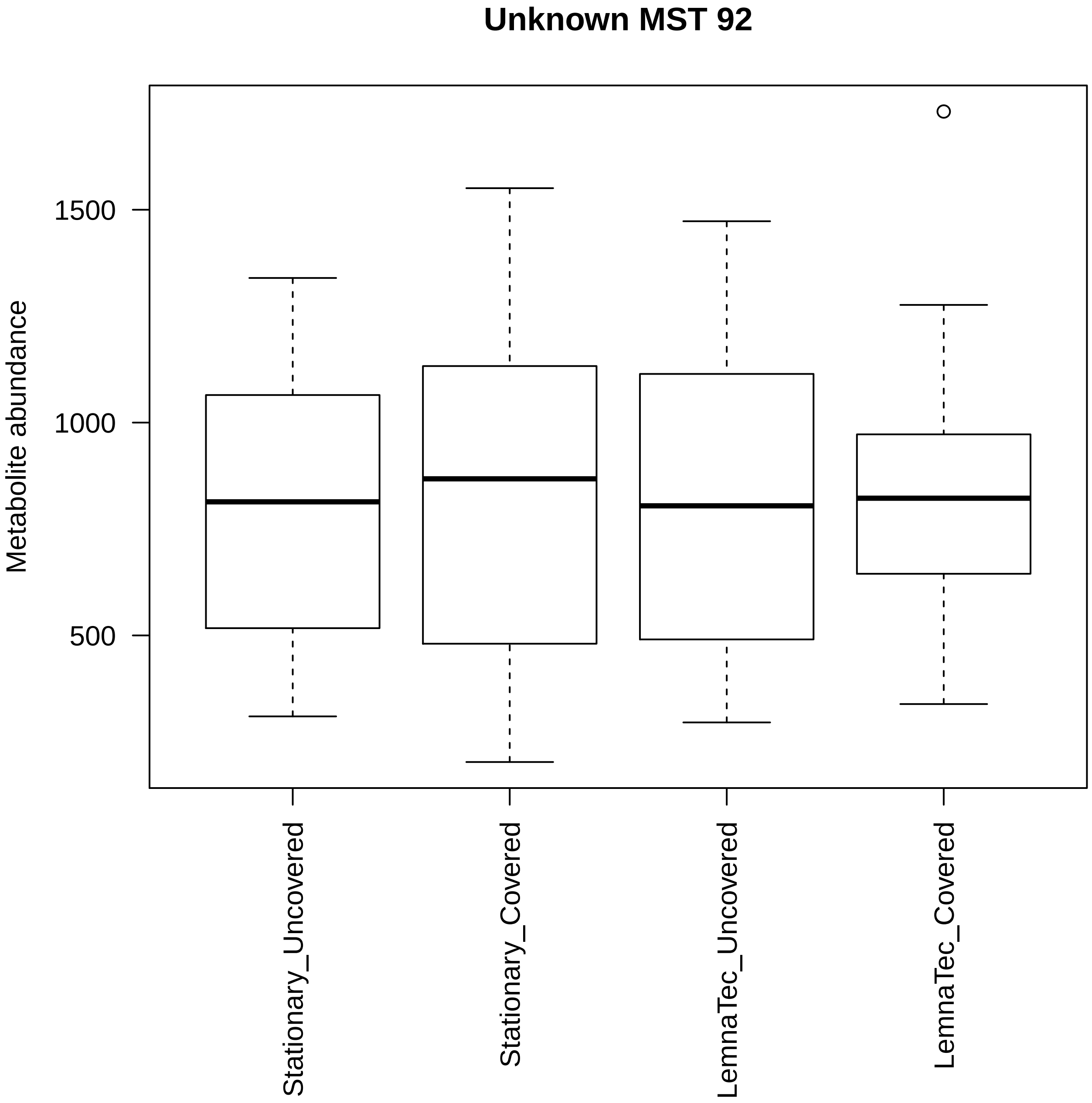

Unknown MST 92
Metabolite abundance
1500
1000
500
Stationary_Uncovered
Stationary_Covered
LemnaTec_Uncovered
LemnaTec_Covered

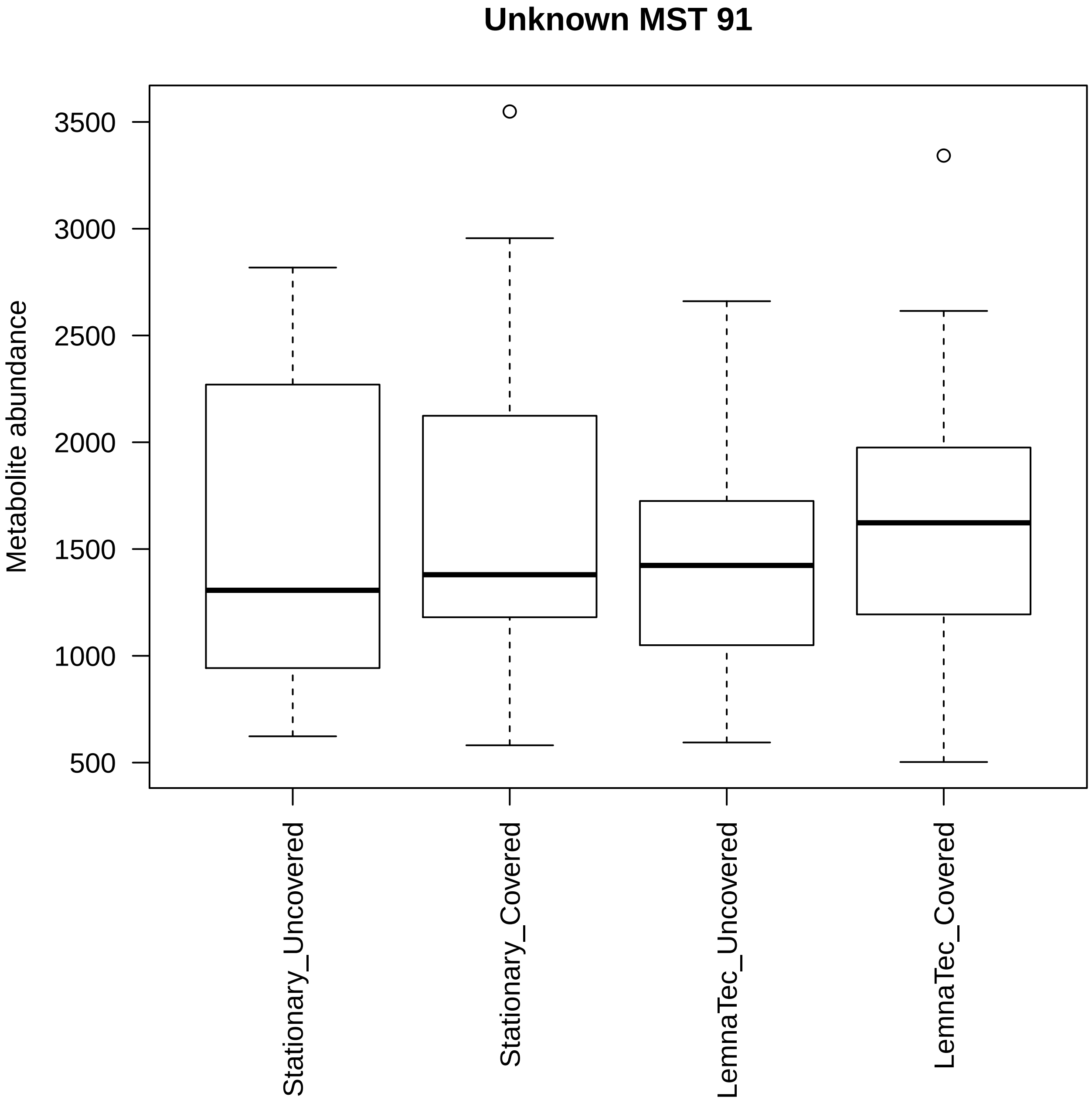

Unknown MST 91
Metabolite abundance
3500
3000
2500
2000
1500
1000
500
Stationary_Uncovered
Stationary_Covered
LemnaTec_Uncovered
LemnaTec_Covered

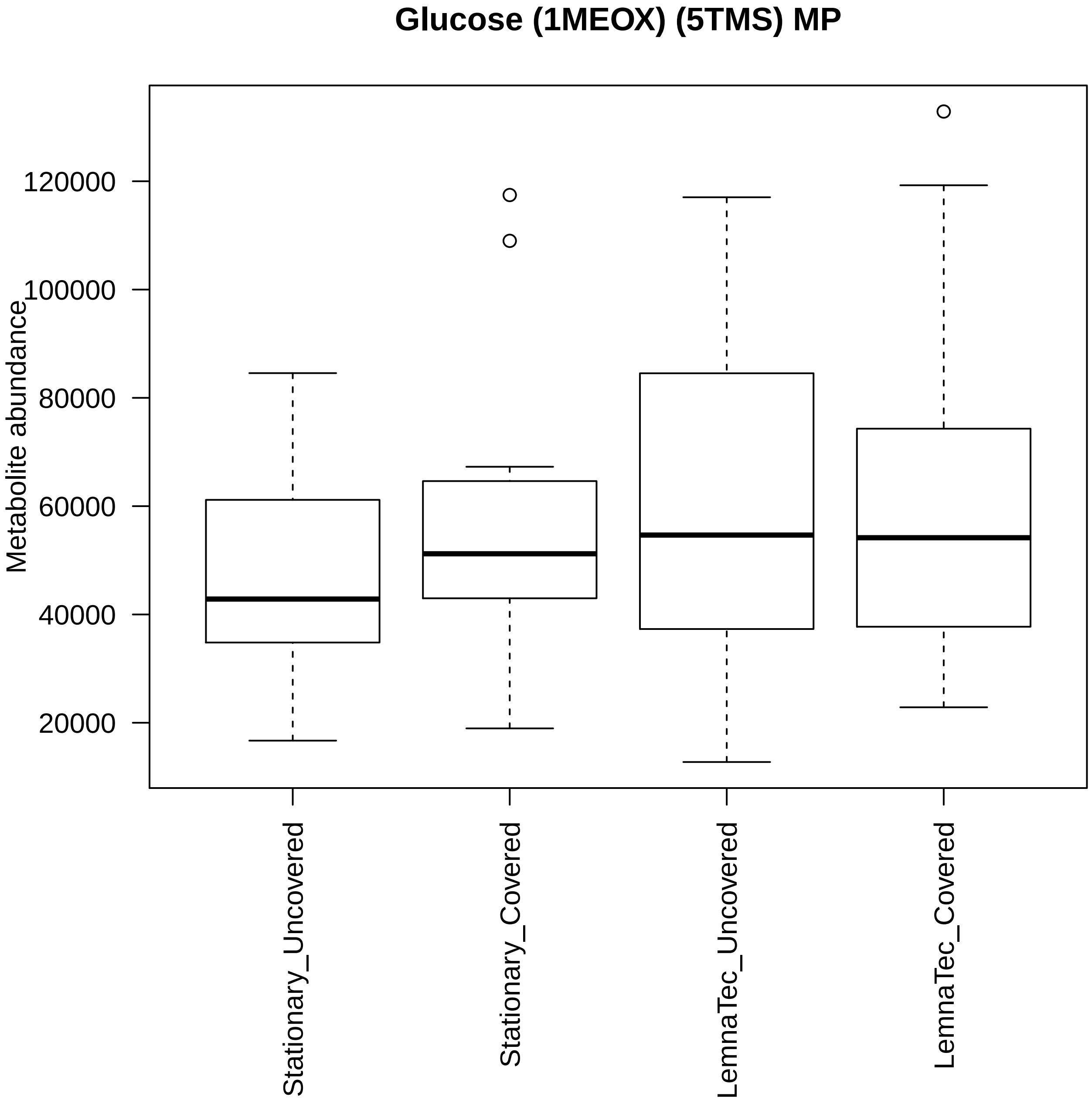

Glucose (1MEOX) (5TMS) MP
Metabolite abundance
120000
100000
80000
60000
40000
20000
Stationary_Uncovered
Stationary_Covered
LemnaTec_Uncovered
LemnaTec_Covered

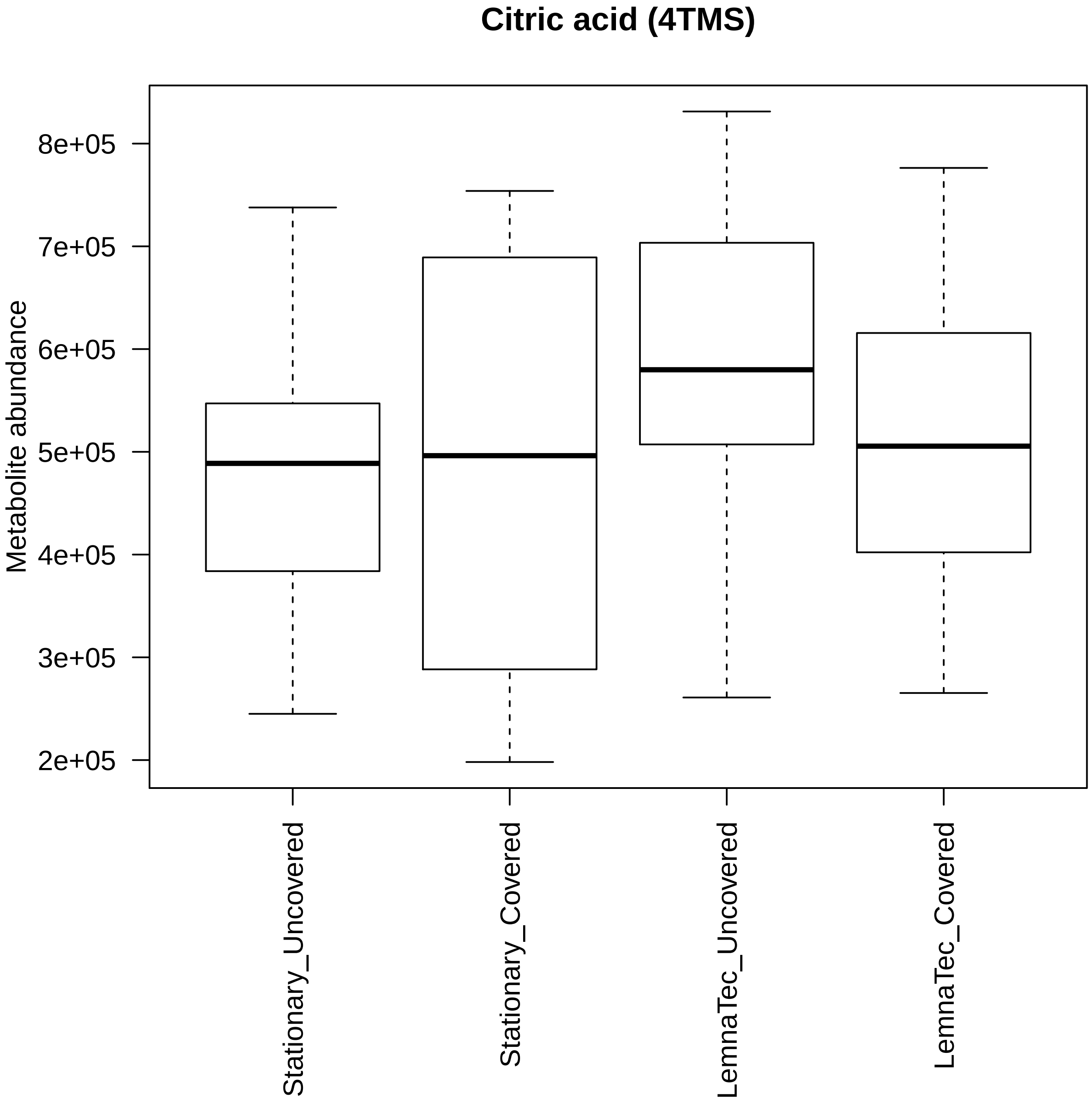
Citric acid (4TMS)
Metabolite abundance
8e+05
7e+05
6e+05
5e+05
4e+05
3e+05
2e+05
Stationary_Uncovered
Stationary_Covered
LemnaTec_Uncovered
LemnaTec_Covered

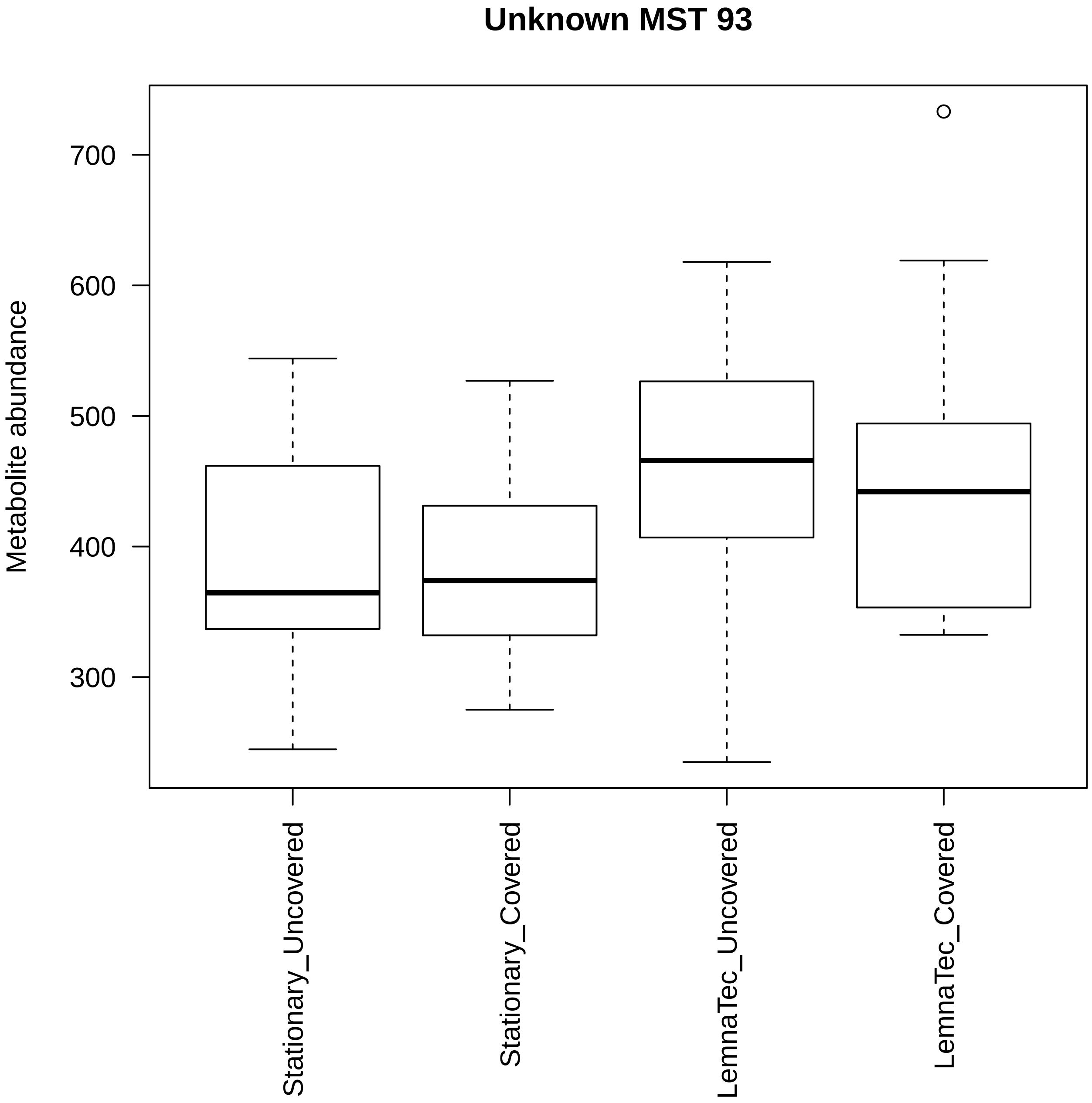
Unknown MST 93
Metabolite abundance
700
600
500
400
300
Stationary_Uncovered
Stationary_Covered
LemnaTec_Uncovered
LemnaTec_Covered

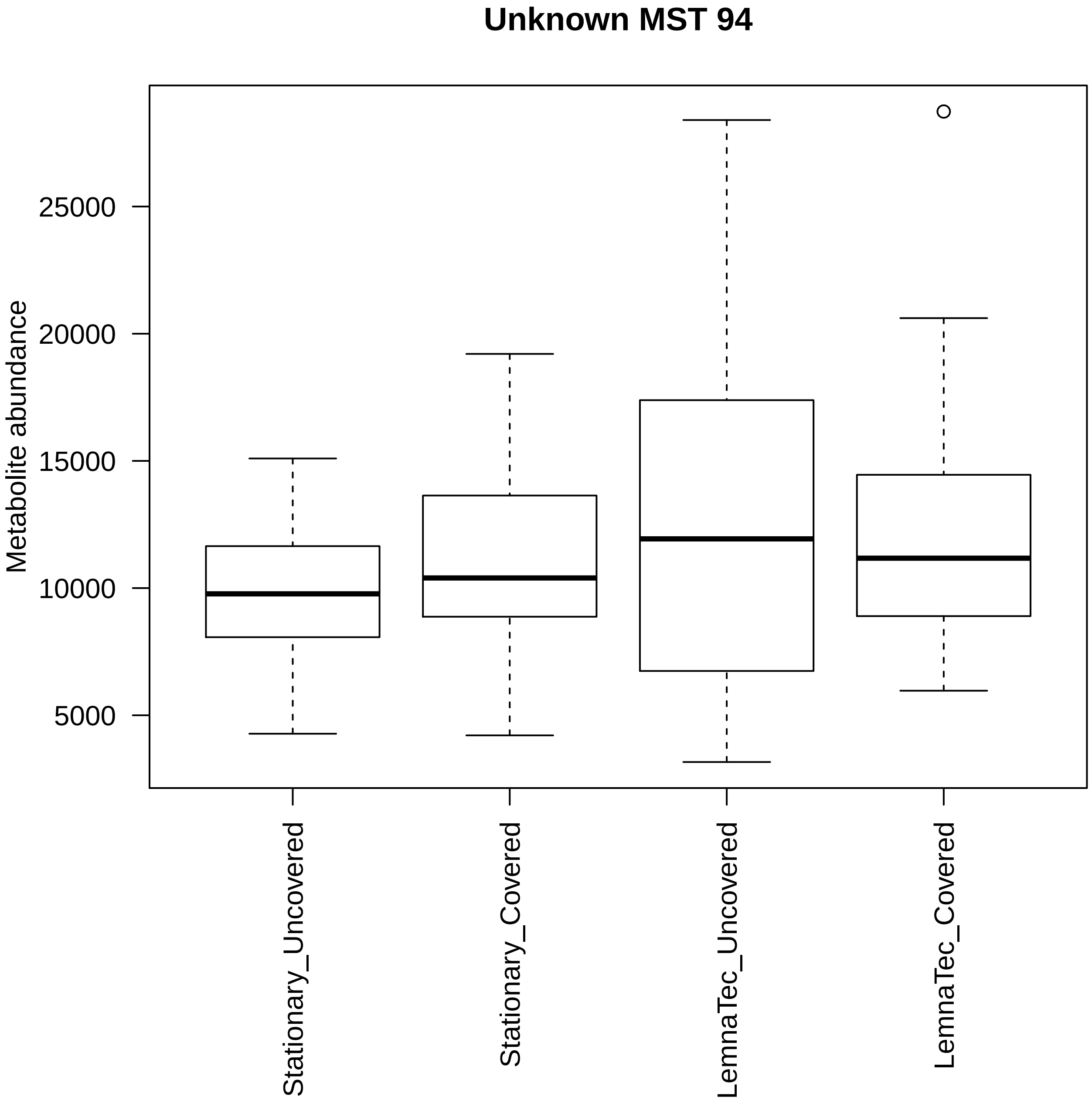

Unknown MST 94
Metabolite abundance
25000
20000
15000
10000
5000
Stationary_Uncovered
Stationary_Covered
LemnaTec_Uncovered
LemnaTec_Covered

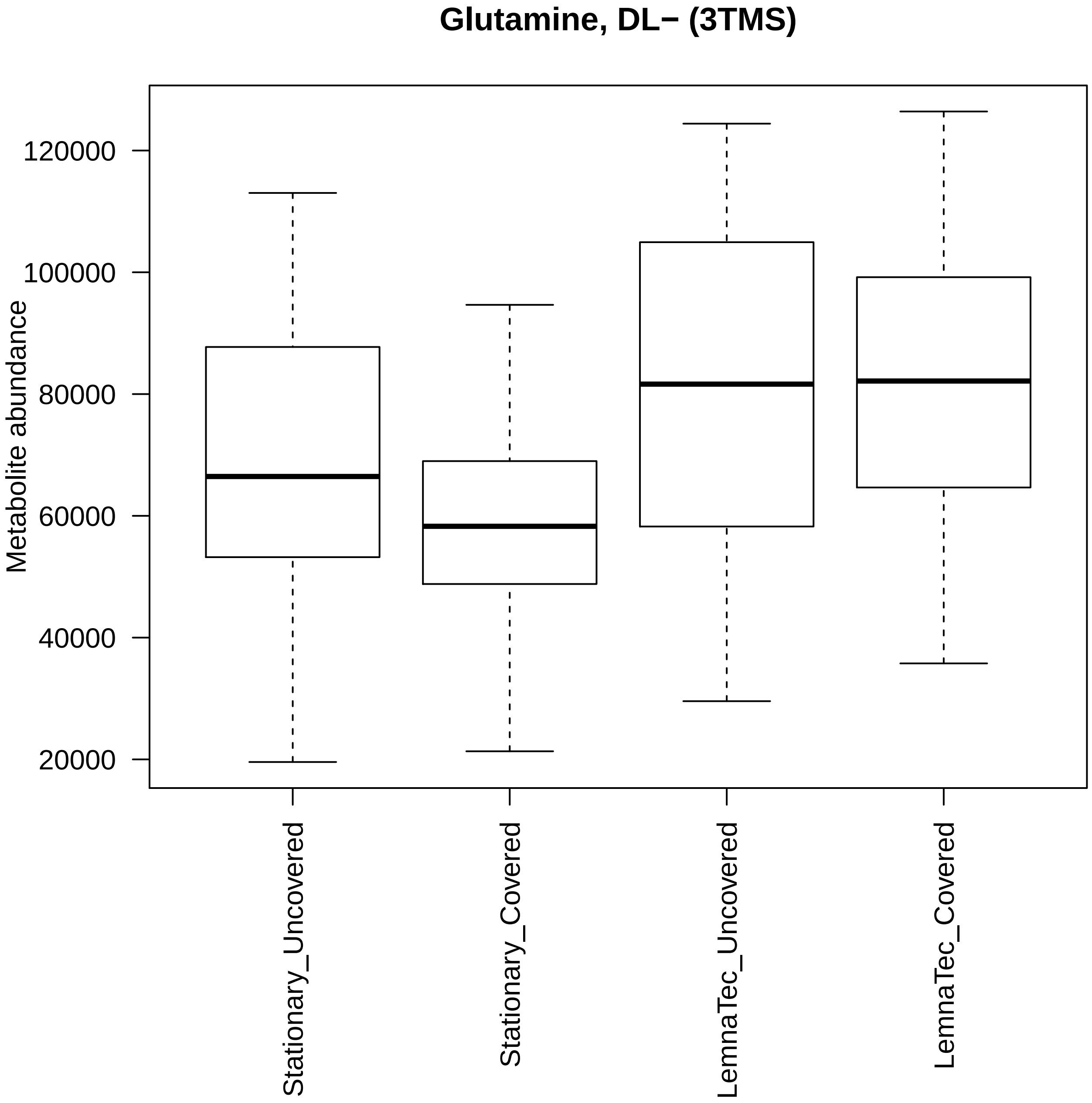
Glutamine, DL− (3TMS)
Metabolite abundance
120000
100000
80000
60000
40000
20000
Stationary_Uncovered
Stationary_Covered
LemnaTec_Uncovered
LemnaTec_Covered

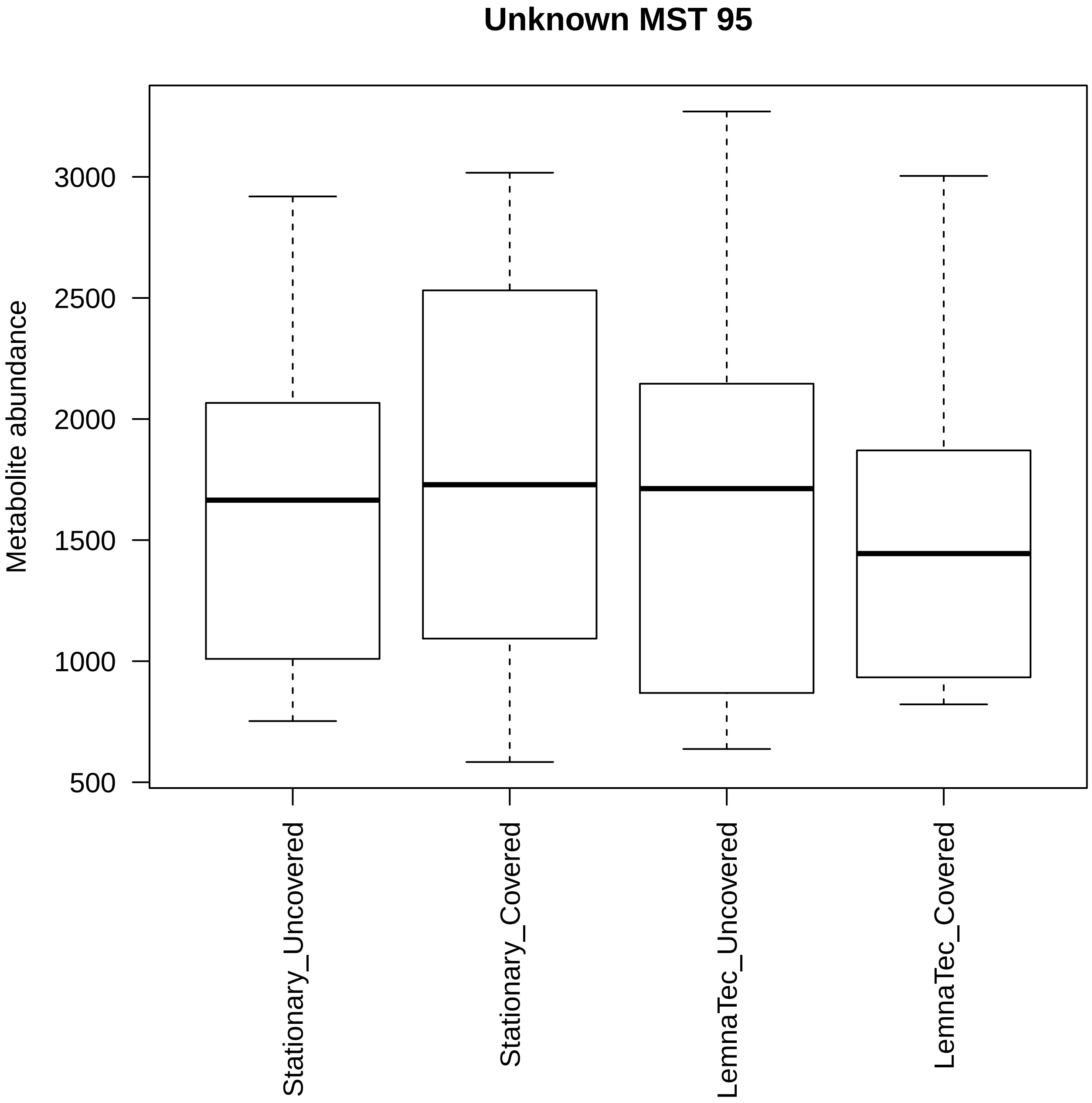
Unknown MST 95
Metabolite abundance
500
1000
1500
2000
2500
3000
Stationary_Uncovered
Stationary_Covered
LemnaTec_Uncovered
LemnaTec_Covered

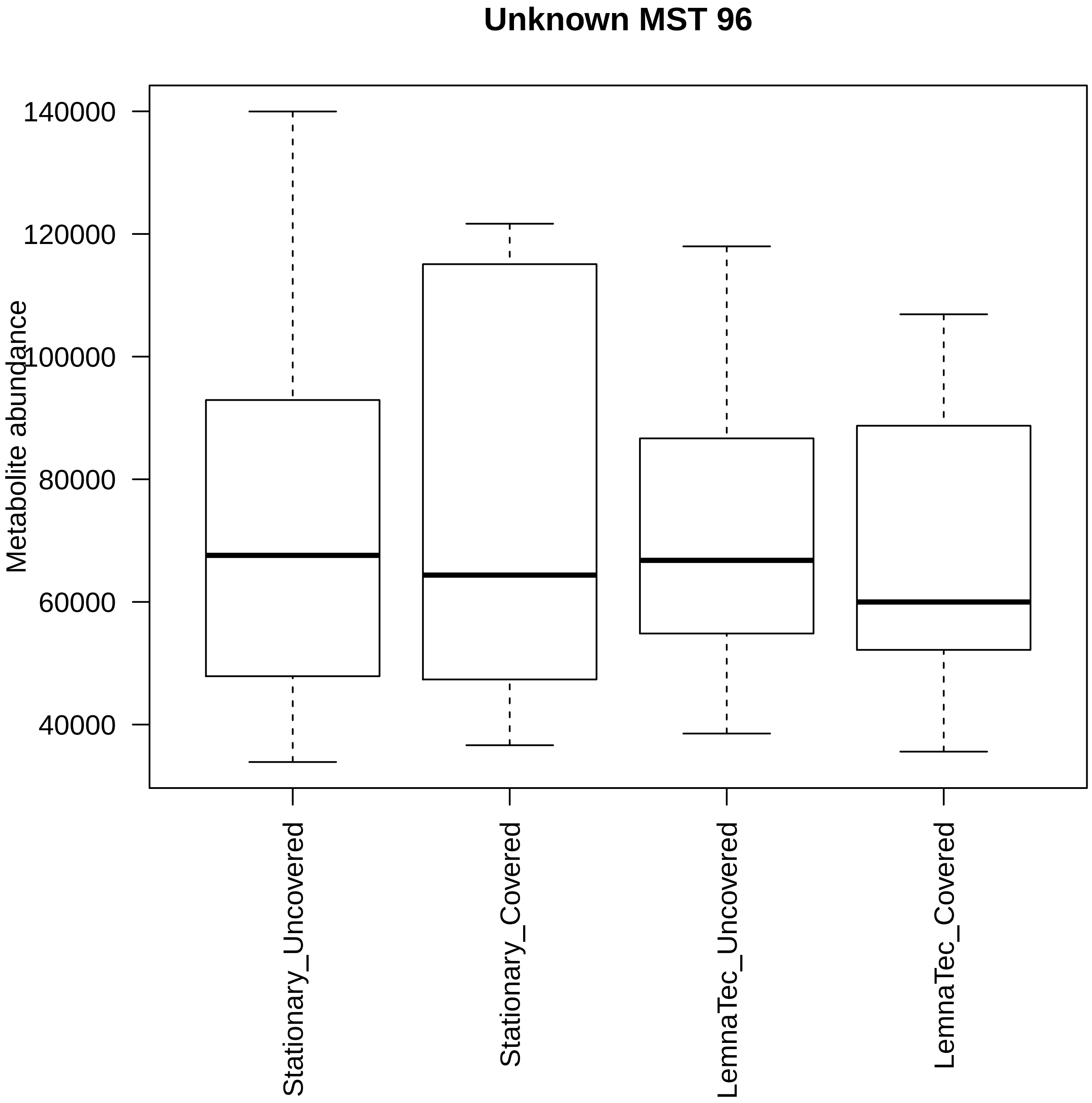
Unknown MST 96
Metabolite abundance
140000
120000
100000
80000
60000
40000
Stationary_Uncovered
Stationary_Covered
LemnaTec_Uncovered
LemnaTec_Covered

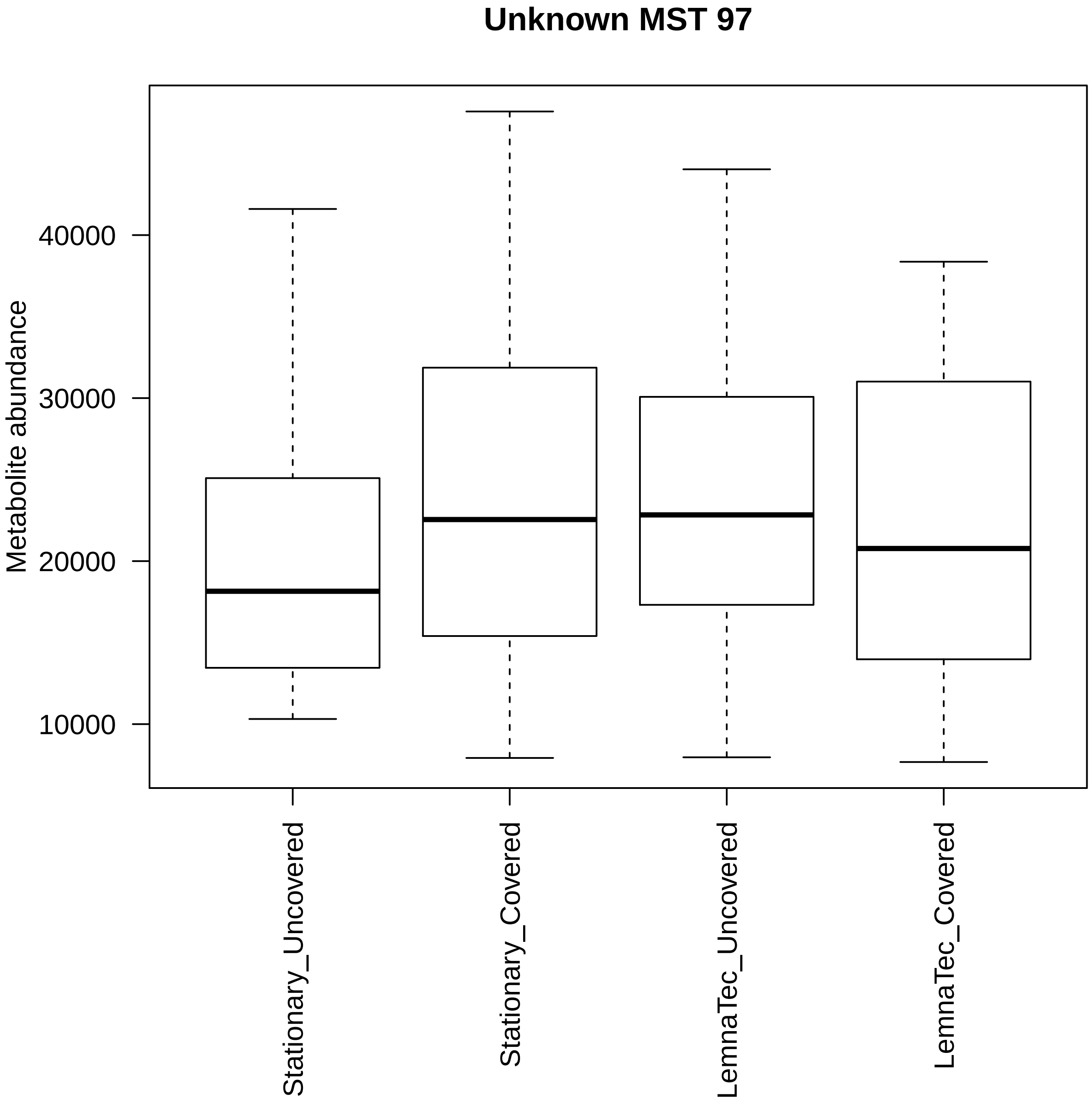

Unknown MST 97
Metabolite abundance
10000
20000
30000
40000
Stationary_Uncovered
Stationary_Covered
LemnaTec_Uncovered
LemnaTec_Covered

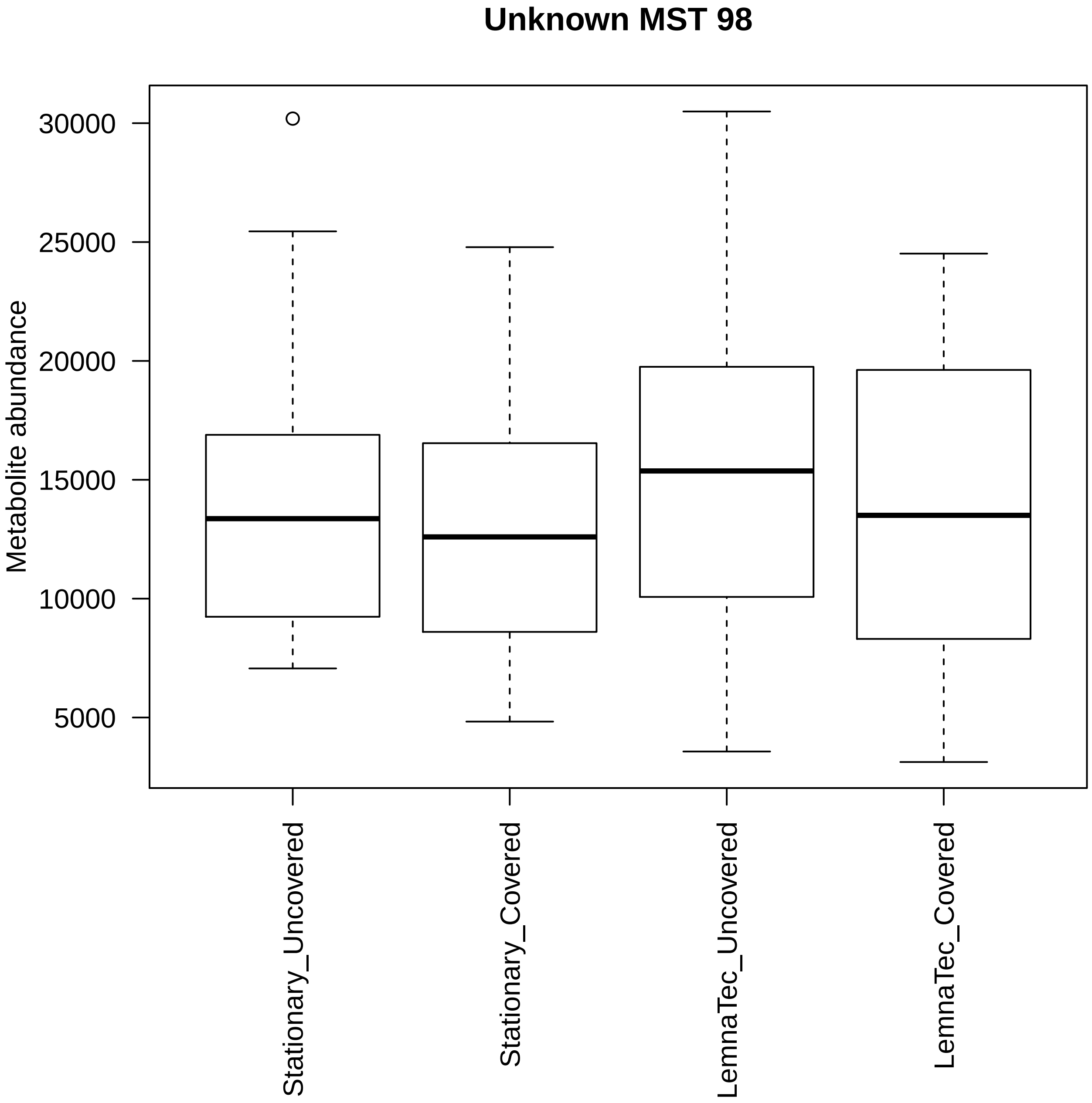

Unknown MST 98
Metabolite abundance
30000
25000
20000
15000
10000
5000
Stationary_Uncovered
Stationary_Covered
LemnaTec_Uncovered
LemnaTec_Covered

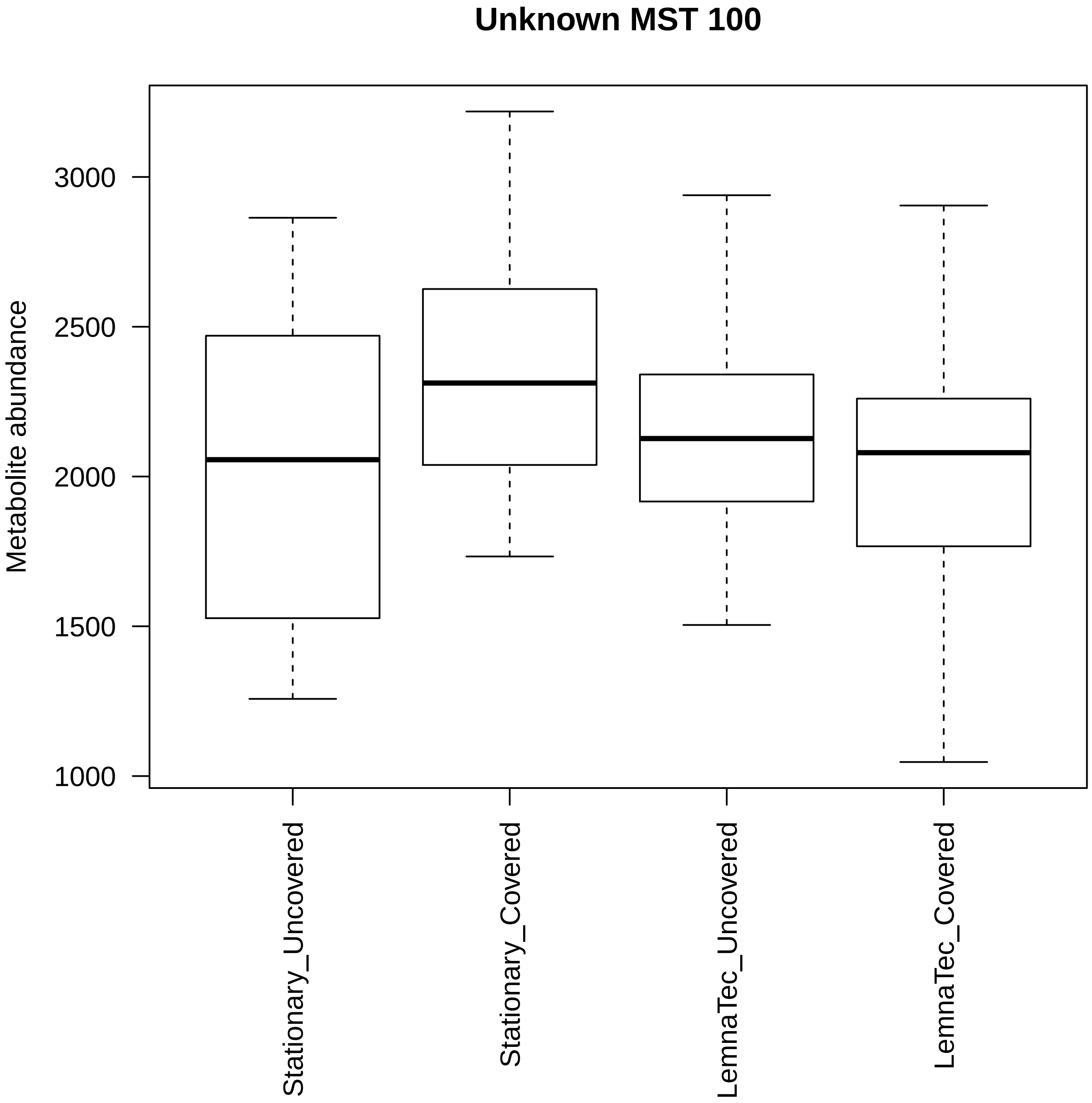

Unknown MST 100
Metabolite abundance
3000
2500
2000
1500
1000
Stationary_Uncovered
Stationary_Covered
LemnaTec_Uncovered
LemnaTec_Covered

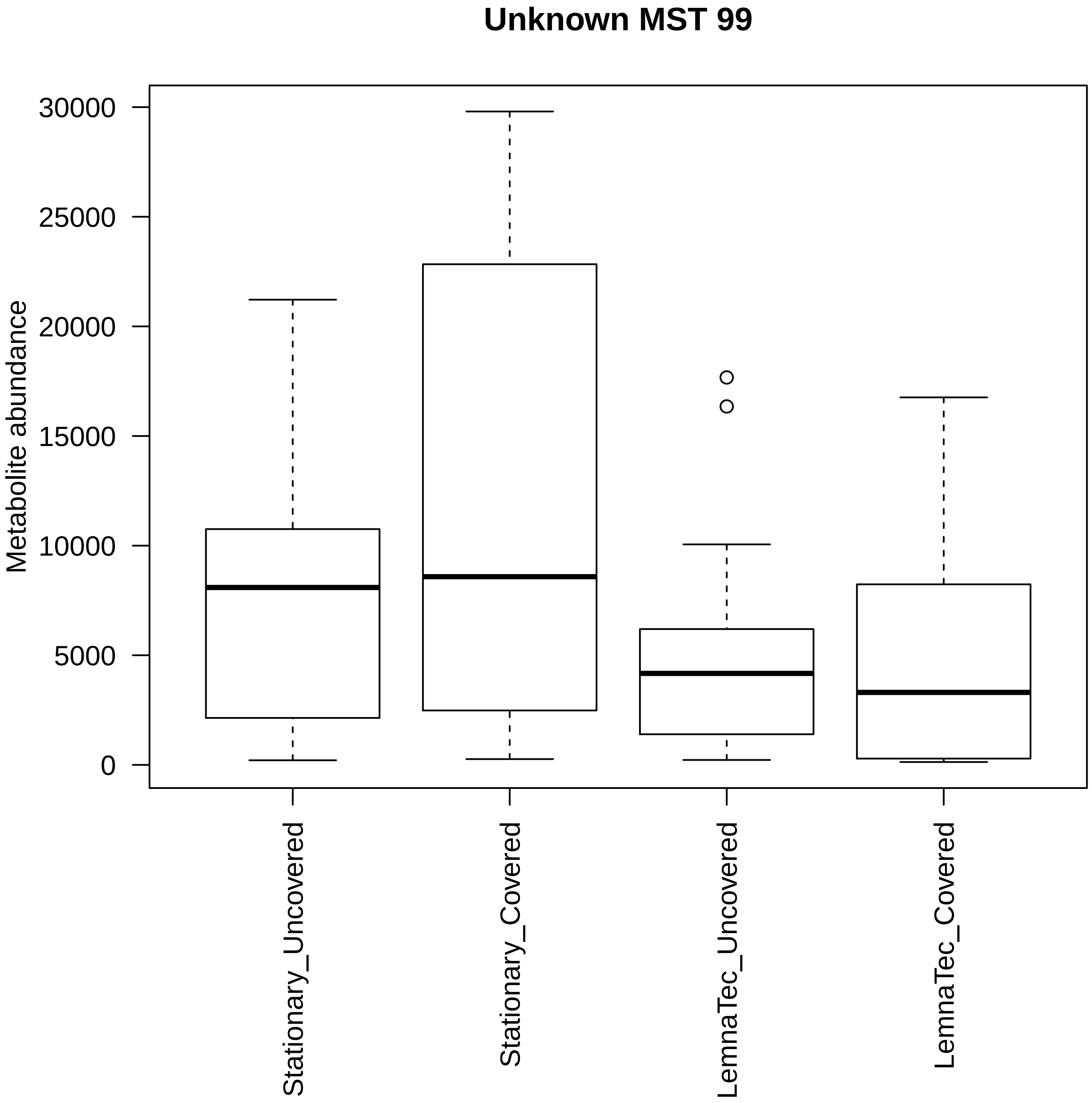
Unknown MST 99
Metabolite abundance
0
5000
10000
15000
20000
25000
30000
Stationary_Uncovered
Stationary_Covered
LemnaTec_Uncovered
LemnaTec_Covered

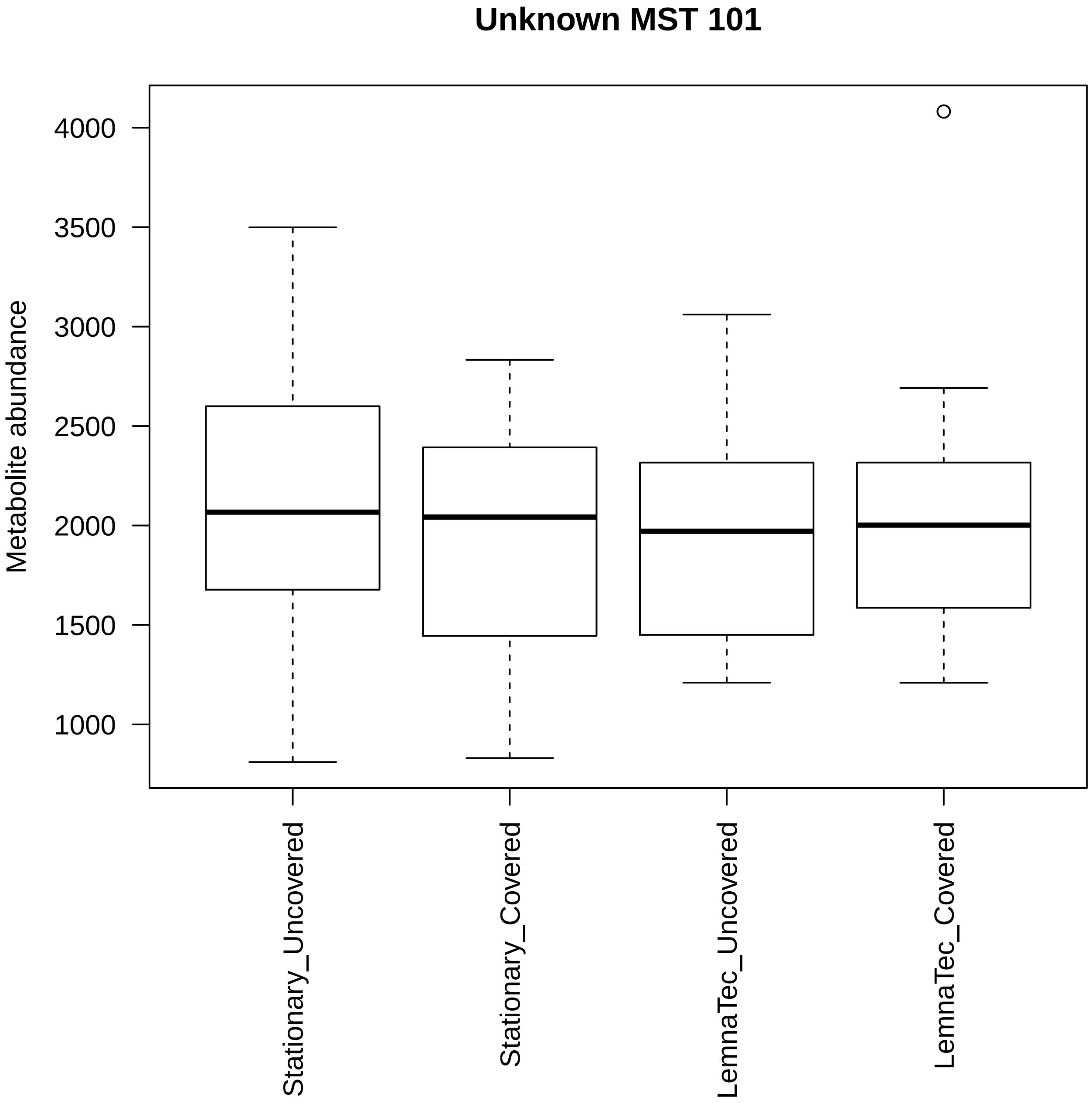
Unknown MST 101
Metabolite abundance
4000
3500
3000
2500
2000
1500
1000
Stationary_Uncovered
Stationary_Covered
LemnaTec_Uncovered
LemnaTec_Covered

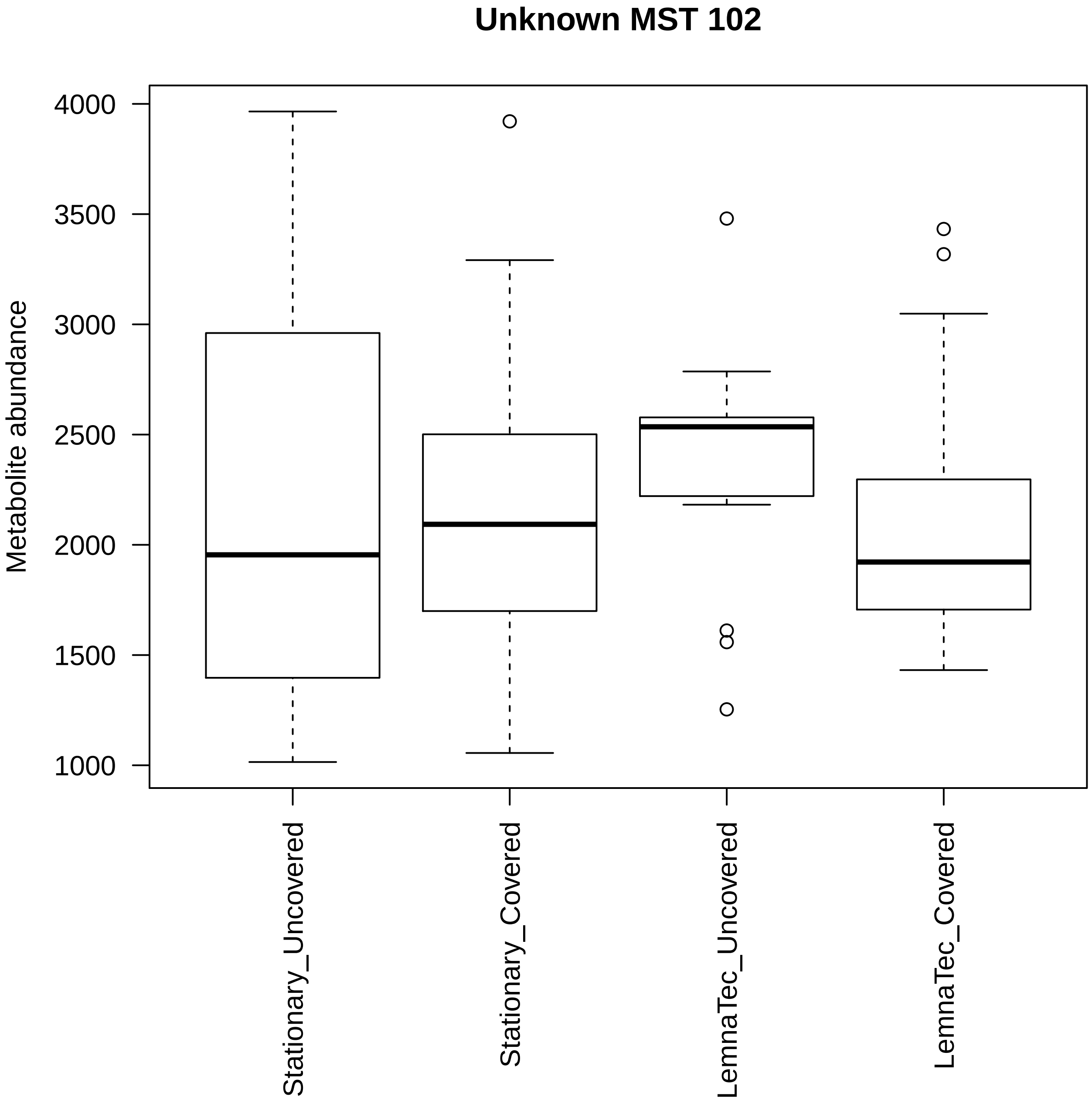

Unknown MST 102
Metabolite abundance
4000
3500
3000
2500
2000
1500
1000
Stationary_Uncovered
Stationary_Covered
LemnaTec_Uncovered
LemnaTec_Covered

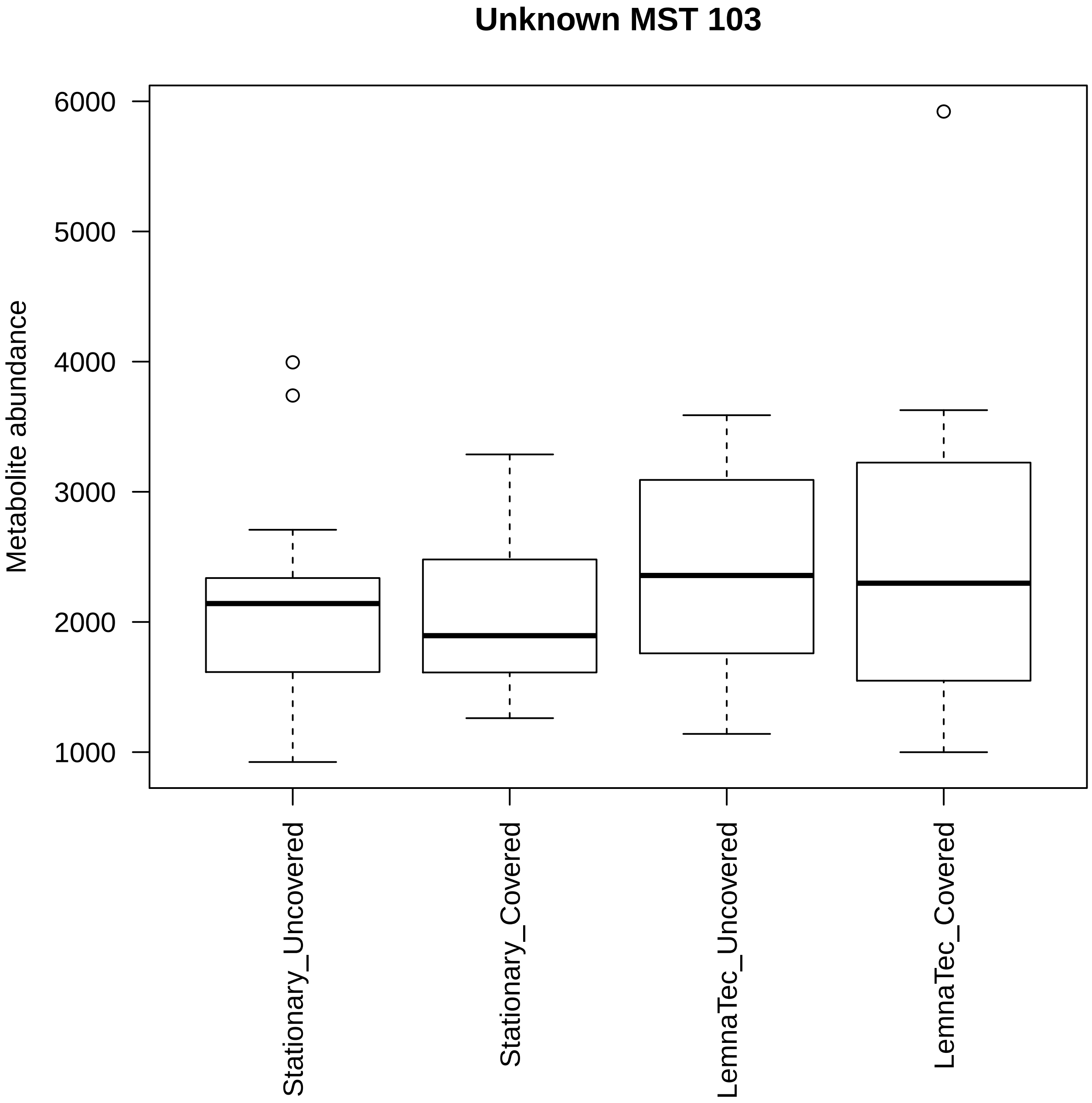
Unknown MST 103
Metabolite abundance
6000
5000
4000
3000
2000
1000
Stationary_Uncovered
Stationary_Covered
LemnaTec_Uncovered
LemnaTec_Covered

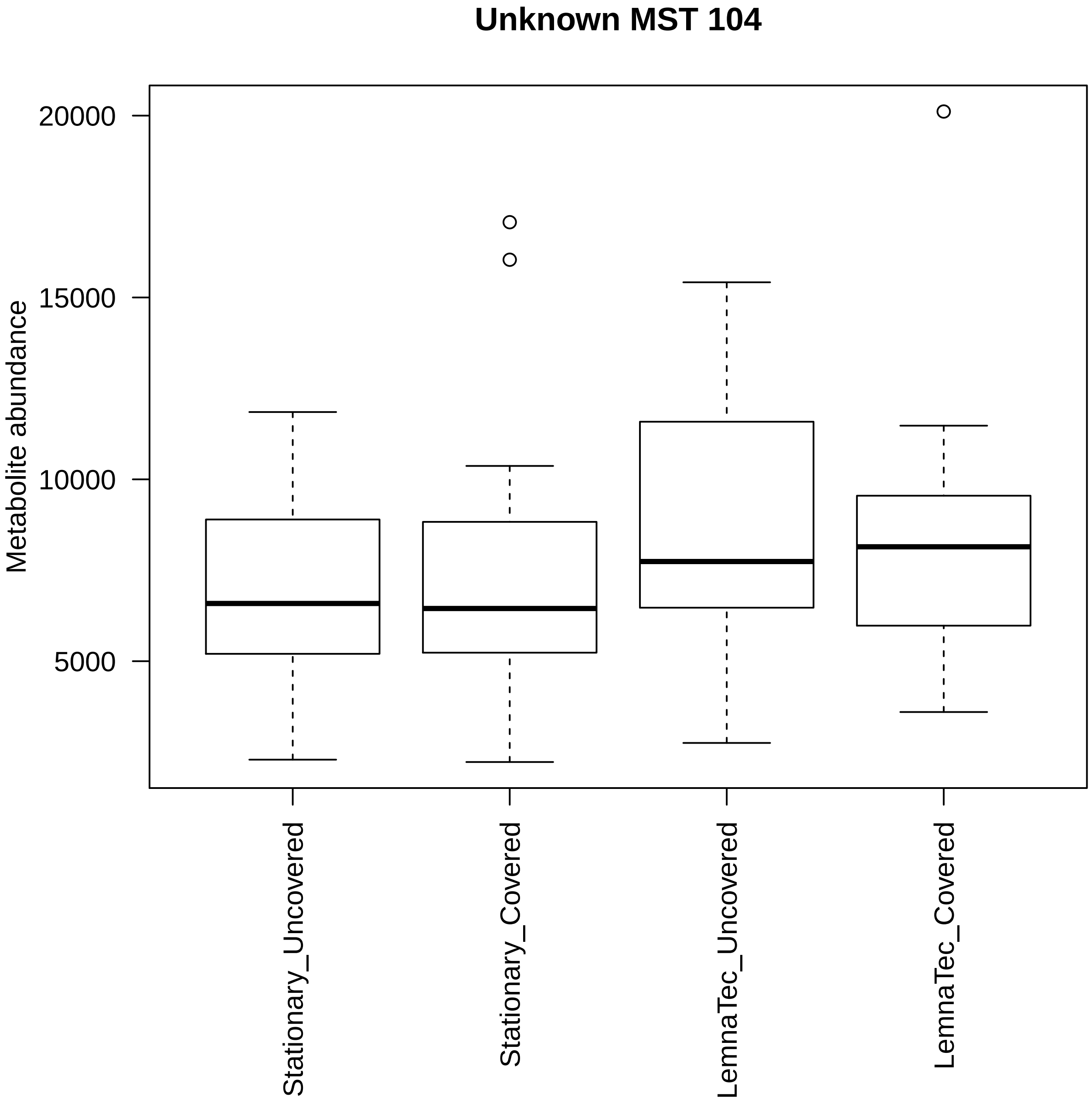
Unknown MST 104
Metabolite abundance
20000
15000
10000
5000
Stationary_Uncovered
Stationary_Covered
LemnaTec_Uncovered
LemnaTec_Covered

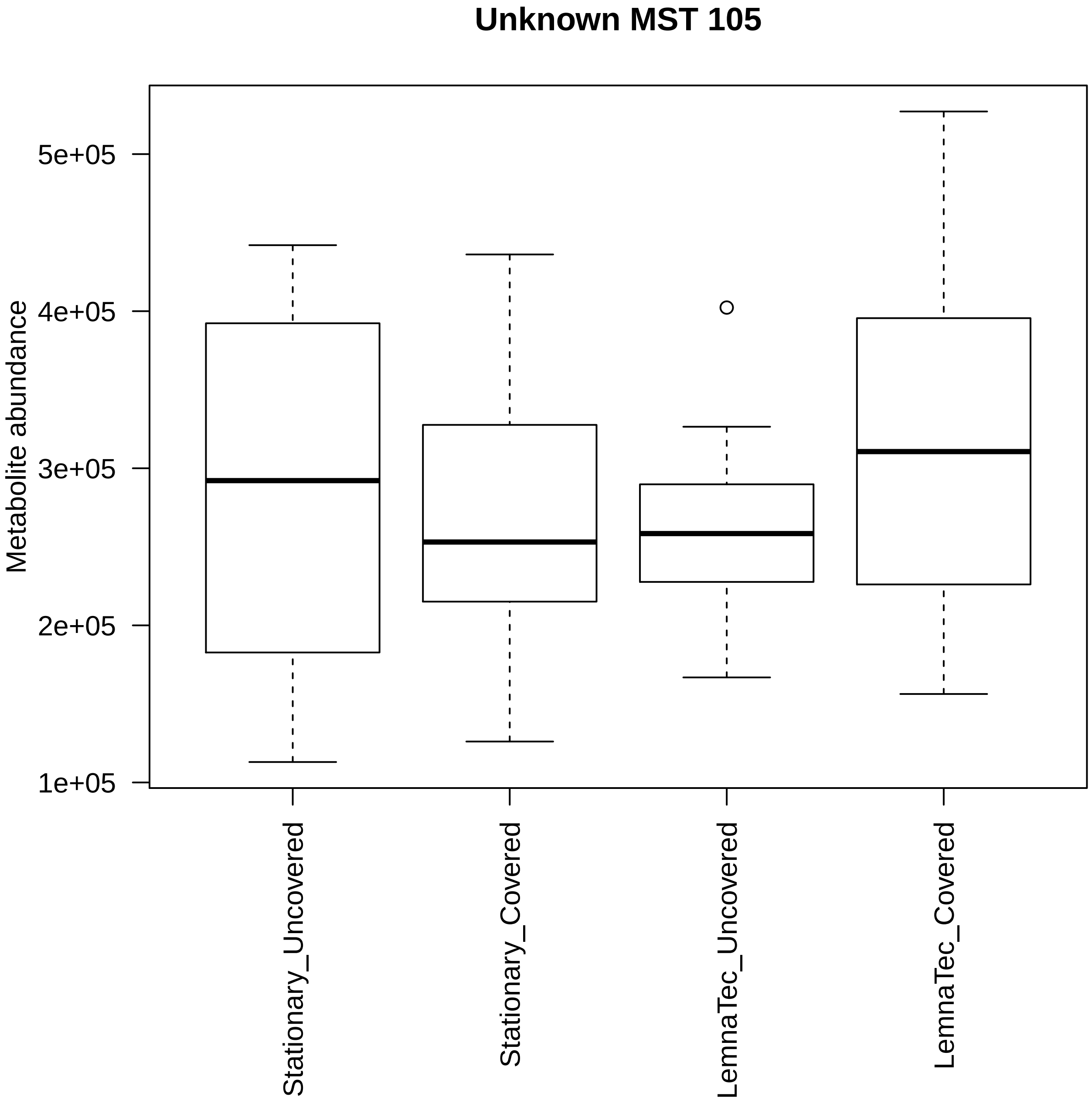

Unknown MST 105
Metabolite abundance
5e+05
4e+05
3e+05
2e+05
1e+05
Stationary_Uncovered
Stationary_Covered
LemnaTec_Uncovered
LemnaTec_Covered

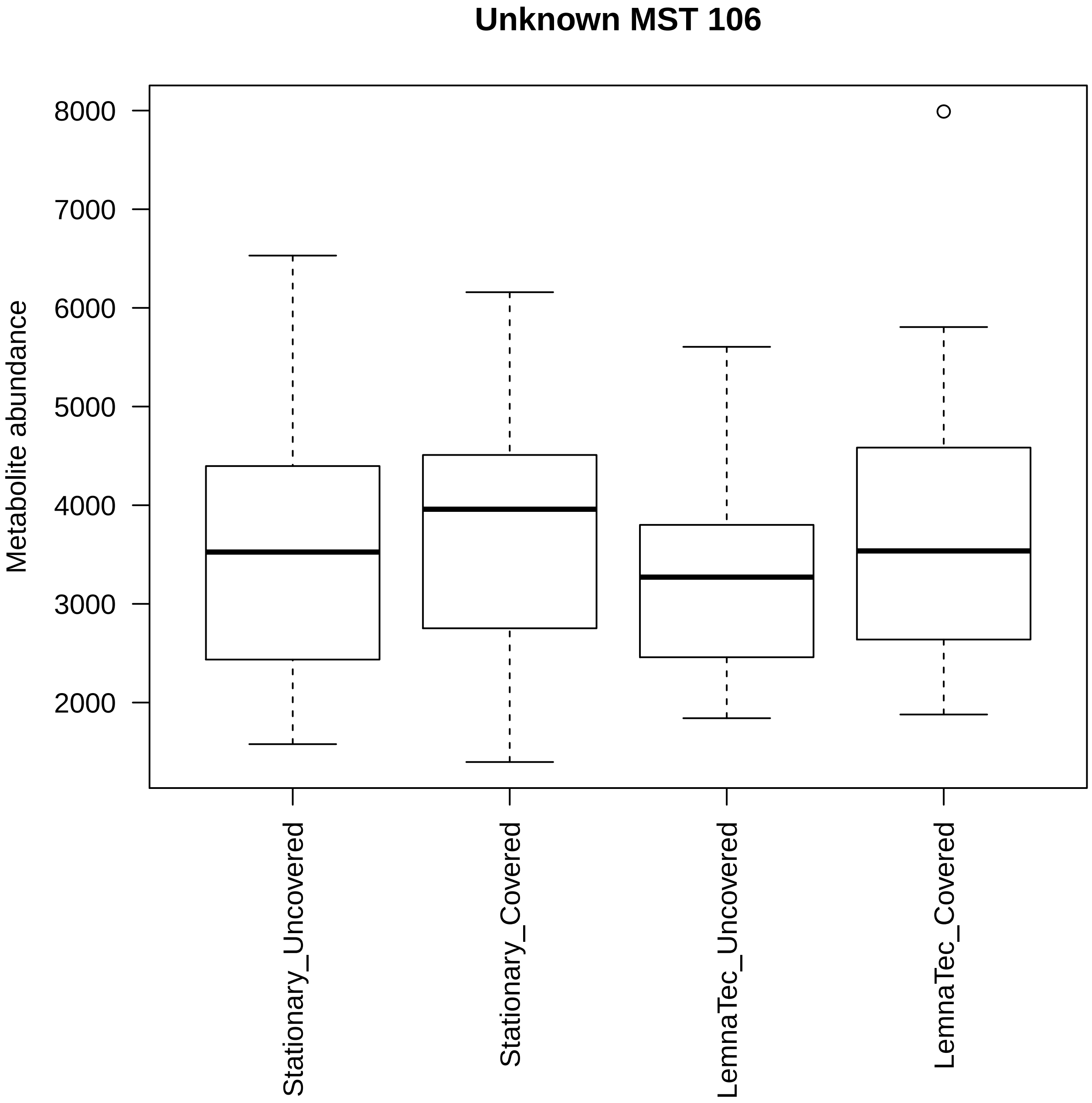
Unknown MST 106
Metabolite abundance
8000
7000
6000
5000
4000
3000
2000
Stationary_Uncovered
Stationary_Covered
LemnaTec_Uncovered
LemnaTec_Covered

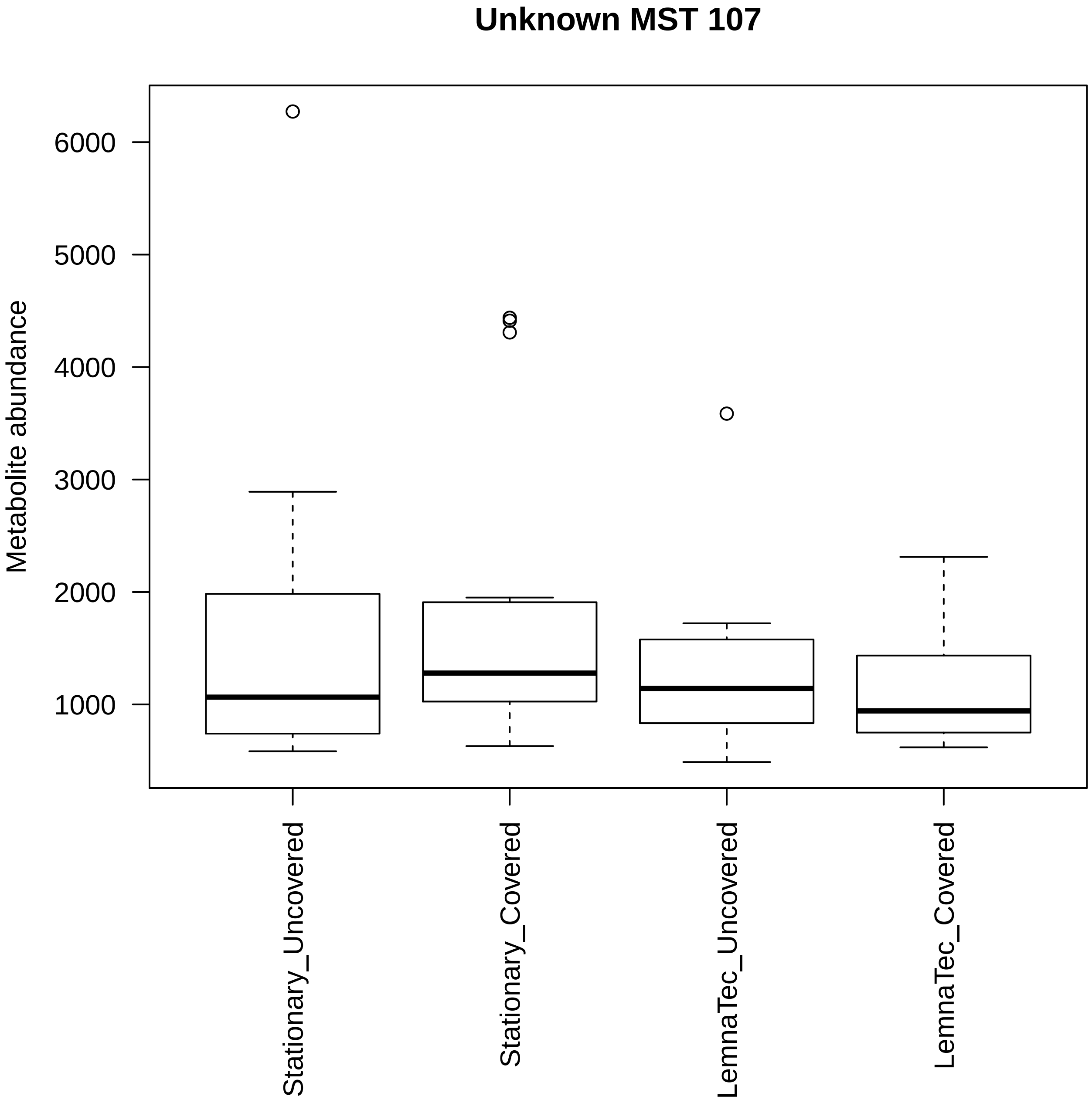

Unknown MST 107
Metabolite abundance
6000
5000
4000
3000
2000
1000
Stationary_Uncovered
Stationary_Covered
LemnaTec_Uncovered
LemnaTec_Covered

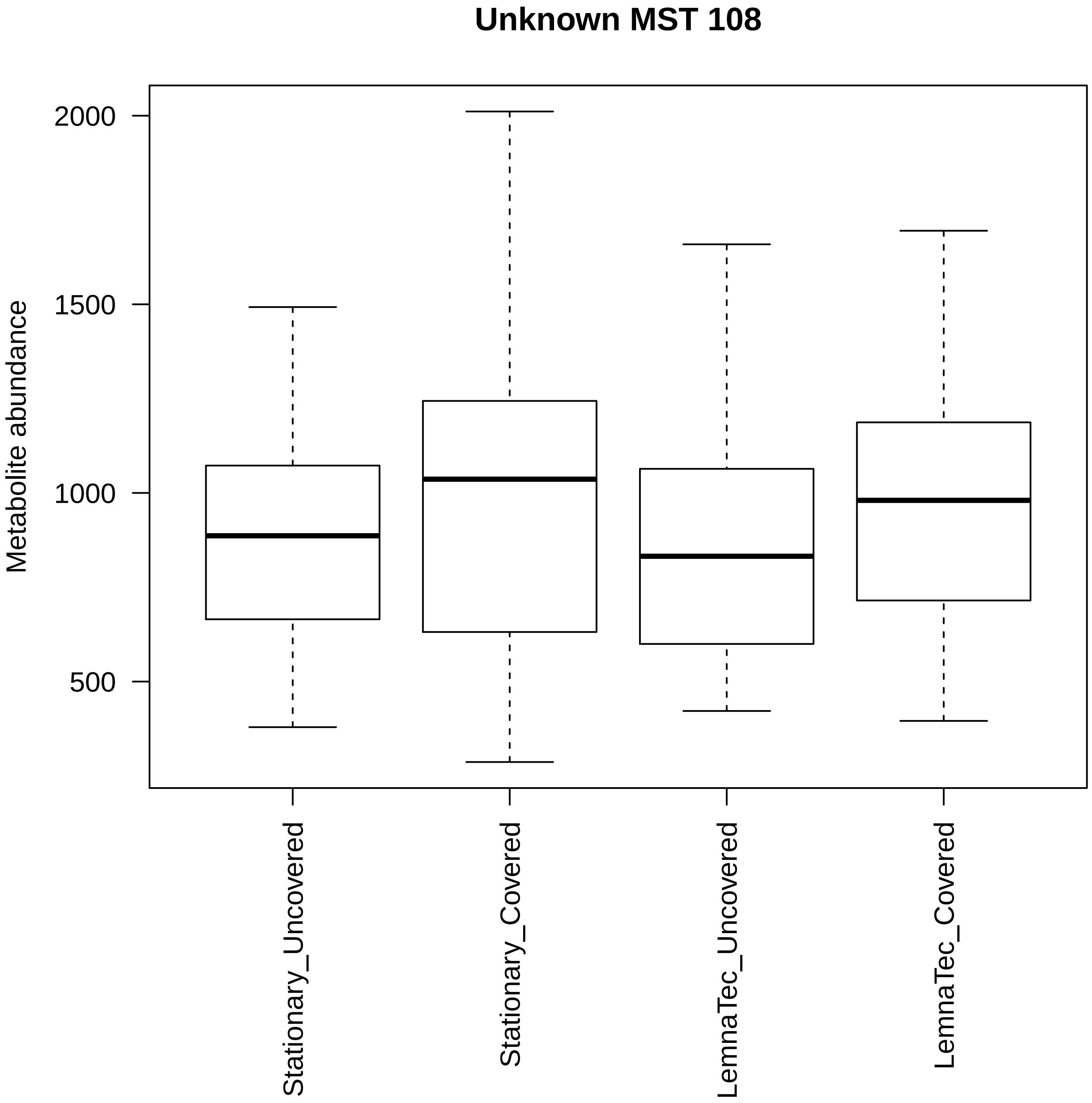

Unknown MST 108
Metabolite abundance
2000
1500
1000
500
Stationary_Uncovered
Stationary_Covered
LemnaTec_Uncovered
LemnaTec_Covered

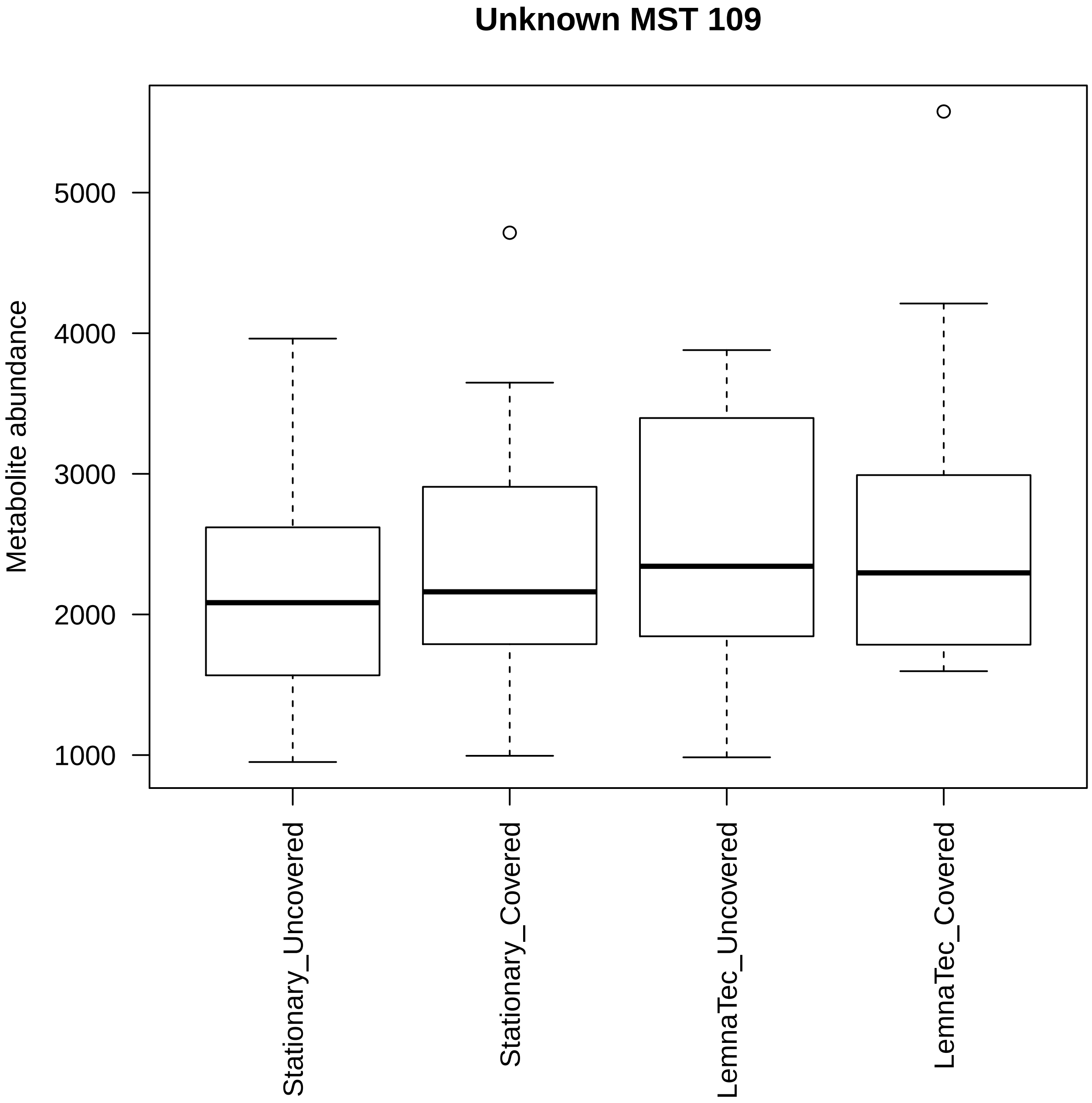
Unknown MST 109
Metabolite abundance
5000
4000
3000
2000
1000
Stationary_Uncovered
Stationary_Covered
LemnaTec_Uncovered
LemnaTec_Covered

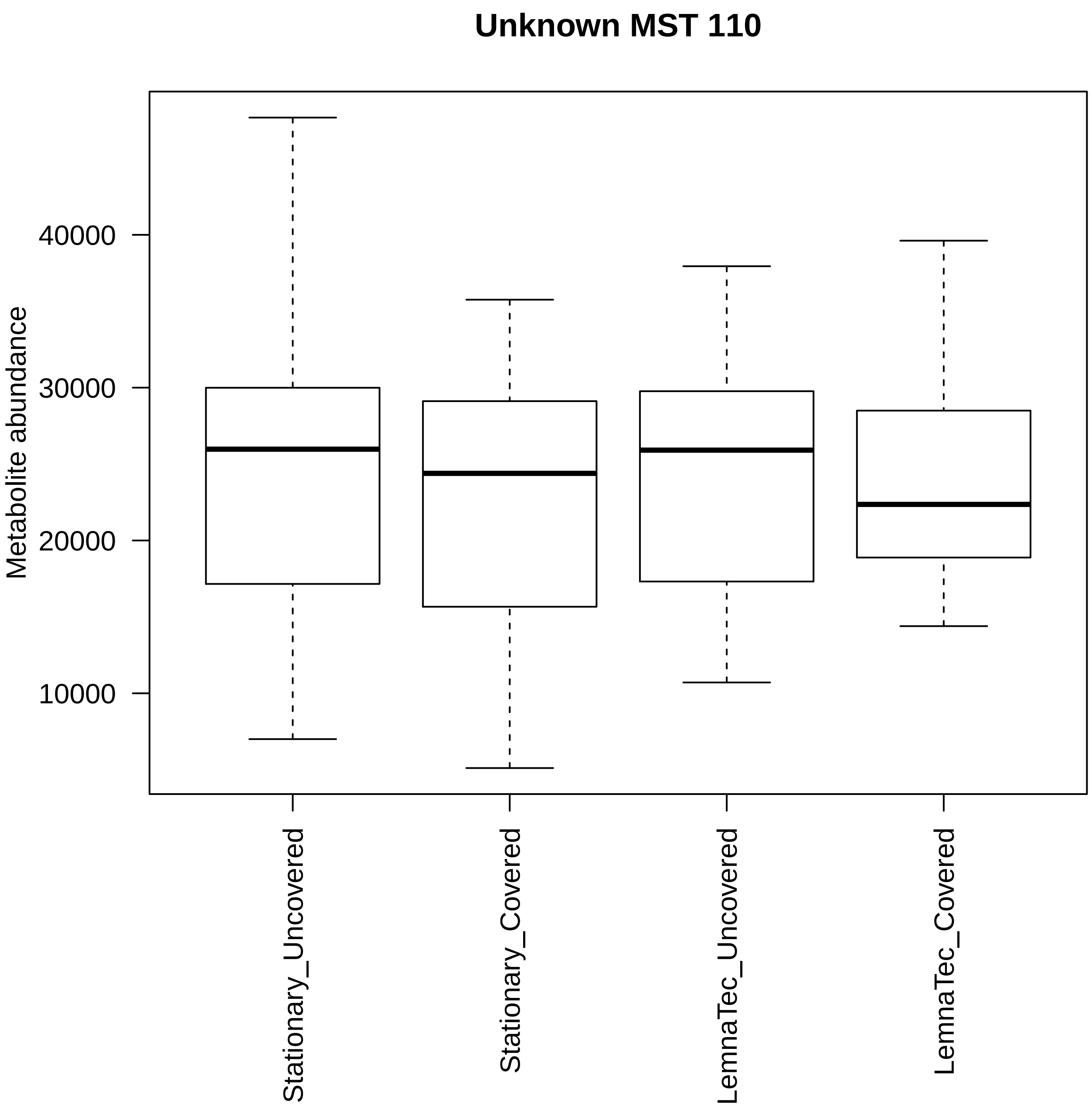
Unknown MST 110
Metabolite abundance
40000
30000
20000
10000
Stationary_Uncovered
Stationary_Covered
LemnaTec_Uncovered
LemnaTec_Covered

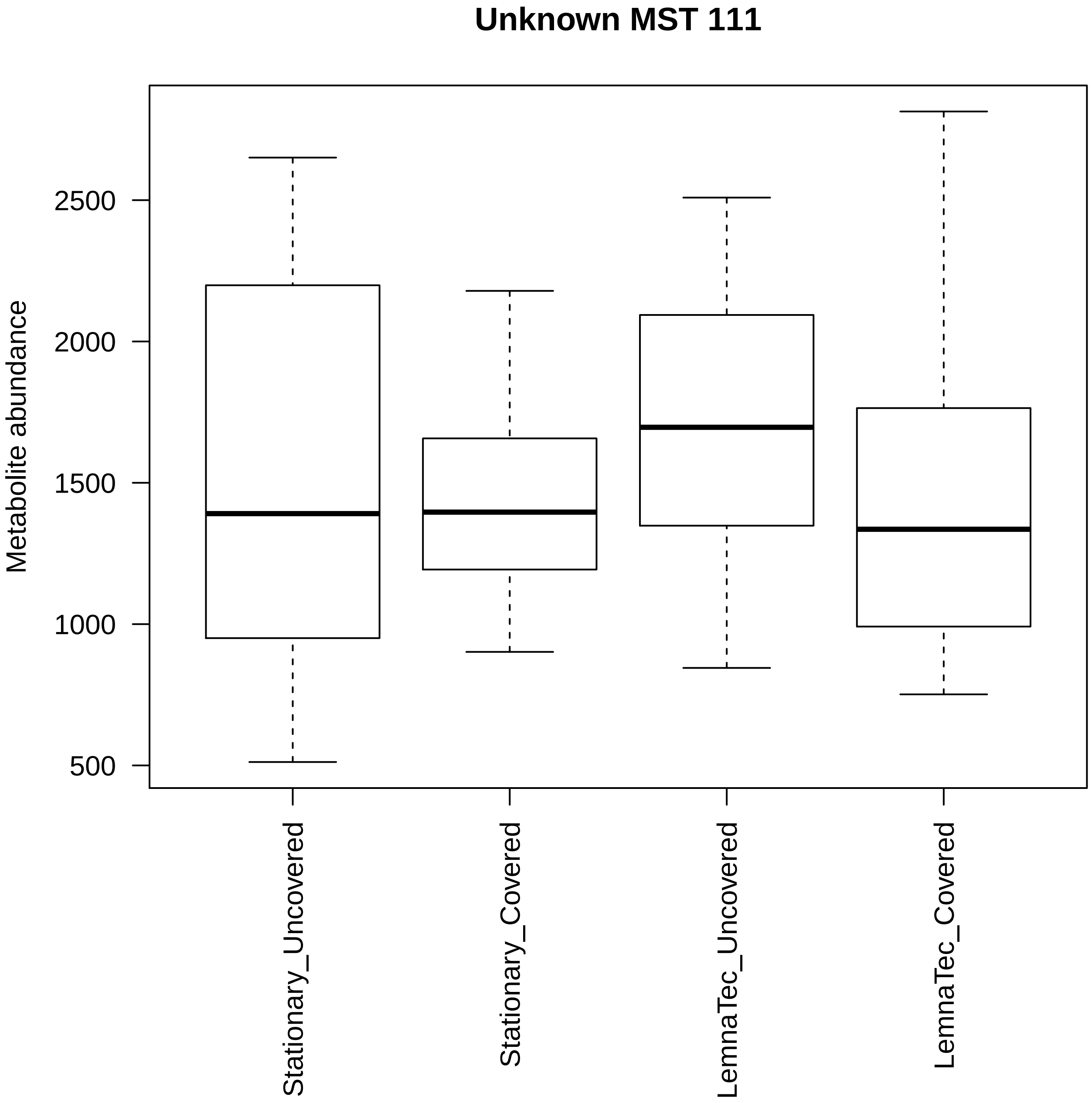

Unknown MST 111
Metabolite abundance
2500
2000
1500
1000
500
Stationary_Uncovered
Stationary_Covered
LemnaTec_Uncovered
LemnaTec_Covered

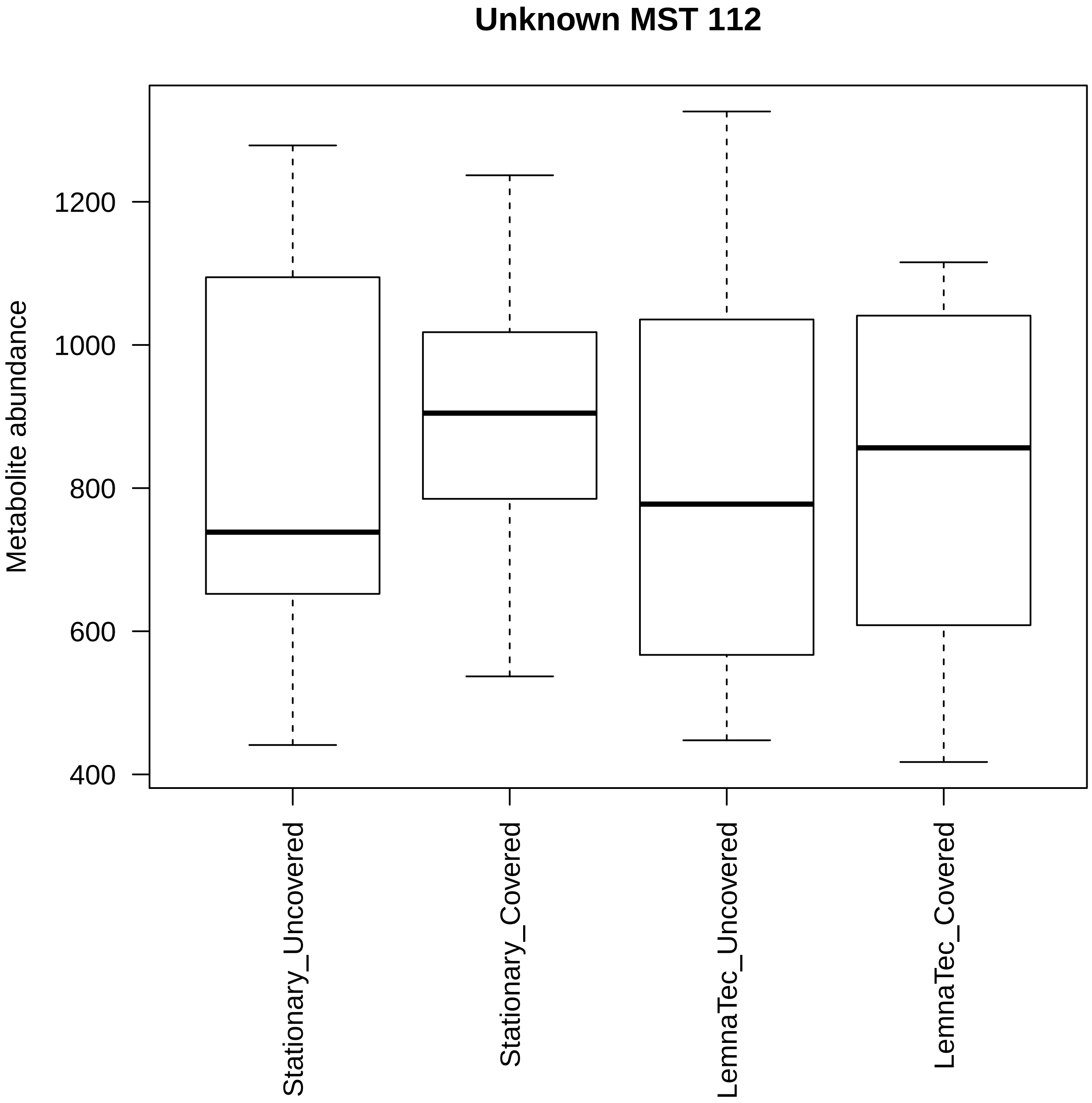

Unknown MST 112
Metabolite abundance
400
600
800
1000
1200
Stationary_Uncovered
Stationary_Covered
LemnaTec_Uncovered
LemnaTec_Covered

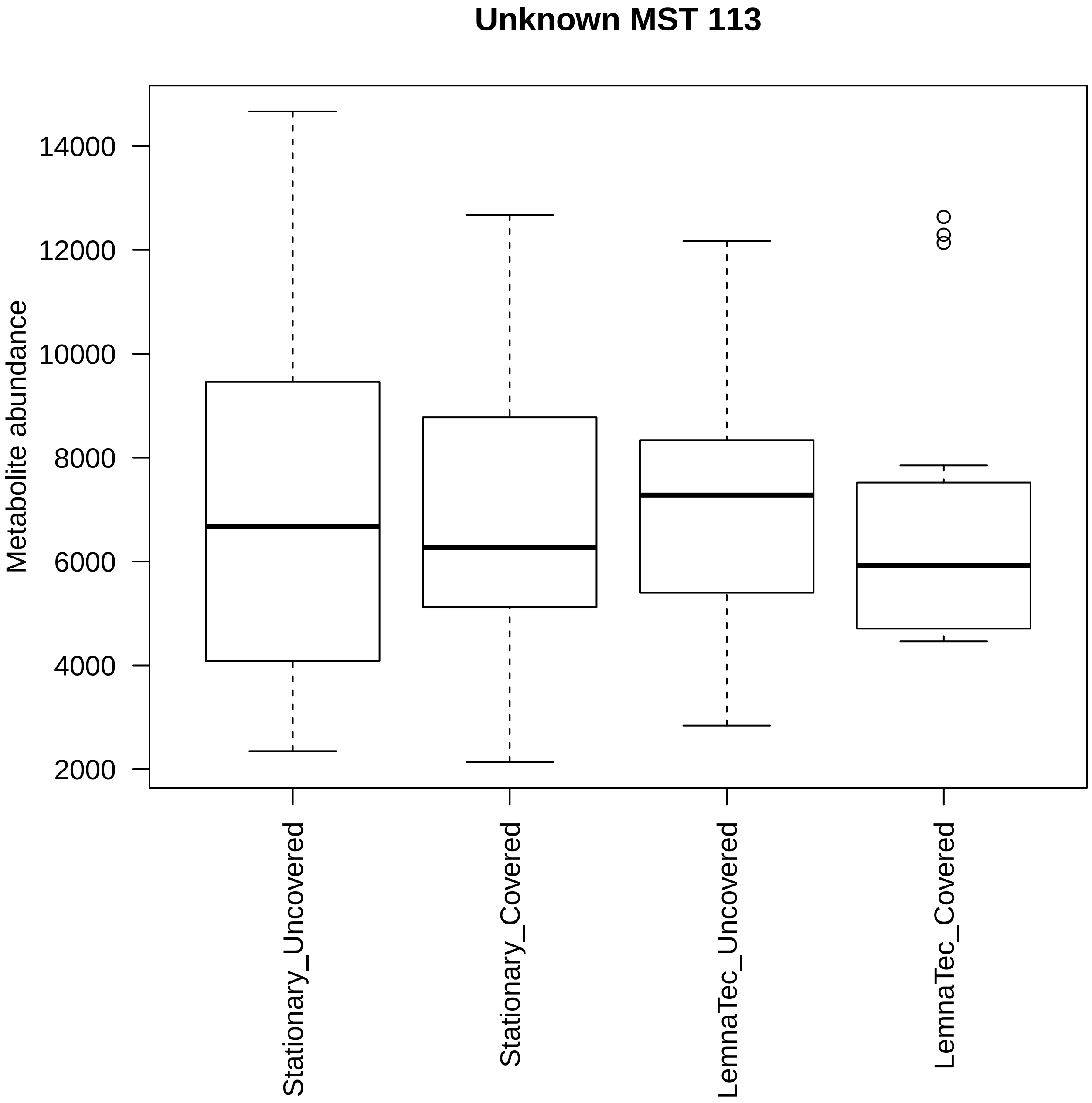
Unknown MST 113
Metabolite abundance
14000
12000
10000
8000
6000
4000
2000
Stationary_Uncovered
Stationary_Covered
LemnaTec_Uncovered
LemnaTec_Covered

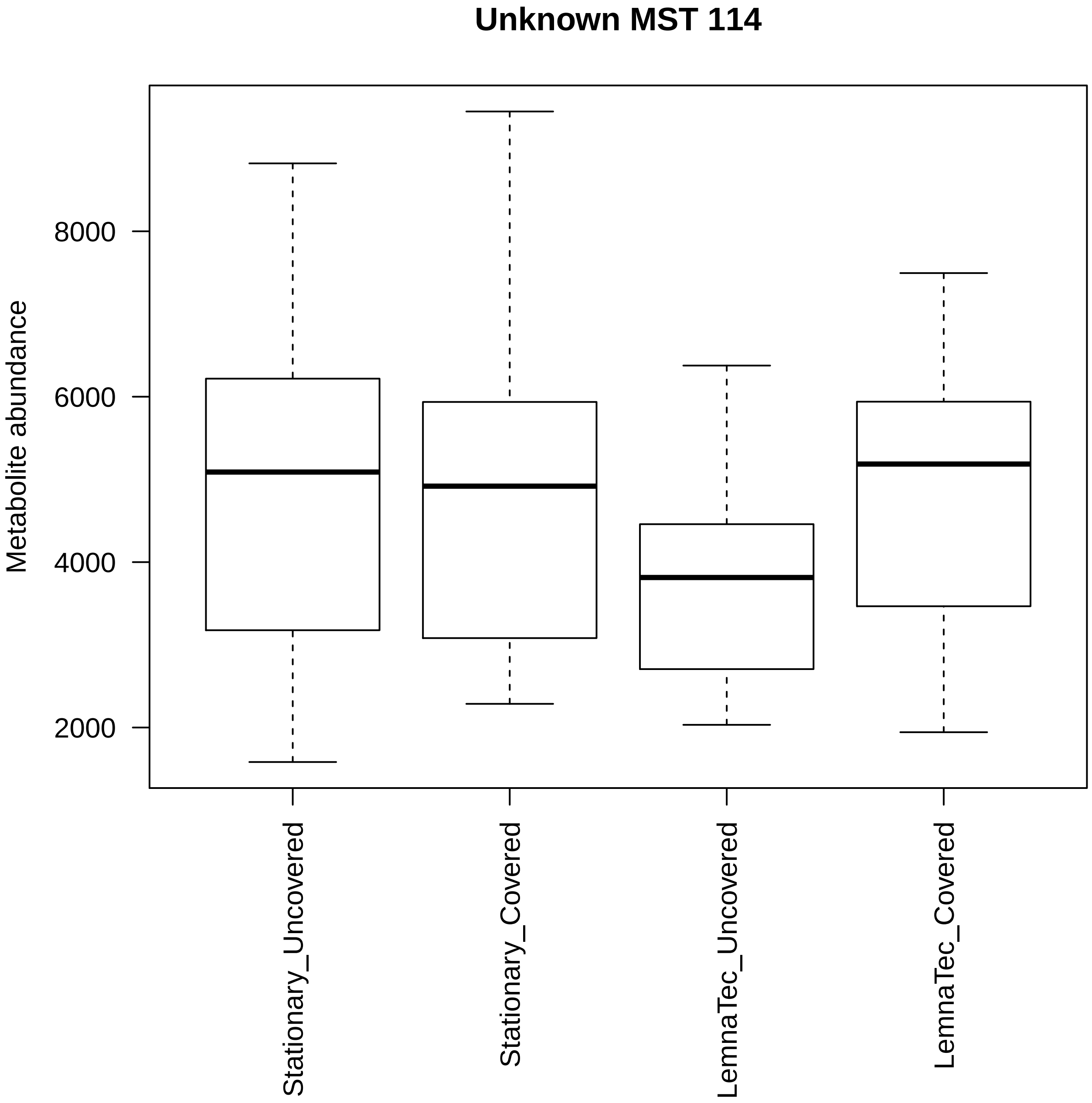

Unknown MST 114
Metabolite abundance
2000
4000
6000
8000
Stationary_Uncovered
Stationary_Covered
LemnaTec_Uncovered
LemnaTec_Covered

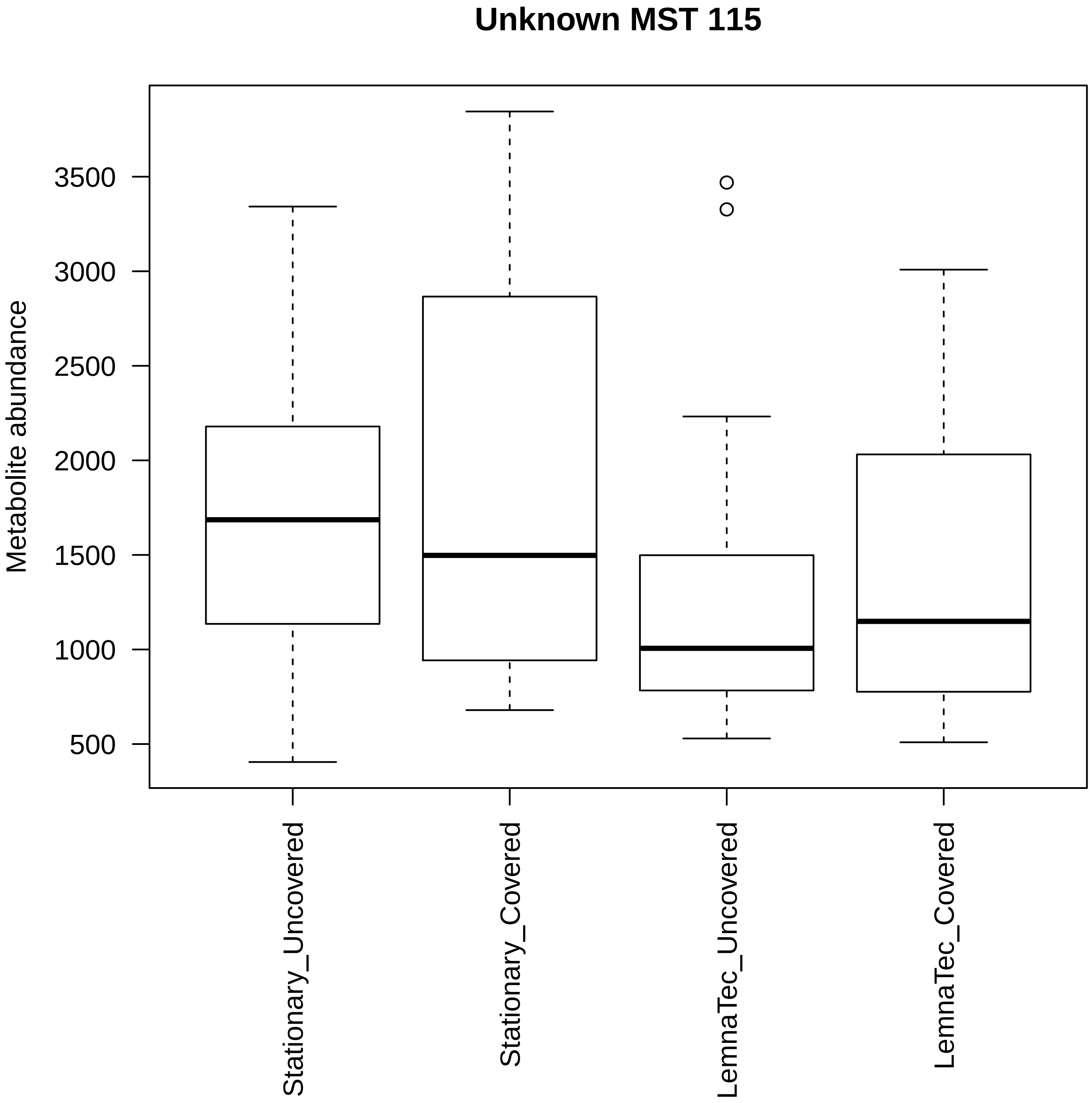

Unknown MST 115
Metabolite abundance
500
1000
1500
2000
2500
3000
3500
Stationary_Uncovered
Stationary_Covered
LemnaTec_Uncovered
LemnaTec_Covered

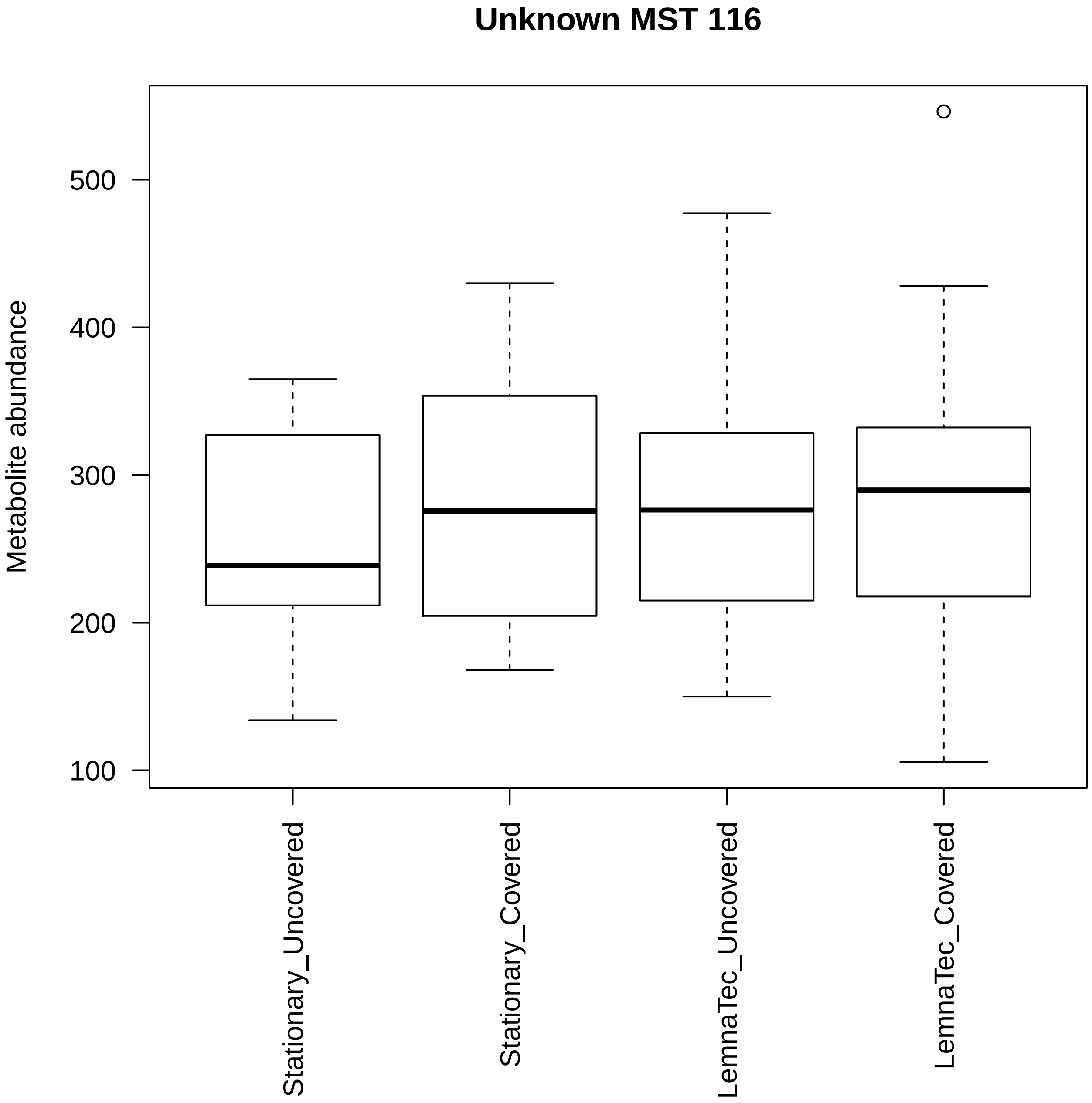
Unknown MST 116
Metabolite abundance
100
200
300
400
500
Stationary_Uncovered
Stationary_Covered
LemnaTec_Uncovered
LemnaTec_Covered

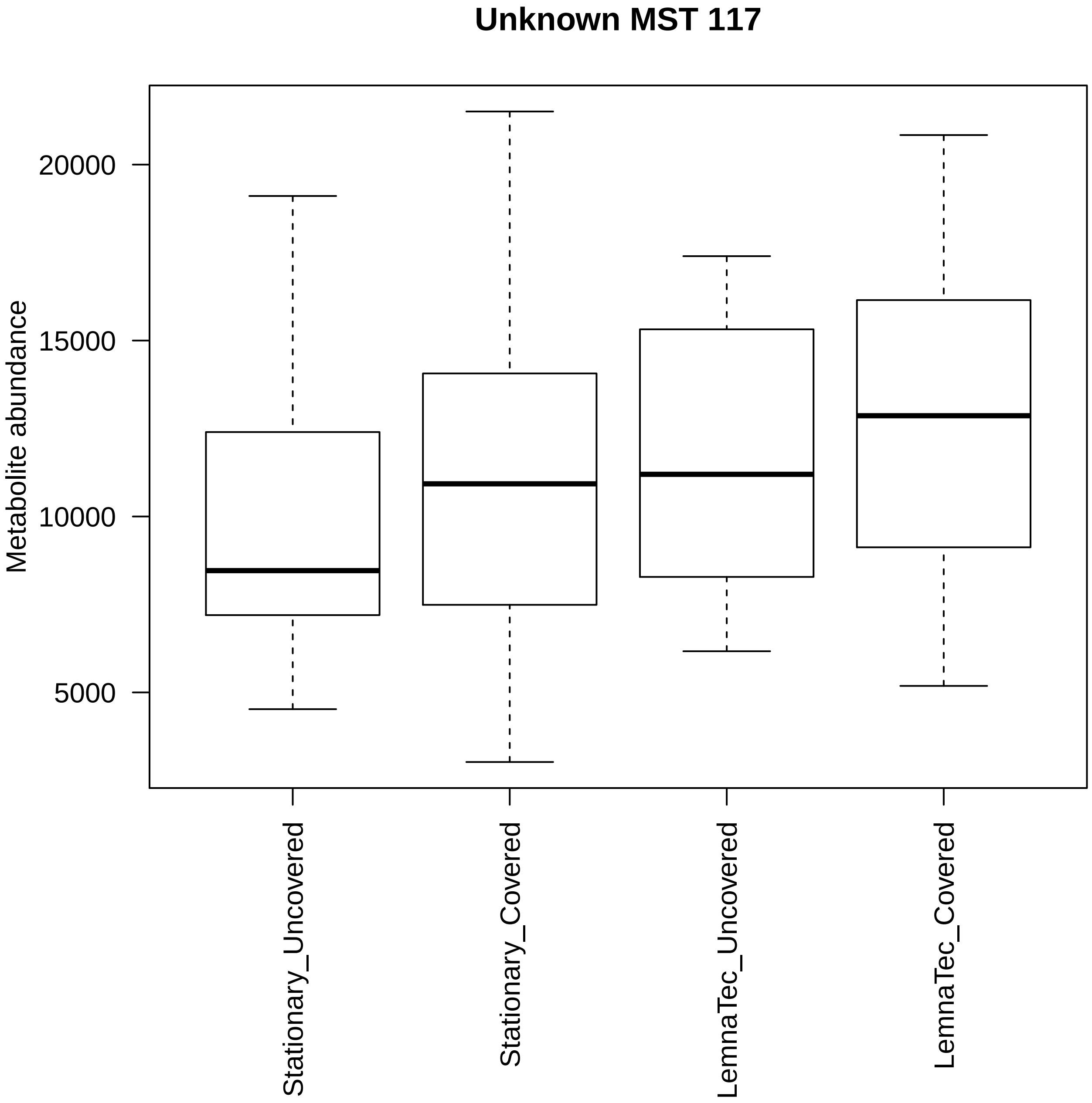

Unknown MST 117
Metabolite abundance
20000
15000
10000
5000
Stationary_Uncovered
Stationary_Covered
LemnaTec_Uncovered
LemnaTec_Covered

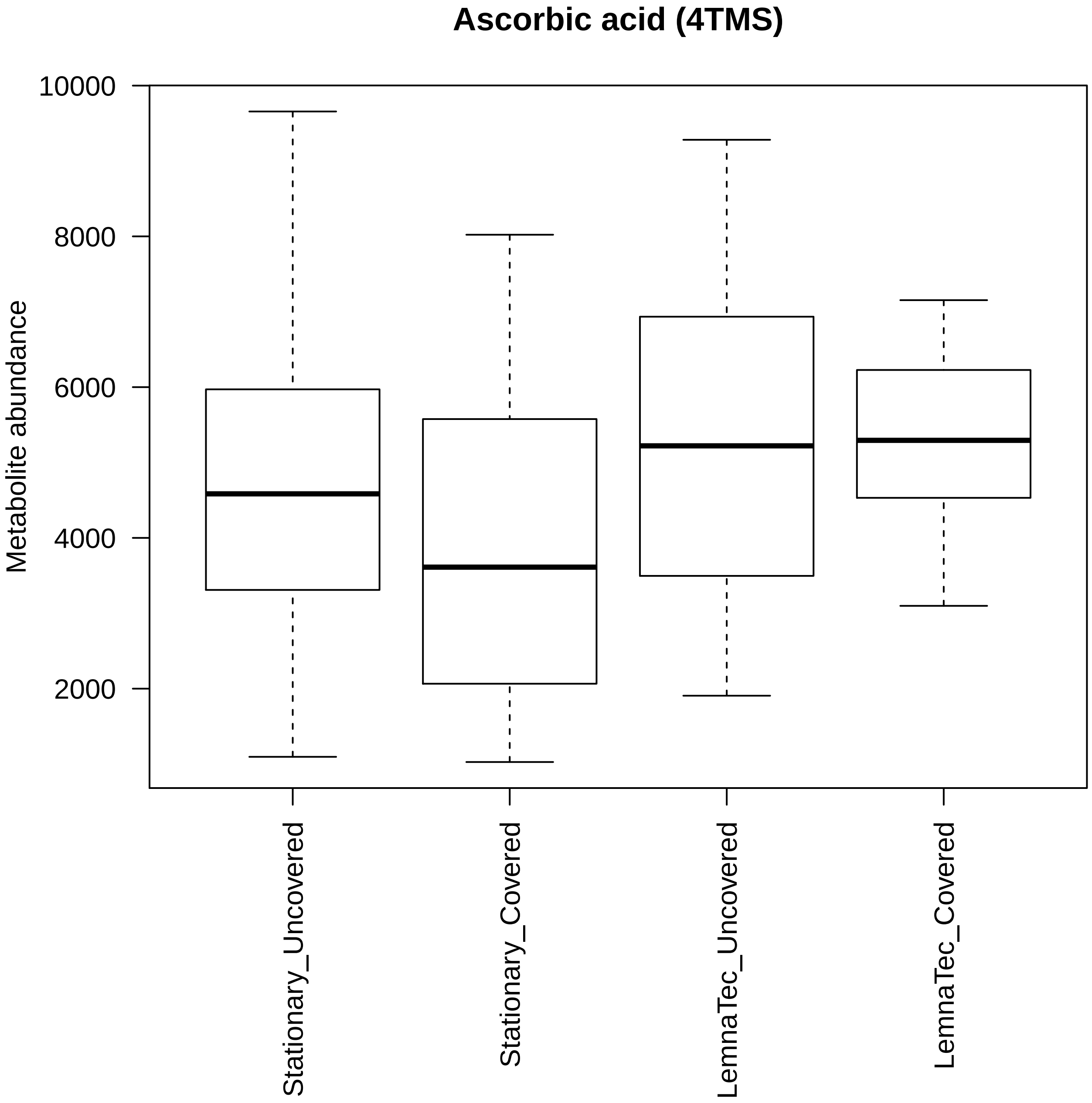
Ascorbic acid (4TMS)
Metabolite abundance
10000
8000
6000
4000
2000
Stationary_Uncovered
Stationary_Covered
LemnaTec_Uncovered
LemnaTec_Covered

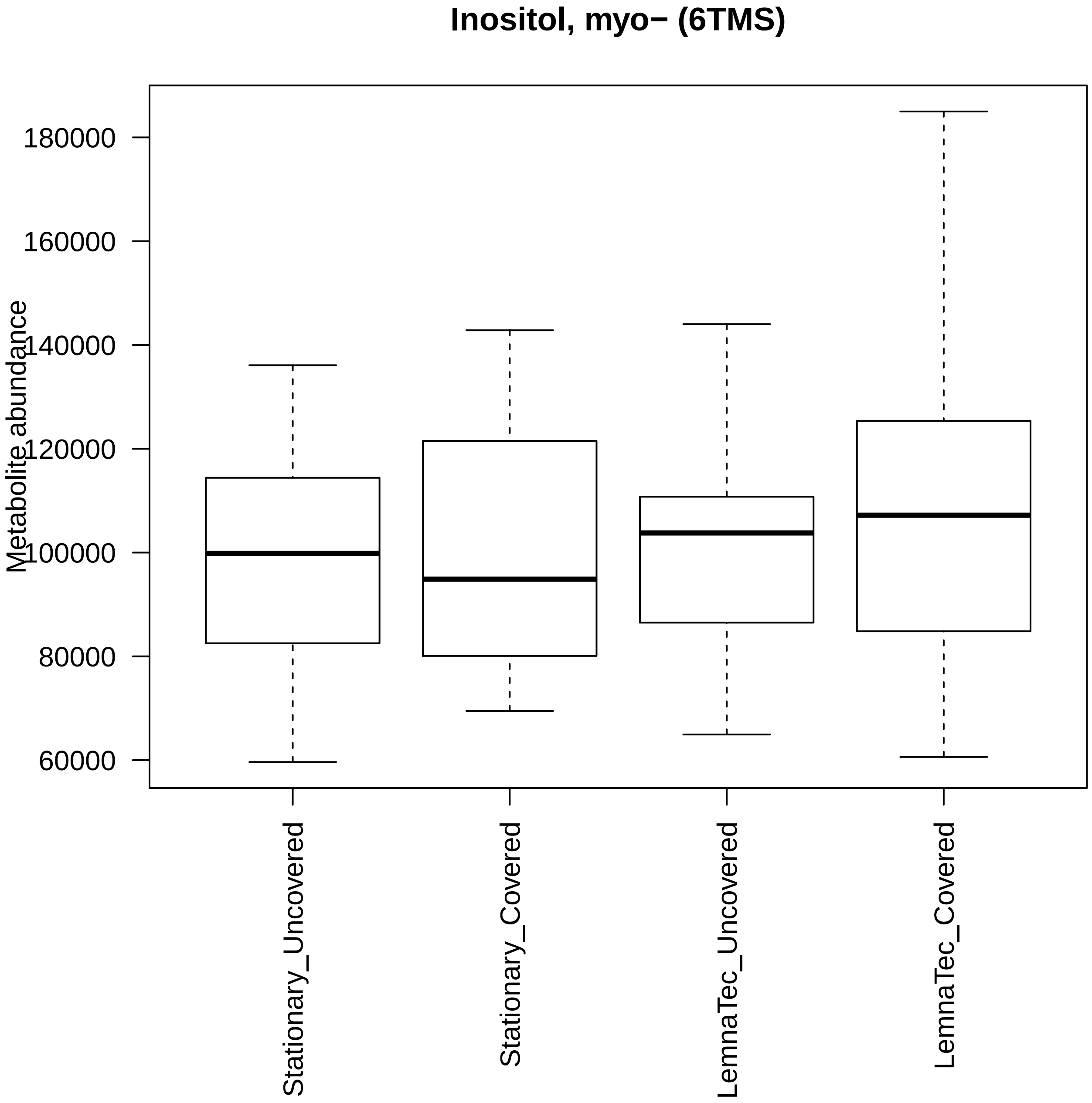

Inositol, myo− (6TMS)
Metabolite abundance
180000
160000
140000
120000
100000
80000
60000
Stationary_Uncovered
Stationary_Covered
LemnaTec_Uncovered
LemnaTec_Covered

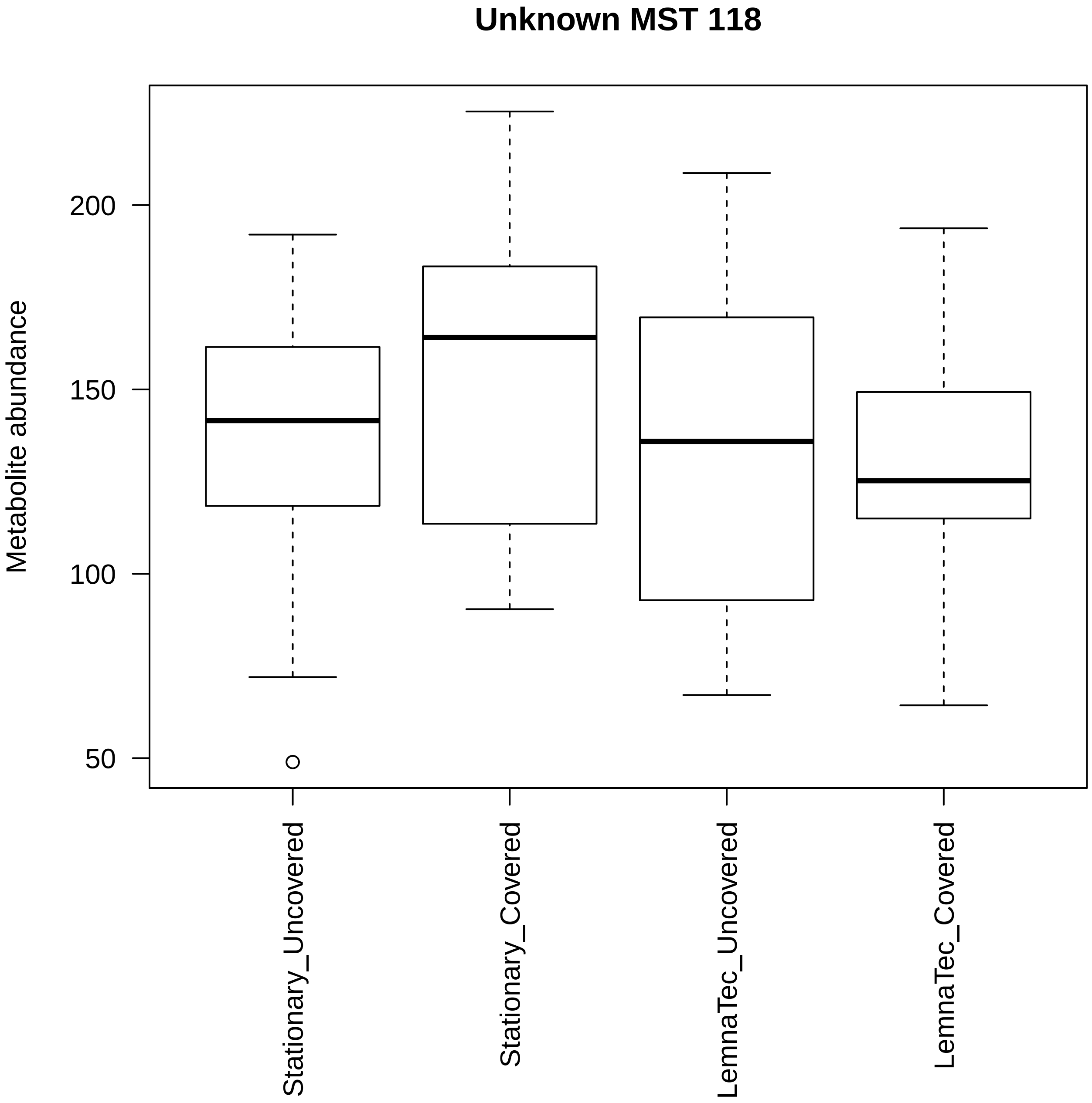

Unknown MST 118
Metabolite abundance
200
150
100
50
Stationary_Uncovered
Stationary_Covered
LemnaTec_Uncovered
LemnaTec_Covered

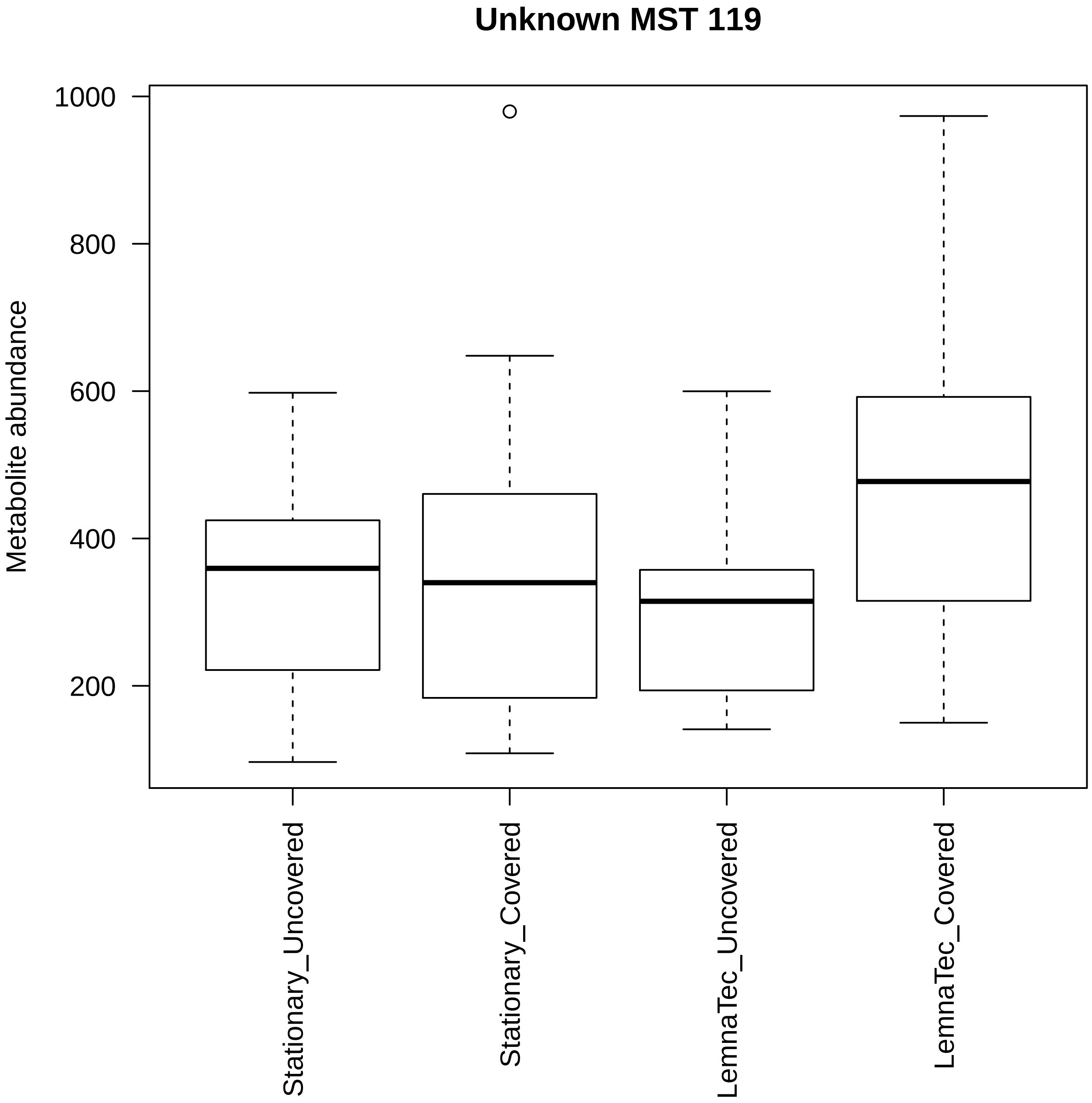

Unknown MST 119
Metabolite abundance
1000
800
600
400
200
Stationary_Uncovered
Stationary_Covered
LemnaTec_Uncovered
LemnaTec_Covered

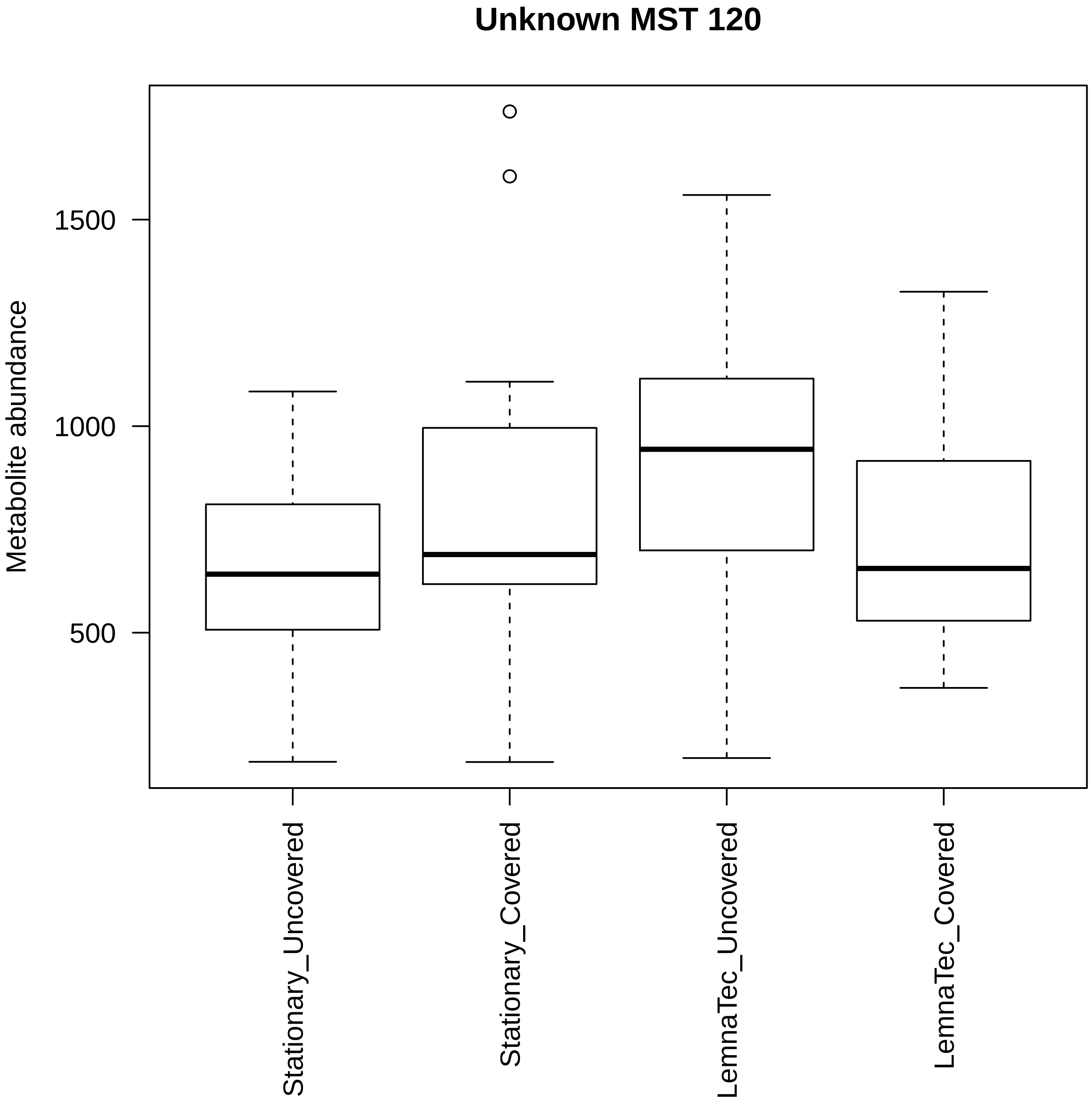
Unknown MST 120
Metabolite abundance
1500
1000
500
Stationary_Uncovered
Stationary_Covered
LemnaTec_Uncovered
LemnaTec_Covered

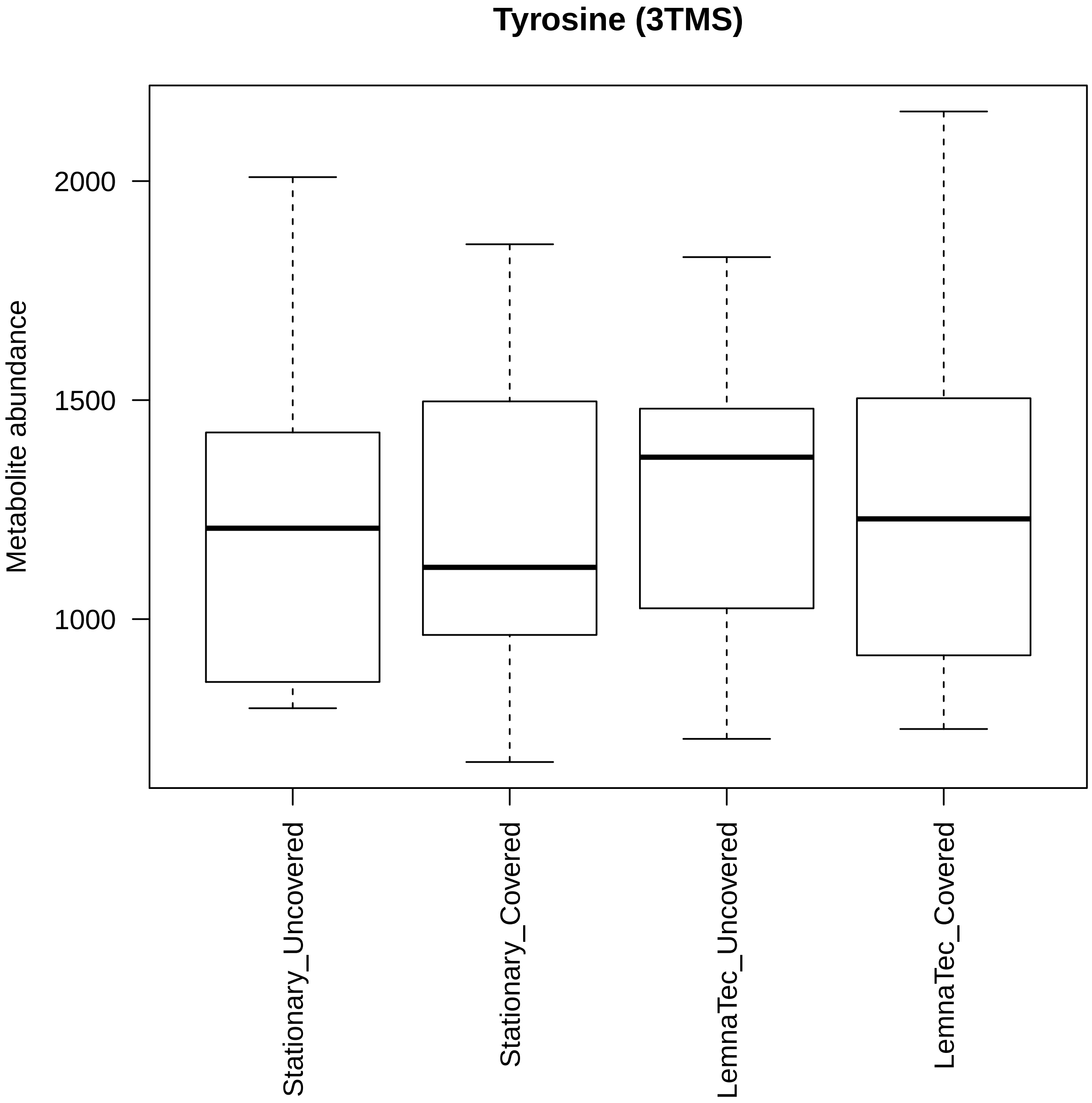
Tyrosine (3TMS)
Metabolite abundance
2000
1500
1000
Stationary_Uncovered
Stationary_Covered
LemnaTec_Uncovered
LemnaTec_Covered

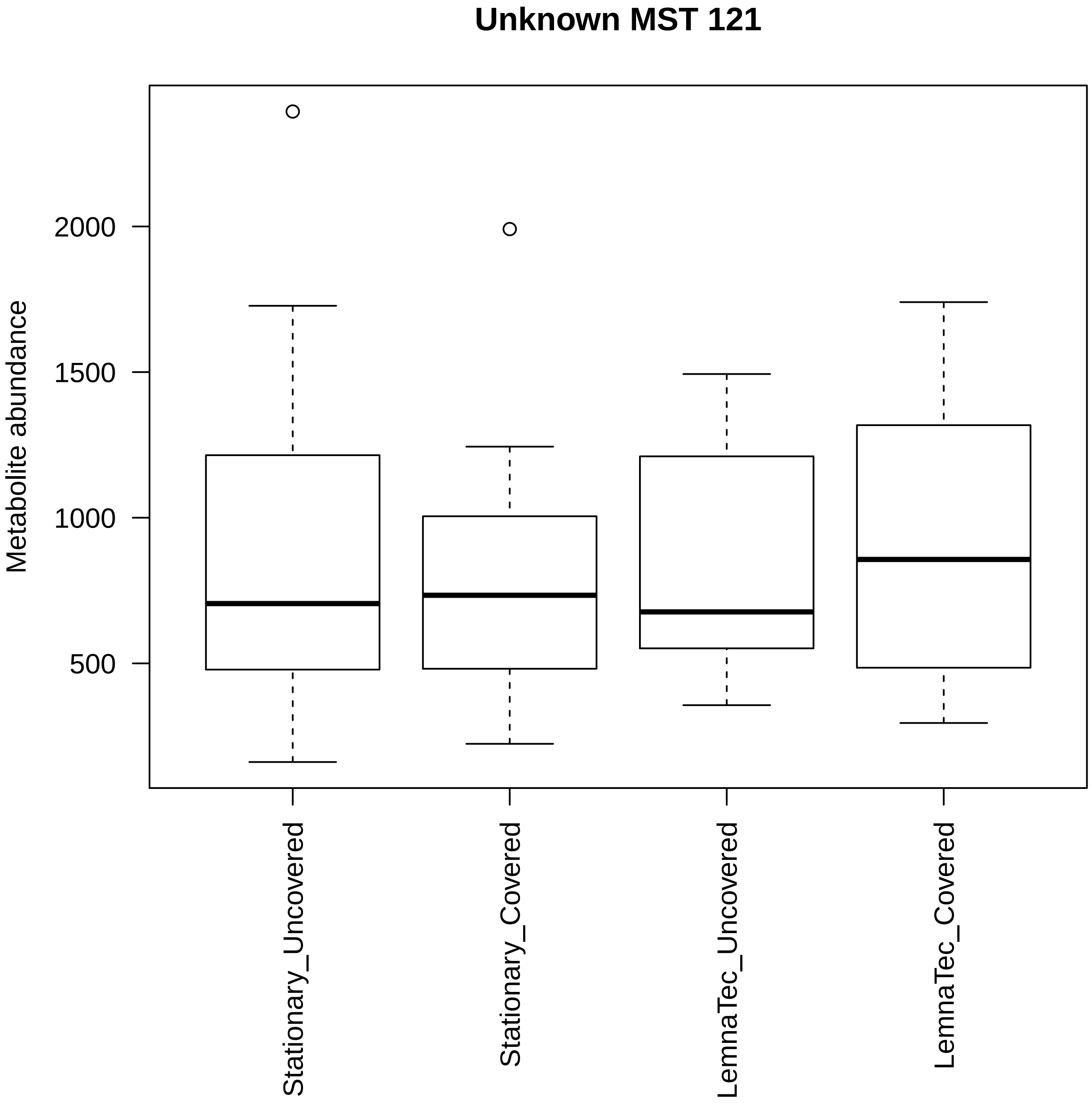
Unknown MST 121
Metabolite abundance
2000
1500
1000
500
Stationary_Uncovered
Stationary_Covered
LemnaTec_Uncovered
LemnaTec_Covered

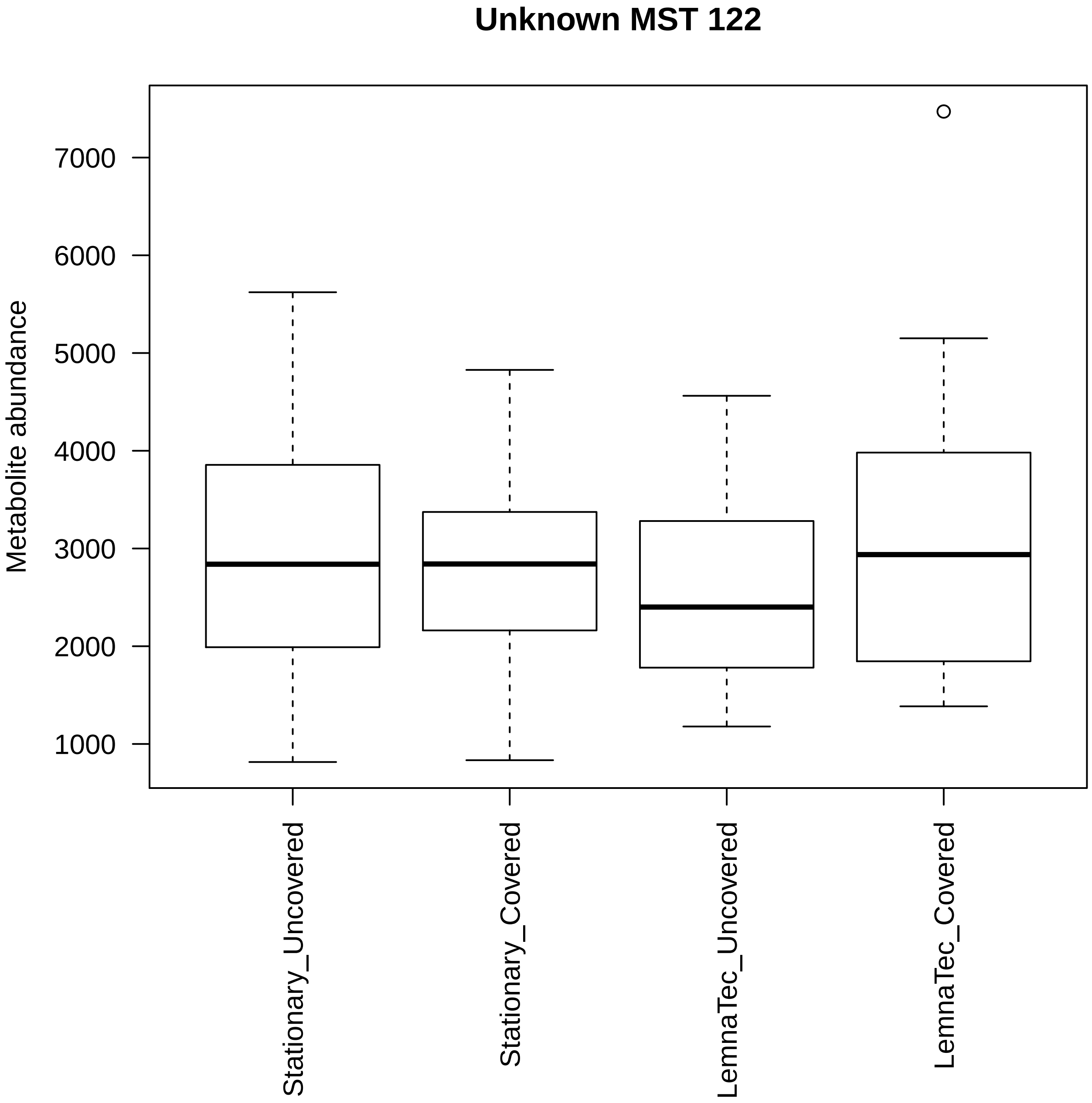
Unknown MST 122
Metabolite abundance
7000
6000
5000
4000
3000
2000
1000
Stationary_Uncovered
Stationary_Covered
LemnaTec_Uncovered
LemnaTec_Covered

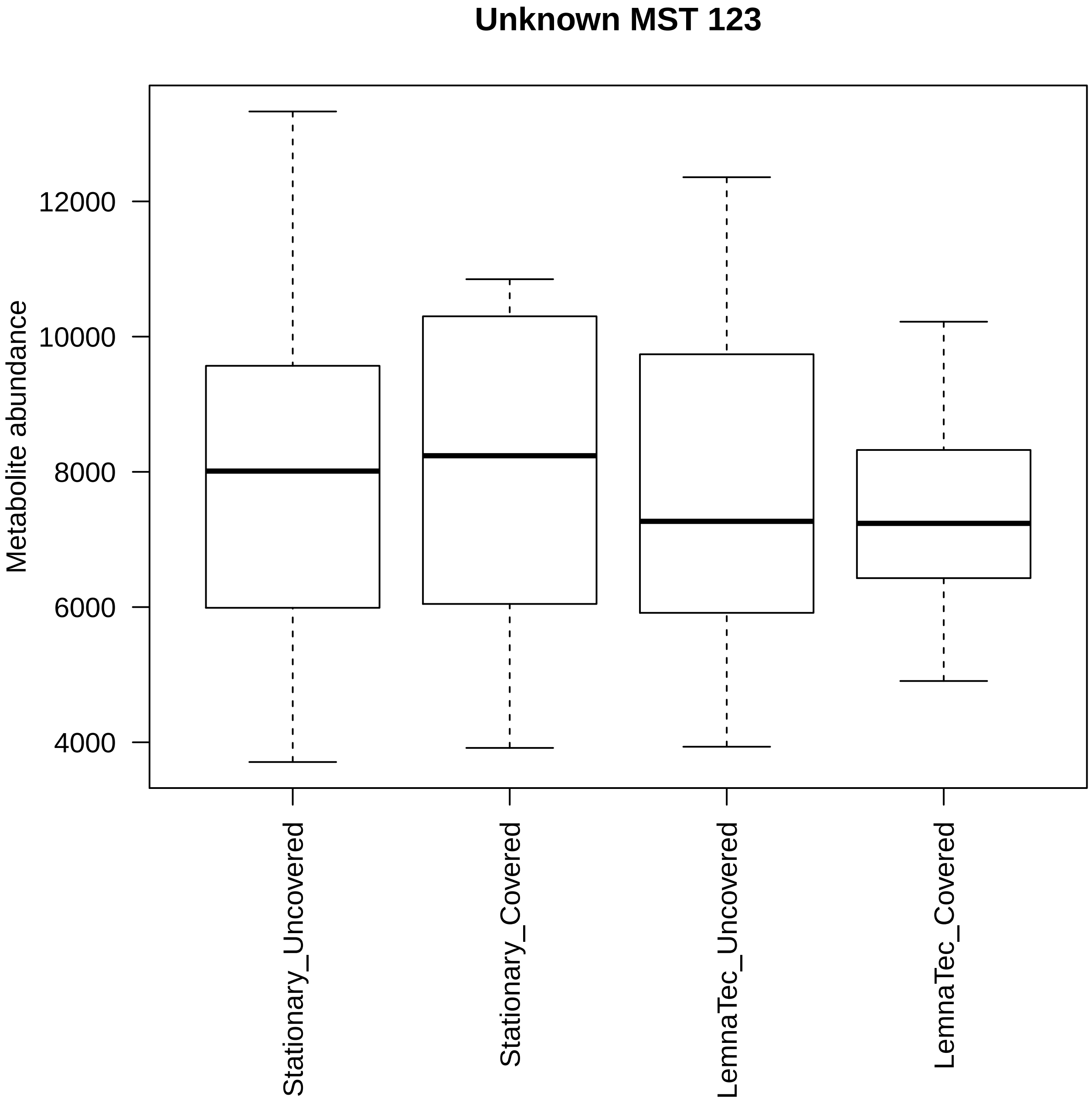

Unknown MST 123
Metabolite abundance
12000
10000
8000
6000
4000
Stationary_Uncovered
Stationary_Covered
LemnaTec_Uncovered
LemnaTec_Covered

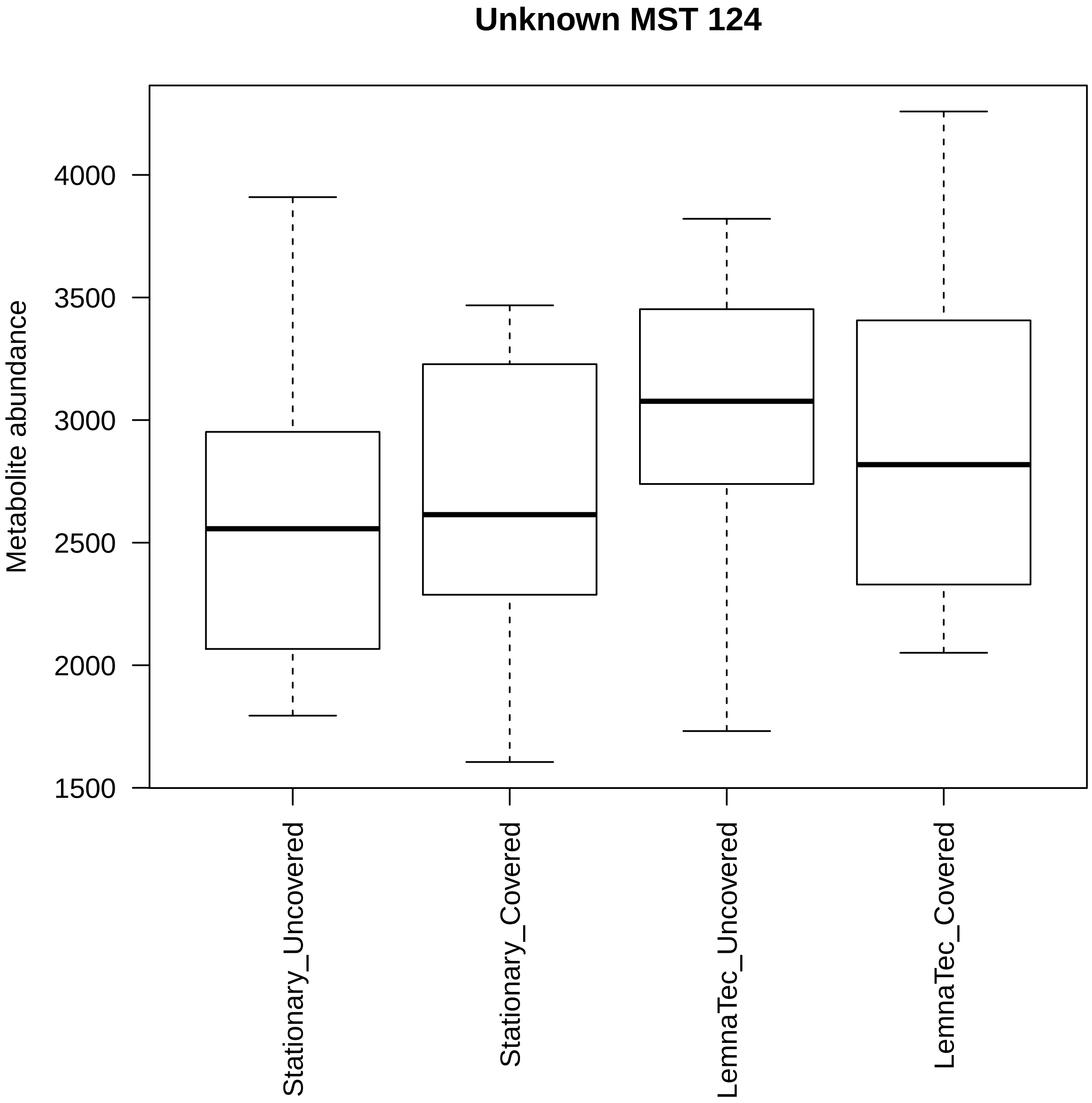
Unknown MST 124
Metabolite abundance
1500
2000
2500
3000
3500
4000
Stationary_Uncovered
Stationary_Covered
LemnaTec_Uncovered
LemnaTec_Covered

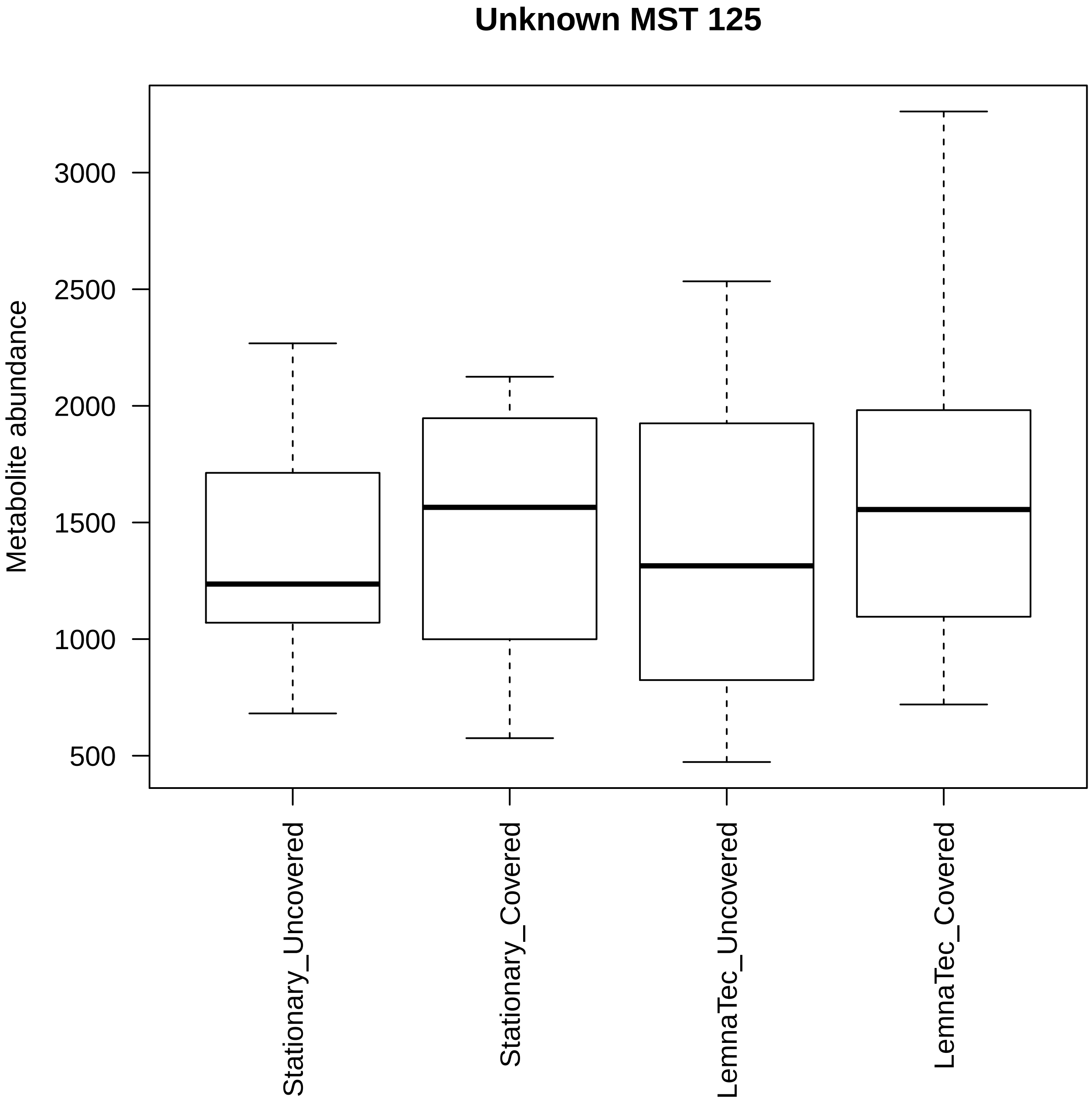

Unknown MST 125
Metabolite abundance
3000
2500
2000
1500
1000
500
Stationary_Uncovered
Stationary_Covered
LemnaTec_Uncovered
LemnaTec_Covered

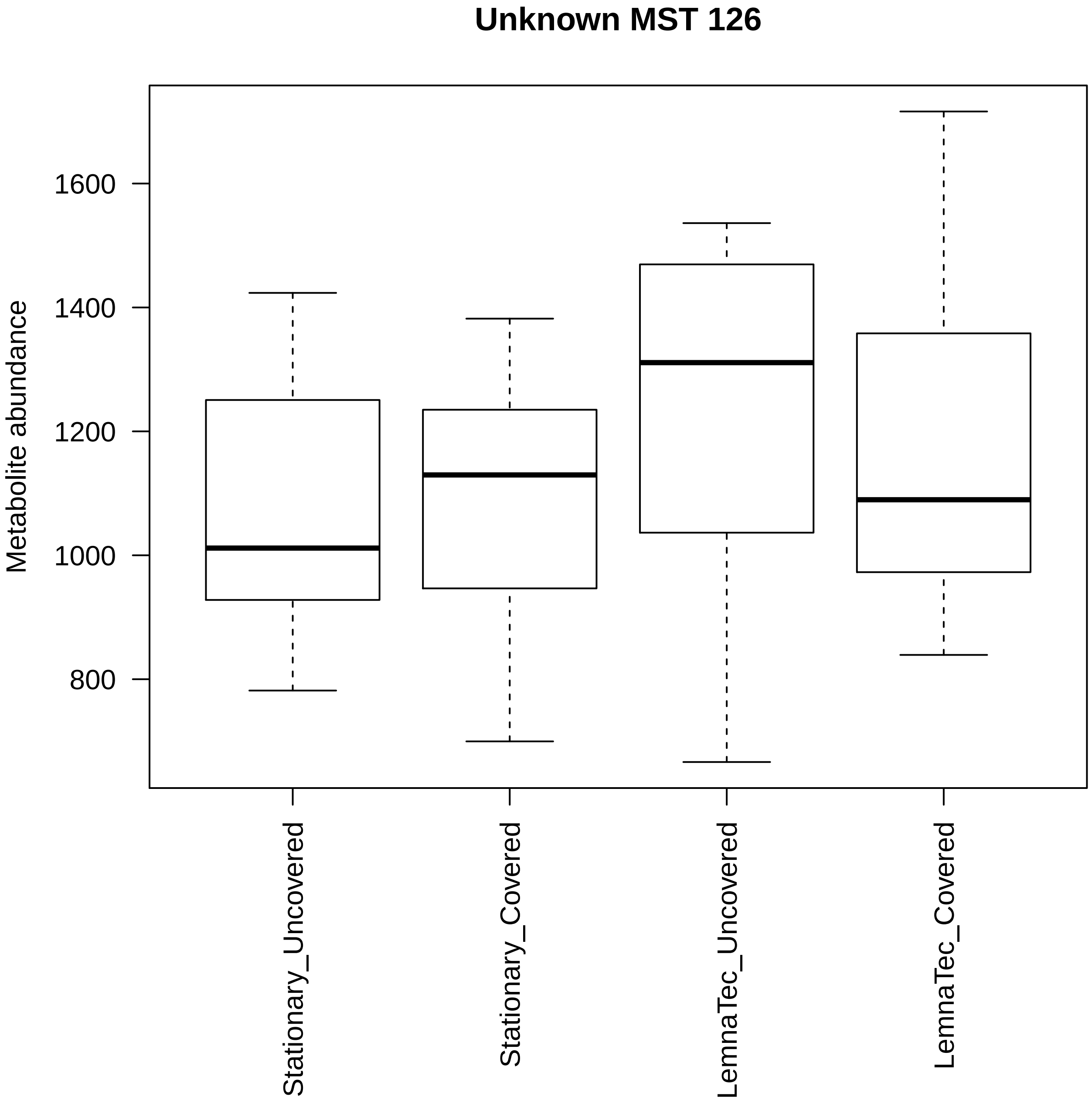

Unknown MST 126
Metabolite abundance
800
1000
1200
1400
1600
Stationary_Uncovered
Stationary_Covered
LemnaTec_Uncovered
LemnaTec_Covered

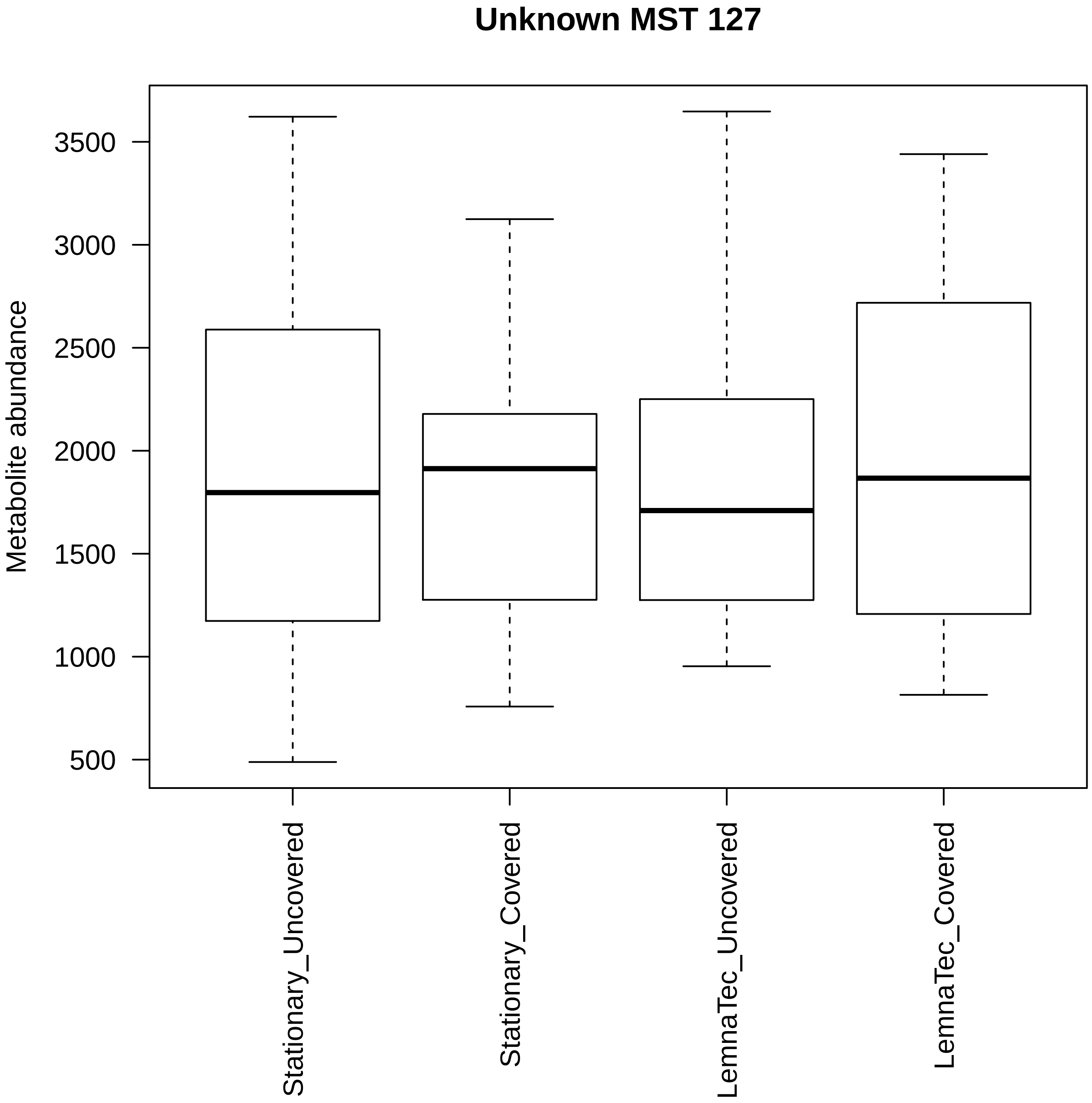

Unknown MST 127
Metabolite abundance
3500
3000
2500
2000
1500
1000
500
Stationary_Uncovered
Stationary_Covered
LemnaTec_Uncovered
LemnaTec_Covered

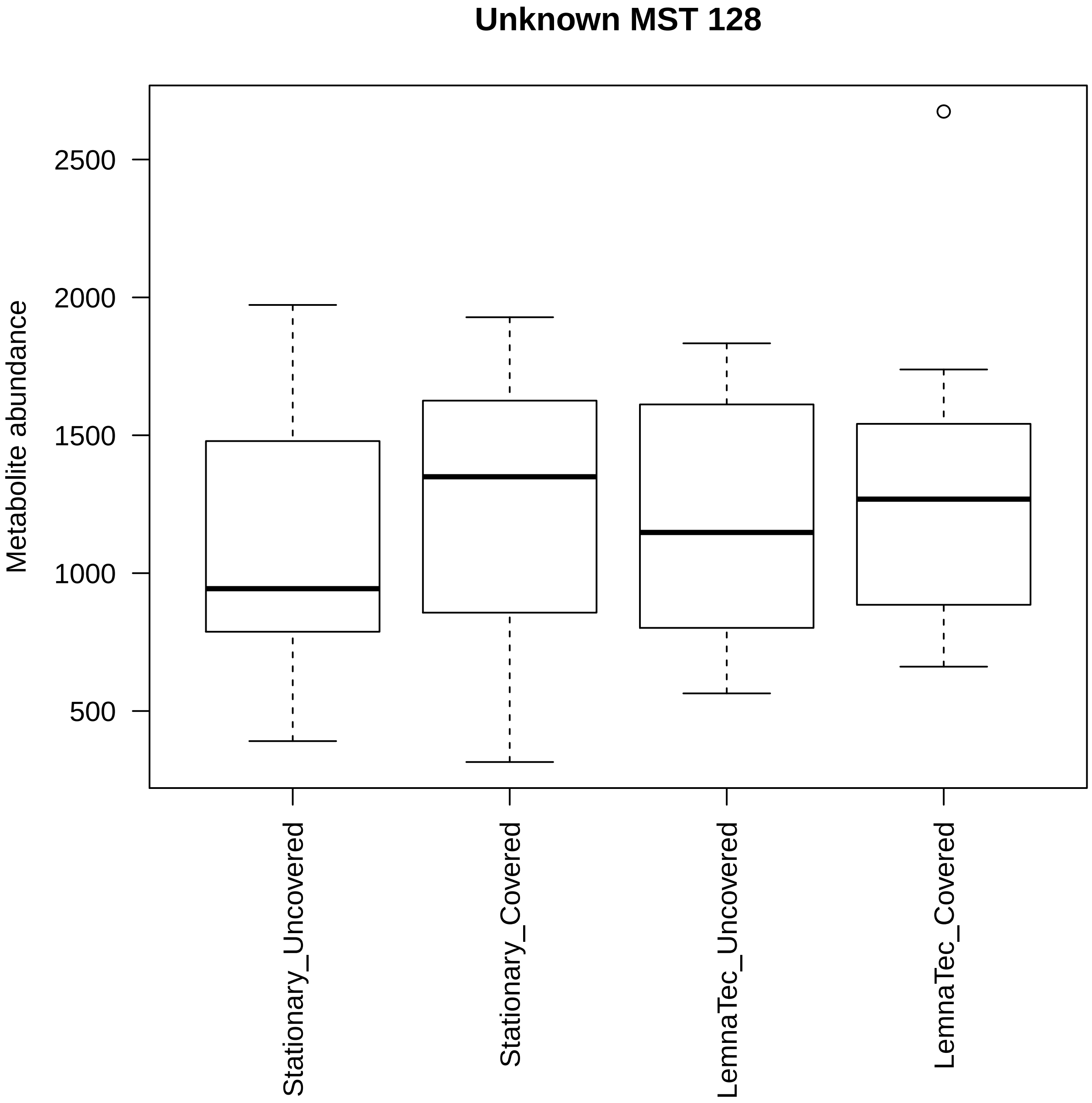

Unknown MST 128
Metabolite abundance
500
1000
1500
2000
2500
Stationary_Uncovered
Stationary_Covered
LemnaTec_Uncovered
LemnaTec_Covered

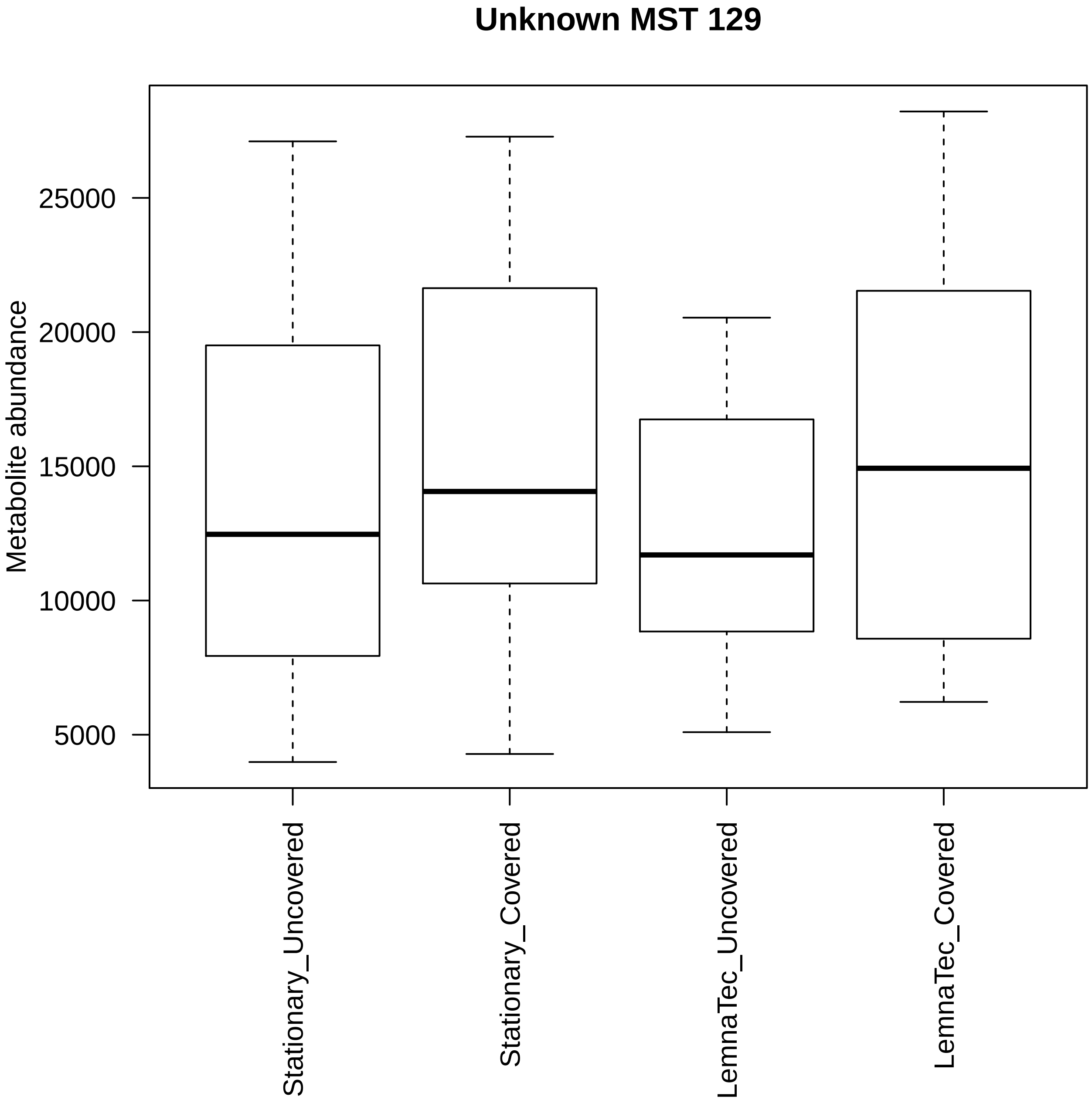
Unknown MST 129
Metabolite abundance
25000
20000
15000
10000
5000
Stationary_Uncovered
Stationary_Covered
LemnaTec_Uncovered
LemnaTec_Covered

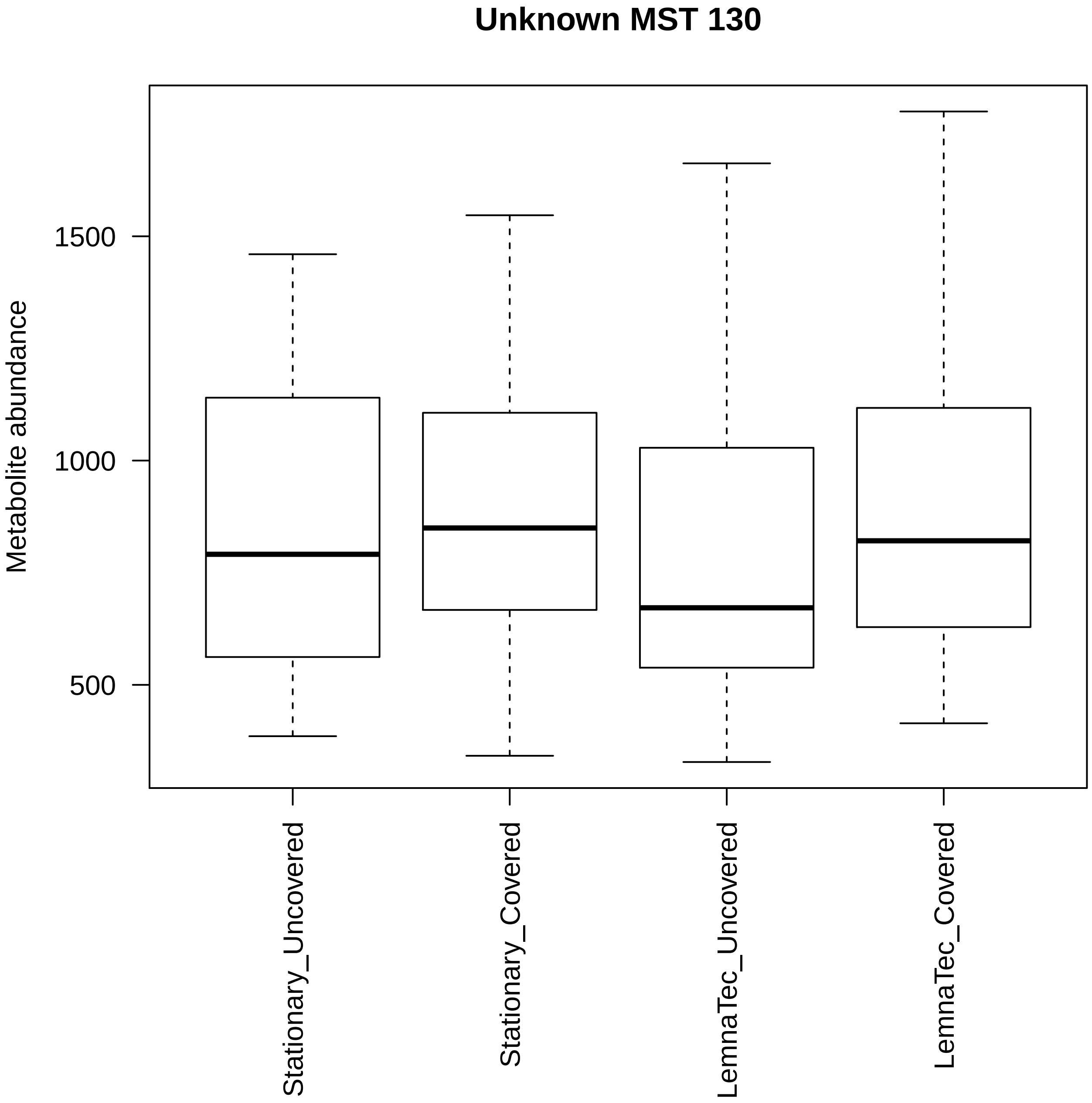

Unknown MST 130
Metabolite abundance
1500
1000
500
Stationary_Uncovered
Stationary_Covered
LemnaTec_Uncovered
LemnaTec_Covered

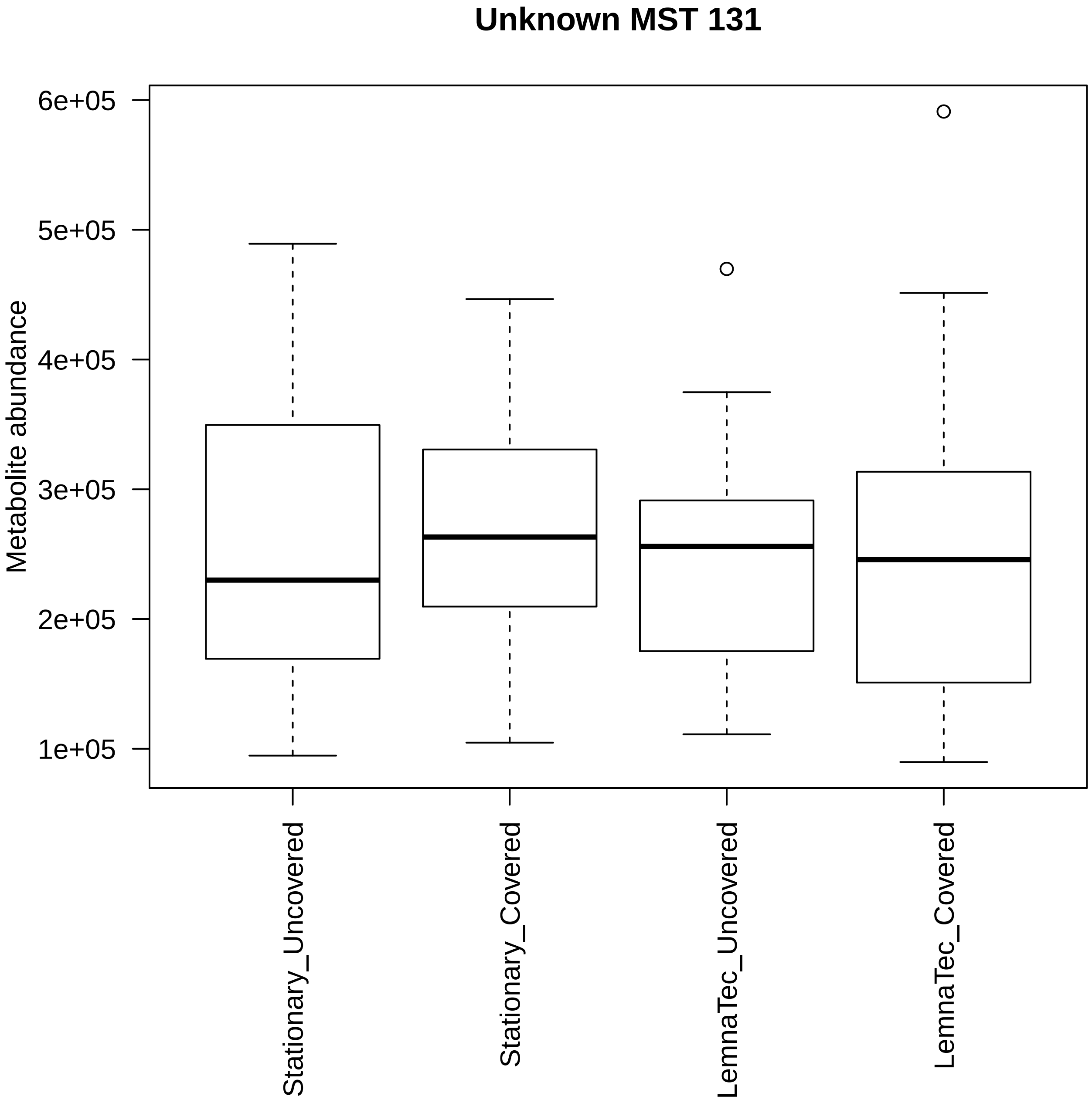

Unknown MST 131
Metabolite abundance
6e+05
5e+05
4e+05
3e+05
2e+05
1e+05
Stationary_Uncovered
Stationary_Covered
LemnaTec_Uncovered
LemnaTec_Covered

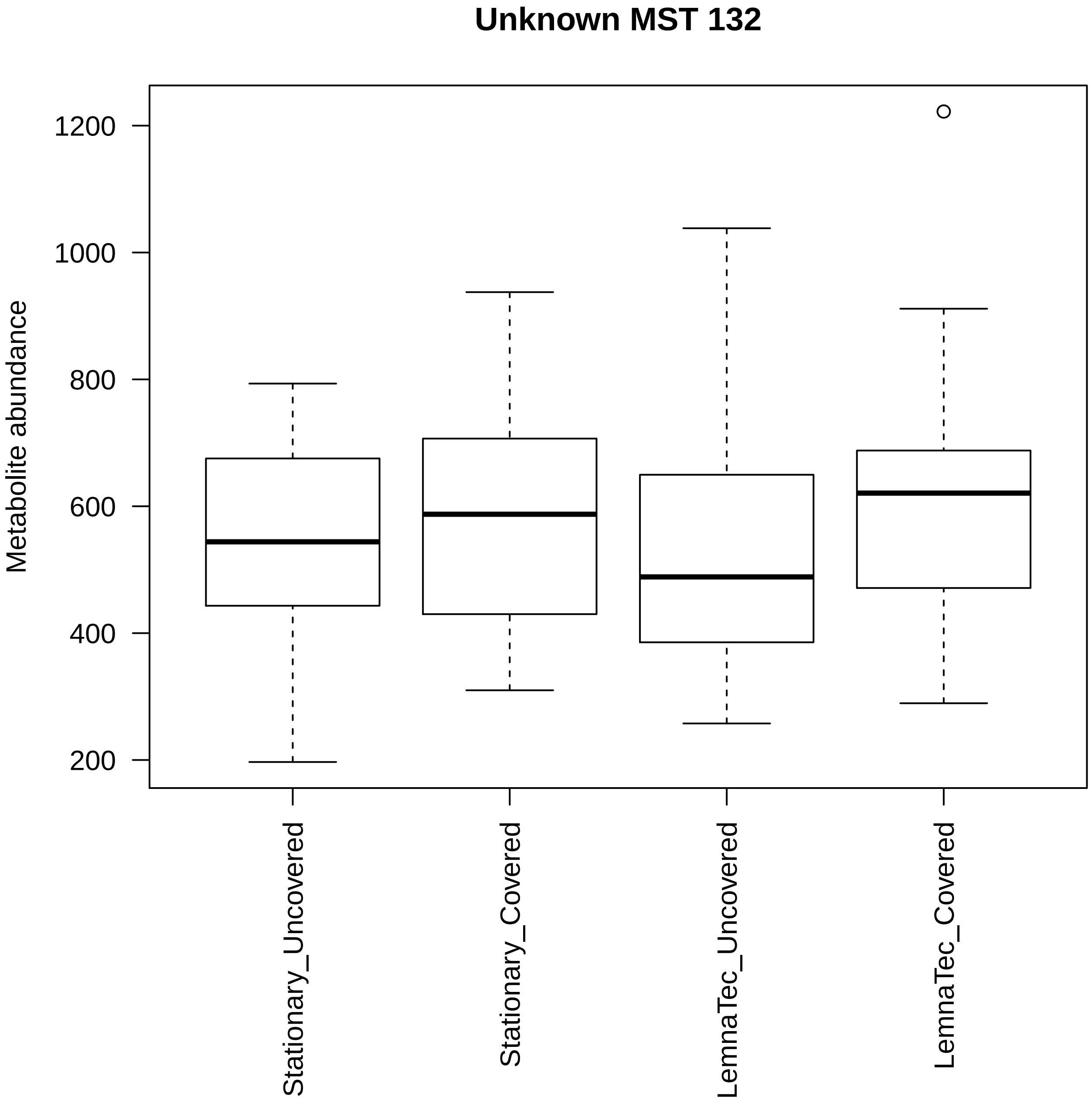

Unknown MST 132
Metabolite abundance
1200
1000
800
600
400
200
Stationary_Uncovered
Stationary_Covered
LemnaTec_Uncovered
LemnaTec_Covered

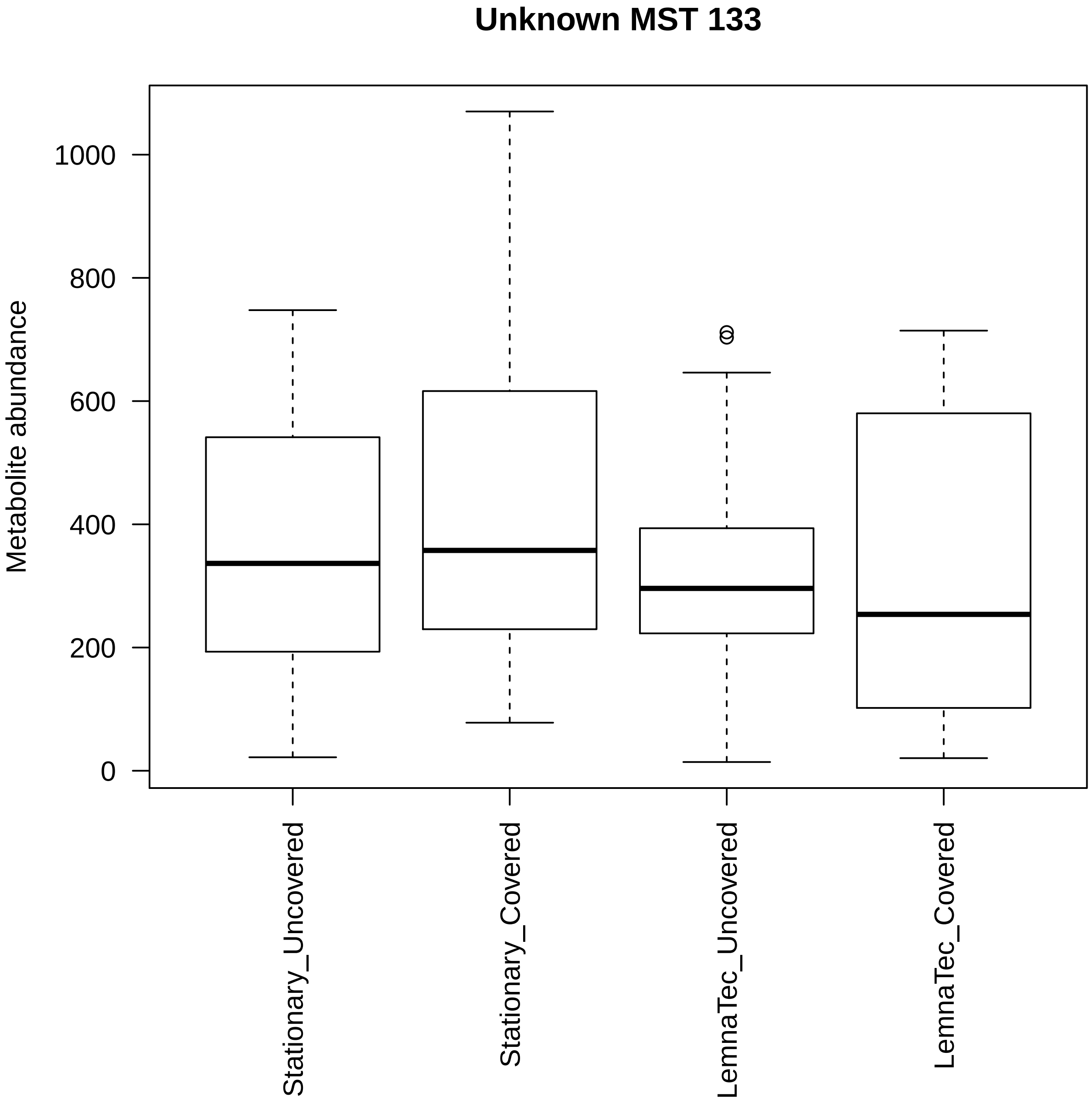

Unknown MST 133
Metabolite abundance
0
200
400
600
800
1000
Stationary_Uncovered
Stationary_Covered
LemnaTec_Uncovered
LemnaTec_Covered

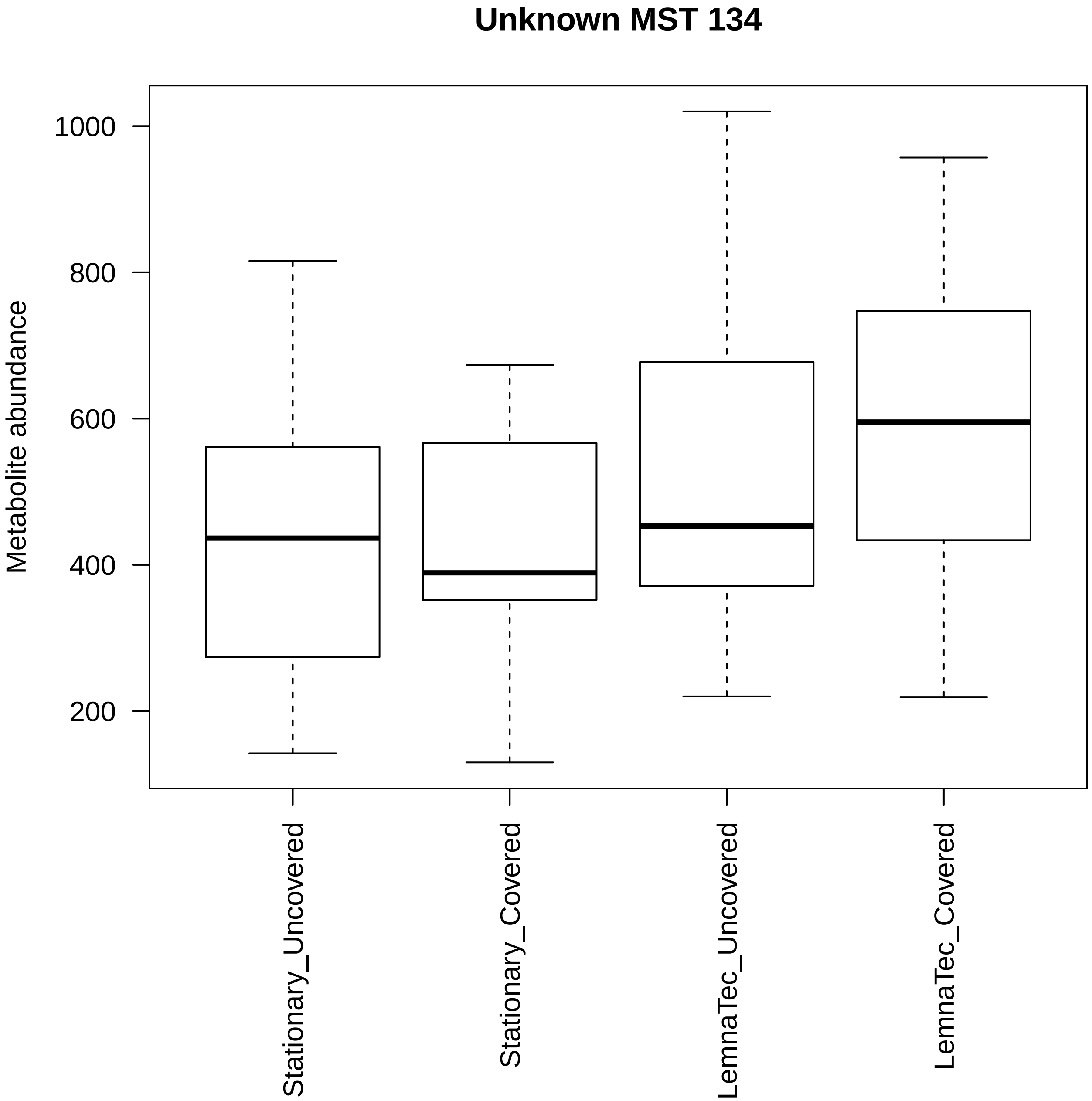

Unknown MST 134
Metabolite abundance
1000
800
600
400
200
Stationary_Uncovered
Stationary_Covered
LemnaTec_Uncovered
LemnaTec_Covered

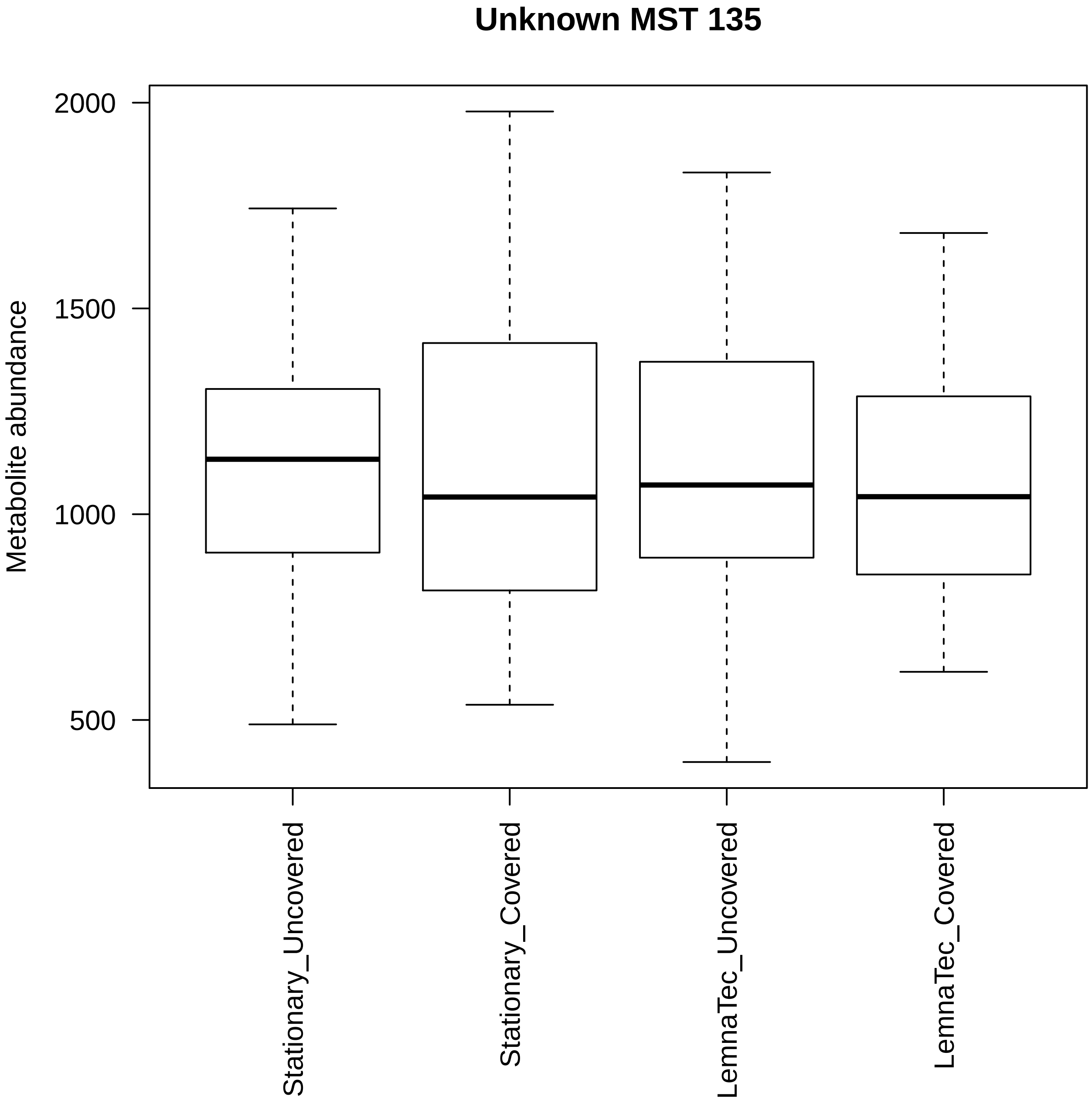

Unknown MST 135
Metabolite abundance
2000
1500
1000
500
Stationary_Uncovered
Stationary_Covered
LemnaTec_Uncovered
LemnaTec_Covered

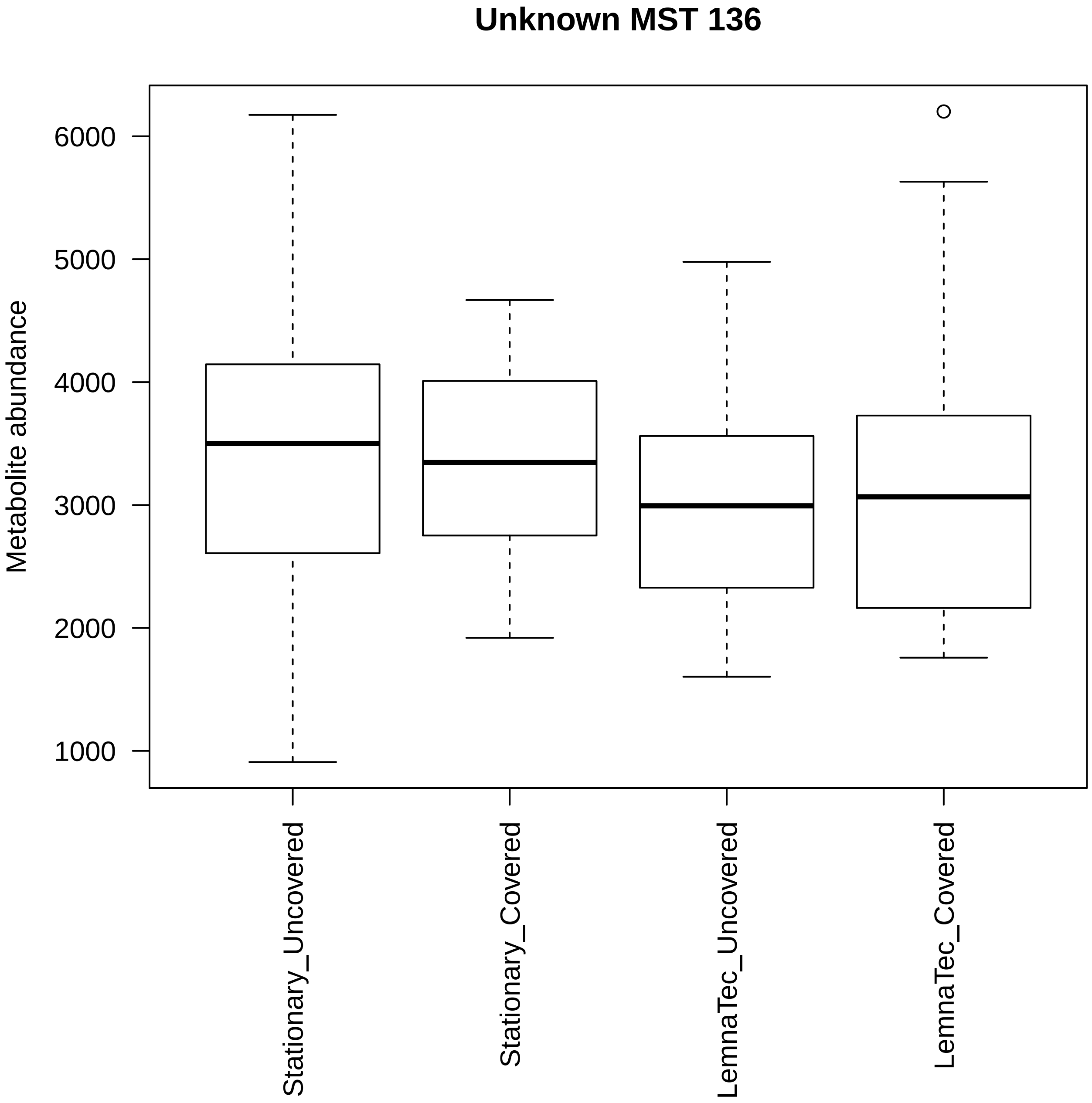
Unknown MST 136
Metabolite abundance
1000
2000
3000
4000
5000
6000
Stationary_Uncovered
Stationary_Covered
LemnaTec_Uncovered
LemnaTec_Covered

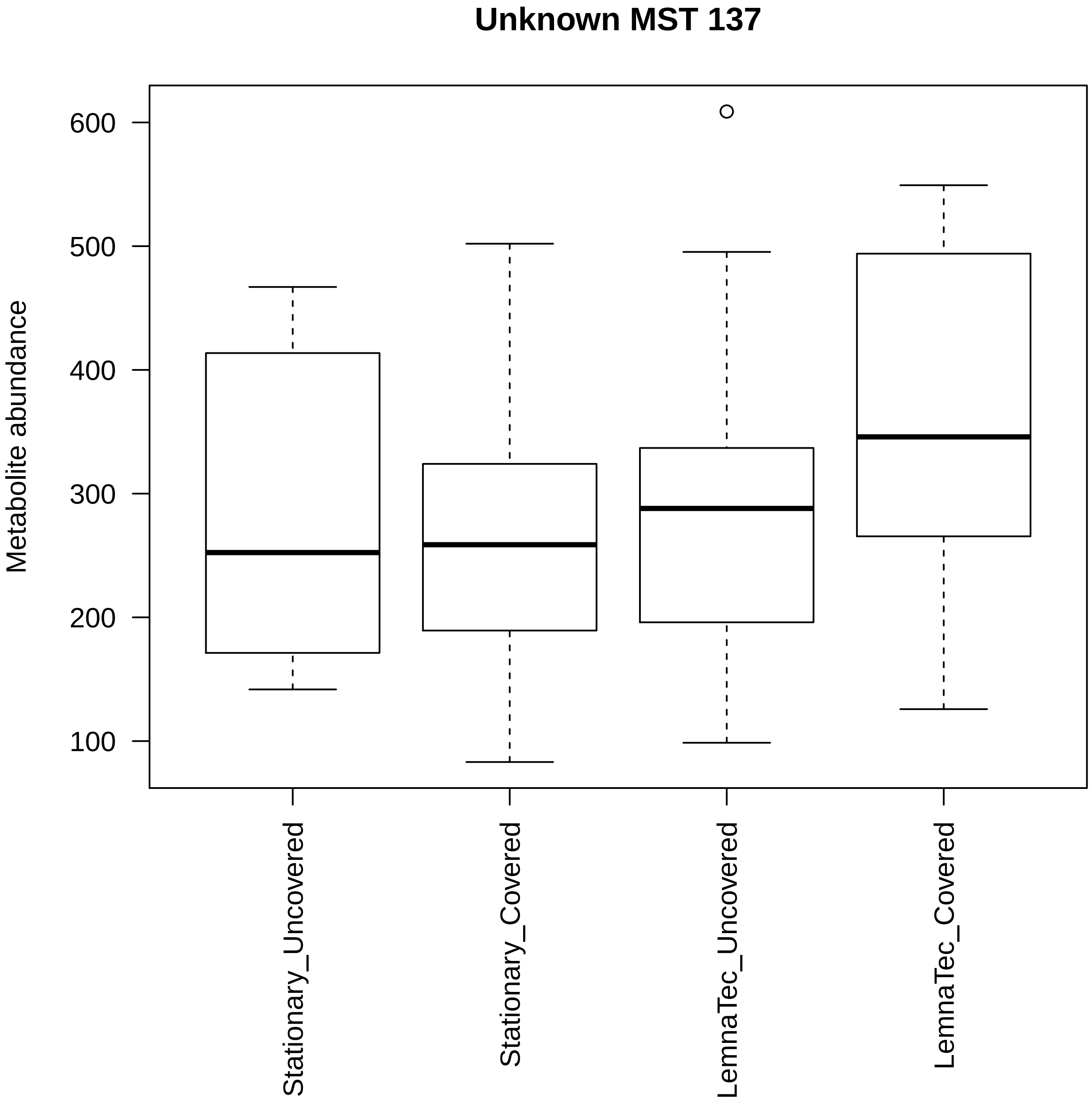

Unknown MST 137
Metabolite abundance
100
200
300
400
500
600
Stationary_Uncovered
Stationary_Covered
LemnaTec_Uncovered
LemnaTec_Covered

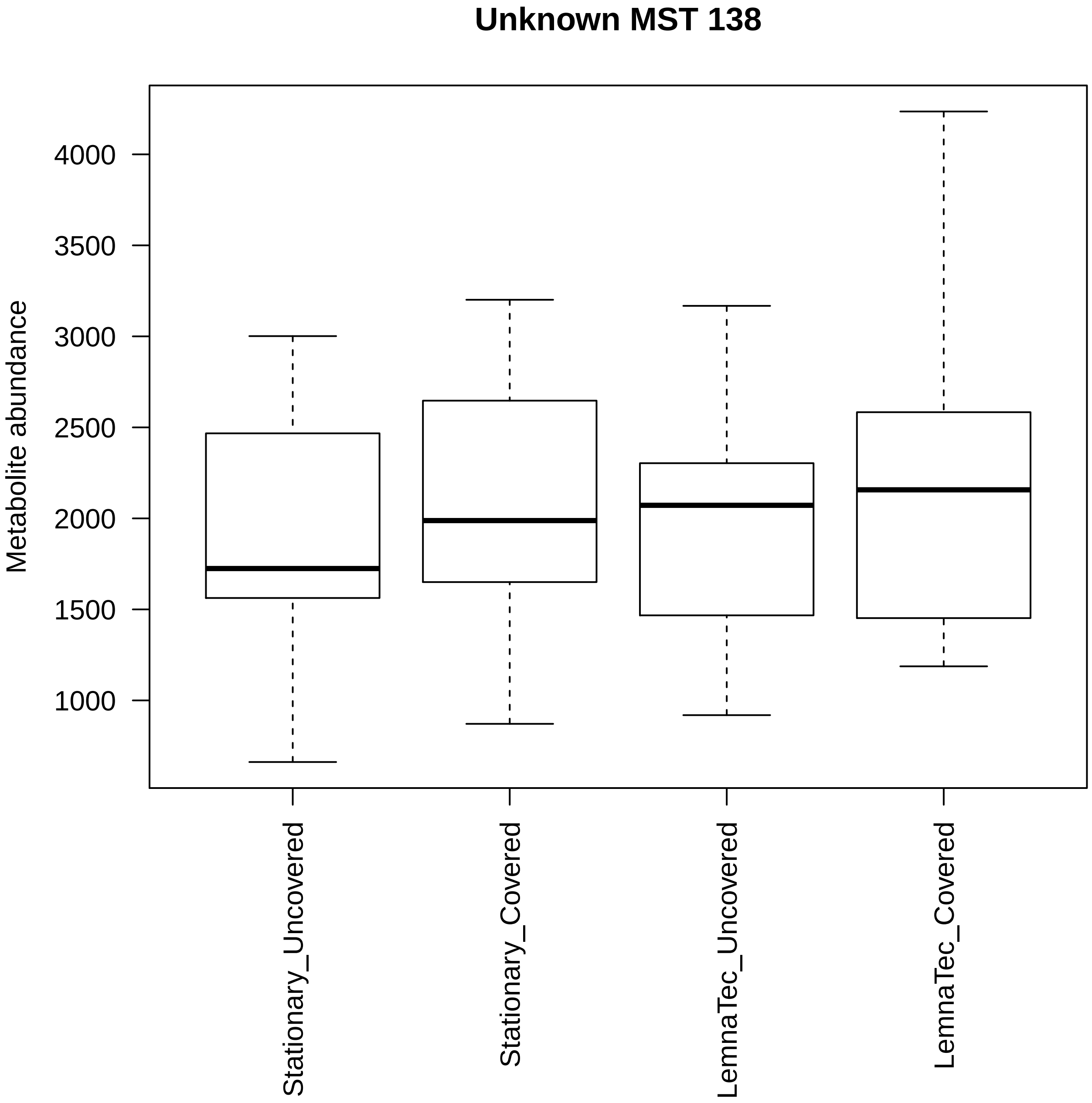
Unknown MST 138
Metabolite abundance
4000
3500
3000
2500
2000
1500
1000
Stationary_Uncovered
Stationary_Covered
LemnaTec_Uncovered
LemnaTec_Covered

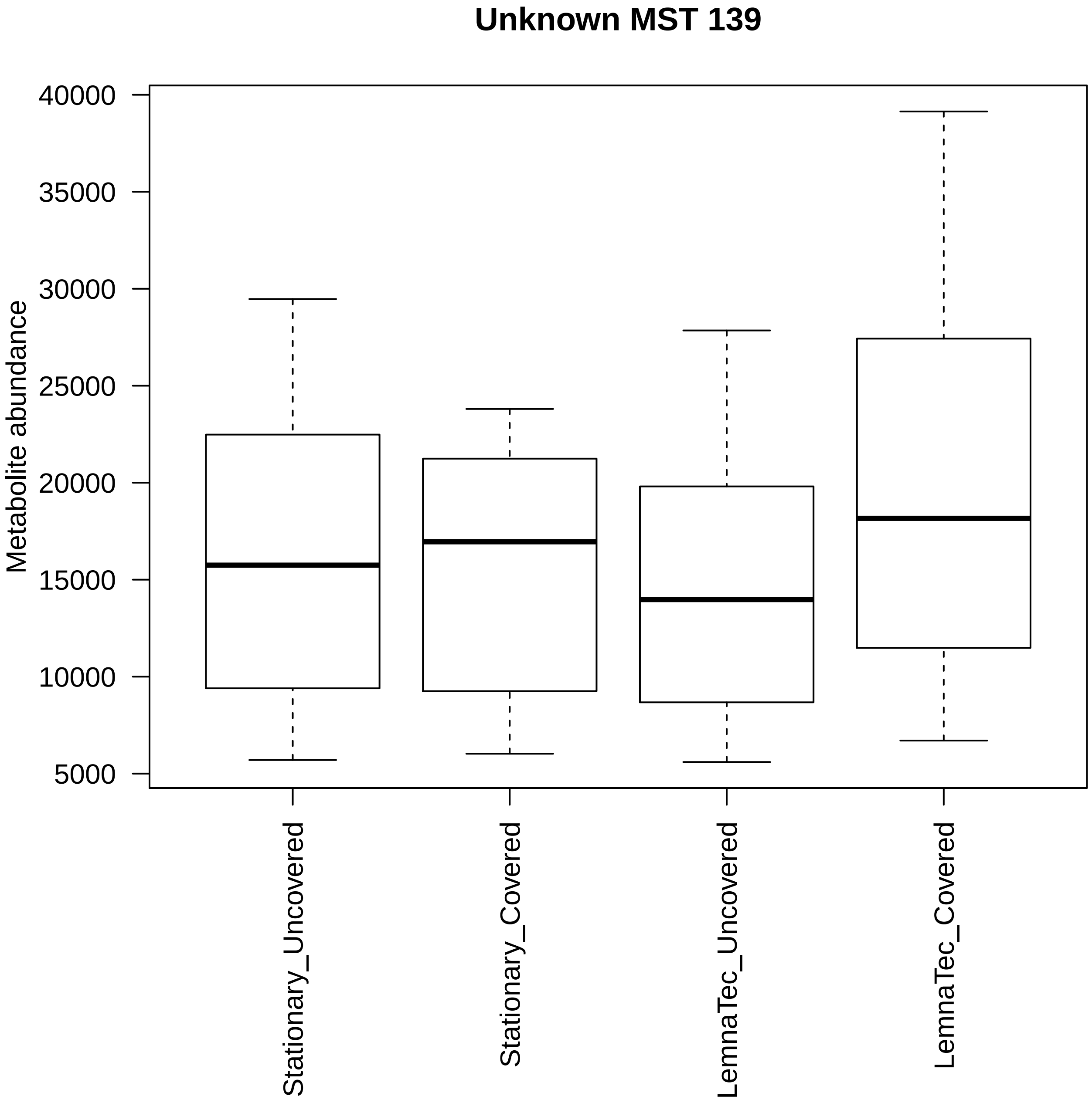
Unknown MST 139
Metabolite abundance
40000
35000
30000
25000
20000
15000
10000
5000
Stationary_Uncovered
Stationary_Covered
LemnaTec_Uncovered
LemnaTec_Covered

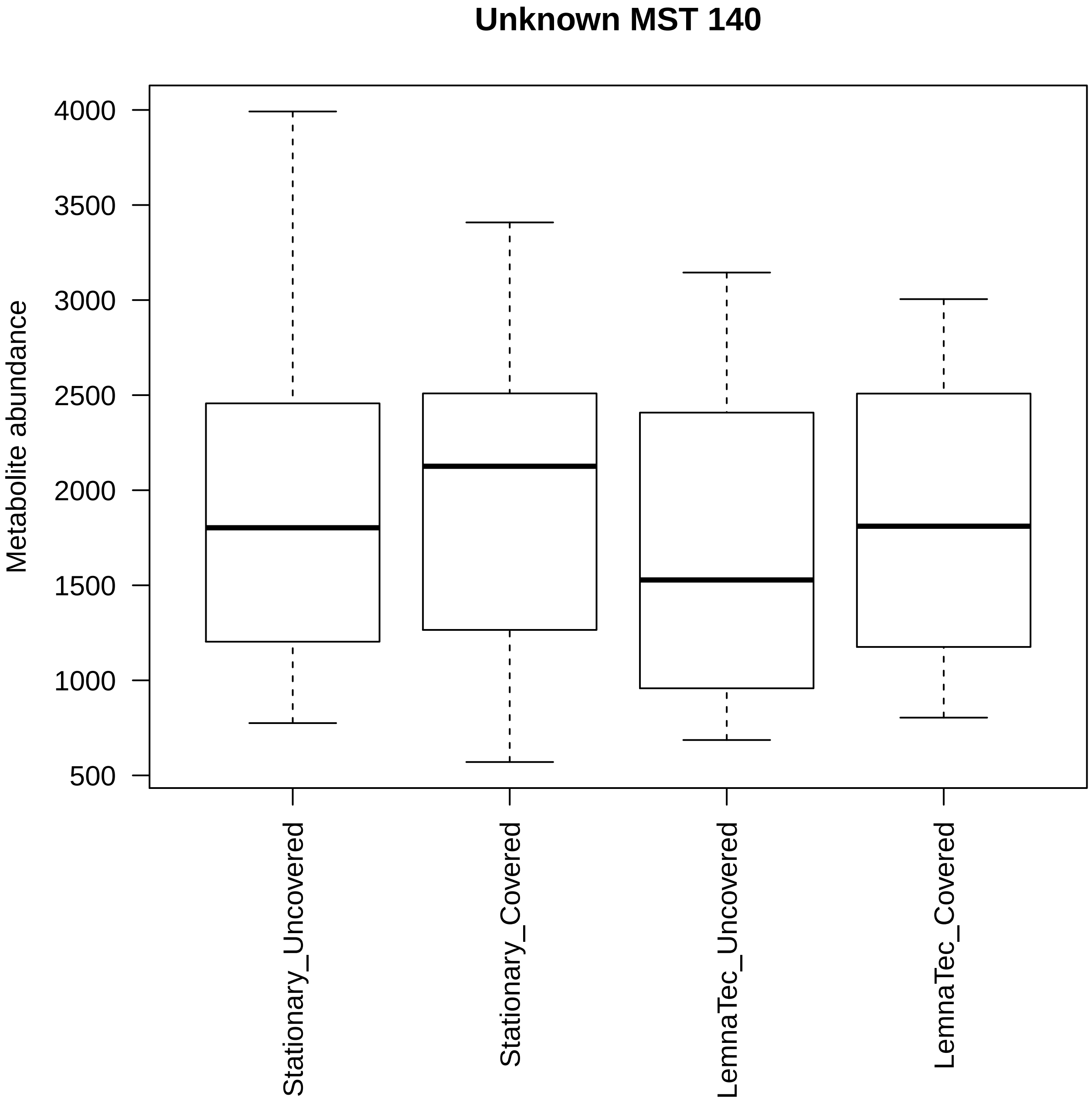

Unknown MST 140
Metabolite abundance
4000
3500
3000
2500
2000
1500
1000
500
Stationary_Uncovered
Stationary_Covered
LemnaTec_Uncovered
LemnaTec_Covered

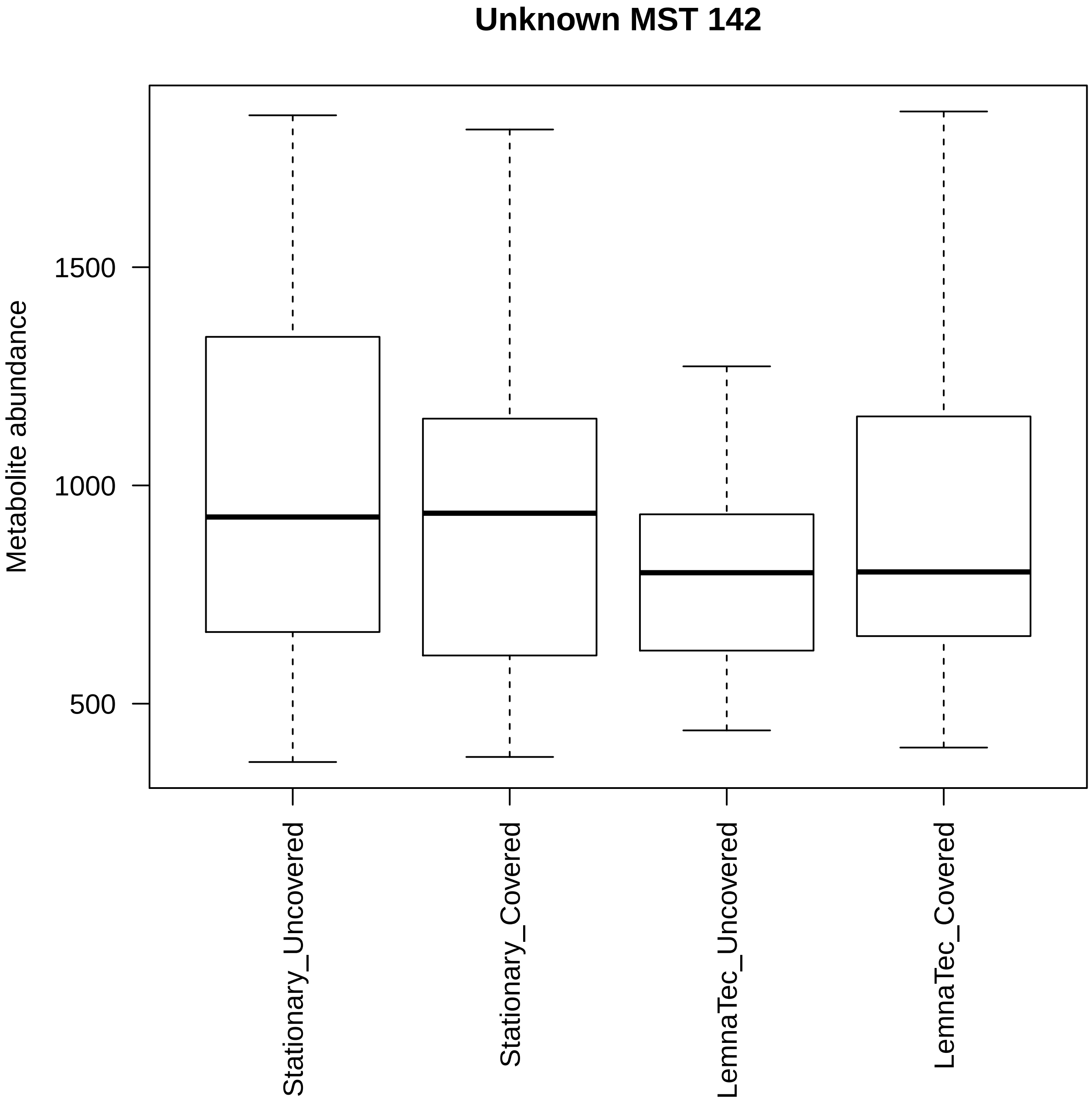

Unknown MST 142
Metabolite abundance
1500
1000
500
Stationary_Uncovered
Stationary_Covered
LemnaTec_Uncovered
LemnaTec_Covered

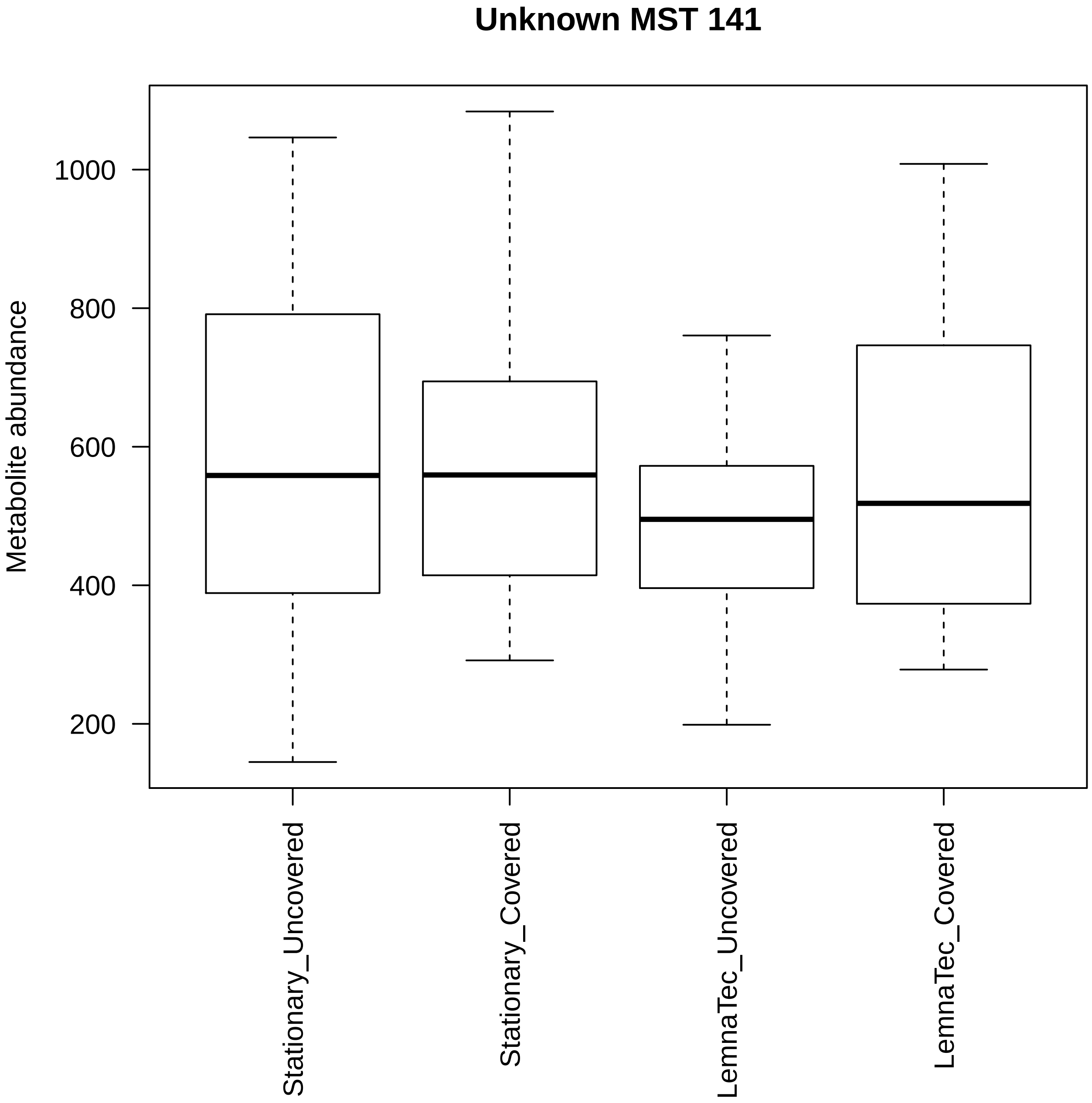
Unknown MST 141
Metabolite abundance
1000
800
600
400
200
Stationary_Uncovered
Stationary_Covered
LemnaTec_Uncovered
LemnaTec_Covered

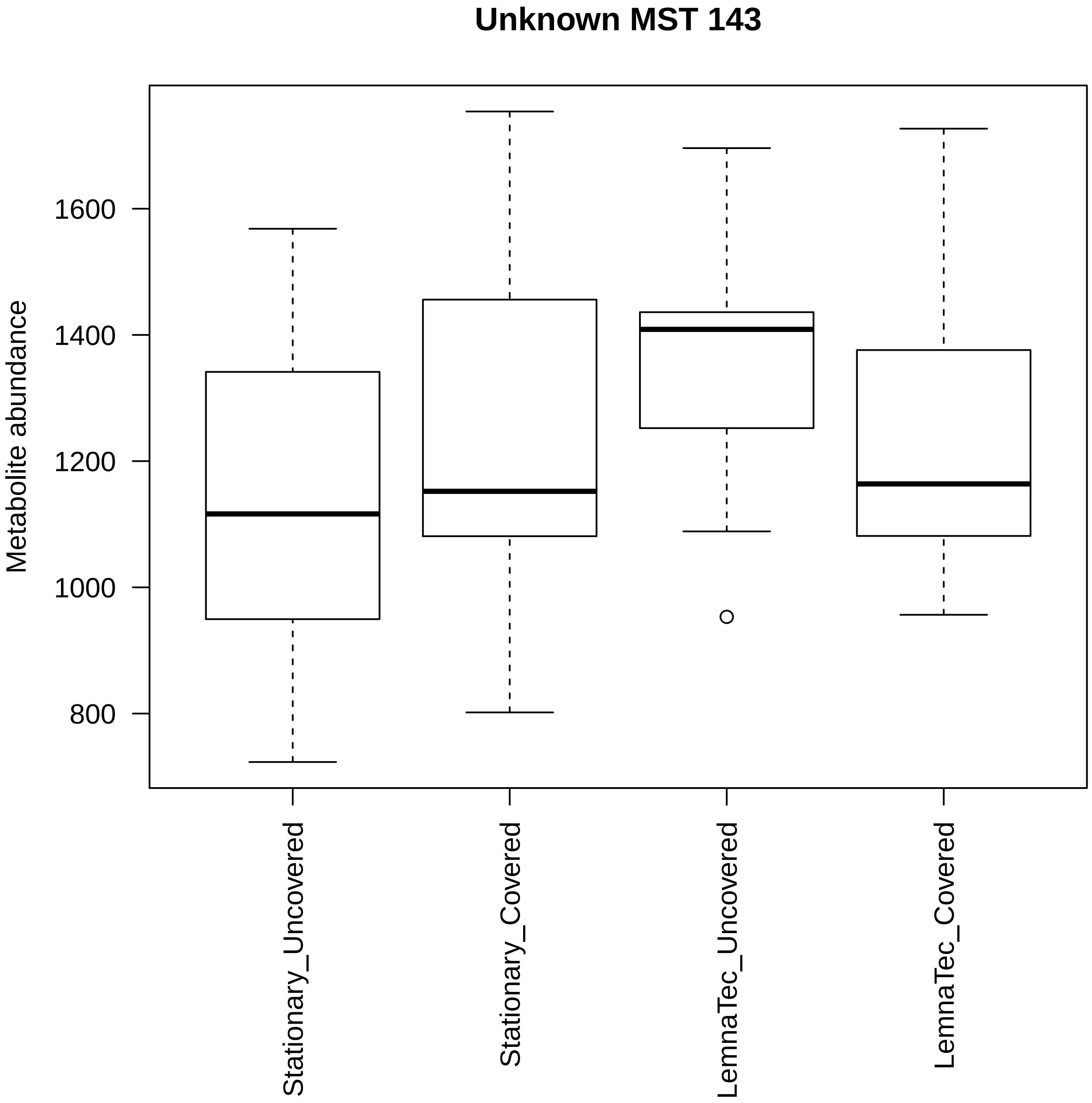
Unknown MST 143
Metabolite abundance
1600
1400
1200
1000
800
Stationary_Uncovered
Stationary_Covered
LemnaTec_Uncovered
LemnaTec_Covered

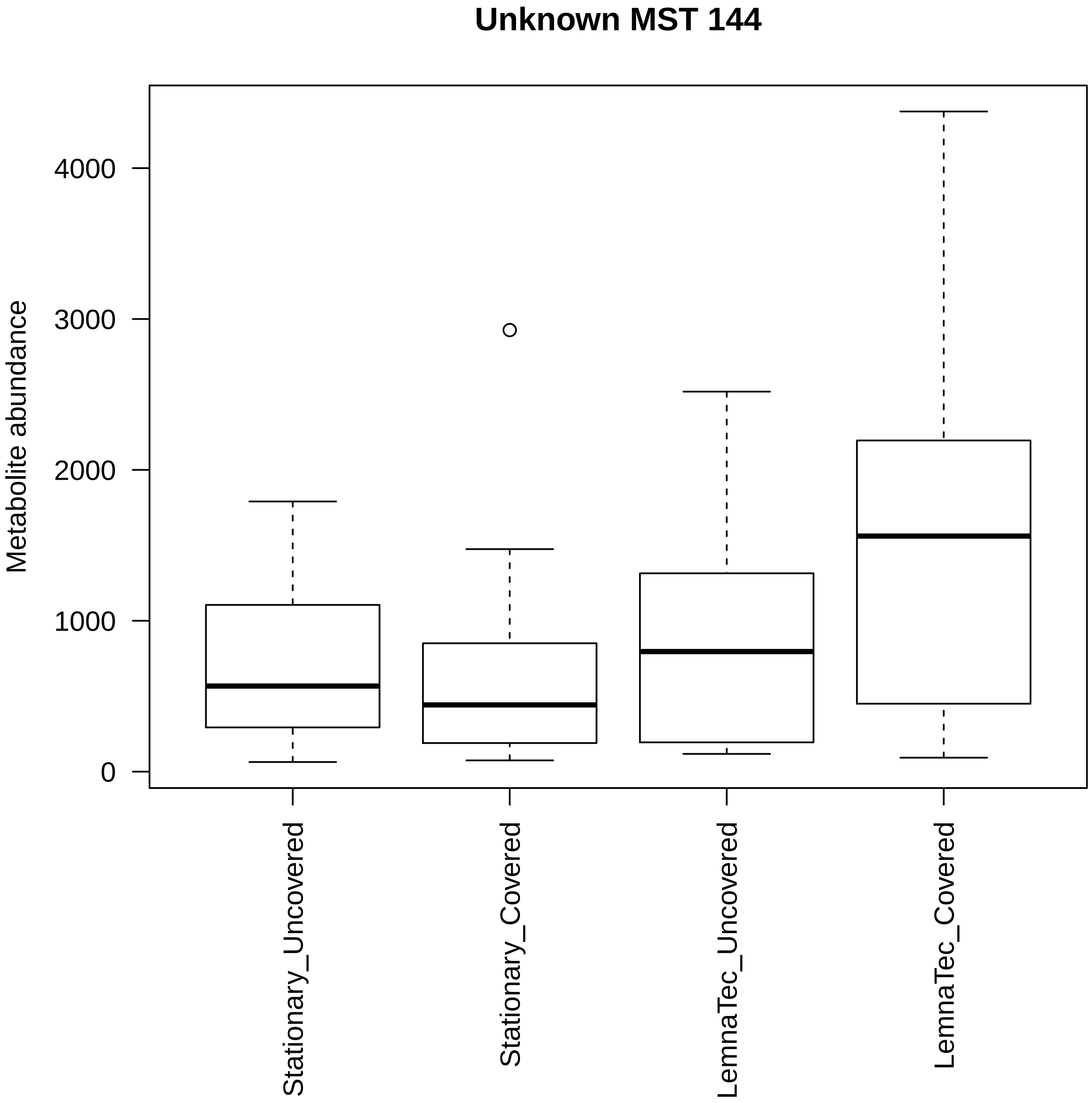

Unknown MST 144
Metabolite abundance
0
1000
2000
3000
4000
Stationary_Uncovered
Stationary_Covered
LemnaTec_Uncovered
LemnaTec_Covered

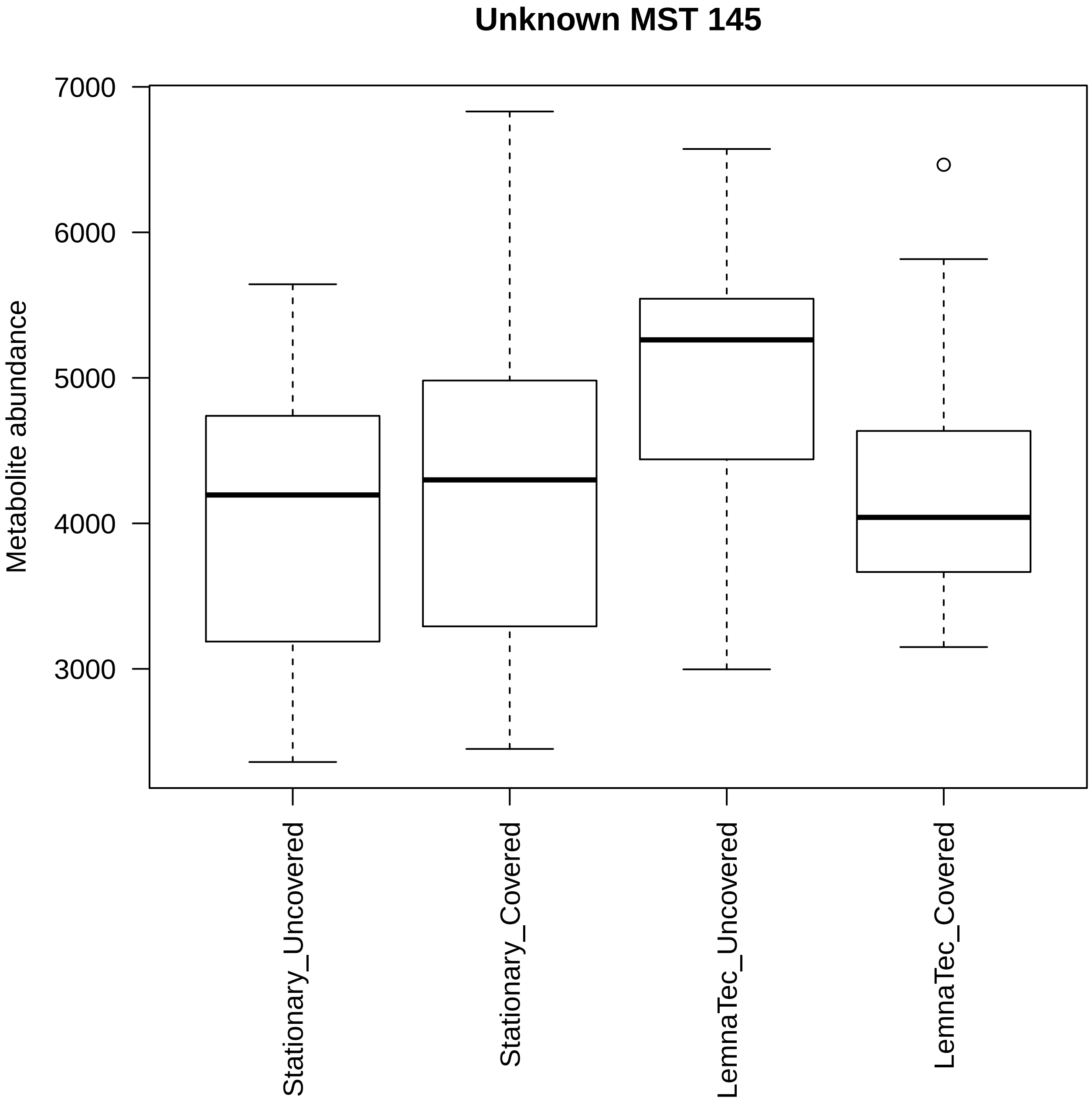

Unknown MST 145
Metabolite abundance
7000
6000
5000
4000
3000
Stationary_Uncovered
Stationary_Covered
LemnaTec_Uncovered
LemnaTec_Covered

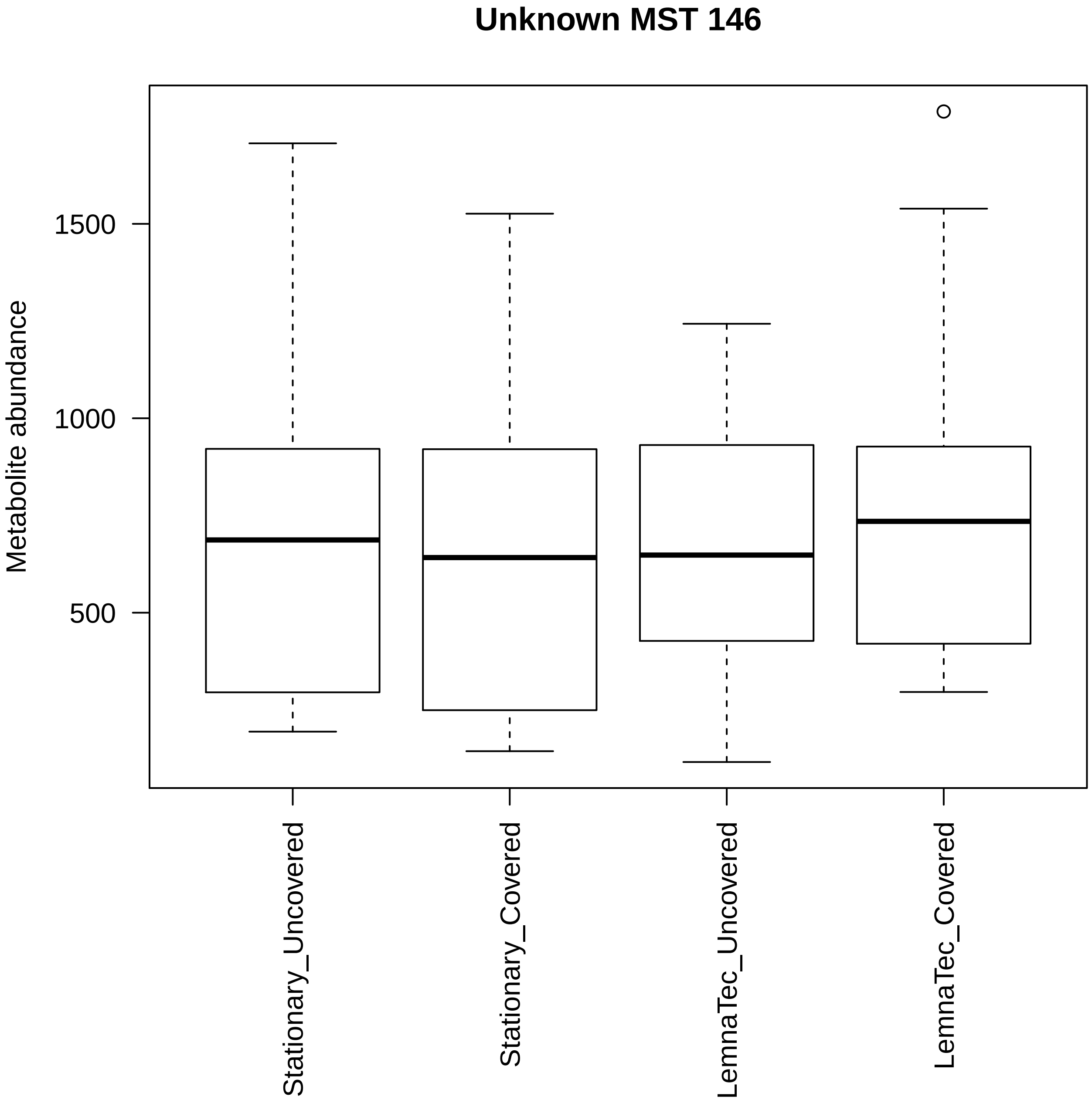
Unknown MST 146
Metabolite abundance
1500
1000
500
Stationary_Uncovered
Stationary_Covered
LemnaTec_Uncovered
LemnaTec_Covered

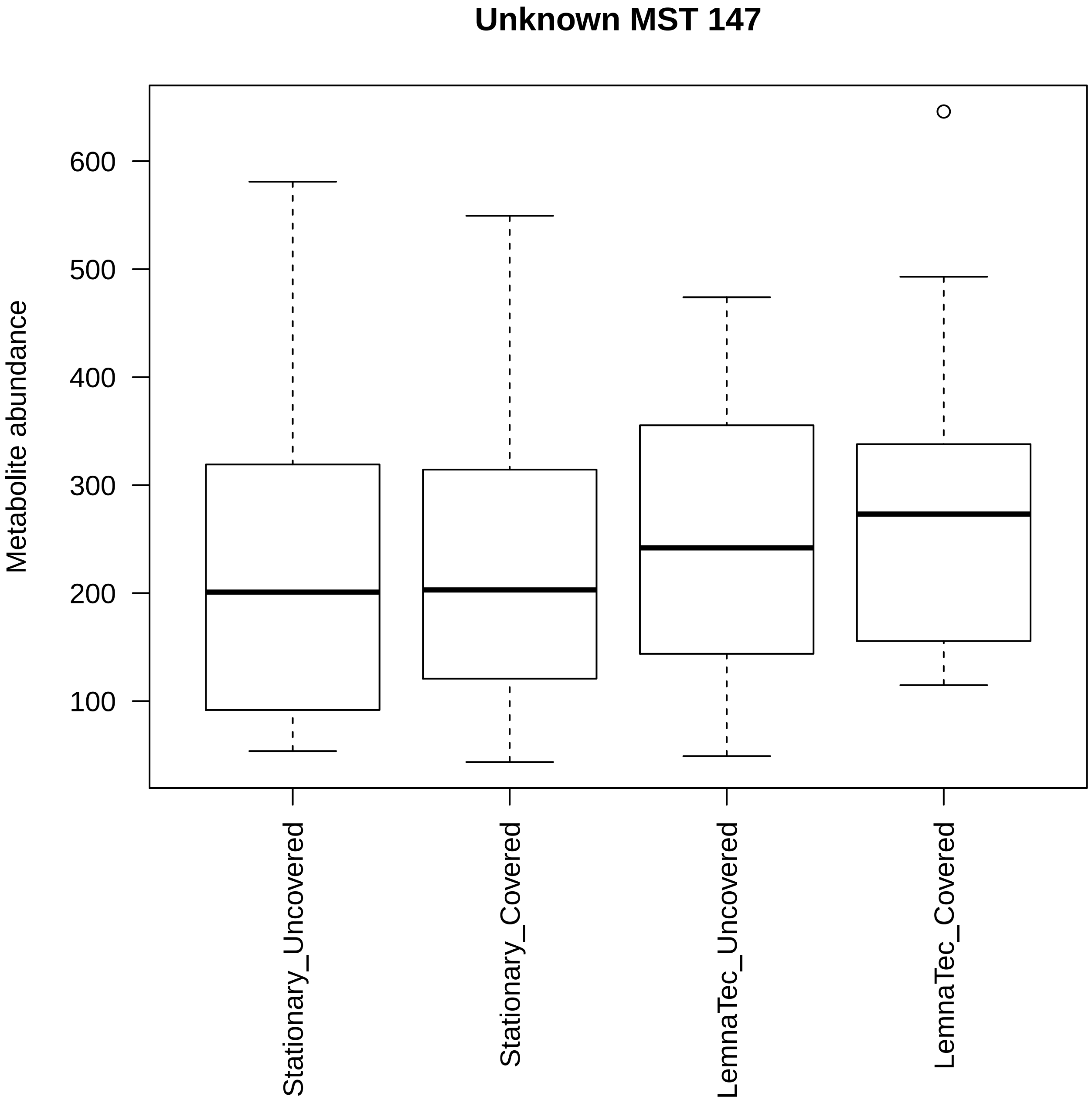
Unknown MST 147
Metabolite abundance
600
500
400
300
200
100
Stationary_Uncovered
Stationary_Covered
LemnaTec_Uncovered
LemnaTec_Covered

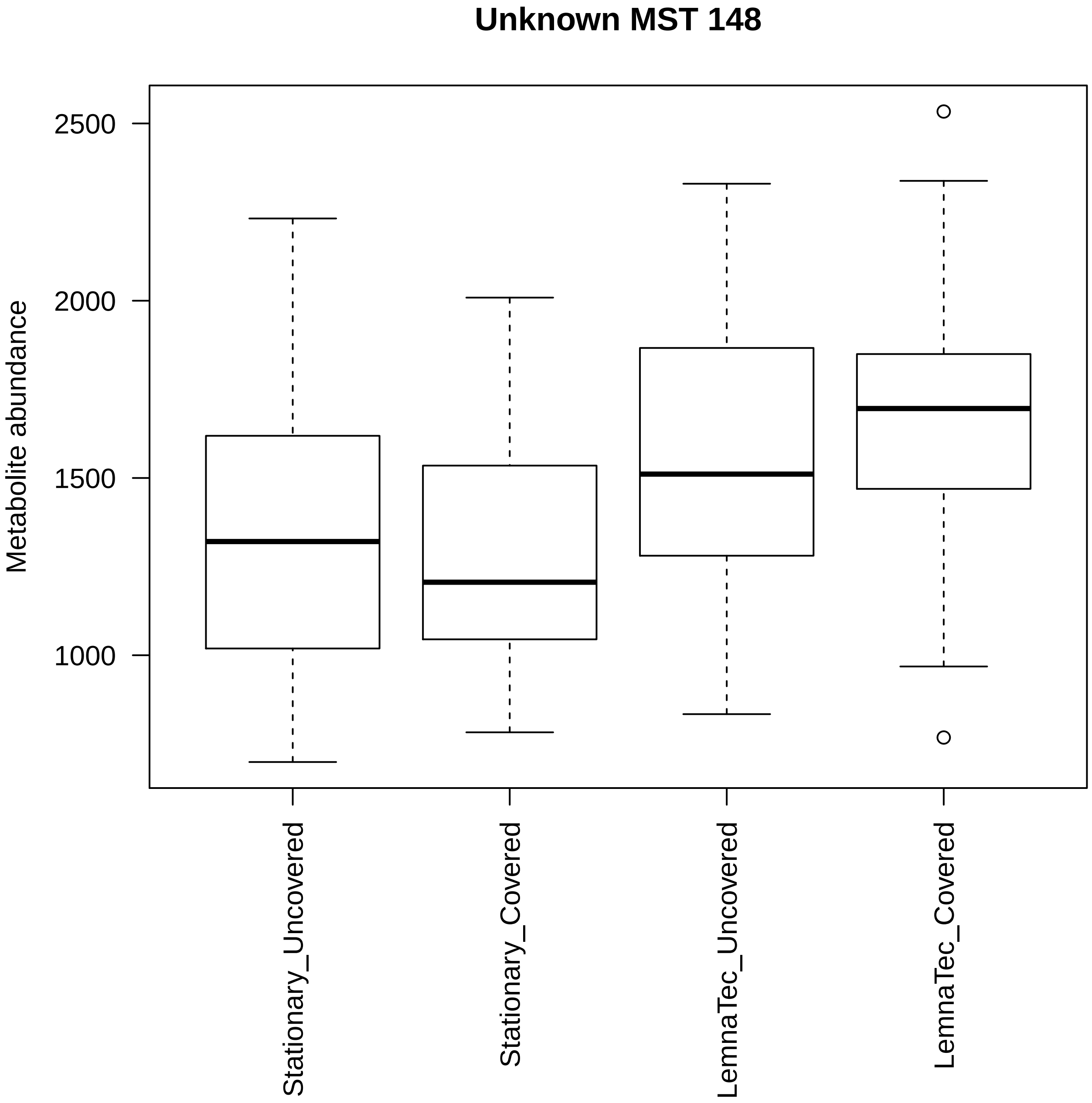

Unknown MST 148
Metabolite abundance
2500
2000
1500
1000
Stationary_Uncovered
Stationary_Covered
LemnaTec_Uncovered
LemnaTec_Covered

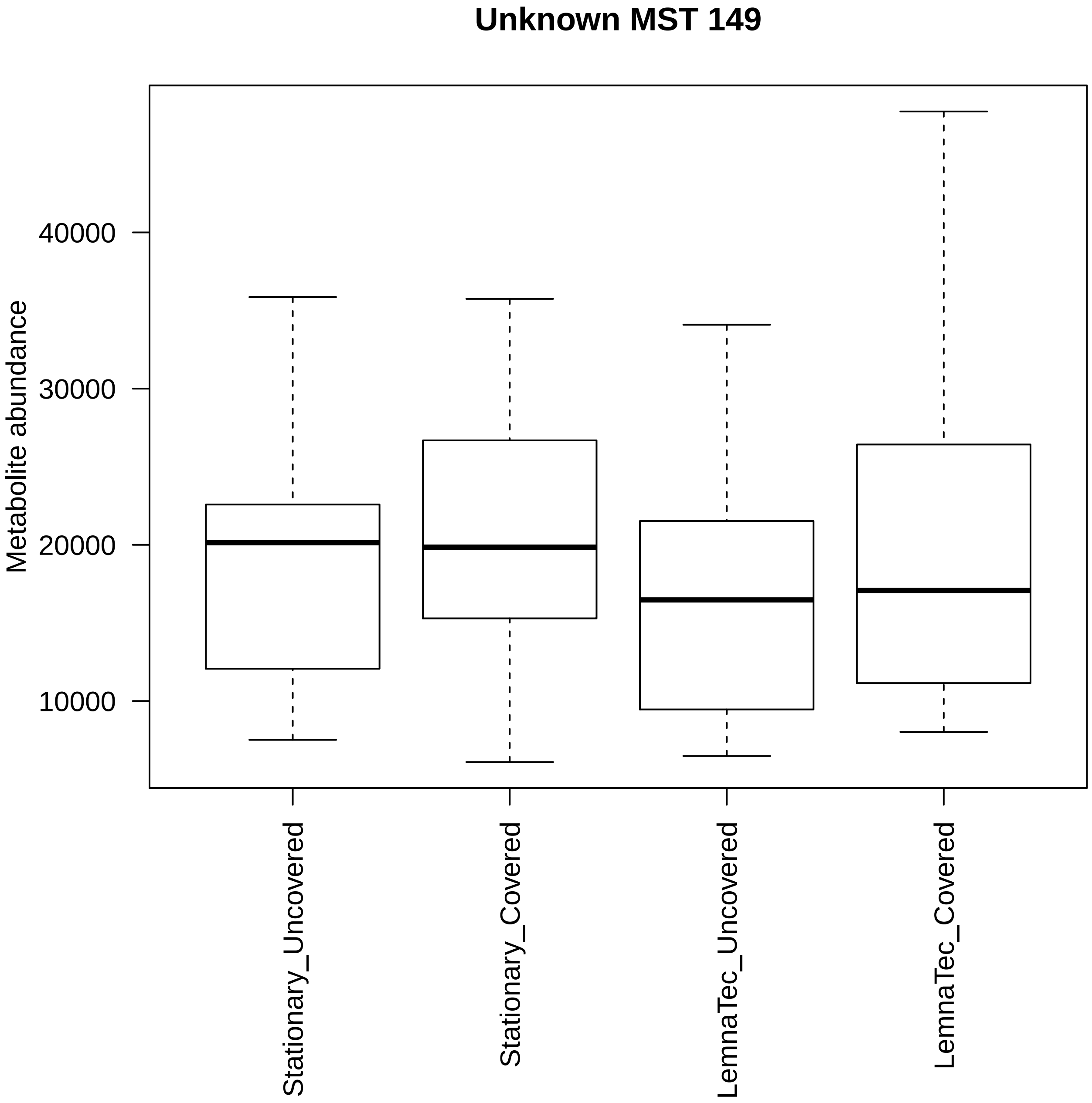

Unknown MST 149
Metabolite abundance
40000
30000
20000
10000
Stationary_Uncovered
Stationary_Covered
LemnaTec_Uncovered
LemnaTec_Covered

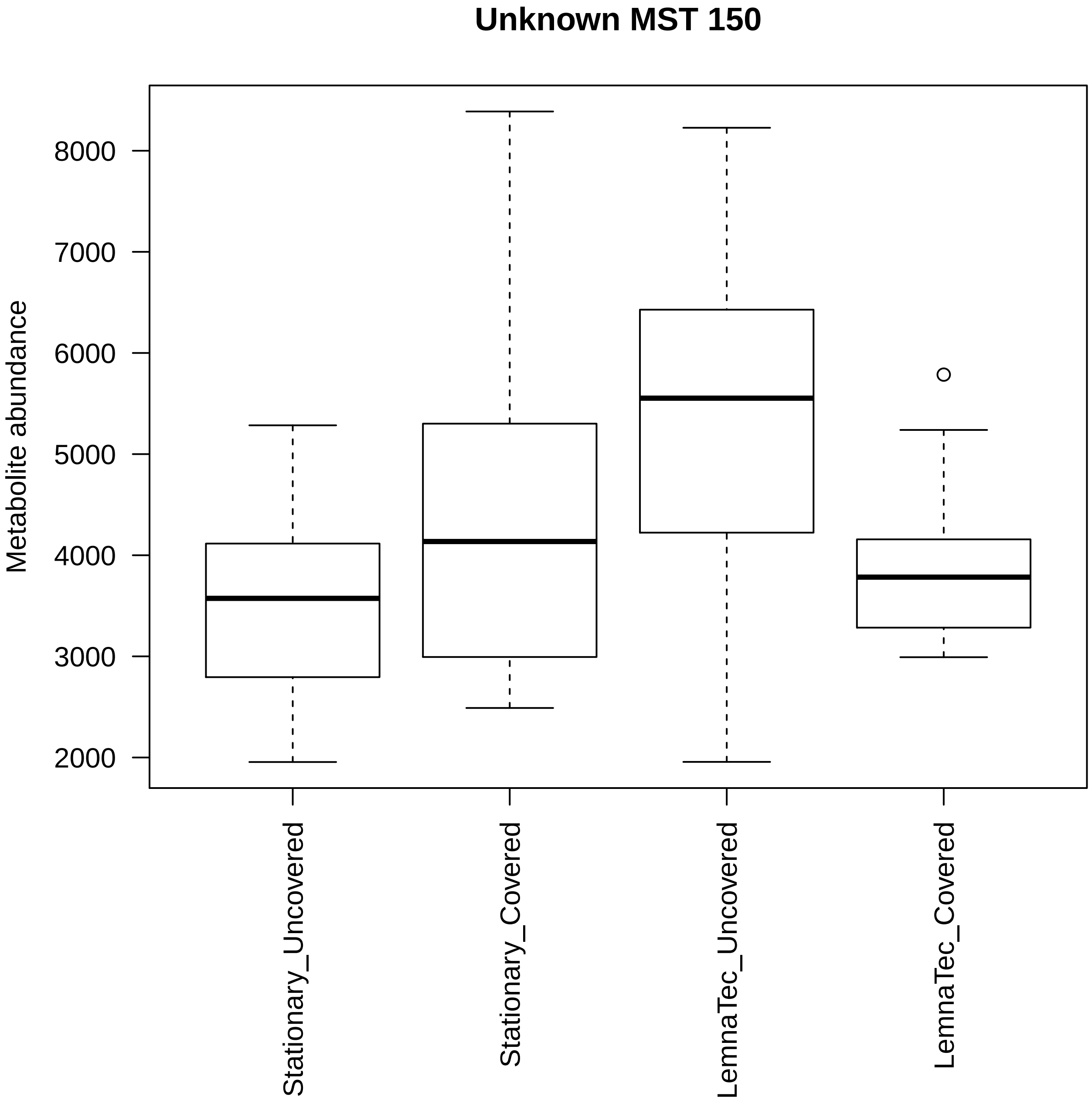

Unknown MST 150
Metabolite abundance
8000
7000
6000
5000
4000
3000
2000
Stationary_Uncovered
Stationary_Covered
LemnaTec_Uncovered
LemnaTec_Covered

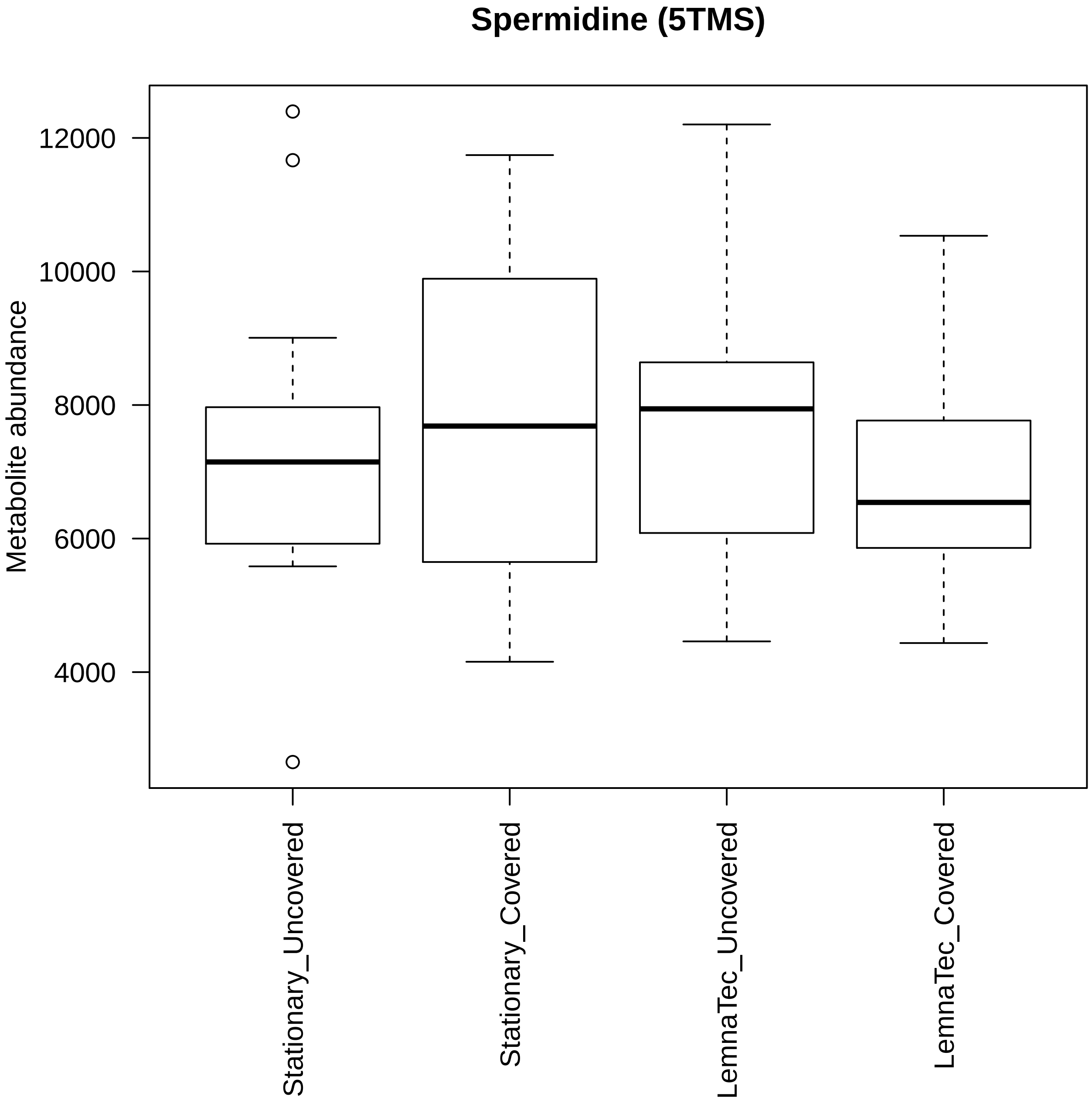

Spermidine (5TMS)
Metabolite abundance
4000
6000
8000
10000
12000
Stationary_Uncovered
Stationary_Covered
LemnaTec_Uncovered
LemnaTec_Covered

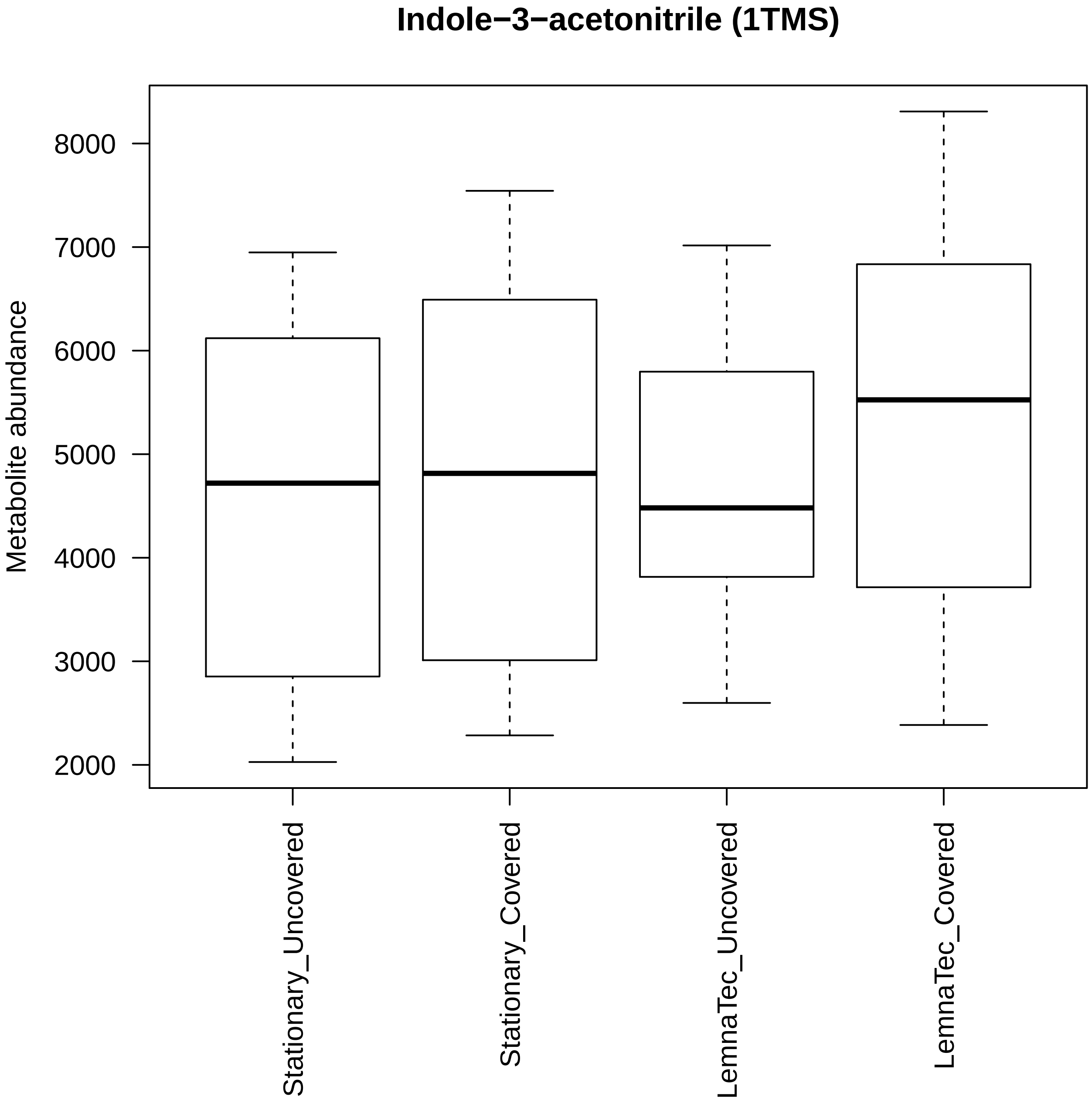
Indole−3−acetonitrile (1TMS)
Metabolite abundance
2000
3000
4000
5000
6000
7000
8000
Stationary_Uncovered
Stationary_Covered
LemnaTec_Uncovered
LemnaTec_Covered

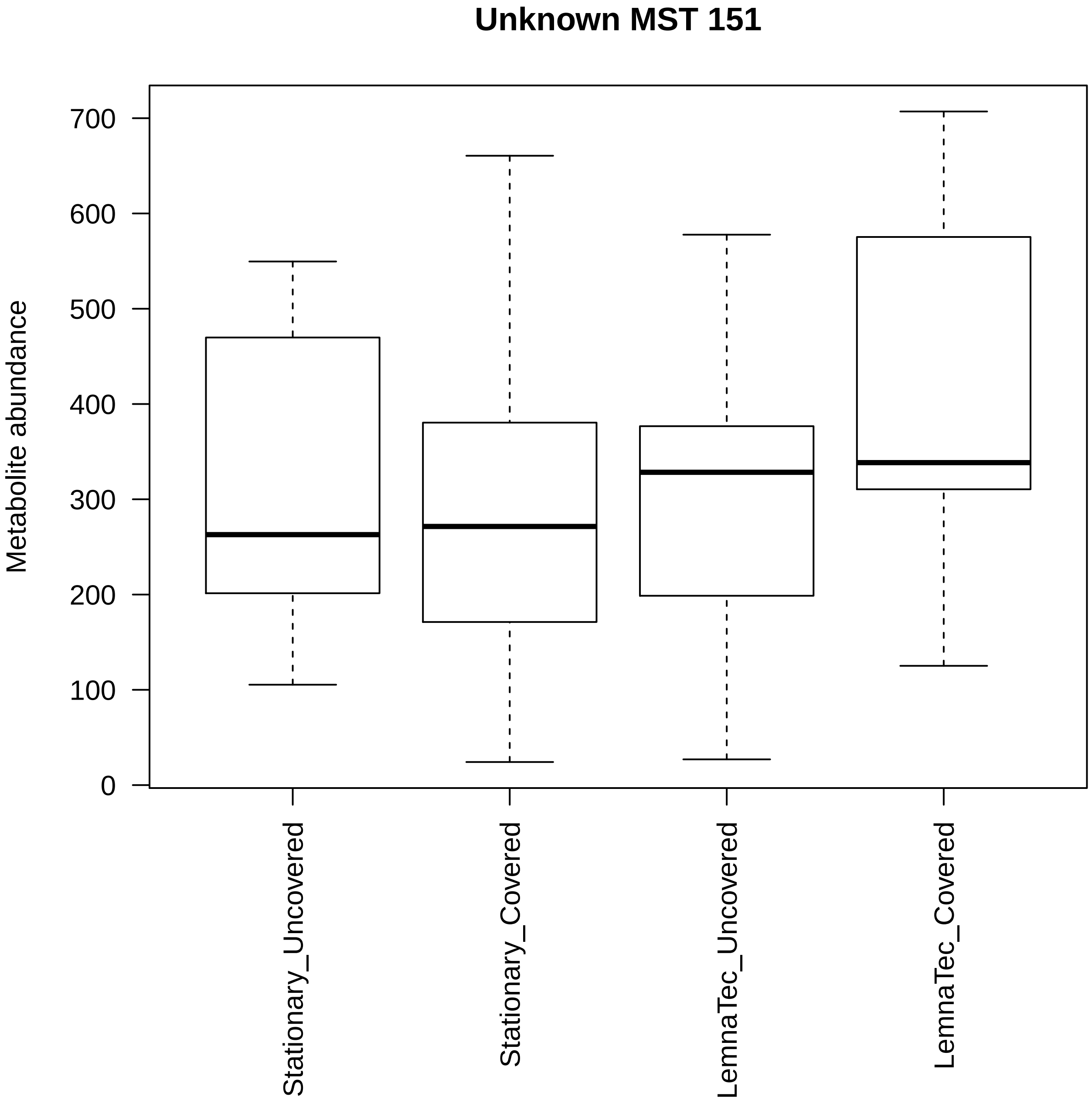

Unknown MST 151
Metabolite abundance
0
100
200
300
400
500
600
700
Stationary_Uncovered
Stationary_Covered
LemnaTec_Uncovered
LemnaTec_Covered

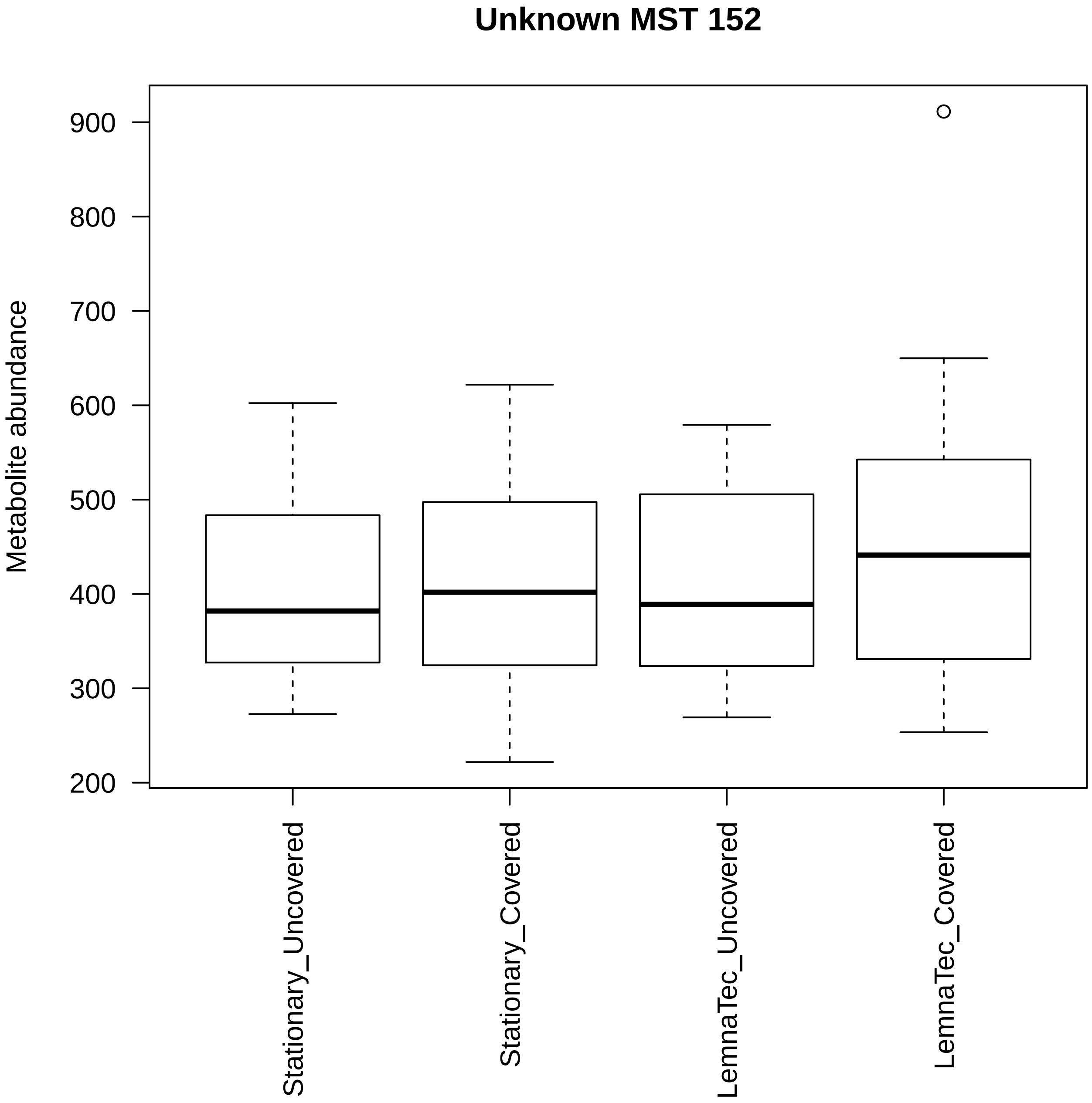

Unknown MST 152
Metabolite abundance
900
800
700
600
500
400
300
200
Stationary_Uncovered
Stationary_Covered
LemnaTec_Uncovered
LemnaTec_Covered

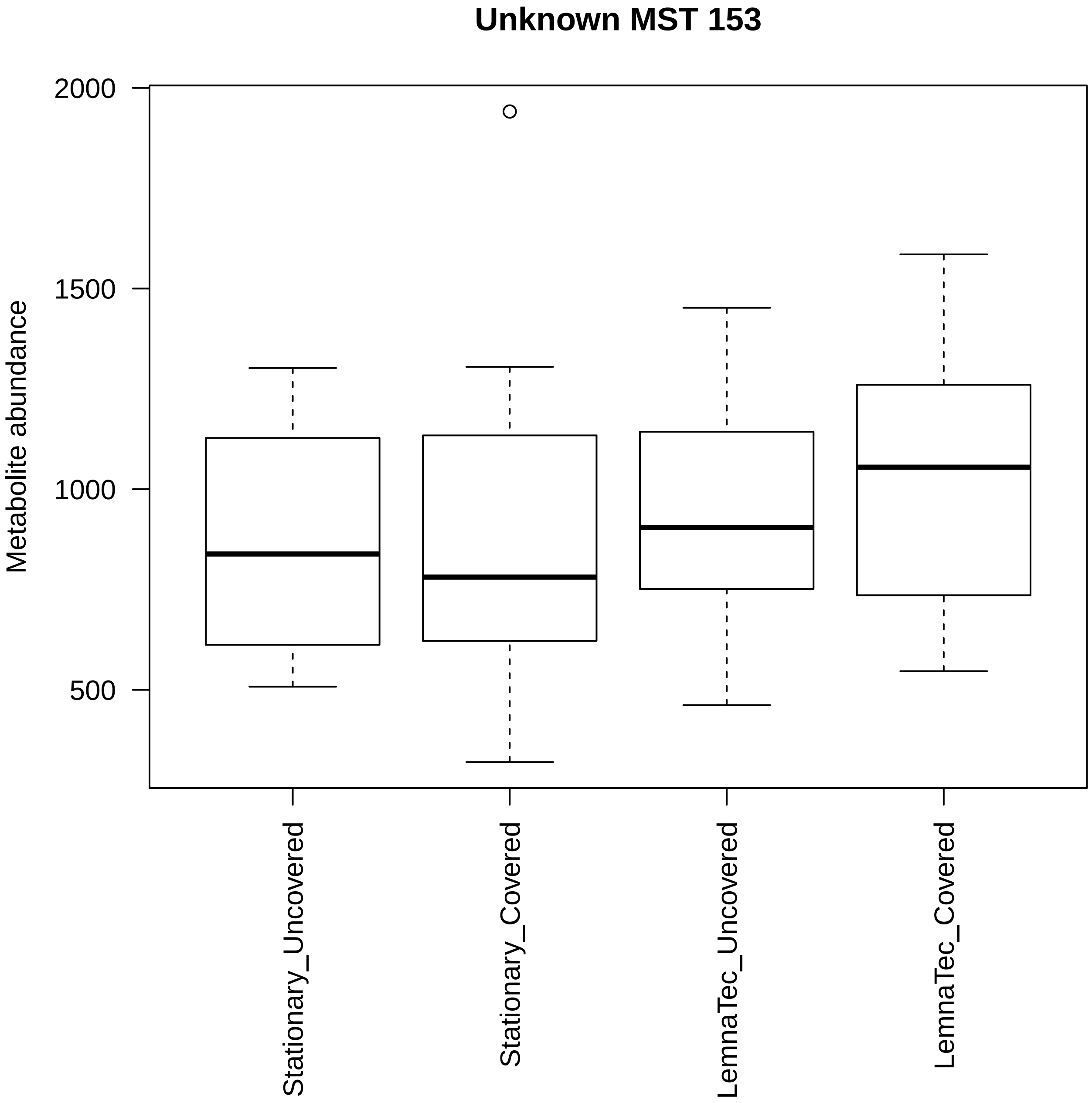
Unknown MST 153
Metabolite abundance
2000
1500
1000
500
Stationary_Uncovered
Stationary_Covered
LemnaTec_Uncovered
LemnaTec_Covered

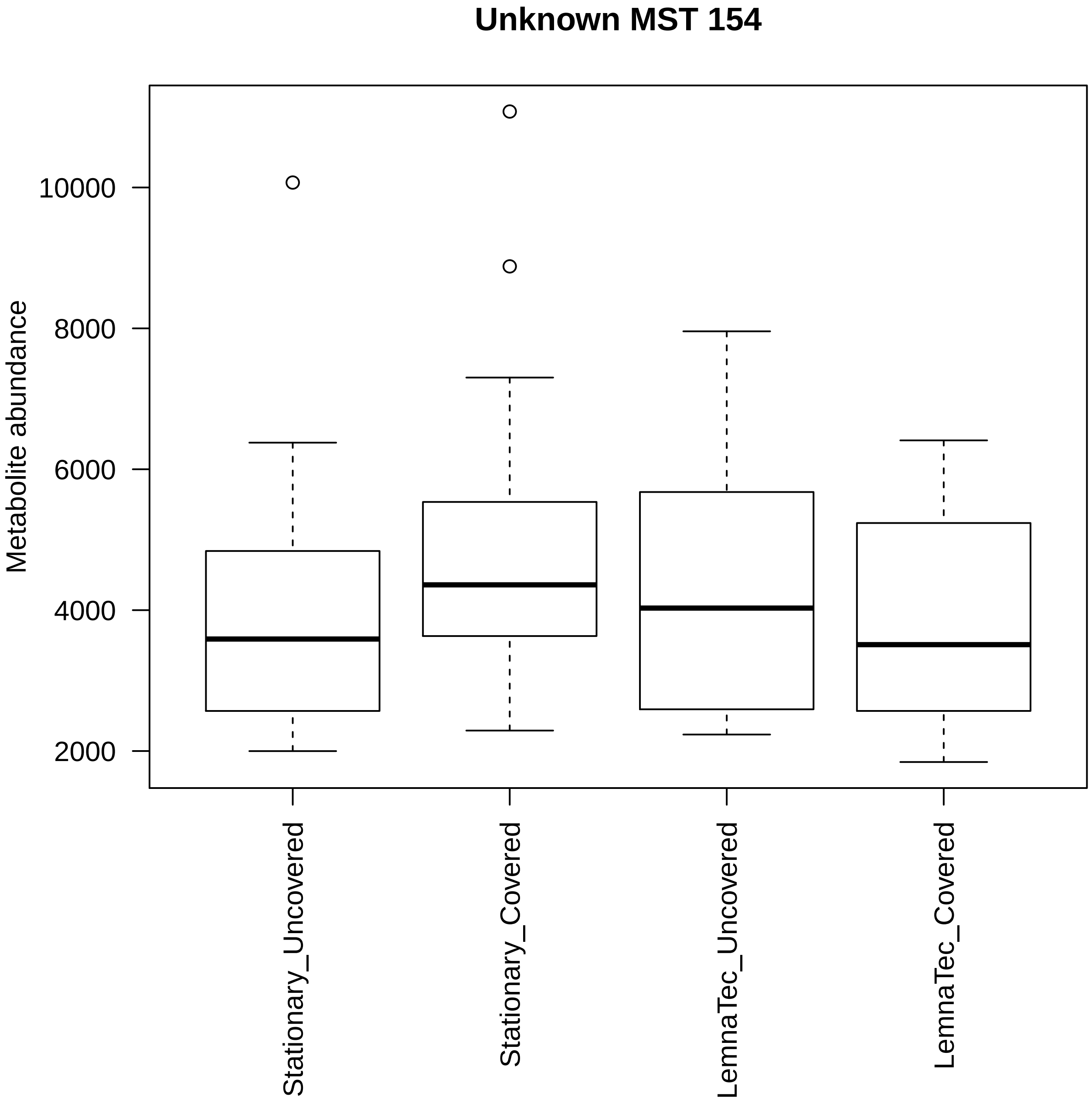

Unknown MST 154
Metabolite abundance
10000
8000
6000
4000
2000
Stationary_Uncovered
Stationary_Covered
LemnaTec_Uncovered
LemnaTec_Covered

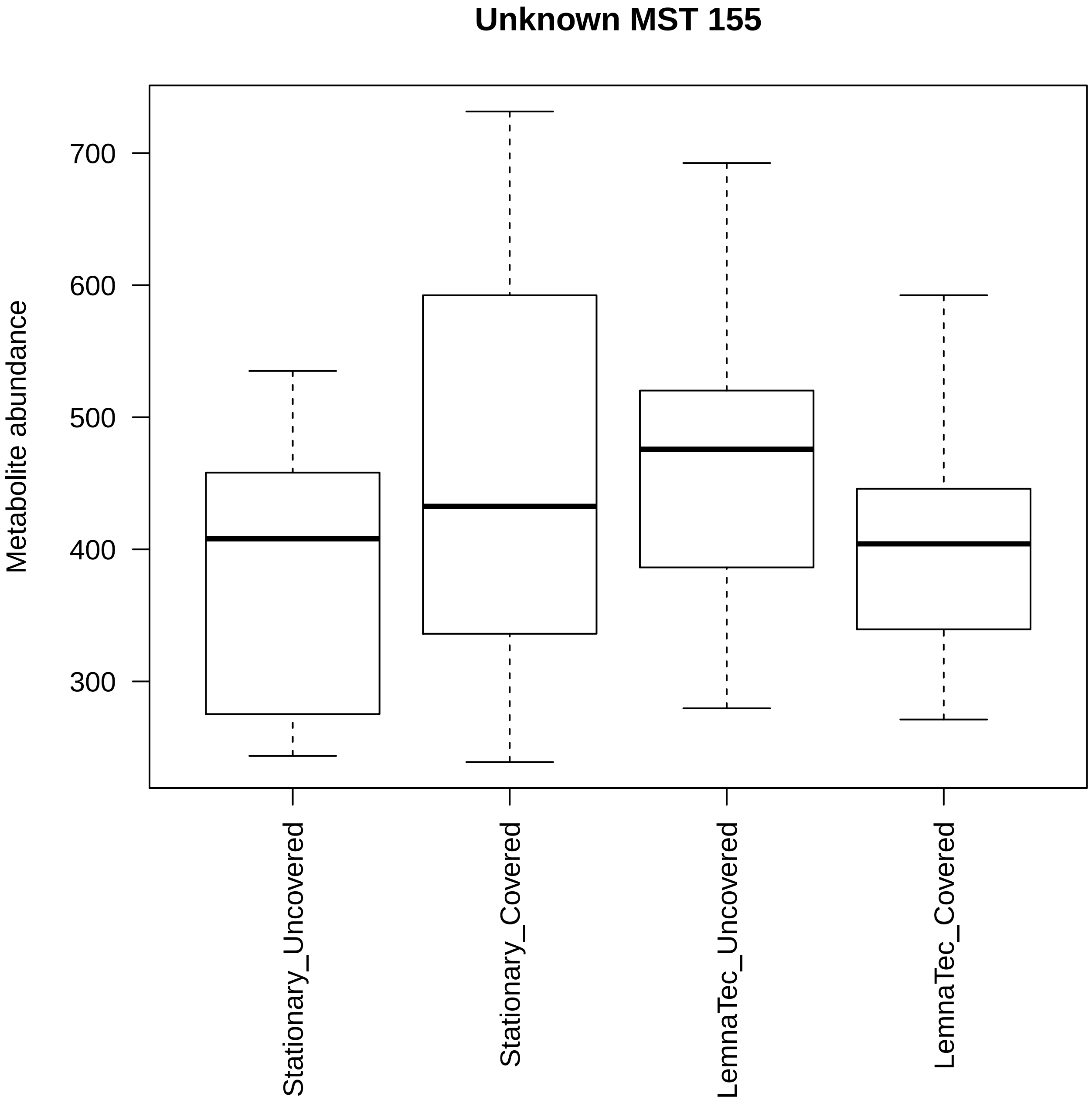

Unknown MST 155
Metabolite abundance
700
600
500
400
300
Stationary_Uncovered
Stationary_Covered
LemnaTec_Uncovered
LemnaTec_Covered

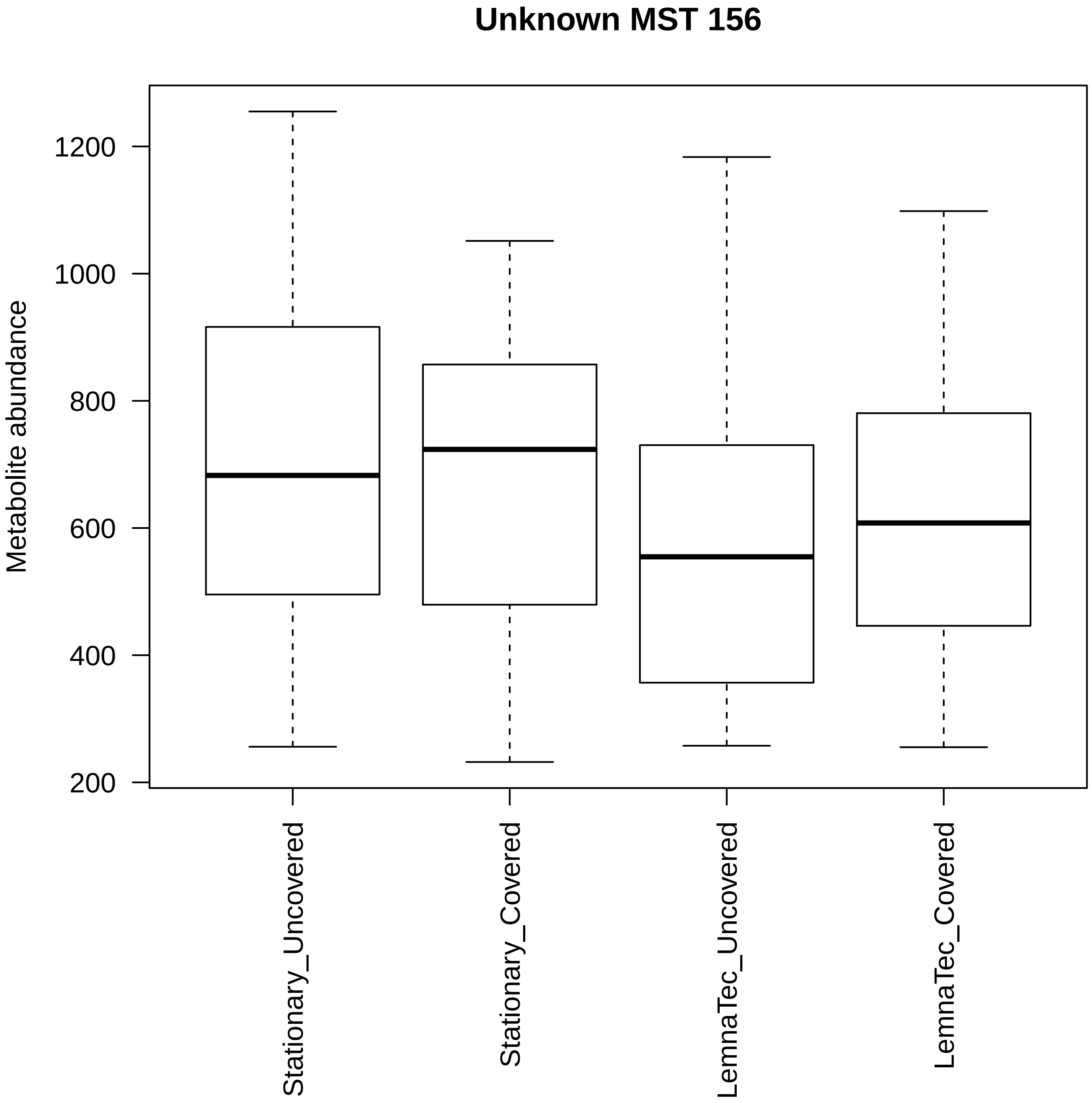

Unknown MST 156
Metabolite abundance
1200
1000
800
600
400
200
Stationary_Uncovered
Stationary_Covered
LemnaTec_Uncovered
LemnaTec_Covered

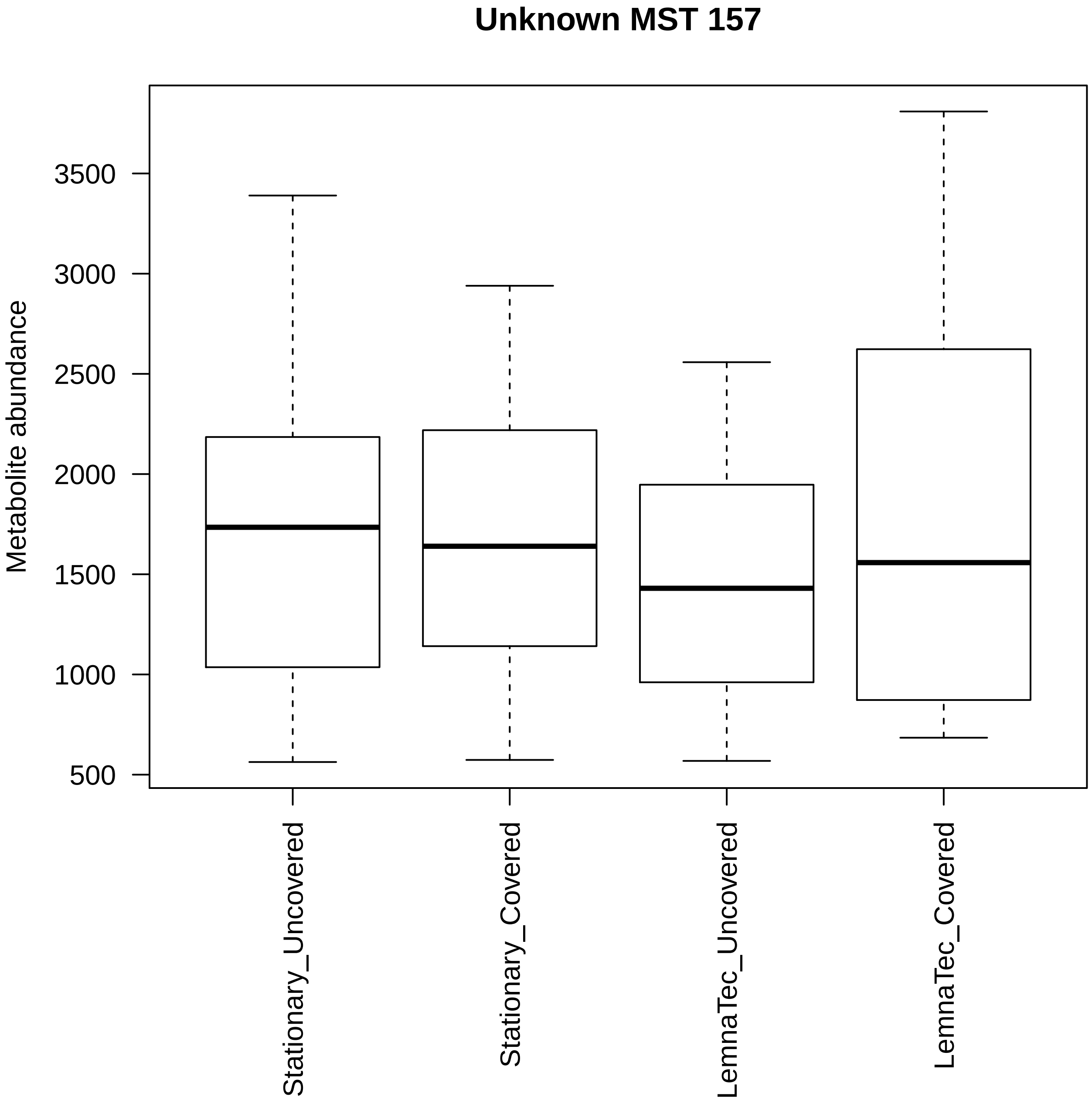
Unknown MST 157
Metabolite abundance
500
1000
1500
2000
2500
3000
3500
Stationary_Uncovered
Stationary_Covered
LemnaTec_Uncovered
LemnaTec_Covered

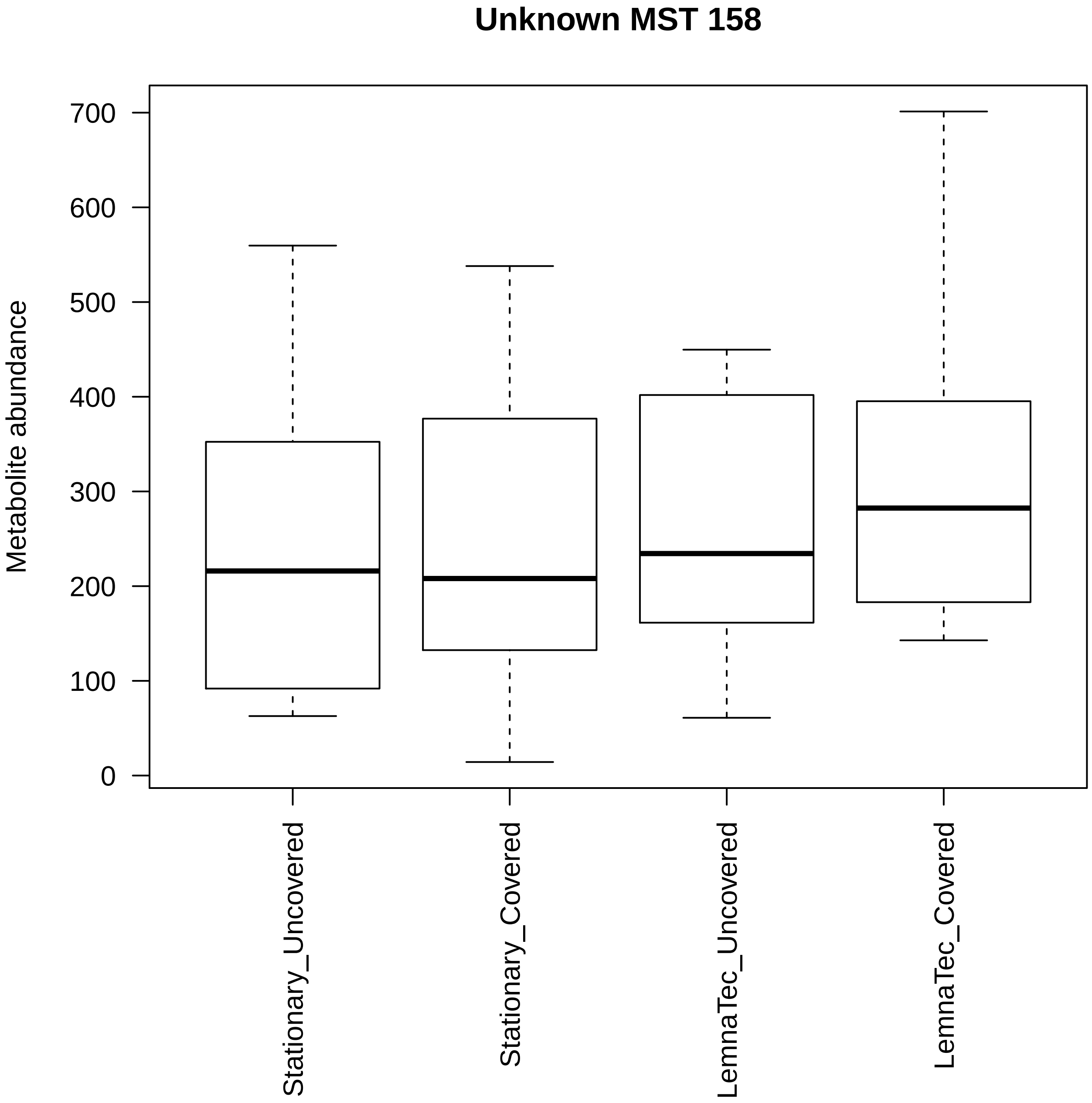
Unknown MST 158
Metabolite abundance
700
600
500
400
300
200
100
0
Stationary_Uncovered
Stationary_Covered
LemnaTec_Uncovered
LemnaTec_Covered

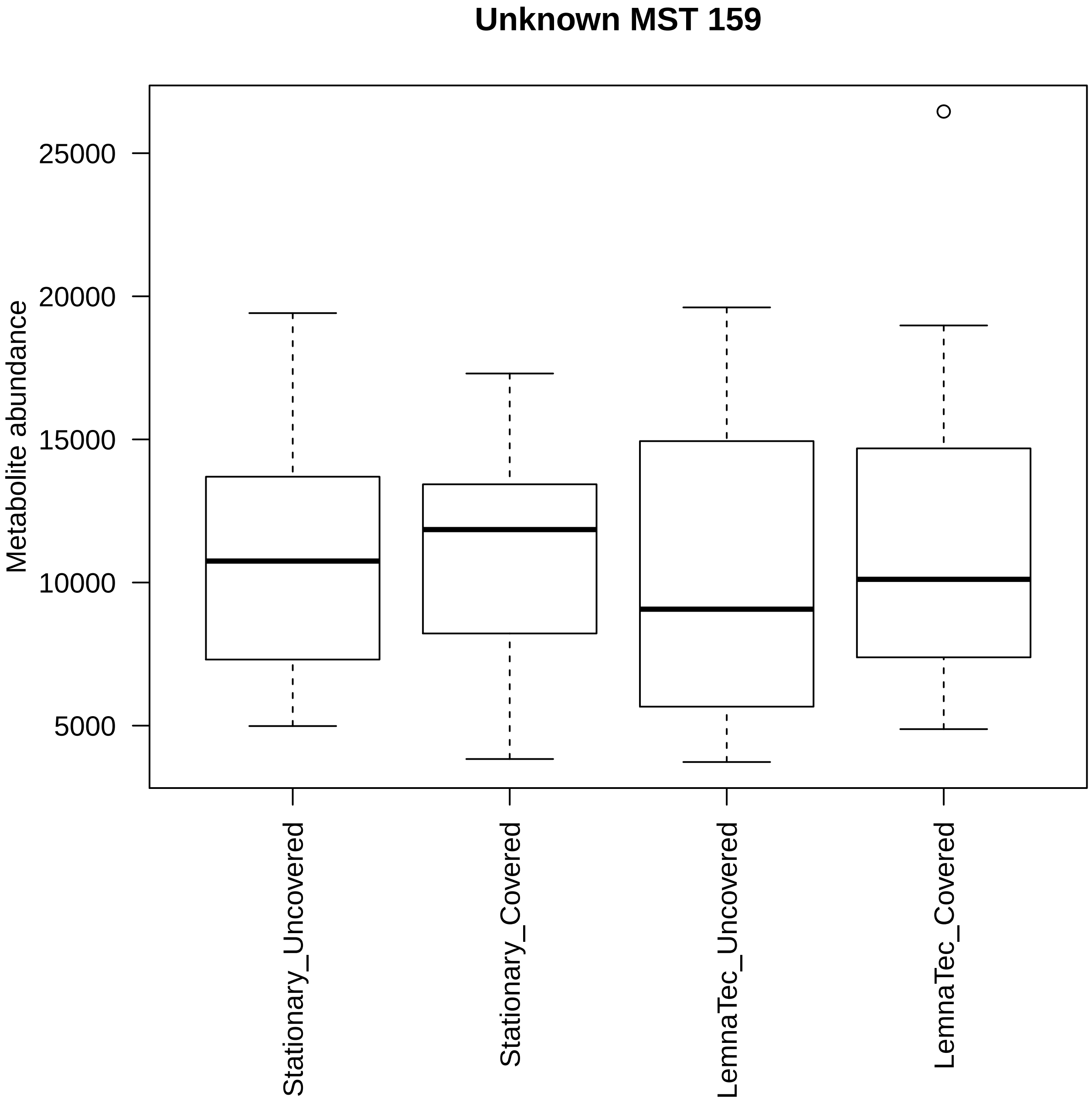

Unknown MST 159
Metabolite abundance
25000
20000
15000
10000
5000
Stationary_Uncovered
Stationary_Covered
LemnaTec_Uncovered
LemnaTec_Covered

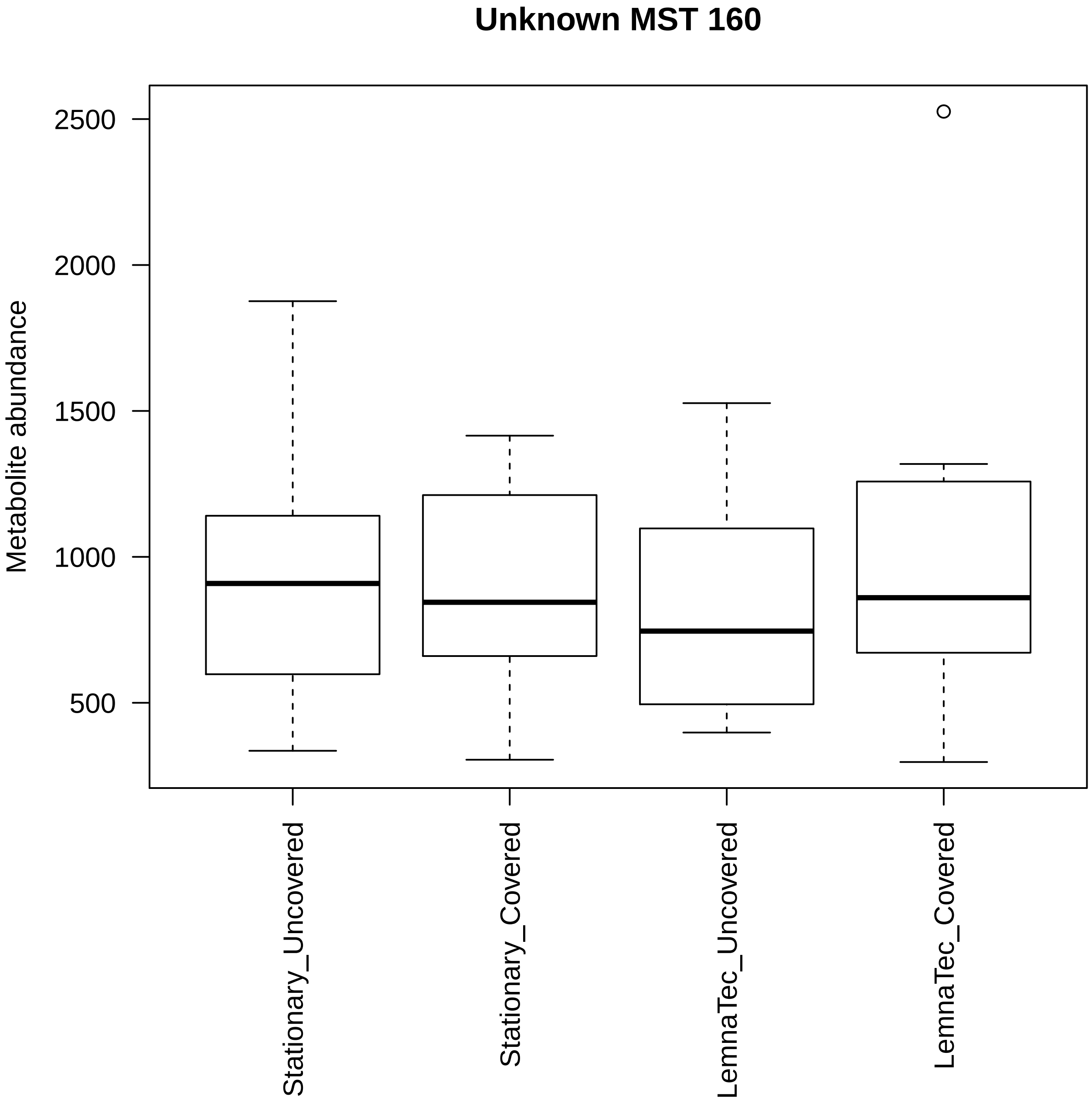
Unknown MST 160
Metabolite abundance
2500
2000
1500
1000
500
Stationary_Uncovered
Stationary_Covered
LemnaTec_Uncovered
LemnaTec_Covered

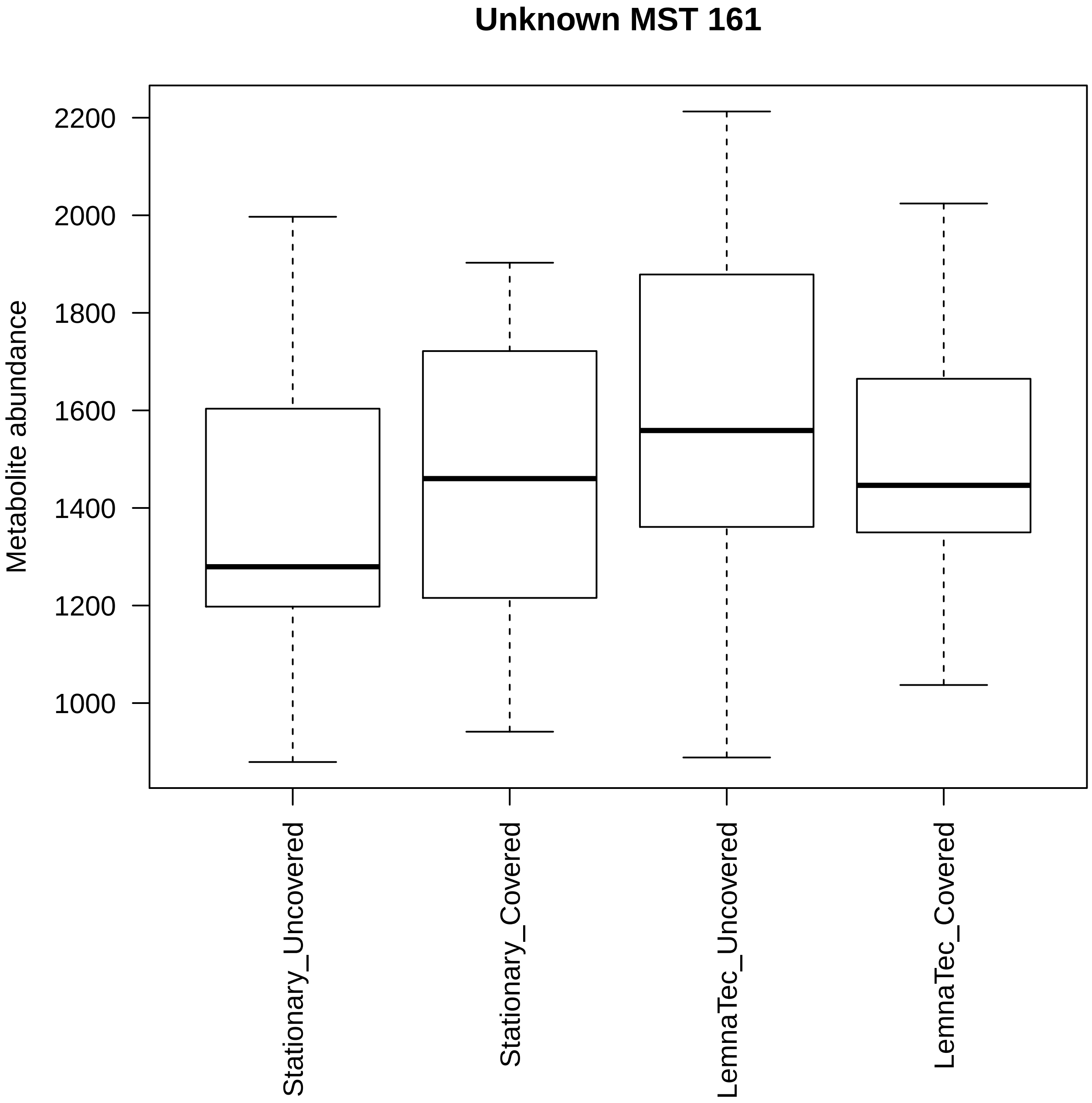

Unknown MST 161
Metabolite abundance
2200
2000
1800
1600
1400
1200
1000
Stationary_Uncovered
Stationary_Covered
LemnaTec_Uncovered
LemnaTec_Covered

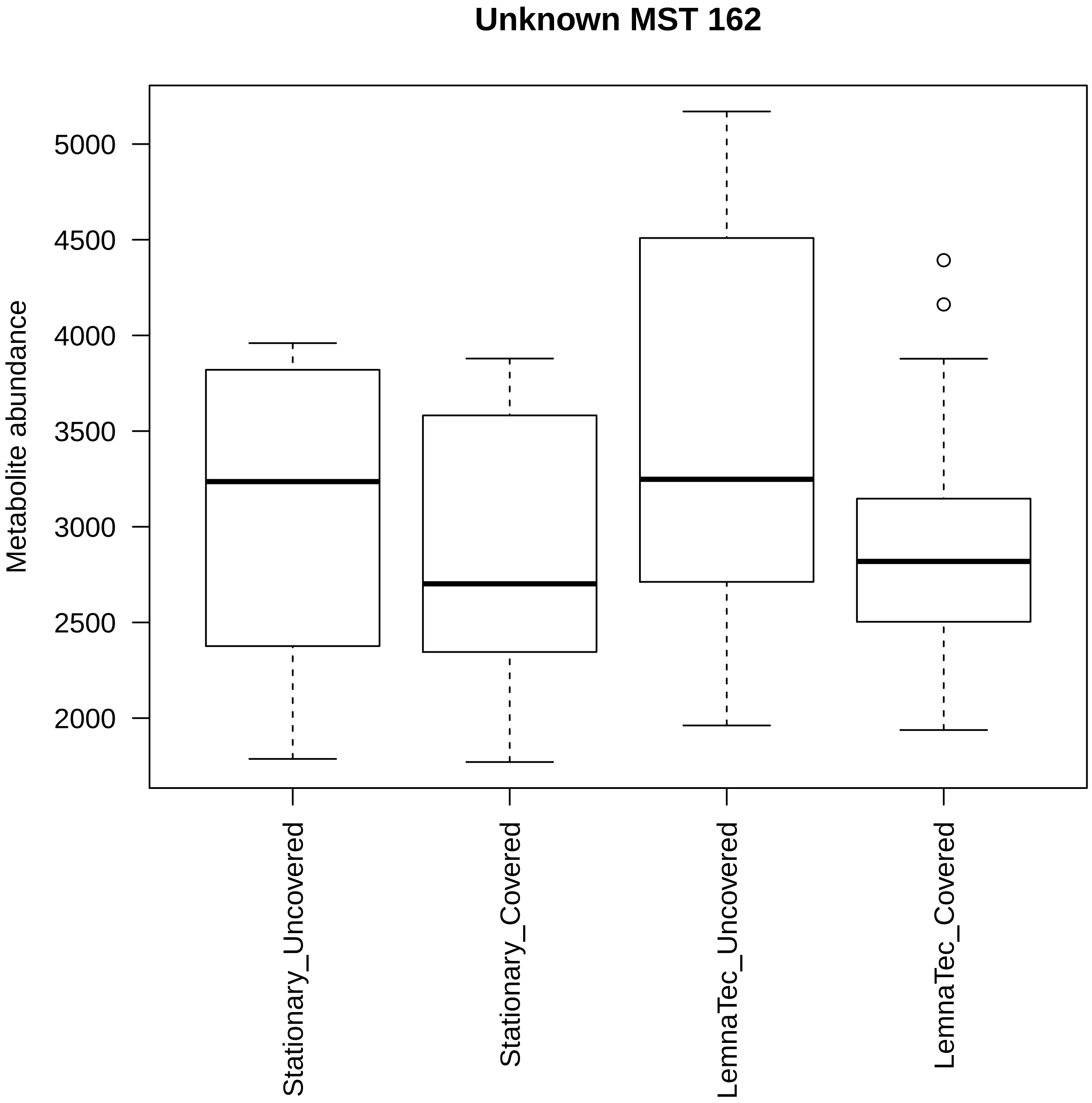

Unknown MST 162
Metabolite abundance
5000
4500
4000
3500
3000
2500
2000
Stationary_Uncovered
Stationary_Covered
LemnaTec_Uncovered
LemnaTec_Covered

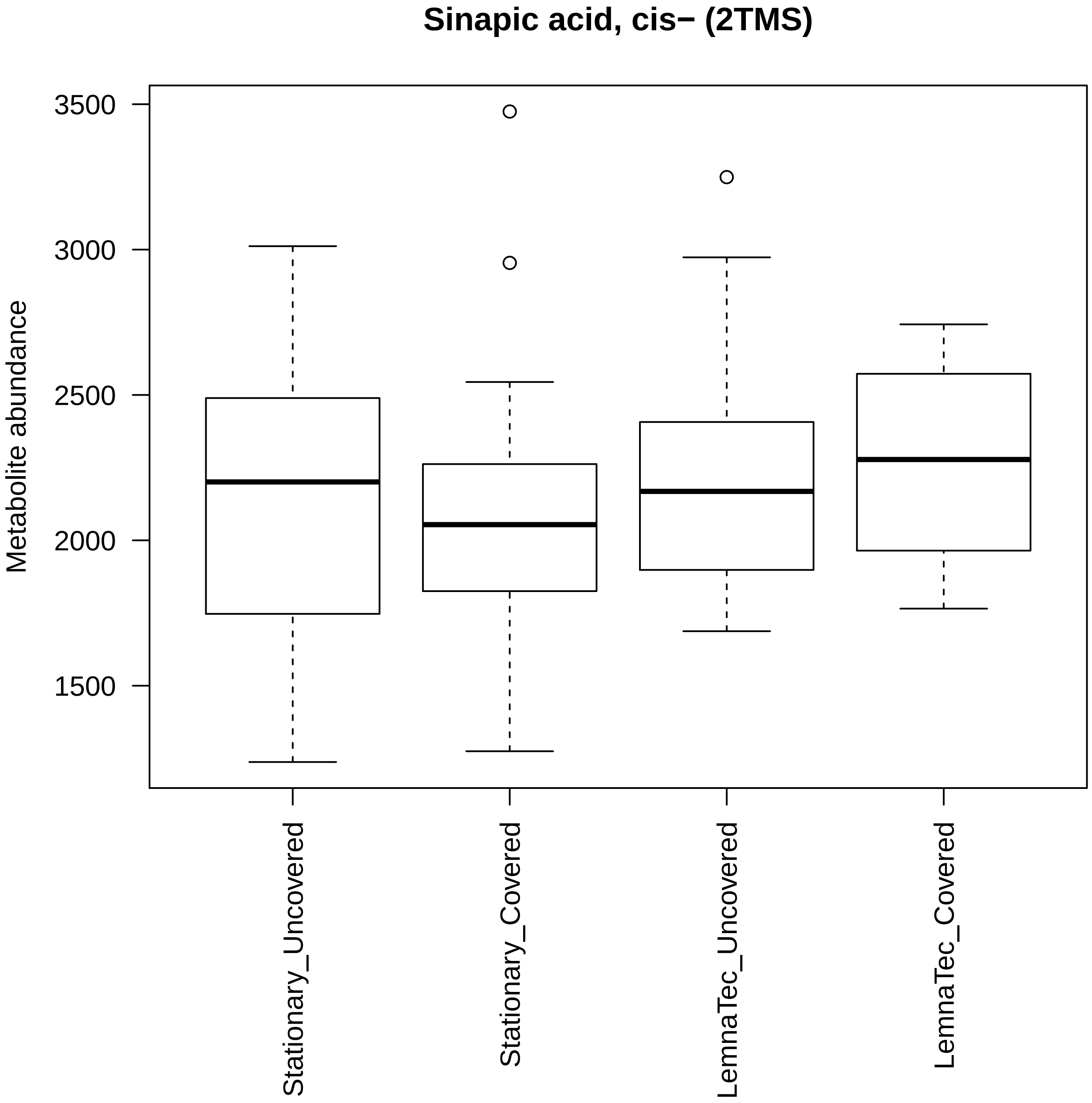
Sinapic acid, cis− (2TMS)
Metabolite abundance
3500
3000
2500
2000
1500
Stationary_Uncovered
Stationary_Covered
LemnaTec_Uncovered
LemnaTec_Covered

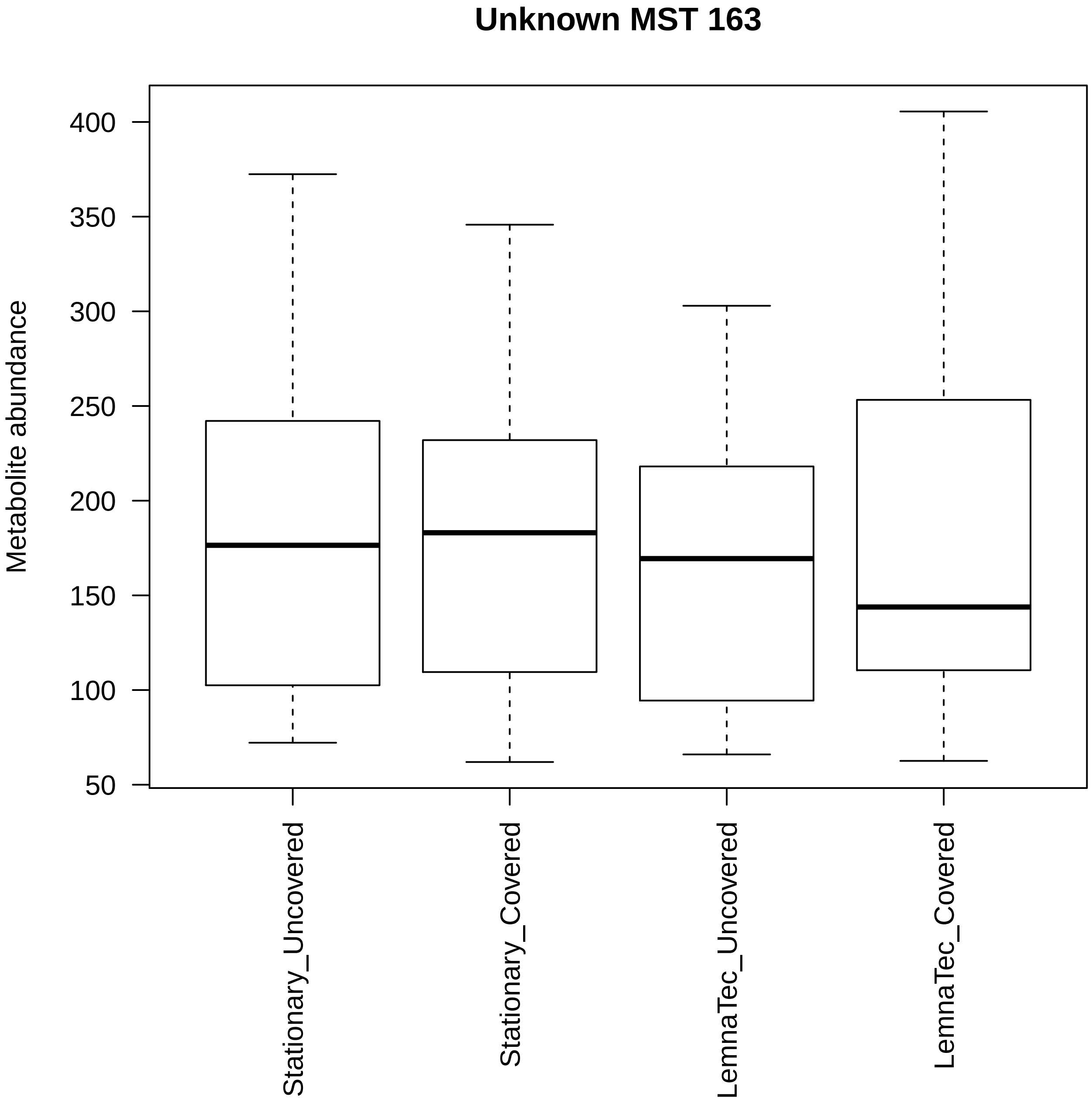

Unknown MST 163
Metabolite abundance
400
350
300
250
200
150
100
50
Stationary_Uncovered
Stationary_Covered
LemnaTec_Uncovered
LemnaTec_Covered

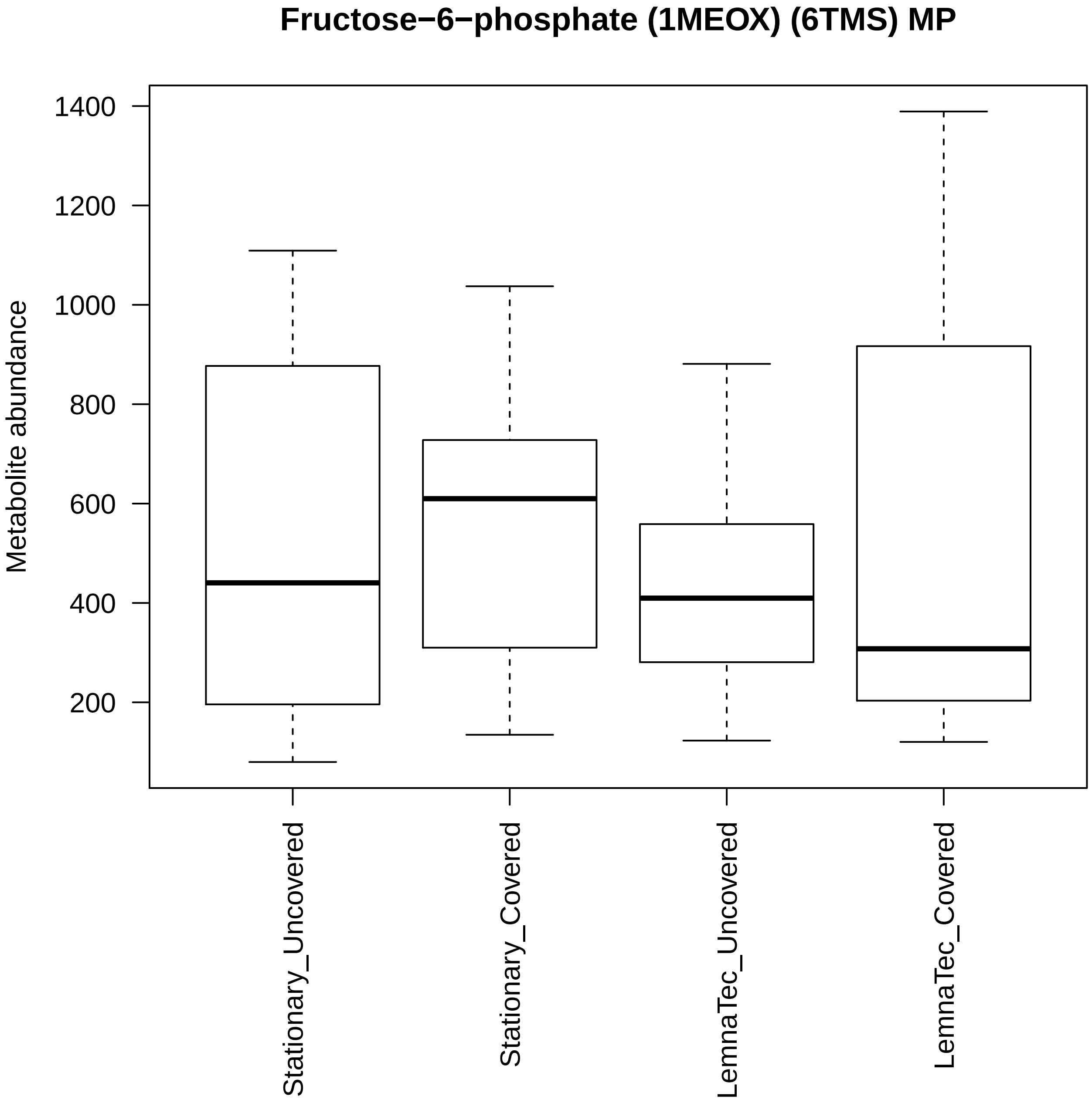
Fructose−6−phosphate (1MEOX) (6TMS) MP
Metabolite abundance
1400
1200
1000
800
600
400
200
Stationary_Uncovered
Stationary_Covered
LemnaTec_Uncovered
LemnaTec_Covered

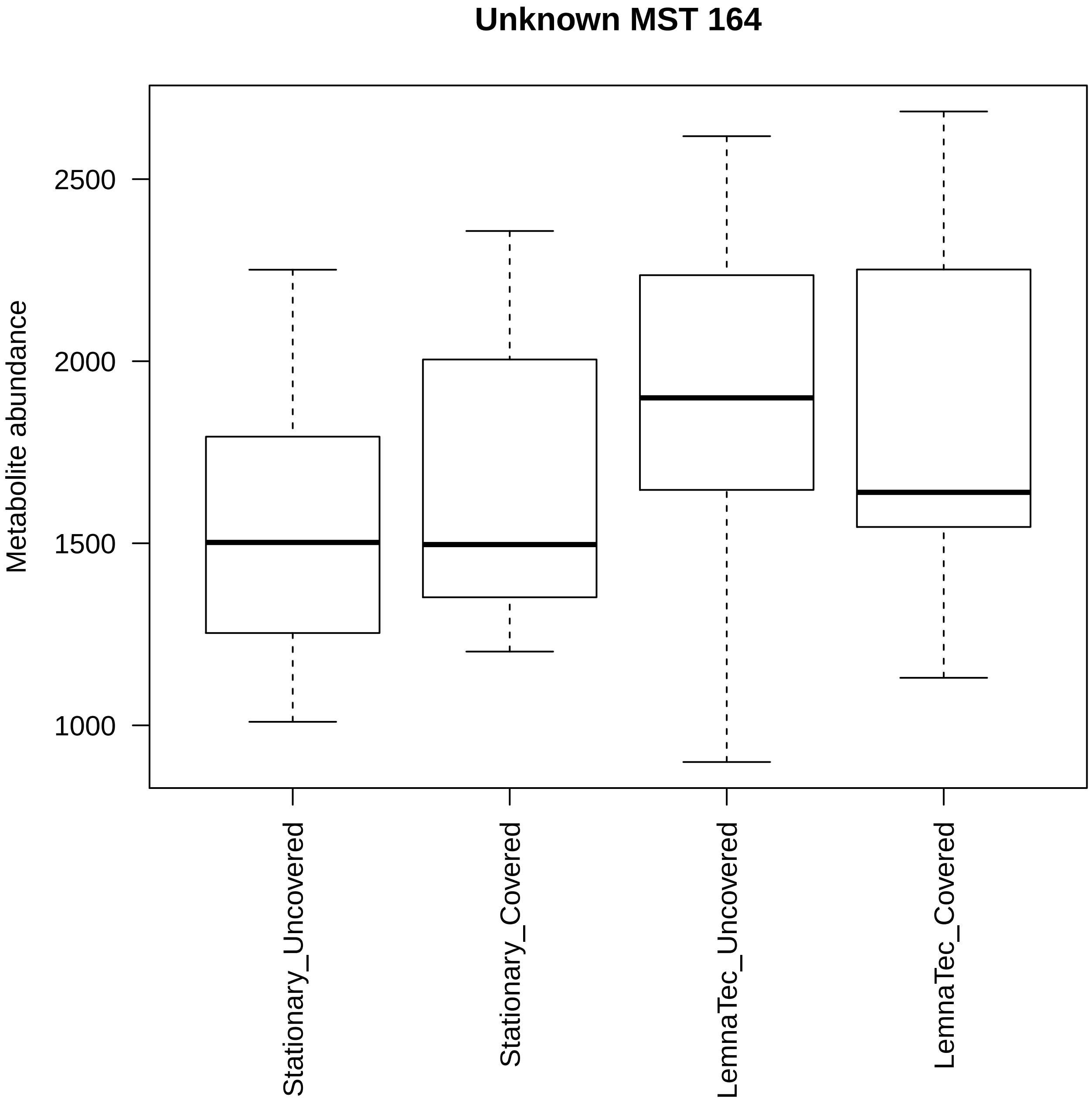
Unknown MST 164
Metabolite abundance
2500
2000
1500
1000
Stationary_Uncovered
Stationary_Covered
LemnaTec_Uncovered
LemnaTec_Covered

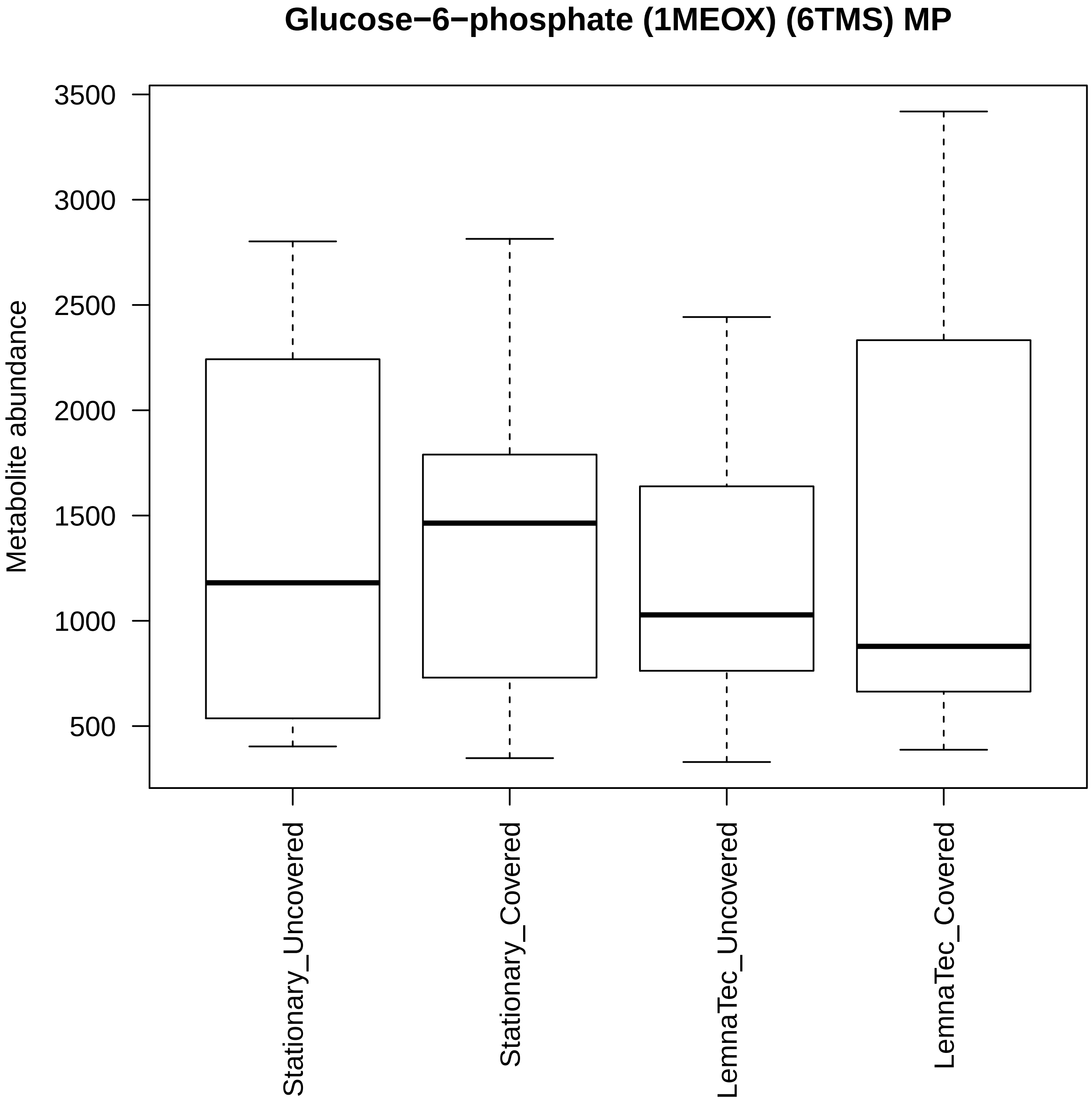
Glucose−6−phosphate (1MEOX) (6TMS) MP
Metabolite abundance
3500
3000
2500
2000
1500
1000
500
Stationary_Uncovered
Stationary_Covered
LemnaTec_Uncovered
LemnaTec_Covered

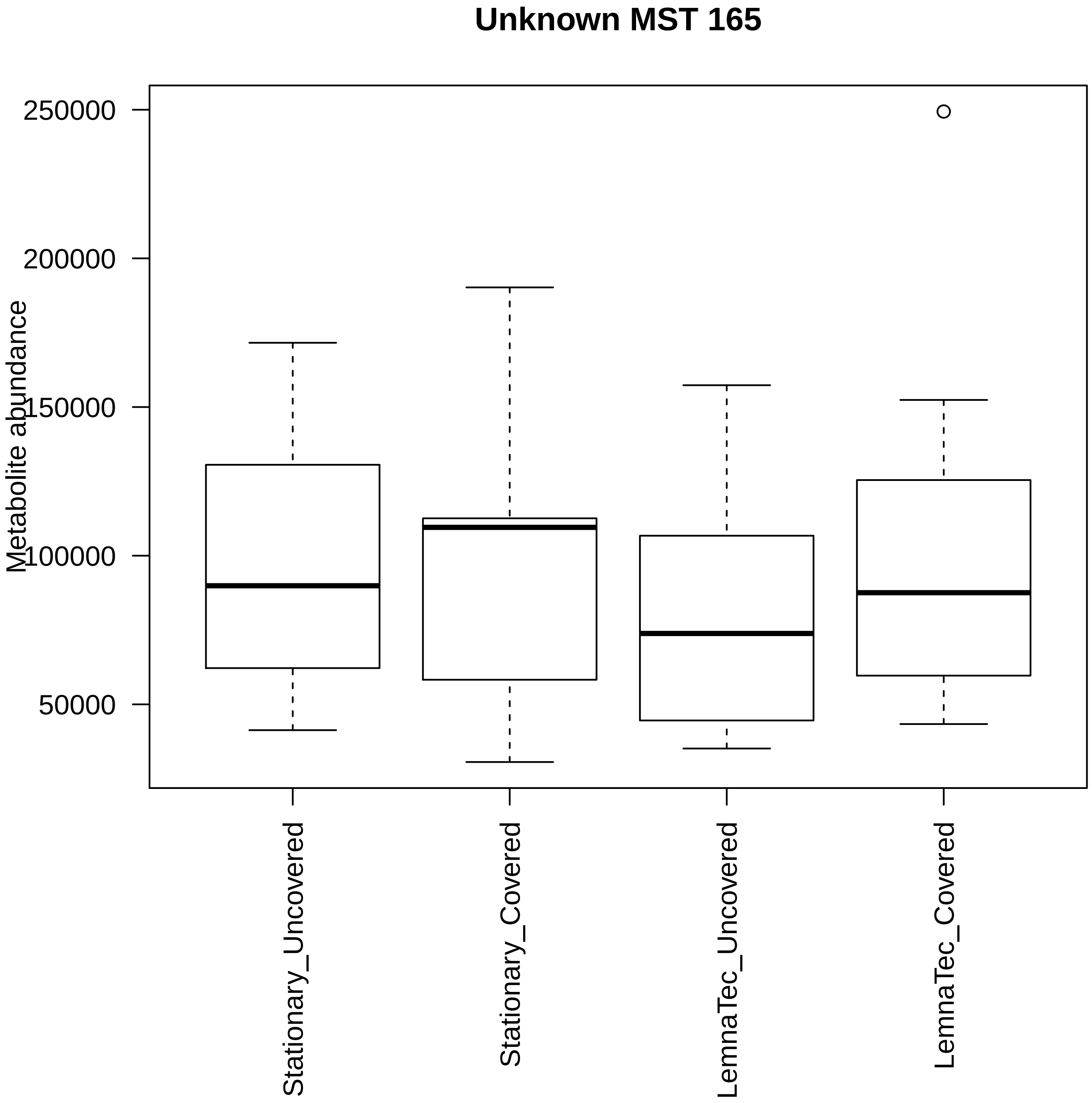
Unknown MST 165
Metabolite abundance
250000
200000
150000
100000
50000
Stationary_Uncovered
Stationary_Covered
LemnaTec_Uncovered
LemnaTec_Covered

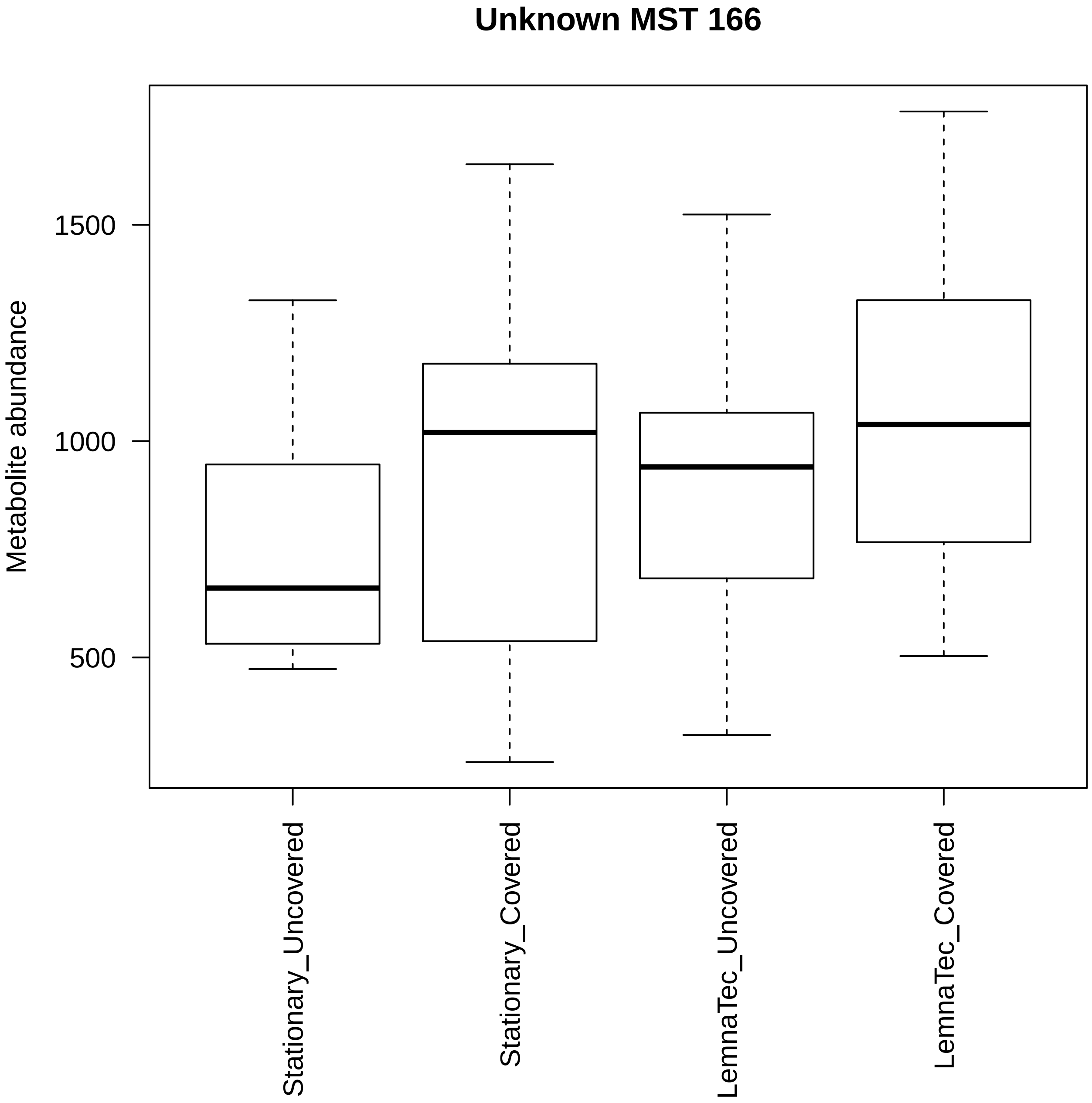
Unknown MST 166
Metabolite abundance
1500
1000
500
Stationary_Uncovered
Stationary_Covered
LemnaTec_Uncovered
LemnaTec_Covered

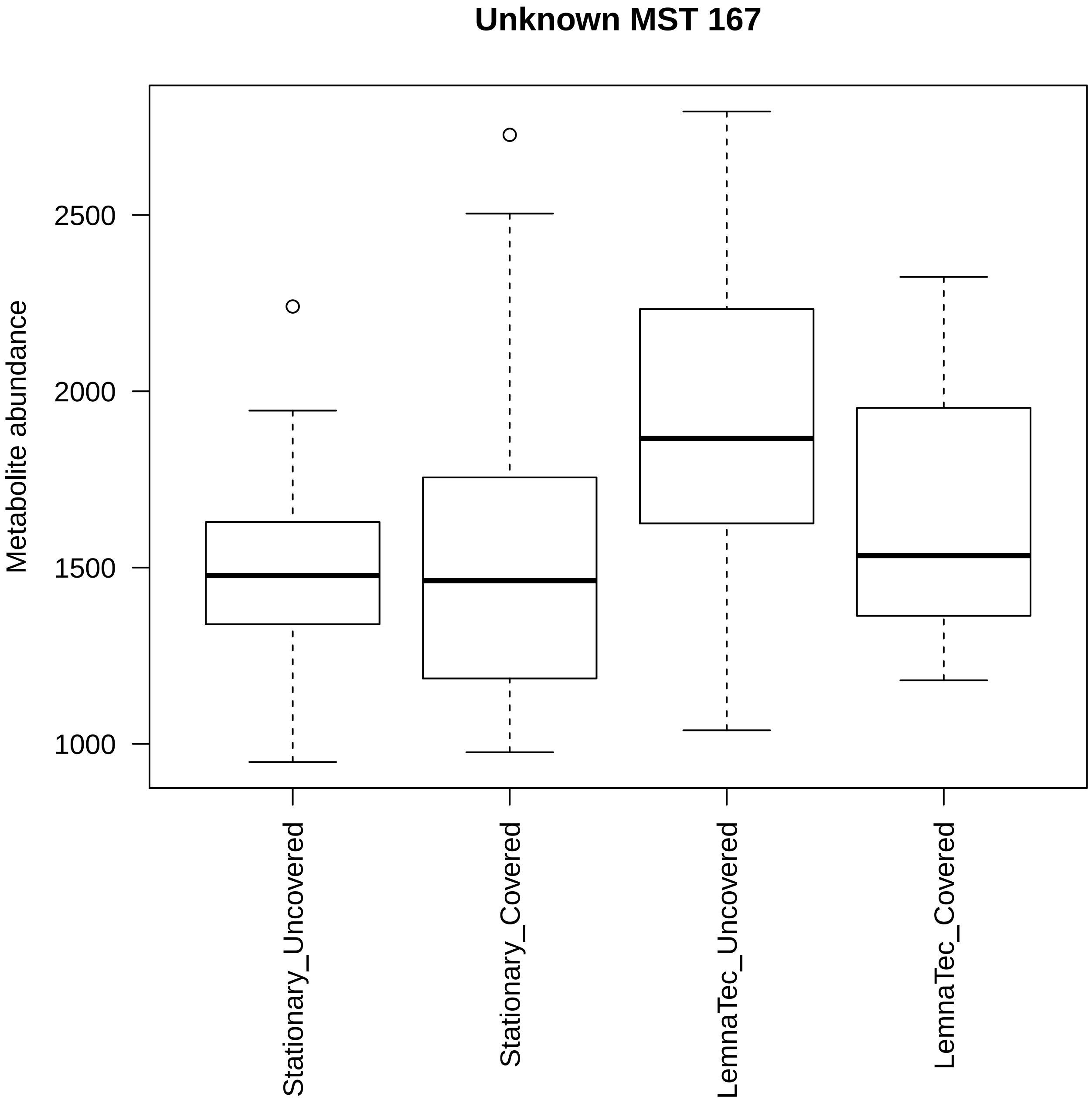
Unknown MST 167
Metabolite abundance
2500
2000
1500
1000
Stationary_Uncovered
Stationary_Covered
LemnaTec_Uncovered
LemnaTec_Covered

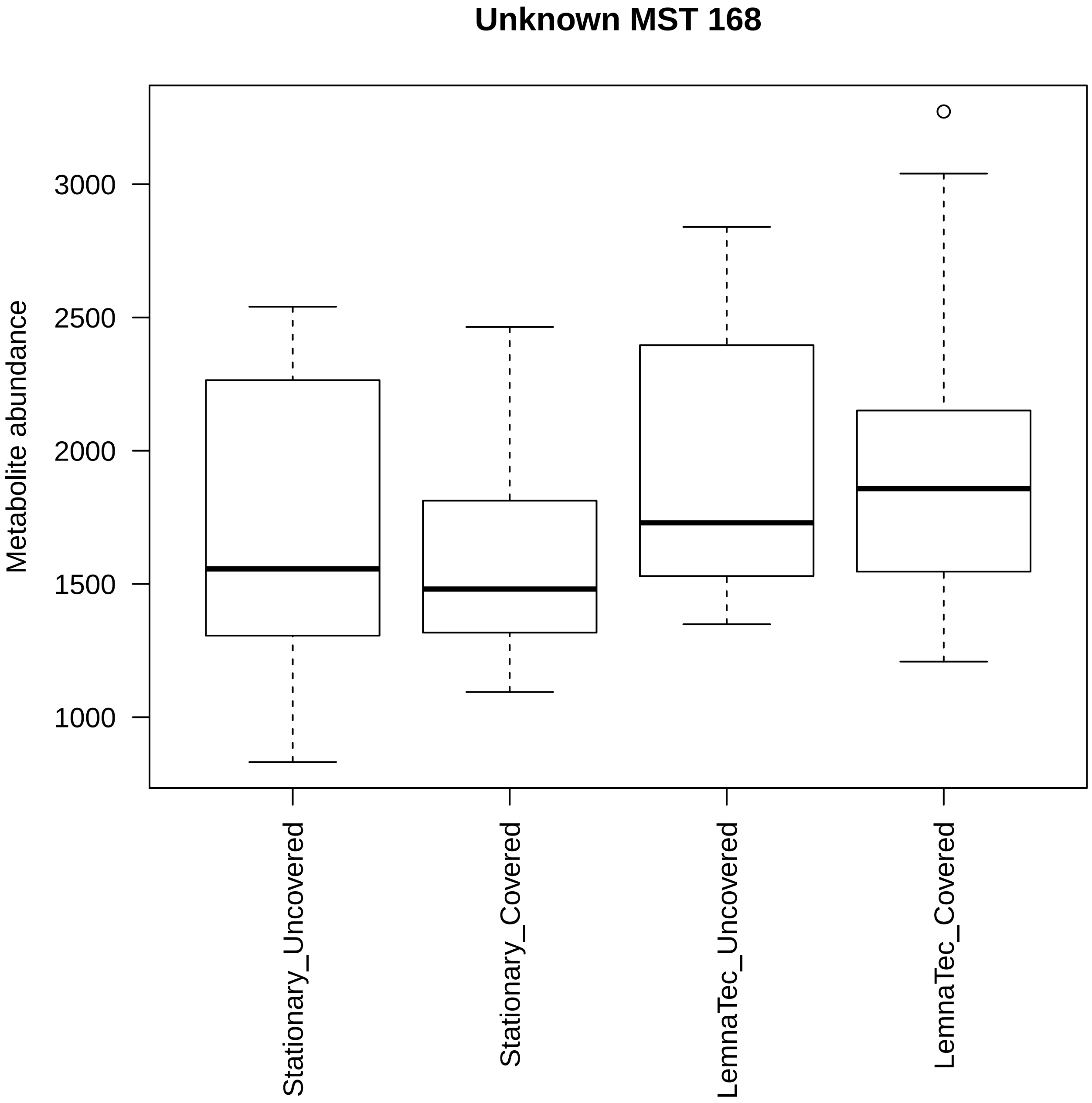

Unknown MST 168
Metabolite abundance
3000
2500
2000
1500
1000
Stationary_Uncovered
Stationary_Covered
LemnaTec_Uncovered
LemnaTec_Covered

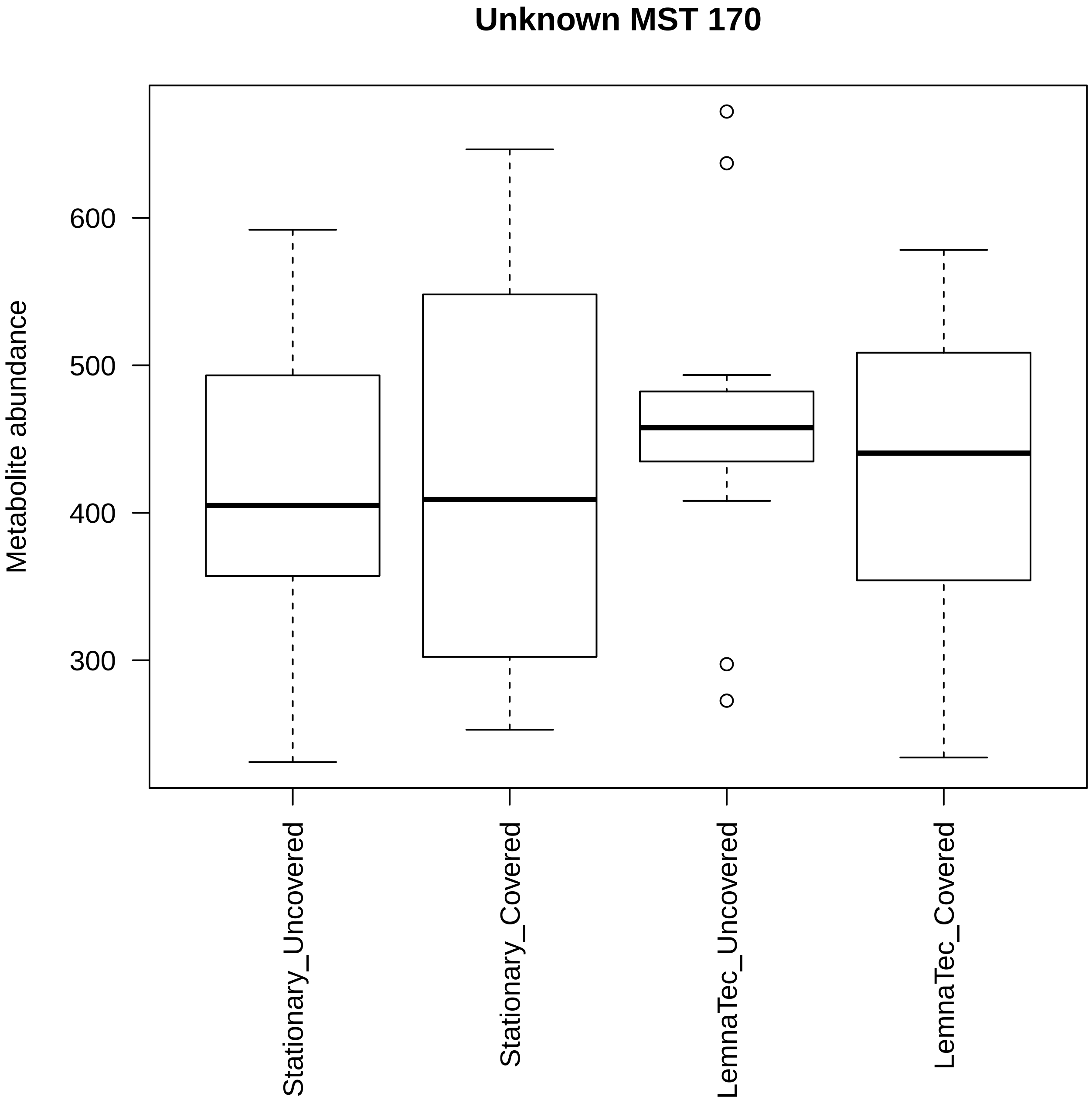
Unknown MST 170
Metabolite abundance
600
500
400
300
Stationary_Uncovered
Stationary_Covered
LemnaTec_Uncovered
LemnaTec_Covered

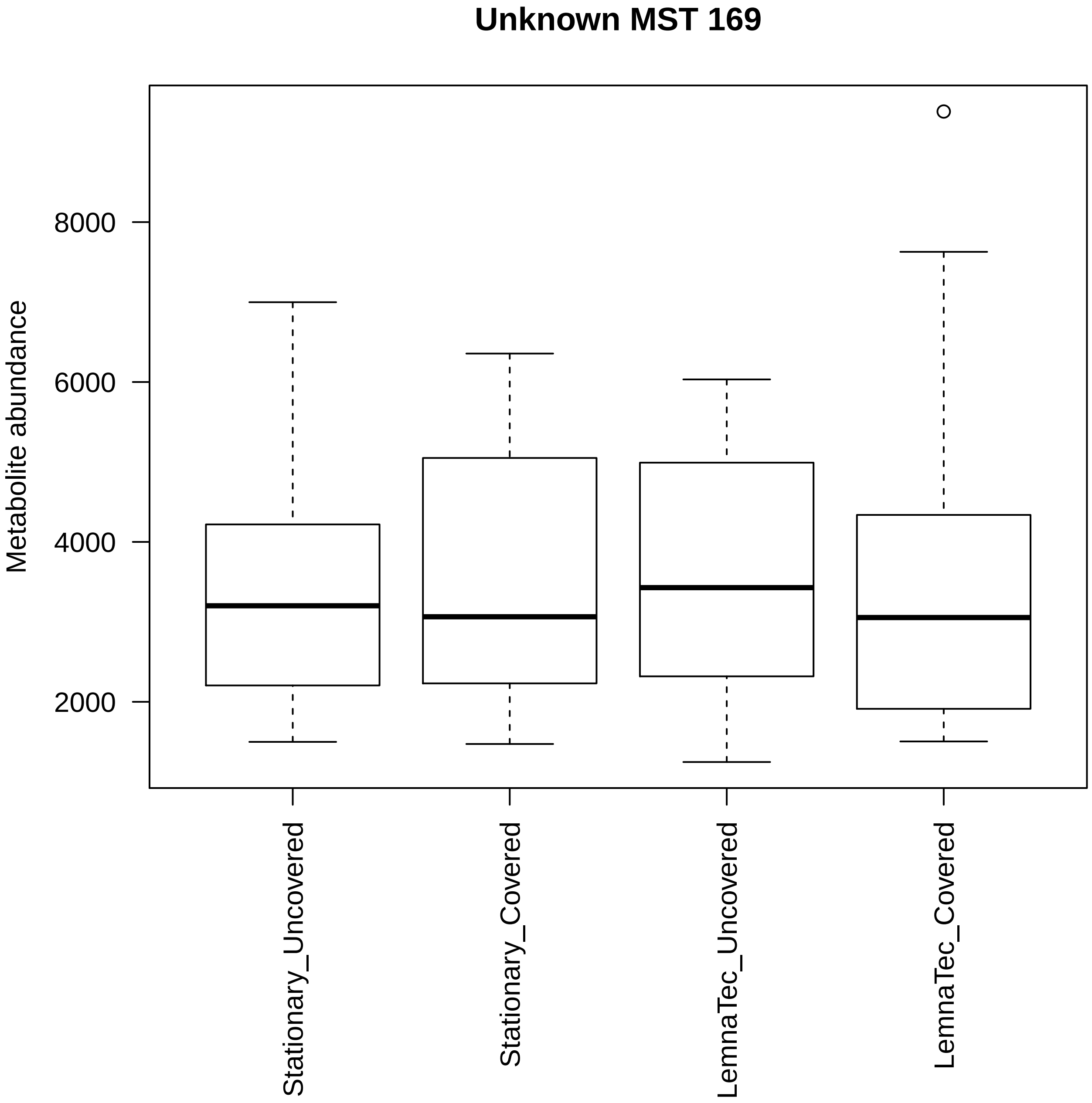
Unknown MST 169
Metabolite abundance
8000
6000
4000
2000
Stationary_Uncovered
Stationary_Covered
LemnaTec_Uncovered
LemnaTec_Covered

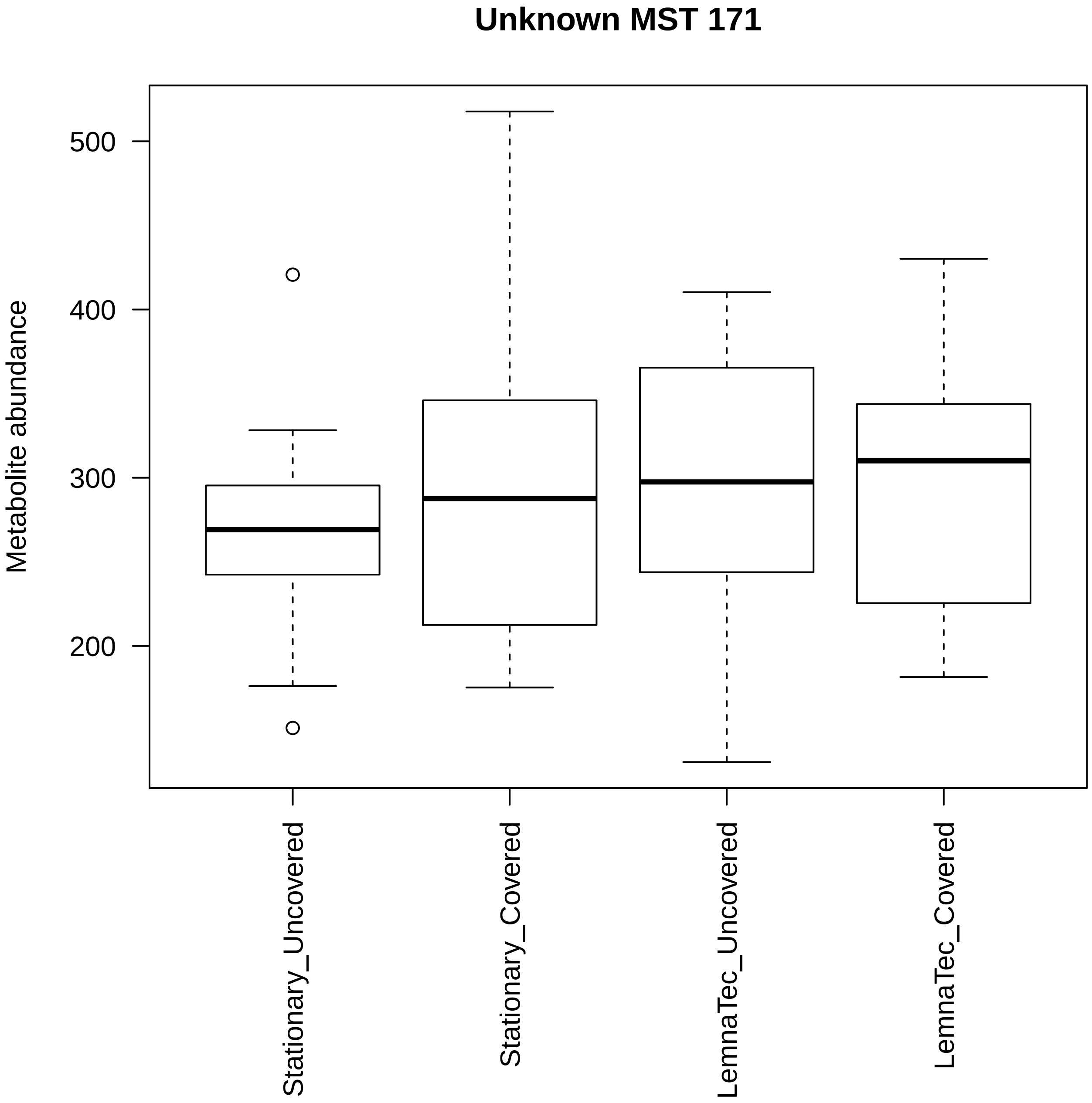
Unknown MST 171
Metabolite abundance
500
400
300
200
Stationary_Uncovered
Stationary_Covered
LemnaTec_Uncovered
LemnaTec_Covered

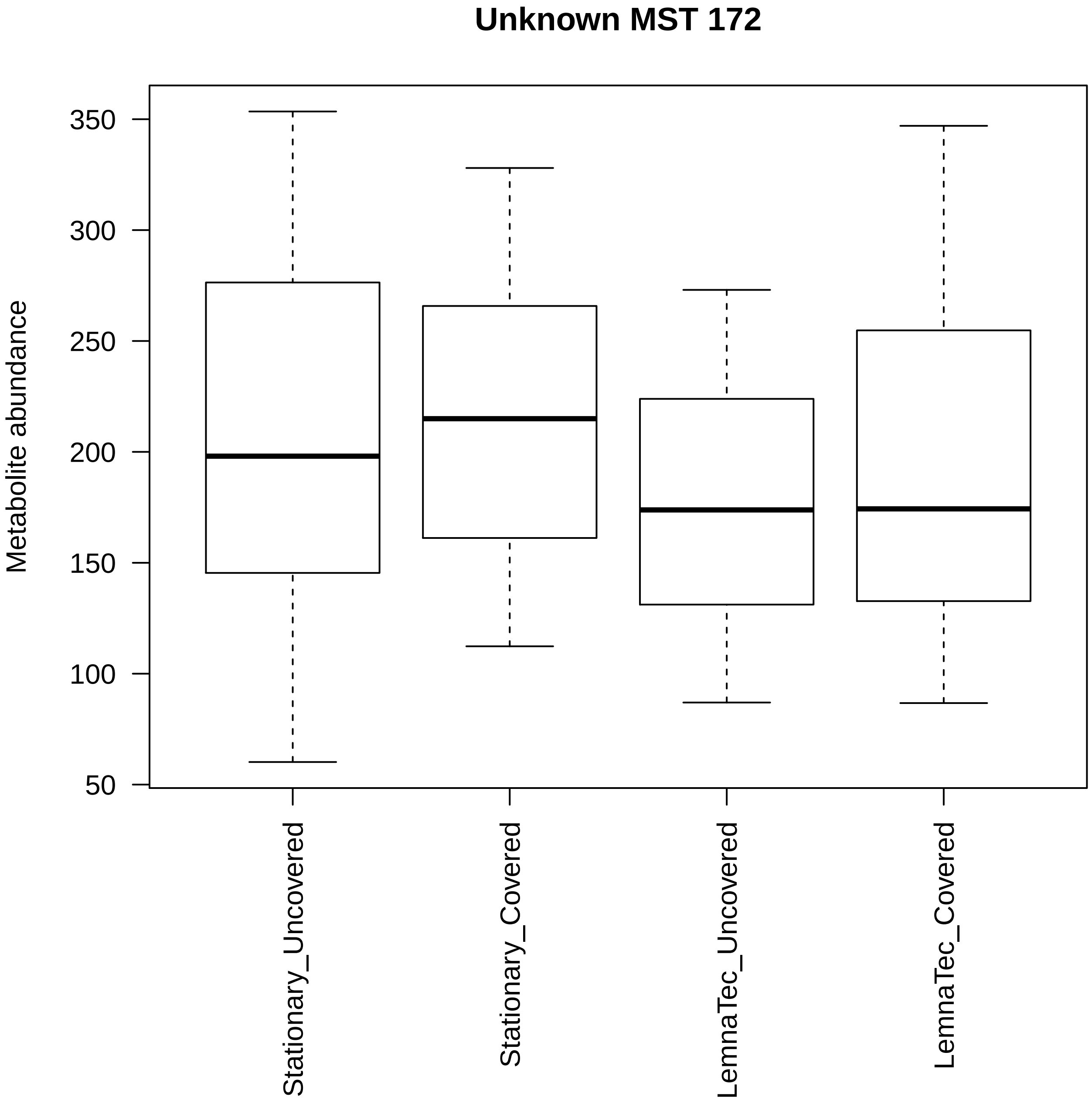
Unknown MST 172
Metabolite abundance
350
300
250
200
150
100
50
Stationary_Uncovered
Stationary_Covered
LemnaTec_Uncovered
LemnaTec_Covered

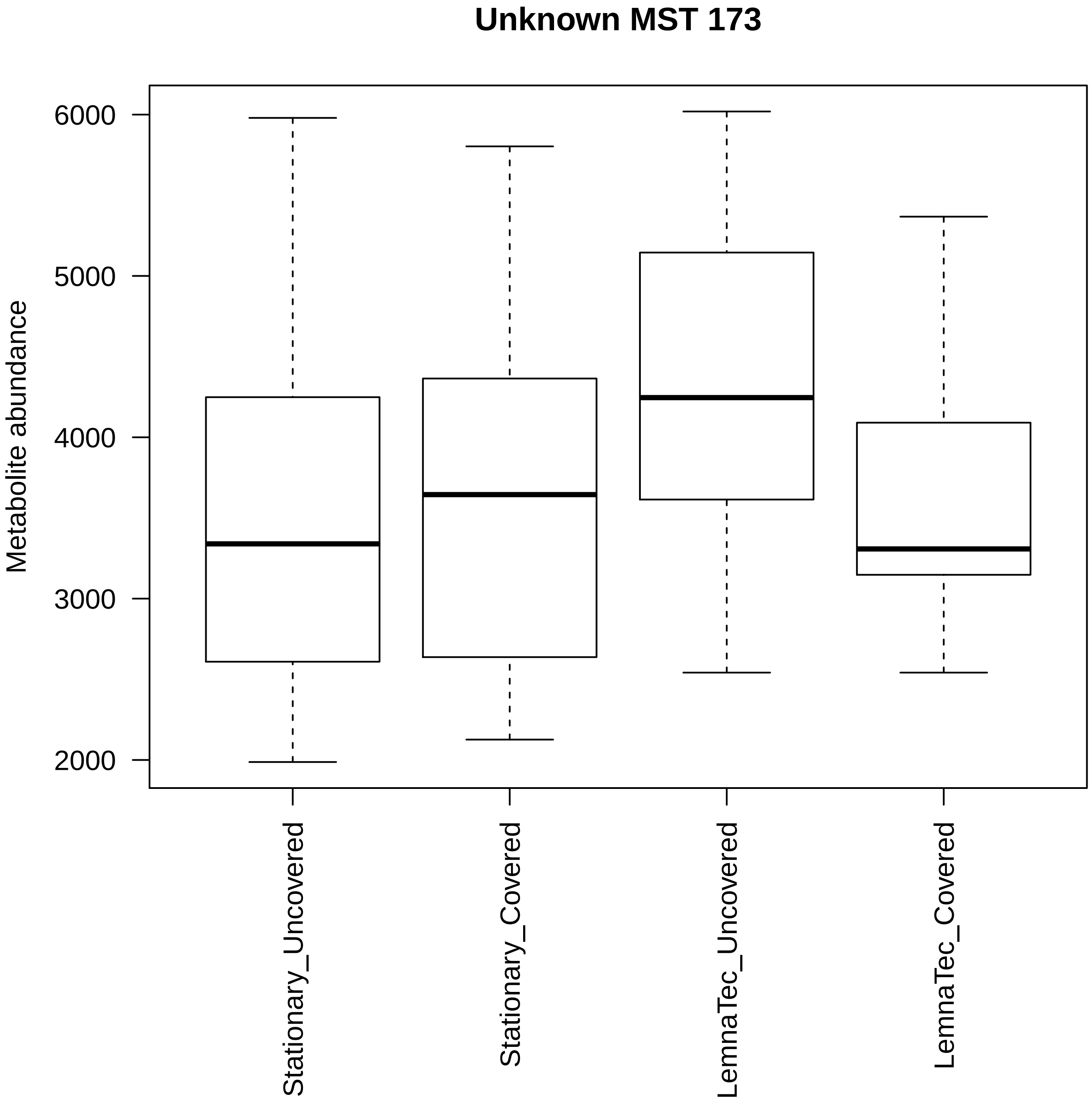
Unknown MST 173
Metabolite abundance
6000
5000
4000
3000
2000
Stationary_Uncovered
Stationary_Covered
LemnaTec_Uncovered
LemnaTec_Covered

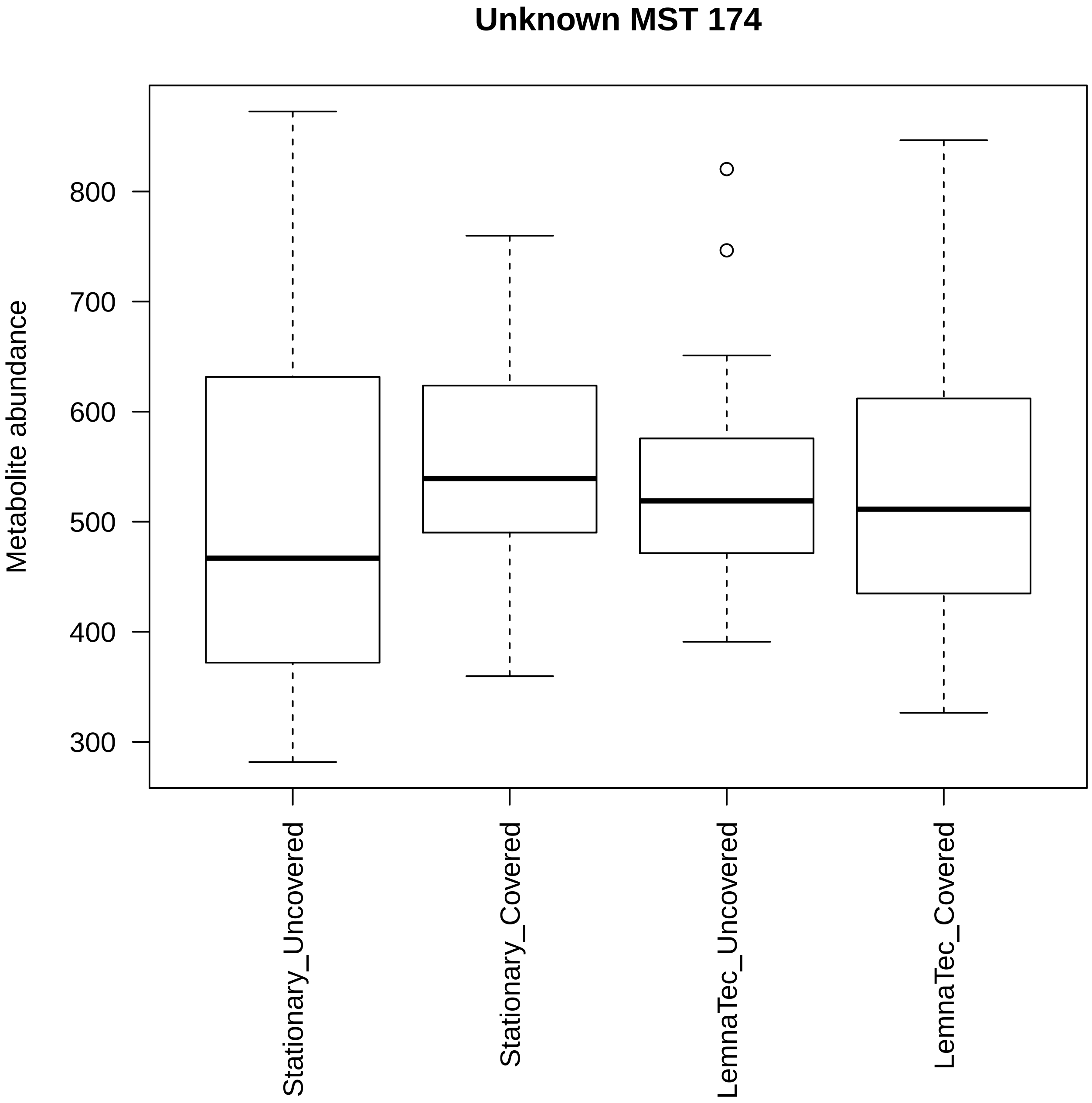

Unknown MST 174
Metabolite abundance
800
700
600
500
400
300
Stationary_Uncovered
Stationary_Covered
LemnaTec_Uncovered
LemnaTec_Covered

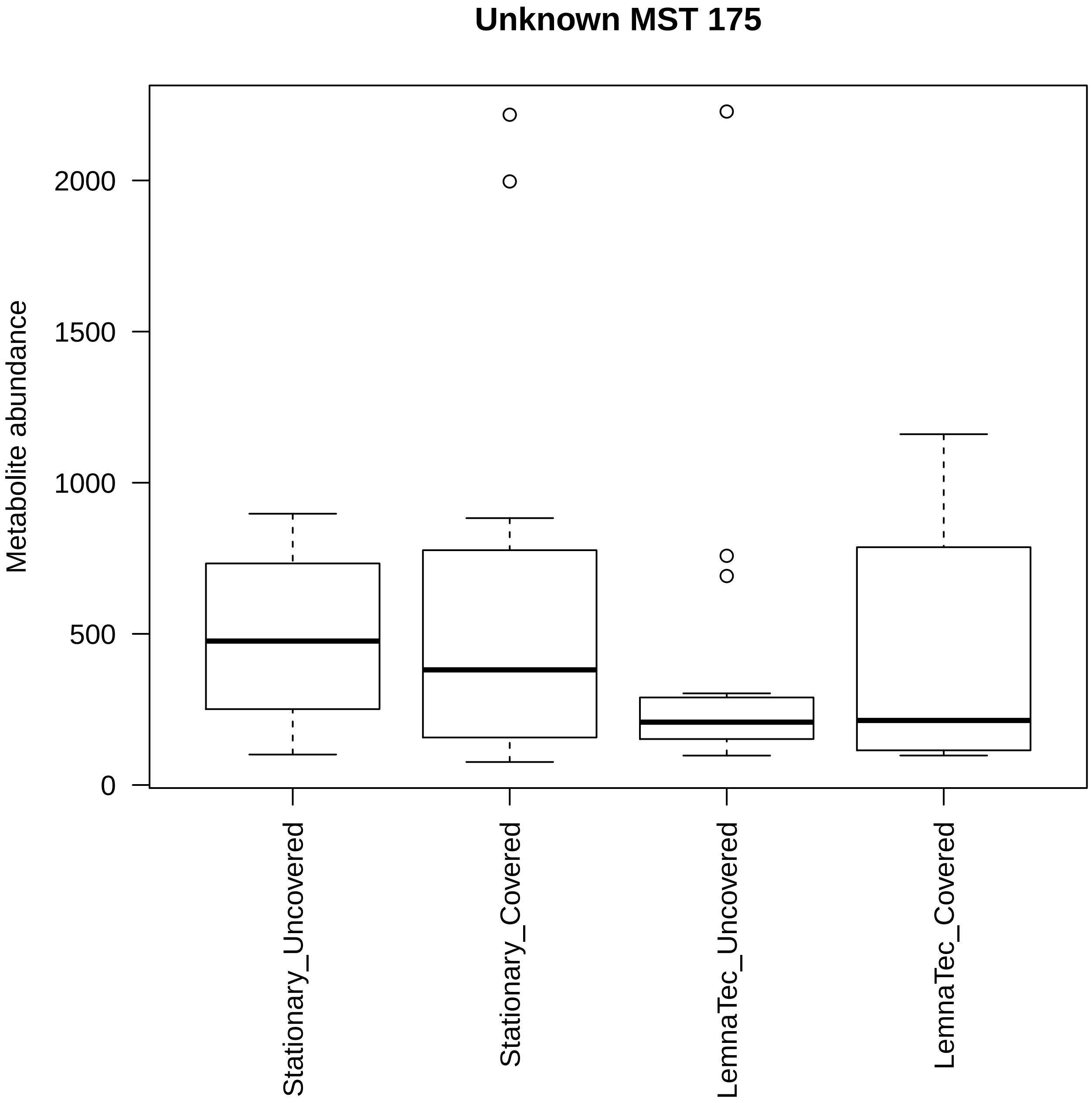
Unknown MST 175
Metabolite abundance
0
500
1000
1500
2000
Stationary_Uncovered
Stationary_Covered
LemnaTec_Uncovered
LemnaTec_Covered

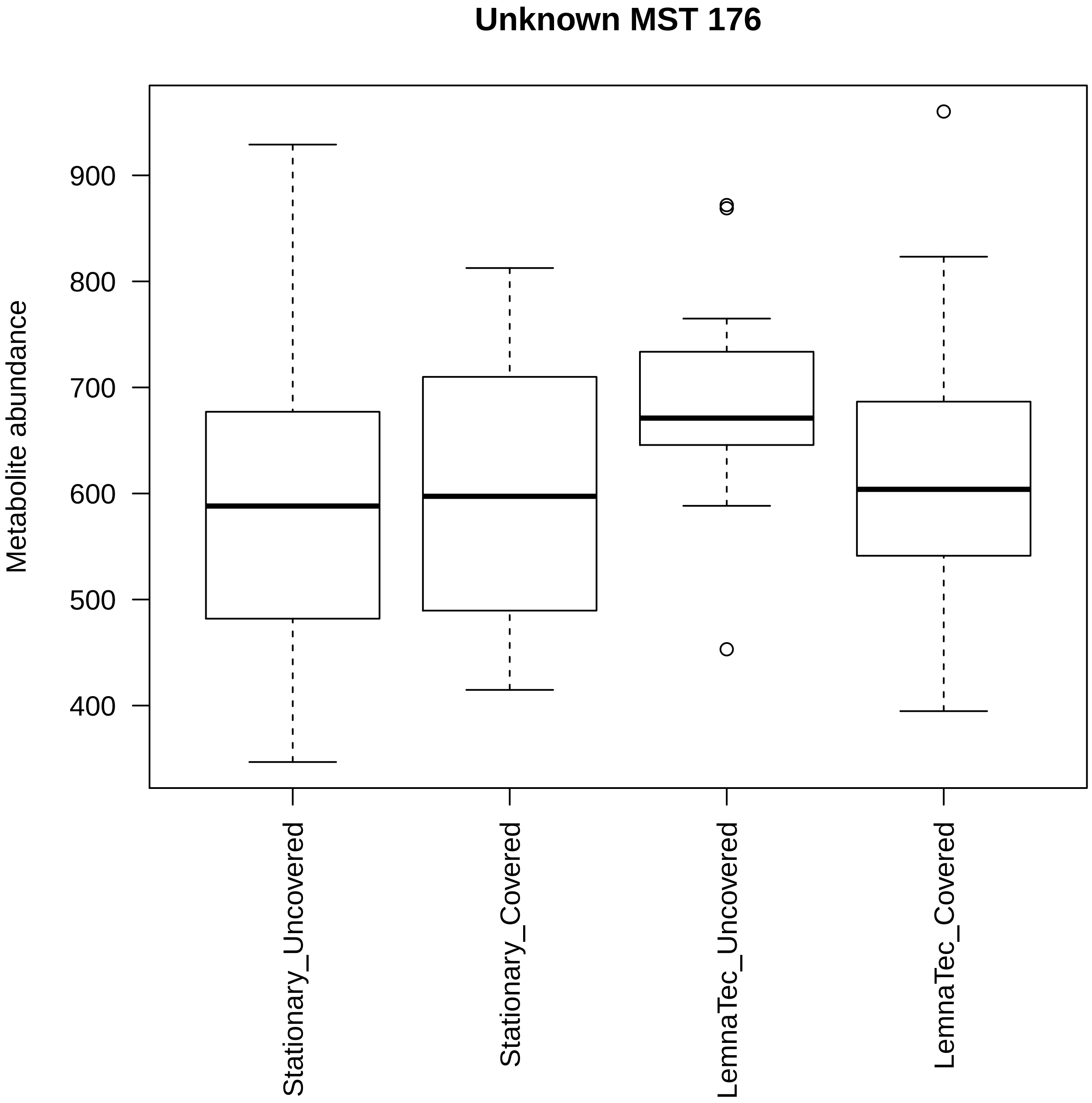
Unknown MST 176
Metabolite abundance
900
800
700
600
500
400
Stationary_Uncovered
Stationary_Covered
LemnaTec_Uncovered
LemnaTec_Covered

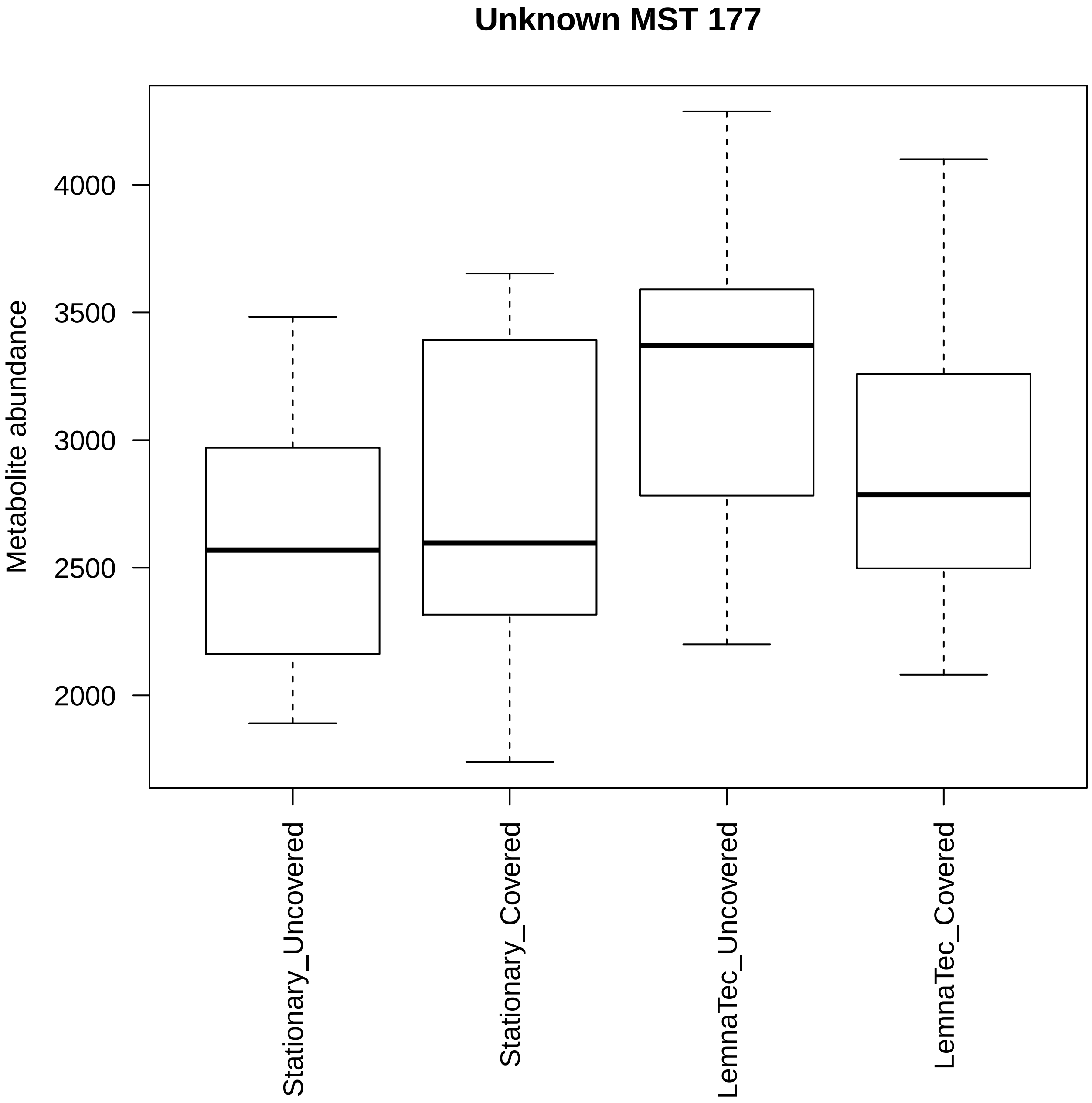
Unknown MST 177
Metabolite abundance
2000
2500
3000
3500
4000
Stationary_Uncovered
Stationary_Covered
LemnaTec_Uncovered
LemnaTec_Covered

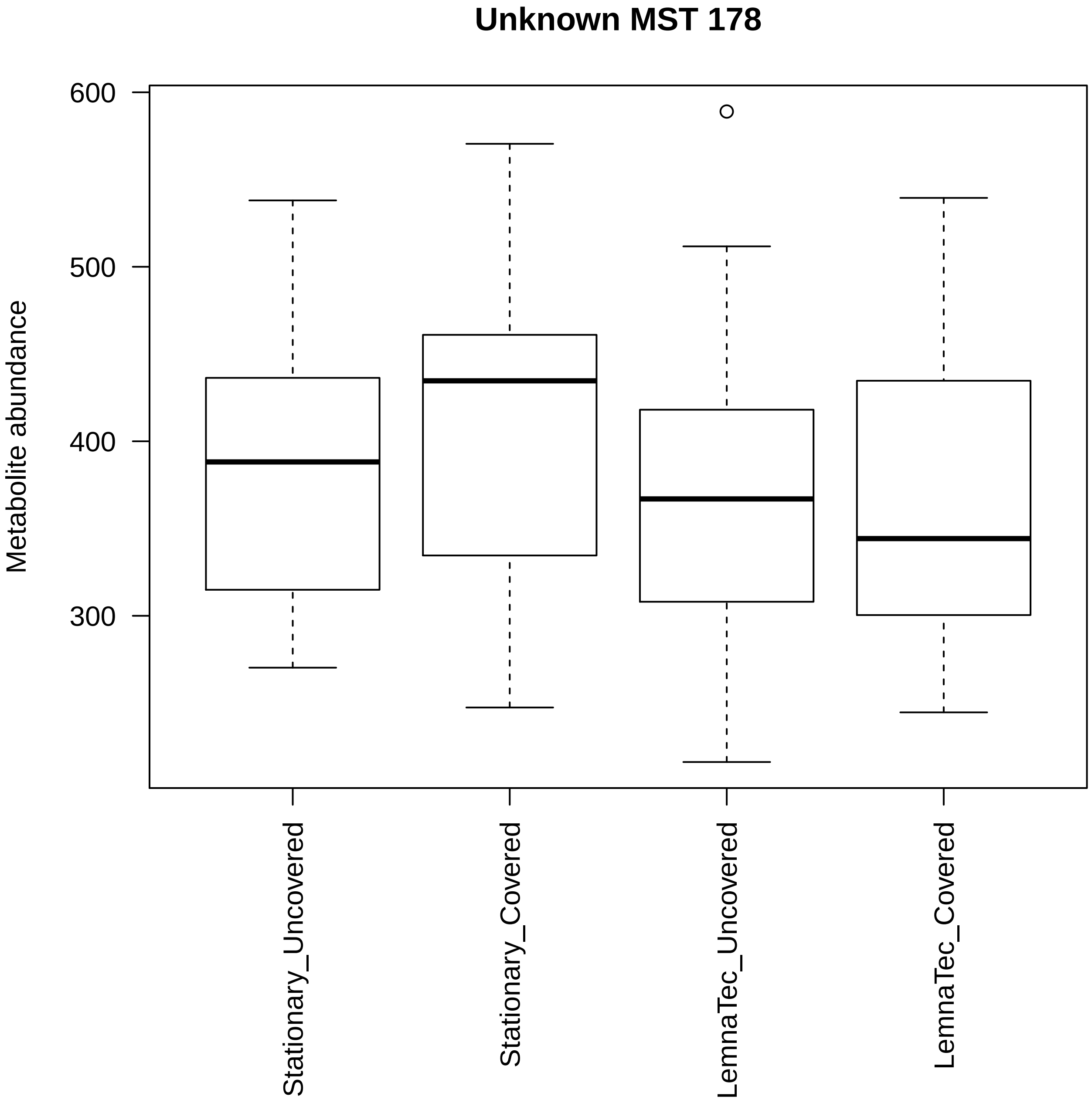

Unknown MST 178
Metabolite abundance
600
500
400
300
Stationary_Uncovered
Stationary_Covered
LemnaTec_Uncovered
LemnaTec_Covered

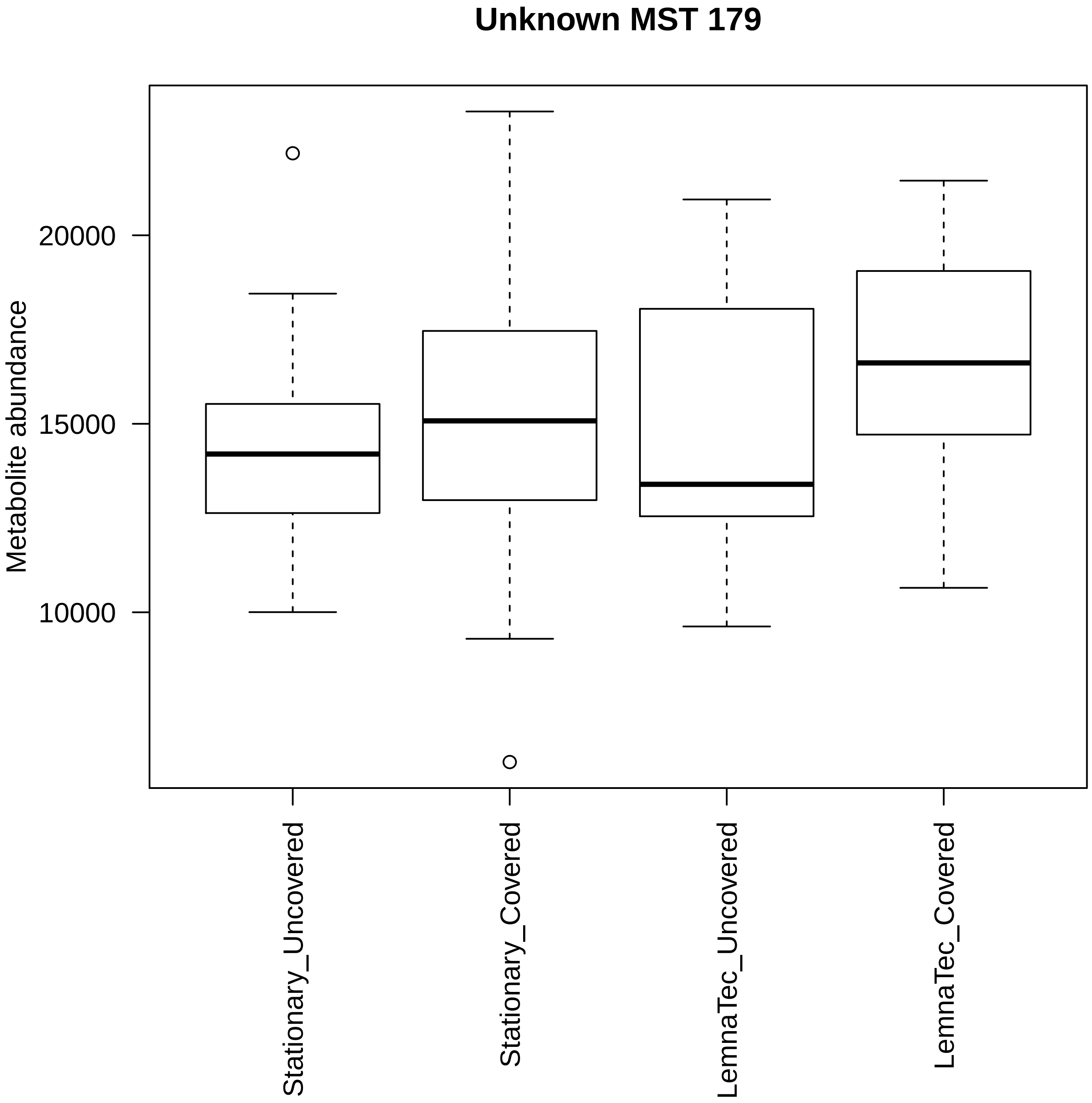
Unknown MST 179
Metabolite abundance
20000
15000
10000
Stationary_Uncovered
Stationary_Covered
LemnaTec_Uncovered
LemnaTec_Covered

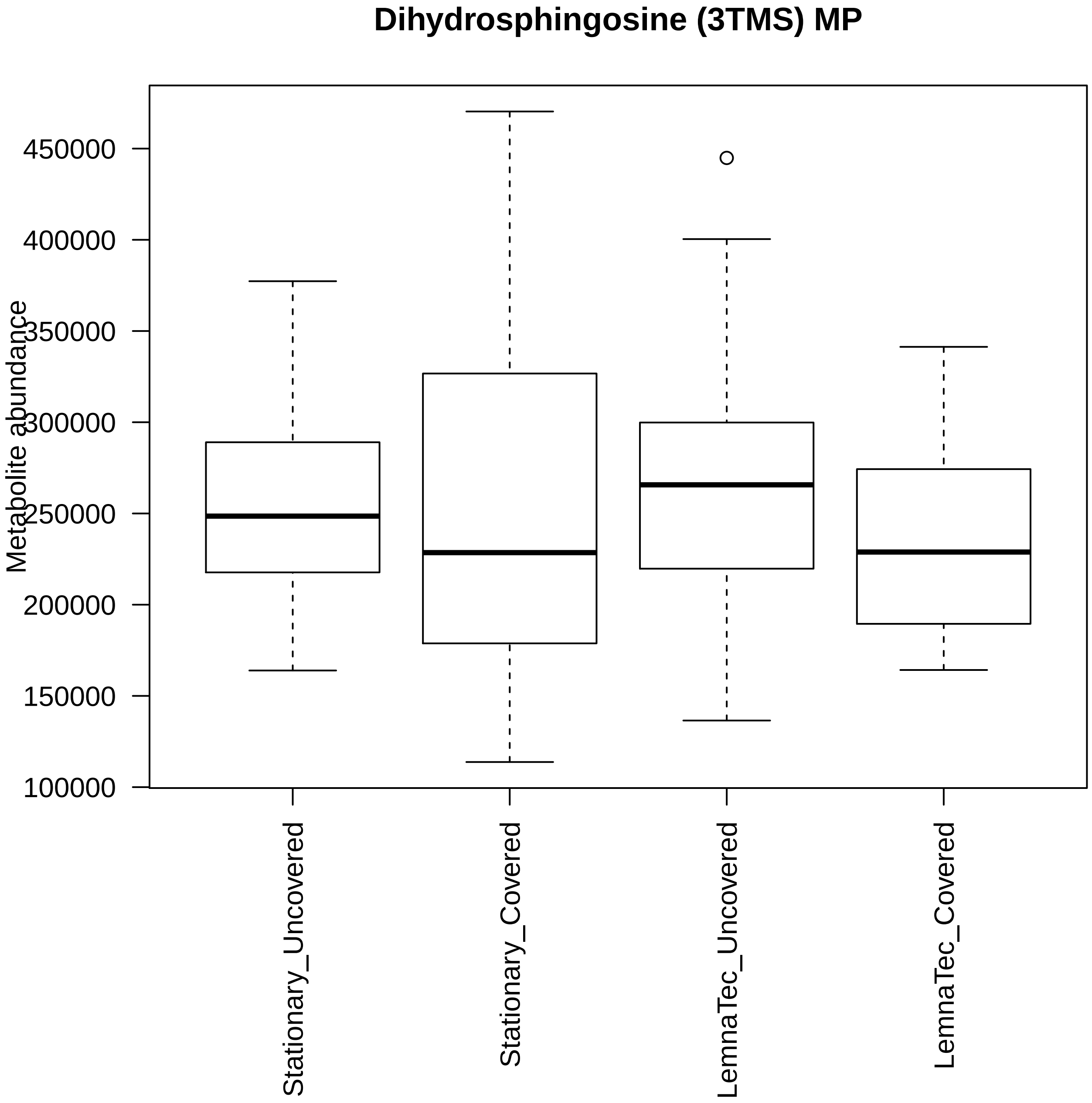

Dihydrosphingosine (3TMS) MP
Metabolite abundance
450000
400000
350000
300000
250000
200000
150000
100000
Stationary_Uncovered
Stationary_Covered
LemnaTec_Uncovered
LemnaTec_Covered

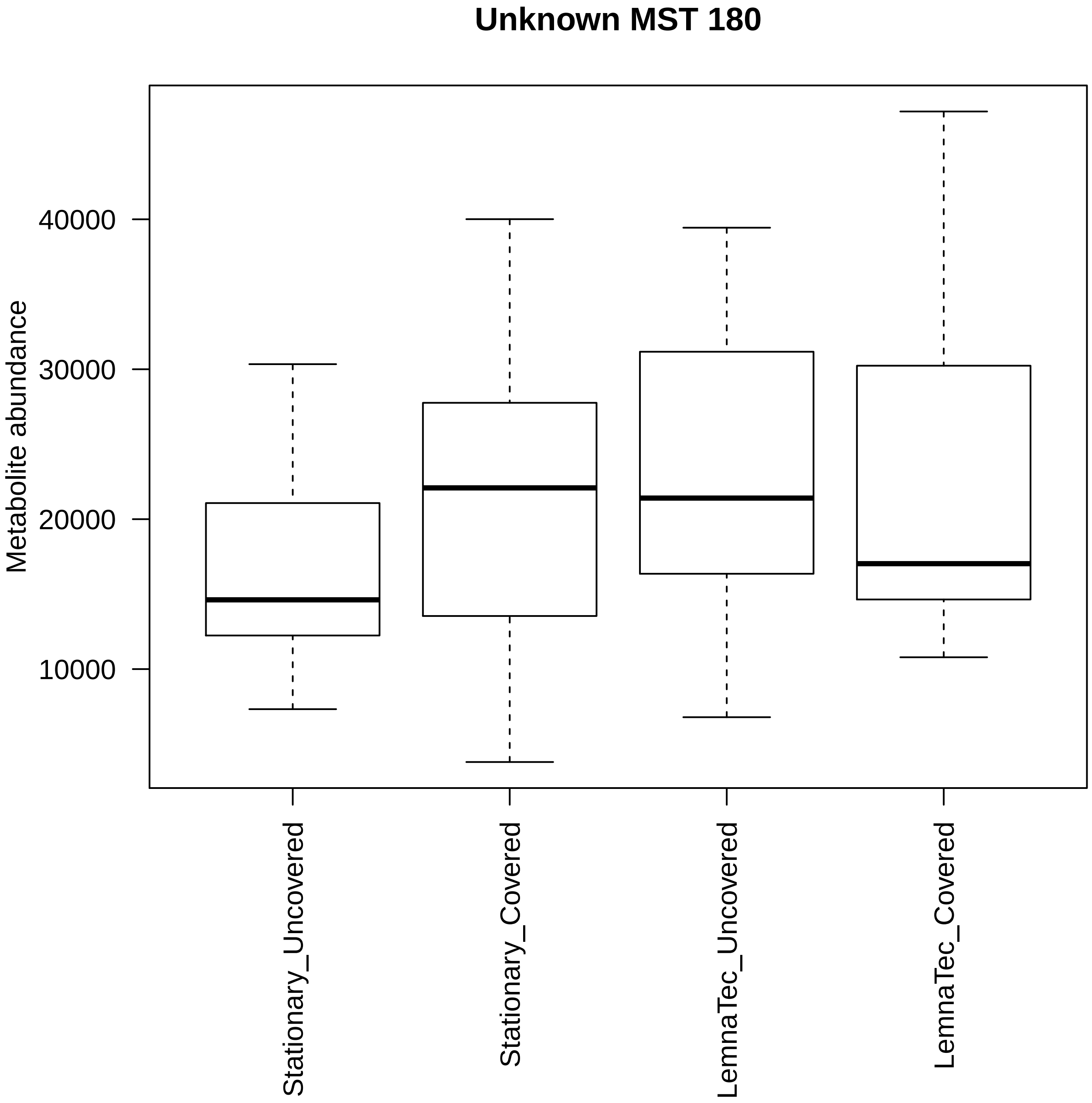

Unknown MST 180
Metabolite abundance
10000
20000
30000
40000
Stationary_Uncovered
Stationary_Covered
LemnaTec_Uncovered
LemnaTec_Covered

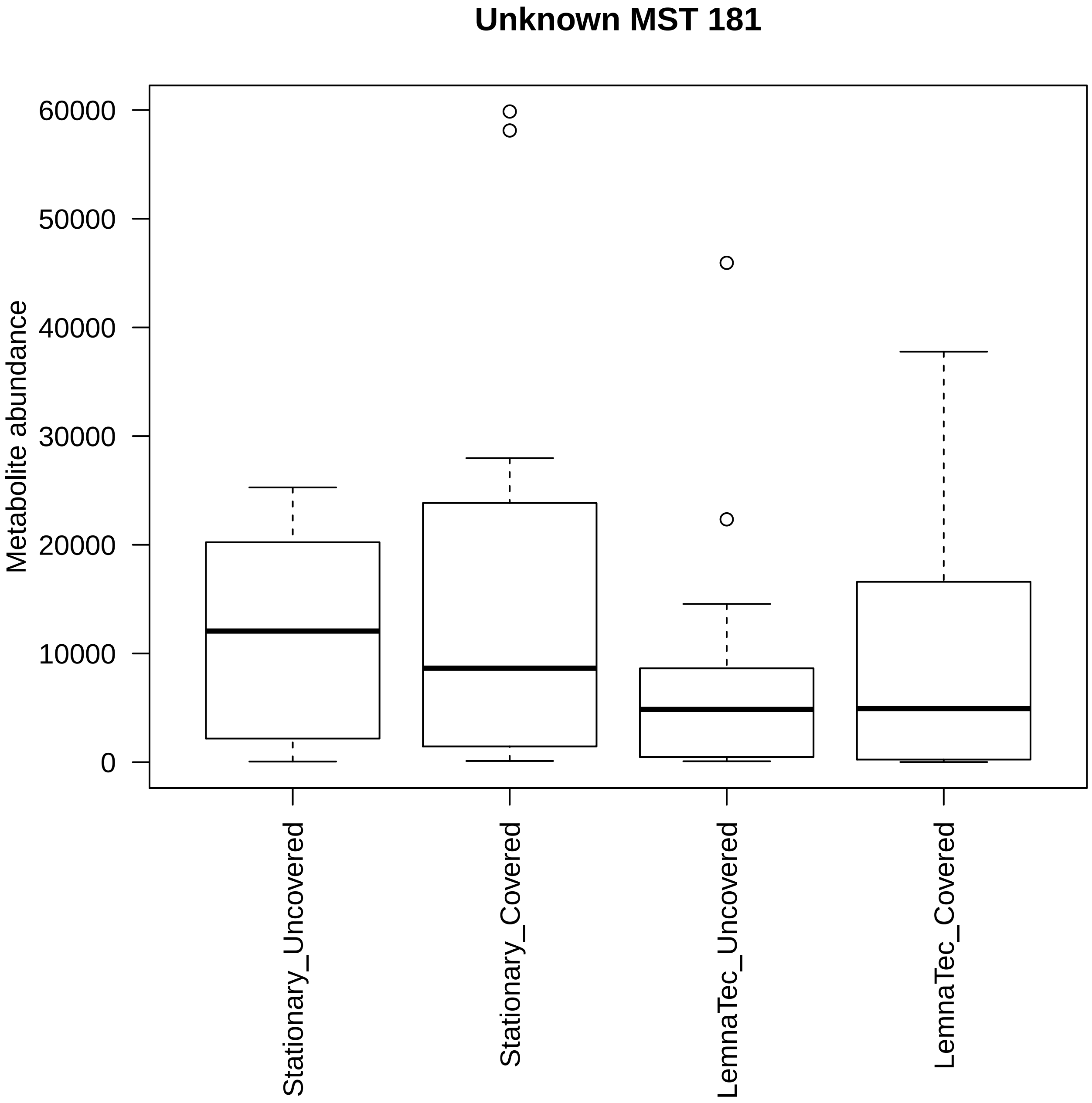
Unknown MST 181
Metabolite abundance
60000
50000
40000
30000
20000
10000
0
Stationary_Uncovered
Stationary_Covered
LemnaTec_Uncovered
LemnaTec_Covered

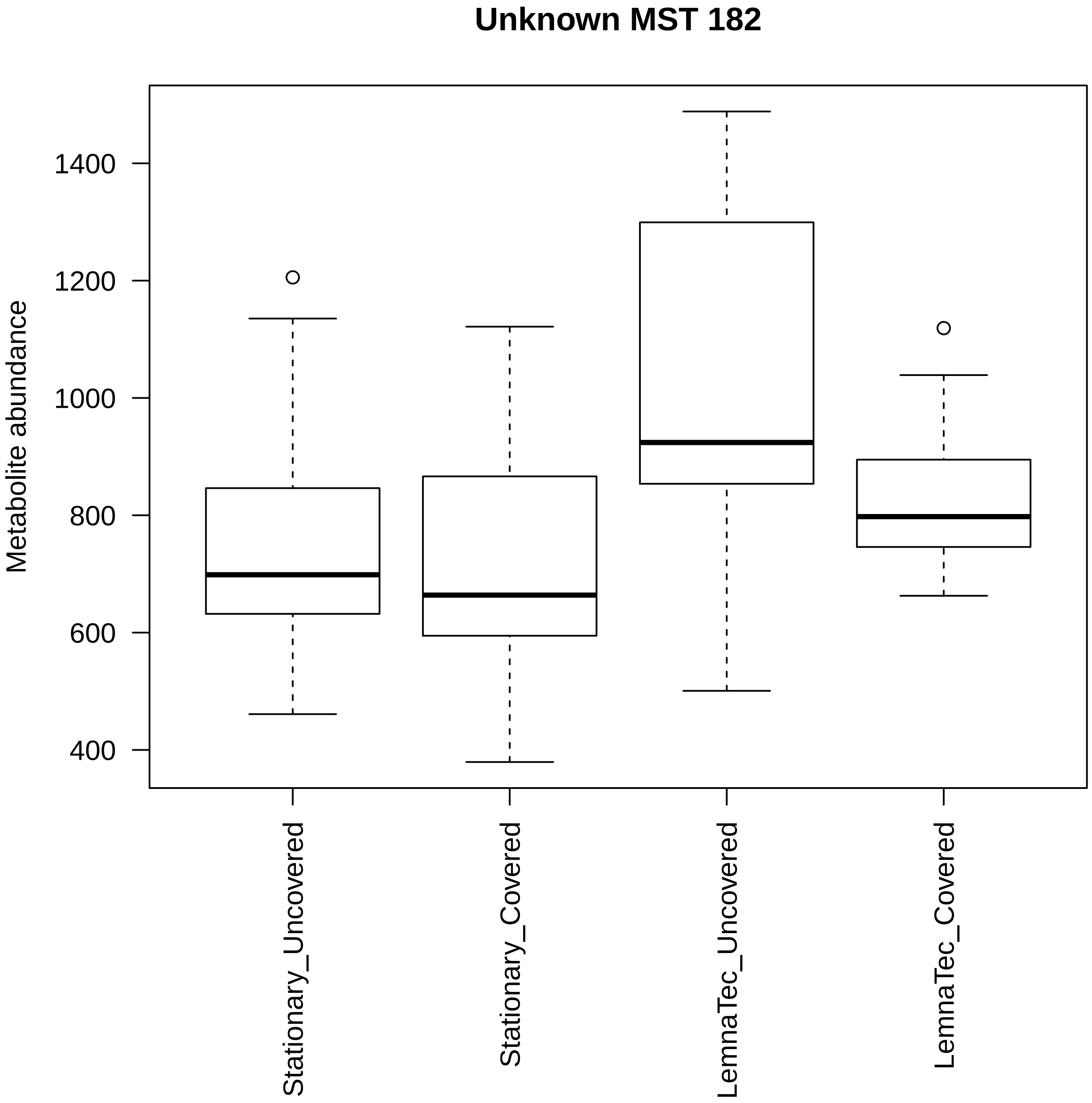
Unknown MST 182
Metabolite abundance
1400
1200
1000
800
600
400
Stationary_Uncovered
Stationary_Covered
LemnaTec_Uncovered
LemnaTec_Covered

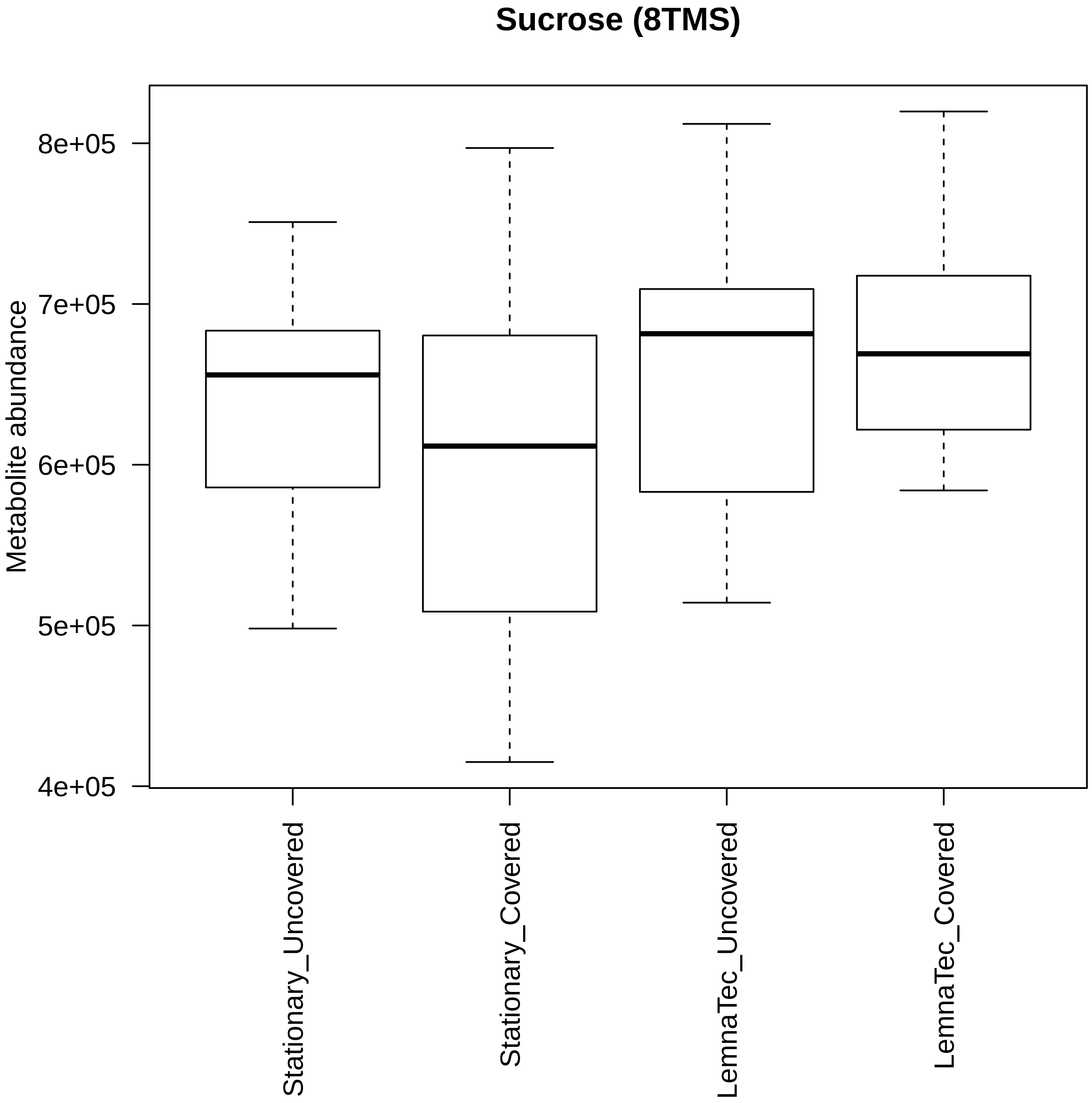
Sucrose (8TMS)
Metabolite abundance
8e+05
7e+05
6e+05
5e+05
4e+05
Stationary_Uncovered
Stationary_Covered
LemnaTec_Uncovered
LemnaTec_Covered

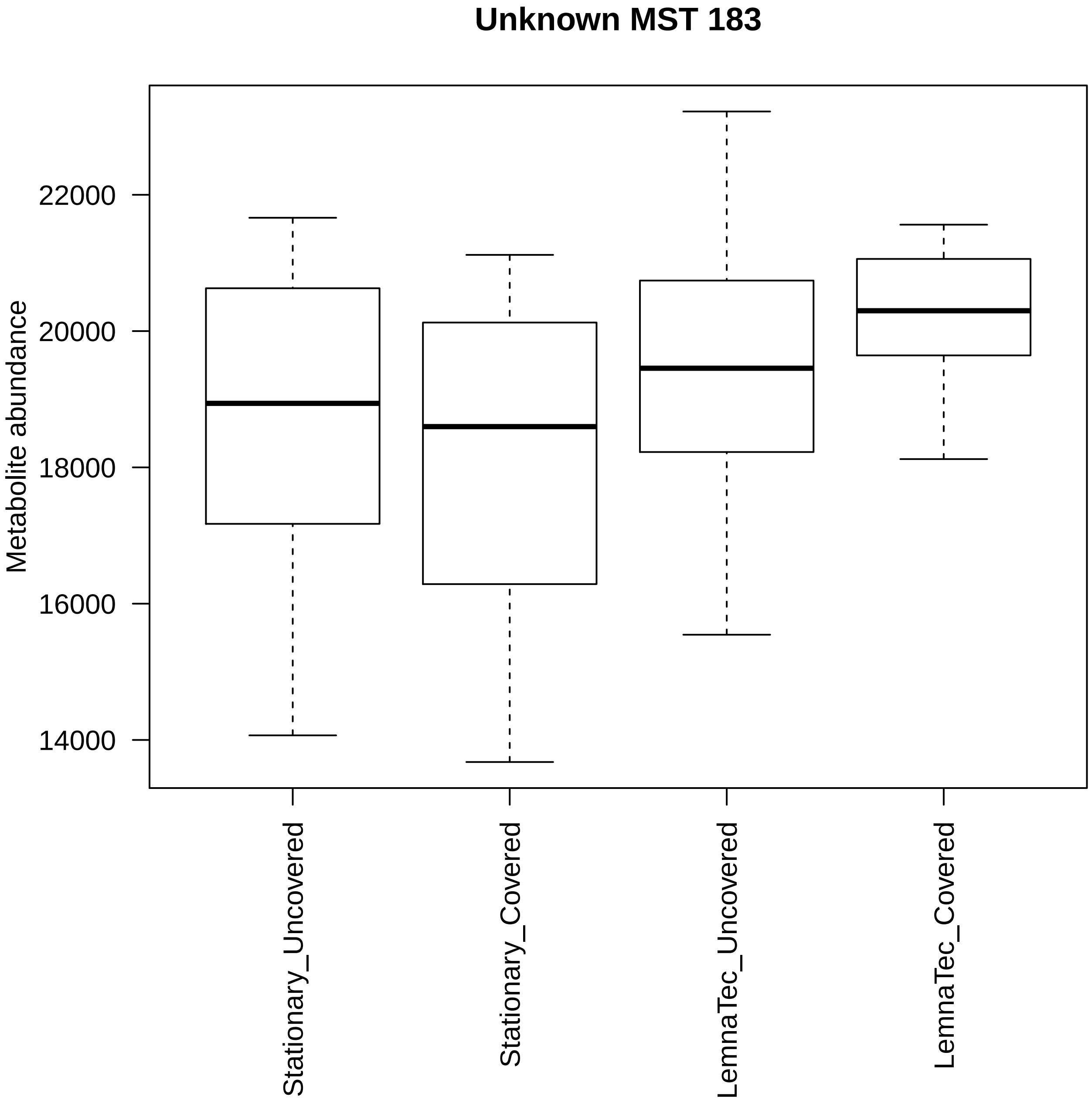
Unknown MST 183
Metabolite abundance
22000
20000
18000
16000
14000
Stationary_Uncovered
Stationary_Covered
LemnaTec_Uncovered
LemnaTec_Covered

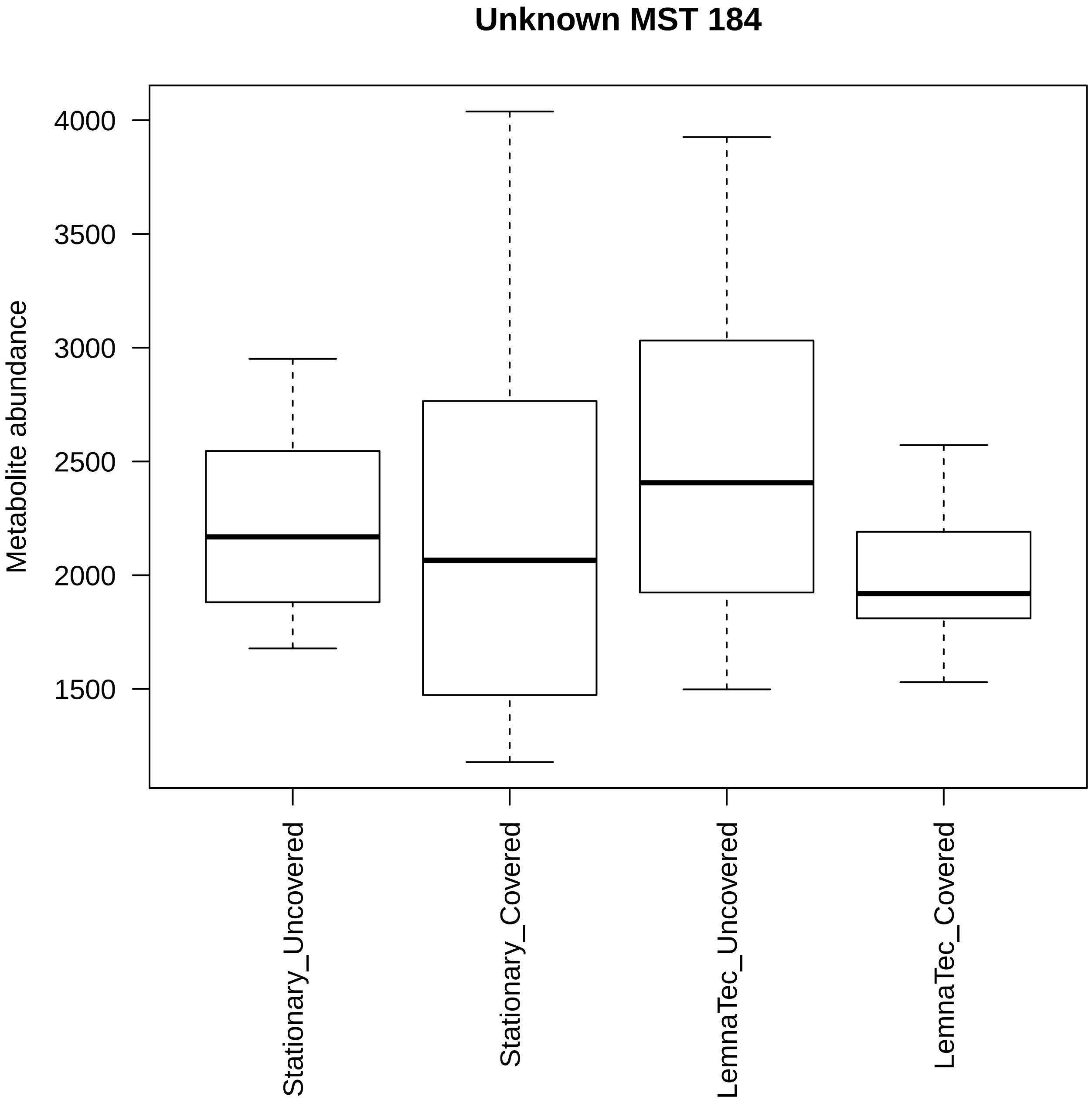
Unknown MST 184
Metabolite abundance
4000
3500
3000
2500
2000
1500
Stationary_Uncovered
Stationary_Covered
LemnaTec_Uncovered
LemnaTec_Covered

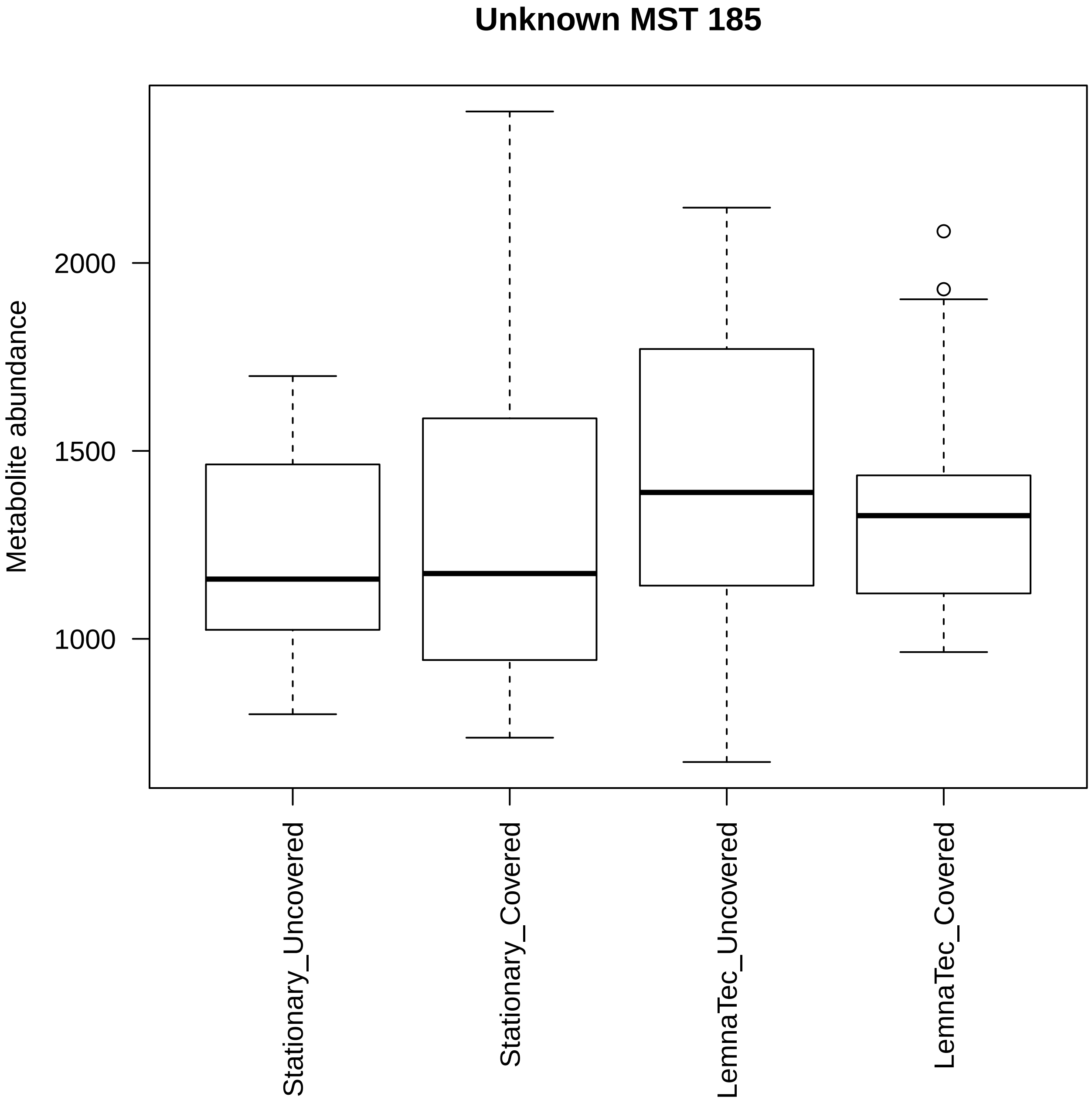
Unknown MST 185
Metabolite abundance
2000
1500
1000
Stationary_Uncovered
Stationary_Covered
LemnaTec_Uncovered
LemnaTec_Covered

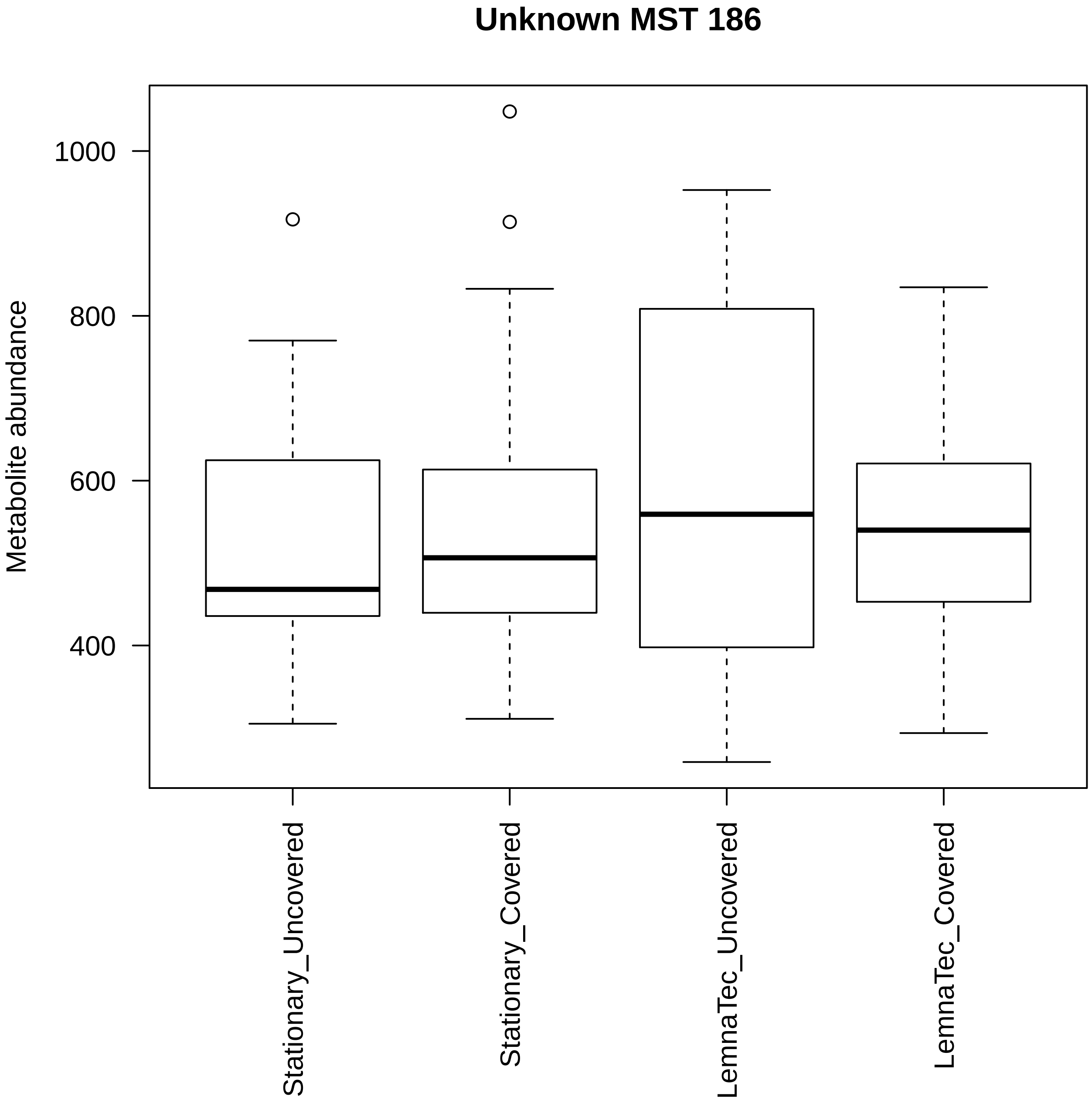
Unknown MST 186
Metabolite abundance
1000
800
600
400
Stationary_Uncovered
Stationary_Covered
LemnaTec_Uncovered
LemnaTec_Covered

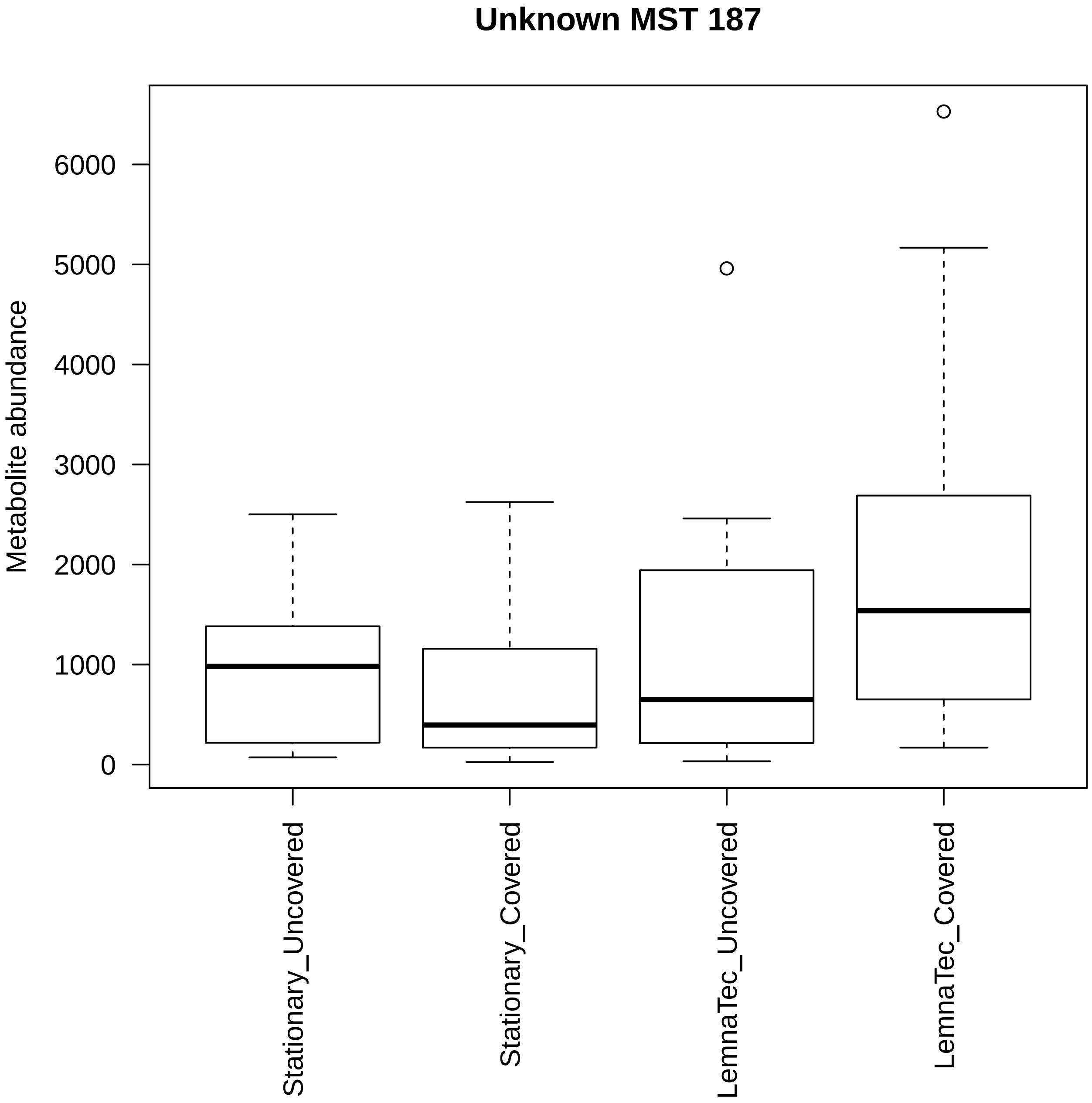

Unknown MST 187
Metabolite abundance
0
1000
2000
3000
4000
5000
6000
Stationary_Uncovered
Stationary_Covered
LemnaTec_Uncovered
LemnaTec_Covered

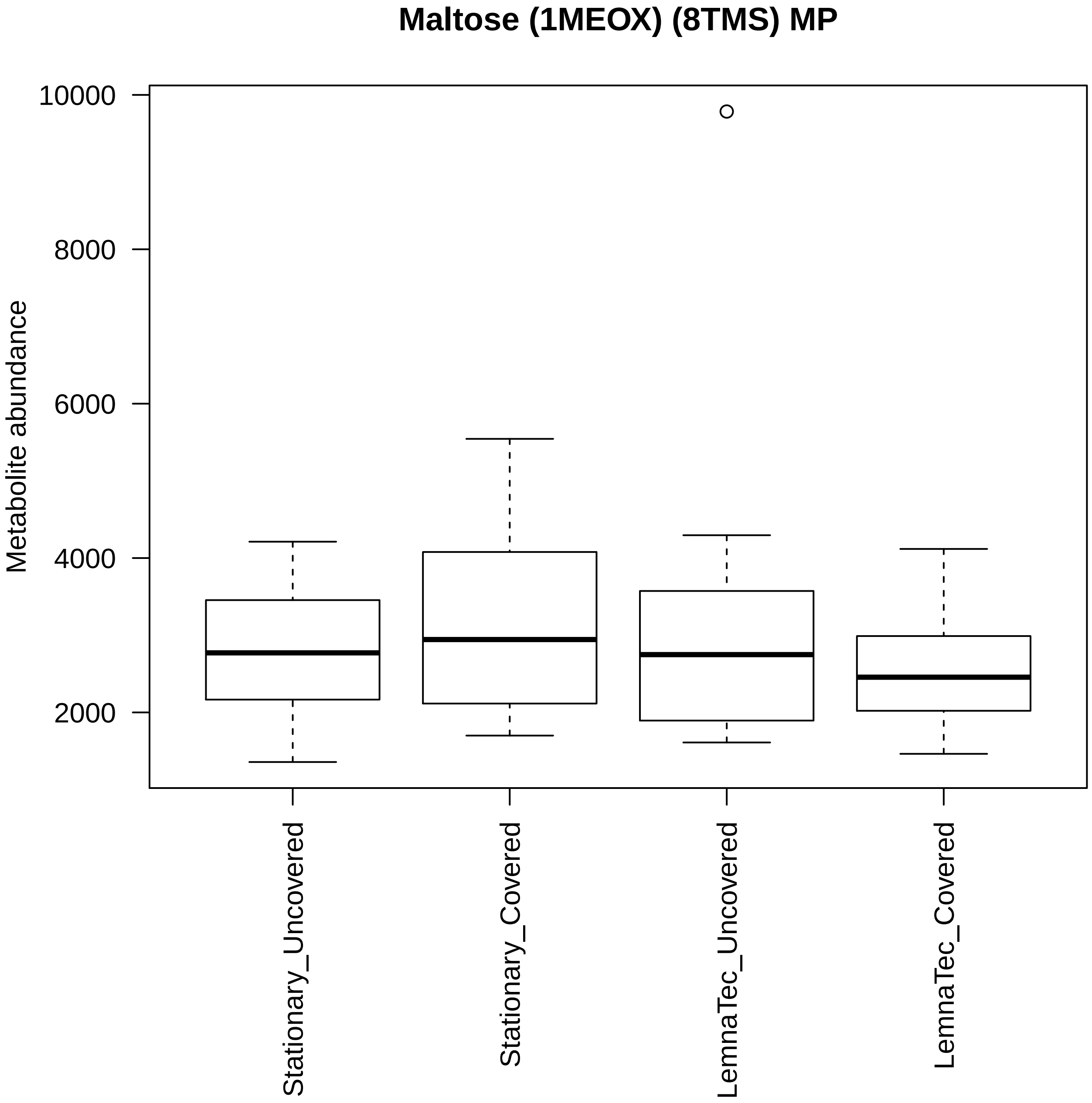
Maltose (1MEOX) (8TMS) MP
Metabolite abundance
10000
8000
6000
4000
2000
Stationary_Uncovered
Stationary_Covered
LemnaTec_Uncovered
LemnaTec_Covered

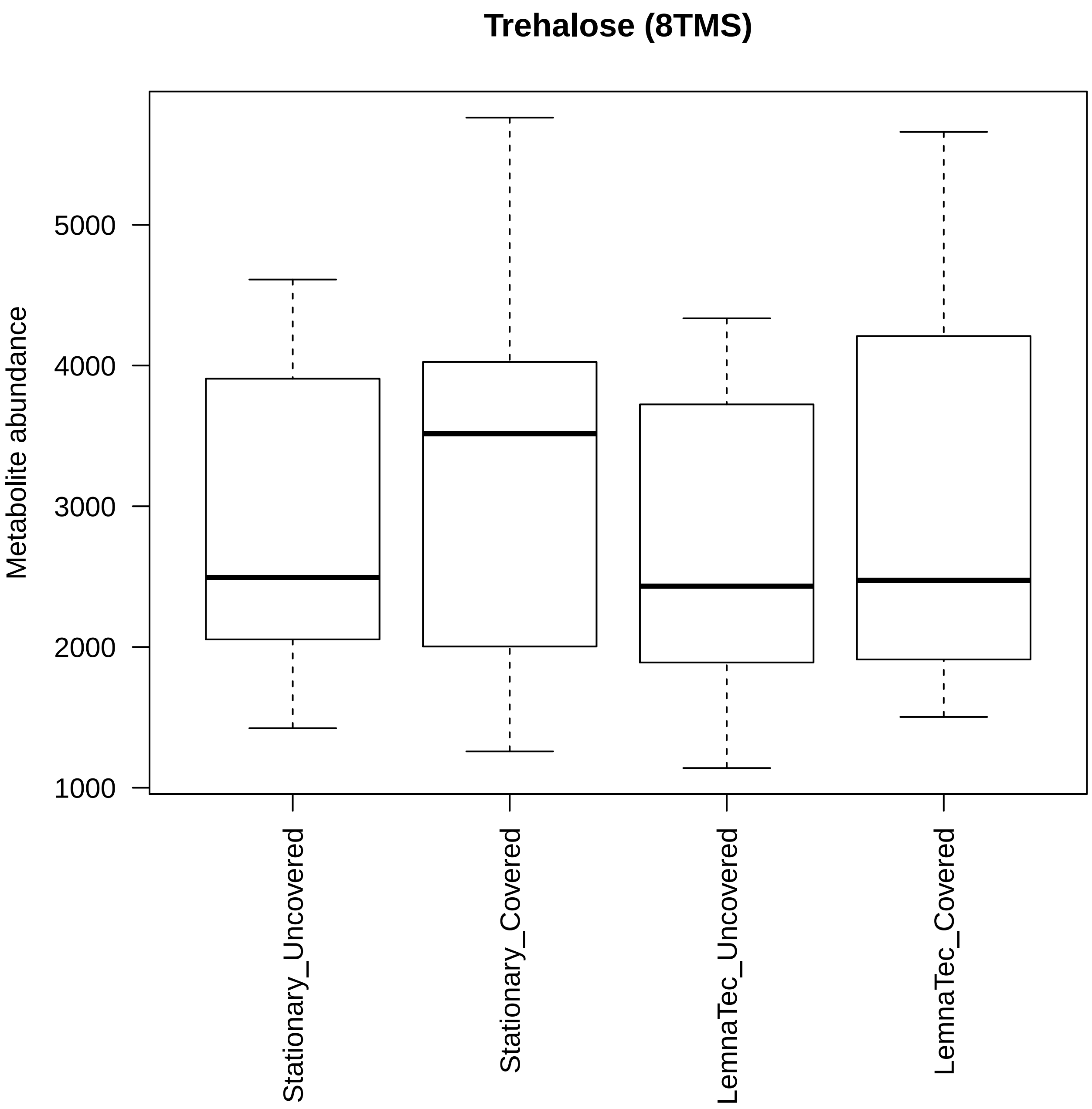
Trehalose (8TMS)
Metabolite abundance
5000
4000
3000
2000
1000
Stationary_Uncovered
Stationary_Covered
LemnaTec_Uncovered
LemnaTec_Covered

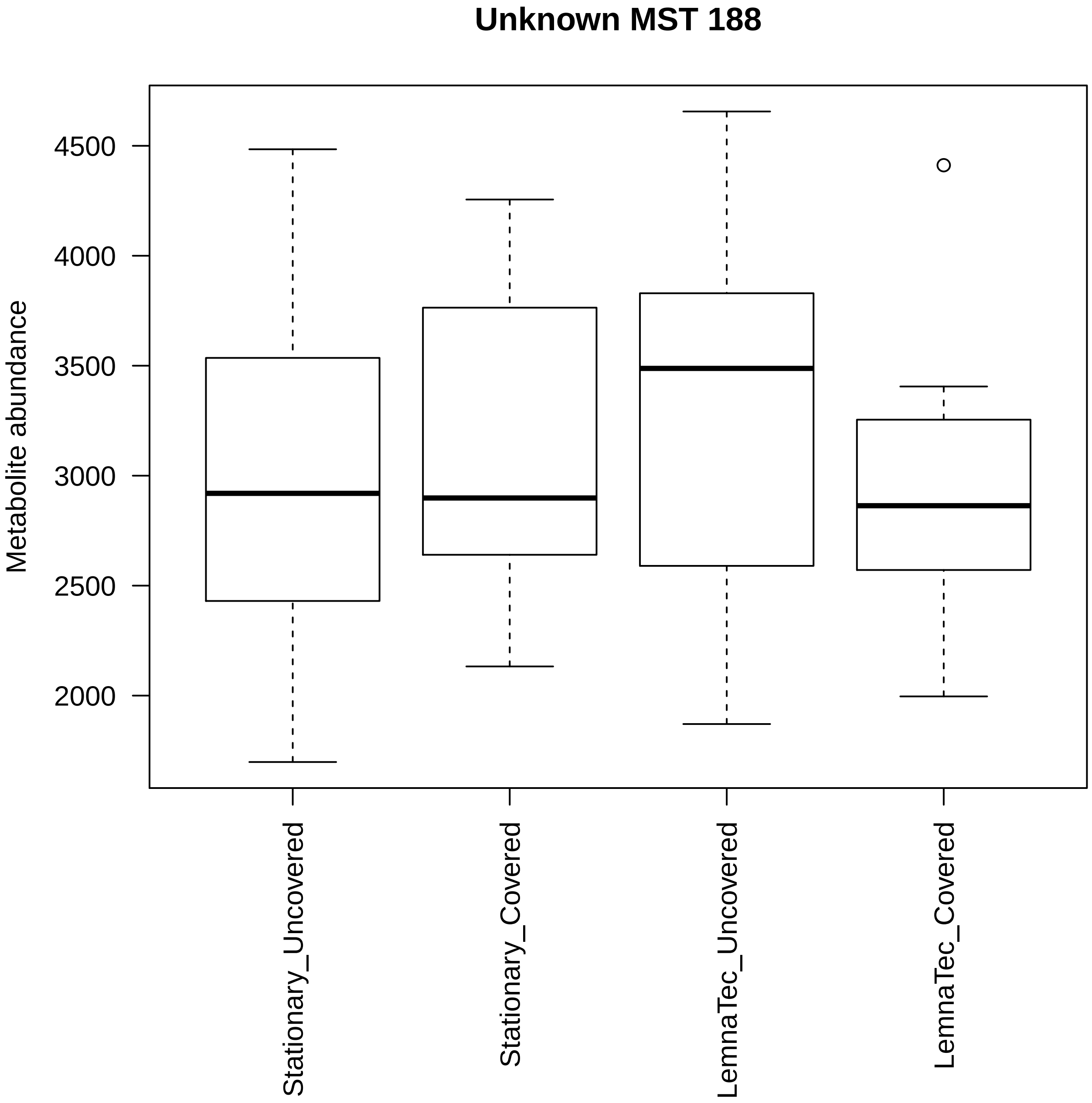

Unknown MST 188
Metabolite abundance
4500
4000
3500
3000
2500
2000
Stationary_Uncovered
Stationary_Covered
LemnaTec_Uncovered
LemnaTec_Covered

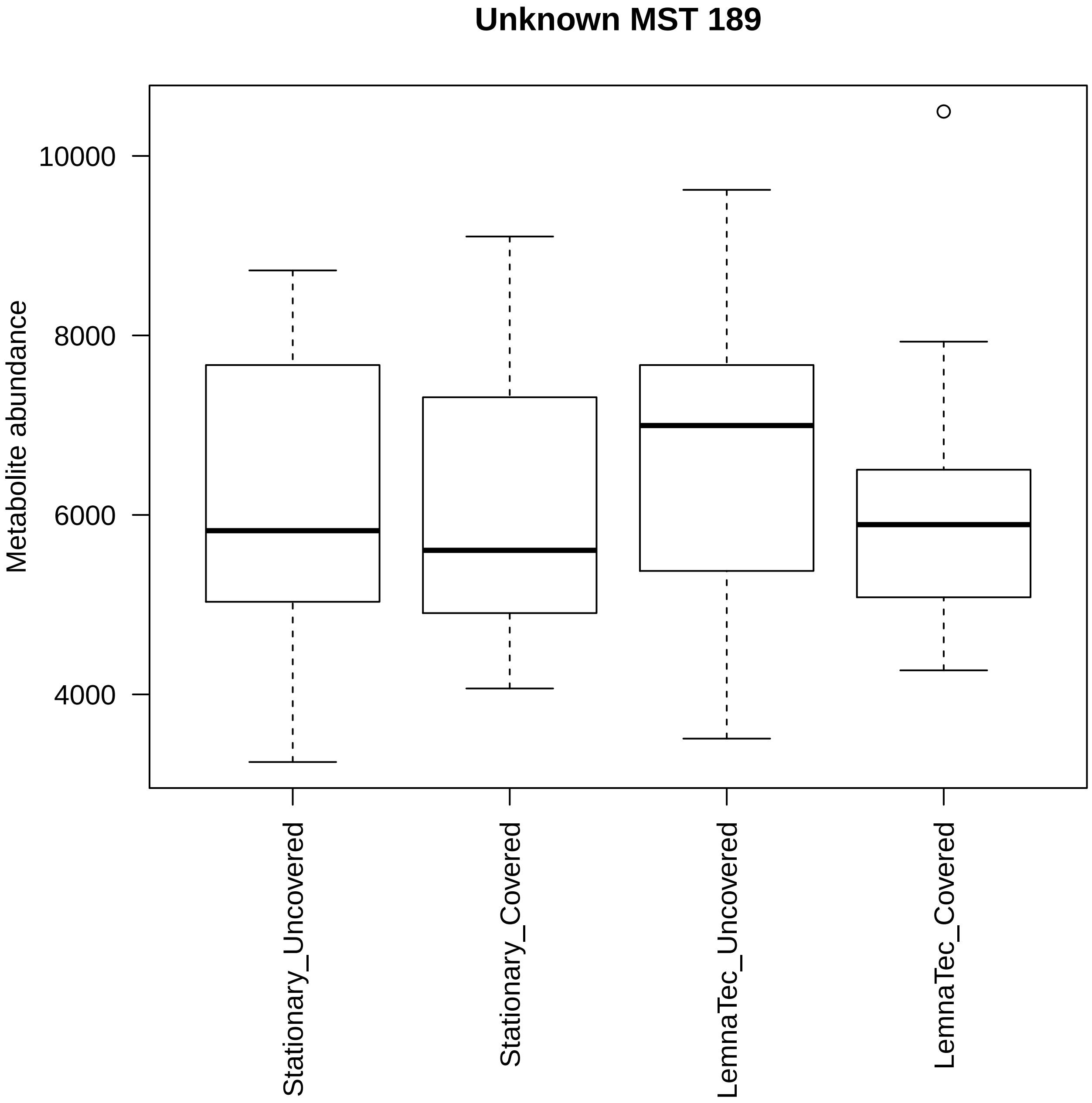

Unknown MST 189
Metabolite abundance
10000
8000
6000
4000
Stationary_Uncovered
Stationary_Covered
LemnaTec_Uncovered
LemnaTec_Covered

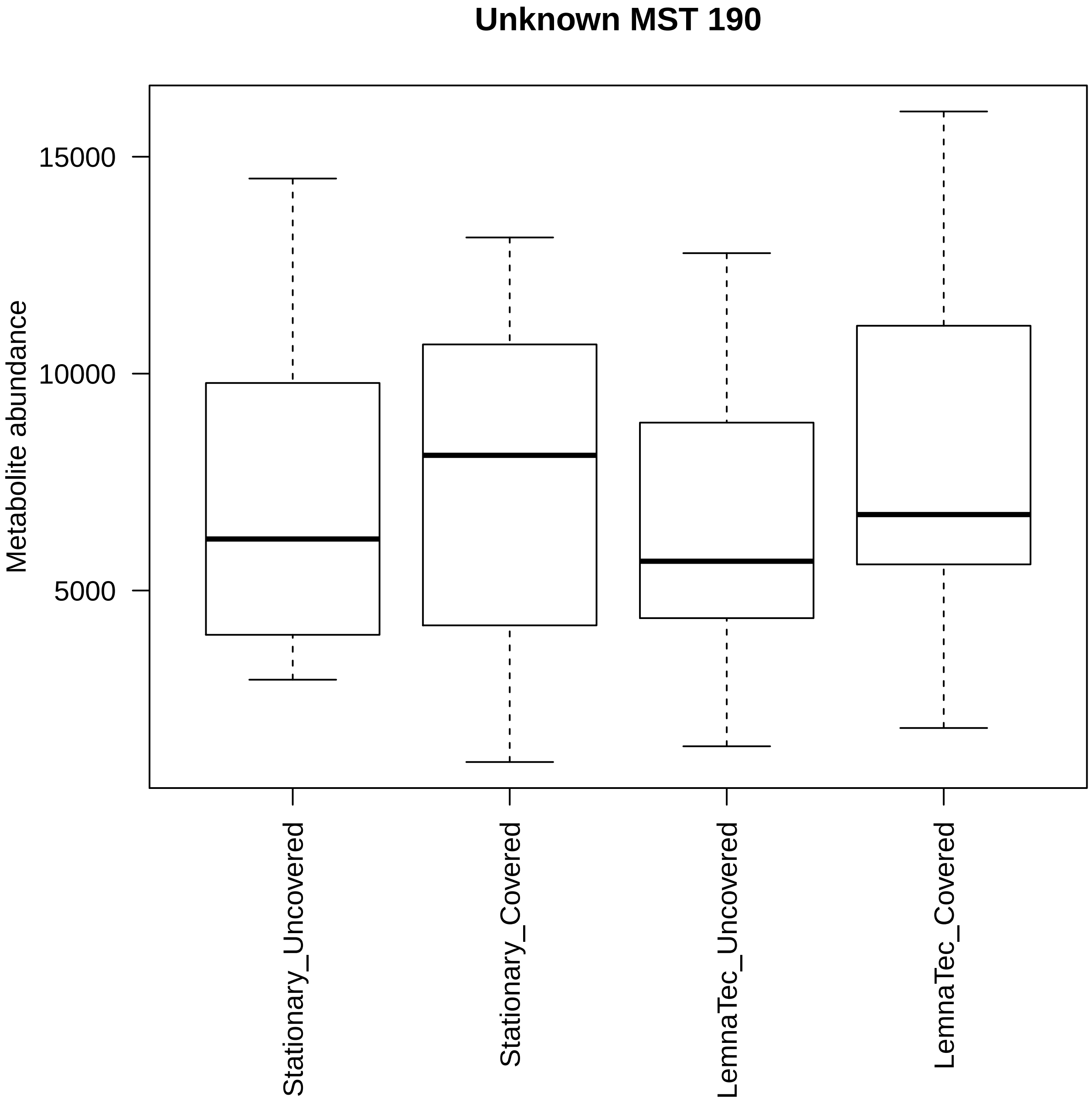
Unknown MST 190
Metabolite abundance
15000
10000
5000
Stationary_Uncovered
Stationary_Covered
LemnaTec_Uncovered
LemnaTec_Covered

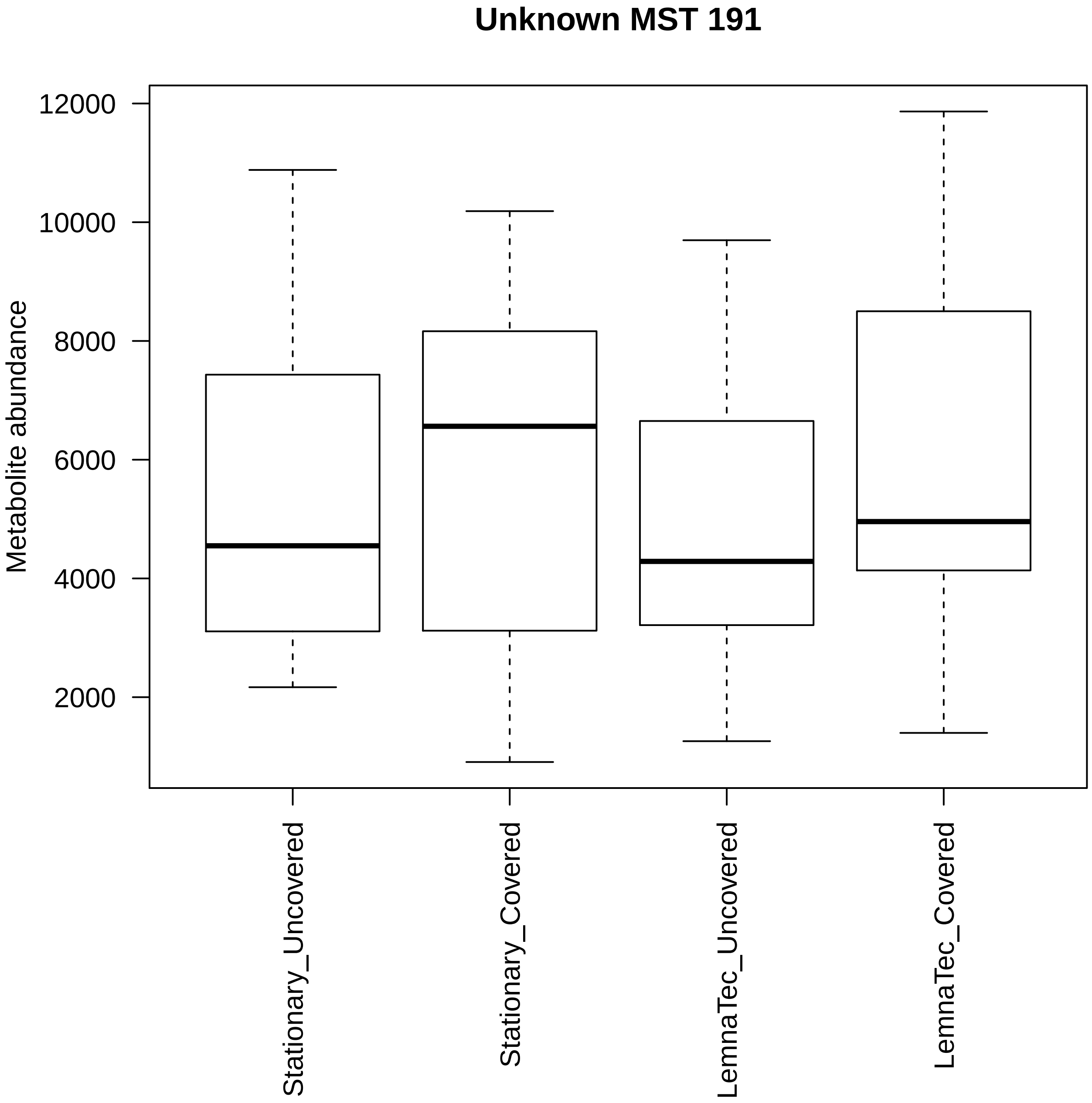
Unknown MST 191
Metabolite abundance
12000
10000
8000
6000
4000
2000
Stationary_Uncovered
Stationary_Covered
LemnaTec_Uncovered
LemnaTec_Covered

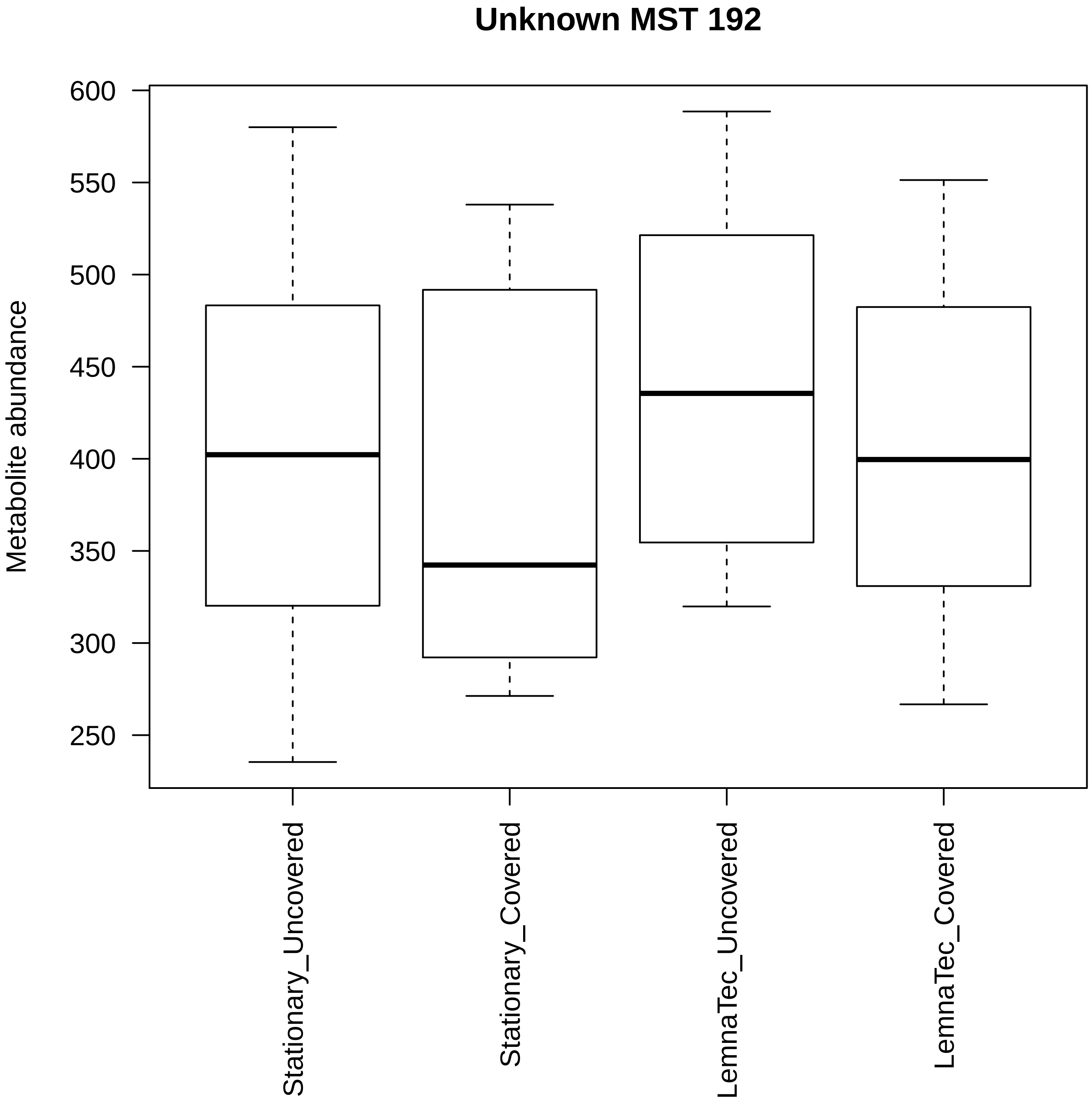
Unknown MST 192
Metabolite abundance
600
550
500
450
400
350
300
250
Stationary_Uncovered
Stationary_Covered
LemnaTec_Uncovered
LemnaTec_Covered

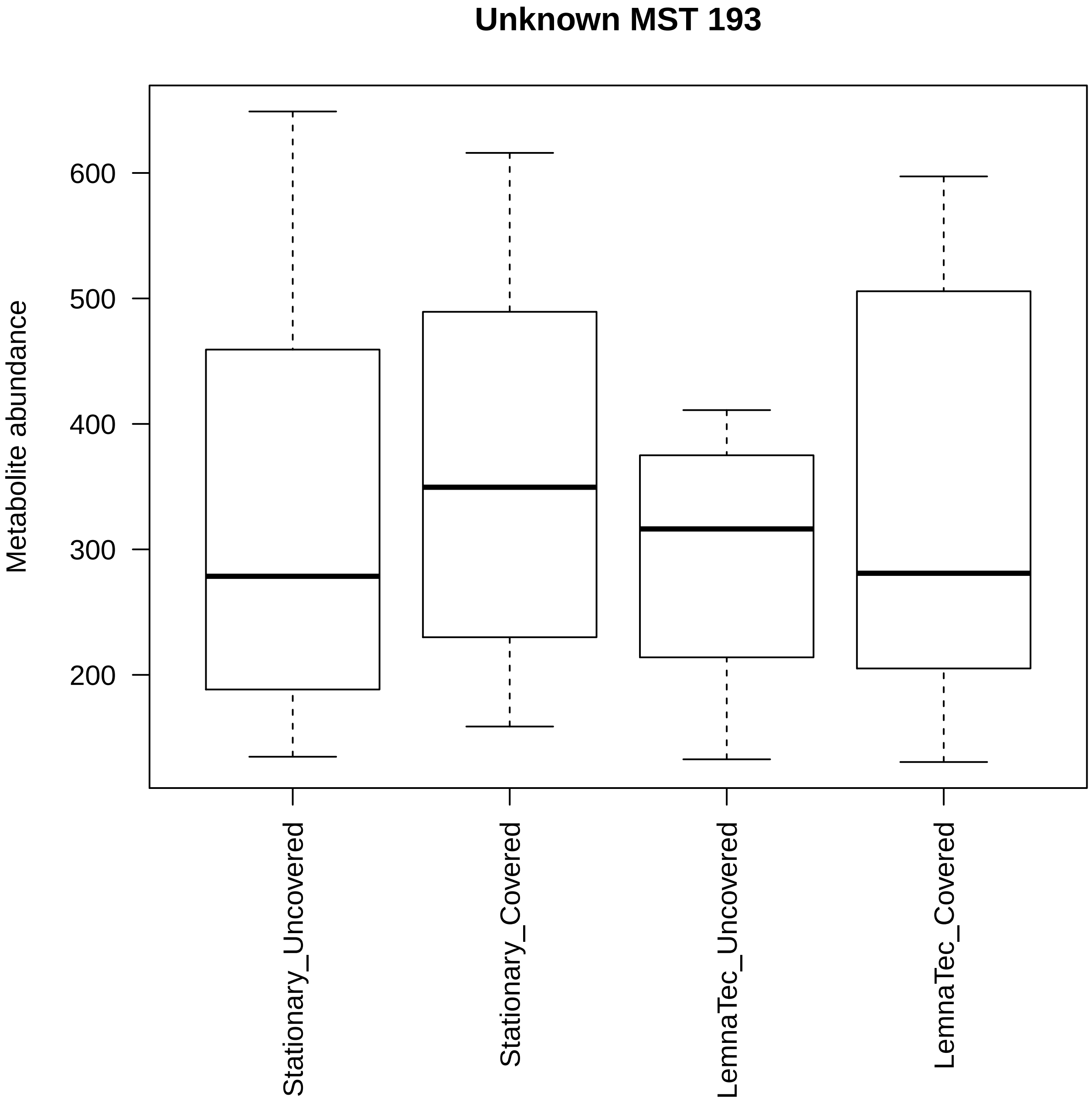

Unknown MST 193
Metabolite abundance
600
500
400
300
200
Stationary_Uncovered
Stationary_Covered
LemnaTec_Uncovered
LemnaTec_Covered

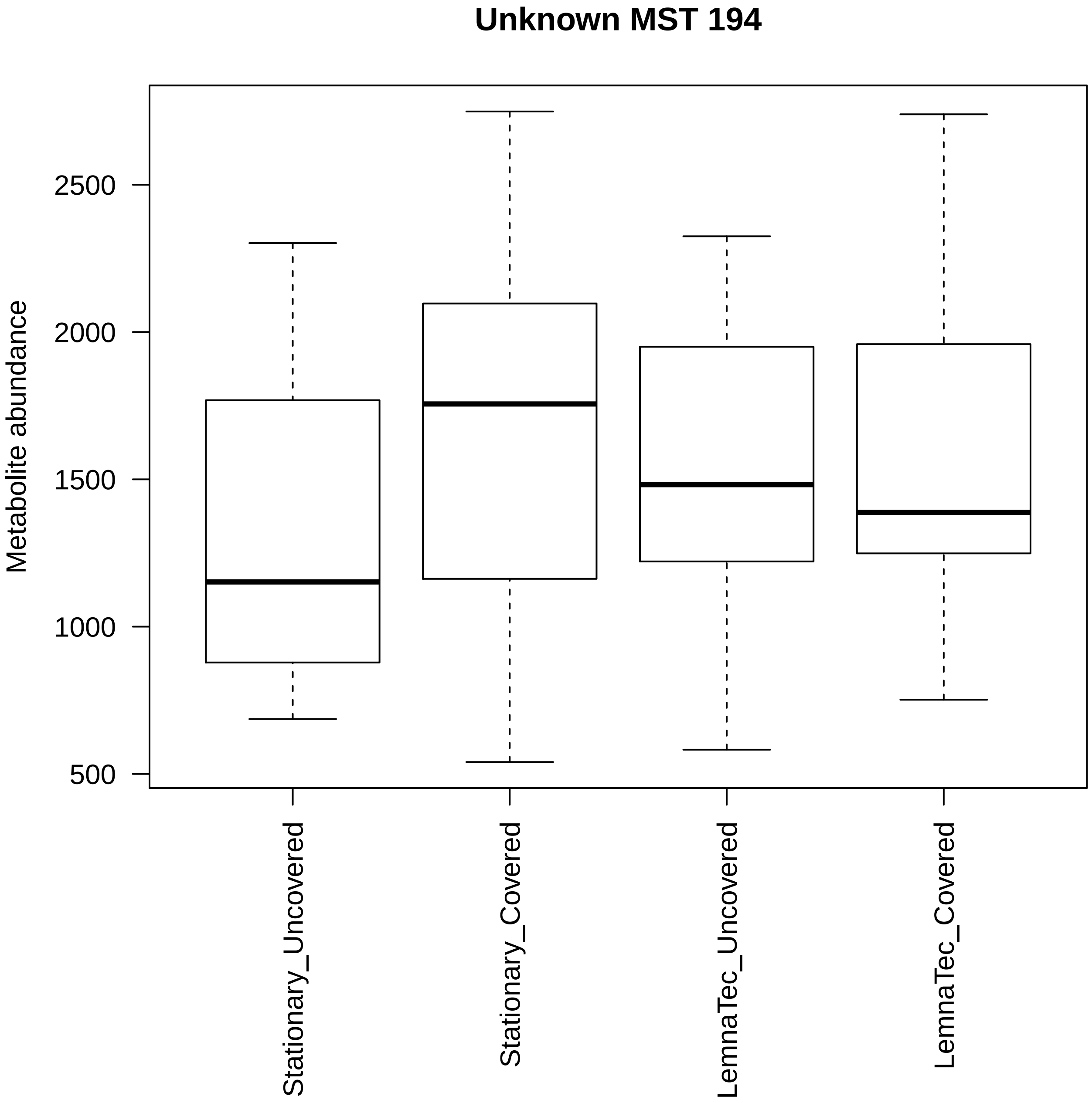
Unknown MST 194
Metabolite abundance
500
1000
1500
2000
2500
Stationary_Uncovered
Stationary_Covered
LemnaTec_Uncovered
LemnaTec_Covered

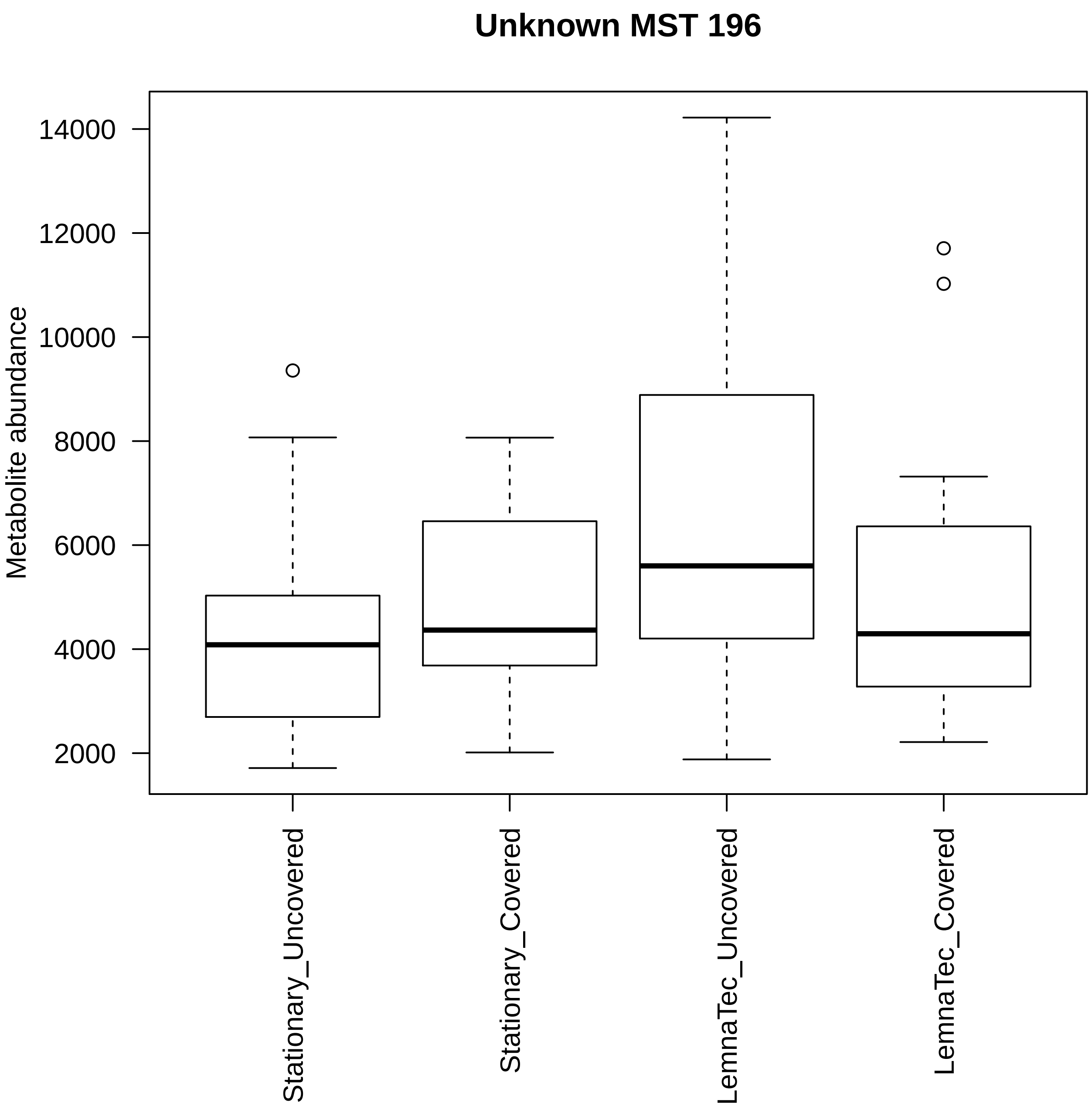

Unknown MST 196
Metabolite abundance
14000
12000
10000
8000
6000
4000
2000
Stationary_Uncovered
Stationary_Covered
LemnaTec_Uncovered
LemnaTec_Covered

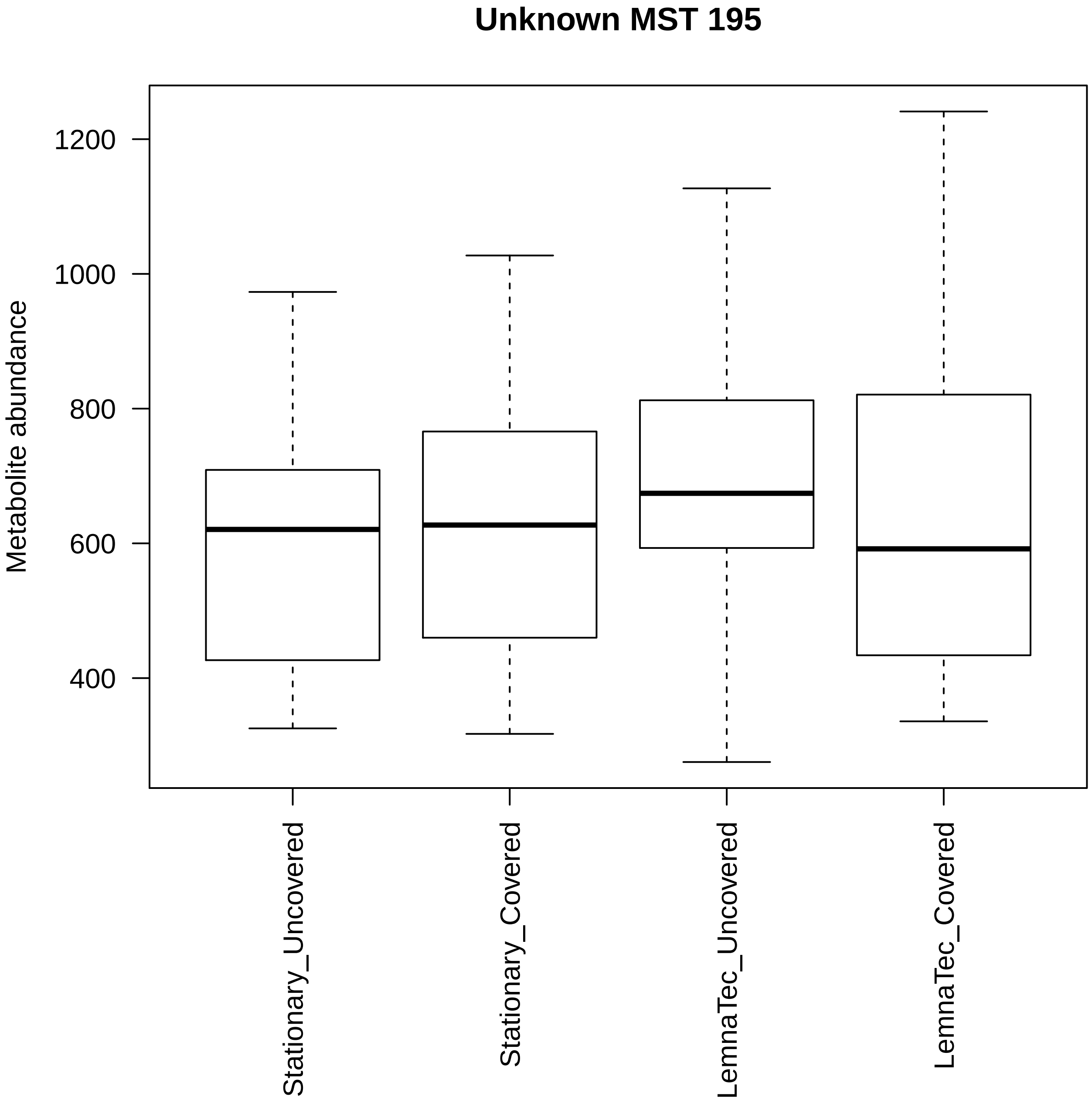
Unknown MST 195
Metabolite abundance
1200
1000
800
600
400
Stationary_Uncovered
Stationary_Covered
LemnaTec_Uncovered
LemnaTec_Covered

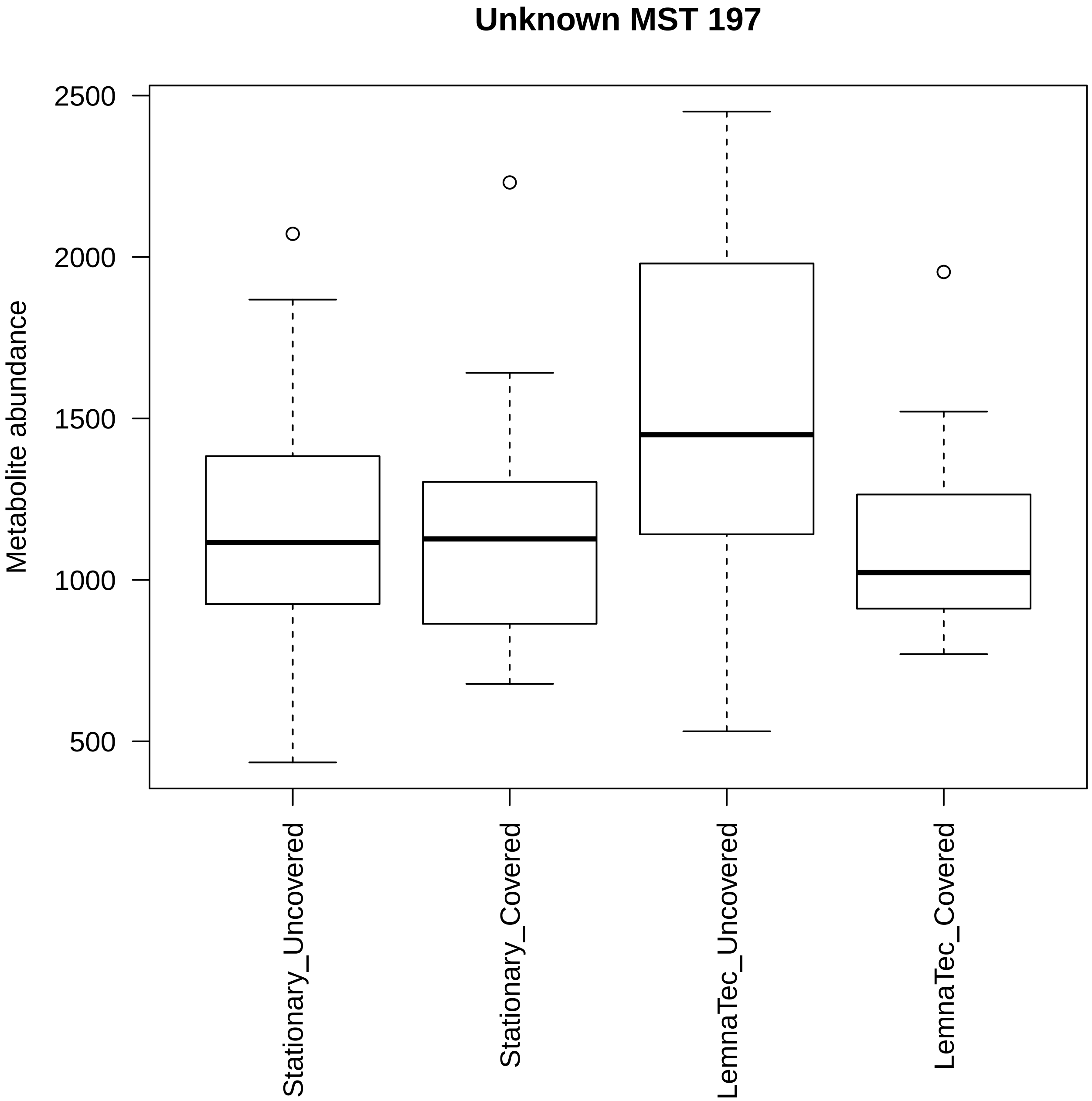

Unknown MST 197
Metabolite abundance
2500
2000
1500
1000
500
Stationary_Uncovered
Stationary_Covered
LemnaTec_Uncovered
LemnaTec_Covered

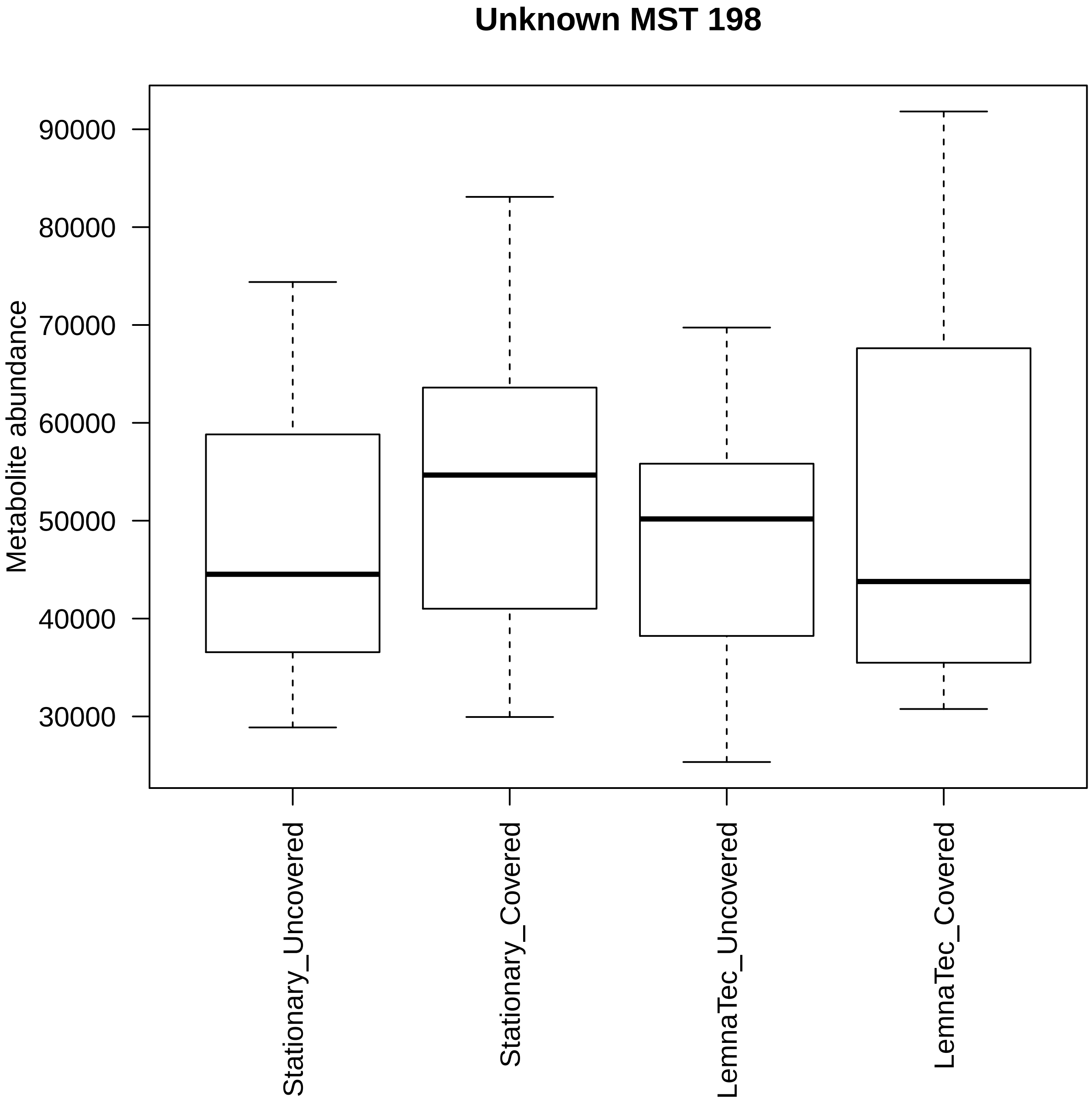
Unknown MST 198
Metabolite abundance
90000
80000
70000
60000
50000
40000
30000
Stationary_Uncovered
Stationary_Covered
LemnaTec_Uncovered
LemnaTec_Covered

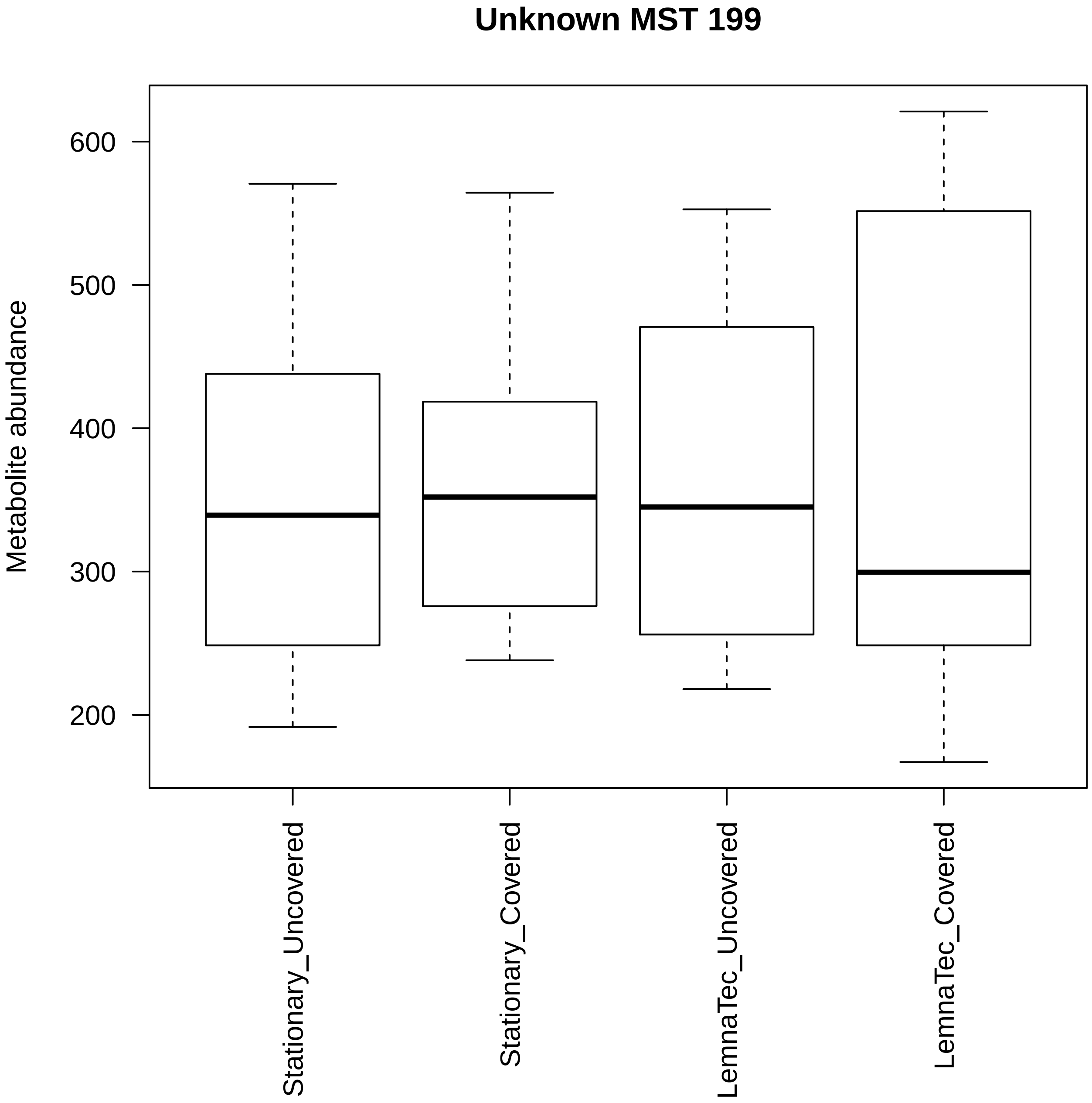
Unknown MST 199
Metabolite abundance
600
500
400
300
200
Stationary_Uncovered
Stationary_Covered
LemnaTec_Uncovered
LemnaTec_Covered

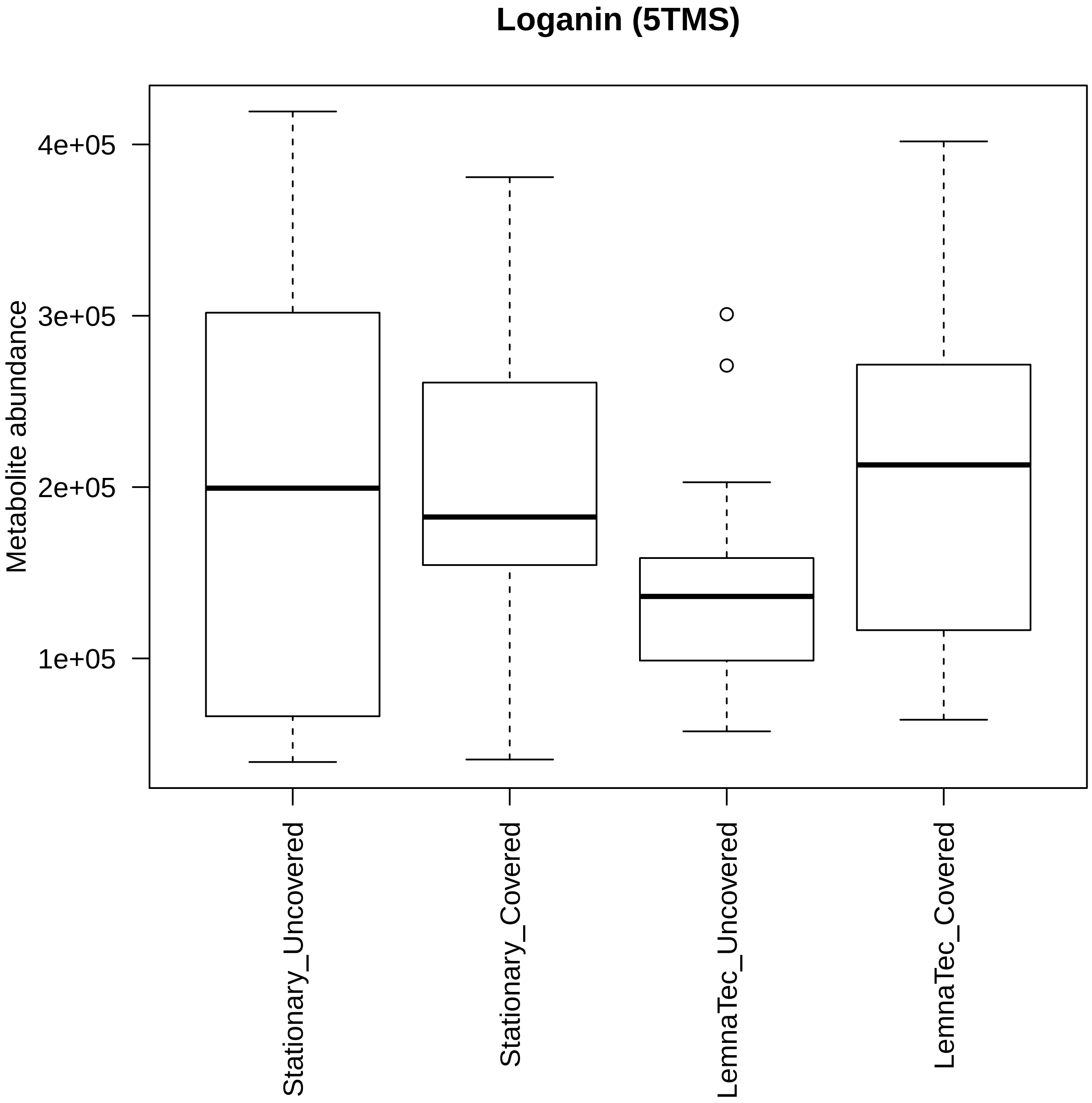
Loganin (5TMS)
Metabolite abundance
4e+05
3e+05
2e+05
1e+05
Stationary_Uncovered
Stationary_Covered
LemnaTec_Uncovered
LemnaTec_Covered

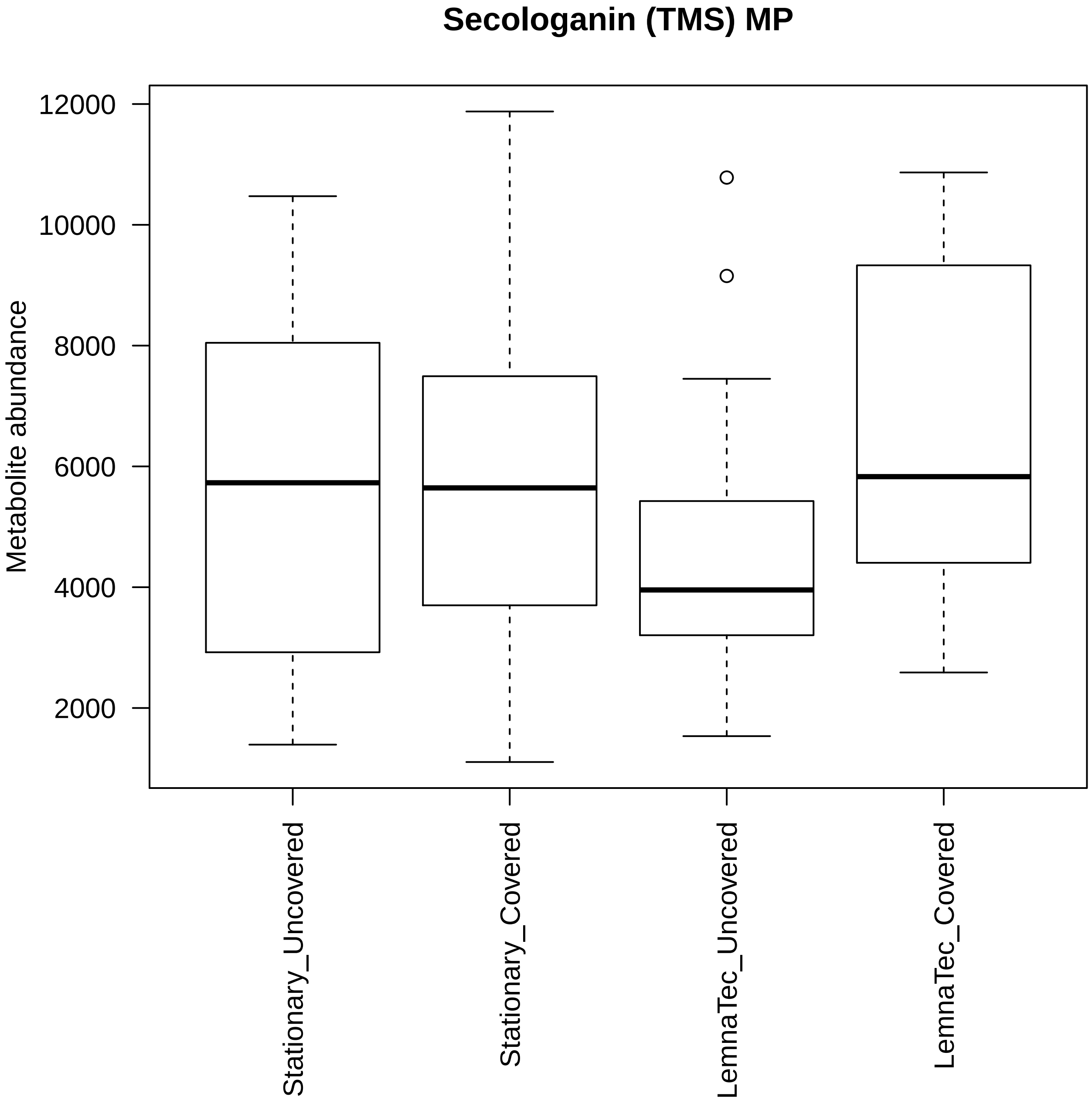
Secologanin (TMS) MP
Metabolite abundance
12000
10000
8000
6000
4000
2000
Stationary_Uncovered
Stationary_Covered
LemnaTec_Uncovered
LemnaTec_Covered

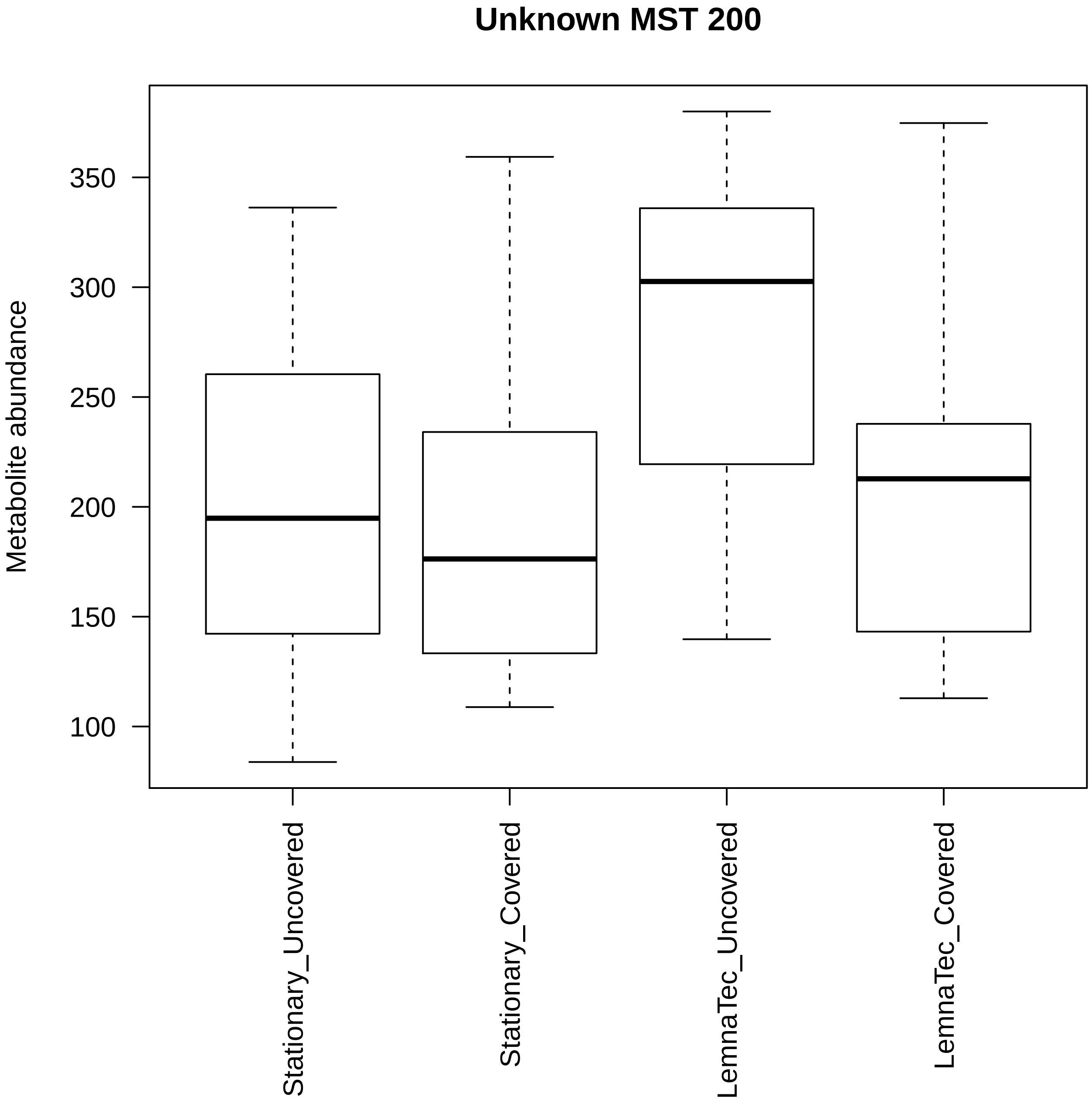

Unknown MST 200
Metabolite abundance
100
150
200
250
300
350
Stationary_Uncovered
Stationary_Covered
LemnaTec_Uncovered
LemnaTec_Covered

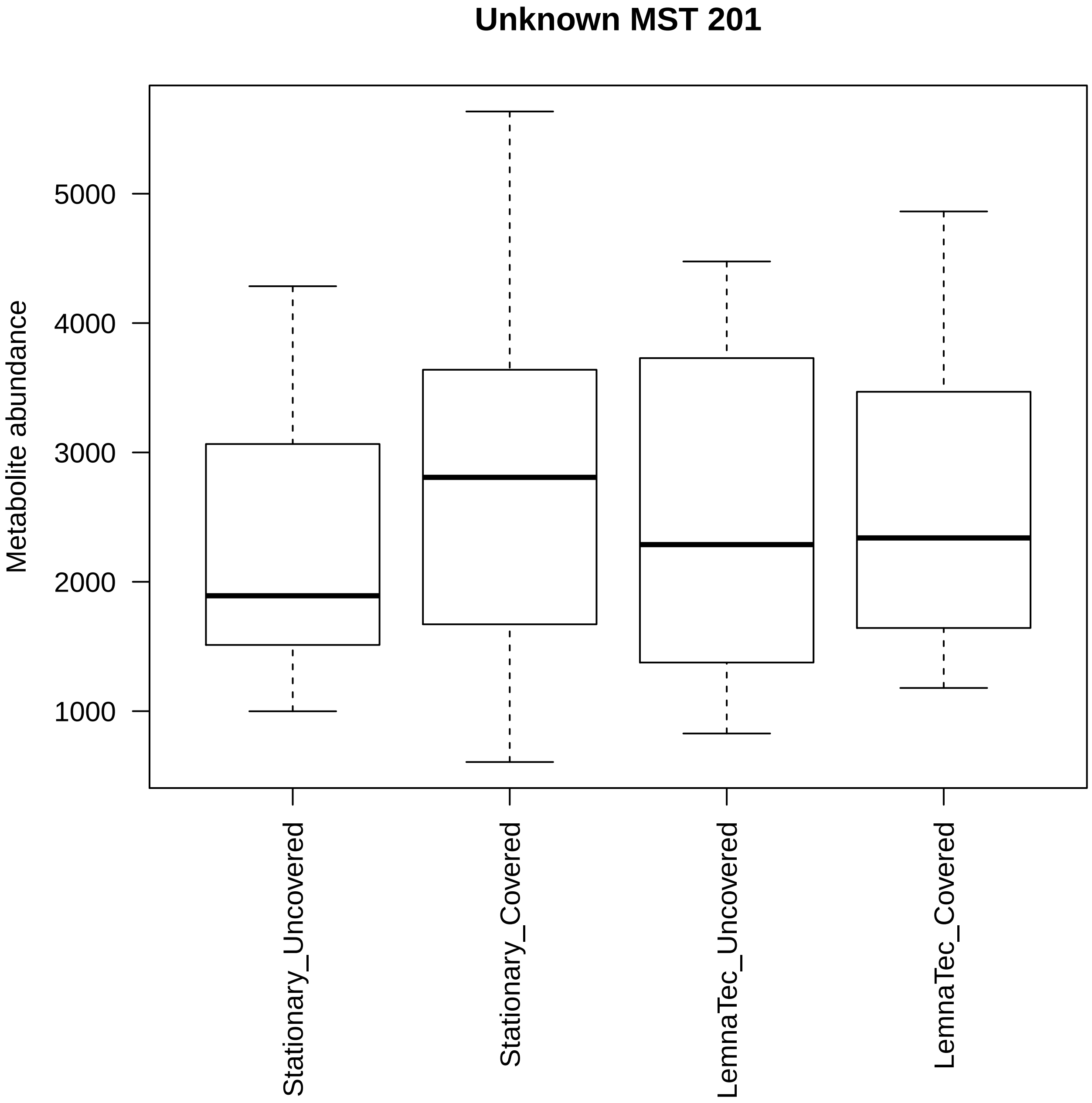
Unknown MST 201
Metabolite abundance
1000
2000
3000
4000
5000
Stationary_Uncovered
Stationary_Covered
LemnaTec_Uncovered
LemnaTec_Covered

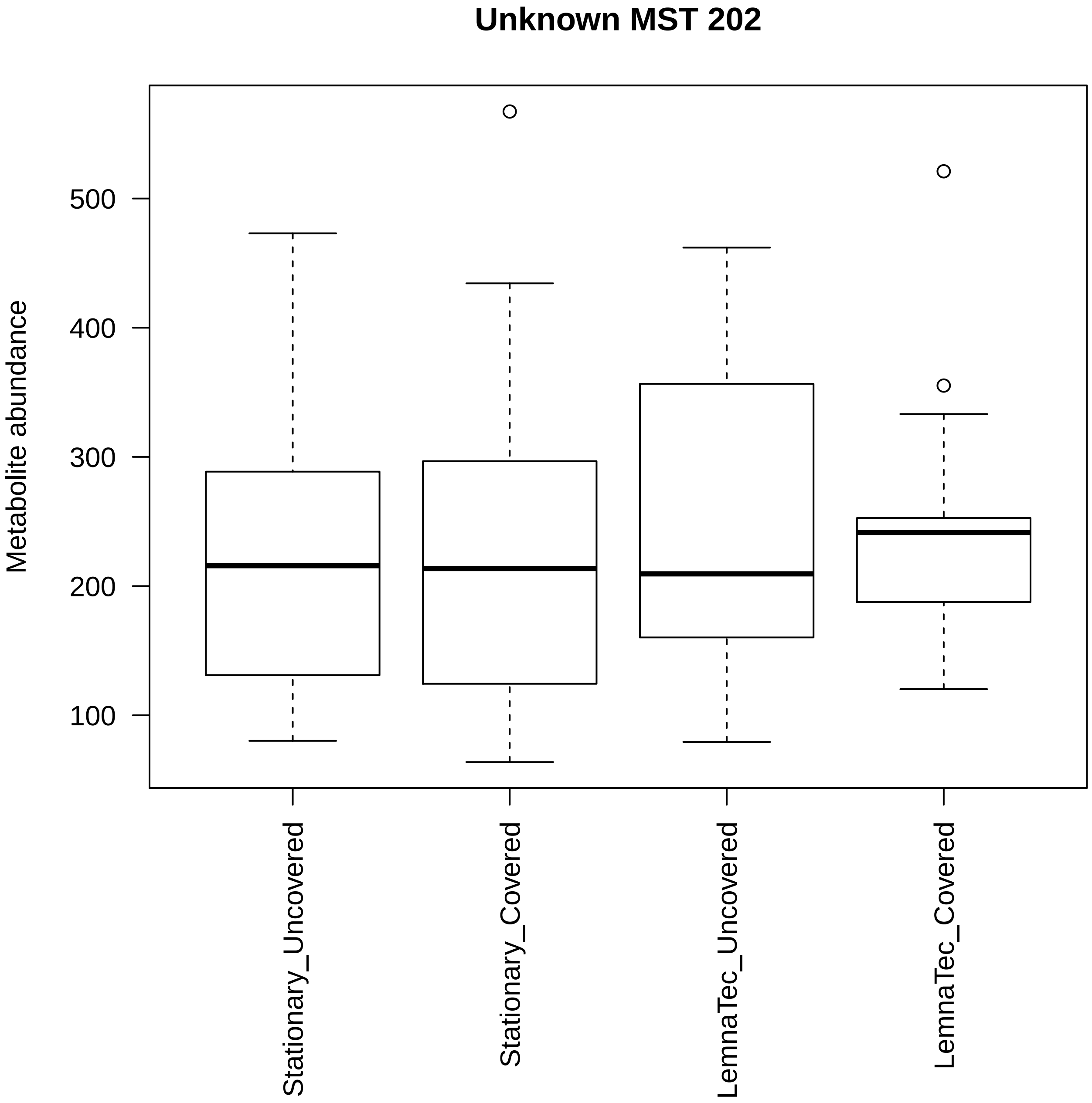

Unknown MST 202
Metabolite abundance
100
200
300
400
500
Stationary_Uncovered
Stationary_Covered
LemnaTec_Uncovered
LemnaTec_Covered

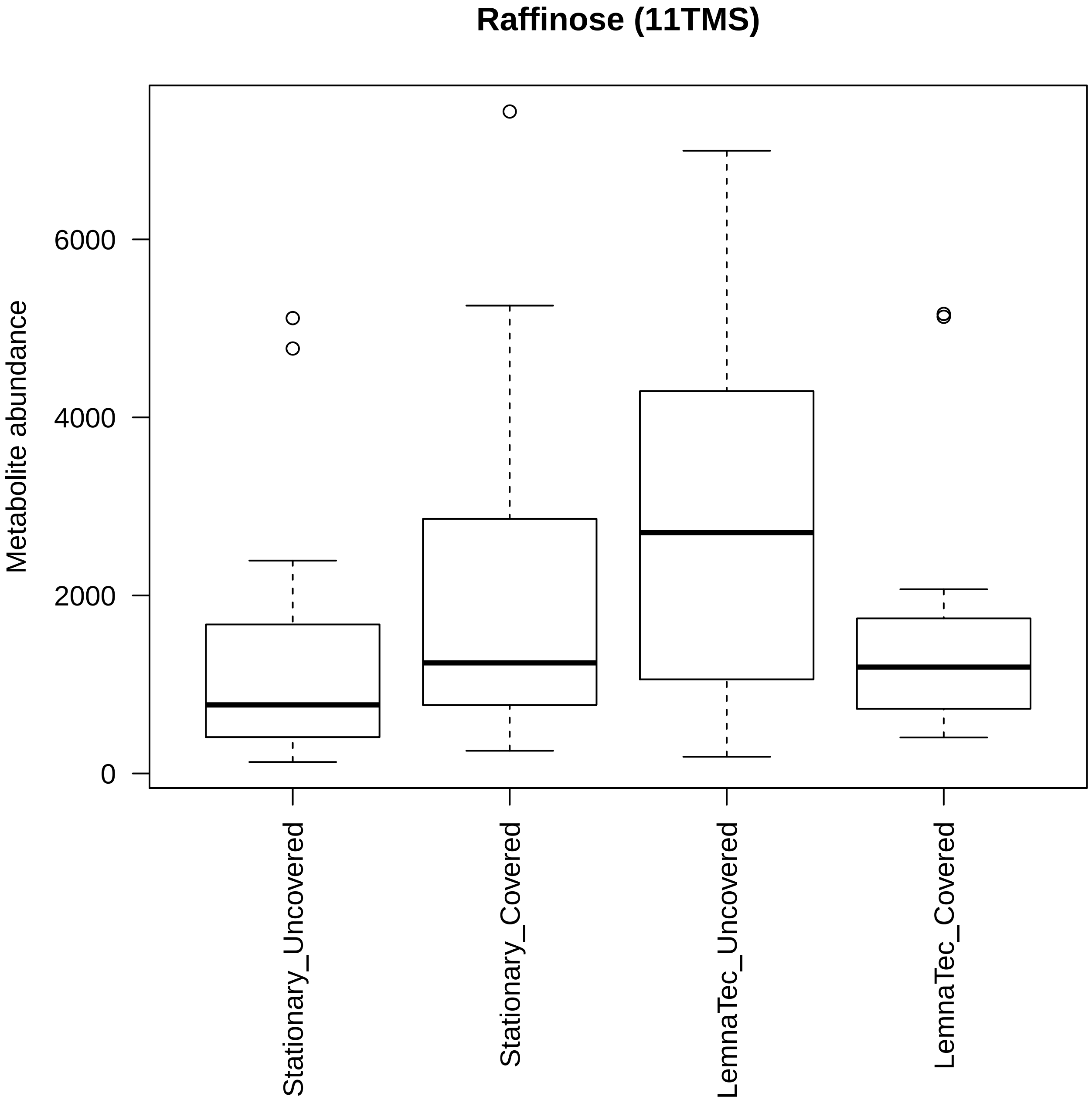

Raffinose (11TMS)
Metabolite abundance
0
2000
4000
6000
Stationary_Uncovered
Stationary_Covered
LemnaTec_Uncovered
LemnaTec_Covered

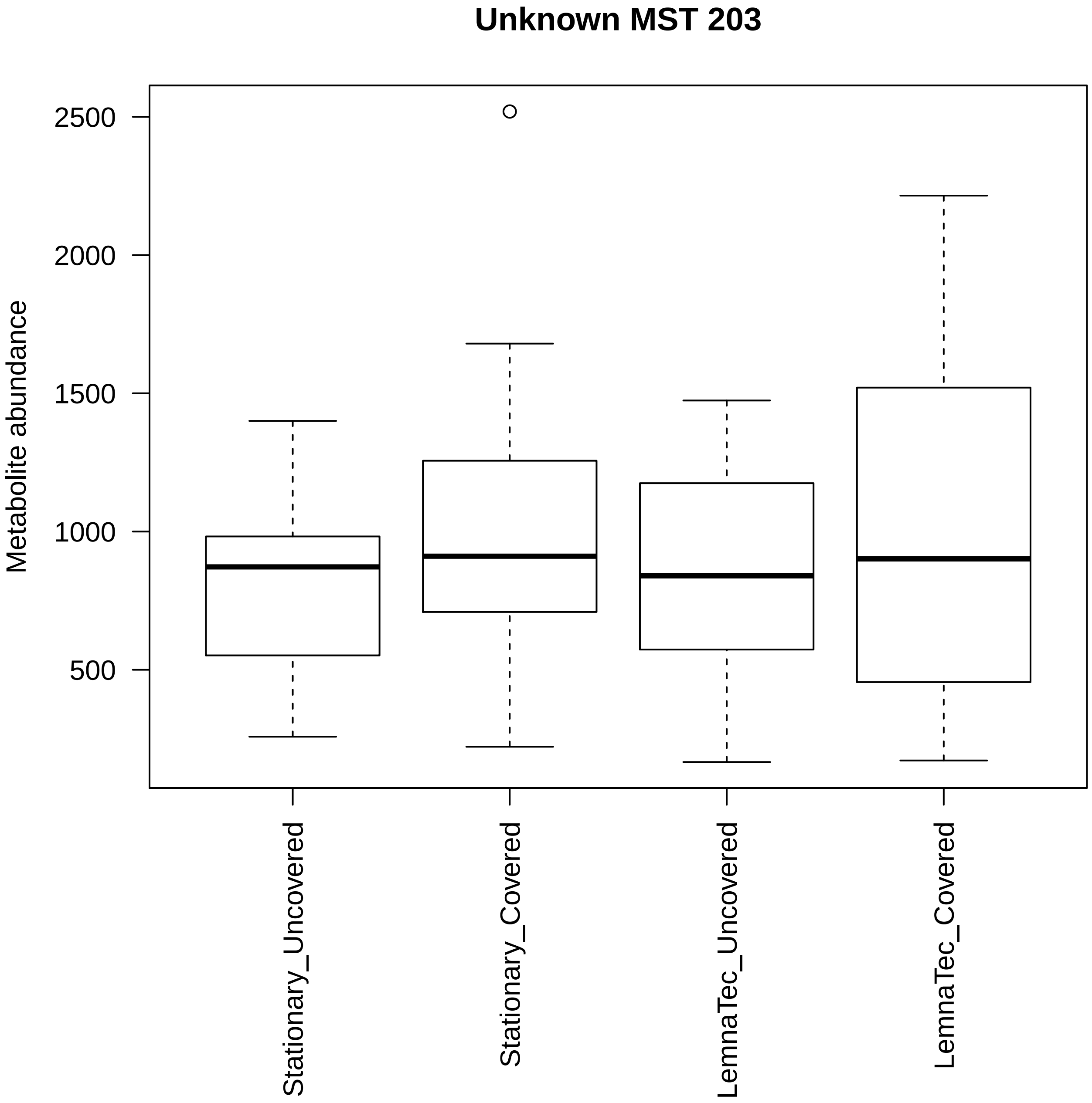
Unknown MST 203
Metabolite abundance
2500
2000
1500
1000
500
Stationary_Uncovered
Stationary_Covered
LemnaTec_Uncovered
LemnaTec_Covered

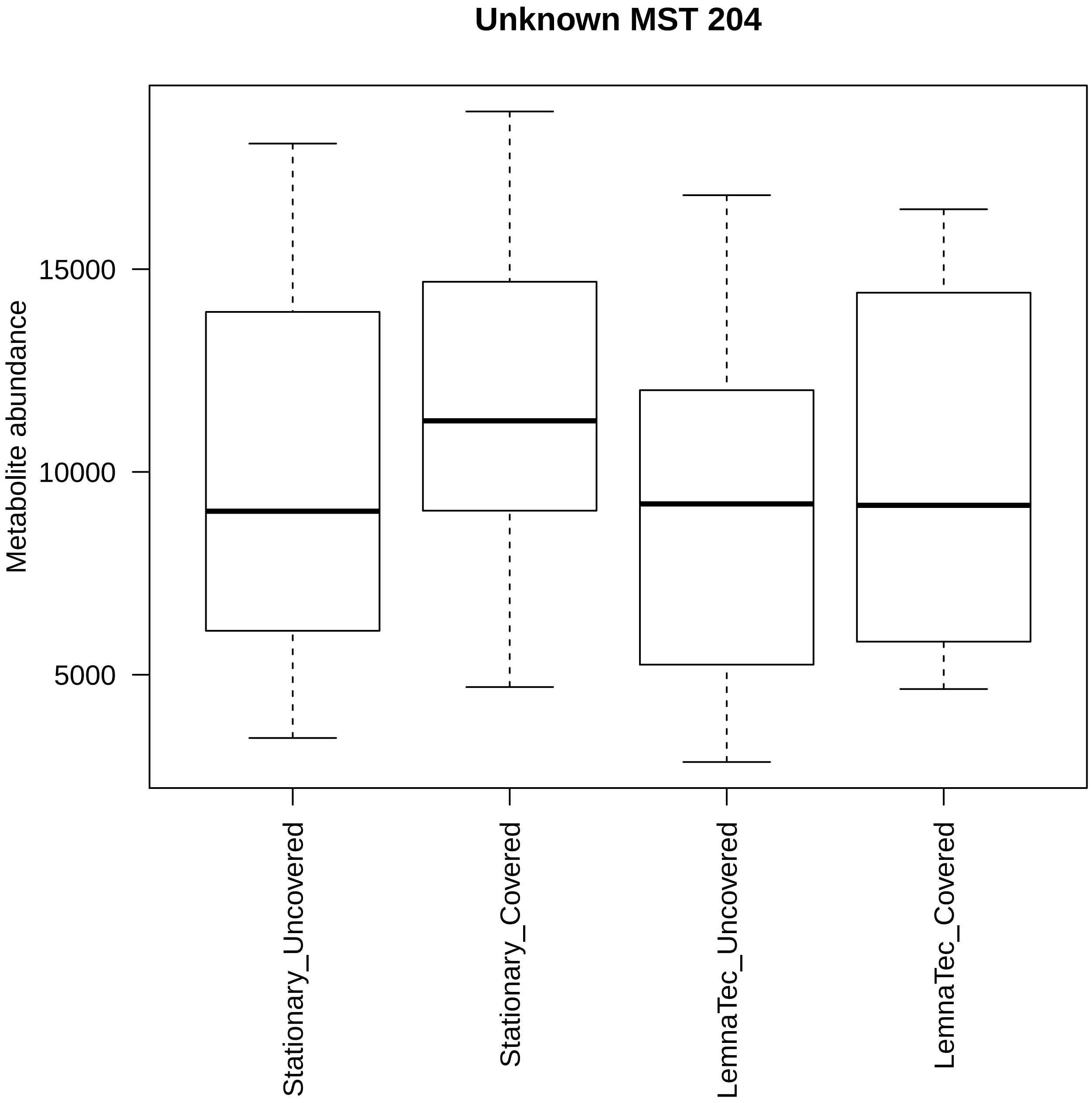

Unknown MST 204
Metabolite abundance
15000
10000
5000
Stationary_Uncovered
Stationary_Covered
LemnaTec_Uncovered
LemnaTec_Covered

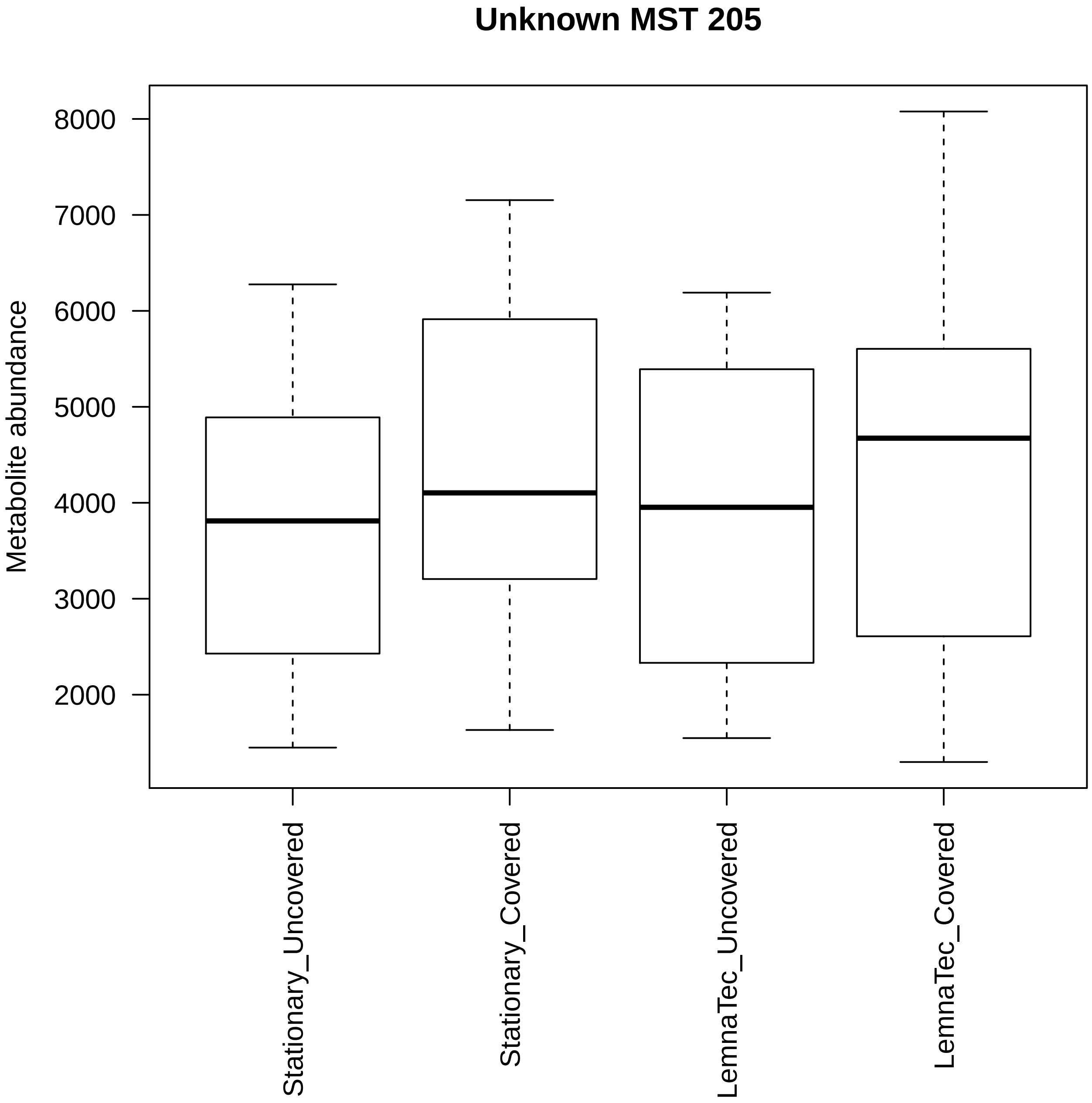

Unknown MST 205
Metabolite abundance
8000
7000
6000
5000
4000
3000
2000
Stationary_Uncovered
Stationary_Covered
LemnaTec_Uncovered
LemnaTec_Covered

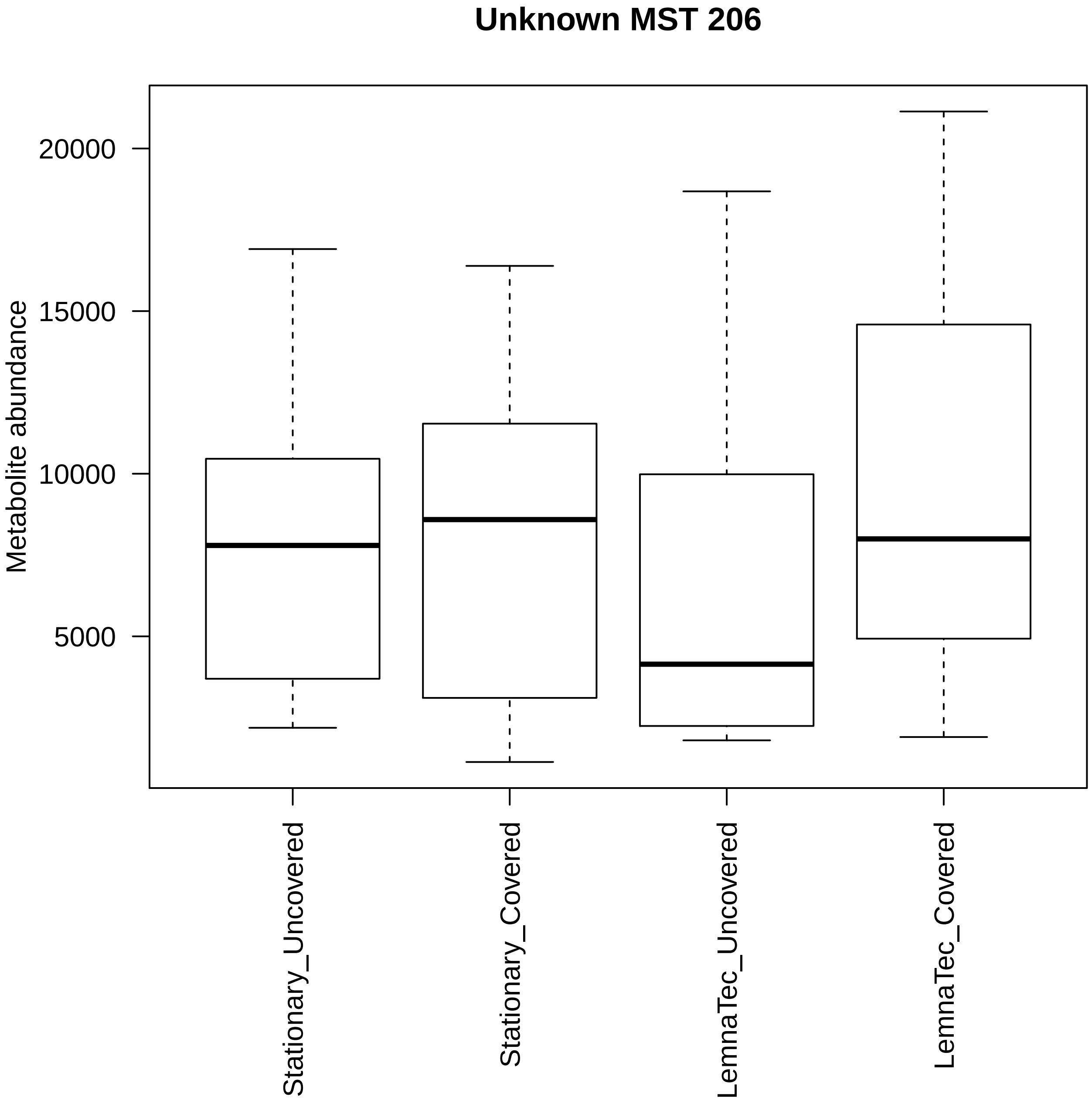

Unknown MST 206
Metabolite abundance
20000
15000
10000
5000
Stationary_Uncovered
Stationary_Covered
LemnaTec_Uncovered
LemnaTec_Covered

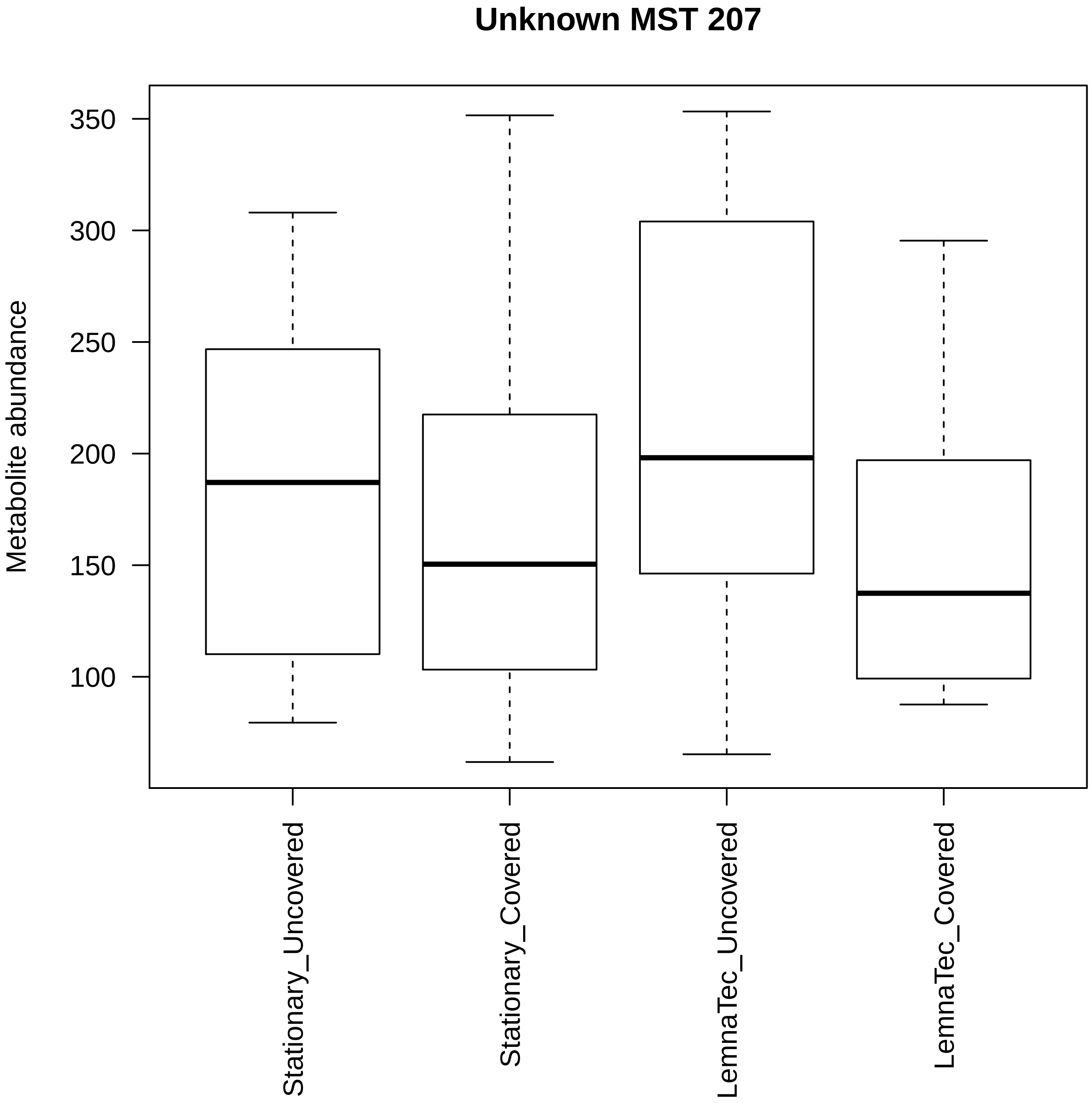
Unknown MST 207
Metabolite abundance
350
300
250
200
150
100
Stationary_Uncovered
Stationary_Covered
LemnaTec_Uncovered
LemnaTec_Covered

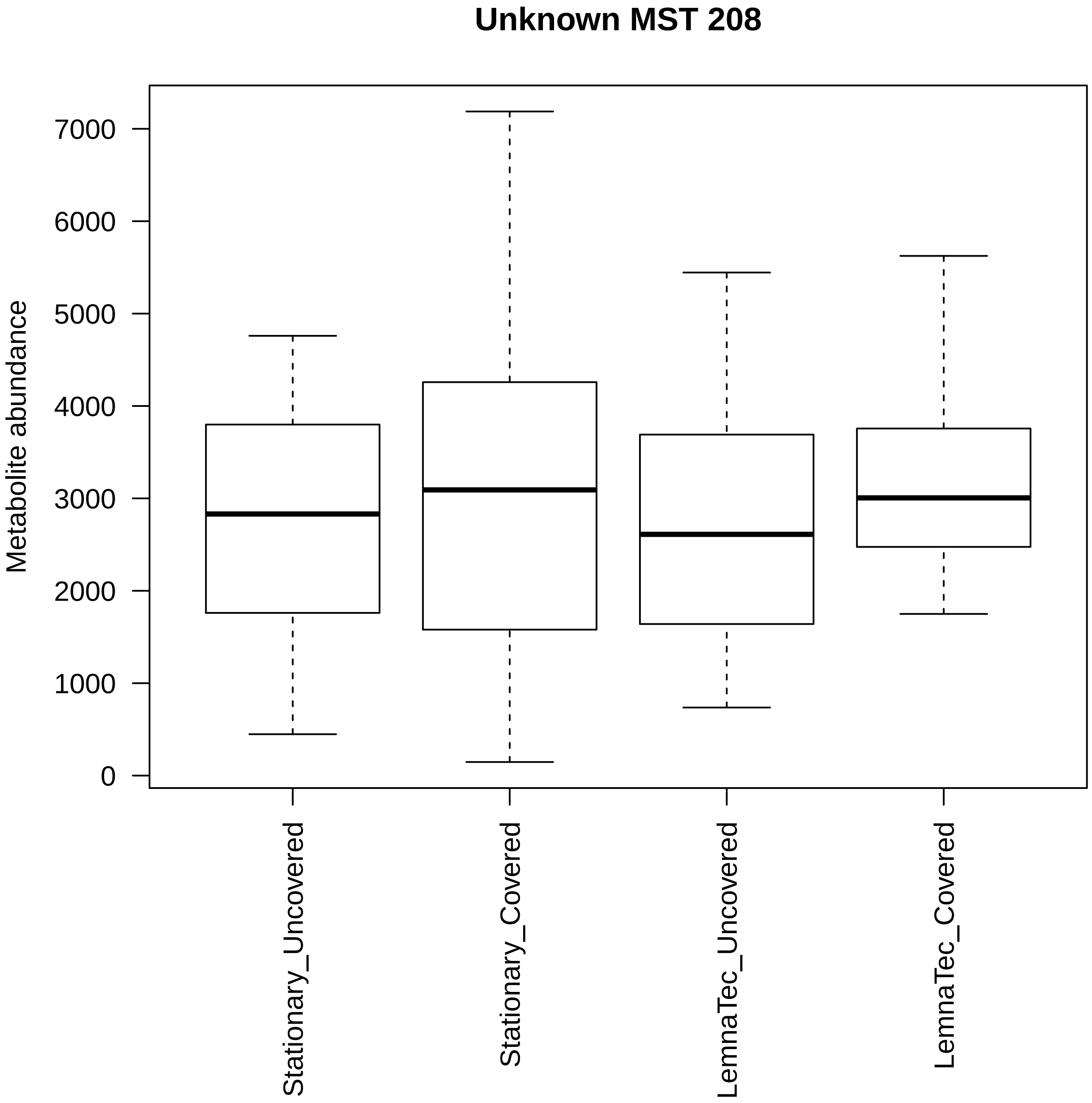

Unknown MST 208
Metabolite abundance
0
1000
2000
3000
4000
5000
6000
7000
Stationary_Uncovered
Stationary_Covered
LemnaTec_Uncovered
LemnaTec_Covered

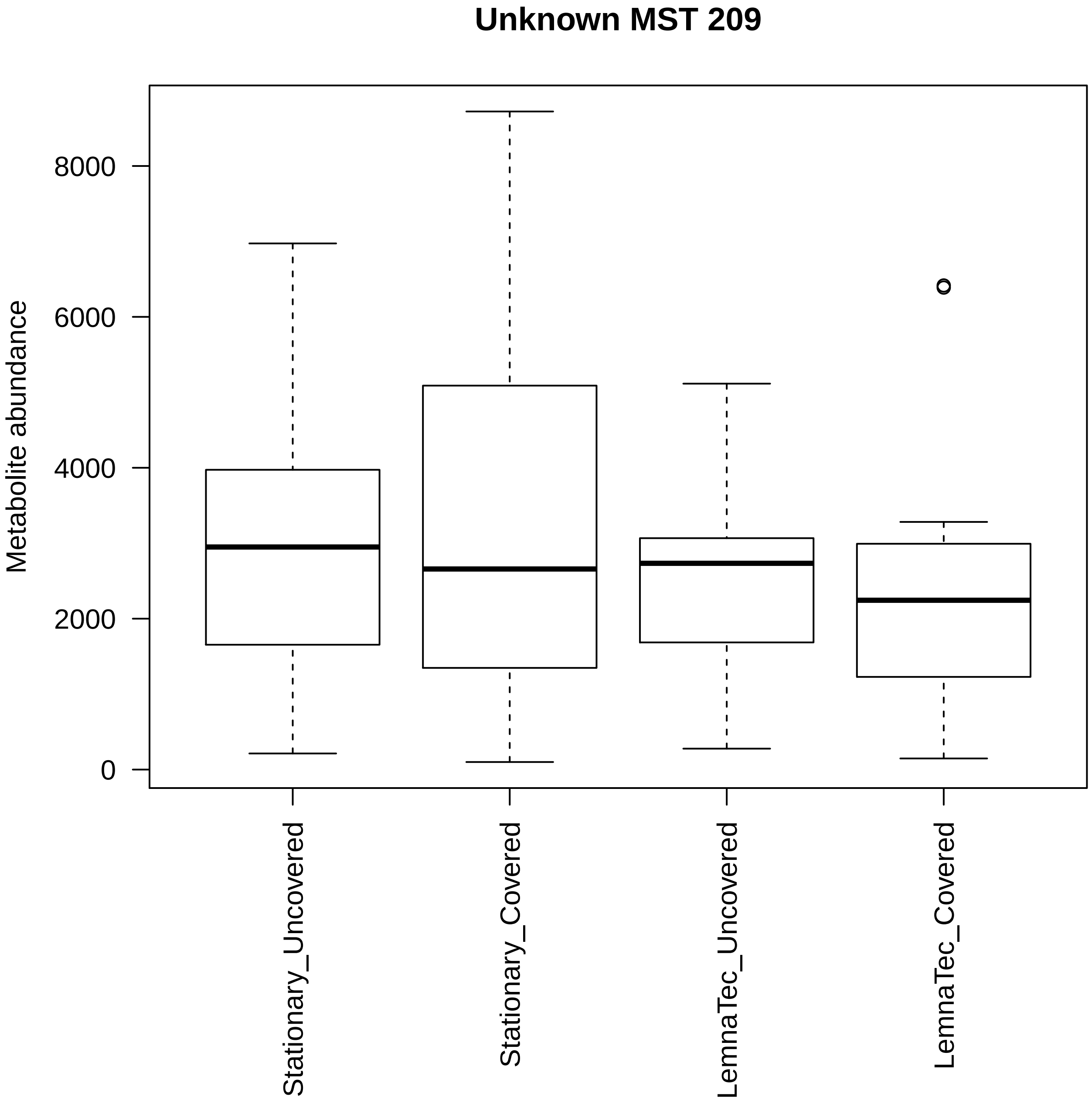

Unknown MST 209
Metabolite abundance
0
2000
4000
6000
8000
Stationary_Uncovered
Stationary_Covered
LemnaTec_Uncovered
LemnaTec_Covered
